# Associations between religiosity and climate change beliefs and behaviours in the Avon Longitudinal Study of Parents and Children (ALSPAC)

**DOI:** 10.1371/journal.pclm.0000469

**Published:** 2025-04-09

**Authors:** Daniel Major-Smith, Isaac Halstead, Jean Golding, Katie Major-Smith

**Affiliations:** 1 Centre for Academic Child Health, Population Health Sciences, Bristol Medical School, University of Bristol, Bristol, United Kingdom; 2 Sustainability, Creativity and Innovation Research Group, Plymouth Marjon University, Plymouth, United Kingdom; 3 School of Psychology, University of Plymouth, Plymouth, United Kingdom

## Abstract

Individual actions are crucial to mitigating the impact of anthropogenic climate change. Understanding the factors shaping individuals’ climate beliefs and behaviours is therefore essential to help encourage sustainable action among the public. One such factor is religion, which – based on theoretical expectations and prior literature – could influence climate beliefs and behaviours, either positively or negatively. To understand the impact of religion in more detail, we used data from two generations of a large-scale longitudinal population-based UK birth cohort study (the Avon Longitudinal Study of Parents and Children; ALSPAC). We explored whether a range of religious/spiritual beliefs and behaviours (religious belief, identity and attendance, in addition to latent classes of religiosity) were associated with a number of climate beliefs and behaviours (e.g., belief in, and concern over, climate change, and pro-environmental actions taken for climate change reasons), adjusted for a range of sociodemographic confounders. Analyses were repeated in three cohorts: the study offspring, their mothers, and the mother’s partners. Overall, we observed a broadly ‘U’-shaped or ‘J’-shaped association between religiosity and climate beliefs and behaviours in the parental generation; participants with intermediate levels of religiosity displayed the lowest levels of belief, concern and behaviours, while the most religious participants displayed similar, and sometimes greater, awareness and actions relative to the least religious. These patterns were not replicated in the offspring generation, with little relationship observed between religion and climate questions. These results indicate a complex association between religion and climate beliefs and behaviours, which varies depending on the religion exposure, the climate outcome, and the generation. The reason for these findings is uncertain, although they perhaps suggest that, among the highly religious in the older generation, religious attendance may promote positive climate beliefs and behaviours.

## Introduction

Human activity is having a dramatic impact on our planet’s climate, and measures to reduce society’s carbon footprint to help mitigate the social and environmental effects of climate change are urgently needed [1]. Although addressing the climate crisis requires high-level systemic change, including stronger governmental action, international collaboration and industry transformation, the behaviour of individuals can collectively contribute to reducing greenhouse gas emissions [2,3]. The behaviours suggested include eating a plant-based diet, avoiding air travel and living car-free [4,5], as well as influencing policy reformation through direct climate action [6].

Many factors shape an individual’s beliefs and behaviours regarding climate change, such as educational attainment [7,8], resource availability [9,10], political affiliation and the wider political landscape [11,12] and experience of climate-related events [13]. In addition to educational, economic and political factors, religion may also play an important role of shaping peoples’ beliefs and behaviours towards climate change. Despite being in general decline in Western societies [14–16], the majority of the world’s population adhere to a religion and it is still central to many peoples’ lives. Religion also has the power to shape individuals’ beliefs and behaviours, including towards the environment [17]. The direction of this association – if any – could be hypothesised to be either positive or negative [18], with previous empirical studies providing conflicting results (discussed below). Note that we focus predominantly on Christianity here largely for pragmatic reasons, as this is where most of the research has been conducted and as most individuals in the current UK study population are Christian (if they have a faith/religion; [19,20]).

There are several lines of evidence which may predict a positive relationship – that is, religious individuals having greater climate awareness and behaviours. This includes a moral responsibility to care about the world, with many religious groups believing that God made humans the ‘custodians’ of nature, and therefore it is our religious duty to care for it. Such perceptions of religiously-informed environmental stewardship have been shown to positively increase belief in, and concerns of, climate change [21] and evoke feelings of environmental guilt which result in greater engagement in pro-environmental actions [22]. Religion may also foster cooperation and prosocial values [23,24], perhaps leading to greater pro-environmental behaviour.

There has also been a push among religious groups in recent years to better acknowledge and address the environmental damage caused by human activity [25]. Religious leaders have been calling for stronger commitments to tackling climate change at the annual UN Climate Change conference, with a recent conference in 2023 (COP28) providing a platform for faith leaders to voice their opinions by hosting the first faith-based pavilion (https://www.unep.org/events/conference/faith-based-engagement-cop28). The Church of England is increasingly engaged in climate issues, including the introduction of commitments to protecting the environment in some dioceses, divesting from fossil fuels, being net-zero, and petitions towards more renewable energy sources (e.g., https://www.theguardian.com/uk-news/2022/jun/22/christians-commit-protecting-environment-oxford-diocese and https://operationnoah.org/articles/more-than-500-church-leaders-write-to-pm-and-chanellor-calling-for-renewables-push/).

The influence of faith leaders on evoking climate action can be great, with Pope Francis’s Encyclical *Laudato Si’* [26] increasing beliefs and concern for climate change among Americans, particularly Catholics [27], and enhancing American’s perceived responsibility for helping tackle the climate crisis [28]. Similar findings were also found after the publication of the Archbishop of Canterbury’s 2020 Lent Book “Saying Yes to Life” [29] which focused on the environmental challenges faced by people globally. After reading this book, UK Christians had an enhanced appreciation of the natural world and greater intentions of performing pro-environmental behaviours, including eating less meat, using more renewable energy and buying more sustainable items [30]. One may therefore predict that religiosity will result in greater environmental and climate awareness and activism.

On the other hand, some religious teachings would appear to go against environmental concerns by emphasising the control that humans have over the natural world, and thus potentially reducing environmental concern and action [18]. This ‘dominion thesis’ has been suggested to have contributed significantly to mass environmental damage in the western world [31], and although it has been claimed that Judeo-Christian religions are becoming more ‘green’ over recent decades [25], evidence for this is somewhat limited [32]. For example, disagreements on religion’s role in addressing environmental issues has been found to inhibit climate action [33], while a review of the environmental action being taken by US Catholic clergy showed that very little is being done to address environmental issues [34]. This limited greening of religion is further emphasised by research reporting little evidence for increased environmental concern among American Christians over the past few decades, while environmental concern actually decreased in some instances [35].

However, it is difficult to determine whether these views are shared among different religious groups and in different regions. For example, most representatives from Evangelical Christian Denominations in Sweden felt that addressing climate change was important and identified themselves as being responsible to take action [36]. Additionally, non-Judeo-Christian religions may have stronger pro-environmental commitments [18], as suggested by an Australian study which found that Buddhists were more likely, and Christians less likely, to endorse climate change beliefs compared to non-religious individuals [37]. One potential reason for this country-level variation is the connection between religious and political identities, particularly in countries such as the US where religious fundamentalism is often associated with conservative, right-wing anti-environmental belief systems, meaning that religious Americans may be less interested in environmental issues compared to religious individuals from other countries [38,39]. Furthermore, climate change is a scientific issue that requires trust towards science and scientists, yet previous research suggests that religious individuals are more likely to hold a negative attitude towards science [40]. However, the relationship between religion and attitudes towards science has been shown to be highly variable between countries, especially compared to the US which has a strong negative association [41].

Given these conflicting theoretical expectations and various country- and religion-level differences, it is perhaps not surprising that there is currently little consensus as to whether religion is positively [17,42], negatively [37], or not at all [43] associated with pro-environmental beliefs and behaviours. A key limitation is that much of this previous work is cross-sectional, meaning that it is difficult to rule out the possibility of reverse causality or whether confounding factors have been adequately controlled for [44,45]; whether these relationships are causal is therefore often unclear. Longitudinal studies with the exposure (here, religion) measured before the outcome (here, climate beliefs and behaviours), and with detailed baseline confounder data, may help overcome some of these limitations of previous research and provide greater evidence for a potential causal interpretation [45]. Many previous studies have also focused on single measures of religiosity and/or climate attitudes/behaviours (e.g., [8,17,37]), potentially overlooking the multidimensional nature of these traits [46]. That is, religious beliefs may have different associations with climate beliefs and behaviours compared to religious behaviours; while the relationship with religion may vary by the specific climate belief or behaviour. For instance, a Christian religious identity has been associated with lower belief in climate change [37], yet overall religiousness has been associated with increased pro-environmental concern [42] and action [17]. Furthermore, much previous research has been conducted in US populations (e.g., [34,35,38,39]); research in other countries is necessary to understand the extent to which findings replicate and are generalisable across nations.

In the current project, we aim to explore these associations in detail in two generations within the Avon Longitudinal Study of Parents and Children (ALSPAC), a UK-based longitudinal birth cohort: the study offspring, the study mothers, and the mother’s partners. Religiosity data were collected in 2019/2020 and have been described previously [19,20], while climate change variables were collected in 2022 (detailed below). Although we make use of longitudinal data, the aim of this paper is primarily descriptive, in that we are exploring whether religiosity is associated with subsequent climate beliefs and behaviours. However, we will adjust for a range of sociodemographic variables to try and remove some key sources of potential confounding. To the extent that this adequately adjusts for all sources of confounding – and assuming no other sources of bias such as selection bias or measurement error [47] – this may reflect a causal effect estimate; we discuss whether the assumptions required for a causal interpretation may plausibly be met in more detail in the discussion. Repeating these analyses in three different cohorts also allows us to explore whether these patterns differ by generation, while also assessing if results replicate across the cohorts. This study will contribute to our understanding of the relationship between religion and climate change beliefs and behaviours in numerous ways, including: i) use of longitudinal data, with detailed baseline confounder data, potentially providing greater evidence for a causal interpretation; ii) using a range of religion variables (i.e., religious belief, identity, service attendance and latent classes of religiosity) and different aspects of climate beliefs (e.g., belief in climate change, concern regarding climate change, engagement in actions for climate reasons) to understand this relationship in more detail; and iii) providing an exploration of these relationships in a UK sample, as less research has been conducted in this population, especially compared to the US.

## Methods

An analysis plan for all analyses reported in this was pre-registered on the Open Science Framework website prior to conducting analyses (https://osf.io/p5vjz/). Other than some minor updates and corrections (see section S1 in S1 Text), the research questions, methods and analyses reported below are identical to those detailed in the analysis plan.

### ALSPAC Study Description

Pregnant women resident in Avon, UK with expected dates of delivery between 1st April 1991 to 31st December 1992 were invited to take part in the study. The initial number of pregnancies enrolled was 14,541, of which there were a total of 14,676 foetuses, resulting in 14,062 live births and 13,988 children who were alive at 1 year of age [48,49]. When the oldest children were approximately 7 years of age an attempt was made to bolster the initial sample with eligible cases who had failed to join the study originally, resulting in an additional 913 children being enrolled. The total sample size for analyses using any data collected after the age of seven is therefore 15,447 pregnancies from 14,833 mothers, resulting in 15,658 foetuses, of which 14,901 were alive at 1 year of age [50,51].

The current research focuses on both ALSPAC generations, the parents (Generation-0; G0) and the study children (Generation-1; G1). When working with parental data, one pregnancy was removed if the mother had two pregnancies enrolled in ALSPAC (to avoid repeated data from the same parent). We also dropped observations for participants who had withdrawn consent for their data to be used or where the study child was not alive at 1 year of age (as parents of such children no longer participated in ALSPAC data collections).

For each mother, we also included their associated partner, usually the father of the study child. Partners/fathers (hereafter ‘partners’) were not formally enrolled into ALSPAC, but were given partner-based questionnaires by the mother (if she had a partner and chose to invite them). This means that partner-based questionnaires may not have been completed by the same partner over time (although numbers of such cases are likely to be relatively small; approx. 5% of all partners); to remove this source of bias, we therefore excluded partners known to have changed identity over the course of the study. In total, 12,113 G0 partners have been in contact with the study by providing data and/or formally enrolling when this started in 2010 (with 3,807 partners currently enrolled; [52]). In total, 14,216 G0 mothers, 10,916 G0 partners and 14,524 G1 offspring were eligible for inclusion in this study, although due to loss-to-follow-up only approximately 30% of participants (20% for G0 partners) had data on climate beliefs and behaviours (see below).

Please note that the study website contains details of all the data that is available through a fully searchable data dictionary and variable search tool: http://www.bristol.ac.uk/alspac/researchers/our-data/. Study data gathered since the G1 offspring were aged 22 were collected and managed using REDCap electronic data capture tools hosted at the University of Bristol [53]. Ethical approval for the study was obtained from the ALSPAC Ethics and Law Committee (IRB00003312) and the Local Research Ethics Committees. Specific approval for the collection of the religion and climate data used in the present study was also given by the ALSPAC Ethics and Law Committee. Informed consent for the use of data collected via questionnaires and clinics was obtained from participants following the recommendations of the Avon Longitudinal Study of Parents and Children Ethics and Law Committee at the time. Full details of the ALSPAC consent procedures are available on the study website (https://www.bristol.ac.uk/alspac/researchers/research-ethics/). For an historical overview of the development of ALSPAC ethics policies and the ALSPAC Ethics and Law Committee, see [54]. Pseudonymised data containing no personally-identifiable information were accessed 16^th^ November 2023.

### Data

#### Religious/Spiritual Beliefs and Behaviours (RSBB) exposures

The religion variables used in this study are displayed in Table S1 in S1 Text, and include three broad measures of religiosity; religious belief (belief in God/a divine power), religious identity (faith/belief system they identify as, including none), and religious attendance (frequency of attendance at a place of worship). These measures were all assessed in late 2019/early 2020, when the study offspring were approximately 28 years of age [19,20]. These measures cover a range of theoretically-relevant religious beliefs and behaviours [46], many of which have been used extensively in previous religion-based research, both in prior ALSPAC publications [55,56] and more broadly [16,23,57,58]. In addition to these variables, we will also use religiosity categories derived by latent class analysis at this time-point (i.e., using multiple religiosity questions to construct latent classes of religiosity, such as “highly religious”, “moderately religious”, “agnostics” and “atheists”; for more details, see [59]).

Given that there are few individuals from religions other than Christianity in this study, we have excluded these individuals from our analyses and focused specifically on individuals who are either Christian (of various denominations, but predominantly [approx. 80%] Church of England/Protestant) or have no religious affiliation. This is because there are so few numbers of ‘other’ religions in this sample – with small numbers of Buddhist, Jewish, Muslim, Hindu, Sikh and ‘other’ (for these ‘others’, see; [60]), together representing around 5% of the total sample – that grouping these disparate religions together is more likely to obscure than to illuminate, especially given that environmental attitudes are likely to vary between different religions [18,37]. Given that we lack statistical power to explore these non-Christian religions in sufficient detail, and that associations with climate beliefs and behaviours plausibly vary by these faiths (meaning they should not be combined together), we therefore focus specifically on Christians and non-believers. We leave it to other studies – with better sources of data on other religions – to explore associations with other faiths and religions in greater detail. While our primary analysis of religious affiliation will focus on Christians as a whole, we also conducted sub-group analyses to explore whether climate beliefs and behaviours vary by Christian denomination (Church of England vs Roman Catholic vs other Christian).

#### Climate beliefs and behaviours outcomes

Questions on climate beliefs and behaviours were collected in late 2021 and early 2022 (G0 data collection from January 2022 to September 2022; G1 data collection from November 2021 to May 2022), and include a range of questions regarding beliefs surrounding climate change and its impact, as well as behaviours undertaken to reduce one’s climate impact. These pro-environmental behaviours comprised 18 actions, including reducing air travel, eating less meat and/or dairy, and reducing household energy use, among others, and whether they were performed due to climate change. See Table S2 in S1 Text for a full summary of the variables used in the present study. Many of these questions were adapted from Bristol City Council’s Quality of Life Survey 2019 [61], with all others – questions 1, 2b, 2c and 4g to 4q – developed by the ALSPAC team. For more information on how these climate change questions were developed and collected, please see the following Data Note [62].

#### Confounders

Although the aim of this paper is primarily to describe associations between religiosity and climate beliefs and behaviours, we will nonetheless adjust for a range of sociodemographic confounders to assess whether associations are independent of these common sources of confounding, and which may provide stronger evidence for a potential causal effect. These sociodemographic variables included age, sex (for G1 offspring), ethnicity, marital/relationship status, urban/rural location, and various measures of SEP (education, occupational social class, income, area-level deprivation and home ownership status), and are described in Table S3 in S1 Text. Note that, although we intended to include ‘ethnicity’ as a confounder in the G0 partners’ analyses, due to the low number of partners with an ‘other than white’ ethnicity its inclusion resulted in a lack of model convergence and uninterpretable results, so this covariate was subsequently excluded from all G0 partner models.

As discussed in the introduction, a further potential confounding factor which may bias associations between religion and climate beliefs and behaviours is political ideology [38,39]. ALSPAC does not have data on political attitudes in the parental G0 generation, but information regarding political views was obtained in G1 offspring at around the time of the 2015 UK general election when the offspring were approximately 22 years old. As part of this questionnaire participants were asked to name the five political issues most important to them, including the economy, education/schools, immigration and LQBTQ+ rights, among others. Using these variables, we performed a principal component analysis to extract components of political ideology from these nine questions (note that we excluded the variable referring to ‘the environment’, as this is a proxy for our outcome and could result in bias if included). We first performed a parallel analysis, using a tetrachoric correlation between the binary variables, to inform the number of principal components to extract.

**Fig 1:**
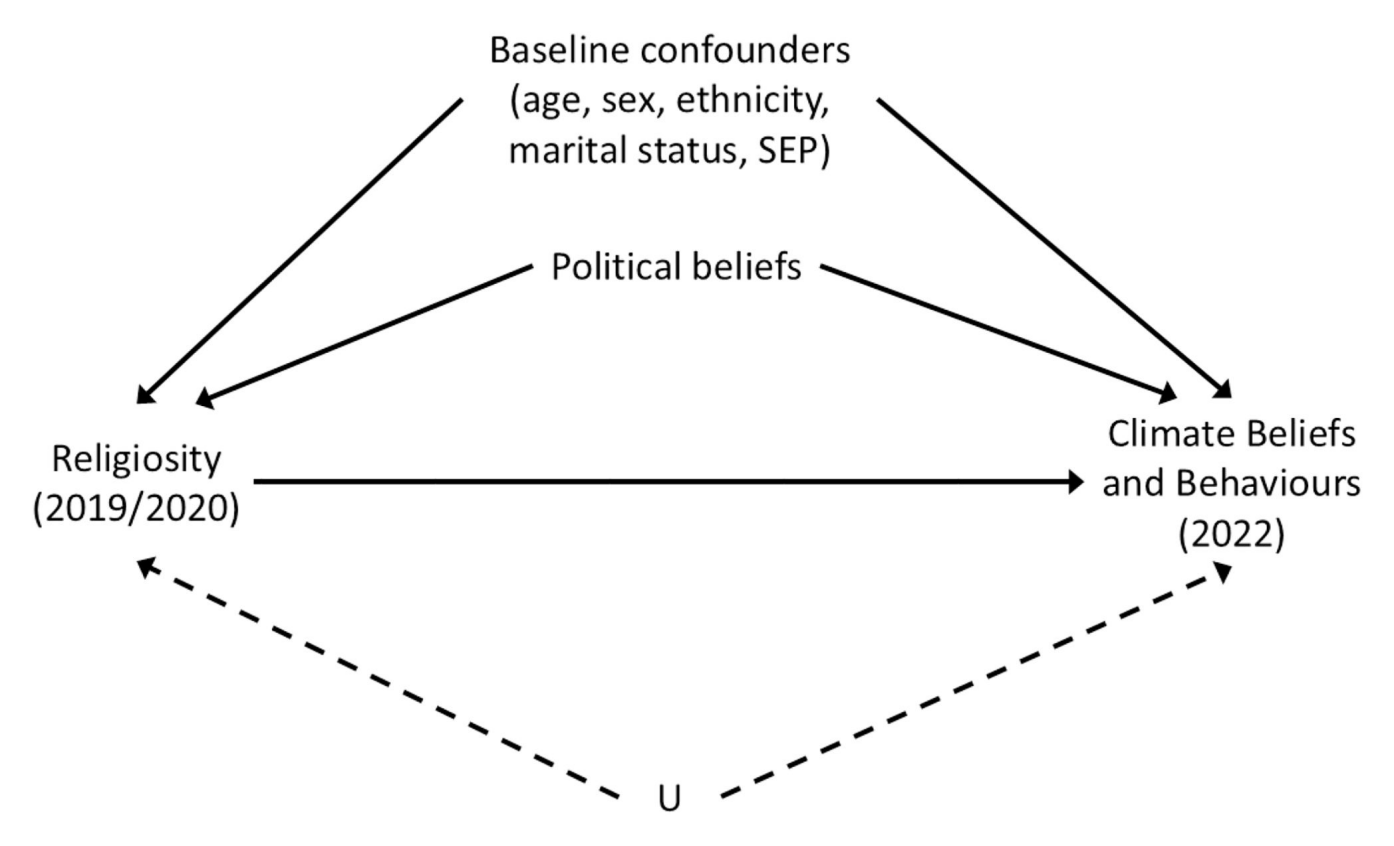
Directed Acyclic Graph (DAG) denoting the assumed causal structure of the data. For simplicity, all potential confounders from Table S3 have been grouped together in the ‘Baseline confounders’ node. The node ‘U’ represents potential unmeasured confounding. Note that data on ‘political beliefs’ are only available in the G1 offspring generation. https://doi.org/10.1371/journal.pclm.0000469.g001

Visual inspection of the scree plot (Fig S1 in S1 Text), and examination of the interpretability of the resulting components, indicated that a two-factor solution was a good fit to the data. We therefore performed a principal components analysis to extract two factors using promax rotation (Table S4 in S1 Text). The first principal component was interpreted as a broadly ‘conservative-liberal’ dimension, with positive factor loadings for concern about the economy, immigration and crime (traditionally more ‘conservative’ concerns) and negative loadings for LQBTQ+ and women’s rights (traditionally more ‘liberal’ concerns). The second component was interpreted as reflecting concern over ‘social issues’, with positive factor loadings for concern about education and the NHS (UK National Health Service). The first principal component explained 22% of the variance, with the second principal component explaining 16% (together explaining 38%); there was little correlation between the components (*r* = 0.01). Both of these principal components were used as additional confounders to try and remove this potential source of bias. As this political ideology variable was only available for G1 offspring, we performed sensitivity analyses comparing models with all variables in Table S3 in S1 Text as confounders (allowing comparability with the G0 results) against models with all variables in Table S3 *plus political ideology components* as confounders (allowing the inclusion of political beliefs as an additional confounder). The Directed Acyclic Graph (DAG) in Fig 1 illustrates the hypothesised causal structure of the data.

### Analysis

We performed analyses for all combinations of religiosity (Table S1 in S1 Text) and climate belief and behaviour variables (Table S2 in S1 Text) to assess whether religion was associated with climate beliefs and behaviours, in both unadjusted and adjusted models (with adjustment made for all potential confounders detailed in Table S3 in S1 Text). The specific regression model depended on the outcome variable (e.g., ordinal regression for ordered categorical outcomes [with the assumption of proportional odds tested by a Brant test]; multinomial regression for unordered categorical variables; and Poisson models for count data). For multinomial models, we used a likelihood ratio test to assess whether there was an overall association between the exposure and outcome. For all other models with three or more levels of the RSBB exposure variable, we used a post-estimation hypothesis test to examine whether there was an overall association between the exposure and outcome. As parameters from generalised linear models are not necessarily intuitive to interpret, we used predicted probabilities and estimated marginal effects from the adjusted models to assist interpretation of these results.

The ‘total number of actions taken for climate reasons’ score was initially modelled using Poisson regression. However, although these data are technically count data (i.e., number of actions), we also used a linear model to explore how robust these results were to different model specifications and because linear regression models are easier to interpret. The distribution of climate actions also had an excess of zeros (i.e., participants who reported engaging in no climate actions), meaning that the Poisson models may also have been mis-specified; we therefore also performed zero-inflated Poisson regressions to model both the count data and the excess zeros.

Analyses were repeated for each of the three cohorts (G0 mothers, G0 partners, and G1 off-spring). Additional analyses in the G1 offspring, exploring whether the inclusion of political ideology as an additional confounder alters results, were also conducted, as described above. Note that throughout this paper *p*-values are interpreted as continuous measures of evidence against the null hypothesis of no association between the exposure and outcome, rather than being arbitrarily dichotomised as either ‘significant’ or ‘non-significant’ [63]. Analyses were conducted in R 4.3.1 [64].

## Results

### G0 Mothers

#### Descriptive statistics

4,562 mothers completed the questionnaire containing the climate questions (32.1% of total 14,216 sample), with a mean age of 59.8 years (SD = 4.26; min = 47; max = 74). In the full sample, 13% of mothers had a degree-level education, 13% were from the most deprived neighbourhood quintile, and 97.5% were of White ethnicity. In the complete-case sample, however (which only includes participants with fully-observed confounder data, any RSBB exposure data and any climate outcome data; *n* = 2,578 [18.1% of full sample]), 23% had a degree, 4% were from the most deprived areas, and 98.8% were of White ethnicity. For full details on these sociodemographic characteristics and other confounders, in both the full and complete-case samples, see Table S5 in S1 Text.

RSBB exposures in the full sample are in Table 1; 43% of mothers believed in God/a divine power, 71% identified as Christian (three-quarters of whom were Church of England) while 14% regularly attended a place of worship (with results of the complete-case sample in Table S6 in S1 Text; descriptive statistics are similar between these two samples).

Descriptive statistics for the climate beliefs and total number of pro-environmental actions performed are in Table 2 (with the individual actions in Table S7 in S1 Text; results are similar in the complete-case sample [Table S8 in S1 Text]). Only 1% of G0 mothers believed that the climate was ‘definitely not’ or ‘probably’ not changing, with 93% of participants ‘some-what’ or ‘very’ concerned about climate change and 1% believing that humans were ‘not at all’ responsible for climate change. On average, G0 mothers reported engaging in 5.7 of 16 pro-environmental actions for climate reasons, although a substantial number of participants (14%) engaged in zero actions (Fig S2 in S1 Text).

#### Climate beliefs and concerns

**Fig 2:**
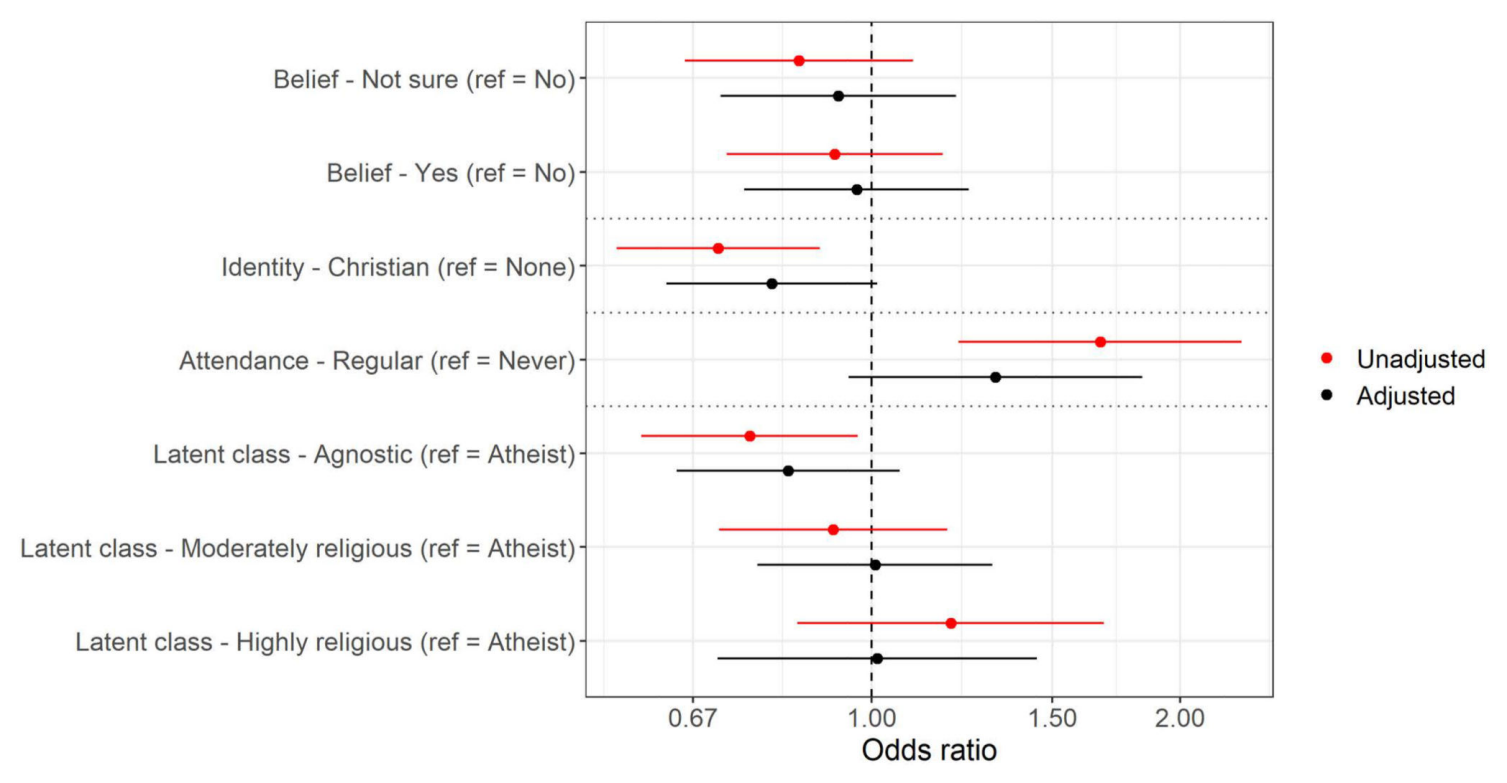
Results of the mothers ordinal regression models with ‘belief that the climate is changing’ as the outcome for four religious exposures (belief [*n* = 2,569], identity [*n* = 2,542], attendance [*n* = 2,542], and latent classes [*n* = 2,575]; models are separated by dashed horizontal lines). Odds ratios above 1 indicate an increased belief in climate change. See Table S9 in S1 Text for full results. https://doi.org/10.1371/journal.pclm.0000469.g002

In adjusted ordinal regression models there was little evidence that religious belief or religiosity latent classes were associated with believing that the climate is changing (Table S9 in S1 Text & Fig 2). There was, however, weak evidence that mothers with a Christian religious affiliation were less likely to believe the climate is changing (odds ratio [OR]=0.800, 95% confidence interval [CI]=0.631 to 1.014, *p*=0.0645), while those who attended religious services regularly were slightly more likely to believe (OR=1.321, 95% CI=0.950 to 1.836, *p*=0.0978). To aid inter*p*retation of these models, when converted to predicted probabilities of the outcomes, 79% of mothers who identified as ‘Christian’ were predicted to respond ‘yes, definitely’ vs 83% of those with no religious affiliation; for religious attendance, the predicted probabilities were 84% for regular attendees vs 80% for occasional/non-attendees (Fig S3 in S1 Text). When Christian denominations were separated, mothers with a Church of England identity were marginally less likely than Catholics to believe in climate change (Table S9 & Fig S4 in S1 Text).

Regarding ‘concern over the impact of climate change’, mothers who believed in God/a divine power (OR=0.835, 95% CI=0.720 to 1.088, *p*=0.0691) and who identified as Christian (OR=0.779, 95% CI=0.652 to 0.930, *p*=0.0057) were less likely to report concern compared to non-religious mothers (e.g., Christians were 6%-points less likely to answer ‘very concerned’). No such negative association was reported for those who regularly attended religious services (OR=1.140, 95% CI=0.908 to 1.432, *p*=0.2580; Table S10 & Fig S5, plus Figs S6 & S7 for predicted probabilities, in S1 Text). For the latent classes, mothers classified as “agnostic” displayed less climate concern compared to “atheists” (OR=0.816, 95% CI=0.669 to 0.996, *p*=0.0454), although no such difference was reported for “highly religious” mothers (OR=0.937, 95% CI=0.722 to 1.217, *p*=0.6276).

**Fig 3:**
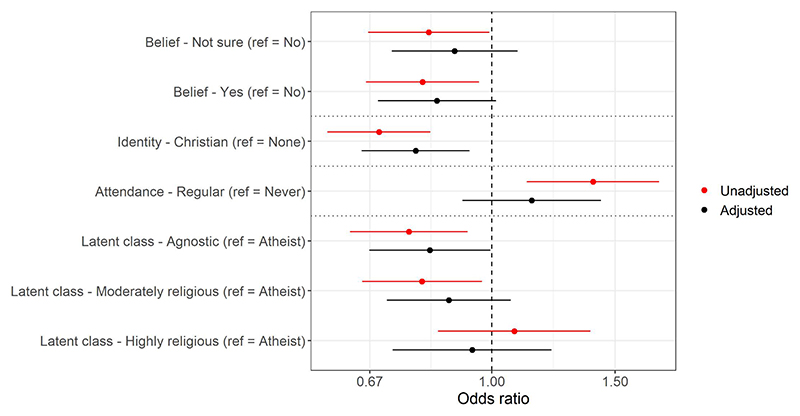
Results of the mothers ordinal regression models with ‘believes that humans are to blame for climate change’ as the outcome for four religious exposures (belief [*n* = 2,561], identity [*n* = 2,535], attendance [*n* = 2,535], and latent classes [*n* = 2,567]; models are separated by dashed horizontal lines). Odds ratios above 1 indicate an increased belief that humans are to blame for climate change. See Table S11 in S1 Text for full results. https://doi.org/10.1371/journal.pclm.0000469.g003

More pronounced differences were observed for the next outcome, with all religious exposures – other than religious attendance – associated with being less likely to endorse that humans are to blame for climate change (Table S11 & Fig 3, plus Figs S8 & S9 for predicted probabilities, in S1 Text). For instance, taking the religious latent classes, 28% of “atheist”, 24% of “agnostic”, 22% of “moderately religious” and 23% of “highly religious” mothers were predicted to respond ‘yes, for all of it’ to this question. Religious mothers were also more likely than non-religious mothers (across all religious exposures) to believe that personal actions will make a difference to long-term climate change (Table S12 & Fig S10, plus Figs S11 & S12 for predicted probabilities, in S1 Text). As an example, 61% of mothers who did not believe in God/a divine power were predicted to answer ‘yes’ to this question, compared to 69% of mothers who believed in God and 65% who were not sure.

#### Climate behaviours

**Fig 4:**
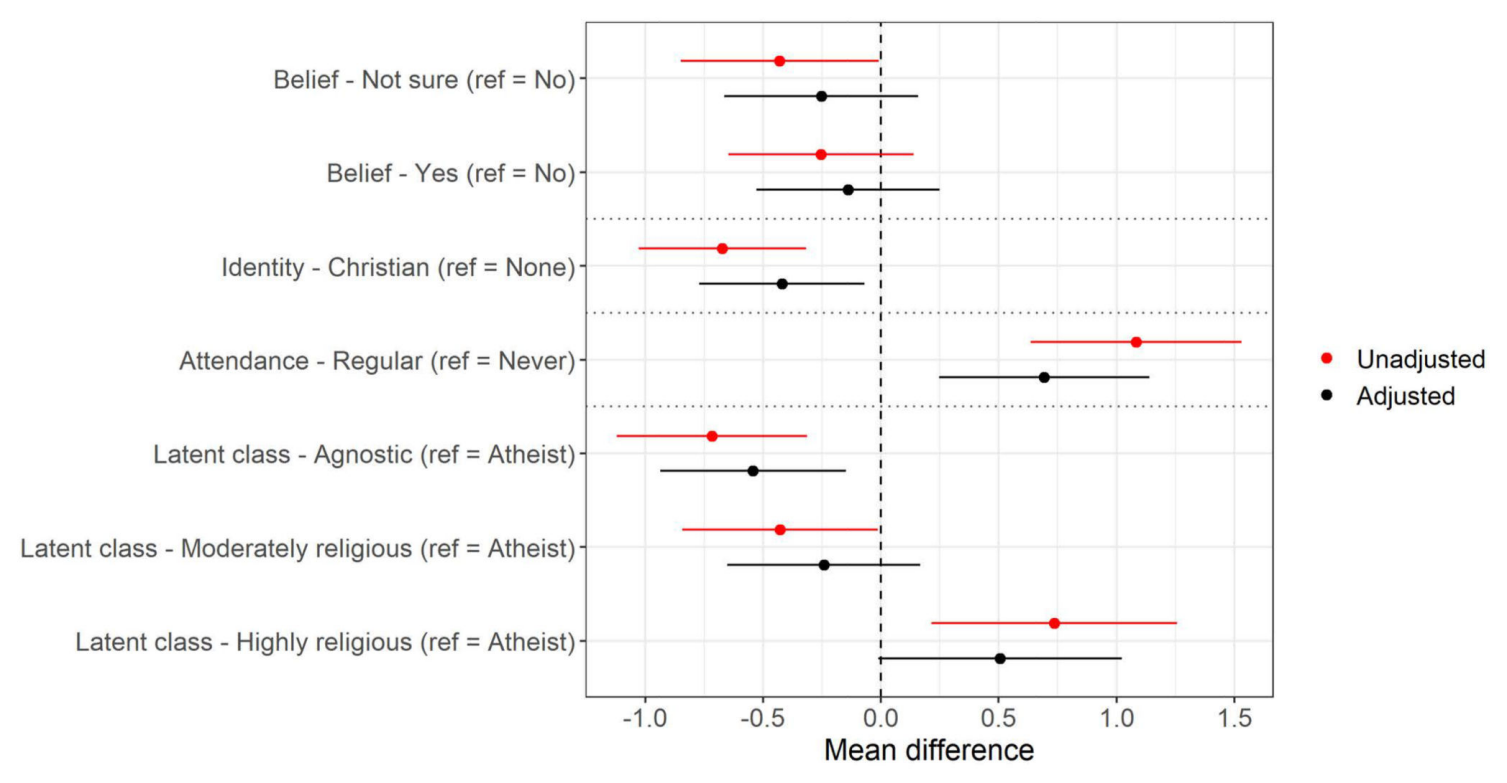
Results of the mothers linear regression models with ‘total number of actions performed due to climate change’ as the outcome for four religious exposures (belief [*n* = 2,218], identity [*n* = 2,195], attendance [*n* = 2,197], and latent classes [*n* = 2,224]; models are separated by dashed horizontal lines). Values above 0 indicate an increased number of pro-environmental actions performed. See Table S13 in S1 Text for full results. https://doi.org/10.1371/journal.pclm.0000469.g004

Turning next to the total number of climate actions performed for climate change reasons, there was a complex pattern of associations with the religious exposures. There was little association between religious belief and the number of actions (*b*_NotSure_=-0.253, 95% CI=-0.664 to 0.159, *p*=0.2285; *b*_Yes_=-0.140, 95% CI=-0.529 to 0.249, *p*=0.4797), while Christian participants engaged in fewer actions (*b*=-0.421, 95% CI=-0.772 to -0.069, *p*=0.0190) and those who attended religious services regularly engaged in more actions (*b*=0.693, 95% CI=0.247 to 1.139, *p*=0.0024). The latent class results may help understand these results, as a ‘J’-shaped pattern emerged; relative to “atheists”, “agnostics” engaged in fewer actions (*b*=-0.543, 95% CI=-0.938 to -0.148, *p*=0.0071), “moderately religious” mothers engaged in slightly fewer actions (*b*=-0.243, 95% CI=0.652 to 0.166, *p*=0.2446), while “highly religious” mothers engaged in more actions (*b*=0.505, 95% CI=-0.012 to 1.022, *p*=0.0555; Fig 4, plus Table S13, with predicted number of behaviours in Fig S13, in S1 Text).

When exploring the Christian denominations separately, mothers with a Church of England identity, but not other Christian denominations, engaged in fewer climate actions (Table S13 & Fig S14 in S1 Text). Patterns of results were qualitatively identical when using alternative Poisson and zero-inflated Poisson models (Tables S14 & S15 and Figs S15-S20 in S1 Text), and when using a reduced number of environmental actions excluding ones which may be prohibitively costly (Tables S16-S18 and Figs S21-S29 in S1 Text). Results were broadly comparable when these actions were analysed individually, albeit with some variability between actions (Table S19 and Figs S30-S77 in S1 Text).

### G0 Partners

1,919 partners completed the questionnaire containing the climate questions (17.6% of total 10,916 sample), with a mean age of 62.4 years (SD = 5.04; min = 43; max = 83). In the full sample, 20% of partners had a degree-level education, 10% were from the most deprived neighbourhoods, and 97.1% were of White ethnicity. As with G0 mothers, the complete-case sample (which only includes participants with fully-observed confounder data, any RSBB exposure data and any climate outcome data; *n* = 1,126 [10.3% of full sample]) was skewed towards partners with a degree (37%) and a White ethnicity (99.4%), and away from those living in the most deprived areas (2%; Table S20 in S1 Text). Partners were less religious than the mothers, with lower rates of religious belief (30% vs 43%), identity (58% vs 70%) and regular service attendance (12% vs 14%; Table 1; complete-case results in Table S6 in S1 Text). Descriptive statistics for climate beliefs and behaviours were similar in partners and mothers (Table 2; with individual actions in Table S7, and complete-case results in Table S8, in S1 Text), although partners were more likely to attribute all of climate change to human activity (29% vs 25%), less likely to think that personal actions will impact long-term climate change (59% vs 66%) and engaged in fewer pro-environmental behaviours for climate reasons (5.1 [out of 16] vs 5.7 in mothers); a substantial minority of partners (21%) also reported engaging in zero actions for climate reasons (Fig S78 in S1 Text).

As the associations between RSBB and climate beliefs and behaviours amongst partners were broadly similar to those in the mothers reported above, only a quick summary of results will be presented here. There was a ‘J’-shaped relationship with belief in climate change, with less religious individuals professing less belief in climate change than non-religious participants, but more religious individuals showing greater belief (Table S21 & Figs S79-S81 in S1 Text), with similar patterns identified for concern over climate change (Table S22 & Figs S82-S84 in S1 Text). Overall, religious partners were less likely to attribute climate change to human activities (Table S23 & Figs S85-S87 in S1 Text), while being more likely to endorse that individual actions can make a difference to climate change (Table S24 & Figs S88-S90 in S1 Text). As with the mothers, a ‘J’-shaped association was reported regarding the number of actions taken for climate reasons, with “atheists” performing more actions than “moderately religious” participants, but fewer than ‘“highly religious” individuals (Table S25 & Figs S91-S93 in S1 Text; results were comparable when using Poisson and zero-inflated Poisson models [Tables S26 & S27 and Figs S94-S99 in S1 Text], when using a reduced number of pro-environmental behaviours [Tables S28-S30 and Figs S100-S108 in S1 Text], and when exploring each action individually [Table S31 & Figs S109-S156 in S1 Text]).

### G1 offspring

4,092 offspring completed the questionnaire containing the climate questions (28.2% of total 14,524 sample), with a mean age of 29.8 years (SD = 0.63; min = 28; max = 31). In the full sample, 49% of offspring were female, 95.1% were of White ethnicity, and 16% were from the most deprived neighbourhoods. The complete-case sample (which only includes participants with fully-observed confounder data, any RSBB exposure data and any climate outcome data; *n* = 1,100 [7.6% of full sample]) was skewed towards female offspring (71%) of White ethnicity (97.3%), and away from those living in the most deprived areas (8.5%; Table S32 in S1 Text). Offspring were less religious than the parental generation, with lower rates of religious belief (15% vs 43% [mothers] and 30% [partners]), identity (30% vs 70% [mothers] and 58% [partners]) and regular religious service attendance (4% vs 14% [mothers] and 12% [partners]; Table 1; complete-case results in Table S6 in S1 Text).

Descriptive statistics for climate beliefs and behaviours among offspring were broadly similar compared to G0 mothers and partners (Table 2; with individual actions in Table S7, and complete-case results in Table S8, in S1 Text), although some differences were noted. For instance, offspring were marginally more likely to ‘definitely not’ or ‘probably not’ believe that the climate is changing (2.1% vs 1.0% [mothers] and 1.4% [partners]), less likely to respond ‘very concerned’ regarding climate change concern (40% vs 44% [mothers] and 44% [partners]), more likely to blame humans for all of climate change (32% vs 23% [mothers] and 28% [partners]), and less likely to endorse that personal actions can impact long-term climate change (52% vs 65% [mothers] and 58% [partners]). ALSPAC offspring also reported engaging in fewer pro-environmental actions for climate reasons than G0 mothers, but similar numbers to G0 partners (5.1 [out of 17] vs 5.7 [mothers] and 5.1 [partners]); a substantial minority of offspring (16%) also reported engaging in zero actions for climate reasons (Fig S157 in S1 Text).

Compared to the G0 mothers and partners, in the offspring generation there were fewer associations between religion and climate beliefs and behaviours. There was, at best, weak evidence that offspring with a Christian identity reported less belief in climate change and those who regularly attended religious services displaying greater belief (Table S33 & Figs S158-S160 in S1 Text). There was little association between the religious exposures and concern over climate change, although a Christian identity was associated with marginally lower levels of concern (Table S34 & Figs S161-S163 in S1 Text). Consistent with the G0 mothers and partners, religious offspring were less likely to attribute human activity as a cause of climate change (Table S35 & Figs S164-S166 in S1 Text) and more likely to believe that individual actions can make a difference to climate change (Table S36 & Figs S167-S169 in S1 Text).

Offspring with a Christian identity, compared to those without a religious identity, reported engaging in fewer pro-environmental behaviours for climate reasons, although no associations were found for the other religious exposures (Table S37 & Figs S170-S172 in S1 Text; results were comparable when using Poisson and zero-inflated Poisson models [Tables S38 & S39 and Figs S173-S178 in S1 Text] and when using a reduced number of proenvironmental actions [Tables S40-S42 & Figs S179-S187 in S1 Text]). Similar patterns were found when assessing each behaviour individually, albeit with some variation between the different actions (Table S43 & Figs S188-S235 in S1 Text). However, stronger results were found for reducing number of children and meat/dairy consumption, with participants who identified as Christian less likely to engage in these behaviours (similar associations between Christian identity and meat/dairy consumption were observed in G0 mothers and partners as well).

Across all outcomes, there was little difference between the adjusted models controlling vs not controlling for political ideology. When comparing between Christian denominations, participants with a Church of England affiliation displayed slightly less belief and concern over climate change, in addition to performing fewer pro-environmental actions, compared to Catholics, other Christians, and non-religious individuals, although responses between Christian denominations were overlapping. An overall summary of the key results across all generations can be found in Table 3.

## Discussion

Overall, we found a complex pattern of associations between the various facets of religiosity and the different climate beliefs and behaviours, which depended on the specific measure of RSBB, the climate outcome and the generation of the participants (Table 3). For instance, among the parental generation (G0 mothers and partners) a Christian identity was negatively associated with belief in climate change, while regularly attending a place of worship was positively associated with such climate beliefs; among the offspring generation, however, no strong associations were found with belief in climate change. As a further example, the total number of pro-environmental actions in the parental generation was negatively associated with Christian identity and positively associated with regular religious attendance, but in the offspring only a weak negative association with Christian identity was reported.

The religiosity latent classes may help explain these results, as either a ‘U’- or ‘J’-shaped pattern often emerged regarding belief, concern and taking action for climate reasons in the parental generation. That is, parents classified as “agnostic” or “moderately religious” had lower climate belief and concern, and performed fewer climate actions, compared to “atheists”, while participants categorised as “highly religious” were often as – and sometimes more – aware and active than “atheists”. It is possible that the greater engagement with Christianity among “highly religious” parents – e.g., attending services, being part of a local community, paying attention to the messages of faith leaders – means that these participants may be more likely to hear, and adhere to, pro-environmental messages espoused by the church, which may partially explain these patterns.

While this may explain why “highly religious” participants showed greater climate beliefs and behaviours, it does not explain why “agnostics” and “moderately religious” participants displayed fewer beliefs and behaviours compared to non-religious individuals. One tentative explanation is that non-religious participants may have greater trust in, and knowledge of, science [40], and hence be more aware and knowledgeable about climate change [65]. That is, there may be two routes to climate knowledge: i) an ‘evidence-based’ route via belief/knowledge of science (exemplified by non-religious participants); and ii) a ‘religious’ route via greater engagement with Christian practices and messages (although these need not be mutually-exclusive; religious engagement may also promote greater belief and knowledge of science, especially regarding the climate). This could plausibly explain the ‘U’/‘J’-shaped relationships reported in this study.

However, this explanation is very speculative, especially as the association between religion and trust in science is highly-variable cross-culturally [40] and we have no knowledge of the direction of association among ALSPAC study participants. An alternative explanation, which does not require religion to play a causal role in explaining these results, could be that people who are “agnostic” and less interested in religion may also be agnostic and less interested in science and climate change. While these results are interesting, at present the reason for these patterns is largely unknown; assuming these results replicate, understanding the mechanisms explaining them is likely complex and requires further research.

A further striking pattern in this study was the difference between the generations, with very few of the associations reported in the parental generation replicated in the offspring. One potential explanation is that the offspring generation have lower rates of religiosity, meaning less power to detect an effect, even if the effect sizes were of a similar magnitude. However, despite less power and wider confidence intervals in the offspring, overall the effect sizes/point estimates do seem smaller compared to the parental generation, meaning that a purely statistical explanation is unlikely to explain such differences. This suggests that the association between religion and climate beliefs and behaviours may be modified by age, with a stronger relationship among the older generation. A tentative explanation could be trust, which is known to be greater in older generations [66], with older religious individuals more likely to listen to religious leaders on climate issues than the younger generation. Alternatively, perhaps older religious individuals are higher in optimism, self-efficacy and/or internal locus of control compared to younger religious individuals, and hence perform more pro-environmental behaviours. Research has indicated that religion may be positively associated with these traits ([67,68]; although see [69]), while previous work using ALSPAC found that religious G0 parents had a greater internal locus of control with little association reported in the offspring generation [70]. Again, these patterns require replication and further investigation before stronger claims can be made.

Despite these differences, some associations were stable across the generations. For instance, there was a consistent negative association between religion and belief that humans are to blame for climate change in all cohorts. This may be due to lower levels of trust in science among religious individuals (although see caveats above regarding this explanation), or the attribution of climate change to God rather than human activities. A consistent positive association between religion and belief that personal actions can impact long-term climate change was also observed. As discussed above, this may reflect religious individuals having a stronger sense of control [67], meaning that these people are more likely to believe that their actions can affect change. However, this finding may depend on the specific religion measure used [69], as belief in a controlling God may reduce one’s sense of control and inhibit climate action [71]; additionally, as mentioned above, in the ALSPAC data there is little relationship between locus of control and religiosity in the offspring generation [70].

Overall, there was also a trend that participants with a Church of England identity displayed less climate beliefs/actions than other denominations (i.e., Catholic or other Christians), although results between the different denominations were overlapping to some extent due to the relatively small number of non-Church of England Christians. Assuming these differences are real, among Catholics this could be a direct effect of the Pope’s acceptance and promotion of anthropogenic climate change [27]. Alternatively, the pathway may be more indirect, such as Catholics and other Christians having higher rates of religious attendance and participation [72] which may shape climate beliefs and behaviours independent of liturgical content; this may be particularly pertinent in this population as ‘Church of England’ was often a default response to religious affiliation in England, even for individuals without religious beliefs or convictions [16].

As discussed in the introduction, previous empirical work has shown inconsistent associations between religion and climate beliefs/behaviours, with positive [17,42], negative [37], and null [43] associations reported in the literature. Direct comparisons are, however, complicated by differences in measurement of religion and climate beliefs/behaviours between studies. For instance, some studies focus on religious identity [37] while others on a range of religiosity measures [17]; similarly, other studies focus on single aspects of climate beliefs (e.g., environmental concern; [42]) while others explore a range of climate beliefs and behaviours [17,37]. This variation means that differences in results could be due to differences in methods, rather than differences between populations (although these explanations are not mutually-exclusive). However, even with these caveats some comparisons can be made. For instance, similar to a previous Australian study [37], in the parental ALSPAC generation a Christian identity had a negative association with belief in climate change and performing climate-friendly actions; while, conversely, regular religious service attendance had a positive association with these items, comparable to a previous cross-cultural study across 91 countries [17].

In addition to differences by country and religious faiths/traditions, our results also therefore suggest that study heterogeneity in the measurement of ‘religion’ and/or ‘climate beliefs/behaviours’ may be responsible for this variation between studies. Different religion and climate variables cannot therefore be used interchangeably, and the different mechanisms by which these religiosity factors may impact climate beliefs/behaviours needs to be considered. In this UK population at least, simply identifying as ‘Christian’ is unlikely to be sufficient in being receptive to pro-environmental messages from religious leaders; individuals may need to actively engage with religion, attend services and be part of religious communities to respond to these messages (assuming said associations are causal). This may plausibly vary by country though, as identifying as religious has been associated with greater environmental behaviour and care for nature in cross-cultural samples [17,42]. Understanding the factors which explain this variability, both between countries and between different aspects of religiosity, is a key area for future theoretical and empirical research.

Regardless of the specific mechanism(s), if these results are corroborated and generalisable, they may have practical implications; namely, as minimally-religious and agnostic older individuals are less likely to believe in climate change or engage in pro-environmental actions, this could be a relevant group to target when attempting to encourage people to mitigate the impacts of climate change (e.g., by disseminating tailored climate information or behaviour change efforts). A better understanding of *why* this group appears to have lower climate awareness and engagement could further aid these efforts by designing more effective communication campaigns (e.g., the type of communication needed, who the communication should come from, how the communication should occur, etc.). Nonetheless, any such practical real-world implications are currently very speculative and would require additional research to prove their efficacy before being implemented.

### Strengths and limitations

A key strength of this research is the use of data from a large-scale longitudinal population-based cohort study, with replication in three cohorts of participants over two generations. A further strength is the range of RSBB and climate belief/behaviour variables explored, providing a detailed and nuanced picture of how relationships vary by both the measurement of religion and different facets of climate change attitude and action. A final strength is that these results are from a UK population; much of the previous work in this area has been conducted in US samples.

Despite these strengths, there are a number of important limitations. First, while these findings are consistent with religious engagement causing climate beliefs/behaviours, there are many other confounders beyond the basic sociodemographic factors controlled for here which may confound this relationship. For instance, factors such as personality, cognitive ability, social support, optimism, self-efficacy and locus of control may shape both religious and climate attitudes and therefore act as confounders [67,68,70,73,74]. Additionally, although RSBB was measured prior to the climate questions, as these climate questions were only asked once it is impossible to rule out reverse causality – i.e., climate beliefs causing religious beliefs. Future work which includes a wider range of potential confounders, uses repeated climate data to rule out reverse causality [75], and employs alternative study designs to triangulate evidence [76], would provide stronger evidence for a causal interpretation.

Second, given the large amount of missing data in the complete-case samples there is the risk of selection biasing results [47]. However, we adjusted for a range of sociodemographic factors known to influence continued ALSPAC participation, such as age at birth, sex and socioeconomic position [49,77], somewhat reducing the risk of bias. Furthermore, although religious attendance is known to predict ALSPAC participation [55], simulations have suggested that any bias due to selection in these religion variables is likely to be minimal [78]. We therefore expect that missing data is unlikely to result in significant bias in our results but may contribute to inefficiency and greater uncertainty in parameter estimates (i.e., wider confidence intervals), but cannot rule out selection bias.

Third, measurement error may be a further source of bias [79]. This may lead to bias towards the null if such error is non-differential (i.e., ‘random’ measurement error), but could potentially bias in other directions if such bias is differential (e.g., if religious individuals report engaging in more climate behaviours than they do in reality). Without external information to validate these data, it is difficult to know the extent and direction of measurement error, if any; however, previous ALSPAC work with sensitive topics such as medical history and mental health has shown good correspondence with gold standard measures [80], providing some assurance against such measurement biases.

We also note generalisability as a limitation, as the extent to which these results can be generalised to other populations and belief systems – especially non-Christian faiths – is difficult to know without further study. As this study focused on parents and offspring from a relatively small area of southwest England, generalisability to the wider UK population (and beyond), in addition to older individuals who are not parents, cannot be assumed. The differences we observed between the generations also suggests that any relationship may be highly-variable depending on context, again cautioning against generalising to different populations. From a practical perspective, differences in climate beliefs and behaviours by religion may also be relatively minor; the largest differences for the climate belief variables were only a handful of percentage points, while the biggest difference in the total number of climate-friendly behaviours – between “agnostics” and “highly religious” – was approximately one action. These effect sizes need to be considered when thinking about the efficacy of potential targeted climate awareness campaigns.

Given the large number of analyses conducted, there is also a risk of some results being false positives. However, as the exposures and outcomes are correlated (e.g., belief in climate change predicts climate concern) it is not clear how to best correct for this – a simple Bonferroni correction would be too conservative, for instance – and advice regarding multiple testing corrections is often contradictory [81]. Nonetheless, as patterns of results were somewhat consistent across climate outcomes and between the mother and partner cohorts, this provides some assurance that they are not simply false positives. Finally, given the quantitative data available here, the mechanisms by which religion may shape climate beliefs/behaviours (if at all) are unclear; a qualitative approach would provide a useful in-depth comparison to further explore these results.

## Conclusion

In this paper we have explored in detail the associations between a range of religiosity exposures and climate belief/behaviour outcomes in two generations of a longitudinal UK birth cohort. We found a complex pattern of results, which differed by generation, the RSBB exposure and the climate outcome. In the parental generation, we observed a ‘U’-shaped or ‘J’-shaped association between religion and a range of climate beliefs and behaviours, with the most and least religious participants displaying the greatest belief in, and concern regarding, climate change, and engaging in the most pro-environmental behaviours for climate reasons. Additional research to assess whether these patterns are causal, the mechanisms by which religion may promote climate beliefs/attitudes, and whether these results replicate (especially in different countries and religious traditions) is needed. If these results are found to be causal and generalisable, climate communication efforts which focus on either agnostic or nominally/minimally religious individuals could perhaps increase climate change awareness and behaviours among the public.

## Supplementary Material

S1 Text. This supporting information file contains additional information regarding differences from the pre-registered analysis plan (Section S1), in addition to all supplementary tables (Tables S1-S43) and figures (Figs S1-S235). (PDF)

Supplementary Information

## Figures and Tables

**Table 1:** Descriptive statistics for the religious/spiritual beliefs and behaviours (RSBB) exposures in the G0 mother (*n* = 14,216), G0 partner (*n* = 10,916) and G1 offspring (*n* = 14,524) samples. Note that the percentages of missing data are calculated separately from the observed data. https://doi.org/10.1371/journal.pclm.0000469.t001

RSBB Variable	G0 mothers	G0 partners	G1 offspring
*Religious belief (belief in God or a divine power)*			
No	1,188 (27.3%)	900 (45.8%)	2,449 (59.0%)
Not sure	1,312 (30.1%)	484 (24.6%)	1,073 (25.9%)
Yes	1,857 (42.6%)	581 (29.6%)	627 (15.1%)
*Missing*	*9,859 (69.4%)*	*8,951 (82.0%)*	*10,375 (71.4%)*
*Religious identity (Christian denominations combined)*			
None	1,234 (28.6%)	815 (42.1%)	2,890 (70.3%)
Christian	3,074 (71.4%)	1,120 (57.9%)	1,220 (29.7%)
*Missing*	*9,908 (69.7%)*	*8,981 (82.3%)*	*10,414 (71.7%)*
*Religious identity (Christian denominations separated)*			
None	1,234 (28.6%)	815 (42.1%)	2,890 (70.3%)
Church of England	2,238 (52.0%)	827 (42.7%)	841 (20.5%)
Roman Catholic	350 (8.1%)	128 (6.6%)	161 (3.9%)
Other Christian	486 (11.3%)	165 (8.5%)	218 (3.9%)
*Missing*	*9,908 (69.7%)*	*8,981 (82.3%)*	*10,414 (71.7%)*
*Religious attendance*			
Occasional/None	3,736 (86.6%)	1,721 (88.3%)	3,928 (95.5%)
Regular	576 (13.5%)	229 (11.7%)	184 (4.5%)
*Missing*	*9,905 (69.7%)*	*8,966 (82.1%)*	*10,412 (71.7%)*
*Religious latent classes*			
Atheist	1,461 (33.4%)	1,013 (51.2%)	2,402 (63.1%)
Agnostic	1,216 (27.8%)	453 (22.9%)	785 (20.6%)
Moderately religious	1,171 (26.8%)	320 (16.2%)	361 (9.5%)
Highly religious	525 (12.0%)	194 (9.8%)	261 (6.8%)
*Missing*	*9,843 (69.2%)*	*8,935 (81.9%)*	*10,715 (73.8%)*

**Table 2:** Descriptive statistics for the climate beliefs and total number of pro-environmental actions performed in the G0 mother (*n* = 14,216), G0 partner (*n* = 10,916) and G1 offspring (*n* = 14,524) samples. Note that the percentages of missing data are calculated separately from the observed data. https://doi.org/10.1371/journal.pclm.0000469.t002

Climate Variable	G0 mothers – N (%) or mean (SD)	G0 partners – N (%) or mean (SD)	G1 offspring – N (%) or mean (SD)
*Believes that the climate is changing*			
Definitely not	13 (0.3%)	7 (0.4%)	44 (1.1%)
Probably not	30 (0.7%)	20 (1.0%)	41 (1.0%)
Yes, maybe	202 (4.5%)	99 (5.2%)	208 (5.2%)
Yes, probably	711 (15.8%)	293 (15.4%)	528 (13.2%)
Yes, definitely	3,544 (78.7%)	1,483 (78.0%)	*3,193 (79.6%)*
*Missing*	*9,715 (68.3%)*	*9,014 (82.6%)*	*10,510 (72.4%)*
*Concerned about the impact of climate change*			
Not at all concerned	49 (1.1%)	31 (1.6%)	92 (2.3%)
Not very concerned	276 (6.2%)	175 (9.3%)	342 (8.6%)
Somewhat concerned	2,206 (49.3%)	846 (44.7%)	1,933 (48.7%)
Very concerned	1,947 (43.5%)	840 (44.4%)	1,600 (40.3%)
*Missing*	*9,738 (68.5%)*	*9,024 (82.7%)*	*10,577 (72.7%)*
*Believes that humans are to blame for climate change*			
Not at all	52 (1.2%)	21 (1.1%)	37 (0.9%)
Yes, for some of it	1,253 (28.0%)	421 (22.2%)	714 (18.0%)
Yes, for most of it	2,144 (47.8%)	921 (48.6%)	1,947 (49.1%)
Yes, for all of it	1,032 (23.0%)	531 (28.0%)	1,267 (32.0%)
*Missing*	*9,735 (68.5%)*	*9,022 (82.6%)*	*10,559 (72.7%)*
*Thinks that personal actions will make* ^a^ *difference to long–term climate changes*			
No	377 (8.4%)	367 (19.4%)	831 (21.0%)
Not sure	1,180 (26.4%)	422 (22.3%)	1,064 (26.9%)
Yes	2,918 (65.2%)	1,104 (58.3%)	2,066 (52.2%)
*Missing*	*9,741 (68.5%)*	*9,023 (82.7%)*	*10,563 (72.7%)*
*Number of actions performed for* *climate reasons (all items)* ^a^	5.72 (3.86)	5.14 (4.17)	5.13 (3.65)
*Missing*	*10,373 (73.0%)*	*9,236 (84.6%)*	*10,964 (75.5%)*
*Number of actions performed for* *climate reasons (reduced items)* ^b^	4.99 (3.18)	4.36 (3.40)	4.74 (3.25)
*Missing*	*10,284 (72.3%)*	*9,203 (84.3%)*	*10,919 (75.1%)*

a Total of 16 behaviours for G0 mothers and partners, 17 for G1 offspring (see Table S2 in S1 Text).

b Total of 11 behaviours for G0 mothers and partners, 12 for G1 offspring (see Table S2 in S1 Text).

**Table 3:** Summary of key results. A dash (“-”) indicates little-to-no association between the religious exposure and climate outcome. Neg = Negative association (e.g., among G0 mothers a Christian identity was negatively associated with belief that the climate is changing); Pos = Positive association (e.g., among G0 partners regular religious attendance was positively associated with concern regarding climate change); U-shaped = ‘U’-shaped association (e.g., among G0 mothers the association between the religiosity latent classes and concern over climate change was U-shaped, with “agnostic” participants less concerned than “atheist” participants, but no difference between “highly religious” and “atheist”); J-shaped = ‘J’-shaped association (e.g., among G0 mothers the association between the religiosity latent classes and number of pro-environmental behaviours was J-shaped, with “agnostic” and “moderately religious” participants engaging in fewer actions than “atheists”, but with “highly religious” engaging in more actions than “atheists”). For all religious exposures the baseline/reference categories reflect lower/no religiosity, so a positive relationship (for instance) between a religious exposure and climate outcome indicates that higher levels of religiosity are associated with greater climate awareness, concern or behaviours. https://doi.org/10.1371/journal.pclm.0000469.t003

Climate outcome	G0 Mothers				G0 Partners				G1 Offspring			
	*Religious belief*	*Religious identity*	*Religious attendance*	*Latent* *classes*	*Religious belief*	*Religious identity*	*Religious attendance*	*Latent* *classes*	*Religious belief*	*Religious identity*	*Religious* *attendance*	*Latent* *classes*
*Believes that the climate is changing*	–	Neg	Pos	–	–	Neg	Pos	J-shaped	–	–	–	–
*Concerned about impact of climate change*	Neg	Neg	–	U-shaped	Neg	Neg	Pos	J-shaped	–	–	–	–
*Believes humans are to blame for climate change*	Neg	Neg	–	Neg	Neg	Neg	–	U-shaped	Neg	Neg	–	–
*Thinks personal actions will impact long-term climate change*	Pos	Pos	Pos	Pos	Pos	–	Pos	Pos	Pos	Pos	–	Pos
*Number of actions performed for climate reasons*	–	Neg	Pos	J-shaped	–	Neg	Pos	J-shaped	–	Neg	–	–

## Data Availability

ALSPAC data access is through a system of managed open access. Information about access to ALSPAC data is given on the ALSPAC website (http://www.bristol.ac.uk/alspac/researchers/access/) and in the ALSPAC data management plan (http://www.bristol.ac.uk/alspac/researchers/data-access/documents/alspac-data-management-plan.pdf). Data used for this submission will be made available on request to the Executive (alspac-exec@bristol.ac.uk). The datasets presented in this article are linked to ALSPAC project number B4123, please quote this project number during your application. Analysis code and synthetic ALSPAC datasets (created using the ‘synthpop’ R package) are openly-available on DM-S’s GitHub page: https://github.com/djsmith-90/ReligionAndClimate_B4123. As raw ALSPAC data cannot be released, these synthesised datasets are modelled on the original data, thus maintaining variable distributions and relations among variables (albeit not perfectly), while at the same time preserving participant anonymity and confidentiality, thus allowing this research to be ‘quasi-reproducible’. Please note that while these synthetic datasets can be used to follow the analysis scripts, as data are simulated they should not be used for research purposes; only the actual, observed, ALSPAC data should be used for formal research and analyses reported in published work.
