# Supplementary material for: Associations between religiosity and climate change beliefs and behaviours in the Avon Longitudinal Study of Parents and Children (ALSPAC)
Source: PLOS Clim. Author manuscript; Available in PMC 2025 Nov 18. (PMC7618382; doi:10.1371/journal.pclm.0000469)

Supporting Information for 'Associations between religiosity and climate change beliefs and behaviours in the Avon Longitudinal Study of Parents and Children (ALSPAC)'

Section S1: Differences from Pre-Registered Analysis Plan (https://osf.io/p5vjz/)

The pre-registered analysis plan was followed as specified, with no substantial changes to the research questions or analysis methods. Some minor changes were made, though, as detailed below:

- Minor updates to the introduction to include additional references and literature published since the analysis plan was pre-registered (e.g., on COP28).
- In the pre-registered analysis plan, regarding the sensitivity analysis including political ideology as an additional confounder in the G1 offspring analyses, we stated that "All principal components which explain a large proportion of the variance (e.g., 40% or more, as a rough heuristic) in these variables will be used as additional confounders to try and remove this potential source of bias." However, as described in the 'confounders' section of the Methods, the principal components were found to explain less of this variation than 40% (22% for component 1 and 16% for component 2). As these components together explain nearly 40% of the variance, and both have a sensible and logical interpretation, we decided to retain both components for analyses.
- In the pre-registered analysis plan we said that count data (i.e., 'total number of actions taken for climate reasons') would be analysed using Poisson regression models. While these Poisson models were performed, we additionally conducted linear regression models on this outcome (as the coefficients are more straightforward to interpret than Poisson models), as well as using zero-inflated Poisson models (due to an excess of zeros in these data, which we did not anticipate prior to exploring the data). As each of these models gave similar results, this suggests that the associations are robust, even to potential model mis-specification.
- Regarding interpretation of analyses, in the pre-registered plan we also said that "Within each cohort, Bonferroni adjustment for the false discovery rate will be applied." However, due to the large number of comparisons and lack of independence between outcomes (e.g., belief in climate change is likely to be correlated with concern regarding climate change and climate change actions), we decided not to apply this Bonferroni false discovery rate; instead, *p*-values are presented 'as is', without taking into consideration the number of tests conducted. As this may nonetheless inflate the false discovery rate, we have noted this as a limitation in our discussion.
- We adjusted for confounders as described, although for partners we did not include 'ethnicity' as a confounder due to the lower number of partners with an 'other than white' ethnicity which resulted in some models being unable to converge and produce interpretable results.

Table S1: The ALSPAC religiosity variables used as exposures in the present study (the latent class variables have not included here, as they are a composite variable).

**Question**	**Variable coding**	**Variable name** (mothers; *partners*; **offspring**)
Do you believe in God or in some divine power?	Unordered category (Yes vs Not sure vs No)	Y3000; *FC3000*; **YPG3000**
What sort of faith/belief would you say you have? (Christian denominations combined)	Binary (None vs Christian)	Y3040; *FC3040*; **YPG3040**
What sort of faith/belief would you say you have? (Christian denominations separate)	Unordered category (None vs Church of England/Protestant vs Roman Catholic vs Other Christian)	Y3040; *FC3040*; **YPG3040**
How often do you attend church/temple/mosque or other religious meetings?	Binary (Regular attendance vs Occasional/non-attendance)	Y3080; *FC3080*; **YPG3080**
Religiosity latent classes	Ordered category ("Highly religious" [characterised by belief in God, regular religious attendance and receiving help/support from co-religionists] vs "Moderately religious" [characterised by belief in God, but less likely to attend religious services or obtain help/support from co-religionists] vs "Agnostic" [characterised by uncertainty regarding belief in God and not attending religious services] vs "atheist" [characterised by lack of belief in God and not attending religious services])	NA; *NA*; **NA**[a]

[a] These latent class variables are composed of multiple religion variables.

Table S2: The ALSPAC climate beliefs and behaviours questions used as outcomes in the present study.

Question	Variable coding	**Variable name** (mothers; *partners*; **offspring**)
1) Do you believe that the climate is changing?	Ordered category (Yes definitely vs Yes probably vs Yes maybe vs Probably not vs Definitely not)	Z3000; *FD3000*; **YPJ3000**
2a) How concerned are you about the impact of climate change? [a]	Ordered category (Not at all concerned vs Not very concerned vs Somewhat concerned vs Very concerned)	Z3001; *FD3001*; **YPJ3001**
2b) Do you believe that humans are to blame for climate change? [a]	Ordered category (Yes, for all of it vs Yes, for most of it vs Yes, for some of it vs Not at all)	Z3002; *FD3002*; **YPJ3002**
2c) Do you think that what you do, however small, will make a difference to the long-term effects of changes to our climate? [a]	Unordered category (Yes vs Not sure vs No)	Z3003; *FD3003*; **YPJ3003**
4) Have you taken any of the following actions whether or not because of concerns about climate change?<br>a) Changed the way I travel locally<br>b) Reduced my household waste<br>c) Reduced energy use at home<br>d) Changed what I buy<br>e) Reduced air travel<br>f) Bought or hired an electric or hybrid vehicle<br>g) Bought foods produced locally<br>h) Recycled/Upcycled more<br>i) Reduced the amount of plastic I used<br>j) Chosen sustainably sourced items<br>k) Improved insulation in the home<br>l) Installed solar panels<br>m) Started growing vegetables<br>n) Planted tree(s)<br>o) Avoided organisations that support fossil fuels<br>p) Not had children, or reduced the number that I had planned<br>q) Other<br>r) Taken action to eat less or no meat and/or dairy products	Unordered category (Action taken due to climate change vs Action taken for other reasons vs Action taken due to climate change and for other reasons vs Have not done this) [b]<br><br>We will also create a 'total climate behaviours score' based on the total number of actions taken for climate change reasons [c]. Given that many of these items are likely to be socially- and economically-patterned (e.g., many people may wish to own an electric vehicle or install solar panels but cannot afford to), we will also create a 'reduced total climate behaviours score' removing items e, f, k, l and m.	Z3020-Z3075; *FD3020-FD3075*; **YPJ3020-YPJ3075**

[a] Individuals who answered 'definitely not' to the question "Do you believe that the climate is changing?" did not answer this question.
[b] Note that question r ("taken action to eat less or no meat and/or dairy products") contained the additional responses "I have always been vegan" and "I have always been vegetarian". Individuals who answered "I have always been vegan" were excluded from analyses for this question.

[c] Note that for G0 mothers and partners item p (‘not had children, or reduced the number of children planned’) was not included in these total scores as so few participants answered in the affirmative to this question (likely because most G0 participants were of post-reproductive age). For all cohorts (G0 mothers, G0 partners and G1 offspring) item q (‘other action’) was also excluded from these total scores as so few participants answered positively to this question and many participants did not answer it.

Table S3: The ALSPAC variables used as confounders in the present study. 'NA' indicates that said variable is not applicable to that cohort (e.g., 'sex' for G0 mothers and partners).

**Variable**	**ALSPAC cohort (variable name)**	**Variable coding**	**When measured**
Age	G0 mothers (*Z6500*); G0 partners (*FD6500*); G1 (*YPJ7500*)	Continuous (years for G0; months for G1)	When study child approx. age 30 years
Sex	G0 mothers and partners (*NA*); G1 (*kz021*)	Binary (Female vs Male)	At birth
Ethnicity	G0 mothers (*c800*); G0 partners (*c801*); G1 (*C804*)	Binary (White vs other than White)	In pregnancy
Marital status	G0 mothers (*a525*); G0 partners (*pa065*); G1 (*NA*)	Unordered category (never married vs currently married vs widowed/divorced/ separated)	In pregnancy
Relationship status (living with a partner)	G0 mothers and partners (*NA*); G1 (*YPG1052*)	Binary (No vs Yes)	Approx. age 28 years
Urban/Rural location	G0 mothers and partners (*jan2014ur01ind_M*); G1 (*jan2021ur01ind_YP*)	Binary (town/village/hamlet vs urban)	January 2014 for G0; January 2021 for G1
Highest educational qualification (G0)	G0 mother (*c645a*); G0 partner (*c666a*); G1 (*NA*)	Ordered category (CSE/none vs vocational vs O-level vs A-level vs degree) [a]	In pregnancy
Highest educational qualification (G1)	G0 mothers and partners (*NA*); G1 (*YPF7970*)	Ordered category (GCSE/none vs A-level [or equivalent] vs degree) [a]	Approx. age 27 years
Occupational social class (G0)	G0 mother (*c755*); G0 partner (*c765*); G1 (*NA*)	Binary (low [III manual/IV/V] vs high [I/II/III non-manual]) [b]	In pregnancy
Occupational social class (G1)	G0 mothers and partners (*NA*); G1 (*YPC2492*)	Ordered category (Managerial, administrative and professional vs Intermediate vs Small employers vs Lower supervisory and technical vs [Semi-]routine) [c]	Approx. age 23 years
Weekly household income after tax (G0)	G0 mothers and partners (*h470*); G1 (*NA*)	Ordered category (£0-£100 vs £100-£199 vs £200-£299 vs £300-£399 vs £400 and above)	When study child approx. age 3 years
Monthly income after tax (G1)	G0 mothers and partners (*NA*); G1 (*YPE6020*)	Ordered category (£0-£499 vs £500-£999 vs £1000-£1499 vs £1500-£1999 vs £2000 and above)	Approx. age 26 years
Index of multiple deprivation	G0 mothers and partners (*jan2014imd2010q5_M*); G1 (*jan2021imd2015q5_YP*)	Ordered category (1st quintile [least deprived] vs 2nd quintile vs 3rd quintile vs 4th quintile vs 5th quintile [most deprived])	January 2014 for G0; January 2021 for G1
Housing status	G0 mothers and partners (*a006*); G1 (*YPG1060*)	Unordered category (owned/mortgaged vs renting vs council/housing association vs other)	In pregnancy for G0; Approx. age 28 for G1

[a] GCSE = General Certificate of Secondary Education qualification (compulsory examinations sat at the end of secondary school at approx. age 16; introduced in 1986 to replace CSE and O-levels); CSE = Certificate of Secondary Education qualification (examinations sat at the end of secondary school at approx. age 16;

compulsory from the early 1970s, unless completing O-level qualifications instead; replaced in 1986 by GCSEs); O-level = Ordinary level qualifications (examinations sat at the end of secondary school, often for more academically-able pupils at approx. age 16; replaced in 1986 by GCSEs); A-level = Advanced level qualification (non-compulsory examinations sat at the end of college or sixth form at approx. age 18).
[b] For more information on these occupational social classes, see: https://sru.soc.surrey.ac.uk/SRU9.html.
[c] For more information on these National Statistics Socio-Economic Classification categories, see: https://www.ons.gov.uk/methodology/classificationsandstandards/otherclassifications/thenationalstatisticssocioeconomicclassificationnssecrebasedonsoc2010.

Table S4: Results of the political ideology principal components analysis on G1 offspring (*n* = 3,951). The analysis was performed using promax rotation to extract two principal components, using a tetrachoric correlation matrix (as the constituent variables were all binary).

**Item**	**Component 1 loadings**	**Component 2 loadings**	**h^2 (communality)**	**u^2 (uniqueness)**	**Item complexity**
Crime	0.39	0.36	0.27	0.73	2.0
Economy	0.60	0.04	0.36	0.64	1.0
Education	-0.20	0.73	0.57	0.43	1.1
Europe	0.05	-0.28	0.08	0.92	1.1
Immigration	0.54	-0.18	0.32	0.68	1.2
LQBTQ rights	-0.76	-0.01	0.58	0.42	1.0
NHS	-0.02	0.73	0.53	0.47	1.0
Unemployment	0.17	0.28	0.11	0.89	1.7
Women's rights	-0.74	0.16	0.59	0.41	1.1

Table S5: Descriptive statistics of sociodemographic characteristics and other confounders for G0 mothers. Columns display descriptive statistics for both the full sample (n = 14,216) and the complete-case sample with fully-observed data on all confounders, any RSBB exposure data and any climate outcome data (*n* = 2,578). Note that the percentages of missing data are calculated separately from the observed data.

**Variable**	**Full sample – N (%) or mean (SD)**	**Complete-case sample – N (%) or mean (SD)**
Age at birth of study child (years)	28.0 (4.96)	29.9 (4.23)
Missing	*850 (6.0%)*	*NA*
Age at completion of climate questions (years)	59.6 (4.44)	59.8 (4.26)
Missing	*9.654 (67.9%)*	*NA*
Ethnicity		
White	11,472 (97.5%)	2,546 (98.8%)
Other than White	297 (2.5%)	32 (1.24%)
Missing	*2,447 (17.2%)*	*NA*
Marital status		
Married	9,390 (75.1%)	2,228 (86.4%)
Never married	2,369 (18.9%)	249 (9.7%)
Separated/Divorced/Widowed	750 (6.0%)	101 (3.9%)
Missing	*1,707 (12.0%)*	*NA*
Urban vs rural location		
Urban	10,489 (83.2%)	2,034 (78.9%)
Rural	2,123 (16.8%)	544 (21.1%)
Missing	*1,604 (11.3%)*	*NA*
Highest educational qualification		
CSE/None	2,411 (6.3%)	163 (6.3%)
Vocational	1,184 (10.0%)	148 (5.7%)
O-level	4,109 (34.6%)	875 (33.9%)
A-level	2,647 (22.3%)	794 (30.8%)
Degree	1,506 (12.7%)	598 (23.2%)
Missing	*2,359 (16.6%)*	*NA*
Occupational social class		
I	557 (5.8%)	243 (9.4%)
II	3,016 (31.4%)	995 (38.6%)
III (non-manual)	4,118 (42.9%)	1,050 (40.7%)
III (manual)	753 (7.9%)	108 (4.2%)
IV/V	1,151 (12.0%)	182 (7.1%)
Missing	*4,621 (32.5%)*	*NA*
Household income (per week)		
< £100	738 (8.8%)	84 (3.3%)
£100 - £199	1,486 (17.6%)	297 (11.5%)
£200 - £299	2,395 (28.4%)	702 (27.2%)

£300 - £399	1,789 (21.2%)	668 (25.9%)
≥ £400	2,014 (23.9%)	827 (32.1%)
Missing	*5,794 (40.8%)*	*NA*
Area-level index of multiple deprivation during pregnancy (quintiles)		
1 (Least deprived)	3,785 (30.5%)	1,074 (41.7%)
2	2,827 (22.8%)	701 (27.2%)
3	2,199 (17.7%)	421 (16.3%)
4	2,033 (16.4%)	272 (10.6%)
5 (Most deprived)	1,576 (12.7%)	110 (4.3%)
Missing	*1,796 (12.6%)*	*NA*
Home ownership status		
Owned/Mortgaged	9,145 (73.3%)	2,299 (89.2%)
Private rented	887 (7.1%)	104 (4.0%)
Council/Housing association	2,002 (16.0%)	107 (4.2%)
Other	436 (3.5%)	68 (2.6%)
Missing	*1,746 (12.3%)*	*NA*

Table S6: Descriptive statistics for the religious/spiritual beliefs and behaviours (RSBB) exposures in the G0 mother (*n* = 2,578), G0 partner (*n* = 1,126) and G1 offspring (*n* = 1,100) complete-case samples (i.e., with fully-observed data on all confounders, any RSBB exposure data and any climate outcome data). Note that the percentages of missing data are calculated separately from the observed data.

**RSBB Variable**	**G0 mothers**	**G0 partners**	**G1 offspring**
Religious belief (belief in God or a divine power)			
No	704 (27.4%)	521 (46.7%)	635 (57.7%)
Not sure	789 (30.7%)	279 (25.0%)	295 (26.8%)
Yes	1,079 (42.0%)	316 (28.3%)	170 (15.4%)
Missing	*6 (0.2%)*	*10 (0.9%)*	*0 (0.0%)*
Religious identity (Christian denominations combined)			
None	732 (28.8%)	480 (43.4%)	753 (68.5%)
Christian	1,813 (71.2%)	625 (56.6%)	346 (31.5%)
Missing	*33 (1.3%)*	*21 (1.9%)*	*1 (0.1%)*
Religious identity (Christian denominations separated)			
None	732 (28.8%)	480 (43.4%)	753 (68.5%)
Church of England	1,319 (51.8%)	463 (41.9%)	236 (21.5%)
Roman Catholic	191 (7.5%)	62 (5.6%)	40 (3.6%)
Other Christian	303 (11.9%)	100 (9.1%)	70 (6.4%)
Missing	*33 (1.3%)*	*21 (1.9%)*	*1 (0.1%)*
Religious attendance			
Occasional/None	2,180 (85.7%)	979 (87.6%)	1,032 (94.6%)
Regular	365 (14.3%)	139 (12.4%)	59 (5.4%)
Missing	*33 (1.3%)*	*8 (0.7%)*	*9 (0.8%)*
Religious latent classes			
Atheist	872 (33.8%)	594 (52.8%)	653 (62.3%)
Agnostic	719 (27.9%)	258 (22.9%)	222 (21.2%)
Moderately religious	663 (25.7%)	155 (13.8%)	88 (8.4%)
Highly religious	324 (12.6%)	119 (10.6%)	85 (8.1%)
Missing	*0 (0.0%)*	*0 (0.0%)*	*52 (4.7%)*

Table S7: Descriptive statistics for the individual pro-environmental action outcomes in the G0 mother (*n* = 14,216), G0 partner (*n* = 10,916) and G1 offspring (*n* = 14,524) samples. Note that the percentages of missing data are calculated separately from the observed data.

**Climate Variable**	**G0 mothers**	**G0 partners**	**G1 offspring**
Changed the way travelled locally			
Not done this	2,752 (61.6%)	1,041 (55.1%)	2,566 (64.2%)
Yes, for climate change	819 (18.4%)	410 (21.7%)	591 (14.8%)
Yes, for other reasons	768 (17.2%)	373 (19.7%)	676 (16.9%)
Yes, for climate and other reasons	125 (2.8%)	66 (3.5%)	165 (4.1%)
Missing	*9,752 (68.6%)*	*9,026 (82.7%)*	*10,526 (72.5%)*
Reduced household waste			
Not done this	360 (8.1%)	262 (13.9%)	926 (23.2%)
Yes, for climate change	3,044 (68.0%)	1,011 (53.5%)	2,361 (59.1%)
Yes, for other reasons	909 (20.3%)	533 (28.2%)	479 (12.0%)
Yes, for climate and other reasons	161 (3.6%)	83 (4.4%)	228 (5.7%)
Missing	*9, 742 (68.5%)*	*9,027 (82.7%)*	*10,530 (72.5%)*
Reduced energy use at home			
Not done this	920 (20.5%)	399 (21.1%)	1,294 (32.4%)
Yes, for climate change	2,155 (48.0%)	795 (42.1%)	1,523 (38.1%)
Yes, for other reasons	1,224 (27.3%)	600 (31.8%)	864 (21.6%)
Yes, for climate and other reasons	188 (4.2%)	93 (4.9%)	317 (7.9%)
Missing	*9,729 (68.4%)*	*9,029 (82.7%)*	*10,526 (72.5%)*
Changed what buy			
Not done this	1,241 (27.8%)	690 (36.6%)	1,463 (36.7%)
Yes, for climate change	2,127 (47.7%)	716 (38.0%)	1,779 (44.6%)
Yes, for other reasons	927 (20.8%)	419 (22.2%)	482 (12.1%)
Yes, for climate and other reasons	166 (3.7%)	60 (3.2%)	264 (6.6%)
Missing	*9,755 (68.6%)*	*9,031 (82.7%)*	*10,536 (72.5%)*
Reduced air travel			
Not done this	2,235 (50.4%)	923 (48.9%)	2,461 (61.9%)
Yes, for climate change	836 (18.8%)	403 (21.4%)	519 (13.0%)
Yes, for other reasons	1,290 (29.1%)	530 (28.1%)	882 (22.2%)
Yes, for climate and other reasons	76 (1.7%)	30 (1.6%)	115 (2.9%)
Missing	*9,779 (68.8%)*	*9,030 (82.7%)*	*10,547 (2.9%)*
Bought or hired electric or hybrid vehicle			
Not done this	3,935 (88.0%)	1,574 (83.3%)	3,690 (92.1%)
Yes, for climate change	370 (8.3%)	207 (11.0%)	162 (4.0%)
Yes, for other reasons	135 (3.0%)	90 (4.8%)	124 (3.1%)
Yes, for climate and other reasons	33 (0.7%)	19 (1.0%)	30 (0.8%)
Missing	*9,743 (68.5%)*	*9,026 (82.7%)*	*10,518 (72.4%)*
Bought foods produced locally			
Not done this	1,192 (26.7%)	559 (29.6%)	1,788 (44.7%)
Yes, for climate change	1,817 (40.7%)	645 (34.1%)	1,164 (29.1%)
Yes, for other reasons	1,265 (28.4%)	616 (32.6%)	767 (19.2%)

Yes, for climate and other reasons	186 (4.2%)	72 (3.8%)	277 (6.9%)
Missing	*9,756 (68.6%)*	*9,024 (82.7%)*	*10,528 (72.5%)*
Recycled or upcycled more			
Not done this	272 (6.1%)	176 (9.3%)	614 (15.3%)
Yes, for climate change	3,047 (68.2%)	1,098 (58.1%)	2,408 (60.1%)
Yes, for other reasons	970 (21.7%)	518 (27.4%)	637 (15.9%)
Yes, for climate and other reasons	181 (4.0%)	99 (5.2%)	345 (8.6%)
Missing	*9,746 (68.6%)*	*9,025 (82.7%)*	*10,520 (72.4%)*
Reduced amount of plastic used			
Not done this	470 (10.5%)	351 (18.5%)	887 (22.2%)
Yes, for climate change	3,197 (71.3%)	1,075 (56.8%)	2,517 (62.9%)
Yes, for other reasons	662 (14.8%)	386 (20.4%)	373 (9.3%)
Yes, for climate and other reasons	156 (3.5%)	81 (4.3%)	225 (5.6%)
Missing	*9,731 (68.5%)*	*9,023 (82.7%)*	*10,522 (72.4%)*
Chosen sustainably sourced items			
Not done this	1,342 (30.2%)	589 (31.4%)	1,602 (40.2%)
Yes, for climate change	2,310 (51.9%)	860 (45.8%)	1,887 (47.3%)
Yes, for other reasons	679 (15.2%)	363 (19.3%)	277 (6.9%)
Yes, for climate and other reasons	120 (2.7%)	67 (3.6%)	224 (5.6%)
Missing	*9,765 (68.7%)*	*9,037 (82.8%)*	*10,534 (72.5%)*
Improved insulation in home			
Not done this	2,594 (58.0%)	978 (51.7%)	3,358 (84.0%)
Yes, for climate change	898 (20.1%)	408 (21.6%)	245 (6.1%)
Yes, for other reasons	886 (19.8%)	447 (23.6%)	318 (7.9%)
Yes, for climate and other reasons	95 (2.1%)	59 (3.1%)	79 (2.0%)
Missing	*9,743 (68.5%)*	*9,024 (82.7%)*	*10,524 (72.5%)*
Installed solar panels			
Not done this	4,022 (90.2%)	1,633 (86.5%)	3,863 (96.5%)
Yes, for climate change	271 (6.1%)	155 (8.2%)	63 (1.6%)
Yes, for other reasons	139 (3.2%)	83 (4.4%)	68 (1.7%)
Yes, for climate and other reasons	29 (0.6%)	16 (0.9%)	10 (0.2%)
Missing	*9,755 (68.6%)*	*9,029 (82.7%)*	*10,520 (72.4%)*
Started growing vegetables			
Not done this	4,022 (59.5%)	1,167 (61.8%)	3,029 (75.8%)
Yes, for climate change	616 (13.8%)	192 (10.2%)	279 (7.0%)
Yes, for other reasons	1,129 (25.2%)	509 (26.9%)	577 (14.4%)
Yes, for climate and other reasons	71 (1.6%)	22 (1.2%)	112 (2.8%)
Missing	*9,737 (68.5%)*	*9,026 (82.7%)*	*10,527 (72.5%)*
Planted tree(s)			
Not done this	2,954 (66.1%)	1,235 (65.8%)	3,333 (83.4%)
Yes, for climate change	734 (16.4%)	241 (12.8%)	290 (7.2%)
Yes, for other reasons	709 (15.9%)	365 (19.5%)	293 (7.3%)
Yes, for climate and other reasons	69 (1.6%)	35 (1.9%)	83 (2.1%)

Missing	*9,750 (68.6%)*	*9,040 (82.8%)*	*10,525 (72.5%)*
Avoided organisations that support fossil fuels			
Not done this	3,670 (82.8%)	1,497 (79.4%)	3,292 (82.6%)
Yes, for climate change	637 (14.4%)	323 (17.1%)	589 (14.8%)
Yes, for other reasons	101 (2.3%)	56 (3.0%)	54 (1.3%)
Yes, for climate and other reasons	24 (0.5%)	9 (0.5%)	52 (1.3%)
Missing	*9,784 (68.8%)*	*9,031 (82.7%)*	*10,537 (72.5%)*
Not had children, or reduced number of children planned [a]			
Not done this	>4,325 (>97.5%)	<1,850 (>98.0%)	3,361 (84.3%)
Yes, for climate change	16 (0.4%)	5 (0.3%)	206 (5.2%)
Yes, for other reasons	70 (1.6%)	28 (1.5%)	336 (8.4%)
Yes, for climate and other reasons	<5 (<0.1%)	<5 (<0.3%)	86 (2.2%)
Missing	*9,797 (68.9%)*	*9,027 (82.7%)*	*10,535 (72.5%)*
Other action [a]			
Not done this	>1,810 (>95.5%)	>1,020 (>94.5%)	2,483 (95.4%)
Yes, for climate change	48 (2.5%)	38 (3.5%)	87 (3.3%)
Yes, for other reasons	24 (1.3%)	14 (1.3%)	21 (0.8%)
Yes, for climate and other reasons	<5 (<0.3%)	<5 (<0.5%)	13 (0.5%)
Missing	*12,328 (86.7%)*	*9,838 (90.1%)*	*11,920 (81.2%)*
Eaten less or no meat and/or dairy			
Not done this	1,838 (43.8%)	864 (47.2%)	1,793 (47.4%)
Yes, for climate change	715 (17.0%)	248 (13.6%)	782 (20.7%)
Yes, for other reasons	1,223 (29.2%)	534 (29.2%)	570 (15.1%)
Yes, for climate and other reasons	419 (10.0%)	184 (10.0%)	638 (16.9%)
Missing	*10,021 (70.5%)*	*9,086 (83.2%)*	*10,741 (74.0%)*

[a] Note that some cells with counts < 5 have been altered to preserve participant anonymity.

Table S8: Descriptive statistics for the climate beliefs and behaviour outcomes in the G0 mother (*n* = *n* = 2,578), G0 partner (*n* = 1,126) and G1 offspring (*n* = 1,100) complete-case samples (i.e., with fully-observed data on all confounders, any RSBB exposure data and any climate outcome data). Note that the percentages of missing data are calculated separately from the observed data.

**Climate Variable**	**G0 mothers – N (%) or mean (SD)**	**G0 partners – N (%) or mean (SD)**	**G1 offspring – N (%) or mean (SD)**
Believes that the climate is changing			
Definitely not	5 (0.2%)	<5 (<0.5%)	<5 (<0.5%)
Probably not	11 (0.4%)	12 (1.1%)	5 (0.5%)
Yes, maybe	98 (3.8%)	51 (4.5%)	42 (3.8%)
Yes, probably	393 (15.3%)	182 (16.2%)	127 (11.6%)
Yes, definitely	2,068 (80.3%)	>875 (>77.5%)	>920 (>83.5%)
Missing	*3 (0.1%)*	*1 (0.1%)*	*0 (0.0%)*
Concerned about the impact of climate change			
Not at all concerned	19 (0.7%)	12 (1.1%)	20 (1.8%)
Not very concerned	147 (5.7%)	107 (9.5%)	79 (7.2%)
Somewhat concerned	1,244 (48.5%)	497 (44.4%)	548 (50.0%)
Very concerned	1,156 (45.0%)	504 (45.0%)	449 (41.0%)
Missing	*12 (0.5%)*	*6 (0.5%)*	*4 (0.4%)*
Believes that humans are to blame for climate change			
Not at all	23 (0.9%)	7 (0.6%)	<5 (<0.5%)
Yes, for some of it	666 (25.9%)	251 (22.4%)	179 (16.4%)
Yes, for most of it	1,237 (48.2%)	537 (47.9%)	559 (51.0%)
Yes, for all of it	641 (25.0%)	326 (29.1%)	>350 (>32.0%)
Missing	*11 (0.4%)*	*5 (0.4%)*	*5 (0.5%)*
Thinks that personal actions will make a difference to long-term climate changes			
No	228 (8.9%)	221 (19.7%)	202 (18.4%)
Not sure	656 (25.6%)	239 (21.3%)	296 (27.0%)
Yes	1,682 (65.6%)	660 (58.9%)	597(54.5%)
Missing	*12 (0.5%)*	*6 (0.5%)*	*5 (0.5%)*
Number of actions performed for climate reasons (all items) [a]	5.86 (3.82)	5.11 (4.17	5.19 (3.51)
Missing	*354 (13.7%)*	*133 (11.8%)*	*117 (10.6%)*
Number of actions performed for climate reasons (reduced items) [b]	5.13 (3.15)	4.35 (3.43)	4.84 (3.17)
Missing	*304 (11.8%)*	*115 (10.2%)*	*105 (9.5%)*
Changed the way travelled locally			
Not done this	1,528 (59.7%)	602 (53.9%)	697 (63.7%)
Yes, for climate change	518 (20.2%)	251 (22.5%)	156 (14.3%)
Yes, for other reasons	432 (16.9%)	225 (20.1%)	189 (17.3%)
Yes, for climate and other reasons	80 (3.1%)	39 (3.5%)	52 (4.7%)
Missing	*20 (0.8%)*	*9 (0.8%)*	*6 (0.5%)*
Reduced household waste			

Not done this	215 (8.4%)	145 (13.0%)	261 (23.8%)
Yes, for climate change	1,756 (68.4%)	612 (54.7%)	665 (60.7%)
Yes, for other reasons	491 (19.1%)	320 (28.6%)	102 (9.3%)
Yes, for climate and other reasons	104 (4.1%)	42 (3.7%)	68 (6.2%)
Missing	*12 (0.5%)*	*7 (0.6%)*	*4 (0.4%)*
Reduced energy use at home			
Not done this	545 (21.2%)	244 (21.9%)	353 (32.2%)
Yes, for climate change	1,248 (48.6%)	472 (42.2%)	411 (37.5%)
Yes, for other reasons	666 (25.9%)	346 (31.0%)	230 (21.0%)
Yes, for climate and other reasons	109 (4.2%)	54 (4.8%)	101 (9.2%)
Missing	*10 (0.4%)*	*11 (1.0%)*	*5 (0.5%)*
Changed what buy			
Not done this	680 (26.7%)	403 (36.3%)	392 (35.9%)
Yes, for climate change	1,259 (49.4%)	437 (39.3%)	489 (44.8%)
Yes, for other reasons	508 (19.9%)	233 (21.0%)	133 (12.2%)
Yes, for climate and other reasons	104 (4.1%)	38 (3.4%)	78 (7.1%)
Missing	*27 (1.0%)*	*15 (1.3%)*	*8 (0.7%)*
Reduced air travel			
Not done this	1,236 (48.6%)	547 (49.2%)	684 (6.7%)
Yes, for climate change	507 (19.9%)	242 (21.7%)	104 (9.5%)
Yes, for other reasons	748 (29.4%)	304 (27.3%)	265 (24.3%)
Yes, for climate and other reasons	52 (2.0%)	20 (1.8%)	38 (3.5%)
Missing	*35 (1.4%)*	*13 (1.2%)*	*9 (0.8%)*
Bought or hired electric or hybrid vehicle			
Not done this	2,248 (87.7%)	919 (82.3%)	1,018 (92.6%)
Yes, for climate change	223 (8.7%)	129 (11.6%)	38 (3.5%)
Yes, for other reasons	74 (2.9%)	57 (5.1%)	31 (2.8%)
Yes, for climate and other reasons	18 (0.7%)	12 (1.1%)	12 (1.1%)
Missing	*15 (0.6%)*	*9 (0.8%)*	*1 (0.1%)*
Bought foods produced locally			
Not done this	665 (26.1%)	338 (30.2%)	494 (45.1%)
Yes, for climate change	1,059 (41.5%)	385 (34.4%)	302 (27.6%)
Yes, for other reasons	710 (27.8%)	352 (31.5%)	216 (19.7%)
Yes, for climate and other reasons	116 (4.6%)	43 (3.9%)	84 (7.7%)
Missing	*28 (1.1%)*	*8 (0.7%)*	*4 (0.4%)*
Recycled or upcycled more			
Not done this	148 (5.8%)	107 (9.6%)	164 (15.0%)
Yes, for climate change	1,760 (68.7%)	648 (58.0%)	661 (60.4%)
Yes, for other reasons	541 (21.1%)	308 (27.6%)	156 (14.2%)
Yes, for climate and other reasons	112 (4.4%)	54 (4.8%)	114 (10.4%)
Missing	*17 (0.7%)*	*9 (0.8%)*	*5 (0.5%)*
Reduced amount of plastic used			
Not done this	243 (9.5%)	208 (18.6%)	236 (21.5%)

Yes, for climate change	1,864 (72.6%)	644 (57.4%)	712 (64.9%)
Yes, for other reasons	360 (14.0%)	221 (19.7%)	89 (8.1%)
Yes, for climate and other reasons	101 (3.9%)	48 (4.3%)	60 (5.5%)
Missing	*10 (0.4%)*	*5 (0.4%)*	*3 (0.3%)*
Chosen sustainably sourced items			
Not done this	754 (29.6%)	350 (31.4%)	419 (38.3%)
Yes, for climate change	1,357 (53.3%)	502 (45.1%)	543 (49.6%)
Yes, for other reasons	359 (14.1%)	219 (19.7%)	62 (5.7%)
Yes, for climate and other reasons	74 (2.9%)	42 (3.8%)	70 (6.4%)
Missing	*34 (1.3%)*	*13 (1.2%)*	*6 (0.5%)*
Improved insulation in home			
Not done this	1,507 (58.8%)	584 (52.1%)	932 (84.8%)
Yes, for climate change	503 (19.6%)	233 (20.8%)	56 (5.1%)
Yes, for other reasons	500 (19.5%)	271 (24.2%)	86 (7.8%)
Yes, for climate and other reasons	55 (2.1%)	33 (2.9%)	25 (2.3%)
Missing	*13 (0.5%)*	*5 (0.4%)*	*1 (0.1%)*
Installed solar panels [c]			
Not done this	2,311 (90.4%)	962 (86.4%)	>1,065 (>97.0%)
Yes, for climate change	148 (5.8%)	90 (8.1%)	10 (0.9%)
Yes, for other reasons	79 (3.1%)	50 (4.5%)	17 (1.5%)
Yes, for climate and other reasons	17 (0.7%)	11 (1.0%)	<5 (<0.5%)
Missing	*23 (0.9%)*	*13 (1.2%)*	*1 (0.1%)*
Started growing vegetables			
Not done this	1,493 (58.2%)	693 (61.9%)	819 (74.7%)
Yes, for climate change	348 (13.6%)	105 (9.4%)	64 (5.8%)
Yes, for other reasons	681 (26.6%)	312 (27.9%)	177 (16.2%)
Yes, for climate and other reasons	42 (1.6%)	10 (0.9%)	36 (3.3%)
Missing	*14 (0.5%)*	*6 (0.5%)*	*4 (0.4%)*
Planted tree(s)			
Not done this	1,721 (67.3%)	749 (67.5%)	903 (82.5%)
Yes, for climate change	404 (15.8%)	138 (12.4%)	70 (6.4%)
Yes, for other reasons	396 (15.5%)	205 (18.5%)	95 (8.7%)
Yes, for climate and other reasons	37 (1.4%)	18 (1.6%)	27 (2.5%)
Missing	*20 (0.8%)*	*16 (1.4%)*	*5 (0.5%)*
Avoided organisations that support fossil fuels			
Not done this	2,085 (82.1%)	885 (79.4%)	923 (84.1%)
Yes, for climate change	380 (15.0%)	193 (17.3%)	148 (13.5%)
Yes, for other reasons	61 (2.4%)	30 (2.7%)	9 (0.8%)
Yes, for climate and other reasons	14 (0.5%)	7 (0.6%)	17 (1.6%)
Missing	*38 (1.5%)*	*11 (1.0%)*	*3 (0.3%)*
Not had children, or reduced number of children planned [c]			
Not done this	>2,480 (>98.0%)	>1,095 (>98.0%)	949 (86.8%)
Yes, for climate change	10 (0.4%)	12 (1.1%)	40 (3.7%)

Yes, for other reasons	36 (1.4%)	<5 (<0.5%)	73 (6.7%)
Yes, for climate and other reasons	<5 (<0.2%)	<5 (<0.5%)	31 (2.8%)
Missing	*43 (1.7%)*	*9 (0.8%)*	*7 (0.6%)*
Other action [c]			
Not done this	>1,100 (>95.5%)	>625 (>94.5%)	>725 (>95.0%)
Yes, for climate change	33 (2.9%)	24 (3.6%)	28 (3.7%)
Yes, for other reasons	12 (1.0%)	7 (1.1%)	<5 (<0.5%)
Yes, for climate and other reasons	<5 (<0.5%)	<5 (<1.0%)	<5 (<0.5%)
Missing	*1,427 (55.4%)*	*465 (41.3%)*	*334 (30.4%)*
Eaten less or no meat and/or dairy			
Not done this	1,012 (42.0%)	500 (46.3%)	478 (46.0%)
Yes, for climate change	453 (18.8%)	150 (13.9%)	198 (19.1%)
Yes, for other reasons	685 (28.4%)	328 (30.4%)	163 (15.7%)
Yes, for climate and other reasons	261 (10.8%)	102 (9.4%)	200 (19.2%)
Missing	*167 (6.5%)*	*46 (4.1%)*	*61 (5.5%)*

[a] Total of 16 behaviours for G0 mothers and partners, 17 for G1 offspring (see table S2).

[b] Total of 11 behaviours for G0 mothers and partners, 12 for G1 offspring (see table S2).

[c] Note that some cells with counts < 5 have been altered to preserve participant anonymity.

Table S9: Results of the mothers ordinal regression models with 'belief that the climate is changing' as the outcome for four religious exposures (belief, identity [both Christian denominations combined together and separate], attendance, and latent classes). Odds ratios above 1 indicate an increased belief in climate change. The 'brant' column shows the *p*-value of the brant test for said variable. The '*p* total' column is the *p*-value for the overall association between the exposure and outcome (if three or more exposure levels). OR = Odds ratio; LCI = Lower 95% confidence interval; UCI = Upper 95% confidence interval.

**Exposure**	**Exposure level**	**Model**	***n***	**OR**	**LCI**	**UCI**	***p*-value**	**brant**	***p* total**
Belief (ref = No)	Not sure	Unadjusted	2569	0.849	0.658	1.097	0.2114	0.3858	0.4566
	Yes			0.920	0.722	1.173	0.5031	0.3691	
	Not sure	Adjusted	2569	0.929	0.713	1.209	0.5825	0.2858	0.8566
	Yes			0.967	0.752	1.245	0.7959	0.4273	
Identity (combined; ref = None)	Christian	Unadjusted	2542	0.709	0.565	0.890	0.0031	0.7306	NA
	Christian	Adjusted	2542	0.800	0.631	1.014	0.0645	0.7916	NA
Identity (separate; ref = None)	C of E	Unadjusted	2542	0.658	0.519	0.833	0.0005	0.8359	0.0025
	Catholic			1.002	0.651	1.542	0.9936	<0.0001	
	Other			0.814	0.575	1.154	0.2485	0.8613	
	C of E	Adjusted	2542	0.773	0.604	0.988	0.0394	0.8971	0.1706
	Catholic			0.999	0.641	1.557	0.9965	0.4791	
	Other			0.829	0.578	1.189	0.3085	0.8405	
Attendance (ref = Occasional/None)	Regular	Unadjusted	2542	1.671	1.216	2.297	0.0015	0.6789	NA
	Regular	Adjusted	2542	1.321	0.950	1.836	0.0978	0.6045	NA
Latent class (ref = "Atheist")	Agnostic	Unadjusted	2575	0.761	0.596	0.970	0.0272	0.1815	0.0363
	Moderately religious			0.918	0.711	1.186	0.5126	0.8034	
	Highly religious			1.195	0.847	1.686	0.3103	0.8956	
	Agnostic	Adjusted	2575	0.829	0.645	1.065	0.1429	0.0542	0.3763
	Moderately religious			1.008	0.774	1.312	0.9542	0.3591	
	Highly religious			1.013	0.708	1.449	0.9451	0.9934	

Table S10: Results of the mothers ordinal regression models with 'concerned about the impact of climate change' as the outcome for four religious exposures (belief, identity [both Christian denominations combined together and separate], attendance, and latent classes). Odds ratios above 1 indicate an increased concern regarding climate change. The 'brant' column shows the *p*-value of the brant test for said variable. The '*p* total' column is the *p*-value for the overall association between the exposure and outcome (if three or more exposure levels). OR = Odds ratio; LCI = Lower 95% confidence interval; UCI = Upper 95% confidence interval.

**Exposure**	**Exposure level**	**Model**	***n***	**OR**	**LCI**	**UCI**	***p*-value**	**brant**	***p* total**
Belief (ref = No)	Not sure	Unadjusted	2560	0.813	0.666	0.992	0.0419	0.3194	0.0407
	Yes			0.796	0.661	0.959	0.0164	0.0921	
	Not sure	Adjusted	2560	0.885	0.720	1.088	0.2466	0.5648	0.1903
	Yes			0.835	0.688	1.014	0.0691	0.2357	
Identity (combined; ref = None)	Christian	Unadjusted	2534	0.690	0.583	0.817	<0.0001	0.1342	NA
	Christian	Adjusted	2534	0.779	0.652	0.930	0.0057	0.4959	NA
Identity (separate; ref = None)	C of E	Unadjusted	2534	0.670	0.561	0.800	<0.0001	0.1118	0.0001
	Catholic			0.830	0.609	1.130	0.2362	0.1938	
	Other			0.700	0.538	0.911	0.0080	0.5101	
	C of E	Adjusted	2534	0.781	0.649	0.941	0.0095	0.4155	0.0379
	Catholic			0.851	0.618	1.171	0.3214	0.3128	
	Other			0.723	0.550	0.950	0.0199	0.6352	
Attendance (ref = Occasional/None)	Regular	Unadjusted	2534	1.395	1.122	1.734	0.0027	0.9986	NA
	Regular	Adjusted	2534	1.140	0.908	1.432	0.2580	0.8951	NA
Latent class (ref = "Atheist")	Agnostic	Unadjusted	2566	0.762	0.628	0.924	0.0057	0.4769	0.0050
	Moderately religious			0.795	0.653	0.968	0.0224	0.0703	
	Highly religious			1.077	0.838	1.382	0.5629	0.8177	
	Agnostic	Adjusted	2566	0.816	0.669	0.996	0.0454	0.7065	0.2238
	Moderately religious			0.869	0.709	1.065	0.1748	0.1340	
	Highly religious			0.937	0.722	1.217	0.6276	0.9611	

Table S11: Results of the mothers ordinal regression models with 'believes that humans are to blame for climate change' as the outcome for four religious exposures (belief, identity [both Christian denominations combined together and separate], attendance, and latent classes). Odds ratios above 1 indicate an increased belief that humans are to blame for climate change. The 'brant' column shows the *p*-value of the brant test for said variable. The '*p* total' column is the *p*-value for the overall association between the exposure and outcome (if three or more exposure levels). OR = Odds ratio; LCI = Lower 95% confidence interval; UCI = Upper 95% confidence interval.

**Exposure**	**Exposure level**	**Model**	***n***	**OR**	**LCI**	**UCI**	***p*-value**	**brant**	***p* total**
Belief (ref = No)	Not sure	Unadjusted	2561	0.673	0.555	0.816	0.0001	0.2299	<0.0001
	Yes			0.586	0.489	0.702	<0.0001	0.2339	
	Not sure	Adjusted	2561	0.737	0.606	0.897	0.0023	0.2575	<0.0001
	Yes			0.615	0.511	0.740	<0.0001	0.2771	
Identity (combined; ref = None)	Christian	Unadjusted	2535	0.613	0.520	0.721	<0.0001	0.3752	NA
	Christian	Adjusted	2535	0.702	0.594	0.830	<0.0001	0.2372	NA
Identity (separate; ref = None)	C of E	Unadjusted	2535	0.617	0.520	0.732	<0.0001	0.4009	<0.0001
	Catholic			0.580	0.431	0.781	0.0003	0.3127	
	Other			0.615	0.477	0.792	0.0002	0.7883	
	C of E	Adjusted	2535	0.729	0.611	0.869	0.0004	0.1824	0.0003
	Catholic			0.607	0.448	0.821	0.0012	0.4365	
	Other			0.658	0.508	0.852	0.0015	0.9234	
Attendance (ref = Occasional/None)	Regular	Unadjusted	2535	1.169	0.950	1.438	0.1407	0.5044	NA
	Regular	Adjusted	2535	0.977	0.789	1.210	0.8320	0.6776	NA
Latent class (ref = "Atheist")	Agnostic	Unadjusted	2567	0.705	0.585	0.849	0.0002	0.6848	<0.0001
	Moderately religious			0.645	0.533	0.781	<0.0001	0.6323	
	Highly religious			0.850	0.669	1.081	0.1846	0.6235	
	Agnostic	Adjusted	2567	0.766	0.634	0.926	0.0057	0.4085	0.0023
	Moderately religious			0.708	0.583	0.860	0.0005	0.5553	
	Highly religious			0.756	0.591	0.968	0.0264	0.8426	

Table S12: Results of the mothers multinomial regression models with 'thinks that personal actions will make a difference to long-term climate change' as the outcome for four religious exposures (belief, identity [both Christian denominations combined together and separate], attendance, and latent classes). The '*p* total' column is the *p*-value for the overall association between the exposure and outcome. RRR = Relative risk ratio; LCI = Lower 95% confidence interval; UCI = Upper 95% confidence interval.

**Exposure**	**Exposure level**	**Outcome level (ref = No)**	**Model**	***n***	**RRR**	**LCI**	**UCI**	***p*-value**	***p* total**
Belief (ref = No)	Not sure	Not sure	Unadjusted	2560	2.047	1.382	3.034	0.0004	<0.0001
		Yes			2.095	1.455	3.018	0.0001	
	Yes	Not sure			1.423	1.001	2.024	0.0494	
		Yes			1.857	1.349	2.557	0.0001	
	Not sure	Not sure	Adjusted	2560	2.125	1.424	3.170	0.0002	<0.0001
		Yes			2.146	1.481	3.110	0.0001	
	Yes	Not sure			1.460	1.018	2.094	0.0398	
		Yes			1.877	1.353	2.605	0.0002	
Identity (combined; ref = None)	Christian	Not sure	Unadjusted	2534	1.333	0.969	1.833	0.0768	0.0037
		Yes			1.601	1.195	2.144	0.0016	
	Christian	Not sure	Adjusted	2534	1.335	0.961	1.855	0.0853	0.0046
		Yes			1.611	1.191	2.180	0.0020	
Identity (separate; ref = None)	C of E	Not sure	Unadjusted	2534	1.332	0.950	1.869	0.0967	0.0736
		Yes			1.595	1.169	2.177	0.0032	
	Catholic	Not sure			1.508	0.775	2.934	0.2263	
		Yes			1.847	0.997	3.422	0.0511	
	Other	Not sure			1.251	0.744	2.105	0.3982	
		Yes			1.504	0.933	2.423	0.0937	
	C of E	Not sure	Adjusted	2534	1.337	0.942	1.897	0.1036	0.0870
		Yes			1.609	1.167	2.218	0.0037	
	Catholic	Not sure			1.483	0.755	2.912	0.2520	
		Yes			1.815	0.971	3.392	0.0616	
	Other	Not sure			1.249	0.735	2.122	0.4114	
		Yes			1.513	0.930	2.461	0.0951	
Attendance (ref = Occasional/None)	Regular	Not sure	Unadjusted	2534	1.270	0.779	2.072	0.3380	0.0299
		Yes			1.620	1.032	2.542	0.0361	
	Regular	Not sure	Adjusted	2534	1.211	0.734	1.998	0.4534	0.0273
		Yes			1.600	1.008	2.538	0.0460	
	Agnostic	Not sure	Unadjusted	2566	1.702	1.157	2.502	0.0069	0.0054

Latent class (ref = “Atheist”)		Yes			1.619	1.132	2.316	0.0083	
	Moderately religious	Not sure			1.158	0.786	1.706	0.4592	
		Yes			1.491	1.050	2.116	0.0256	
	Highly religious	Not sure			1.669	0.977	2.851	0.0609	
		Yes			1.931	1.176	3.171	0.0093	
	Agnostic	Not sure	Adjusted	2566	1.729	1.169	2.557	0.0061	0.0075
		Yes			1.643	1.143	2.363	0.0073	
	Moderately religious	Not sure			1.180	0.795	1.750	0.4111	
		Yes			1.498	1.049	2.141	0.0264	
	Highly religious	Not sure			1.613	0.934	2.787	0.0863	
		Yes			1.905	1.148	3.159	0.0126	

Table S13: Results of the mothers linear regression models with ‘total number of actions performed due to climate change’ as the outcome for four religious exposures (belief, identity [both Christian denominations combined together and separate], attendance, and latent classes). Values above 0 indicate an increased number of pro-environmental actions performed. The ‘*p* total’ column is the *p*-value for the overall association between the exposure and outcome (if three or more exposure levels). *b* = Mean difference; LCI = Lower 95% confidence interval; UCI = Upper 95% confidence interval.

**Exposure**	**Exposure level**	**Model**	***n***	***b***	**LCI**	**UCI**	***p*-value**	***p* total**
Belief (ref = No)	Not sure	Unadjusted	2218	-0.430	-0.851	-0.010	0.0448	0.1324
	Yes			-0.255	-0.648	0.139	0.2048	
	Not sure	Adjusted	2218	-0.253	-0.664	0.159	0.2285	0.4841
	Yes			-0.140	-0.529	0.249	0.4797	
Identity (combined; ref = None)	Christian	Unadjusted	2195	-0.673	-1.028	-0.319	0.0002	NA
	Christian	Adjusted	2195	-0.421	-0.772	-0.069	0.0190	NA
Identity (separate; ref = None)	C of E	Unadjusted	2195	-0.804	-1.176	-0.432	<0.0001	0.0002
	Catholic			-0.117	-0.770	0.536	0.7259	
	Other			-0.444	-0.995	0.106	0.1134	
	C of E	Adjusted	2195	-0.501	-0.870	-0.133	0.0077	0.0489
	Catholic			-0.065	-0.705	0.576	0.8431	
	Other			-0.293	-0.835	0.249	0.2892	
Attendance (ref = Occasional/None)	Regular	Unadjusted	2197	1.084	0.636	1.532	<0.0001	NA
	Regular	Adjusted	2197	0.693	0.247	1.139	0.0024	NA
Latent class (ref = “Atheist”)	Agnostic	Unadjusted	2224	-0.717	-1.121	-0.314	0.0005	<0.0001
	Moderately religious			-0.428	-0.843	-0.013	0.0434	
	Highly religious			0.736	0.215	1.257	0.0057	
	Agnostic	Adjusted	2224	-0.543	-0.938	-0.148	0.0071	0.0007
	Moderately religious			-0.243	-0.652	0.166	0.2446	
	Highly religious			0.505	-0.012	1.022	0.0555	

Table S14: Results of the mothers Poisson regression models with 'total number of actions performed due to climate change' as the outcome for four religious exposures (belief, identity [both Christian denominations combined together and separate], attendance, and latent classes). Incidence rate ratios above 1 indicate an increased number of pro-environmental actions performed. The '*p* total' column is the *p*-value for the overall association between the exposure and outcome (if three or more exposure levels). IRR = Incidence rate ratio; LCI = Lower 95% confidence interval; UCI = Upper 95% confidence interval.

Exposure	Exposure level	Model	*n*	IRR	LCI	UCI	*p*-value	*p* total
Belief (ref = No)	Not sure	Unadjusted	2218	0.929	0.888	0.973	0.0016	0.0066
	Yes			0.958	0.919	0.999	0.0471	
	Not sure	Adjusted	2218	0.959	0.916	1.004	0.0720	0.1984
	Yes			0.978	0.937	1.021	0.3040	
Identity (combined; ref = None)	Christian	Unadjusted	2195	0.894	0.861	0.928	<0.0001	NA
	Christian	Adjusted	2195	0.933	0.898	0.970	0.0004	NA
Identity (separate; ref = None)	C of E	Unadjusted	2195	0.873	0.839	0.909	<0.0001	<0.0001
	Catholic			0.982	0.916	1.052	0.5966	
	Other			0.930	0.877	0.986	0.0159	
	C of E	Adjusted	2195	0.919	0.883	0.958	0.0001	0.0005
	Catholic			0.991	0.924	1.062	0.7962	
	Other			0.953	0.897	1.012	0.1149	
Attendance (ref = Occasional/None)	Regular	Unadjusted	2197	1.191	1.137	1.247	<0.0001	NA
	Regular	Adjusted	2197	1.115	1.064	1.170	<0.0001	NA
Latent class (ref = "Atheist")	Agnostic	Unadjusted	2224	0.882	0.844	0.922	<0.0001	<0.0001
	Moderately religious			0.930	0.888	0.973	0.0016	
	Highly religious			1.121	1.063	1.183	<0.0001	
	Agnostic	Adjusted	2224	0.909	0.869	0.951	<0.0001	<0.0001
	Moderately religious			0.960	0.916	1.005	0.0778	
	Highly religious			1.078	1.021	1.139	0.0068	

Table S15: Results of the mothers zero-inflated Poisson regression models with 'total number of actions performed due to climate change' as the outcome for four religious exposures (belief, identity [both Christian denominations combined together and separate], attendance, and latent classes). Incidence rate ratios above 1 indicate an increased number of pro-environmental actions performed, while odds ratios above 1 indicate an increased probability of excess zeros. IRR = Incidence rate ratio; LCI = Lower 95% confidence interval; UCI = Upper 95% confidence interval; OR = Odds ratio.

**Exposure**	**Exposure level**	**Model**	***n***	**IRR**	**IRR LCI**	**IRR UCI**	**IRR *p***	**OR**	**OR LCI**	**OR UCI**	**OR *p***
Belief (ref = No)	Not sure	Unadjusted	2218	0.925	0.884	0.968	0.0008	0.965	0.702	1.327	0.8275
	Yes			0.926	0.888	0.966	0.0003	0.762	0.560	1.038	0.0849
	Not sure	Adjusted	2218	0.950	0.907	0.994	0.0280	0.929	0.669	1.289	0.6582
	Yes			0.945	0.905	0.987	0.0104	0.757	0.549	1.043	0.0888
Identity (combined; ref = None)	Christian	Unadjusted	2195	0.901	0.868	0.936	<0.0001	1.066	0.802	1.416	0.6611
	Christian	Adjusted	2195	0.931	0.895	0.968	0.0003	1.000	0.744	1.344	0.9992
Identity (separate; ref = None)	C of E	Unadjusted	2195	0.889	0.855	0.926	<0.0001	1.152	0.858	1.548	0.3461
	Catholic			0.980	0.914	1.050	0.5570	0.983	0.579	1.669	0.9490
	Other			0.901	0.849	0.956	0.0006	0.748	0.461	1.214	0.2401
	C of E	Adjusted	2195	0.925	0.888	0.964	0.0002	0.868	0.724	1.039	0.6809
	Catholic			0.987	0.920	1.058	0.7103	1.053	0.986	1.125	0.9850
	Other			0.918	0.865	0.976	0.0058	1.119	1.018	1.230	0.1713
Attendance (ref = Occasional/None)	Regular	Unadjusted	2197	1.097	1.047	1.149	0.0001	0.431	0.270	0.686	0.0004
	Regular	Adjusted	2197	1.050	1.002	1.102	0.0427	0.506	0.313	0.816	0.0052
Latent class (ref = "Atheist")	Agnostic	Unadjusted	2224	0.887	0.848	0.927	<0.0001	1.039	0.765	1.410	0.8078
	Moderately religious			0.915	0.874	0.957	0.0001	0.886	0.640	1.226	0.4654
	Highly religious			1.033	0.979	1.090	0.2383	0.438	0.262	0.732	0.0016
	Agnostic	Adjusted	2224	0.908	0.868	0.950	<0.0001	0.994	0.726	1.361	0.9711
	Moderately religious			0.939	0.896	0.983	0.0074	0.843	0.602	1.179	0.3183
	Highly religious			1.011	0.957	1.068	0.7072	0.491	0.290	0.834	0.0084

Table S16: Results of the mothers linear regression models with 'total number of actions performed due to climate change (excluding ones which may be prohibitively costly)' as the outcome for four religious exposures (belief, identity [both Christian denominations combined together and separate], attendance, and latent classes). Values above 0 indicate an increased number of pro-environmental actions performed. The '*p* total' column is the *p*-value for the overall association between the exposure and outcome (if three or more exposure levels). *b* = Mean difference; LCI = Lower 95% confidence interval; UCI = Upper 95% confidence interval.

**Exposure**	**Exposure level**	**Model**	***n***	***b***	**LCI**	**UCI**	***p*-value**	***p* total**
Belief (ref = No)	Not sure	Unadjusted	2268	-0.302	-0.646	0.041	0.0846	0.2204
	Yes			-0.193	-0.514	0.128	0.2392	
	Not sure	Adjusted	2268	-0.160	-0.498	0.177	0.3516	0.6463
	Yes			-0.098	-0.416	0.221	0.5480	
Identity (combined; ref = None)	Christian	Unadjusted	2244	-0.556	-0.846	-0.266	0.0002	NA
	Christian	Adjusted	2244	-0.349	-0.639	-0.060	0.0179	NA
Identity (separate; ref = None)	C of E	Unadjusted	2244	-0.637	-0.942	-0.333	<0.0001	0.0005
	Catholic			-0.183	-0.717	0.350	0.5008	
	Other			-0.433	-0.883	0.016	0.0589	
	C of E	Adjusted	2244	-0.393	-0.697	-0.089	0.0112	0.0777
	Catholic			-0.120	-0.646	0.406	0.6553	
	Other			-0.306	-0.751	0.139	0.1781	
Attendance (ref = Occasional/None)	Regular	Unadjusted	2246	0.852	0.484	1.221	<0.0001	NA
	Regular	Adjusted	2246	0.563	0.196	0.931	0.0027	NA
Latent class (ref = "Atheist")	Agnostic	Unadjusted	2274	-0.527	-0.857	-0.197	0.0017	<0.0001
	Moderately religious			-0.289	-0.629	0.050	0.0944	
	Highly religious			0.543	0.116	0.970	0.0127	
	Agnostic	Adjusted	2274	-0.380	-0.704	-0.056	0.0216	0.0049
	Moderately religious			-0.149	-0.485	0.186	0.3824	
	Highly religious			0.377	-0.048	0.802	0.0818	

Table S17: Results of the mothers Poisson regression models with 'total number of actions performed due to climate change (excluding ones which may be prohibitively costly)' as the outcome for four religious exposures (belief, identity [both Christian denominations combined together and separate], attendance, and latent classes). Incidence rate ratios above 1 indicate an increased number of pro-environmental actions performed. The '*p* total' column is the *p*-value for the overall association between the exposure and outcome (if three or more exposure levels). IRR = Incidence rate ratio; LCI = Lower 95% confidence interval; UCI = Upper 95% confidence interval.

**Exposure**	**Exposure level**	**Model**	***n***	**IRR**	**LCI**	**UCI**	***p*-value**	***p* total**
Belief (ref = No)	Not sure	Unadjusted	2268	0.943	0.899	0.989	0.0165	0.0536
	Yes			0.964	0.922	1.008	0.1032	
	Not sure	Adjusted	2268	0.970	0.924	1.018	0.2156	0.4631
	Yes			0.982	0.938	1.028	0.4371	
Identity (combined; ref = None)	Christian	Unadjusted	2244	0.899	0.864	0.936	<0.0001	NA
	Christian	Adjusted	2244	0.936	0.899	0.975	0.0016	NA
Identity (separate; ref = None)	C of E	Unadjusted	2244	0.885	0.848	0.923	<0.0001	<0.0001
	Catholic			0.967	0.899	1.040	0.3670	
	Other			0.922	0.866	0.981	0.0107	
	C of E	Adjusted	2244	0.928	0.889	0.969	0.0007	0.0066
	Catholic			0.979	0.909	1.055	0.5776	
	Other			0.944	0.885	1.006	0.0750	
Attendance (ref = Occasional/None)	Regular	Unadjusted	2246	1.171	1.115	1.230	<0.0001	NA
	Regular	Adjusted	2246	1.108	1.053	1.165	0.0001	NA
Latent class (ref = "Atheist")	Agnostic	Unadjusted	2274	0.900	0.859	0.944	<0.0001	<0.0001
	Moderately religious			0.945	0.901	0.991	0.0208	
	Highly religious			1.103	1.042	1.168	0.0008	
	Agnostic	Adjusted	2274	0.926	0.884	0.971	0.0016	<0.0001
	Moderately religious			0.971	0.925	1.020	0.2404	
	Highly religious			1.068	1.008	1.133	0.0264	

Table S18: Results of the mothers zero-inflated Poisson regression models with 'total number of actions performed due to climate change (excluding ones which may be prohibitively costly)' as the outcome for four religious exposures (belief, identity [both Christian denominations combined together and separate], attendance, and latent classes). Incidence rate ratios above 1 indicate an increased number of pro-environmental actions performed, while odds ratios above 1 indicate an increased probability of excess zeros. IRR = Incidence rate ratio; LCI = Lower 95% confidence interval; UCI = Upper 95% confidence interval; OR = Odds ratio.

**Exposure**	**Exposure level**	**Model**	***n***	**IRR**	**IRR LCI**	**IRR UCI**	**IRR *p***	**OR**	**OR LCI**	**OR UCI**	**OR *p***
Belief (ref = No)	Not sure	Unadjusted	2268	0.930	0.886	0.976	0.0031	0.906	0.664	1.237	0.5357
	Yes			0.930	0.889	0.972	0.0015	0.768	0.570	1.035	0.0833
	Not sure	Adjusted	2268	0.953	0.907	1.000	0.0524	0.880	0.639	1.213	0.4351
	Yes			0.948	0.906	0.993	0.0228	0.759	0.557	1.035	0.0809
Identity (combined; ref = None)	Christian	Unadjusted	2244	0.907	0.872	0.944	<0.0001	1.068	0.810	1.409	0.6396
	Christian	Adjusted	2244	0.935	0.897	0.975	0.0015	1.004	0.753	1.338	0.9804
Identity (separate; ref = None)	C of E	Unadjusted	2244	0.889	0.855	0.926	<0.0001	1.152	0.858	1.548	0.3461
	Catholic			0.980	0.914	1.050	0.5570	0.983	0.579	1.669	0.9490
	Other			0.901	0.849	0.956	0.0006	0.748	0.461	1.214	0.2401
	C of E	Adjusted	2244	0.925	0.888	0.964	0.0002	0.868	0.724	1.039	0.6809
	Catholic			0.987	0.920	1.058	0.7103	1.053	0.986	1.125	0.9850
	Other			0.918	0.865	0.976	0.0058	1.119	1.018	1.230	0.1713
Attendance (ref = Occasional/None)	Regular	Unadjusted	2246	1.075	1.023	1.129	0.0043	0.436	0.277	0.686	0.0003
	Regular	Adjusted	2246	1.037	0.985	1.091	0.1645	0.495	0.311	0.788	0.0031
Latent class (ref = "Atheist")	Agnostic	Unadjusted	2274	0.903	0.861	0.947	<0.0001	1.023	0.759	1.377	0.8818
	Moderately religious			0.926	0.883	0.972	0.0019	0.865	0.630	1.188	0.3714
	Highly religious			1.016	0.959	1.076	0.5838	0.468	0.287	0.763	0.0023
	Agnostic	Adjusted	2274	0.924	0.881	0.970	0.0013	0.987	0.727	1.340	0.9349
	Moderately religious			0.948	0.903	0.996	0.0342	0.827	0.596	1.148	0.2571
	Highly religious			0.997	0.940	1.058	0.9318	0.509	0.308	0.842	0.0085

Table S19: Results of the mothers multinomial regression models for each of the individual climate change action outcomes for four religious exposures (belief, identity [both Christian denominations combined together and separate], attendance, and latent classes). The '*p* total' column is the *p*-value for the overall association between the exposure and outcome. RRR = Relative risk ratio; LCI = Lower 95% confidence interval; UCI = Upper 95% confidence interval.

**Exposure**	**Exposure level**	**Outcome level (ref = No)**	**Model**	***n***	**RRR**	**LCI**	**UCI**	***p*-value**	***p* total**
Changed the way travel locally									
Belief (ref = No)	Not sure	For climate reasons	Unadjusted	2552	0.729	0.562	0.948	0.0181	0.0508
		For other reasons			1.033	0.784	1.363	0.8164	
		For climate and other			0.941	0.534	1.658	0.8336	
	Yes	For climate reasons			0.748	0.589	0.950	0.0174	
		For other reasons			0.806	0.617	1.053	0.1135	
		For climate and other			0.703	0.403	1.224	0.2127	
	Not sure	For climate reasons	Adjusted	2552	0.820	0.624	1.077	0.1541	0.1803
		For other reasons			1.105	0.832	1.468	0.4917	
		For climate and other			1.063	0.594	1.902	0.8360	
	Yes	For climate reasons			0.799	0.621	1.028	0.0808	
		For other reasons			0.840	0.638	1.106	0.2146	
		For climate and other			0.756	0.427	1.338	0.3368	
Identity (combined; ref = None)	Christian	For climate reasons	Unadjusted	2526	0.684	0.551	0.849	0.0006	0.0010
		For other reasons			0.740	0.586	0.934	0.0114	
		For climate and other			0.635	0.395	1.021	0.0607	
	Christian	For climate reasons	Adjusted	2526	0.798	0.635	1.004	0.0539	0.0877
		For other reasons			0.791	0.620	1.009	0.0592	
		For climate and other			0.714	0.436	1.169	0.1811	
Identity (separate; ref = None)	C of E	For climate reasons	Unadjusted	2526	0.625	0.496	0.788	0.0001	0.0043
		For other reasons			0.735	0.575	0.940	0.0141	
		For climate and other			0.622	0.375	1.030	0.0653	
	Catholic	For climate reasons			1.038	0.705	1.527	0.8507	
		For other reasons			0.874	0.562	1.358	0.5487	
		For climate and other			0.668	0.252	1.773	0.4179	
	Other	For climate reasons			0.750	0.534	1.052	0.0952	
		For other reasons			0.687	0.470	1.004	0.0524	
		For climate and other			0.672	0.311	1.453	0.3127	
	C of E	For climate reasons	Adjusted	2526	0.762	0.597	0.972	0.0288	0.3634
		For other reasons			0.807	0.625	1.043	0.1008	
		For climate and other			0.730	0.432	1.233	0.2391	

Exposure	Exposure level	Outcome level (ref = No)	Model	*n*	RRR	LCI	UCI	*p*-value	*p* total
	Catholic	For climate reasons			1.037	0.692	1.554	0.8606	
		For other reasons			0.831	0.528	1.308	0.4241	
		For climate and other			0.640	0.238	1.722	0.3768	
	Other	For climate reasons			0.799	0.561	1.138	0.2130	
		For other reasons			0.703	0.476	1.038	0.0763	
		For climate and other			0.677	0.307	1.495	0.3349	
Attendance (ref = Occasional/None)	Regular	For climate reasons	Unadjusted	2525	2.020	1.555	2.625	<0.0001	<0.0001
		For other reasons			1.129	0.820	1.553	0.4565	
		For climate and other			1.309	0.695	2.467	0.4047	
	Regular	For climate reasons	Adjusted	2525	1.611	1.222	2.124	0.0007	0.0059
		For other reasons			0.980	0.705	1.361	0.9023	
		For climate and other			1.065	0.556	2.042	0.8490	
Latent class (ref = "Atheist")	Agnostic	For climate reasons	Unadjusted	2558	0.691	0.531	0.900	0.0061	<0.0001
		For other reasons			1.038	0.794	1.356	0.7874	
		For climate and other			0.783	0.453	1.353	0.3801	
	Moderately religious	For climate reasons			0.709	0.544	0.922	0.0104	
		For other reasons			0.779	0.585	1.037	0.0871	
		For climate and other			0.415	0.212	0.813	0.0104	
	Highly religious	For climate reasons			1.502	1.107	2.038	0.0090	
		For other reasons			1.143	0.798	1.637	0.4673	
		For climate and other			1.116	0.563	2.211	0.7540	
	Agnostic	For climate reasons	Adjusted	2558	0.747	0.569	0.982	0.0367	0.0128
		For other reasons			1.078	0.819	1.417	0.5934	
		For climate and other			0.815	0.466	1.426	0.4738	
	Moderately religious	For climate reasons			0.799	0.608	1.051	0.1085	
		For other reasons			0.840	0.626	1.126	0.2430	
		For climate and other			0.455	0.230	0.901	0.0239	
	Highly religious	For climate reasons			1.303	0.945	1.797	0.1059	
		For other reasons			1.044	0.721	1.512	0.8213	
		For climate and other			0.972	0.481	1.962	0.9365	
Reduced household waste									
Belief (ref = No)	Not sure	For climate reasons	Unadjusted	2560	0.837	0.579	1.211	0.3453	0.2014
		For other reasons			0.772	0.507	1.176	0.2283	

**Exposure**	**Exposure level**	**Outcome level (ref = No)**	**Model**	***n***	**RRR**	**LCI**	**UCI**	***p*-value**	***p* total**
		For climate and other			0.674	0.377	1.205	0.1832	
	Yes	For climate reasons			1.024	0.715	1.467	0.8955	
		For other reasons			1.063	0.711	1.591	0.7650	
		For climate and other			0.584	0.327	1.042	0.0689	
	Not sure	For climate reasons	Adjusted	2560	0.843	0.579	1.227	0.3718	0.2425
		For other reasons			0.754	0.491	1.157	0.1960	
		For climate and other			0.696	0.383	1.263	0.2328	
	Yes	For climate reasons			1.007	0.697	1.455	0.9703	
		For other reasons			1.030	0.682	1.556	0.8885	
		For climate and other			0.587	0.323	1.065	0.0798	
Identity (combined; ref = None)	Christian	For climate reasons	Unadjusted	2534	0.884	0.640	1.221	0.4544	0.0013
		For other reasons			1.031	0.714	1.488	0.8706	
		For climate and other			0.428	0.261	0.701	0.0007	
	Christian	For climate reasons	Adjusted	2534	0.923	0.662	1.285	0.6338	0.0076
		For other reasons			1.025	0.702	1.495	0.8988	
		For climate and other			0.464	0.278	0.774	0.0033	
Identity (separate; ref = None)	C of E	For climate reasons	Unadjusted	2534	0.952	0.676	1.340	0.7769	0.0036
		For other reasons			1.143	0.776	1.685	0.4978	
		For climate and other			0.511	0.303	0.860	0.0116	
	Catholic	For climate reasons			1.094	0.591	2.024	0.7751	
		For other reasons			1.184	0.593	2.363	0.6319	
		For climate and other			0.255	0.069	0.942	0.0404	
	Other	For climate reasons			0.613	0.393	0.958	0.0317	
		For other reasons			0.654	0.387	1.104	0.1118	
		For climate and other			0.258	0.109	0.607	0.0019	
	C of E	For climate reasons	Adjusted	2534	1.016	0.715	1.444	0.9297	0.0064
		For other reasons			1.161	0.779	1.730	0.4633	
		For climate and other			0.570	0.332	0.979	0.0415	
	Catholic	For climate reasons			1.124	0.600	2.106	0.7151	
		For other reasons			1.185	0.586	2.398	0.6362	
		For climate and other			0.283	0.075	1.058	0.0605	
	Other	For climate reasons			0.580	0.366	0.918	0.0200	
		For other reasons			0.587	0.343	1.006	0.0526	
		For climate and other			0.245	0.102	0.588	0.0016	

**Exposure**	**Exposure level**	**Outcome level (ref = No)**	**Model**	***n***	**RRR**	**LCI**	**UCI**	***p*-value**	***p* total**
Attendance (ref = Occasional/None)	Regular	For climate reasons	Unadjusted	2534	0.998	0.670	1.487	0.9924	0.2957
		For other reasons			0.789	0.496	1.253	0.3146	
		For climate and other			0.680	0.328	1.411	0.3003	
	Regular	For climate reasons	Adjusted	2534	0.836	0.553	1.263	0.3946	0.1981
		For other reasons			0.680	0.421	1.098	0.1148	
		For climate and other			0.532	0.251	1.123	0.0979	
Latent class (ref = "Atheist")	Agnostic	For climate reasons	Unadjusted	2566	0.859	0.600	1.229	0.4043	0.0985
		For other reasons			0.829	0.552	1.243	0.3637	
		For climate and other			0.478	0.264	0.862	0.0142	
	Moderately religious	For climate reasons			1.041	0.711	1.524	0.8377	
		For other reasons			1.006	0.656	1.542	0.9787	
		For climate and other			0.436	0.226	0.841	0.0132	
	Highly religious	For climate reasons			0.962	0.604	1.533	0.8709	
		For other reasons			0.776	0.454	1.326	0.3537	
		For climate and other			0.571	0.265	1.231	0.1530	
	Agnostic	For climate reasons	Adjusted	2566	0.867	0.602	1.247	0.4413	0.1293
		For other reasons			0.807	0.534	1.218	0.3071	
		For climate and other			0.504	0.276	0.919	0.0255	
	Moderately religious	For climate reasons			1.064	0.721	1.569	0.7561	
		For other reasons			1.009	0.653	1.559	0.9683	
		For climate and other			0.458	0.235	0.894	0.0222	
	Highly religious	For climate reasons			0.833	0.515	1.348	0.4566	
		For other reasons			0.680	0.391	1.182	0.1715	
		For climate and other			0.473	0.215	1.042	0.0631	
Reduced energy use at home									
Belief (ref = No)	Not sure	For climate reasons	Unadjusted	2562	1.004	0.774	1.302	0.9778	0.2090
		For other reasons			1.194	0.891	1.601	0.2347	
		For climate and other			1.027	0.604	1.748	0.9204	
	Yes	For climate reasons			1.295	1.013	1.657	0.0395	
		For other reasons			1.272	0.961	1.684	0.0922	
		For climate and other			1.123	0.679	1.858	0.6512	
	Not sure	For climate reasons	Adjusted	2562	1.052	0.806	1.374	0.7089	0.2406
		For other reasons			1.173	0.870	1.582	0.2963	

Exposure	Exposure level	Outcome level (ref = No)	Model	*n*	RRR	LCI	UCI	*p*-value	*p* total
		For climate and other			1.031	0.599	1.773	0.9134	
	Yes	For climate reasons			1.301	1.009	1.678	0.0424	
		For other reasons			1.197	0.897	1.597	0.2223	
		For climate and other			1.079	0.643	1.811	0.7729	
Identity (combined; ref = None)	Christian	For climate reasons	Unadjusted	2535	0.938	0.750	1.174	0.5773	0.1048
		For other reasons			1.081	0.838	1.395	0.5484	
		For climate and other			0.640	0.416	0.986	0.0428	
	Christian	For climate reasons	Adjusted	2535	1.007	0.798	1.271	0.9522	0.2231
		For other reasons			1.037	0.797	1.350	0.7859	
		For climate and other			0.651	0.416	1.017	0.0595	
Identity (separate; ref = None)	C of E	For climate reasons	Unadjusted	2535	0.872	0.689	1.103	0.2531	0.0708
		For other reasons			1.099	0.842	1.435	0.4864	
		For climate and other			0.678	0.431	1.065	0.0917	
	Catholic	For climate reasons			1.169	0.767	1.780	0.4672	
		For other reasons			1.080	0.665	1.753	0.7557	
		For climate and other			0.501	0.185	1.356	0.1736	
	Other	For climate reasons			1.115	0.786	1.583	0.5419	
		For other reasons			0.997	0.664	1.496	0.9873	
		For climate and other			0.547	0.251	1.194	0.1297	
	C of E	For climate reasons	Adjusted	2535	0.957	0.749	1.223	0.7272	0.2508
		For other reasons			1.063	0.807	1.400	0.6631	
		For climate and other			0.700	0.438	1.119	0.1361	
	Catholic	For climate reasons			1.245	0.809	1.915	0.3195	
		For other reasons			1.108	0.676	1.816	0.6839	
		For climate and other			0.556	0.203	1.526	0.2548	
	Other	For climate reasons			1.082	0.755	1.550	0.6682	
		For other reasons			0.881	0.581	1.335	0.5500	
		For climate and other			0.488	0.221	1.080	0.0768	
Attendance (ref = Occasional/None)	Regular	For climate reasons	Unadjusted	2535	1.650	1.219	2.233	0.0012	0.0002
		For other reasons			0.967	0.675	1.384	0.8526	
		For climate and other			1.168	0.628	2.173	0.6245	
	Regular	For climate reasons	Adjusted	2535	1.471	1.077	2.010	0.0152	0.0093
		For other reasons			0.963	0.666	1.393	0.8422	
		For climate and other			1.059	0.561	1.997	0.8604	

Exposure	Exposure level	Outcome level (ref = No)	Model	*n*	RRR	LCI	UCI	*p*-value	*p* total
Latent class (ref = "Atheist")	Agnostic	For climate reasons	Unadjusted	2568	1.010	0.783	1.303	0.9392	0.0231
		For other reasons			1.071	0.807	1.420	0.6369	
		For climate and other			0.842	0.498	1.422	0.5197	
	Moderately religious	For climate reasons			1.126	0.867	1.463	0.3729	
		For other reasons			1.050	0.783	1.409	0.7448	
		For climate and other			0.820	0.474	1.418	0.4769	
	Highly religious	For climate reasons			1.824	1.278	2.603	0.0009	
		For other reasons			1.186	0.786	1.789	0.4153	
		For climate and other			1.530	0.794	2.948	0.2040	
	Agnostic	For climate reasons	Adjusted	2568	1.059	0.817	1.374	0.6638	0.1325
		For other reasons			1.065	0.798	1.421	0.6702	
		For climate and other			0.871	0.511	1.483	0.6106	
	Moderately religious	For climate reasons			1.145	0.875	1.497	0.3230	
		For other reasons			0.975	0.722	1.317	0.8696	
		For climate and other			0.797	0.455	1.393	0.4253	
	Highly religious	For climate reasons			1.676	1.164	2.413	0.0055	
		For other reasons			1.136	0.745	1.732	0.5523	
		For climate and other			1.395	0.712	2.733	0.3324	
Changed what buy									
Belief (ref = No)	Not sure	For climate reasons	Unadjusted	2545	0.797	0.625	1.018	0.0696	0.1149
		For other reasons			1.128	0.830	1.532	0.4410	
		For climate and other			1.040	0.616	1.757	0.8828	
	Yes	For climate reasons			0.966	0.767	1.215	0.7652	
		For other reasons			1.227	0.917	1.641	0.1688	
		For climate and other			0.869	0.517	1.459	0.5948	
	Not sure	For climate reasons	Adjusted	2545	0.863	0.669	1.112	0.2548	0.2979
		For other reasons			1.155	0.843	1.581	0.3697	
		For climate and other			1.119	0.654	1.917	0.6815	
	Yes	For climate reasons			1.017	0.799	1.295	0.8898	
		For other reasons			1.197	0.886	1.616	0.2414	
		For climate and other			0.888	0.520	1.516	0.6632	
Identity (combined; ref = None)	Christian	For climate reasons	Unadjusted	2518	0.720	0.582	0.889	0.0023	0.0005
		For other reasons			1.017	0.778	1.329	0.9034	

Exposure	Exposure level	Outcome level (ref = No)	Model	*n*	RRR	LCI	UCI	*p*-value	*p* total
		For climate and other			0.546	0.353	0.843	0.0064	
	Christian	For climate reasons	Adjusted	2518	0.818	0.655	1.023	0.0779	0.0223
		For other reasons			1.056	0.800	1.394	0.7026	
		For climate and other			0.585	0.373	0.920	0.0201	
Identity (separate; ref = None)	C of E	For climate reasons	Unadjusted	2518	0.679	0.543	0.848	0.0006	0.0031
		For other reasons			1.024	0.775	1.353	0.8700	
		For climate and other			0.571	0.362	0.901	0.0162	
	Catholic	For climate reasons			0.927	0.621	1.383	0.7109	
		For other reasons			1.163	0.711	1.903	0.5476	
		For climate and other			0.681	0.285	1.627	0.3874	
	Other	For climate reasons			0.794	0.575	1.095	0.1596	
		For other reasons			0.909	0.602	1.370	0.6478	
		For climate and other			0.361	0.155	0.842	0.0184	
	C of E	For climate reasons	Adjusted	2518	0.800	0.633	1.010	0.0610	0.0811
		For other reasons			1.097	0.822	1.465	0.5302	
		For climate and other			0.636	0.396	1.022	0.0616	
	Catholic	For climate reasons			0.955	0.631	1.446	0.8268	
		For other reasons			1.123	0.678	1.861	0.6515	
		For climate and other			0.691	0.284	1.678	0.4137	
	Other	For climate reasons			0.818	0.584	1.145	0.2419	
		For other reasons			0.855	0.560	1.305	0.4675	
		For climate and other			0.340	0.144	0.805	0.0141	
Attendance (ref = Occasional/None)	Regular	For climate reasons	Unadjusted	2518	2.303	1.704	3.114	<0.0001	<0.0001
		For other reasons			1.392	0.954	2.032	0.0863	
		For climate and other			1.237	0.627	2.441	0.5390	
	Regular	For climate reasons	Adjusted	2518	1.918	1.403	2.621	<0.0001	<0.0001
		For other reasons			1.208	0.819	1.782	0.3415	
		For climate and other			0.989	0.494	1.981	0.9755	
Latent class (ref = "Atheist")	Agnostic	For climate reasons	Unadjusted	2551	0.745	0.589	0.943	0.0145	<0.0001
		For other reasons			1.048	0.784	1.402	0.7516	
		For climate and other			0.719	0.432	1.195	0.2034	
	Moderately religious	For climate reasons			0.869	0.682	1.108	0.2577	
		For other reasons			1.121	0.830	1.514	0.4575	
		For climate and other			0.646	0.372	1.121	0.1206	

Exposure	Exposure level	Outcome level (ref = No)	Model	*n*	RRR	LCI	UCI	*p*-value	*p* total
	Highly religious	For climate reasons			1.962	1.386	2.778	0.0001	
		For other reasons			1.804	1.181	2.755	0.0063	
		For climate and other			1.166	0.562	2.419	0.6805	
	Agnostic	For climate reasons	Adjusted	2551	0.808	0.633	1.031	0.0862	0.0038
		For other reasons			1.078	0.800	1.452	0.6226	
		For climate and other			0.775	0.461	1.303	0.3361	
	Moderately religious	For climate reasons			0.949	0.737	1.222	0.6851	
		For other reasons			1.137	0.834	1.549	0.4169	
		For climate and other			0.681	0.387	1.198	0.1824	
	Highly religious	For climate reasons			1.743	1.216	2.499	0.0025	
		For other reasons			1.555	1.006	2.403	0.0471	
		For climate and other			0.964	0.456	2.037	0.9228	
Reduced air travel									
Belief (ref = No)	Not sure	For climate reasons	Unadjusted	2537	0.721	0.552	0.942	0.0166	0.0106
		For other reasons			1.034	0.810	1.320	0.7905	
		For climate and other			1.105	0.567	2.152	0.7700	
	Yes	For climate reasons			0.719	0.561	0.922	0.0093	
		For other reasons			1.131	0.902	1.420	0.2867	
		For climate and other			0.581	0.283	1.191	0.1379	
	Not sure	For climate reasons	Adjusted	2537	0.827	0.624	1.096	0.1861	0.0777
		For other reasons			1.088	0.846	1.399	0.5115	
		For climate and other			1.380	0.689	2.762	0.3630	
	Yes	For climate reasons			0.779	0.598	1.014	0.0634	
		For other reasons			1.140	0.902	1.443	0.2730	
		For climate and other			0.683	0.325	1.435	0.3143	
Identity (combined; ref = None)	Christian	For climate reasons	Unadjusted	2511	0.608	0.487	0.760	<0.0001	<0.0001
		For other reasons			0.927	0.754	1.139	0.4702	
		For climate and other			0.518	0.293	0.915	0.0234	
	Christian	For climate reasons	Adjusted	2511	0.733	0.578	0.930	0.0104	0.0293
		For other reasons			1.005	0.811	1.245	0.9656	
		For climate and other			0.645	0.356	1.170	0.1487	
Identity (separate; ref = None)	C of E	For climate reasons	Unadjusted	2511	0.552	0.435	0.700	<0.0001	0.0001
		For other reasons			0.858	0.691	1.066	0.1670	

Exposure	Exposure level	Outcome level (ref = No)	Model	*n*	RRR	LCI	UCI	*p*-value	*p* total
		For climate and other			0.508	0.277	0.931	0.0284	
	Catholic	For climate reasons			0.836	0.555	1.261	0.3935	
		For other reasons			1.005	0.688	1.469	0.9788	
		For climate and other			0.520	0.152	1.786	0.2992	
	Other	For climate reasons			0.748	0.522	1.072	0.1139	
		For other reasons			1.230	0.901	1.680	0.1923	
		For climate and other			0.572	0.211	1.548	0.2715	
	C of E	For climate reasons	Adjusted	2511	0.693	0.538	0.892	0.0044	0.1238
		For other reasons			0.955	0.763	1.196	0.6870	
		For climate and other			0.677	0.360	1.276	0.2283	
	Catholic	For climate reasons			0.879	0.571	1.356	0.5606	
		For other reasons			1.003	0.679	1.481	0.9884	
		For climate and other			0.532	0.151	1.874	0.3257	
	Other	For climate reasons			0.825	0.565	1.204	0.3182	
		For other reasons			1.254	0.910	1.730	0.1670	
		For climate and other			0.606	0.217	1.690	0.3386	
Attendance (ref = Occasional/None)	Regular	For climate reasons	Unadjusted	2511	1.888	1.424	2.503	<0.0001	0.0002
		For other reasons			1.423	1.092	1.854	0.0090	
		For climate and other			1.394	0.643	3.021	0.4000	
	Regular	For climate reasons	Adjusted	2511	1.462	1.083	1.974	0.0130	0.0972
		For other reasons			1.200	0.913	1.579	0.1913	
		For climate and other			1.026	0.464	2.270	0.9495	
Latent class (ref = "Atheist")	Agnostic	For climate reasons	Unadjusted	2543	0.655	0.500	0.857	0.0021	<0.0001
		For other reasons			1.002	0.793	1.267	0.9836	
		For climate and other			0.759	0.388	1.484	0.4197	
	Moderately religious	For climate reasons			0.607	0.460	0.803	0.0005	
		For other reasons			1.007	0.794	1.277	0.9531	
		For climate and other			0.427	0.188	0.971	0.0422	
	Highly religious	For climate reasons			1.593	1.156	2.196	0.0044	
		For other reasons			1.537	1.130	2.092	0.0062	
		For climate and other			1.084	0.452	2.601	0.8559	
	Agnostic	For climate reasons	Adjusted	2543	0.720	0.543	0.953	0.0219	0.0036
		For other reasons			1.048	0.824	1.332	0.7036	
		For climate and other			0.839	0.420	1.676	0.6186	

**Exposure**	**Exposure level**	**Outcome level (ref = No)**	**Model**	***n***	**RRR**	**LCI**	**UCI**	***p*-value**	***p* total**
	Moderately religious	For climate reasons			0.688	0.514	0.920	0.0118	
		For other reasons			1.050	0.823	1.340	0.6943	
		For climate and other			0.513	0.221	1.191	0.1204	
	Highly religious	For climate reasons			1.394	0.992	1.960	0.0557	
		For other reasons			1.376	1.001	1.891	0.0495	
		For climate and other			0.899	0.365	2.215	0.8176	
Bought or hired an electric or hybrid vehicle									
Belief (ref = No)	Not sure	For climate reasons	Unadjusted	2557	0.726	0.501	1.052	0.0905	0.5300
		For other reasons			1.127	0.623	2.040	0.6920	
		For climate and other			1.216	0.384	3.852	0.7393	
	Yes	For climate reasons			0.961	0.694	1.331	0.8108	
		For other reasons			0.900	0.503	1.612	0.7229	
		For climate and other			0.772	0.235	2.542	0.6707	
	Not sure	For climate reasons	Adjusted	2557	0.770	0.525	1.128	0.1793	0.7387
		For other reasons			1.110	0.608	2.024	0.7343	
		For climate and other			0.977	0.290	3.291	0.9699	
	Yes	For climate reasons			1.010	0.720	1.417	0.9543	
		For other reasons			0.944	0.521	1.712	0.8506	
		For climate and other			0.734	0.213	2.531	0.6248	
Identity (combined; ref = None)	Christian	For climate reasons	Unadjusted	2530	0.880	0.651	1.189	0.4045	0.6492
		For other reasons			1.077	0.639	1.814	0.7805	
		For climate and other			0.627	0.242	1.624	0.3362	
	Christian	For climate reasons	Adjusted	2530	1.021	0.745	1.399	0.8992	0.7447
		For other reasons			1.058	0.620	1.804	0.8365	
		For climate and other			0.567	0.207	1.555	0.2704	
Identity (separate; ref = None)	C of E	For climate reasons	Unadjusted	2530	0.784	0.567	1.084	0.1406	0.3863
		For other reasons			1.034	0.596	1.792	0.9060	
		For climate and other			0.699	0.259	1.886	0.4797	
	Catholic	For climate reasons			0.948	0.542	1.656	0.8502	
		For other reasons			1.329	0.553	3.195	0.5248	
		For climate and other			NA	NA	NA	NA	
	Other	For climate reasons			1.273	0.826	1.963	0.2742	
		For other reasons			1.113	0.500	2.478	0.7923	

Exposure	Exposure level	Outcome level (ref = No)	Model	*n*	RRR	LCI	UCI	*p*-value	*p* total
		For climate and other			0.707	0.146	3.424	0.6666	
	C of E	For climate reasons	Adjusted	2530	0.941	0.670	1.320	0.7237	0.5425
		For other reasons			1.002	0.571	1.761	0.9931	
		For climate and other			0.618	0.217	1.758	0.3667	
	Catholic	For climate reasons			0.954	0.538	1.692	0.8727	
		For other reasons			1.326	0.542	3.243	0.5365	
		For climate and other			NA	NA	NA	NA	
	Other	For climate reasons			1.404	0.894	2.203	0.1405	
		For other reasons			1.138	0.504	2.570	0.7554	
		For climate and other			0.746	0.143	3.884	0.7279	
Attendance (ref = Occasional/None)	Regular	For climate reasons	Unadjusted	2530	1.813	1.294	2.541	0.0005	0.0006
		For other reasons			0.359	0.130	0.991	0.0481	
		For climate and other			0.777	0.178	3.392	0.7372	
	Regular	For climate reasons	Adjusted	2530	1.489	1.047	2.118	0.0268	0.0207
		For other reasons			0.377	0.135	1.052	0.0624	
		For climate and other			0.959	0.207	4.437	0.9578	
Latent class (ref = "Atheist")	Agnostic	For climate reasons	Unadjusted	2563	0.709	0.486	1.033	0.0733	0.0068
		For other reasons			1.078	0.599	1.941	0.8018	
		For climate and other			0.735	0.239	2.258	0.5912	
	Moderately religious	For climate reasons			0.888	0.616	1.279	0.5224	
		For other reasons			1.361	0.769	2.408	0.2898	
		For climate and other			0.490	0.129	1.855	0.2936	
	Highly religious	For climate reasons			1.596	1.078	2.362	0.0195	
		For other reasons			0.346	0.103	1.157	0.0849	
		For climate and other			0.692	0.146	3.277	0.6423	
	Agnostic	For climate reasons	Adjusted	2563	0.751	0.511	1.105	0.1464	0.0657
		For other reasons			1.096	0.605	1.985	0.7633	
		For climate and other			0.656	0.204	2.110	0.4790	
	Moderately religious	For climate reasons			1.005	0.689	1.465	0.9809	
		For other reasons			1.421	0.795	2.540	0.2356	
		For climate and other			0.490	0.126	1.907	0.3034	
	Highly religious	For climate reasons			1.421	0.944	2.137	0.0918	
		For other reasons			0.371	0.110	1.257	0.1114	
		For climate and other			0.758	0.150	3.832	0.7372	

Exposure	Exposure level	Outcome level (ref = No)	Model	*n*	RRR	LCI	UCI	*p*-value	*p* total
Bought foods produced locally									
Belief (ref = No)	Not sure	For climate reasons	Unadjusted	2544	0.880	0.683	1.134	0.3234	0.7143
		For other reasons			0.932	0.706	1.231	0.6197	
		For climate and other			0.842	0.505	1.405	0.5106	
	Yes	For climate reasons			1.063	0.836	1.352	0.6173	
		For other reasons			1.131	0.870	1.471	0.3582	
		For climate and other			0.946	0.585	1.529	0.8212	
	Not sure	For climate reasons	Adjusted	2544	0.962	0.739	1.253	0.7756	0.7958
		For other reasons			0.949	0.714	1.262	0.7207	
		For climate and other			0.925	0.545	1.570	0.7730	
	Yes	For climate reasons			1.157	0.899	1.489	0.2569	
		For other reasons			1.134	0.865	1.487	0.3612	
		For climate and other			1.027	0.624	1.692	0.9159	
Identity (combined; ref = None)	Christian	For climate reasons	Unadjusted	2517	0.870	0.699	1.082	0.2107	0.0066
		For other reasons			0.978	0.769	1.244	0.8547	
		For climate and other			0.496	0.330	0.746	0.0007	
	Christian	For climate reasons	Adjusted	2517	1.007	0.800	1.267	0.9544	0.0509
		For other reasons			1.003	0.782	1.287	0.9816	
		For climate and other			0.567	0.370	0.868	0.0091	
Identity (separate; ref = None)	C of E	For climate reasons	Unadjusted	2517	0.852	0.677	1.072	0.1724	0.0437
		For other reasons			1.000	0.777	1.286	1.0000	
		For climate and other			0.520	0.338	0.802	0.0030	
	Catholic	For climate reasons			0.896	0.604	1.328	0.5847	
		For other reasons			0.944	0.611	1.457	0.7938	
		For climate and other			0.206	0.062	0.688	0.0103	
	Other	For climate reasons			0.930	0.666	1.300	0.6723	
		For other reasons			0.903	0.621	1.312	0.5915	
		For climate and other			0.580	0.298	1.130	0.1094	
	C of E	For climate reasons	Adjusted	2517	1.017	0.798	1.296	0.8911	0.1590
		For other reasons			1.042	0.803	1.353	0.7563	
		For climate and other			0.620	0.394	0.974	0.0382	
	Catholic	For climate reasons			0.930	0.616	1.403	0.7279	
		For other reasons			0.920	0.590	1.434	0.7115	

Exposure	Exposure level	Outcome level (ref = No)	Model	*n*	RRR	LCI	UCI	*p*-value	*p* total
		For climate and other			0.207	0.061	0.700	0.0113	
	Other	For climate reasons			1.009	0.711	1.432	0.9607	
		For other reasons			0.892	0.608	1.309	0.5589	
		For climate and other			0.618	0.310	1.229	0.1700	
Attendance (ref = Occasional/None)	Regular	For climate reasons	Unadjusted	2517	1.295	0.979	1.713	0.0699	0.0884
		For other reasons			0.939	0.683	1.292	0.6997	
		For climate and other			1.243	0.715	2.158	0.4408	
	Regular	For climate reasons	Adjusted	2517	1.084	0.808	1.455	0.5906	0.3331
		For other reasons			0.830	0.597	1.154	0.2676	
		For climate and other			1.012	0.572	1.791	0.9671	
Latent class (ref = "Atheist")	Agnostic	For climate reasons	Unadjusted	2550	0.829	0.648	1.061	0.1357	0.1376
		For other reasons			0.979	0.750	1.277	0.8750	
		For climate and other			0.731	0.444	1.205	0.2193	
	Moderately religious	For climate reasons			1.145	0.886	1.479	0.2997	
		For other reasons			1.178	0.891	1.557	0.2512	
		For climate and other			0.763	0.447	1.303	0.3218	
	Highly religious	For climate reasons			1.261	0.912	1.743	0.1600	
		For other reasons			1.039	0.722	1.496	0.8366	
		For climate and other			1.138	0.615	2.108	0.6803	
	Agnostic	For climate reasons	Adjusted	2550	0.901	0.697	1.164	0.4250	0.2825
		For other reasons			1.009	0.769	1.324	0.9467	
		For climate and other			0.814	0.488	1.359	0.4320	
	Moderately religious	For climate reasons			1.292	0.989	1.687	0.0602	
		For other reasons			1.227	0.922	1.633	0.1608	
		For climate and other			0.875	0.504	1.517	0.6338	
	Highly religious	For climate reasons			1.164	0.828	1.635	0.3815	
		For other reasons			0.964	0.662	1.402	0.8468	
		For climate and other			1.044	0.552	1.973	0.8953	
Recycled or upcycled more									
Belief (ref = No)	Not sure	For climate reasons	Unadjusted	2555	1.106	0.712	1.718	0.6531	0.6584
		For other reasons			1.167	0.724	1.881	0.5270	
		For climate and other			1.312	0.703	2.451	0.3938	
	Yes	For climate reasons			1.095	0.729	1.645	0.6604	

Exposure	Exposure level	Outcome level (ref = No)	Model	*n*	RRR	LCI	UCI	*p*-value	*p* total
		For other reasons			1.111	0.714	1.730	0.6407	
		For climate and other			0.837	0.454	1.542	0.5681	
	Not sure	For climate reasons	Adjusted	2555	1.134	0.722	1.782	0.5856	0.5959
		For other reasons			1.155	0.709	1.883	0.5631	
		For climate and other			1.332	0.701	2.529	0.3812	
	Yes	For climate reasons			1.076	0.706	1.638	0.7340	
		For other reasons			1.043	0.661	1.646	0.8569	
		For climate and other			0.790	0.421	1.484	0.4641	
Identity (combined; ref = None)	Christian	For climate reasons	Unadjusted	2529	1.313	0.916	1.881	0.1377	0.0139
		For other reasons			1.337	0.903	1.980	0.1472	
		For climate and other			0.723	0.434	1.205	0.2135	
	Christian	For climate reasons	Adjusted	2529	1.377	0.947	2.003	0.0940	0.0112
		For other reasons			1.313	0.873	1.974	0.1905	
		For climate and other			0.729	0.428	1.241	0.2444	
Identity (separate; ref = None)	C of E	For climate reasons	Unadjusted	2529	1.379	0.939	2.025	0.1017	0.0433
		For other reasons			1.374	0.904	2.089	0.1374	
		For climate and other			0.763	0.442	1.319	0.3329	
	Catholic	For climate reasons			1.060	0.559	2.011	0.8577	
		For other reasons			0.942	0.463	1.916	0.8696	
		For climate and other			0.246	0.066	0.919	0.0370	
	Other	For climate reasons			1.233	0.684	2.219	0.4860	
		For other reasons			1.490	0.793	2.800	0.2153	
		For climate and other			0.932	0.410	2.121	0.8668	
	C of E	For climate reasons	Adjusted	2529	1.483	0.995	2.212	0.0531	0.0411
		For other reasons			1.378	0.892	2.126	0.1481	
		For climate and other			0.796	0.451	1.406	0.4326	
	Catholic	For climate reasons			1.007	0.520	1.951	0.9828	
		For other reasons			0.880	0.425	1.823	0.7303	
		For climate and other			0.235	0.062	0.896	0.0340	
	Other	For climate reasons			1.270	0.693	2.326	0.4391	
		For other reasons			1.432	0.750	2.736	0.2763	
		For climate and other			0.878	0.377	2.042	0.7625	
Attendance (ref = Occasional/None)	Regular	For climate reasons	Unadjusted	2528	1.018	0.629	1.647	0.9426	0.7251
		For other reasons			0.905	0.535	1.530	0.7097	

Exposure	Exposure level	Outcome level (ref = No)	Model	*n*	RRR	LCI	UCI	*p*-value	*p* total
		For climate and other			0.785	0.374	1.647	0.5222	
	Regular	For climate reasons	Adjusted	2528	0.818	0.496	1.347	0.4295	0.4975
		For other reasons			0.744	0.432	1.280	0.2851	
		For climate and other			0.584	0.273	1.252	0.1670	
Latent class (ref = "Atheist")	Agnostic	For climate reasons	Unadjusted	2561	1.221	0.792	1.885	0.3661	0.6763
		For other reasons			1.367	0.856	2.185	0.1904	
		For climate and other			1.066	0.579	1.965	0.8368	
	Moderately religious	For climate reasons			1.135	0.738	1.747	0.5642	
		For other reasons			1.133	0.708	1.813	0.6026	
		For climate and other			0.692	0.360	1.333	0.2709	
	Highly religious	For climate reasons			1.167	0.671	2.031	0.5845	
		For other reasons			1.187	0.651	2.166	0.5760	
		For climate and other			0.731	0.314	1.703	0.4673	
	Agnostic	For climate reasons	Adjusted	2561	1.269	0.815	1.978	0.2922	0.5857
		For other reasons			1.378	0.855	2.222	0.1884	
		For climate and other			1.107	0.593	2.068	0.7501	
	Moderately religious	For climate reasons			1.174	0.755	1.827	0.4760	
		For other reasons			1.126	0.696	1.821	0.6282	
		For climate and other			0.697	0.357	1.362	0.2909	
	Highly religious	For climate reasons			1.007	0.569	1.783	0.9802	
		For other reasons			1.017	0.548	1.887	0.9581	
		For climate and other			0.586	0.246	1.396	0.2278	
Reduced the amount of plastic used									
Belief (ref = No)	Not sure	For climate reasons	Unadjusted	2562	1.202	0.858	1.684	0.2860	0.1886
		For other reasons			1.233	0.813	1.871	0.3243	
		For climate and other			1.448	0.810	2.588	0.2121	
	Yes	For climate reasons			1.479	1.070	2.044	0.0177	
		For other reasons			1.456	0.979	2.165	0.0635	
		For climate and other			1.149	0.642	2.058	0.6393	
	Not sure	For climate reasons	Adjusted	2562	1.279	0.905	1.806	0.1632	0.1081
		For other reasons			1.333	0.872	2.038	0.1844	
		For climate and other			1.547	0.854	2.803	0.1499	
	Yes	For climate reasons			1.581	1.133	2.208	0.0071	

Exposure	Exposure level	Outcome level (ref = No)	Model	*n*	RRR	LCI	UCI	*p*-value	*p* total
		For other reasons			1.570	1.045	2.358	0.0299	
		For climate and other			1.229	0.675	2.235	0.5003	
Identity (combined; ref = None)	Christian	For climate reasons	Unadjusted	2535	1.075	0.800	1.445	0.6322	0.3264
		For other reasons			1.069	0.745	1.534	0.7163	
		For climate and other			0.723	0.442	1.183	0.1971	
	Christian	For climate reasons	Adjusted	2535	1.196	0.879	1.625	0.2542	0.2560
		For other reasons			1.160	0.799	1.684	0.4366	
		For climate and other			0.808	0.485	1.346	0.4122	
Identity (separate; ref = None)	C of E	For climate reasons	Unadjusted	2535	1.021	0.749	1.392	0.8943	0.4493
		For other reasons			1.060	0.726	1.547	0.7624	
		For climate and other			0.792	0.475	1.322	0.3720	
	Catholic	For climate reasons			1.224	0.690	2.171	0.4892	
		For other reasons			1.142	0.571	2.284	0.7068	
		For climate and other			0.480	0.150	1.540	0.2172	
	Other	For climate reasons			1.251	0.773	2.025	0.3612	
		For other reasons			1.069	0.593	1.926	0.8253	
		For climate and other			0.537	0.213	1.359	0.1893	
	C of E	For climate reasons	Adjusted	2535	1.154	0.837	1.592	0.3822	0.4412
		For other reasons			1.167	0.790	1.725	0.4377	
		For climate and other			0.898	0.528	1.530	0.6933	
	Catholic	For climate reasons			1.243	0.692	2.233	0.4658	
		For other reasons			1.175	0.581	2.379	0.6535	
		For climate and other			0.497	0.153	1.616	0.2450	
	Other	For climate reasons			1.347	0.822	2.207	0.2378	
		For other reasons			1.103	0.605	2.012	0.7490	
		For climate and other			0.577	0.224	1.484	0.2538	
Attendance (ref = Occasional/None)	Regular	For climate reasons	Unadjusted	2535	1.313	0.873	1.976	0.1911	0.1465
		For other reasons			0.970	0.586	1.606	0.9062	
		For climate and other			0.899	0.430	1.879	0.7776	
	Regular	For climate reasons	Adjusted	2535	1.153	0.757	1.755	0.5081	0.2414
		For other reasons			0.872	0.520	1.462	0.6029	
		For climate and other			0.746	0.351	1.588	0.4477	
Latent class (ref = "Atheist")	Agnostic	For climate reasons	Unadjusted	2568	0.984	0.709	1.366	0.9233	0.3123
		For other reasons			1.005	0.672	1.504	0.9813	

Exposure	Exposure level	Outcome level (ref = No)	Model	*n*	RRR	LCI	UCI	*p*-value	*p* total
		For climate and other			0.960	0.550	1.675	0.8857	
	Moderately religious	For climate reasons			1.394	0.971	2.001	0.0715	
		For other reasons			1.295	0.838	2.001	0.2451	
		For climate and other			0.779	0.405	1.496	0.4527	
	Highly religious	For climate reasons			1.369	0.863	2.170	0.1818	
		For other reasons			1.182	0.675	2.069	0.5580	
		For climate and other			0.952	0.429	2.113	0.9034	
	Agnostic	For climate reasons	Adjusted	2568	1.047	0.749	1.465	0.7868	0.2741
		For other reasons			1.070	0.710	1.612	0.7454	
		For climate and other			1.020	0.578	1.799	0.9466	
	Moderately religious	For climate reasons			1.520	1.051	2.200	0.0262	
		For other reasons			1.409	0.904	2.194	0.1295	
		For climate and other			0.851	0.437	1.654	0.6334	
	Highly religious	For climate reasons			1.294	0.805	2.079	0.2866	
		For other reasons			1.132	0.638	2.009	0.6708	
		For climate and other			0.865	0.382	1.958	0.7278	
Chosen sustainably sourced items									
Belief (ref = No)	Not sure	For climate reasons	Unadjusted	2538	0.814	0.644	1.028	0.0842	0.4899
		For other reasons			0.983	0.706	1.369	0.9203	
		For climate and other			0.729	0.397	1.339	0.3080	
	Yes	For climate reasons			0.978	0.785	1.220	0.8446	
		For other reasons			1.037	0.757	1.420	0.8226	
		For climate and other			0.755	0.425	1.340	0.3368	
	Not sure	For climate reasons	Adjusted	2538	0.913	0.714	1.167	0.4684	0.6726
		For other reasons			1.101	0.785	1.545	0.5770	
		For climate and other			0.846	0.453	1.579	0.5984	
	Yes	For climate reasons			1.084	0.859	1.369	0.4964	
		For other reasons			1.134	0.820	1.569	0.4467	
		For climate and other			0.839	0.464	1.517	0.5615	
Identity (combined; ref = None)	Christian	For climate reasons	Unadjusted	2511	0.781	0.638	0.956	0.0165	0.0142
		For other reasons			0.827	0.623	1.098	0.1885	
		For climate and other			0.497	0.304	0.813	0.0054	
	Christian	For climate reasons	Adjusted	2511	0.938	0.757	1.162	0.5576	0.2445

Exposure	Exposure level	Outcome level (ref = No)	Model	*n*	RRR	LCI	UCI	*p*-value	*p* total
		For other reasons			0.935	0.697	1.254	0.6530	
		For climate and other			0.582	0.349	0.971	0.0383	
Identity (separate; ref = None)	C of E	For climate reasons	Unadjusted	2511	0.740	0.599	0.915	0.0053	0.0956
		For other reasons			0.792	0.588	1.067	0.1248	
		For climate and other			0.494	0.293	0.834	0.0083	
	Catholic	For climate reasons			0.988	0.679	1.438	0.9506	
		For other reasons			0.908	0.532	1.552	0.7247	
		For climate and other			0.370	0.109	1.263	0.1126	
	Other	For climate reasons			0.857	0.626	1.174	0.3373	
		For other reasons			0.948	0.613	1.466	0.8101	
		For climate and other			0.586	0.258	1.332	0.2021	
	C of E	For climate reasons	Adjusted	2511	0.917	0.733	1.146	0.4451	0.7399
		For other reasons			0.918	0.675	1.249	0.5859	
		For climate and other			0.600	0.349	1.034	0.0658	
	Catholic	For climate reasons			1.083	0.730	1.606	0.6924	
		For other reasons			0.965	0.557	1.669	0.8973	
		For climate and other			0.397	0.114	1.376	0.1450	
	Other	For climate reasons			0.951	0.684	1.322	0.7646	
		For other reasons			0.998	0.638	1.560	0.9927	
		For climate and other			0.623	0.269	1.441	0.2687	
Attendance (ref = Occasional/None)	Regular	For climate reasons	Unadjusted	2511	1.646	1.253	2.163	0.0003	0.0024
		For other reasons			1.203	0.815	1.775	0.3526	
		For climate and other			1.475	0.746	2.917	0.2641	
	Regular	For climate reasons	Adjusted	2511	1.348	1.012	1.796	0.0413	0.1651
		For other reasons			1.051	0.704	1.569	0.8068	
		For climate and other			1.191	0.590	2.405	0.6256	
Latent class (ref = "Atheist")	Agnostic	For climate reasons	Unadjusted	2544	0.757	0.604	0.949	0.0156	0.0158
		For other reasons			0.992	0.723	1.362	0.9603	
		For climate and other			0.602	0.328	1.106	0.1019	
	Moderately religious	For climate reasons			0.977	0.773	1.234	0.8437	
		For other reasons			1.069	0.768	1.490	0.6919	
		For climate and other			0.586	0.303	1.132	0.1114	
	Highly religious	For climate reasons			1.379	1.011	1.881	0.0426	
		For other reasons			1.257	0.810	1.950	0.3083	

**Exposure**	**Exposure level**	**Outcome level (ref = No)**	**Model**	***n***	**RRR**	**LCI**	**UCI**	***p*-value**	***p* total**
		For climate and other			1.210	0.580	2.526	0.6110	
	Agnostic	For climate reasons	Adjusted	2544	0.839	0.662	1.062	0.1442	0.1818
		For other reasons			1.083	0.784	1.497	0.6289	
		For climate and other			0.678	0.365	1.261	0.2197	
	Moderately religious	For climate reasons			1.123	0.879	1.433	0.3540	
		For other reasons			1.203	0.857	1.690	0.2860	
		For climate and other			0.686	0.350	1.344	0.2722	
	Highly religious	For climate reasons			1.270	0.916	1.759	0.1515	
		For other reasons			1.185	0.753	1.863	0.4629	
		For climate and other			1.085	0.509	2.314	0.8328	
Improved home insulation									
Belief (ref = No)	Not sure	For climate reasons	Unadjusted	2559	0.917	0.700	1.202	0.5317	0.1112
		For other reasons			1.080	0.822	1.418	0.5804	
		For climate and other			1.283	0.640	2.571	0.4827	
	Yes	For climate reasons			1.204	0.941	1.539	0.1401	
		For other reasons			1.342	1.043	1.726	0.0221	
		For climate and other			1.082	0.544	2.151	0.8226	
	Not sure	For climate reasons	Adjusted	2559	0.955	0.726	1.257	0.7430	0.1320
		For other reasons			1.074	0.815	1.416	0.6125	
		For climate and other			1.332	0.656	2.702	0.4274	
	Yes	For climate reasons			1.246	0.968	1.605	0.0875	
		For other reasons			1.327	1.026	1.718	0.0314	
		For climate and other			1.122	0.556	2.266	0.7471	
Identity (combined; ref = None)	Christian	For climate reasons	Unadjusted	2532	1.018	0.813	1.274	0.8776	0.0512
		For other reasons			1.162	0.924	1.462	0.1979	
		For climate and other			0.515	0.297	0.891	0.0176	
	Christian	For climate reasons	Adjusted	2532	1.085	0.860	1.369	0.4899	0.0763
		For other reasons			1.129	0.891	1.429	0.3149	
		For climate and other			0.521	0.296	0.917	0.0237	
Identity (separate; ref = None)	C of E	For climate reasons	Unadjusted	2532	0.956	0.753	1.213	0.7100	0.0432
		For other reasons			1.156	0.909	1.469	0.2372	
		For climate and other			0.512	0.284	0.924	0.0262	
	Catholic	For climate reasons			1.528	1.042	2.241	0.0300	

**Exposure**	**Exposure level**	**Outcome level (ref = No)**	**Model**	***n***	**RRR**	**LCI**	**UCI**	***p*-value**	***p* total**
		For other reasons			0.963	0.613	1.512	0.8699	
		For climate and other			0.522	0.154	1.765	0.2953	
	Other	For climate reasons			0.989	0.693	1.412	0.9529	
		For other reasons			1.312	0.932	1.848	0.1196	
		For climate and other			0.523	0.196	1.392	0.1940	
	C of E	For climate reasons	Adjusted	2532	1.028	0.804	1.315	0.8243	0.1336
		For other reasons			1.118	0.873	1.431	0.3760	
		For climate and other			0.528	0.287	0.971	0.0398	
	Catholic	For climate reasons			1.548	1.046	2.289	0.0287	
		For other reasons			0.985	0.623	1.557	0.9487	
		For climate and other			0.499	0.145	1.713	0.2693	
	Other	For climate reasons			1.043	0.725	1.500	0.8224	
		For other reasons			1.255	0.885	1.781	0.2027	
		For climate and other			0.505	0.187	1.367	0.1789	
Attendance (ref = Occasional/None)	Regular	For climate reasons	Unadjusted	2532	1.289	0.975	1.703	0.0744	0.3553
		For other reasons			1.046	0.780	1.402	0.7631	
		For climate and other			0.928	0.414	2.080	0.8568	
	Regular	For climate reasons	Adjusted	2532	1.224	0.917	1.632	0.1702	0.5295
		For other reasons			1.093	0.808	1.477	0.5639	
		For climate and other			0.849	0.373	1.934	0.6975	
Latent class (ref = "Atheist")	Agnostic	For climate reasons	Unadjusted	2565	0.848	0.650	1.105	0.2225	0.1410
		For other reasons			1.247	0.961	1.620	0.0973	
		For climate and other			0.815	0.409	1.621	0.5597	
	Moderately religious	For climate reasons			1.129	0.871	1.464	0.3599	
		For other reasons			1.328	1.016	1.737	0.0380	
		For climate and other			0.809	0.393	1.664	0.5646	
	Highly religious	For climate reasons			1.263	0.913	1.746	0.1578	
		For other reasons			1.413	1.011	1.976	0.0429	
		For climate and other			1.156	0.503	2.657	0.7328	
	Agnostic	For climate reasons	Adjusted	2565	0.869	0.664	1.137	0.3064	0.1908
		For other reasons			1.246	0.956	1.624	0.1033	
		For climate and other			0.814	0.405	1.633	0.5617	
	Moderately religious	For climate reasons			1.171	0.898	1.527	0.2440	
		For other reasons			1.296	0.986	1.703	0.0628	

Exposure	Exposure level	Outcome level (ref = No)	Model	*n*	RRR	LCI	UCI	*p*-value	*p* total
		For climate and other			0.851	0.409	1.769	0.6651	
	Highly religious	For climate reasons			1.238	0.887	1.727	0.2087	
		For other reasons			1.476	1.047	2.082	0.0264	
		For climate and other			1.080	0.461	2.528	0.8594	
Installed solar panels									
Belief (ref = No)	Not sure	For climate reasons	Unadjusted	2549	0.671	0.412	1.093	0.1094	0.0620
		For other reasons			0.914	0.498	1.678	0.7725	
		For climate and other			0.873	0.280	2.720	0.8145	
	Yes	For climate reasons			1.326	0.891	1.972	0.1644	
		For other reasons			1.136	0.657	1.964	0.6475	
		For climate and other			0.552	0.168	1.818	0.3287	
	Not sure	For climate reasons	Adjusted	2549	0.721	0.437	1.189	0.2002	0.0554
		For other reasons			0.911	0.491	1.692	0.7680	
		For climate and other			0.875	0.273	2.802	0.8219	
	Yes	For climate reasons			1.456	0.963	2.202	0.0747	
		For other reasons			1.165	0.664	2.045	0.5945	
		For climate and other			0.623	0.183	2.122	0.4488	
Identity (combined; ref = None)	Christian	For climate reasons	Unadjusted	2522	0.930	0.646	1.339	0.6962	0.4206
		For other reasons			1.089	0.655	1.811	0.7428	
		For climate and other			0.451	0.173	1.174	0.1030	
	Christian	For climate reasons	Adjusted	2522	1.107	0.756	1.622	0.6018	0.5386
		For other reasons			1.150	0.680	1.942	0.6026	
		For climate and other			0.527	0.195	1.419	0.2048	
Identity (separate; ref = None)	C of E	For climate reasons	Unadjusted	2522	0.766	0.515	1.141	0.1895	0.0847
		For other reasons			0.958	0.556	1.650	0.8768	
		For climate and other			0.544	0.203	1.455	0.2251	
	Catholic	For climate reasons			1.163	0.612	2.208	0.6456	
		For other reasons			1.498	0.652	3.442	0.3411	
		For climate and other			NA	NA	NA	NA	
	Other	For climate reasons			1.536	0.931	2.532	0.0928	
		For other reasons			1.429	0.693	2.947	0.3331	
		For climate and other			0.313	0.039	2.512	0.2742	
	C of E	For climate reasons	Adjusted	2522	0.933	0.616	1.413	0.7434	0.1721

Exposure	Exposure level	Outcome level (ref = No)	Model	*n*	RRR	LCI	UCI	*p*-value	*p* total
		For other reasons			1.016	0.580	1.779	0.9568	
		For climate and other			0.652	0.235	1.810	0.4119	
	Catholic	For climate reasons			1.241	0.640	2.404	0.5225	
		For other reasons			1.538	0.658	3.595	0.3200	
		For climate and other			NA	NA	NA	NA	
	Other	For climate reasons			1.769	1.048	2.984	0.0327	
		For other reasons			1.490	0.708	3.135	0.2930	
		For climate and other			0.371	0.044	3.097	0.3597	
Attendance (ref = Occasional/None)	Regular	For climate reasons	Unadjusted	2522	2.504	1.712	3.663	<0.0001	0.0002
		For other reasons			1.202	0.641	2.252	0.5663	
		For climate and other			0.859	0.196	3.764	0.8400	
	Regular	For climate reasons	Adjusted	2522	2.234	1.494	3.339	0.0001	0.0026
		For other reasons			1.094	0.575	2.085	0.7839	
		For climate and other			0.844	0.185	3.854	0.8266	
Latent class (ref = "Atheist")	Agnostic	For climate reasons	Unadjusted	2555	0.664	0.409	1.075	0.0959	0.0006
		For other reasons			0.943	0.519	1.714	0.8484	
		For climate and other			0.354	0.097	1.292	0.1159	
	Moderately religious	For climate reasons			0.952	0.608	1.491	0.8309	
		For other reasons			1.201	0.675	2.137	0.5333	
		For climate and other			0.262	0.057	1.198	0.0841	
	Highly religious	For climate reasons			2.290	1.464	3.582	0.0003	
		For other reasons			1.272	0.618	2.621	0.5136	
		For climate and other			0.578	0.126	2.658	0.4817	
	Agnostic	For climate reasons	Adjusted	2555	0.707	0.432	1.157	0.1681	0.0036
		For other reasons			0.938	0.511	1.720	0.8355	
		For climate and other			0.363	0.098	1.345	0.1293	
	Moderately religious	For climate reasons			1.064	0.670	1.689	0.7928	
		For other reasons			1.288	0.713	2.327	0.4016	
		For climate and other			0.312	0.066	1.466	0.1401	
	Highly religious	For climate reasons			2.267	1.418	3.627	0.0006	
		For other reasons			1.258	0.599	2.638	0.5444	
		For climate and other			0.572	0.120	2.732	0.4838	
Started growing vegetables									

**Exposure**	**Exposure level**	**Outcome level (ref = No)**	**Model**	***n***	**RRR**	**LCI**	**UCI**	***p*-value**	***p* total**
Belief (ref = No)	Not sure	For climate reasons	Unadjusted	2558	0.711	0.521	0.971	0.0322	0.1085
		For other reasons			0.761	0.600	0.964	0.0239	
		For climate and other			0.624	0.289	1.349	0.2309	
	Yes	For climate reasons			0.874	0.659	1.158	0.3475	
		For other reasons			0.797	0.639	0.994	0.0443	
		For climate and other			0.594	0.287	1.229	0.1602	
	Not sure	For climate reasons	Adjusted	2558	0.734	0.534	1.010	0.0578	0.1167
		For other reasons			0.761	0.596	0.971	0.0282	
		For climate and other			0.648	0.293	1.432	0.2836	
	Yes	For climate reasons			0.860	0.643	1.152	0.3119	
		For other reasons			0.763	0.606	0.960	0.0208	
		For climate and other			0.554	0.261	1.173	0.1228	
Identity (combined; ref = None)	Christian	For climate reasons	Unadjusted	2531	0.825	0.638	1.066	0.1404	0.1994
		For other reasons			0.826	0.676	1.008	0.0602	
		For climate and other			0.827	0.426	1.606	0.5744	
	Christian	For climate reasons	Adjusted	2531	0.865	0.662	1.129	0.2845	0.4505
		For other reasons			0.857	0.695	1.055	0.1456	
		For climate and other			0.875	0.438	1.748	0.7054	
Identity (separate; ref = None)	C of E	For climate reasons	Unadjusted	2531	0.762	0.580	1.002	0.0517	0.2704
		For other reasons			0.819	0.664	1.011	0.0629	
		For climate and other			0.855	0.426	1.714	0.6584	
	Catholic	For climate reasons			0.943	0.596	1.494	0.8039	
		For other reasons			0.726	0.494	1.067	0.1032	
		For climate and other			0.265	0.034	2.049	0.2033	
	Other	For climate reasons			1.039	0.704	1.536	0.8460	
		For other reasons			0.928	0.678	1.270	0.6419	
		For climate and other			1.105	0.413	2.957	0.8418	
	C of E	For climate reasons	Adjusted	2531	0.816	0.614	1.083	0.1595	0.5026
		For other reasons			0.872	0.700	1.086	0.2221	
		For climate and other			0.947	0.458	1.956	0.8830	
	Catholic	For climate reasons			0.952	0.594	1.524	0.8372	
		For other reasons			0.690	0.465	1.024	0.0655	
		For climate and other			0.270	0.034	2.116	0.2127	
	Other	For climate reasons			1.015	0.679	1.517	0.9413	

**Exposure**	**Exposure level**	**Outcome level (ref = No)**	**Model**	***n***	**RRR**	**LCI**	**UCI**	***p*-value**	***p* total**
		For other reasons			0.912	0.660	1.261	0.5783	
		For climate and other			1.003	0.365	2.759	0.9953	
Attendance (ref = Occasional/None)	Regular	For climate reasons	Unadjusted	2531	1.065	0.760	1.492	0.7140	0.7710
		For other reasons			1.135	0.878	1.467	0.3331	
		For climate and other			1.252	0.549	2.856	0.5934	
	Regular	For climate reasons	Adjusted	2531	0.911	0.643	1.290	0.5987	0.9445
		For other reasons			0.942	0.721	1.231	0.6624	
		For climate and other			0.938	0.403	2.185	0.8825	
Latent class (ref = "Atheist")	Agnostic	For climate reasons	Unadjusted	2564	0.569	0.415	0.782	0.0005	0.0129
		For other reasons			0.895	0.712	1.126	0.3447	
		For climate and other			0.601	0.275	1.316	0.2030	
	Moderately religious	For climate reasons			0.892	0.666	1.195	0.4455	
		For other reasons			0.791	0.621	1.007	0.0570	
		For climate and other			0.466	0.193	1.127	0.0903	
	Highly religious	For climate reasons			0.915	0.628	1.333	0.6422	
		For other reasons			0.989	0.734	1.332	0.9414	
		For climate and other			1.028	0.422	2.503	0.9512	
	Agnostic	For climate reasons	Adjusted	2564	0.597	0.432	0.824	0.0017	0.0448
		For other reasons			0.911	0.720	1.152	0.4356	
		For climate and other			0.636	0.285	1.418	0.2689	
	Moderately religious	For climate reasons			0.910	0.674	1.229	0.5391	
		For other reasons			0.797	0.622	1.022	0.0738	
		For climate and other			0.463	0.188	1.139	0.0937	
	Highly religious	For climate reasons			0.807	0.547	1.190	0.2798	
		For other reasons			0.852	0.626	1.160	0.3104	
		For climate and other			0.785	0.314	1.960	0.6040	
Planted trees									
Belief (ref = No)	Not sure	For climate reasons	Unadjusted	2552	0.875	0.661	1.157	0.3475	0.7177
		For other reasons			0.891	0.667	1.190	0.4361	
		For climate and other			1.242	0.526	2.932	0.6212	
	Yes	For climate reasons			0.797	0.612	1.037	0.0916	
		For other reasons			0.949	0.727	1.238	0.6975	
		For climate and other			1.038	0.451	2.392	0.9298	

**Exposure**	**Exposure level**	**Outcome level (ref = No)**	**Model**	***n***	**RRR**	**LCI**	**UCI**	***p*-value**	***p* total**
	Not sure	For climate reasons	Adjusted	2552	0.920	0.691	1.225	0.5672	0.8449
		For other reasons			0.956	0.711	1.287	0.7689	
		For climate and other			1.412	0.586	3.402	0.4423	
	Yes	For climate reasons			0.832	0.634	1.093	0.1870	
		For other reasons			1.004	0.762	1.324	0.9747	
		For climate and other			1.213	0.514	2.862	0.6598	
Identity (combined; ref = None)	Christian	For climate reasons	Unadjusted	2526	0.714	0.565	0.901	0.0045	0.0167
		For other reasons			0.935	0.733	1.192	0.5880	
		For climate and other			0.551	0.284	1.072	0.0794	
	Christian	For climate reasons	Adjusted	2526	0.785	0.617	1.000	0.0501	0.1037
		For other reasons			1.076	0.835	1.387	0.5695	
		For climate and other			0.638	0.320	1.274	0.2030	
Identity (separate; ref = None)	C of E	For climate reasons	Unadjusted	2526	0.746	0.583	0.954	0.0194	0.0521
		For other reasons			1.016	0.789	1.309	0.9020	
		For climate and other			0.562	0.275	1.147	0.1132	
	Catholic	For climate reasons			0.663	0.421	1.044	0.0759	
		For other reasons			0.595	0.357	0.993	0.0470	
		For climate and other			0.448	0.101	1.984	0.2905	
	Other	For climate reasons			0.614	0.416	0.908	0.0145	
		For other reasons			0.821	0.558	1.209	0.3181	
		For climate and other			0.576	0.189	1.755	0.3314	
	C of E	For climate reasons	Adjusted	2526	0.838	0.649	1.081	0.1741	0.0711
		For other reasons			1.206	0.925	1.572	0.1670	
		For climate and other			0.656	0.313	1.374	0.2636	
	Catholic	For climate reasons			0.695	0.437	1.105	0.1242	
		For other reasons			0.651	0.386	1.098	0.1076	
		For climate and other			0.543	0.120	2.456	0.4275	
	Other	For climate reasons			0.641	0.430	0.957	0.0295	
		For other reasons			0.872	0.586	1.298	0.4994	
		For climate and other			0.628	0.201	1.962	0.4231	
Attendance (ref = Occasional/None)	Regular	For climate reasons	Unadjusted	2525	1.027	0.750	1.406	0.8691	0.2746
		For other reasons			1.194	0.882	1.616	0.2515	
		For climate and other			2.003	0.933	4.299	0.0745	
	Regular	For climate reasons	Adjusted	2525	0.946	0.683	1.310	0.7381	0.4011

Exposure	Exposure level	Outcome level (ref = No)	Model	*n*	RRR	LCI	UCI	*p*-value	*p* total
		For other reasons			1.051	0.768	1.439	0.7547	
		For climate and other			1.998	0.904	4.413	0.0870	
Latent class (ref = "Atheist")	Agnostic	For climate reasons	Unadjusted	2558	0.735	0.557	0.971	0.0301	0.0333
		For other reasons			0.971	0.734	1.284	0.8362	
		For climate and other			0.334	0.122	0.912	0.0324	
	Moderately religious	For climate reasons			0.775	0.585	1.026	0.0751	
		For other reasons			0.951	0.713	1.267	0.7298	
		For climate and other			0.363	0.133	0.991	0.0479	
	Highly religious	For climate reasons			0.835	0.584	1.194	0.3236	
		For other reasons			1.055	0.738	1.510	0.7681	
		For climate and other			1.564	0.705	3.470	0.2715	
	Agnostic	For climate reasons	Adjusted	2558	0.779	0.587	1.035	0.0848	0.0933
		For other reasons			1.042	0.782	1.387	0.7810	
		For climate and other			0.372	0.134	1.028	0.0566	
	Moderately religious	For climate reasons			0.816	0.612	1.090	0.1684	
		For other reasons			1.038	0.772	1.396	0.8026	
		For climate and other			0.406	0.147	1.126	0.0832	
	Highly religious	For climate reasons			0.807	0.558	1.167	0.2545	
		For other reasons			0.994	0.687	1.439	0.9758	
		For climate and other			1.647	0.719	3.769	0.2378	
Avoided organisations that support fossil fuels									
Belief (ref = No)	Not sure	For climate reasons	Unadjusted	2534	0.561	0.422	0.744	0.0001	0.0001
		For other reasons			0.638	0.296	1.375	0.2513	
		For climate and other			0.299	0.079	1.133	0.0757	
	Yes	For climate reasons			0.634	0.491	0.820	0.0005	
		For other reasons			1.329	0.715	2.469	0.3689	
		For climate and other			0.226	0.060	0.857	0.0288	
	Not sure	For climate reasons	Adjusted	2534	0.621	0.463	0.833	0.0015	0.0019
		For other reasons			0.651	0.297	1.424	0.2822	
		For climate and other			0.322	0.082	1.262	0.1040	
	Yes	For climate reasons			0.659	0.504	0.863	0.0024	
		For other reasons			1.223	0.643	2.324	0.5396	
		For climate and other			0.234	0.058	0.950	0.0422	

Exposure	Exposure level	Outcome level (ref = No)	Model	*n*	RRR	LCI	UCI	*p*-value	*p* total
Identity (combined; ref = None)	Christian	For climate reasons	Unadjusted	2508	0.543	0.432	0.682	<0.0001	<0.0001
		For other reasons			0.999	0.559	1.785	0.9978	
		For climate and other			0.363	0.127	1.041	0.0593	
	Christian	For climate reasons	Adjusted	2508	0.607	0.477	0.773	<0.0001	0.0005
		For other reasons			1.011	0.551	1.854	0.9720	
		For climate and other			0.448	0.147	1.359	0.1561	
Identity (separate; ref = None)	C of E	For climate reasons	Unadjusted	2508	0.475	0.370	0.610	<0.0001	<0.0001
		For other reasons			0.794	0.422	1.492	0.4730	
		For climate and other			0.419	0.140	1.252	0.1192	
	Catholic	For climate reasons			0.718	0.466	1.104	0.1314	
		For other reasons			0.897	0.296	2.723	0.8479	
		For climate and other			NA	NA	NA	NA	
	Other	For climate reasons			0.751	0.524	1.075	0.1178	
		For other reasons			2.053	0.986	4.274	0.0546	
		For climate and other			0.335	0.041	2.739	0.3078	
	C of E	For climate reasons	Adjusted	2508	0.551	0.424	0.716	<0.0001	0.0006
		For other reasons			0.836	0.434	1.610	0.5923	
		For climate and other			0.567	0.179	1.794	0.3345	
	Catholic	For climate reasons			0.712	0.455	1.112	0.1351	
		For other reasons			0.822	0.262	2.580	0.7376	
		For climate and other			NA	NA	NA	NA	
	Other	For climate reasons			0.782	0.538	1.137	0.1975	
		For other reasons			1.968	0.907	4.267	0.0866	
		For climate and other			0.331	0.038	2.875	0.3164	
Attendance (ref = Occasional/None)	Regular	For climate reasons	Unadjusted	2507	1.457	1.088	1.951	0.0115	0.0803
		For other reasons			1.423	0.732	2.767	0.2987	
		For climate and other			1.079	0.240	4.843	0.9211	
	Regular	For climate reasons	Adjusted	2507	1.185	0.873	1.609	0.2757	0.6615
		For other reasons			1.224	0.613	2.443	0.5675	
		For climate and other			0.733	0.153	3.507	0.6978	
Latent class (ref = "Atheist")	Agnostic	For climate reasons	Unadjusted	2540	0.559	0.419	0.747	0.0001	<0.0001
		For other reasons			1.044	0.533	2.043	0.9010	
		For climate and other			0.552	0.165	1.843	0.3344	
		For climate reasons			0.586	0.437	0.785	0.0003	

Exposure	Exposure level	Outcome level (ref = No)	Model	*n*	RRR	LCI	UCI	*p*-value	*p* total
	Moderately religious	For other reasons			1.135	0.579	2.223	0.7121	
		For climate and other			NA	NA	NA	NA	
	Highly religious	For climate reasons			1.056	0.760	1.468	0.7463	
		For other reasons			1.385	0.614	3.124	0.4328	
		For climate and other			0.692	0.146	3.282	0.6432	
	Agnostic	For climate reasons	Adjusted	2540	0.602	0.447	0.811	0.0008	0.0060
		For other reasons			1.041	0.524	2.066	0.9084	
		For climate and other			0.611	0.176	2.124	0.4382	
	Moderately religious	For climate reasons			0.635	0.469	0.860	0.0033	
		For other reasons			1.075	0.538	2.147	0.8378	
		For climate and other			NA	NA	NA	NA	
	Highly religious	For climate reasons			0.903	0.640	1.275	0.5614	
		For other reasons			1.103	0.476	2.559	0.8188	
		For climate and other			0.486	0.094	2.527	0.3912	
Taken action to eat less or no meat and/or dairy									
Belief (ref = No)	Not sure	For climate reasons	Unadjusted	2405	0.728	0.546	0.971	0.0307	0.0916
		For other reasons			0.980	0.754	1.274	0.8808	
		For climate and other			0.840	0.588	1.200	0.3378	
	Yes	For climate reasons			0.668	0.510	0.876	0.0035	
		For other reasons			1.003	0.786	1.281	0.9787	
		For climate and other			0.804	0.576	1.124	0.2024	
	Not sure	For climate reasons	Adjusted	2405	0.809	0.598	1.095	0.1697	0.2391
		For other reasons			1.033	0.788	1.354	0.8140	
		For climate and other			0.934	0.644	1.354	0.7188	
	Yes	For climate reasons			0.693	0.520	0.922	0.0120	
		For other reasons			0.995	0.772	1.283	0.9677	
		For climate and other			0.815	0.574	1.158	0.2539	
Identity (combined; ref = None)	Christian	For climate reasons	Unadjusted	2378	0.526	0.412	0.672	<0.0001	<0.0001
		For other reasons			0.779	0.622	0.975	0.0291	
		For climate and other			0.565	0.420	0.761	0.0002	
	Christian	For climate reasons	Adjusted	2378	0.596	0.460	0.772	0.0001	0.0005
		For other reasons			0.817	0.647	1.033	0.0916	
		For climate and other			0.638	0.467	0.872	0.0049	

Exposure	Exposure level	Outcome level (ref = No)	Model	*n*	RRR	LCI	UCI	*p*-value	*p* total
Identity (separate; ref = None)	C of E	For climate reasons	Unadjusted	2378	0.476	0.367	0.617	<0.0001	<0.0001
		For other reasons			0.734	0.580	0.929	0.0100	
		For climate and other			0.525	0.383	0.719	0.0001	
	Catholic	For climate reasons			1.067	0.692	1.646	0.7688	
		For other reasons			1.210	0.807	1.813	0.3564	
		For climate and other			0.754	0.422	1.348	0.3416	
	Other	For climate reasons			0.497	0.335	0.739	0.0005	
		For other reasons			0.775	0.552	1.087	0.1399	
		For climate and other			0.659	0.418	1.039	0.0727	
	C of E	For climate reasons	Adjusted	2378	0.559	0.425	0.736	<0.0001	0.0007
		For other reasons			0.791	0.619	1.010	0.0605	
		For climate and other			0.621	0.446	0.864	0.0048	
	Catholic	For climate reasons			1.092	0.691	1.725	0.7073	
		For other reasons			1.201	0.789	1.827	0.3929	
		For climate and other			0.783	0.429	1.431	0.4271	
	Other	For climate reasons			0.513	0.339	0.776	0.0016	
		For other reasons			0.754	0.531	1.071	0.1151	
		For climate and other			0.651	0.405	1.047	0.0764	
Attendance (ref = Occasional/None)	Regular	For climate reasons	Unadjusted	2382	1.856	1.373	2.509	0.0001	0.0001
		For other reasons			1.189	0.889	1.591	0.2441	
		For climate and other			1.831	1.275	2.628	0.0010	
	Regular	For climate reasons	Adjusted	2382	1.399	1.016	1.927	0.0398	0.0574
		For other reasons			0.952	0.703	1.288	0.7497	
		For climate and other			1.350	0.924	1.972	0.1213	
Latent class (ref = "Atheist")	Agnostic	For climate reasons	Unadjusted	2411	0.620	0.465	0.826	0.0011	<0.0001
		For other reasons			0.985	0.767	1.264	0.9030	
		For climate and other			0.749	0.527	1.065	0.1079	
	Moderately religious	For climate reasons			0.585	0.434	0.789	0.0004	
		For other reasons			1.023	0.794	1.318	0.8615	
		For climate and other			0.669	0.462	0.970	0.0337	
	Highly religious	For climate reasons			1.413	0.999	2.000	0.0509	
		For other reasons			1.332	0.953	1.862	0.0934	
		For climate and other			1.609	1.060	2.440	0.0254	
	Agnostic	For climate reasons	Adjusted	2411	0.679	0.504	0.917	0.0114	0.0068

**Exposure**	**Exposure level**	**Outcome level (ref = No)**	**Model**	***n***	**RRR**	**LCI**	**UCI**	***p*-value**	***p* total**
		For other reasons			1.029	0.796	1.330	0.8288	
		For climate and other			0.819	0.570	1.178	0.2818	
	Moderately religious	For climate reasons			0.651	0.477	0.888	0.0068	
		For other reasons			1.065	0.820	1.384	0.6363	
		For climate and other			0.721	0.492	1.058	0.0946	
	Highly religious	For climate reasons			1.190	0.824	1.720	0.3531	
		For other reasons			1.138	0.804	1.611	0.4650	
		For climate and other			1.303	0.842	2.016	0.2355	

Table S20: Descriptive statistics of sociodemographic characteristics and other confounders for G0 partners. Columns display descriptive statistics for both the full sample (n = 10,916) and the complete-case sample with fully-observed data on all confounders, any RSBB exposure data and any climate outcome data (*n* = 1,126). Note that the percentages of missing data are calculated separately from the observed data.

**Variable**	**Full sample – N (%) or mean (SD)**	**Complete-case sample – N (%) or mean (SD)**
Age at birth of study child (years)	30.7 (5.77)	32.4 (4.93)
Missing	*101 (0.9%)*	*NA*
Age at completion of climate questions (years)	62.4 (5.04)	62.4 (4.94)
Missing	*8.997 (82.4%)*	*NA*
Ethnicity		
White	9.426 (97.1%)	1,117 (99.4%)
Other than White	282 (2.9%)	7 (0.6%)
Missing	*1,208 (11.1%)*	*NA*
Marital status		
Married	6,450 (82.7%)	1,033 (91.7%)
Never married	1,021 (13.1%)	63 (5.6%)
Separated/Divorced/Widowed	328 (4.2%)	30 (2.7%)
Missing	*3,117 (28.6%)*	*NA*
Urban vs rural location		
Urban	8,057 (81.8%)	876 (77.8%)
Rural	1,796 (18.2%)	250 (22.2%)
Missing	*1,063 (9.7%)*	*NA*
Highest educational qualification		
CSE/None	2,201 (23.0%)	87 (7.7%)
Vocational	776 (8.1%)	47 (4.2%)
O-level	2,083 (21.7%)	223 (19.8%)
A-level	2,612 (27.2%)	349 (31.0%)
Degree	1,916 (20.0%)	420 (37.3%)
Missing	*1,328 (12.2%)*	*NA*
Occupational social class		
I	1,062 (11.8%)	237 (21.0%)
II	3,157 (35.2%)	491 (43.6%)
III (non-manual)	992 (11.0%)	148 (13.1%)
III (manual)	2,685 (29.9%)	185 (16.4%)
IV/V	1,078 (12.0%)	65 (5.8%)
Missing	*1,942 (17.8%)*	*NA*
Household income (per week)		
< £100	450 (6.2%)	10 (0.9%)
£100 - £199	1,200 (16.6%)	81 (7.2%)
£200 - £299	2,128 (29.4%)	274 (24.3%)

£300 - £399	1,604 (22.2%)	325 (28.9%)
≥ £400	1,860 (25.7%)	436 (38.7%)
Missing	*3,674 (33.7%)*	*NA*
Area-level index of multiple deprivation during pregnancy (quintiles)		
1 (Least deprived)	3,222 (33.2%)	523 (46.4%)
2	2,300 (23.7%)	301 (26.7%)
3	1,712 (17.7%)	165 (14.6%)
4	1,449 (15.0%)	114 (10.1%)
5 (Most deprived)	1,006 (10.4%)	23 (2.0%)
Missing	*1,227 (11.2%)*	*NA*
Home ownership status		
Owned/Mortgaged	7,817 (76.7%)	1,043 (92.6%)
Private rented	681 (6.7%)	31 (2.8%)
Council/Housing association	1,378 (13.5%)	28 (2.5%)
Other	316 (3.1%)	24 (2.1%)
Missing	*724 (6.6%)*	*NA*

Table S21: Results of the partners ordinal regression models with ‘belief that the climate is changing’ as the outcome for four religious exposures (belief, identity [both Christian denominations combined together and separate], attendance, and latent classes). Odds ratios above 1 indicate an increased belief in climate change. The ‘brant’ column shows the *p*-value of the brant test for said variable. The ‘*p* total’ column is the *p*-value for the overall association between the exposure and outcome (if three or more exposure levels). OR = Odds ratio; LCI = Lower 95% confidence interval; UCI = Upper 95% confidence interval.

**Exposure**	**Exposure level**	**Model**	***n***	**OR**	**LCI**	**UCI**	***p*-value**	**brant**	***p* total**
Belief (ref = No)	Not sure	Unadjusted	1115	0.672	0.478	0.944	0.0220	0.8146	0.0726
	Yes			0.837	0.597	1.174	0.3016	0.8518	
	Not sure	Adjusted	1115	0.744	0.521	1.064	0.1051	0.9998	0.2663
	Yes			0.867	0.605	1.242	0.4352	0.9996	
Identity (combined; ref = None)	Christian	Unadjusted	1104	0.535	0.396	0.723	<0.0001	0.1711	NA
	Christian	Adjusted	1104	0.592	0.431	0.813	0.0012	0.4488	NA
Identity (separate; ref = None)	C of E	Unadjusted	1104	0.475	0.347	0.651	<0.0001	0.1996	0.0001
	Catholic			0.800	0.408	1.568	0.5155	0.9535	
	Other			0.777	0.451	1.340	0.3648	0.7373	
	C of E	Adjusted	1104	0.530	0.381	0.739	0.0002	0.3163	0.0017
	Catholic			0.908	0.446	1.849	0.7911	1.0000	
	Other			0.826	0.467	1.460	0.5108	1.0000	
Attendance (ref = Occasional/None)	Regular	Unadjusted	1117	2.791	1.577	4.940	0.0004	0.5438	NA
	Regular	Adjusted	1117	2.511	1.392	4.529	0.0022	0.6629	NA
Latent class (ref = “Atheist”)	Agnostic	Unadjusted	1125	0.669	0.478	0.934	0.0183	0.5654	0.0010
	Moderately religious			0.698	0.467	1.045	0.0805	0.9362	
	Highly religious			2.126	1.157	3.906	0.0151	0.8762	
	Agnostic	Adjusted	1125	0.742	0.523	1.054	0.0959	0.5654	0.0111
	Moderately religious			0.764	0.500	1.165	0.2111	0.9362	
	Highly religious			2.100	1.115	3.953	0.0215	0.8762	

Table S22: Results of the partners ordinal regression models with 'concerned about the impact of climate change' as the outcome for four religious exposures (belief, identity [both Christian denominations combined together and separate], attendance, and latent classes). Odds ratios above 1 indicate an increased concern regarding climate change. The 'brant' column shows the *p*-value of the brant test for said variable. The '*p* total' column is the *p*-value for the overall association between the exposure and outcome (if three or more exposure levels). OR = Odds ratio; LCI = Lower 95% confidence interval; UCI = Upper 95% confidence interval.

**Exposure**	**Exposure level**	**Model**	***n***	**OR**	**LCI**	**UCI**	***p*-value**	**brant**	***p* total**
Belief (ref = No)	Not sure	Unadjusted	1110	0.747	0.566	0.987	0.0402	0.2913	0.0635
	Yes			0.779	0.595	1.019	0.0681	0.8855	
	Not sure	Adjusted	1110	0.809	0.607	1.078	0.1485	0.4264	0.2208
	Yes			0.815	0.615	1.080	0.1536	0.9607	
Identity (combined; ref = None)	Christian	Unadjusted	1100	0.577	0.458	0.728	<0.0001	0.3346	NA
	Christian	Adjusted	1100	0.653	0.514	0.831	0.0005	0.2182	NA
Identity (separate; ref = None)	C of E	Unadjusted	1100	0.5529	0.4315	0.7085	<0.0001	0.3602	0.0001
	Catholic			0.6200	0.3788	1.0145	0.0571	0.1810	
	Other			0.6741	0.4444	1.0225	0.0635	0.9824	
	C of E	Adjusted	1100	0.6271	0.4848	0.8110	0.0004	0.2145	0.0049
	Catholic			0.6820	0.4099	1.1349	0.1408	0.1221	
	Other			0.7730	0.5020	1.1903	0.2424	0.9998	
Attendance (ref = Occasional/None)	Regular	Unadjusted	1112	1.495	1.057	2.115	0.0230	0.9696	NA
	Regular	Adjusted	1112	1.352	0.944	1.938	0.1002	0.9728	NA
Latent class (ref = "Atheist")	Agnostic	Unadjusted	1120	0.870	0.658	1.149	0.3253	0.2862	0.0131
	Moderately religious			0.715	0.509	1.005	0.0533	0.7146	
	Highly religious			1.509	1.025	2.223	0.0372	0.9526	
	Agnostic	Adjusted	1120	1.007	0.756	1.342	0.9606	0.4379	0.0394
	Moderately religious			0.743	0.523	1.057	0.0986	0.6425	
	Highly religious			1.516	1.013	2.268	0.0429	0.9879	

Table S23: Results of the partners ordinal regression models with 'believes that humans are to blame for climate change' as the outcome for four religious exposures (belief, identity [both Christian denominations combined together and separate], attendance, and latent classes). Odds ratios above 1 indicate an increased belief that humans are to blame for climate change. The 'brant' column shows the *p*-value of the brant test for said variable. The '*p* total' column is the *p*-value for the overall association between the exposure and outcome (if three or more exposure levels). OR = Odds ratio; LCI = Lower 95% confidence interval; UCI = Upper 95% confidence interval.

**Exposure**	**Exposure level**	**Model**	***n***	**OR**	**LCI**	**UCI**	***p*-value**	**brant**	***p* total**
Belief (ref = No)	Not sure	Unadjusted	1111	0.692	0.525	0.911	0.0088	0.9778	0.0088
	Yes			0.718	0.553	0.933	0.0131	0.5192	
	Not sure	Adjusted	1111	0.741	0.560	0.982	0.0371	0.5763	0.0403
	Yes			0.748	0.571	0.980	0.0352	0.1938	
Identity (combined; ref = None)	Christian	Unadjusted	1100	0.651	0.520	0.816	0.0002	0.3406	NA
	Christian	Adjusted	1100	0.707	0.561	0.892	0.0035	0.9849	NA
Identity (separate; ref = None)	C of E	Unadjusted	1100	0.653	0.513	0.832	0.0006	0.4599	0.0023
	Catholic			0.759	0.461	1.250	0.2791	0.9088	
	Other			0.588	0.394	0.878	0.0094	0.8379	
	C of E	Adjusted	1100	0.717	0.559	0.920	0.0089	0.8340	0.0257
	Catholic			0.799	0.480	1.331	0.3896	0.9988	
	Other			0.617	0.408	0.933	0.0219	0.6808	
Attendance (ref = Occasional/None)	Regular	Unadjusted	1113	1.205	0.864	1.681	0.2715	0.9978	NA
	Regular	Adjusted	1113	1.148	0.814	1.618	0.4326	0.9988	NA
Latent class (ref = "Atheist")	Agnostic	Unadjusted	1121	0.815	0.618	1.075	0.1472	0.9863	0.0127
	Moderately religious			0.595	0.429	0.827	0.0020	0.3710	
	Highly religious			1.036	0.717	1.499	0.8493	0.9586	
	Agnostic	Adjusted	1121	0.891	0.672	1.181	0.4216	0.6069	0.0378
	Moderately religious			0.617	0.440	0.864	0.0049	0.3141	
	Highly religious			1.029	0.702	1.507	0.8837	0.9471	

Table S24: Results of the partners multinomial regression models with 'thinks that personal actions will make a difference to long-term climate change' as the outcome for four religious exposures (belief, identity [both Christian denominations combined together and separate], attendance, and latent classes). The '*p* total' column is the *p*-value for the overall association between the exposure and outcome. RRR = Relative risk ratio; LCI = Lower 95% confidence interval; UCI = Upper 95% confidence interval.

**Exposure**	**Exposure level**	**Outcome level (ref = No)**	**Model**	***n***	**RRR**	**LCI**	**UCI**	***p*-value**	***p* total**
Belief (ref = No)	Not sure	Not sure	Unadjusted	1110	1.566	1.009	2.431	0.0457	0.0148
		Yes			1.037	0.710	1.514	0.8524	
	Yes	Not sure			1.398	0.884	2.210	0.1517	
		Yes			1.546	1.061	2.253	0.0234	
	Not sure	Not sure	Adjusted	1110	1.606	1.021	2.526	0.0404	0.0134
		Yes			1.084	0.734	1.601	0.6847	
	Yes	Not sure			1.316	0.817	2.120	0.2590	
		Yes			1.588	1.072	2.353	0.0212	
Identity (combined; ref = None)	Christian	Not sure	Unadjusted	1099	1.197	0.824	1.741	0.3454	0.5390
		Yes			1.021	0.749	1.393	0.8951	
	Christian	Not sure	Adjusted	1099	1.191	0.806	1.761	0.3794	0.6778
		Yes			1.087	0.786	1.504	0.6138	
Identity (separate; ref = None)	C of E	Not sure	Unadjusted	1099	1.316	0.883	1.962	0.1777	0.6225
		Yes			1.023	0.731	1.431	0.8962	
	Catholic	Not sure			0.777	0.325	1.859	0.5712	
		Yes			1.007	0.516	1.966	0.9837	
	Other	Not sure			0.960	0.482	1.912	0.9076	
		Yes			1.024	0.587	1.784	0.9339	
	C of E	Not sure	Adjusted	1099	1.342	0.885	2.034	0.1655	0.6321
		Yes			1.111	0.783	1.576	0.5567	
	Catholic	Not sure			0.767	0.312	1.883	0.5622	
		Yes			1.001	0.501	2.001	0.9982	
	Other	Not sure			0.852	0.418	1.735	0.6586	
		Yes			1.034	0.580	1.845	0.9087	
Attendance (ref = Occasional/None)	Regular	Not sure	Unadjusted	1112	1.760	0.928	3.339	0.0834	0.0164
		Yes			2.126	1.222	3.699	0.0076	
	Regular	Not sure	Adjusted	1112	1.716	0.891	3.304	0.1064	0.0423
		Yes			1.987	1.127	3.501	0.0176	
	Agnostic	Not sure	Unadjusted	1120	1.450	0.925	2.274	0.1053	0.0362

Latent class (ref = "Atheist")		Yes			1.179	0.801	1.735	0.4030	
	Moderately religious	Not sure			0.923	0.517	1.649	0.7868	
		Yes			1.279	0.811	2.017	0.2897	
	Highly religious	Not sure			2.130	1.047	4.331	0.0368	
		Yes			2.385	1.283	4.433	0.0060	
	Agnostic	Not sure	Adjusted	1120	1.543	0.971	2.453	0.0667	0.0254
		Yes			1.259	0.846	1.873	0.2558	
	Moderately religious	Not sure			0.893	0.492	1.623	0.7107	
		Yes			1.384	0.863	2.219	0.1778	
	Highly religious	Not sure			2.042	0.987	4.227	0.0544	
		Yes			2.332	1.235	4.405	0.0091	

Table S25: Results of the partners linear regression models with 'total number of actions performed due to climate change' as the outcome for four religious exposures (belief, identity [both Christian denominations combined together and separate], attendance, and latent classes). Values above 0 indicate an increased number of pro-environmental actions performed. The '*p* total' column is the *p*-value for the overall association between the exposure and outcome (if three or more exposure levels). *b* = Mean difference; LCI = Lower 95% confidence interval; UCI = Upper 95% confidence interval.

**Exposure**	**Exposure level**	**Model**	***n***	***b***	**LCI**	**UCI**	***p*-value**	***p* total**
Belief (ref = No)	Not sure	Unadjusted	984	-0.461	-1.107	0.185	0.1617	0.3617
	Yes			-0.086	-0.707	0.536	0.7869	
	Not sure	Adjusted	984	-0.339	-0.975	0.298	0.2966	0.5095
	Yes			0.031	-0.592	0.654	0.9234	
Identity (combined; ref = None)	Christian	Unadjusted	975	-1.039	-1.569	-0.509	0.0001	NA
	Christian	Adjusted	975	-0.785	-1.315	-0.254	0.0038	NA
Identity (separate; ref = None)	C of E	Unadjusted	975	-1.096	-1.663	-0.530	0.0002	0.0016
	Catholic			-1.107	-2.290	0.076	0.0665	
	Other			-0.729	-1.680	0.222	0.1327	
	C of E	Adjusted	975	-0.843	-1.409	-0.278	0.0035	0.0247
	Catholic			-0.955	-2.121	0.211	0.1083	
	Other			-0.389	-1.337	0.560	0.4213	
Attendance (ref = Occasional/None)	Regular	Unadjusted	989	1.172	0.394	1.951	0.0032	NA
	Regular	Adjusted	989	0.913	0.136	1.690	0.0212	NA
Latent class (ref = "Atheist")	Agnostic	Unadjusted	993	-0.226	-0.868	0.415	0.4886	0.0013
	Moderately religious			-0.590	-1.369	0.190	0.1381	
	Highly religious			1.401	0.540	2.262	0.0014	
	Agnostic	Adjusted	993	0.015	-0.618	0.649	0.9622	0.0017
	Moderately religious			-0.342	-1.114	0.430	0.3848	
	Highly religious			1.363	0.505	2.221	0.0019	

Table S26: Results of the partners Poisson regression models with ‘total number of actions performed due to climate change’ as the outcome for four religious exposures (belief, identity [both Christian denominations combined together and separate], attendance, and latent classes). Incidence rate ratios above 1 indicate an increased number of pro-environmental actions performed. The ‘*p* total’ column is the *p*-value for the overall association between the exposure and outcome (if three or more exposure levels). IRR = Incidence rate ratio; LCI = Lower 95% confidence interval; UCI = Upper 95% confidence interval.

**Exposure**	**Exposure level**	**Model**	***n***	**IRR**	**LCI**	**UCI**	***p*-value**	***p* total**
Belief (ref = No)	Not sure	Unadjusted	984	0.912	0.851	0.978	0.0095	0.0313
	Yes			0.984	0.922	1.050	0.6208	
	Not sure	Adjusted	984	0.935	0.871	1.003	0.0600	0.1102
	Yes			1.008	0.942	1.078	0.8212	
Identity (combined; ref = None)	Christian	Unadjusted	975	0.818	0.774	0.865	<0.0001	NA
	Christian	Adjusted	975	0.859	0.811	0.910	<0.0001	NA
Identity (separate; ref = None)	C of E	Unadjusted	975	0.808	0.761	0.858	<0.0001	<0.0001
	Catholic			0.806	0.708	0.919	0.0012	
	Other			0.872	0.789	0.965	0.0081	
	C of E	Adjusted	975	0.848	0.797	0.902	<0.0001	<0.0001
	Catholic			0.830	0.727	0.947	0.0057	
	Other			0.936	0.843	1.038	0.2117	
Attendance (ref = Occasional/None)	Regular	Unadjusted	989	1.237	1.145	1.335	<0.0001	NA
	Regular	Adjusted	989	1.177	1.087	1.274	0.0001	NA
Latent class (ref = “Atheist”)	Agnostic	Unadjusted	993	0.956	0.891	1.024	0.2002	<0.0001
	Moderately religious			0.884	0.810	0.965	0.0058	
	Highly religious			1.275	1.173	1.386	<0.0001	
	Agnostic	Adjusted	993	1.003	0.934	1.076	0.9445	<0.0001
	Moderately religious			0.931	0.852	1.018	0.1175	
	Highly religious			1.269	1.164	1.384	<0.0001	

Table S27: Results of the partners zero-inflated Poisson regression models with 'total number of actions performed due to climate change' as the outcome for four religious exposures (belief, identity [both Christian denominations combined together and separate], attendance, and latent classes). Incidence rate ratios above 1 indicate an increased number of pro-environmental actions performed, while odds ratios above 1 indicate an increased probability of excess zeros. IRR = Incidence rate ratio; LCI = Lower 95% confidence interval; UCI = Upper 95% confidence interval; OR = Odds ratio.

**Exposure**	**Exposure level**	**Model**	***n***	**IRR**	**IRR LCI**	**IRR UCI**	**IRR *p***	**OR**	**OR LCI**	**OR UCI**	**OR *p***
Belief (ref = No)	Not sure	Unadjusted	984	0.872	0.813	0.935	0.0001	0.798	0.539	1.180	0.2587
	Yes			0.965	0.904	1.031	0.2925	0.916	0.636	1.320	0.6374
	Not sure	Adjusted	984	0.877	0.817	0.941	0.0003	0.733	0.485	1.107	0.1401
	Yes			0.984	0.920	1.053	0.6438	0.914	0.619	1.350	0.6526
Identity (combined; ref = None)	Christian	Unadjusted	975	0.873	0.825	0.923	<0.0001	1.372	0.995	1.893	0.0537
	Christian	Adjusted	975	0.898	0.847	0.951	0.0003	1.261	0.896	1.774	0.1835
Identity (separate; ref = None)	C of E	Unadjusted	975	0.876	0.825	0.931	<0.0001	1.471	1.049	2.064	0.0254
	Catholic			0.774	0.679	0.884	0.0002	0.778	0.345	1.752	0.5439
	Other			0.920	0.832	1.019	0.1092	1.308	0.747	2.292	0.3476
	C of E	Adjusted	975	0.901	0.847	0.959	0.0011	0.835	0.720	0.968	0.1006
	Catholic			0.786	0.687	0.899	0.0004	1.003	0.840	1.198	0.4178
	Other			0.957	0.862	1.063	0.4129	1.085	1.015	1.159	0.5277
Attendance (ref = Occasional/None)	Regular	Unadjusted	989	1.130	1.046	1.220	0.0019	0.600	0.354	1.016	0.0575
	Regular	Adjusted	989	1.093	1.010	1.184	0.0282	0.666	0.384	1.155	0.1479
Latent class (ref = "Atheist")	Agnostic	Unadjusted	993	0.859	0.801	0.921	<0.0001	0.573	0.379	0.869	0.0087
	Moderately religious			0.893	0.818	0.975	0.0118	1.042	0.671	1.619	0.8537
	Highly religious			1.118	1.028	1.215	0.0092	0.478	0.262	0.870	0.0157
	Agnostic	Adjusted	993	0.881	0.820	0.947	0.0006	0.488	0.315	0.756	0.0013
	Moderately religious			0.925	0.846	1.011	0.0873	1.002	0.628	1.599	0.9937
	Highly religious			1.119	1.026	1.220	0.0113	0.489	0.262	0.915	0.0252

Table S28: Results of the partners linear regression models with ‘total number of actions performed due to climate change (excluding ones which may be prohibitively costly)’ as the outcome for four religious exposures (belief, identity [both Christian denominations combined together and separate], attendance, and latent classes). Values above 0 indicate an increased number of pro-environmental actions performed. The ‘*p* total’ column is the *p*-value for the overall association between the exposure and outcome (if three or more exposure levels). *b* = Mean difference; LCI = Lower 95% confidence interval; UCI = Upper 95% confidence interval.

**Exposure**	**Exposure level**	**Model**	***n***	***b***	**LCI**	**UCI**	***p*-value**	***p* total**
Belief (ref = No)	Not sure	Unadjusted	1002	-0.350	-0.874	0.174	0.1907	0.4112
	Yes			-0.067	-0.574	0.440	0.7958	
	Not sure	Adjusted	1002	-0.254	-0.771	0.264	0.3361	0.5720
	Yes			0.015	-0.495	0.524	0.9549	
Identity (combined; ref = None)	Christian	Unadjusted	993	-0.888	-1.319	-0.457	0.0001	NA
	Christian	Adjusted	993	-0.706	-1.139	-0.274	0.0014	NA
Identity (separate; ref = None)	C of E	Unadjusted	993	-0.973	-1.434	-0.513	<0.0001	0.0006
	Catholic			-0.751	-1.698	0.195	0.1197	
	Other			-0.563	-1.342	0.216	0.1567	
	C of E	Adjusted	993	-0.788	-1.249	-0.327	0.0008	0.0084
	Catholic			-0.697	-1.634	0.241	0.1451	
	Other			-0.306	-1.085	0.473	0.4405	
Attendance (ref = Occasional/None)	Regular	Unadjusted	1007	0.797	0.160	1.435	0.0143	NA
	Regular	Adjusted	1007	0.619	-0.018	1.256	0.0570	NA
Latent class (ref = “Atheist”)	Agnostic	Unadjusted	1011	-0.179	-0.700	0.343	0.5011	0.0042
	Moderately religious			-0.407	-1.041	0.227	0.2078	
	Highly religious			1.071	0.363	1.779	0.0031	
	Agnostic	Adjusted	1011	0.011	-0.505	0.527	0.9659	0.0149
	Moderately religious			-0.232	-0.861	0.398	0.4705	
	Highly religious			1.059	0.352	1.765	0.0034	

Table S29: Results of the partners Poisson regression models with 'total number of actions performed due to climate change (excluding ones which may be prohibitively costly)' as the outcome for four religious exposures (belief, identity [both Christian denominations combined together and separate], attendance, and latent classes). Incidence rate ratios above 1 indicate an increased number of pro-environmental actions performed. The '*p* total' column is the *p*-value for the overall association between the exposure and outcome (if three or more exposure levels). IRR = Incidence rate ratio; LCI = Lower 95% confidence interval; UCI = Upper 95% confidence interval.

**Exposure**	**Exposure level**	**Model**	***n***	**IRR**	**LCI**	**UCI**	***p*-value**	***p* total**
Belief (ref = No)	Not sure	Unadjusted	1002	0.922	0.856	0.992	0.0308	0.0904
	Yes			0.985	0.918	1.057	0.6730	
	Not sure	Adjusted	1002	0.942	0.874	1.016	0.1222	0.2353
	Yes			1.005	0.934	1.080	0.9011	
Identity (combined; ref = None)	Christian	Unadjusted	993	0.818	0.770	0.868	<0.0001	NA
	Christian	Adjusted	993	0.851	0.800	0.905	<0.0001	NA
Identity (separate; ref = None)	C of E	Unadjusted	993	0.800	0.750	0.854	<0.0001	<0.0001
	Catholic			0.846	0.739	0.968	0.0152	
	Other			0.884	0.793	0.986	0.0268	
	C of E	Adjusted	993	0.833	0.780	0.890	<0.0001	<0.0001
	Catholic			0.856	0.746	0.981	0.0258	
	Other			0.941	0.841	1.052	0.2843	
Attendance (ref = Occasional/None)	Regular	Unadjusted	1007	1.188	1.092	1.291	0.0001	NA
	Regular	Adjusted	1007	1.140	1.046	1.243	0.0029	NA
Latent class (ref = "Atheist")	Agnostic	Unadjusted	1011	0.959	0.890	1.033	0.2673	<0.0001
	Moderately religious			0.906	0.826	0.994	0.0371	
	Highly religious			1.247	1.138	1.366	<0.0001	
	Agnostic	Adjusted	1011	1.002	0.929	1.081	0.9597	<0.0001
	Moderately religious			0.945	0.860	1.038	0.2385	
	Highly religious			1.245	1.133	1.368	<0.0001	

Table S30: Results of the partners zero-inflated Poisson regression models with 'total number of actions performed due to climate change (excluding ones which may be prohibitively costly)' as the outcome for four religious exposures (belief, identity [both Christian denominations combined together and separate], attendance, and latent classes). Incidence rate ratios above 1 indicate an increased number of pro-environmental actions performed, while odds ratios above 1 indicate an increased probability of excess zeros. IRR = Incidence rate ratio; LCI = Lower 95% confidence interval; UCI = Upper 95% confidence interval; OR = Odds ratio.

**Exposure**	**Exposure level**	**Model**	***n***	**IRR**	**IRR LCI**	**IRR UCI**	**IRR *p***	**OR**	**OR LCI**	**OR UCI**	**OR *p***
Belief (ref = No)	Not sure	Unadjusted	1002	0.887	0.823	0.956	0.0017	0.835	0.571	1.221	0.3521
	Yes			0.963	0.897	1.033	0.2926	0.901	0.629	1.292	0.5710
	Not sure	Adjusted	1002	0.889	0.823	0.959	0.0025	0.769	0.515	1.148	0.1982
	Yes			0.983	0.913	1.057	0.6378	0.916	0.624	1.344	0.6535
Identity (combined; ref = None)	Christian	Unadjusted	993	0.874	0.823	0.928	<0.0001	1.367	0.998	1.874	0.0516
	Christian	Adjusted	993	0.895	0.841	0.953	0.0005	1.284	0.920	1.794	0.1420
Identity (separate; ref = None)	C of E	Unadjusted	993	0.873	0.818	0.932	<0.0001	1.487	1.068	2.072	0.0189
	Catholic			0.810	0.706	0.930	0.0028	0.776	0.354	1.700	0.5261
	Other			0.921	0.825	1.028	0.1421	1.221	0.696	2.143	0.4862
	C of E	Adjusted	993	0.895	0.837	0.957	0.0013	0.820	0.695	0.967	0.0632
	Catholic			0.810	0.704	0.932	0.0032	1.033	0.859	1.243	0.4275
	Other			0.957	0.855	1.072	0.4504	1.065	0.991	1.145	0.6191
Attendance (ref = Occasional/None)	Regular	Unadjusted	1007	1.089	1.001	1.185	0.0471	0.633	0.380	1.053	0.0784
	Regular	Adjusted	1007	1.070	0.980	1.168	0.1289	0.717	0.421	1.221	0.2203
Latent class (ref = "Atheist")	Agnostic	Unadjusted	1011	0.872	0.809	0.941	0.0004	0.630	0.423	0.938	0.0230
	Moderately religious			0.922	0.840	1.013	0.0905	1.072	0.697	1.649	0.7505
	Highly religious			1.081	0.987	1.185	0.0945	0.455	0.249	0.832	0.0106
	Agnostic	Adjusted	1011	0.889	0.823	0.960	0.0027	0.541	0.355	0.824	0.0042
	Moderately religious			0.947	0.861	1.042	0.2638	1.042	0.661	1.645	0.8582
	Highly religious			1.090	0.991	1.198	0.0768	0.476	0.254	0.894	0.0210

Table S31: Results of the partners multinomial regression models for each of the individual climate change action outcomes for four religious exposures (belief, identity [both Christian denominations combined together and separate], attendance, and latent classes). The '*p* total' column is the *p*-value for the overall association between the exposure and outcome. RRR = Relative risk ratio; LCI = Lower 95% confidence interval; UCI = Upper 95% confidence interval.

**Exposure**	**Exposure level**	**Outcome level (ref = No)**	**Model**	***n***	**RRR**	**LCI**	**UCI**	***p*-value**	***p* total**
Changed the way travel locally									
Belief (ref = No)	Not sure	For climate reasons	Unadjusted	1108	0.496	0.337	0.729	0.0004	0.0084
		For other reasons			0.710	0.482	1.047	0.0840	
		For climate and other			0.422	0.167	1.067	0.0683	
	Yes	For climate reasons			0.741	0.521	1.055	0.0961	
		For other reasons			1.006	0.700	1.446	0.9732	
		For climate and other			0.879	0.421	1.835	0.7320	
	Not sure	For climate reasons	Adjusted	1108	0.500	0.334	0.750	0.0008	0.0214
		For other reasons			0.691	0.463	1.032	0.0710	
		For climate and other			0.423	0.164	1.091	0.0751	
	Yes	For climate reasons			0.795	0.545	1.158	0.2319	
		For other reasons			0.953	0.651	1.394	0.8025	
		For climate and other			0.919	0.424	1.991	0.8311	
Identity (combined; ref = None)	Christian	For climate reasons	Unadjusted	1096	0.460	0.341	0.622	<0.0001	<0.0001
		For other reasons			0.700	0.511	0.959	0.0263	
		For climate and other			0.558	0.291	1.068	0.0782	
	Christian	For climate reasons	Adjusted	1096	0.500	0.363	0.688	<0.0001	0.0002
		For other reasons			0.677	0.487	0.941	0.0204	
		For climate and other			0.615	0.312	1.214	0.1612	
Identity (separate; ref = None)	C of E	For climate reasons	Unadjusted	1096	0.446	0.321	0.619	<0.0001	0.0004
		For other reasons			0.706	0.504	0.988	0.0423	
		For climate and other			0.432	0.203	0.920	0.0296	
	Catholic	For climate reasons			0.585	0.299	1.145	0.1176	
		For other reasons			0.695	0.339	1.425	0.3210	
		For climate and other			0.939	0.265	3.325	0.9217	
	Other	For climate reasons			0.454	0.254	0.811	0.0076	
		For other reasons			0.675	0.378	1.204	0.1829	
		For climate and other			0.928	0.334	2.577	0.8859	
	C of E	For climate reasons	Adjusted	1096	0.492	0.348	0.696	0.0001	0.0063
		For other reasons			0.692	0.487	0.984	0.0402	
		For climate and other			0.470	0.215	1.030	0.0593	

Exposure	Exposure level	Outcome level (ref = No)	Model	*n*	RRR	LCI	UCI	*p*-value	*p* total
	Catholic	For climate reasons			0.570	0.280	1.159	0.1207	
		For other reasons			0.676	0.322	1.419	0.3002	
		For climate and other			1.147	0.306	4.293	0.8386	
	Other	For climate reasons			0.491	0.266	0.907	0.0231	
		For other reasons			0.610	0.334	1.113	0.1071	
		For climate and other			1.039	0.356	3.038	0.9436	
Attendance (ref = Occasional/None)	Regular	For climate reasons	Unadjusted	1110	1.430	0.927	2.206	0.1062	0.1093
		For other reasons			1.194	0.744	1.916	0.4621	
		For climate and other			2.469	1.123	5.430	0.0246	
	Regular	For climate reasons	Adjusted	1110	1.249	0.786	1.982	0.3466	0.3191
		For other reasons			1.078	0.659	1.761	0.7653	
		For climate and other			2.169	0.952	4.945	0.0655	
Latent class (ref = "Atheist")	Agnostic	For climate reasons	Unadjusted	1117	0.542	0.364	0.805	0.0024	0.0103
		For other reasons			0.838	0.572	1.226	0.3624	
		For climate and other			0.652	0.271	1.566	0.3384	
	Moderately religious	For climate reasons			0.569	0.350	0.923	0.0225	
		For other reasons			0.962	0.615	1.505	0.8650	
		For climate and other			0.641	0.215	1.917	0.4265	
	Highly religious	For climate reasons			1.403	0.884	2.227	0.1512	
		For other reasons			0.990	0.573	1.711	0.9719	
		For climate and other			1.870	0.758	4.614	0.1742	
	Agnostic	For climate reasons	Adjusted	1117	0.586	0.386	0.888	0.0117	0.0831
		For other reasons			0.809	0.545	1.200	0.2915	
		For climate and other			0.690	0.280	1.700	0.4205	
	Moderately religious	For climate reasons			0.635	0.382	1.055	0.0795	
		For other reasons			0.938	0.588	1.495	0.7881	
		For climate and other			0.711	0.231	2.190	0.5524	
	Highly religious	For climate reasons			1.361	0.828	2.236	0.2236	
		For other reasons			0.871	0.494	1.535	0.6325	
		For climate and other			1.802	0.698	4.652	0.2236	
Reduced household waste									
Belief (ref = No)	Not sure	For climate reasons	Unadjusted	1109	1.252	0.787	1.992	0.3427	0.9142
		For other reasons			1.376	0.835	2.269	0.2103	

Exposure	Exposure level	Outcome level (ref = No)	Model	*n*	RRR	LCI	UCI	*p*-value	*p* total
		For climate and other			1.121	0.473	2.656	0.7947	
	Yes	For climate reasons			1.112	0.724	1.708	0.6290	
		For other reasons			1.170	0.733	1.865	0.5109	
		For climate and other			0.869	0.373	2.025	0.7452	
	Not sure	For climate reasons	Adjusted	1109	1.262	0.785	2.031	0.3365	0.9039
		For other reasons			1.422	0.852	2.372	0.1780	
		For climate and other			1.238	0.508	3.018	0.6379	
	Yes	For climate reasons			1.046	0.670	1.635	0.8420	
		For other reasons			1.138	0.701	1.846	0.6014	
		For climate and other			0.878	0.364	2.118	0.7729	
Identity (combined; ref = None)	Christian	For climate reasons	Unadjusted	1098	0.952	0.661	1.372	0.7923	0.2768
		For other reasons			1.234	0.828	1.840	0.3019	
		For climate and other			0.830	0.414	1.662	0.5985	
	Christian	For climate reasons	Adjusted	1098	0.930	0.636	1.359	0.7085	0.2556
		For other reasons			1.249	0.825	1.891	0.2928	
		For climate and other			0.940	0.454	1.943	0.8667	
Identity (separate; ref = None)	C of E	For climate reasons	Unadjusted	1098	0.998	0.673	1.482	0.9935	0.1884
		For other reasons			1.286	0.836	1.977	0.2523	
		For climate and other			0.717	0.328	1.570	0.4057	
	Catholic	For climate reasons			1.934	0.662	5.654	0.2282	
		For other reasons			2.909	0.961	8.806	0.0588	
		For climate and other			1.600	0.272	9.393	0.6029	
	Other	For climate reasons			0.605	0.333	1.097	0.0977	
		For other reasons			0.724	0.372	1.407	0.3404	
		For climate and other			1.010	0.355	2.877	0.9844	
	C of E	For climate reasons	Adjusted	1098	0.992	0.659	1.493	0.9690	0.1666
		For other reasons			1.330	0.851	2.076	0.2104	
		For climate and other			0.830	0.368	1.871	0.6535	
	Catholic	For climate reasons			1.783	0.597	5.327	0.3005	
		For other reasons			2.924	0.945	9.045	0.0626	
		For climate and other			1.994	0.327	12.178	0.4547	
	Other	For climate reasons			0.564	0.303	1.050	0.0710	
		For other reasons			0.663	0.332	1.324	0.2442	
		For climate and other			1.008	0.338	3.006	0.9884	

Exposure	Exposure level	Outcome level (ref = No)	Model	*n*	RRR	LCI	UCI	*p*-value	*p* total
Attendance (ref = Occasional/None)	Regular	For climate reasons	Unadjusted	1111	0.848	0.492	1.461	0.5525	0.6130
		For other reasons			1.050	0.588	1.874	0.8703	
		For climate and other			1.325	0.515	3.408	0.5590	
	Regular	For climate reasons	Adjusted	1111	0.797	0.454	1.398	0.4281	0.5976
		For other reasons			1.020	0.561	1.854	0.9479	
		For climate and other			1.153	0.436	3.046	0.7739	
Latent class (ref = "Atheist")	Agnostic	For climate reasons	Unadjusted	1119	1.166	0.732	1.855	0.5186	0.9840
		For other reasons			1.055	0.638	1.745	0.8353	
		For climate and other			1.238	0.522	2.934	0.6275	
	Moderately religious	For climate reasons			1.107	0.635	1.928	0.7203	
		For other reasons			1.032	0.566	1.882	0.9177	
		For climate and other			0.977	0.326	2.927	0.9674	
	Highly religious	For climate reasons			0.869	0.487	1.551	0.6354	
		For other reasons			0.743	0.391	1.412	0.3644	
		For climate and other			1.238	0.437	3.510	0.6878	
	Agnostic	For climate reasons	Adjusted	1119	1.219	0.756	1.966	0.4164	0.9641
		For other reasons			1.108	0.661	1.857	0.6976	
		For climate and other			1.444	0.592	3.520	0.4196	
	Moderately religious	For climate reasons			1.094	0.617	1.938	0.7593	
		For other reasons			1.018	0.548	1.889	0.9555	
		For climate and other			1.052	0.340	3.252	0.9302	
	Highly religious	For climate reasons			0.796	0.436	1.450	0.4555	
		For other reasons			0.681	0.351	1.321	0.2557	
		For climate and other			1.091	0.372	3.200	0.8741	
Reduced energy use at home									
Belief (ref = No)	Not sure	For climate reasons	Unadjusted	1105	0.810	0.554	1.186	0.2796	0.0498
		For other reasons			1.186	0.799	1.760	0.3977	
		For climate and other			0.803	0.391	1.648	0.5500	
	Yes	For climate reasons			1.431	0.977	2.097	0.0656	
		For other reasons			1.550	1.031	2.328	0.0349	
		For climate and other			0.827	0.385	1.776	0.6270	
	Not sure	For climate reasons	Adjusted	1105	0.850	0.575	1.257	0.4165	0.1298
		For other reasons			1.228	0.819	1.842	0.3207	

Exposure	Exposure level	Outcome level (ref = No)	Model	*n*	RRR	LCI	UCI	*p*-value	*p* total
		For climate and other			0.850	0.406	1.778	0.6663	
	Yes	For climate reasons			1.410	0.948	2.096	0.0898	
		For other reasons			1.462	0.958	2.231	0.0782	
		For climate and other			0.847	0.383	1.871	0.6812	
Identity (combined; ref = None)	Christian	For climate reasons	Unadjusted	1094	1.010	0.738	1.382	0.9516	0.0249
		For other reasons			1.487	1.062	2.082	0.0210	
		For climate and other			0.861	0.472	1.570	0.6259	
	Christian	For climate reasons	Adjusted	1094	1.032	0.744	1.431	0.8507	0.1340
		For other reasons			1.407	0.992	1.996	0.0557	
		For climate and other			0.935	0.500	1.749	0.8338	
Identity (separate; ref = None)	C of E	For climate reasons	Unadjusted	1094	1.120	0.797	1.574	0.5146	0.1008
		For other reasons			1.592	1.106	2.291	0.0124	
		For climate and other			0.761	0.384	1.505	0.4320	
	Catholic	For climate reasons			0.812	0.400	1.648	0.5635	
		For other reasons			1.446	0.709	2.947	0.3103	
		For climate and other			0.907	0.243	3.388	0.8841	
	Other	For climate reasons			0.723	0.412	1.269	0.2584	
		For other reasons			1.126	0.629	2.017	0.6888	
		For climate and other			1.185	0.462	3.035	0.7241	
	C of E	For climate reasons	Adjusted	1094	1.158	0.813	1.650	0.4170	0.2209
		For other reasons			1.543	1.058	2.250	0.0242	
		For climate and other			0.832	0.410	1.688	0.6112	
	Catholic	For climate reasons			0.798	0.384	1.657	0.5443	
		For other reasons			1.365	0.655	2.846	0.4062	
		For climate and other			1.023	0.261	4.001	0.9743	
	Other	For climate reasons			0.715	0.398	1.284	0.2618	
		For other reasons			0.951	0.518	1.745	0.8715	
		For climate and other			1.235	0.462	3.302	0.6738	
Attendance (ref = Occasional/None)	Regular	For climate reasons	Unadjusted	1107	1.623	0.984	2.677	0.0581	0.1479
		For other reasons			1.182	0.685	2.041	0.5476	
		For climate and other			1.904	0.826	4.388	0.1304	
	Regular	For climate reasons	Adjusted	1107	1.475	0.881	2.467	0.1393	0.3767
		For other reasons			1.187	0.677	2.082	0.5501	
		For climate and other			1.731	0.732	4.090	0.2113	

**Exposure**	**Exposure level**	**Outcome level (ref = No)**	**Model**	***n***	**RRR**	**LCI**	**UCI**	***p*-value**	***p* total**
Latent class (ref = "Atheist")	Agnostic	For climate reasons	Unadjusted	1115	1.003	0.679	1.481	0.9891	0.1535
		For other reasons			1.286	0.858	1.926	0.2231	
		For climate and other			1.133	0.549	2.339	0.7359	
	Moderately religious	For climate reasons			1.223	0.752	1.988	0.4182	
		For other reasons			1.579	0.958	2.603	0.0731	
		For climate and other			0.826	0.295	2.315	0.7168	
	Highly religious	For climate reasons			1.688	0.988	2.885	0.0554	
		For other reasons			1.071	0.583	1.968	0.8243	
		For climate and other			1.598	0.621	4.108	0.3308	
	Agnostic	For climate reasons	Adjusted	1115	1.069	0.715	1.597	0.7462	0.3253
		For other reasons			1.284	0.848	1.946	0.2375	
		For climate and other			1.256	0.596	2.649	0.5489	
	Moderately religious	For climate reasons			1.253	0.759	2.069	0.3771	
		For other reasons			1.538	0.920	2.571	0.1009	
		For climate and other			0.914	0.319	2.622	0.8675	
	Highly religious	For climate reasons			1.577	0.908	2.740	0.1058	
		For other reasons			0.980	0.524	1.835	0.9505	
		For climate and other			1.513	0.570	4.015	0.4059	
Changed what buy									
Belief (ref = No)	Not sure	For climate reasons	Unadjusted	1101	0.877	0.627	1.228	0.4455	0.8947
		For other reasons			0.910	0.607	1.362	0.6456	
		For climate and other			0.782	0.343	1.781	0.5583	
	Yes	For climate reasons			0.860	0.622	1.189	0.3607	
		For other reasons			0.972	0.663	1.426	0.8856	
		For climate and other			0.612	0.261	1.435	0.2588	
	Not sure	For climate reasons	Adjusted	1101	0.936	0.661	1.326	0.7111	0.9654
		For other reasons			0.966	0.638	1.463	0.8714	
		For climate and other			0.982	0.417	2.314	0.9663	
	Yes	For climate reasons			0.905	0.644	1.271	0.5634	
		For other reasons			1.092	0.733	1.627	0.6668	
		For climate and other			0.761	0.314	1.843	0.5449	
Identity (combined; ref = None)	Christian	For climate reasons	Unadjusted	1090	0.584	0.442	0.771	0.0001	0.0012
		For other reasons			0.829	0.594	1.158	0.2725	

**Exposure**	**Exposure level**	**Outcome level (ref = No)**	**Model**	***n***	**RRR**	**LCI**	**UCI**	***p*-value**	***p* total**
		For climate and other			0.556	0.282	1.092	0.0885	
	Christian	For climate reasons	Adjusted	1090	0.634	0.475	0.848	0.0021	0.0142
		For other reasons			0.929	0.656	1.315	0.6780	
		For climate and other			0.775	0.380	1.581	0.4838	
Identity (separate; ref = None)	C of E	For climate reasons	Unadjusted	1090	0.577	0.428	0.778	0.0003	0.0373
		For other reasons			0.848	0.595	1.209	0.3624	
		For climate and other			0.498	0.234	1.060	0.0705	
	Catholic	For climate reasons			0.476	0.255	0.887	0.0195	
		For other reasons			0.748	0.367	1.522	0.4229	
		For climate and other			0.569	0.125	2.587	0.4658	
	Other	For climate reasons			0.694	0.421	1.143	0.1517	
		For other reasons			0.798	0.433	1.469	0.4680	
		For climate and other			0.831	0.267	2.590	0.7494	
	C of E	For climate reasons	Adjusted	1090	0.631	0.462	0.860	0.0036	0.1813
		For other reasons			0.947	0.656	1.369	0.7733	
		For climate and other			0.718	0.326	1.581	0.4113	
	Catholic	For climate reasons			0.477	0.249	0.915	0.0260	
		For other reasons			0.823	0.394	1.719	0.6038	
		For climate and other			0.839	0.174	4.047	0.8269	
	Other	For climate reasons			0.774	0.460	1.301	0.3341	
		For other reasons			0.909	0.484	1.707	0.7658	
		For climate and other			0.992	0.297	3.309	0.9897	
Attendance (ref = Occasional/None)	Regular	For climate reasons	Unadjusted	1103	1.562	1.016	2.402	0.0420	0.1349
		For other reasons			1.378	0.825	2.302	0.2204	
		For climate and other			2.157	0.890	5.230	0.0889	
	Regular	For climate reasons	Adjusted	1103	1.487	0.951	2.324	0.0817	0.2541
		For other reasons			1.424	0.840	2.414	0.1888	
		For climate and other			1.890	0.752	4.748	0.1758	
Latent class (ref = "Atheist")	Agnostic	For climate reasons	Unadjusted	1111	0.758	0.540	1.065	0.1099	0.0407
		For other reasons			0.948	0.639	1.407	0.7920	
		For climate and other			0.677	0.278	1.644	0.3884	
	Moderately religious	For climate reasons			0.625	0.415	0.942	0.0248	
		For other reasons			0.766	0.474	1.238	0.2766	
		For climate and other			0.431	0.125	1.489	0.1831	

Exposure	Exposure level	Outcome level (ref = No)	Model	*n*	RRR	LCI	UCI	*p*-value	*p* total
	Highly religious	For climate reasons			1.643	1.011	2.669	0.0451	
		For other reasons			1.333	0.738	2.407	0.3412	
		For climate and other			2.356	0.921	6.030	0.0738	
	Agnostic	For climate reasons	Adjusted	1111	0.833	0.586	1.183	0.3062	0.1620
		For other reasons			1.007	0.672	1.510	0.9729	
		For climate and other			0.891	0.356	2.233	0.8058	
	Moderately religious	For climate reasons			0.670	0.437	1.026	0.0653	
		For other reasons			0.829	0.506	1.359	0.4575	
		For climate and other			0.559	0.157	1.988	0.3691	
	Highly religious	For climate reasons			1.693	1.023	2.802	0.0407	
		For other reasons			1.433	0.781	2.628	0.2449	
		For climate and other			2.492	0.929	6.681	0.0696	
Reduced air travel									
Belief (ref = No)	Not sure	For climate reasons	Unadjusted	1103	0.633	0.431	0.928	0.0190	0.0063
		For other reasons			0.957	0.678	1.352	0.8050	
		For climate and other			0.833	0.246	2.819	0.7694	
	Yes	For climate reasons			0.499	0.343	0.726	0.0003	
		For other reasons			0.718	0.511	1.009	0.0561	
		For climate and other			1.314	0.484	3.571	0.5918	
	Not sure	For climate reasons	Adjusted	1103	0.700	0.467	1.051	0.0852	0.0548
		For other reasons			0.951	0.665	1.359	0.7822	
		For climate and other			0.889	0.254	3.116	0.8541	
	Yes	For climate reasons			0.552	0.370	0.825	0.0038	
		For other reasons			0.709	0.496	1.014	0.0594	
		For climate and other			1.452	0.503	4.192	0.4907	
Identity (combined; ref = None)	Christian	For climate reasons	Unadjusted	1092	0.429	0.315	0.586	<0.0001	<0.0001
		For other reasons			0.701	0.526	0.936	0.0158	
		For climate and other			0.865	0.347	2.152	0.7546	
	Christian	For climate reasons	Adjusted	1092	0.501	0.360	0.697	<0.0001	0.0005
		For other reasons			0.728	0.539	0.984	0.0390	
		For climate and other			1.001	0.385	2.607	0.9980	
Identity (separate; ref = None)	C of E	For climate reasons	Unadjusted	1092	0.442	0.316	0.619	<0.0001	0.0001
		For other reasons			0.723	0.531	0.983	0.0387	

Exposure	Exposure level	Outcome level (ref = No)	Model	*n*	RRR	LCI	UCI	*p*-value	*p* total
		For climate and other			0.689	0.246	1.932	0.4787	
	Catholic	For climate reasons			0.554	0.275	1.117	0.0986	
		For other reasons			0.849	0.456	1.580	0.6062	
		For climate and other			0.790	0.096	6.539	0.8272	
	Other	For climate reasons			0.310	0.164	0.587	0.0003	
		For other reasons			0.536	0.314	0.916	0.0226	
		For climate and other			1.633	0.475	5.614	0.4360	
	C of E	For climate reasons	Adjusted	1092	0.532	0.372	0.760	0.0005	0.0072
		For other reasons			0.742	0.538	1.022	0.0678	
		For climate and other			0.775	0.264	2.274	0.6424	
	Catholic	For climate reasons			0.586	0.279	1.228	0.1567	
		For other reasons			0.896	0.470	1.708	0.7377	
		For climate and other			1.146	0.129	10.177	0.9025	
	Other	For climate reasons			0.336	0.172	0.658	0.0015	
		For other reasons			0.582	0.334	1.015	0.0566	
		For climate and other			1.957	0.518	7.390	0.3220	
Attendance (ref = Occasional/None)	Regular	For climate reasons	Unadjusted	1105	1.216	0.769	1.921	0.4030	0.1528
		For other reasons			1.172	0.763	1.799	0.4691	
		For climate and other			3.386	1.255	9.139	0.0160	
	Regular	For climate reasons	Adjusted	1105	1.055	0.647	1.720	0.8301	0.1943
		For other reasons			1.084	0.693	1.697	0.7238	
		For climate and other			3.543	1.212	10.359	0.0208	
Latent class (ref = "Atheist")	Agnostic	For climate reasons	Unadjusted	1113	0.634	0.429	0.938	0.0226	0.0021
		For other reasons			0.834	0.590	1.179	0.3041	
		For climate and other			0.596	0.161	2.200	0.4370	
	Moderately religious	For climate reasons			0.413	0.247	0.690	0.0008	
		For other reasons			0.603	0.392	0.927	0.0210	
		For climate and other			0.283	0.036	2.240	0.2316	
	Highly religious	For climate reasons			1.080	0.664	1.759	0.7555	
		For other reasons			0.832	0.506	1.367	0.4669	
		For climate and other			3.012	1.049	8.642	0.0404	
	Agnostic	For climate reasons	Adjusted	1113	0.754	0.498	1.140	0.1808	0.0198
		For other reasons			0.848	0.593	1.213	0.3658	
		For climate and other			0.541	0.141	2.070	0.3691	

**Exposure**	**Exposure level**	**Outcome level (ref = No)**	**Model**	***n***	**RRR**	**LCI**	**UCI**	***p*-value**	***p* total**
	Moderately religious	For climate reasons			0.483	0.282	0.826	0.0079	
		For other reasons			0.593	0.380	0.926	0.0215	
		For climate and other			0.306	0.037	2.523	0.2710	
	Highly religious	For climate reasons			1.069	0.632	1.808	0.8027	
		For other reasons			0.805	0.479	1.351	0.4108	
		For climate and other			3.541	1.113	11.262	0.0322	
Bought or hired an electric or hybrid vehicle									
Belief (ref = No)	Not sure	For climate reasons	Unadjusted	1107	0.821	0.516	1.307	0.4062	0.6517
		For other reasons			1.265	0.658	2.430	0.4808	
		For climate and other			1.898	0.380	9.482	0.4351	
	Yes	For climate reasons			0.772	0.493	1.208	0.2574	
		For other reasons			1.077	0.562	2.066	0.8226	
		For climate and other			2.696	0.639	11.374	0.1770	
	Not sure	For climate reasons	Adjusted	1107	0.942	0.580	1.528	0.8074	0.6662
		For other reasons			1.387	0.709	2.716	0.3392	
		For climate and other			2.568	0.440	14.977	0.2945	
	Yes	For climate reasons			0.881	0.548	1.419	0.6036	
		For other reasons			1.042	0.531	2.043	0.9055	
		For climate and other			3.654	0.712	18.753	0.1205	
Identity (combined; ref = None)	Christian	For climate reasons	Unadjusted	1096	0.639	0.440	0.927	0.0184	0.0605
		For other reasons			0.981	0.569	1.693	0.9461	
		For climate and other			2.208	0.594	8.211	0.2371	
	Christian	For climate reasons	Adjusted	1096	0.738	0.497	1.094	0.1307	0.1596
		For other reasons			1.013	0.575	1.786	0.9635	
		For climate and other			3.060	0.715	13.087	0.1315	
Identity (separate; ref = None)	C of E	For climate reasons	Unadjusted	1096	0.647	0.432	0.970	0.0352	0.1434
		For other reasons			1.042	0.584	1.856	0.8902	
		For climate and other			2.003	0.497	8.070	0.3286	
	Catholic	For climate reasons			0.899	0.408	1.980	0.7909	
		For other reasons			1.273	0.424	3.820	0.6664	
		For climate and other			NA	NA	NA	NA	
	Other	For climate reasons			0.453	0.201	1.019	0.0557	
		For other reasons			0.548	0.161	1.863	0.3357	

**Exposure**	**Exposure level**	**Outcome level (ref = No)**	**Model**	***n***	**RRR**	**LCI**	**UCI**	***p*-value**	***p* total**
		For climate and other			4.393	0.871	22.151	0.0730	
	C of E	For climate reasons	Adjusted	1096	0.753	0.492	1.153	0.1917	0.2284
		For other reasons			1.087	0.598	1.976	0.7851	
		For climate and other			2.899	0.612	13.734	0.1799	
	Catholic	For climate reasons			0.992	0.432	2.280	0.9854	
		For other reasons			1.242	0.394	3.914	0.7114	
		For climate and other			NA	NA	NA	NA	
	Other	For climate reasons			0.510	0.217	1.201	0.1233	
		For other reasons			0.541	0.153	1.907	0.3388	
		For climate and other			5.974	0.999	35.715	0.0501	
Attendance (ref = Occasional/None)	Regular	For climate reasons	Unadjusted	1109	1.106	0.640	1.913	0.7177	0.0805
		For other reasons			0.875	0.366	2.088	0.7626	
		For climate and other			5.204	1.623	16.682	0.0055	
	Regular	For climate reasons	Adjusted	1109	1.047	0.588	1.864	0.8755	0.0748
		For other reasons			0.930	0.380	2.280	0.8748	
		For climate and other			6.073	1.691	21.811	0.0057	
Latent class (ref = "Atheist")	Agnostic	For climate reasons	Unadjusted	1117	0.728	0.451	1.175	0.1937	0.0816
		For other reasons			0.961	0.479	1.925	0.9102	
		For climate and other			1.495	0.248	9.009	0.6611	
	Moderately religious	For climate reasons			0.587	0.310	1.112	0.1020	
		For other reasons			1.614	0.798	3.262	0.1826	
		For climate and other			2.510	0.415	15.184	0.3162	
	Highly religious	For climate reasons			0.999	0.551	1.812	0.9971	
		For other reasons			0.916	0.345	2.432	0.8597	
		For climate and other			8.546	2.008	36.372	0.0037	
	Agnostic	For climate reasons	Adjusted	1117	0.858	0.521	1.412	0.5468	0.1329
		For other reasons			0.946	0.464	1.931	0.8793	
		For climate and other			2.075	0.307	14.028	0.4542	
	Moderately religious	For climate reasons			0.703	0.363	1.361	0.2955	
		For other reasons			1.589	0.766	3.299	0.2138	
		For climate and other			3.279	0.446	24.102	0.2433	
	Highly religious	For climate reasons			1.037	0.551	1.951	0.9101	
		For other reasons			1.007	0.370	2.739	0.9888	
		For climate and other			13.278	2.546	69.251	0.0021	

Exposure	Exposure level	Outcome level (ref = No)	Model	*n*	RRR	LCI	UCI	*p*-value	*p* total
Bought foods produced locally									
Belief (ref = No)	Not sure	For climate reasons	Unadjusted	1108	0.903	0.623	1.309	0.5903	0.5658
		For other reasons			1.108	0.767	1.602	0.5839	
		For climate and other			0.904	0.420	1.946	0.7965	
	Yes	For climate reasons			1.019	0.722	1.438	0.9134	
		For other reasons			0.898	0.627	1.287	0.5587	
		For climate and other			0.550	0.237	1.278	0.1648	
	Not sure	For climate reasons	Adjusted	1108	0.965	0.656	1.419	0.8546	0.7183
		For other reasons			1.178	0.806	1.721	0.3976	
		For climate and other			0.970	0.440	2.138	0.9404	
	Yes	For climate reasons			1.120	0.778	1.612	0.5419	
		For other reasons			0.999	0.686	1.455	0.9962	
		For climate and other			0.656	0.275	1.569	0.3435	
Identity (combined; ref = None)	Christian	For climate reasons	Unadjusted	1097	0.814	0.604	1.097	0.1768	0.3622
		For other reasons			0.904	0.666	1.227	0.5174	
		For climate and other			0.621	0.326	1.183	0.1477	
	Christian	For climate reasons	Adjusted	1097	0.918	0.671	1.258	0.5959	0.8052
		For other reasons			0.998	0.725	1.372	0.9889	
		For climate and other			0.751	0.384	1.468	0.4026	
Identity (separate; ref = None)	C of E	For climate reasons	Unadjusted	1097	0.769	0.557	1.063	0.1114	0.2784
		For other reasons			0.974	0.704	1.349	0.8751	
		For climate and other			0.592	0.291	1.205	0.1482	
	Catholic	For climate reasons			1.084	0.585	2.009	0.7966	
		For other reasons			0.540	0.254	1.146	0.1082	
		For climate and other			0.609	0.133	2.790	0.5232	
	Other	For climate reasons			0.847	0.499	1.439	0.5394	
		For other reasons			0.815	0.468	1.418	0.4694	
		For climate and other			0.761	0.245	2.364	0.6372	
	C of E	For climate reasons	Adjusted	1097	0.858	0.611	1.204	0.3751	0.5949
		For other reasons			1.065	0.759	1.493	0.7160	
		For climate and other			0.726	0.348	1.515	0.3929	
	Catholic	For climate reasons			1.213	0.632	2.330	0.5610	
		For other reasons			0.639	0.295	1.386	0.2572	

Exposure	Exposure level	Outcome level (ref = No)	Model	*n*	RRR	LCI	UCI	*p*-value	*p* total
		For climate and other			0.830	0.175	3.946	0.8151	
	Other	For climate reasons			1.015	0.583	1.766	0.9593	
		For other reasons			0.897	0.505	1.592	0.7101	
		For climate and other			0.815	0.253	2.621	0.7315	
Attendance (ref = Occasional/None)	Regular	For climate reasons	Unadjusted	1110	1.359	0.879	2.100	0.1677	0.1227
		For other reasons			0.817	0.502	1.328	0.4144	
		For climate and other			1.476	0.615	3.543	0.3838	
	Regular	For climate reasons	Adjusted	1110	1.251	0.793	1.973	0.3349	0.2422
		For other reasons			0.802	0.486	1.325	0.3895	
		For climate and other			1.448	0.587	3.572	0.4218	
Latent class (ref = "Atheist")	Agnostic	For climate reasons	Unadjusted	1118	0.862	0.593	1.252	0.4358	0.0780
		For other reasons			1.010	0.699	1.461	0.9570	
		For climate and other			1.073	0.499	2.309	0.8570	
	Moderately religious	For climate reasons			0.750	0.483	1.165	0.2008	
		For other reasons			0.848	0.549	1.312	0.4596	
		For climate and other			0.564	0.187	1.701	0.3090	
	Highly religious	For climate reasons			1.585	0.979	2.566	0.0610	
		For other reasons			0.739	0.420	1.300	0.2940	
		For climate and other			1.227	0.434	3.471	0.6997	
	Agnostic	For climate reasons	Adjusted	1118	0.971	0.658	1.433	0.8828	0.1868
		For other reasons			1.088	0.744	1.591	0.6630	
		For climate and other			1.239	0.561	2.734	0.5960	
	Moderately religious	For climate reasons			0.848	0.536	1.342	0.4819	
		For other reasons			0.960	0.612	1.506	0.8593	
		For climate and other			0.701	0.227	2.168	0.5372	
	Highly religious	For climate reasons			1.635	0.987	2.707	0.0562	
		For other reasons			0.763	0.427	1.364	0.3620	
		For climate and other			1.284	0.440	3.745	0.6474	
Recycled or upcycled more									
Belief (ref = No)	Not sure	For climate reasons	Unadjusted	1107	1.086	0.641	1.840	0.7600	0.6869
		For other reasons			1.032	0.588	1.812	0.9126	
		For climate and other			0.675	0.283	1.613	0.3770	
	Yes	For climate reasons			0.850	0.529	1.365	0.5007	

Exposure	Exposure level	Outcome level (ref = No)	Model	*n*	RRR	LCI	UCI	*p*-value	*p* total
		For other reasons			0.717	0.428	1.201	0.2058	
		For climate and other			0.648	0.299	1.405	0.2721	
	Not sure	For climate reasons	Adjusted	1107	1.115	0.650	1.912	0.6936	0.7143
		For other reasons			1.034	0.582	1.837	0.9104	
		For climate and other			0.763	0.313	1.863	0.5530	
	Yes	For climate reasons			0.758	0.463	1.242	0.2711	
		For other reasons			0.669	0.392	1.144	0.1420	
		For climate and other			0.700	0.311	1.572	0.3875	
Identity (combined; ref = None)	Christian	For climate reasons	Unadjusted	1096	0.814	0.536	1.237	0.3357	0.2168
		For other reasons			1.059	0.674	1.663	0.8047	
		For climate and other			0.709	0.365	1.377	0.3105	
	Christian	For climate reasons	Adjusted	1096	0.783	0.508	1.208	0.2691	0.2146
		For other reasons			1.051	0.659	1.676	0.8356	
		For climate and other			0.805	0.402	1.612	0.5409	
Identity (separate; ref = None)	C of E	For climate reasons	Unadjusted	1096	0.866	0.551	1.362	0.5334	0.3623
		For other reasons			1.176	0.723	1.912	0.5130	
		For climate and other			0.601	0.284	1.275	0.1846	
	Catholic	For climate reasons			0.741	0.310	1.774	0.5012	
		For other reasons			0.885	0.343	2.281	0.8003	
		For climate and other			0.709	0.168	2.983	0.6386	
	Other	For climate reasons			0.667	0.330	1.347	0.2591	
		For other reasons			0.729	0.335	1.583	0.4243	
		For climate and other			1.102	0.398	3.052	0.8514	
	C of E	For climate reasons	Adjusted	1096	0.840	0.527	1.341	0.4663	0.3820
		For other reasons			1.178	0.713	1.945	0.5223	
		For climate and other			0.700	0.321	1.525	0.3689	
	Catholic	For climate reasons			0.637	0.260	1.563	0.3248	
		For other reasons			0.850	0.321	2.251	0.7441	
		For climate and other			0.738	0.168	3.250	0.6884	
	Other	For climate reasons			0.654	0.315	1.356	0.2535	
		For other reasons			0.699	0.314	1.558	0.3810	
		For climate and other			1.200	0.413	3.482	0.7377	
Attendance (ref = Occasional/None)	Regular	For climate reasons	Unadjusted	1109	0.849	0.468	1.539	0.5893	0.1222
		For other reasons			0.687	0.355	1.330	0.2652	

Exposure	Exposure level	Outcome level (ref = No)	Model	*n*	RRR	LCI	UCI	*p*-value	*p* total
		For climate and other			1.735	0.747	4.029	0.2001	
	Regular	For climate reasons	Adjusted	1109	0.768	0.415	1.418	0.3982	0.0805
		For other reasons			0.628	0.318	1.239	0.1799	
		For climate and other			1.733	0.723	4.155	0.2179	
Latent class (ref = "Atheist")	Agnostic	For climate reasons	Unadjusted	1117	0.993	0.594	1.659	0.9784	0.1629
		For other reasons			0.740	0.424	1.292	0.2902	
		For climate and other			0.624	0.258	1.511	0.2959	
	Moderately religious	For climate reasons			0.804	0.443	1.460	0.4734	
		For other reasons			0.795	0.421	1.502	0.4799	
		For climate and other			0.510	0.171	1.522	0.2274	
	Highly religious	For climate reasons			0.870	0.450	1.682	0.6791	
		For other reasons			0.497	0.234	1.055	0.0686	
		For climate and other			1.333	0.521	3.409	0.5485	
	Agnostic	For climate reasons	Adjusted	1117	1.034	0.611	1.750	0.8995	0.1043
		For other reasons			0.734	0.415	1.297	0.2871	
		For climate and other			0.698	0.282	1.729	0.4374	
	Moderately religious	For climate reasons			0.748	0.405	1.383	0.3550	
		For other reasons			0.780	0.405	1.502	0.4577	
		For climate and other			0.577	0.188	1.770	0.3360	
	Highly religious	For climate reasons			0.783	0.396	1.545	0.4799	
		For other reasons			0.437	0.202	0.946	0.0356	
		For climate and other			1.355	0.511	3.599	0.5415	
Reduced the amount of plastic used									
Belief (ref = No)	Not sure	For climate reasons	Unadjusted	1111	0.867	0.590	1.274	0.4669	0.8739
		For other reasons			0.815	0.512	1.298	0.3891	
		For climate and other			0.906	0.416	1.971	0.8025	
	Yes	For climate reasons			1.070	0.733	1.562	0.7272	
		For other reasons			0.845	0.533	1.342	0.4763	
		For climate and other			0.981	0.458	2.101	0.9603	
	Not sure	For climate reasons	Adjusted	1111	0.936	0.628	1.395	0.7455	0.9537
		For other reasons			0.894	0.554	1.442	0.6457	
		For climate and other			1.200	0.530	2.715	0.6622	
	Yes	For climate reasons			1.102	0.740	1.640	0.6337	

Exposure	Exposure level	Outcome level (ref = No)	Model	*n*	RRR	LCI	UCI	*p*-value	*p* total
		For other reasons			0.927	0.572	1.502	0.7588	
		For climate and other			1.228	0.547	2.757	0.6183	
Identity (combined; ref = None)	Christian	For climate reasons	Unadjusted	1100	0.652	0.470	0.905	0.0105	0.0748
		For other reasons			0.712	0.480	1.054	0.0897	
		For climate and other			0.625	0.329	1.186	0.1501	
	Christian	For climate reasons	Adjusted	1100	0.705	0.500	0.994	0.0464	0.2470
		For other reasons			0.796	0.529	1.198	0.2741	
		For climate and other			0.837	0.424	1.652	0.6076	
Identity (separate; ref = None)	C of E	For climate reasons	Unadjusted	1100	0.602	0.426	0.851	0.0040	0.1795
		For other reasons			0.692	0.457	1.047	0.0814	
		For climate and other			0.477	0.232	0.981	0.0443	
	Catholic	For climate reasons			1.080	0.500	2.330	0.8453	
		For other reasons			0.926	0.365	2.354	0.8722	
		For climate and other			1.091	0.271	4.385	0.9024	
	Other	For climate reasons			0.721	0.403	1.291	0.2709	
		For other reasons			0.718	0.352	1.466	0.3630	
		For climate and other			1.206	0.448	3.243	0.7109	
	C of E	For climate reasons	Adjusted	1100	0.651	0.453	0.934	0.0199	0.3808
		For other reasons			0.777	0.505	1.195	0.2499	
		For climate and other			0.632	0.296	1.350	0.2358	
	Catholic	For climate reasons			1.152	0.518	2.562	0.7295	
		For other reasons			1.122	0.429	2.931	0.8142	
		For climate and other			1.799	0.423	7.657	0.4268	
	Other	For climate reasons			0.792	0.432	1.452	0.4512	
		For other reasons			0.746	0.355	1.570	0.4404	
		For climate and other			1.553	0.540	4.466	0.4139	
Attendance (ref = Occasional/None)	Regular	For climate reasons	Unadjusted	1113	1.205	0.738	1.968	0.4567	0.3934
		For other reasons			0.929	0.504	1.714	0.8144	
		For climate and other			1.841	0.791	4.282	0.1566	
	Regular	For climate reasons	Adjusted	1113	1.095	0.658	1.823	0.7275	0.4755
		For other reasons			0.875	0.465	1.646	0.6788	
		For climate and other			1.775	0.736	4.281	0.2018	
Latent class (ref = "Atheist")	Agnostic	For climate reasons	Unadjusted	1121	0.854	0.582	1.254	0.4206	0.2986
		For other reasons			0.753	0.472	1.199	0.2318	

Exposure	Exposure level	Outcome level (ref = No)	Model	*n*	RRR	LCI	UCI	*p*-value	*p* total
		For climate and other			0.825	0.368	1.852	0.6419	
	Moderately religious	For climate reasons			0.814	0.518	1.279	0.3717	
		For other reasons			0.622	0.351	1.103	0.1042	
		For climate and other			0.772	0.291	2.046	0.6029	
	Highly religious	For climate reasons			1.533	0.857	2.740	0.1497	
		For other reasons			0.865	0.417	1.794	0.6964	
		For climate and other			2.188	0.840	5.699	0.1091	
	Agnostic	For climate reasons	Adjusted	1121	0.951	0.639	1.417	0.8063	0.4501
		For other reasons			0.831	0.514	1.341	0.4477	
		For climate and other			1.088	0.468	2.531	0.8439	
	Moderately religious	For climate reasons			0.864	0.539	1.386	0.5454	
		For other reasons			0.685	0.379	1.237	0.2093	
		For climate and other			0.963	0.346	2.676	0.9422	
	Highly religious	For climate reasons			1.535	0.841	2.802	0.1624	
		For other reasons			0.852	0.402	1.803	0.6748	
		For climate and other			2.442	0.896	6.659	0.0810	
Chosen sustainably sourced items									
Belief (ref = No)	Not sure	For climate reasons	Unadjusted	1103	0.955	0.680	1.341	0.7902	0.9804
		For other reasons			0.906	0.594	1.384	0.6492	
		For climate and other			0.898	0.387	2.082	0.8013	
	Yes	For climate reasons			0.910	0.657	1.260	0.5690	
		For other reasons			0.917	0.613	1.372	0.6748	
		For climate and other			1.204	0.574	2.528	0.6229	
	Not sure	For climate reasons	Adjusted	1103	1.032	0.724	1.471	0.8614	0.9302
		For other reasons			1.047	0.675	1.624	0.8366	
		For climate and other			1.094	0.456	2.625	0.8399	
	Yes	For climate reasons			0.951	0.674	1.343	0.7751	
		For other reasons			1.013	0.662	1.551	0.9509	
		For climate and other			1.588	0.724	3.483	0.2488	
Identity (combined; ref = None)	Christian	For climate reasons	Unadjusted	1093	0.651	0.491	0.861	0.0027	0.0212
		For other reasons			0.873	0.616	1.236	0.4433	
		For climate and other			0.772	0.402	1.486	0.4389	
	Christian	For climate reasons	Adjusted	1093	0.711	0.529	0.955	0.0237	0.0660

Exposure	Exposure level	Outcome level (ref = No)	Model	*n*	RRR	LCI	UCI	*p*-value	*p* total
		For other reasons			1.016	0.703	1.467	0.9344	
		For climate and other			1.010	0.508	2.007	0.9783	
Identity (separate; ref = None)	C of E	For climate reasons	Unadjusted	1093	0.623	0.461	0.842	0.0021	0.2341
		For other reasons			0.890	0.615	1.288	0.5368	
		For climate and other			0.763	0.378	1.539	0.4495	
	Catholic	For climate reasons			0.644	0.355	1.168	0.1477	
		For other reasons			0.642	0.291	1.415	0.2721	
		For climate and other			0.628	0.136	2.891	0.5504	
	Other	For climate reasons			0.796	0.481	1.319	0.3765	
		For other reasons			0.953	0.511	1.779	0.8800	
		For climate and other			0.932	0.294	2.949	0.9043	
	C of E	For climate reasons	Adjusted	1093	0.683	0.498	0.938	0.0185	0.3862
		For other reasons			1.064	0.721	1.569	0.7557	
		For climate and other			1.038	0.497	2.165	0.9215	
	Catholic	For climate reasons			0.658	0.351	1.233	0.1913	
		For other reasons			0.789	0.348	1.788	0.5696	
		For climate and other			0.849	0.177	4.073	0.8382	
	Other	For climate reasons			0.891	0.525	1.513	0.6693	
		For other reasons			0.950	0.492	1.832	0.8772	
		For climate and other			0.998	0.296	3.371	0.9980	
Attendance (ref = Occasional/None)	Regular	For climate reasons	Unadjusted	1106	1.616	1.038	2.516	0.0336	0.0580
		For other reasons			1.415	0.822	2.435	0.2101	
		For climate and other			2.700	1.187	6.143	0.0178	
	Regular	For climate reasons	Adjusted	1106	1.486	0.936	2.358	0.0928	0.1122
		For other reasons			1.350	0.769	2.373	0.2963	
		For climate and other			2.757	1.168	6.507	0.0206	
Latent class (ref = "Atheist")	Agnostic	For climate reasons	Unadjusted	1113	0.860	0.613	1.206	0.3831	0.0459
		For other reasons			0.769	0.503	1.175	0.2250	
		For climate and other			1.191	0.543	2.611	0.6628	
	Moderately religious	For climate reasons			0.748	0.499	1.119	0.1579	
		For other reasons			0.736	0.446	1.214	0.2301	
		For climate and other			0.657	0.215	2.010	0.4614	
	Highly religious	For climate reasons			1.975	1.185	3.292	0.0090	
		For other reasons			1.344	0.712	2.534	0.3615	

Exposure	Exposure level	Outcome level (ref = No)	Model	*n*	RRR	LCI	UCI	*p*-value	*p* total
		For climate and other			3.313	1.303	8.424	0.0119	
	Agnostic	For climate reasons	Adjusted	1113	0.956	0.671	1.360	0.8013	0.1210
		For other reasons			0.884	0.570	1.370	0.5813	
		For climate and other			1.489	0.657	3.376	0.3400	
	Moderately religious	For climate reasons			0.814	0.533	1.243	0.3397	
		For other reasons			0.831	0.494	1.400	0.4867	
		For climate and other			0.893	0.282	2.825	0.8476	
	Highly religious	For climate reasons			1.988	1.168	3.383	0.0113	
		For other reasons			1.310	0.678	2.531	0.4210	
		For climate and other			3.759	1.412	10.005	0.0080	
Improved home insulation									
Belief (ref = No)	Not sure	For climate reasons	Unadjusted	1111	1.004	0.683	1.475	0.9843	0.4417
		For other reasons			1.450	1.017	2.068	0.0402	
		For climate and other			0.925	0.392	2.182	0.8593	
	Yes	For climate reasons			1.009	0.703	1.446	0.9626	
		For other reasons			1.223	0.865	1.728	0.2548	
		For climate and other			0.666	0.272	1.628	0.3723	
	Not sure	For climate reasons	Adjusted	1111	1.027	0.691	1.527	0.8934	0.5476
		For other reasons			1.444	1.001	2.083	0.0493	
		For climate and other			0.956	0.393	2.325	0.9215	
	Yes	For climate reasons			0.946	0.648	1.381	0.7736	
		For other reasons			1.136	0.791	1.631	0.4915	
		For climate and other			0.679	0.267	1.728	0.4168	
Identity (combined; ref = None)	Christian	For climate reasons	Unadjusted	1100	1.002	0.736	1.364	0.9887	0.4326
		For other reasons			1.256	0.934	1.689	0.1316	
		For climate and other			0.865	0.420	1.784	0.6953	
	Christian	For climate reasons	Adjusted	1100	1.035	0.749	1.429	0.8368	0.7757
		For other reasons			1.172	0.860	1.597	0.3152	
		For climate and other			0.934	0.440	1.984	0.8593	
Identity (separate; ref = None)	C of E	For climate reasons	Unadjusted	1100	1.029	0.740	1.431	0.8655	0.8448
		For other reasons			1.219	0.887	1.676	0.2218	
		For climate and other			0.802	0.361	1.780	0.5870	
	Catholic	For climate reasons			1.174	0.585	2.357	0.6512	

Exposure	Exposure level	Outcome level (ref = No)	Model	*n*	RRR	LCI	UCI	*p*-value	*p* total
		For other reasons			1.683	0.901	3.147	0.1026	
		For climate and other			1.229	0.267	5.653	0.7911	
	Other	For climate reasons			0.796	0.441	1.438	0.4502	
		For other reasons			1.194	0.710	2.010	0.5033	
		For climate and other			0.956	0.267	3.416	0.9444	
	C of E	For climate reasons	Adjusted	1100	1.061	0.752	1.498	0.7359	0.9717
		For other reasons			1.135	0.815	1.581	0.4545	
		For climate and other			0.894	0.391	2.047	0.7915	
	Catholic	For climate reasons			1.168	0.569	2.396	0.6726	
		For other reasons			1.579	0.823	3.028	0.1690	
		For climate and other			1.209	0.247	5.923	0.8149	
	Other	For climate reasons			0.841	0.455	1.555	0.5808	
		For other reasons			1.128	0.657	1.939	0.6622	
		For climate and other			0.947	0.253	3.546	0.9351	
Attendance (ref = Occasional/None)	Regular	For climate reasons	Unadjusted	1113	1.197	0.763	1.877	0.4339	0.1856
		For other reasons			0.922	0.585	1.453	0.7262	
		For climate and other			2.469	1.067	5.711	0.0347	
	Regular	For climate reasons	Adjusted	1113	1.070	0.669	1.712	0.7777	0.3032
		For other reasons			0.930	0.581	1.487	0.7606	
		For climate and other			2.360	0.979	5.689	0.0559	
Latent class (ref = "Atheist")	Agnostic	For climate reasons	Unadjusted	1121	1.096	0.744	1.616	0.6419	0.0020
		For other reasons			1.450	1.018	2.066	0.0394	
		For climate and other			1.104	0.471	2.587	0.8207	
	Moderately religious	For climate reasons			0.988	0.614	1.589	0.9597	
		For other reasons			1.543	1.022	2.328	0.0390	
		For climate and other			NA	NA	NA	NA	
	Highly religious	For climate reasons			1.251	0.777	2.013	0.3566	
		For other reasons			0.634	0.358	1.122	0.1179	
		For climate and other			1.555	0.598	4.042	0.3656	
	Agnostic	For climate reasons	Adjusted	1121	1.191	0.798	1.777	0.3926	0.0051
		For other reasons			1.470	1.020	2.118	0.0388	
		For climate and other			1.140	0.472	2.756	0.7704	
	Moderately religious	For climate reasons			0.951	0.582	1.553	0.8400	
		For other reasons			1.419	0.926	2.173	0.1080	

Exposure	Exposure level	Outcome level (ref = No)	Model	*n*	RRR	LCI	UCI	*p*-value	*p* total
		For climate and other			NA	NA	NA	NA	
	Highly religious	For climate reasons			1.136	0.689	1.872	0.6172	
		For other reasons			0.594	0.331	1.068	0.0819	
		For climate and other			1.471	0.538	4.020	0.4521	
Installed solar panels									
Belief (ref = No)	Not sure	For climate reasons	Unadjusted	1103	0.653	0.355	1.202	0.1708	0.2421
		For other reasons			0.748	0.339	1.650	0.4720	
		For climate and other			0.731	0.141	3.798	0.7098	
	Yes	For climate reasons			1.313	0.809	2.131	0.2710	
		For other reasons			1.488	0.791	2.801	0.2180	
		For climate and other			1.379	0.367	5.179	0.6345	
	Not sure	For climate reasons	Adjusted	1103	0.720	0.384	1.349	0.3052	0.2914
		For other reasons			0.820	0.360	1.867	0.6358	
		For climate and other			0.817	0.146	4.586	0.8186	
	Yes	For climate reasons			1.434	0.858	2.398	0.1692	
		For other reasons			1.666	0.844	3.292	0.1415	
		For climate and other			1.252	0.285	5.493	0.7658	
Identity (combined; ref = None)	Christian	For climate reasons	Unadjusted	1092	0.869	0.562	1.343	0.5261	0.5095
		For other reasons			1.460	0.800	2.666	0.2180	
		For climate and other			1.357	0.395	4.666	0.6279	
	Christian	For climate reasons	Adjusted	1092	0.997	0.629	1.580	0.9905	0.3884
		For other reasons			1.634	0.862	3.098	0.1326	
		For climate and other			1.790	0.454	7.048	0.4053	
Identity (separate; ref = None)	C of E	For climate reasons	Unadjusted	1092	0.842	0.525	1.351	0.4756	0.8730
		For other reasons			1.408	0.741	2.676	0.2960	
		For climate and other			1.042	0.259	4.193	0.9542	
	Catholic	For climate reasons			0.942	0.357	2.489	0.9043	
		For other reasons			0.933	0.210	4.151	0.9271	
		For climate and other			1.980	0.217	18.065	0.5447	
	Other	For climate reasons			0.945	0.428	2.087	0.8887	
		For other reasons			2.044	0.822	5.085	0.1240	
		For climate and other			2.481	0.447	13.774	0.2989	
	C of E	For climate reasons	Adjusted	1092	0.960	0.584	1.577	0.8715	0.7414

Exposure	Exposure level	Outcome level (ref = No)	Model	*n*	RRR	LCI	UCI	*p*-value	*p* total
		For other reasons			1.529	0.776	3.012	0.2195	
		For climate and other			1.296	0.283	5.940	0.7389	
	Catholic	For climate reasons			1.128	0.410	3.106	0.8158	
		For other reasons			1.351	0.290	6.298	0.7019	
		For climate and other			4.063	0.350	47.225	0.2627	
	Other	For climate reasons			1.098	0.477	2.528	0.8255	
		For other reasons			2.397	0.886	6.483	0.0850	
		For climate and other			4.751	0.634	35.606	0.1294	
Attendance (ref = Occasional/None)	Regular	For climate reasons	Unadjusted	1105	1.759	1.001	3.092	0.0495	0.1361
		For other reasons			0.828	0.322	2.129	0.6953	
		For climate and other			2.795	0.731	10.688	0.1332	
	Regular	For climate reasons	Adjusted	1105	1.547	0.857	2.793	0.1478	0.3173
		For other reasons			0.819	0.302	2.220	0.6941	
		For climate and other			2.580	0.582	11.437	0.2121	
Latent class (ref = "Atheist")	Agnostic	For climate reasons	Unadjusted	1113	0.645	0.349	1.192	0.1614	0.3454
		For other reasons			0.940	0.442	1.999	0.8728	
		For climate and other			0.376	0.045	3.142	0.3666	
	Moderately religious	For climate reasons			1.138	0.609	2.126	0.6843	
		For other reasons			1.826	0.872	3.826	0.1105	
		For climate and other			0.664	0.079	5.565	0.7058	
	Highly religious	For climate reasons			1.381	0.722	2.641	0.3297	
		For other reasons			1.084	0.404	2.910	0.8725	
		For climate and other			2.602	0.640	10.580	0.1815	
	Agnostic	For climate reasons	Adjusted	1113	0.730	0.388	1.375	0.3303	0.4952
		For other reasons			1.059	0.481	2.329	0.8866	
		For climate and other			0.366	0.040	3.389	0.3761	
	Moderately religious	For climate reasons			1.221	0.636	2.345	0.5490	
		For other reasons			2.035	0.930	4.453	0.0755	
		For climate and other			0.527	0.053	5.225	0.5843	
	Highly religious	For climate reasons			1.326	0.670	2.624	0.4172	
		For other reasons			1.139	0.400	3.240	0.8076	
		For climate and other			2.320	0.468	11.490	0.3026	
Started growing vegetables									

Exposure	Exposure level	Outcome level (ref = No)	Model	*n*	RRR	LCI	UCI	*p*-value	*p* total
Belief (ref = No)	Not sure	For climate reasons	Unadjusted	1110	0.833	0.495	1.401	0.4902	0.3130
		For other reasons			0.809	0.576	1.135	0.2202	
		For climate and other			0.217	0.027	1.748	0.1511	
	Yes	For climate reasons			0.992	0.609	1.614	0.9730	
		For other reasons			1.050	0.766	1.438	0.7630	
		For climate and other			0.207	0.026	1.665	0.1385	
	Not sure	For climate reasons	Adjusted	1110	0.814	0.477	1.390	0.4511	0.3559
		For other reasons			0.804	0.565	1.143	0.2242	
		For climate and other			0.434	0.045	4.219	0.4721	
	Yes	For climate reasons			0.914	0.549	1.521	0.7299	
		For other reasons			1.134	0.812	1.584	0.4596	
		For climate and other			0.136	0.009	1.943	0.1414	
Identity (combined; ref = None)	Christian	For climate reasons	Unadjusted	1099	0.781	0.517	1.180	0.2400	0.0502
		For other reasons			0.858	0.654	1.126	0.2685	
		For climate and other			0.178	0.037	0.842	0.0295	
	Christian	For climate reasons	Adjusted	1099	0.734	0.477	1.129	0.1593	0.1920
		For other reasons			0.893	0.671	1.188	0.4365	
		For climate and other			0.220	0.033	1.457	0.1164	
Identity (separate; ref = None)	C of E	For climate reasons	Unadjusted	1099	0.803	0.516	1.249	0.3307	0.3763
		For other reasons			0.839	0.626	1.125	0.2403	
		For climate and other			0.239	0.050	1.136	0.0719	
	Catholic	For climate reasons			0.760	0.285	2.027	0.5832	
		For other reasons			1.038	0.576	1.872	0.9005	
		For climate and other			NA	NA	NA	NA	
	Other	For climate reasons			0.692	0.313	1.530	0.3627	
		For other reasons			0.840	0.513	1.374	0.4868	
		For climate and other			NA	NA	NA	NA	
	C of E	For climate reasons	Adjusted	1099	0.759	0.479	1.202	0.2396	0.4547
		For other reasons			0.861	0.633	1.170	0.3380	
		For climate and other			0.378	0.058	2.456	0.3083	
	Catholic	For climate reasons			0.774	0.282	2.122	0.6182	
		For other reasons			1.224	0.660	2.271	0.5208	
		For climate and other			NA	NA	NA	NA	
	Other	For climate reasons			0.605	0.264	1.382	0.2330	

**Exposure**	**Exposure level**	**Outcome level (ref = No)**	**Model**	***n***	**RRR**	**LCI**	**UCI**	***p*-value**	***p* total**
		For other reasons			0.867	0.517	1.456	0.5900	
		For climate and other			NA	NA	NA	NA	
Attendance (ref = Occasional/None)	Regular	For climate reasons	Unadjusted	1112	1.404	0.784	2.512	0.2537	0.4990
		For other reasons			1.292	0.869	1.920	0.2062	
		For climate and other			0.871	0.109	6.951	0.8964	
	Regular	For climate reasons	Adjusted	1112	1.182	0.646	2.165	0.5872	0.7870
		For other reasons			1.229	0.812	1.860	0.3290	
		For climate and other			1.302	0.121	14.043	0.8279	
Latent class (ref = "Atheist")	Agnostic	For climate reasons	Unadjusted	1120	1.056	0.639	1.743	0.8324	0.1371
		For other reasons			0.899	0.640	1.263	0.5382	
		For climate and other			NA	NA	NA	NA	
	Moderately religious	For climate reasons			0.704	0.346	1.433	0.3332	
		For other reasons			1.180	0.800	1.742	0.4039	
		For climate and other			NA	NA	NA	NA	
	Highly religious	For climate reasons			1.287	0.679	2.438	0.4391	
		For other reasons			0.986	0.626	1.555	0.9531	
		For climate and other			0.562	0.070	4.502	0.5873	
	Agnostic	For climate reasons	Adjusted	1120	1.073	0.640	1.797	0.7902	0.0991
		For other reasons			0.936	0.657	1.334	0.7154	
		For climate and other			NA	NA	NA	NA	
	Moderately religious	For climate reasons			0.696	0.336	1.444	0.3309	
		For other reasons			1.339	0.891	2.012	0.1596	
		For climate and other			NA	NA	NA	NA	
	Highly religious	For climate reasons			1.045	0.536	2.038	0.8974	
		For other reasons			0.951	0.591	1.530	0.8366	
		For climate and other			0.744	0.065	8.454	0.8112	
Planted trees									
Belief (ref = No)	Not sure	For climate reasons	Unadjusted	1100	0.886	0.563	1.397	0.6036	0.1681
		For other reasons			0.726	0.486	1.084	0.1177	
		For climate and other			0.582	0.185	1.830	0.3545	
	Yes	For climate reasons			0.954	0.619	1.470	0.8302	
		For other reasons			0.960	0.669	1.378	0.8250	
		For climate and other			0.132	0.017	1.026	0.0530	

Exposure	Exposure level	Outcome level (ref = No)	Model	*n*	RRR	LCI	UCI	*p*-value	*p* total
	Not sure	For climate reasons	Adjusted	1100	0.919	0.573	1.474	0.7262	0.3987
		For other reasons			0.728	0.483	1.098	0.1299	
		For climate and other			0.643	0.189	2.192	0.4805	
	Yes	For climate reasons			0.976	0.619	1.539	0.9167	
		For other reasons			0.948	0.650	1.384	0.7828	
		For climate and other			0.177	0.022	1.447	0.1062	
Identity (combined; ref = None)	Christian	For climate reasons	Unadjusted	1089	0.738	0.511	1.067	0.1059	0.0765
		For other reasons			0.891	0.649	1.221	0.4718	
		For climate and other			0.354	0.131	0.953	0.0399	
	Christian	For climate reasons	Adjusted	1089	0.753	0.511	1.109	0.1512	0.2349
		For other reasons			0.911	0.656	1.266	0.5799	
		For climate and other			0.433	0.148	1.271	0.1278	
Identity (separate; ref = None)	C of E	For climate reasons	Unadjusted	1089	0.730	0.490	1.086	0.1202	0.3259
		For other reasons			0.880	0.627	1.235	0.4596	
		For climate and other			0.236	0.066	0.845	0.0264	
	Catholic	For climate reasons			0.948	0.423	2.122	0.8966	
		For other reasons			1.244	0.646	2.395	0.5133	
		For climate and other			0.652	0.082	5.149	0.6848	
	Other	For climate reasons			0.660	0.323	1.348	0.2543	
		For other reasons			0.743	0.405	1.361	0.3360	
		For climate and other			0.726	0.159	3.318	0.6798	
	C of E	For climate reasons	Adjusted	1089	0.742	0.489	1.125	0.1601	0.4041
		For other reasons			0.892	0.627	1.268	0.5247	
		For climate and other			0.268	0.070	1.035	0.0562	
	Catholic	For climate reasons			0.960	0.414	2.229	0.9249	
		For other reasons			1.408	0.714	2.776	0.3228	
		For climate and other			1.098	0.126	9.576	0.9323	
	Other	For climate reasons			0.688	0.327	1.447	0.3241	
		For other reasons			0.739	0.394	1.384	0.3441	
		For climate and other			1.185	0.224	6.258	0.8416	
Attendance (ref = Occasional/None)	Regular	For climate reasons	Unadjusted	1102	0.722	0.392	1.330	0.2962	0.6879
		For other reasons			0.998	0.627	1.588	0.9929	
		For climate and other			1.367	0.388	4.809	0.6264	
	Regular	For climate reasons	Adjusted	1102	0.648	0.345	1.217	0.1771	0.3977

**Exposure**	**Exposure level**	**Outcome level (ref = No)**	**Model**	***n***	**RRR**	**LCI**	**UCI**	***p*-value**	***p* total**
		For other reasons			0.904	0.559	1.463	0.6821	
		For climate and other			1.898	0.498	7.238	0.3481	
Latent class (ref = "Atheist")	Agnostic	For climate reasons	Unadjusted	1110	1.078	0.693	1.677	0.7397	0.3399
		For other reasons			0.697	0.463	1.051	0.0853	
		For climate and other			0.547	0.152	1.961	0.3541	
	Moderately religious	For climate reasons			1.046	0.602	1.817	0.8729	
		For other reasons			1.213	0.786	1.873	0.3831	
		For climate and other			0.326	0.042	2.535	0.2840	
	Highly religious	For climate reasons			0.824	0.436	1.556	0.5505	
		For other reasons			0.621	0.350	1.100	0.1025	
		For climate and other			0.750	0.165	3.412	0.7098	
	Agnostic	For climate reasons	Adjusted	1110	1.161	0.733	1.840	0.5238	0.2373
		For other reasons			0.714	0.469	1.088	0.1167	
		For climate and other			0.657	0.172	2.514	0.5399	
	Moderately religious	For climate reasons			1.101	0.620	1.953	0.7433	
		For other reasons			1.265	0.807	1.982	0.3052	
		For climate and other			0.422	0.051	3.499	0.4243	
	Highly religious	For climate reasons			0.765	0.394	1.484	0.4282	
		For other reasons			0.545	0.301	0.985	0.0443	
		For climate and other			1.129	0.228	5.584	0.8821	
Avoided organisations that support fossil fuels									
Belief (ref = No)	Not sure	For climate reasons	Unadjusted	1105	0.531	0.352	0.799	0.0024	0.0013
		For other reasons			0.162	0.038	0.700	0.0148	
		For climate and other			0.811	0.147	4.460	0.8093	
	Yes	For climate reasons			0.597	0.408	0.874	0.0080	
		For other reasons			0.584	0.253	1.346	0.2066	
		For climate and other			0.365	0.041	3.284	0.3685	
	Not sure	For climate reasons	Adjusted	1105	0.545	0.354	0.837	0.0056	0.0030
		For other reasons			0.154	0.035	0.688	0.0143	
		For climate and other			0.339	0.028	4.050	0.3927	
	Yes	For climate reasons			0.612	0.407	0.920	0.0182	
		For other reasons			0.643	0.266	1.559	0.3288	
		For climate and other			0.259	0.023	2.952	0.2763	

**Exposure**	**Exposure level**	**Outcome level (ref = No)**	**Model**	***n***	**RRR**	**LCI**	**UCI**	***p*-value**	***p* total**
Identity (combined; ref = None)	Christian	For climate reasons	Unadjusted	1094	0.424	0.307	0.584	<0.0001	<0.0001
		For other reasons			0.644	0.311	1.334	0.2365	
		For climate and other			0.482	0.107	2.171	0.3422	
	Christian	For climate reasons	Adjusted	1094	0.448	0.318	0.630	<0.0001	<0.0001
		For other reasons			0.719	0.332	1.557	0.4024	
		For climate and other			0.303	0.046	2.012	0.2165	
Identity (separate; ref = None)	C of E	For climate reasons	Unadjusted	1094	0.427	0.301	0.607	<0.0001	0.0002
		For other reasons			0.638	0.289	1.407	0.2652	
		For climate and other			0.435	0.079	2.388	0.3379	
	Catholic	For climate reasons			0.266	0.104	0.681	0.0058	
		For other reasons			0.405	0.052	3.125	0.3858	
		For climate and other			NA	NA	NA	NA	
	Other	For climate reasons			0.515	0.281	0.945	0.0320	
		For other reasons			0.840	0.237	2.969	0.7860	
		For climate and other			1.050	0.116	9.515	0.9657	
	C of E	For climate reasons	Adjusted	1094	0.449	0.310	0.652	<0.0001	0.0011
		For other reasons			0.677	0.294	1.559	0.3597	
		For climate and other			0.167	0.015	1.855	0.1452	
	Catholic	For climate reasons			0.275	0.104	0.730	0.0095	
		For other reasons			0.485	0.057	4.131	0.5081	
		For climate and other			NA	NA	NA	NA	
	Other	For climate reasons			0.567	0.300	1.073	0.0811	
		For other reasons			1.157	0.309	4.337	0.8291	
		For climate and other			1.794	0.140	23.075	0.6537	
Attendance (ref = Occasional/None)	Regular	For climate reasons	Unadjusted	1107	1.197	0.759	1.886	0.4390	0.4093
		For other reasons			1.445	0.542	3.855	0.4623	
		For climate and other			NA	NA	NA	NA	
	Regular	For climate reasons	Adjusted	1107	1.041	0.643	1.686	0.8706	0.5792
		For other reasons			1.730	0.619	4.830	0.2956	
		For climate and other			NA	NA	NA	NA	
Latent class (ref = "Atheist")	Agnostic	For climate reasons	Unadjusted	1115	0.436	0.281	0.677	0.0002	<0.0001
		For other reasons			0.088	0.012	0.661	0.0181	
		For climate and other			0.973	0.177	5.355	0.9752	
		For climate reasons			0.362	0.201	0.649	0.0007	

Exposure	Exposure level	Outcome level (ref = No)	Model	*n*	RRR	LCI	UCI	*p*-value	*p* total
	Moderately religious	For other reasons			0.587	0.199	1.734	0.3352	
		For climate and other			0.807	0.089	7.286	0.8488	
	Highly religious	For climate reasons			1.008	0.624	1.628	0.9751	
		For other reasons			0.661	0.194	2.254	0.5080	
		For climate and other			NA	NA	NA	NA	
	Agnostic	For climate reasons	Adjusted	1115	0.487	0.309	0.769	0.0020	0.0005
		For other reasons			0.089	0.012	0.681	0.0199	
		For climate and other			0.769	0.080	7.378	0.8202	
	Moderately religious	For climate reasons			0.374	0.203	0.688	0.0016	
		For other reasons			0.578	0.189	1.771	0.3373	
		For climate and other			0.515	0.041	6.448	0.6071	
	Highly religious	For climate reasons			0.952	0.569	1.591	0.8500	
		For other reasons			0.731	0.205	2.609	0.6298	
		For climate and other			NA	NA	NA	NA	
Taken action to eat less or no meat and/or dairy									
Belief (ref = No)	Not sure	For climate reasons	Unadjusted	1070	0.734	0.462	1.165	0.1895	0.4714
		For other reasons			0.732	0.517	1.034	0.0770	
		For climate and other			0.681	0.398	1.166	0.1613	
	Yes	For climate reasons			0.892	0.579	1.374	0.6035	
		For other reasons			0.776	0.555	1.086	0.1393	
		For climate and other			0.779	0.469	1.293	0.3341	
	Not sure	For climate reasons	Adjusted	1070	0.827	0.510	1.340	0.4398	0.7233
		For other reasons			0.750	0.522	1.075	0.1174	
		For climate and other			0.703	0.401	1.231	0.2172	
	Yes	For climate reasons			0.938	0.591	1.489	0.7870	
		For other reasons			0.810	0.568	1.156	0.2460	
		For climate and other			0.846	0.495	1.445	0.5400	
Identity (combined; ref = None)	Christian	For climate reasons	Unadjusted	1061	0.491	0.338	0.712	0.0002	0.0001
		For other reasons			0.700	0.525	0.934	0.0152	
		For climate and other			0.500	0.325	0.768	0.0016	
	Christian	For climate reasons	Adjusted	1061	0.546	0.369	0.809	0.0025	0.0056
		For other reasons			0.761	0.563	1.029	0.0764	
		For climate and other			0.567	0.360	0.891	0.0139	

Exposure	Exposure level	Outcome level (ref = No)	Model	*n*	RRR	LCI	UCI	*p*-value	*p* total
Identity (separate; ref = None)	C of E	For climate reasons	Unadjusted	1061	0.469	0.312	0.704	0.0003	0.0007
		For other reasons			0.719	0.530	0.977	0.0347	
		For climate and other			0.462	0.287	0.743	0.0015	
	Catholic	For climate reasons			0.581	0.243	1.390	0.2224	
		For other reasons			1.016	0.555	1.859	0.9598	
		For climate and other			0.364	0.106	1.247	0.1078	
	Other	For climate reasons			0.539	0.278	1.045	0.0672	
		For other reasons			0.462	0.264	0.807	0.0067	
		For climate and other			0.728	0.363	1.461	0.3720	
	C of E	For climate reasons	Adjusted	1061	0.531	0.347	0.814	0.0037	0.0130
		For other reasons			0.769	0.558	1.060	0.1089	
		For climate and other			0.510	0.310	0.839	0.0080	
	Catholic	For climate reasons			0.652	0.261	1.629	0.3600	
		For other reasons			1.170	0.620	2.206	0.6281	
		For climate and other			0.450	0.127	1.595	0.2161	
	Other	For climate reasons			0.557	0.275	1.127	0.1036	
		For other reasons			0.517	0.290	0.924	0.0259	
		For climate and other			0.921	0.439	1.933	0.8273	
Attendance (ref = Occasional/None)	Regular	For climate reasons	Unadjusted	1074	1.953	1.166	3.271	0.0110	0.0082
		For other reasons			1.318	0.848	2.048	0.2190	
		For climate and other			2.376	1.353	4.173	0.0026	
	Regular	For climate reasons	Adjusted	1074	1.588	0.915	2.757	0.1004	0.0364
		For other reasons			1.191	0.752	1.888	0.4557	
		For climate and other			2.359	1.298	4.288	0.0049	
Latent class (ref = "Atheist")	Agnostic	For climate reasons	Unadjusted	1080	0.738	0.460	1.182	0.2064	0.0076
		For other reasons			0.738	0.521	1.047	0.0884	
		For climate and other			0.792	0.466	1.346	0.3888	
	Moderately religious	For climate reasons			0.779	0.439	1.384	0.3947	
		For other reasons			0.960	0.641	1.439	0.8449	
		For climate and other			0.364	0.151	0.878	0.0244	
	Highly religious	For climate reasons			1.888	1.083	3.289	0.0249	
		For other reasons			1.018	0.617	1.679	0.9436	
		For climate and other			1.903	1.021	3.547	0.0429	
	Agnostic	For climate reasons	Adjusted	1080	0.874	0.535	1.430	0.5929	0.0327

**Exposure**	**Exposure level**	**Outcome level (ref = No)**	**Model**	***n***	**RRR**	**LCI**	**UCI**	***p*-value**	***p* total**
		For other reasons			0.802	0.558	1.153	0.2345	
		For climate and other			0.886	0.508	1.546	0.6710	
	Moderately religious	For climate reasons			0.926	0.508	1.690	0.8028	
		For other reasons			1.040	0.680	1.592	0.8562	
		For climate and other			0.377	0.152	0.934	0.0351	
	Highly religious	For climate reasons			1.771	0.975	3.217	0.0606	
		For other reasons			1.017	0.603	1.717	0.9484	
		For climate and other			2.119	1.092	4.111	0.0263	

Table S32: Descriptive statistics of sociodemographic characteristics and other confounders for G1 offspring. Columns display descriptive statistics for both the full sample (n = 14,524) and the complete-case sample with fully-observed data on all confounders, any RSBB exposure data and any climate outcome data (*n* = 1,100). Note that the percentages of missing data are calculated separately from the observed data.

**Variable**	**Full sample – N (%) or mean (SD)**	**Complete-case sample – N (%) or mean (SD)**
Age at completion of climate questions (years)	29.8 (0.63)	29.8 (0.62)
Missing	*10,432 (71.8%)*	*NA*
Assigned sex at birth		
Male	7,467 (51.4%)	321 (29.2%)
Female	7,057 (48.6%)	779 (70.8%)
Missing	*0 (0.0%)*	*NA*
Ethnicity		
White	11,186 (95.1%)	1,070 (97.3%)
Other than White	579 (4.9%)	30 (2.7%)
Missing	*2,759 (19.0%)*	*NA*
Relationship status		
Not in relationship	1,731 (40.6%)	422 (38.4%)
In a relationship	2,529 (59.4%)	678 (61.6%)
Missing	*10,264 (70.7%)*	*NA*
Urban vs rural location		
Urban	9,876 (84.9%)	928 (84.4%)
Rural	1,753 (15.1%)	172 (15.6%)
Missing	*2,895 (19.9%)*	*NA*
Highest educational qualification		
GCSE	454 (13.5%)	91 (8.3%)
A-level	1,300 (38.7%)	434 (39.4%)
Degree	1,069 (31.8%)	395 (35.9%)
Post-graduate degree	538 (16.0%)	180 (16.4%)
Missing	*11,163 (76.9%)*	*NA*
Occupational social class		
Manager/Professional	1,483 (45.3%)	564 (51.3%)
Intermediate	838 (25.6%)	276 (25.1%)
Small employers	45 (1.4%)	16 (1.4%)
Lower supervisory and technical	165 (5.0%)	40 (3.6%)
Routine/Semi-routine	745 (22.7%)	204 (18.6%)
Missing	*11,248 (77.4%)*	*NA*
Household income (per month)		
< £500	294 (8.2%)	58 (5.3%)
£500 - £999	390 (10.8%)	77 (7.0%)
£1000 - £1499	1,217 (3.8%)	380 (34.6%)

£1500 - £1999	1,097 (30.5%)	406 (36.9%)
≥ £2000	600 (16.7%)	179 (16.3%)
Missing	*10,926 (75.2%)*	*NA*
Area-level index of multiple deprivation during pregnancy (quintiles)		
1 (Least deprived)	2,689 (23.8%)	318 (28.9%)
2	2,742 (24.2%)	311 (28.3%)
3	2,021 (17.9%)	230 (20.9%)
4	2,072 (18.3%)	148 (13.4%)
5 (Most deprived)	1,786 (15.8%)	93 (8.5%)
Missing	*3,214 (22.1%)*	*NA*
Home ownership status		
Owned/Mortgaged	2,353 (55.4%)	700 (63.6%)
Private rented	1,461 (34.4%)	337 (30.6%)
Council/Housing association	252 (5.9%)	28 (2.6%)
Other	178 (4.2%)	35 (3.2%)
Missing	*10,280 (70.8%)*	*NA*

Table S33: Results of the offspring ordinal regression models with 'belief that the climate is changing' as the outcome for four religious exposures (belief, identity [both Christian denominations combined together and separate], attendance, and latent classes). Odds ratios above 1 indicate an increased belief in climate change. The 'brant' column shows the *p*-value of the brant test for said variable. The '*p* total' column is the *p*-value for the overall association between the exposure and outcome (if three or more exposure levels). OR = Odds ratio; LCI = Lower 95% confidence interval; UCI = Upper 95% confidence interval.

**Exposure**	**Exposure level**	**Model**	***n***	**OR**	**LCI**	**UCI**	***p*-value**	**brant**	***p* total**
Belief (ref = No)	Not sure	Unadjusted	1100	1.084	0.744	1.581	0.6732	0.6481	0.9120
	Yes			1.007	0.639	1.588	0.9750	0.8409	
	Not sure	Adjusted	1100	1.051	0.706	1.565	0.8071	0.6605	0.9682
	Yes			1.033	0.639	1.668	0.8959	0.7446	
	Not sure	Adjusted (politics)	1100	1.086	0.727	1.622	0.6886	0.6310	0.9186
	Yes			1.049	0.647	1.702	0.8449	0.8769	
Identity (combined; ref = None)	Christian	Unadjusted	1099	0.773	0.552	1.081	0.1322	0.7014	NA
	Christian	Adjusted	1099	0.823	0.578	1.172	0.2803	0.5949	NA
	Christian	Adjusted (politics)	1099	0.857	0.599	1.225	0.3960	0.5913	NA
Identity (separate; ref = None)	C of E	Unadjusted	1099	0.628	0.436	0.904	0.0123	0.9206	0.0158
	Catholic			0.801	0.346	1.856	0.6051	0.0888	
	Other			2.253	0.888	5.718	0.0873	0.1560	
	C of E	Adjusted	1099	0.684	0.463	1.010	0.0558	0.9323	0.0952
	Catholic			0.928	0.387	2.224	0.8668	0.2343	
	Other			2.031	0.776	5.316	0.1492	0.1337	
	C of E	Adjusted (politics)	1099	0.708	0.478	1.049	0.0855	0.9317	0.1169
	Catholic			1.003	0.415	2.421	0.9948	0.3944	
	Other			2.110	0.802	5.547	0.1301	0.1471	
Attendance (ref = Occasional/None)	Regular	Unadjusted	1091	1.765	0.748	4.165	0.1946	0.9616	NA
	Regular	Adjusted	1091	1.561	0.631	3.863	0.3357	0.9635	NA
	Regular	Adjusted (politics)	1091	1.552	0.623	3.866	0.3453	1.0000	NA
Latent class (ref = "Atheist")	Agnostic	Unadjusted	1048	1.586	1.013	2.483	0.0439	<0.0001	0.1070
	Moderately religious			1.271	0.684	2.362	0.4486	0.8586	
	Highly religious			1.317	0.693	2.500	0.4005	0.2708	
	Agnostic	Adjusted	1048	1.554	0.965	2.501	0.0698	0.9866	0.1771
	Moderately religious			1.371	0.709	2.652	0.3488	0.9299	
	Highly religious			1.217	0.615	2.406	0.5730	0.9812	
	Agnostic	Adjusted (politics)	1048	1.604	0.992	2.594	0.0541	1.0000	0.2347

	Moderately religious			1.385	0.716	2.681	0.3332	0.9231	
	Highly religious			1.191	0.599	2.366	0.6181	0.9897	

Table S34: Results of the offspring ordinal regression models with 'concerned about the impact of climate change' as the outcome for four religious exposures (belief, identity [both Christian denominations combined together and separate], attendance, and latent classes). Odds ratios above 1 indicate an increased concern regarding climate change. The 'brant' column shows the *p*-value of the brant test for said variable. The '*p* total' column is the *p*-value for the overall association between the exposure and outcome (if three or more exposure levels). OR = Odds ratio; LCI = Lower 95% confidence interval; UCI = Upper 95% confidence interval.

Exposure	Exposure level	Model	*n*	OR	LCI	UCI	*p*-value	brant	*p* total
Belief (ref = No)	Not sure	Unadjusted	1096	1.093	0.840	1.423	0.5071	0.0266	0.6916
	Yes			0.942	0.675	1.314	0.7233	0.5016	
	Not sure	Adjusted	1096	1.095	0.831	1.442	0.5191	0.0384	0.6702
	Yes			0.928	0.661	1.303	0.6650	0.6058	
	Not sure	Adjusted (politics)	1096	1.167	0.883	1.542	0.2773	0.0497	0.5162
	Yes			0.987	0.700	1.391	0.9391	0.4975	
Identity (combined; ref = None)	Christian	Unadjusted	1095	0.780	0.609	1.000	0.0500	0.2705	NA
	Christian	Adjusted	1095	0.815	0.631	1.053	0.1182	0.2984	NA
	Christian	Adjusted (politics)	1095	0.874	0.674	1.132	0.3068	0.2142	NA
Identity (separate; ref = None)	C of E	Unadjusted	1095	0.692	0.520	0.921	0.0116	0.4267	0.0627
	Catholic			1.250	0.668	2.337	0.4855	0.9060	
	Other			0.894	0.554	1.440	0.6441	0.5123	
	C of E	Adjusted	1095	0.760	0.564	1.023	0.0703	0.5663	0.1425
	Catholic			1.378	0.720	2.638	0.3325	0.9494	
	Other			0.765	0.468	1.249	0.2840	0.4359	
	C of E	Adjusted (politics)	1095	0.819	0.607	1.107	0.1937	0.4448	0.2433
	Catholic			1.497	0.778	2.880	0.2264	0.9480	
	Other			0.797	0.486	1.307	0.3686	0.3650	
Attendance (ref = Occasional/None)	Regular	Unadjusted	1088	1.249	0.748	2.086	0.3960	0.6450	NA
	Regular	Adjusted	1088	1.123	0.660	1.910	0.6697	0.4613	NA
	Regular	Adjusted (politics)	1088	1.174	0.687	2.006	0.5564	0.3820	NA
Latent class (ref = "Atheist")	Agnostic	Unadjusted	1045	1.138	0.851	1.521	0.3840	0.0808	0.8241
	Moderately religious			1.110	0.718	1.716	0.6389	0.6139	
	Highly religious			1.091	0.699	1.702	0.7024	0.3824	
	Agnostic	Adjusted	1045	1.079	0.795	1.463	0.6260	0.0709	0.9554
	Moderately religious			1.059	0.677	1.656	0.8019	0.9179	
	Highly religious			1.080	0.681	1.712	0.7424	0.2495	
	Agnostic	Adjusted (politics)	1045	1.142	0.840	1.553	0.3969	0.0743	0.8383

	Moderately religious			1.095	0.697	1.720	0.6936	0.9073	
	Highly religious			1.103	0.693	1.755	0.6805	0.2275	

Table S35: Results of the offspring ordinal regression models with 'believes that humans are to blame for climate change' as the outcome for four religious exposures (belief, identity [both Christian denominations combined together and separate], attendance, and latent classes). Odds ratios above 1 indicate an increased belief that humans are to blame for climate change. The 'brant' column shows the *p*-value of the brant test for said variable. The '*p* total' column is the *p*-value for the overall association between the exposure and outcome (if three or more exposure levels). OR = Odds ratio; LCI = Lower 95% confidence interval; UCI = Upper 95% confidence interval.

**Exposure**	**Exposure level**	**Model**	***n***	**OR**	**LCI**	**UCI**	***p*-value**	**brant**	***p* total**
Belief (ref = No)	Not sure	Unadjusted	1095	0.836	0.643	1.086	0.1795	0.2093	0.1063
	Yes			0.726	0.525	1.004	0.0526	0.7891	
	Not sure	Adjusted	1095	0.814	0.622	1.065	0.1331	1.0000	0.0993
	Yes			0.730	0.525	1.014	0.0603	1.0000	
	Not sure	Adjusted (politics)	1095	0.846	0.646	1.109	0.2255	1.0000	0.1915
	Yes			0.760	0.546	1.057	0.1030	1.0000	
Identity (combined; ref = None)	Christian	Unadjusted	1094	0.652	0.510	0.832	0.0006	0.9112	NA
	Christian	Adjusted	1094	0.679	0.530	0.871	0.0023	1.0000	NA
	Christian	Adjusted (politics)	1094	0.711	0.554	0.914	0.0077	1.0000	NA
Identity (separate; ref = None)	C of E	Unadjusted	1094	0.583	0.439	0.773	0.0002	0.7354	0.0028
	Catholic			0.863	0.470	1.587	0.6355	0.9514	
	Other			0.792	0.503	1.248	0.3153	0.3295	
	C of E	Adjusted	1094	0.638	0.477	0.853	0.0024	1.0000	0.0148
	Catholic			0.933	0.501	1.737	0.8262	1.0000	
	Other			0.696	0.437	1.109	0.1273	1.0000	
	C of E	Adjusted (politics)	1094	0.668	0.499	0.895	0.0069	1.0000	0.0390
	Catholic			0.981	0.526	1.829	0.9508	0.8574	
	Other			0.724	0.454	1.155	0.1753	1.0000	
Attendance (ref = Occasional/None)	Regular	Unadjusted	1087	1.017	0.625	1.655	0.9454	0.4248	NA
	Regular	Adjusted	1087	0.910	0.551	1.502	0.7113	0.5485	NA
	Regular	Adjusted (politics)	1087	0.948	0.572	1.572	0.8371	0.6293	NA
Latent class (ref = "Atheist")	Agnostic	Unadjusted	1045	0.934	0.700	1.245	0.6404	0.3056	0.2754
	Moderately religious			1.356	0.886	2.077	0.1612	0.9999	
	Highly religious			0.785	0.512	1.205	0.2685	0.5637	
	Agnostic	Adjusted	1045	0.871	0.648	1.172	0.3633	1.0000	0.2096
	Moderately religious			1.343	0.866	2.081	0.1878	1.0000	
	Highly religious			0.759	0.490	1.176	0.2177	1.0000	
	Agnostic	Adjusted (politics)	1045	0.901	0.669	1.215	0.4949	1.0000	0.2316

	Moderately religious			1.371	0.882	2.130	0.1607	1.0000	
	Highly religious			0.766	0.493	1.190	0.2350	1.0000	

Table S36: Results of the offspring multinomial regression models with 'thinks that personal actions will make a difference to long-term climate change' as the outcome for four religious exposures (belief, identity [both Christian denominations combined together and separate], attendance, and latent classes). The '*p* total' column is the *p*-value for the overall association between the exposure and outcome. RRR = Relative risk ratio; LCI = Lower 95% confidence interval; UCI = Upper 95% confidence interval.

**Exposure**	**Exposure level**	**Outcome level (ref = No)**	**Model**	***n***	**RRR**	**LCI**	**UCI**	***p*-value**	***p* total**
Belief (ref = No)	Not sure	Not sure	Unadjusted	1095	2.089	1.347	3.238	0.0010	0.0064
		Yes			1.729	1.157	2.583	0.0075	
	Yes	Not sure			1.734	1.008	2.981	0.0466	
		Yes			1.689	1.037	2.752	0.0353	
	Not sure	Not sure	Adjusted	1095	2.117	1.348	3.324	0.0011	0.0083
		Yes			1.618	1.071	2.446	0.0224	
	Yes	Not sure			1.687	0.968	2.941	0.0650	
		Yes			1.683	1.020	2.776	0.0416	
	Not sure	Not sure	Adjusted (politics)	1095	2.143	1.361	3.373	0.0010	0.0087
		Yes			1.596	1.054	2.417	0.0273	
	Yes	Not sure			1.701	0.972	2.976	0.0626	
		Yes			1.645	0.995	2.722	0.0525	
Identity (combined; ref = None)	Christian	Not sure	Unadjusted	1094	1.064	0.713	1.587	0.7609	0.0734
		Yes			1.398	0.982	1.989	0.0629	
	Christian	Not sure	Adjusted	1094	1.038	0.688	1.564	0.8604	0.0814
		Yes			1.382	0.961	1.989	0.0810	
	Christian	Not sure	Adjusted (politics)	1094	1.041	0.687	1.577	0.8501	0.1159
		Yes			1.358	0.940	1.962	0.1025	
Identity (separate; ref = None)	C of E	Not sure	Unadjusted	1094	1.188	0.741	1.907	0.4741	0.1251
		Yes			1.577	1.037	2.399	0.0332	
	Catholic	Not sure			1.132	0.458	2.800	0.7883	
		Yes			0.844	0.359	1.982	0.6966	
	Other	Not sure			0.697	0.314	1.546	0.3742	
		Yes			1.269	0.665	2.424	0.4702	
	C of E	Not sure	Adjusted	1094	1.199	0.735	1.956	0.4678	0.1424
		Yes			1.593	1.030	2.463	0.0363	
	Catholic	Not sure			1.056	0.415	2.691	0.9084	
		Yes			0.842	0.347	2.045	0.7041	
	Other	Not sure			0.635	0.281	1.434	0.2742	

		Yes			1.180	0.608	2.290	0.6247	
	C of E	Not sure	Adjusted (politics)	1094	1.201	0.733	1.968	0.4679	0.1870
		Yes			1.560	1.005	2.422	0.0475	
	Catholic	Not sure			1.078	0.422	2.752	0.8749	
		Yes			0.847	0.348	2.063	0.7151	
	Other	Not sure			0.636	0.281	1.439	0.2772	
		Yes			1.160	0.597	2.253	0.6622	
Attendance (ref = Occasional/None)	Regular	Not sure	Unadjusted	1087	0.955	0.416	2.195	0.9138	0.7371
		Yes			1.200	0.583	2.470	0.6202	
	Regular	Not sure	Adjusted	1087	0.881	0.373	2.079	0.7723	0.6142
		Yes			1.205	0.573	2.537	0.6228	
	Regular	Not sure	Adjusted (politics)	1087	0.868	0.366	2.055	0.7457	0.6807
		Yes			1.153	0.546	2.436	0.7069	
Latent class (ref = "Atheist")	Agnostic	Not sure	Unadjusted	1045	1.764	1.091	2.850	0.0205	0.0428
		Yes			1.696	1.092	2.632	0.0186	
	Moderately religious	Not sure			1.877	0.865	4.073	0.1112	
		Yes			2.331	1.156	4.703	0.0181	
	Highly religious	Not sure			1.318	0.638	2.726	0.4559	
		Yes			1.630	0.859	3.095	0.1350	
	Agnostic	Not sure	Adjusted	1045	1.754	1.069	2.879	0.0261	0.0772
		Yes			1.579	1.002	2.488	0.0490	
	Moderately religious	Not sure			1.684	0.760	3.734	0.1995	
		Yes			2.190	1.065	4.506	0.0332	
	Highly religious	Not sure			1.318	0.625	2.782	0.4680	
		Yes			1.647	0.852	3.185	0.1379	
	Agnostic	Not sure	Adjusted (politics)	1045	1.765	1.072	2.905	0.0255	0.0850
		Yes			1.559	0.987	2.461	0.0568	
	Moderately religious	Not sure			1.697	0.765	3.764	0.1932	
		Yes			2.196	1.067	4.519	0.0327	
	Highly religious	Not sure			1.299	0.613	2.754	0.4951	
		Yes			1.592	0.820	3.091	0.1692	

Table S37: Results of the offspring linear regression models with 'total number of actions performed due to climate change' as the outcome for four religious exposures (belief, identity [both Christian denominations combined together and separate], attendance, and latent classes). Values above 0 indicate an increased number of pro-environmental actions performed. The '*p* total' column is the *p*-value for the overall association between the exposure and outcome (if three or more exposure levels). *b* = Mean difference; LCI = Lower 95% confidence interval; UCI = Upper 95% confidence interval.

**Exposure**	**Exposure level**	**Model**	***n***	***b***	**LCI**	**UCI**	***p*-value**	***p* total**
Belief (ref = No)	Not sure	Unadjusted	983	0.035	-0.478	0.547	0.8942	0.8746
	Yes			-0.145	-0.779	0.489	0.6537	
	Not sure	Adjusted	983	-0.037	-0.529	0.454	0.8820	0.8193
	Yes			-0.194	-0.798	0.409	0.5280	
	Not sure	Adjusted (politics)	983	0.045	-0.444	0.535	0.8558	0.8654
	Yes			-0.135	-0.737	0.467	0.6594	
Identity (combined; ref = None)	Christian	Unadjusted	982	-0.534	-1.008	-0.060	0.0272	NA
	Christian	Adjusted	982	-0.501	-0.954	-0.049	0.0299	NA
	Christian	Adjusted (politics)	982	-0.400	-0.852	0.052	0.0831	NA
Identity (separate; ref = None)	C of E	Unadjusted	982	-0.735	-1.283	-0.188	0.0085	0.0410
	Catholic			-0.676	-1.868	0.517	0.2664	
	Other			0.169	-0.718	1.056	0.7089	
	C of E	Adjusted	982	-0.682	-1.210	-0.155	0.0113	0.0835
	Catholic			-0.377	-1.515	0.760	0.5152	
	Other			-0.023	-0.871	0.826	0.9585	
	C of E	Adjusted (politics)	982	-0.582	-1.109	-0.056	0.0302	0.1765
	Catholic			-0.239	-1.369	0.890	0.6776	
	Other			0.063	-0.780	0.906	0.8834	
Attendance (ref = Occasional/None)	Regular	Unadjusted	975	0.124	-0.859	1.106	0.8052	NA
	Regular	Adjusted	975	0.027	-0.915	0.970	0.9546	NA
	Regular	Adjusted (politics)	975	0.072	-0.864	1.009	0.8793	NA
Latent class (ref = "Atheist")	Agnostic	Unadjusted	940	-0.075	-0.637	0.486	0.7921	0.0013
	Moderately religious			0.285	-0.549	1.119	0.5027	
	Highly religious			0.038	-0.796	0.872	0.9283	
	Agnostic	Adjusted	940	-0.196	-0.735	0.343	0.4761	0.0017
	Moderately religious			0.157	-0.640	0.954	0.6987	
	Highly religious			-0.051	-0.845	0.743	0.9002	
	Agnostic	Adjusted (politics)	940	-0.120	-0.657	0.416	0.6602	
	Moderately religious			0.210	-0.579	1.000	0.6010	
	Highly religious			-0.048	-0.839	0.742	0.9045	

Table S38: Results of the offspring Poisson regression models with ‘total number of actions performed due to climate change’ as the outcome for four religious exposures (belief, identity [both Christian denominations combined together and separate], attendance, and latent classes). Incidence rate ratios above 1 indicate an increased number of pro-environmental actions performed. The ‘*p* total’ column is the *p*-value for the overall association between the exposure and outcome (if three or more exposure levels). IRR = Incidence rate ratio; LCI = Lower 95% confidence interval; UCI = Upper 95% confidence interval.

**Exposure**	**Exposure level**	**Model**	***n***	**IRR**	**LCI**	**UCI**	***p*-value**	***p* total**
Belief (ref = No)	Not sure	Unadjusted	983	1.007	0.945	1.073	0.8376	0.7267
	Yes			0.972	0.898	1.053	0.4878	
	Not sure	Adjusted	983	0.994	0.932	1.060	0.8507	0.6486
	Yes			0.963	0.888	1.043	0.3525	
	Not sure	Adjusted (politics)	983	1.009	0.945	1.076	0.7937	0.6830
	Yes			0.970	0.895	1.052	0.4607	
Identity (combined; ref = None)	Christian	Unadjusted	982	0.900	0.848	0.956	0.0007	NA
	Christian	Adjusted	982	0.905	0.852	0.962	0.0014	NA
	Christian	Adjusted (politics)	982	0.921	0.867	0.980	0.0090	NA
Identity (separate; ref = None)	C of E	Unadjusted	982	0.863	0.803	0.927	0.0001	0.0002
	Catholic			0.874	0.747	1.022	0.0915	
	Other			1.031	0.926	1.149	0.5724	
	C of E	Adjusted	982	0.870	0.810	0.936	0.0002	0.0023
	Catholic			0.929	0.793	1.087	0.3584	
	Other			0.996	0.893	1.110	0.9415	
	C of E	Adjusted (politics)	982	0.886	0.824	0.953	0.0011	0.0117
	Catholic			0.951	0.812	1.113	0.5310	
	Other			1.009	0.905	1.125	0.8671	
Attendance (ref = Occasional/None)	Regular	Unadjusted	975	1.024	0.907	1.156	0.7037	NA
	Regular	Adjusted	975	1.004	0.888	1.136	0.9477	NA
	Regular	Adjusted (politics)	975	1.011	0.894	1.145	0.8579	NA
Latent class (ref = “Atheist”)	Agnostic	Unadjusted	940	0.985	0.918	1.058	0.6837	0.6981
	Moderately religious			1.055	0.953	1.169	0.3028	
	Highly religious			1.007	0.908	1.118	0.8896	
	Agnostic	Adjusted	940	0.959	0.893	1.031	0.2588	0.6225
	Moderately religious			1.027	0.926	1.139	0.6194	
	Highly religious			0.989	0.890	1.099	0.8387	
	Agnostic	Adjusted (politics)	940	0.973	0.905	1.046	0.4543	0.7526

	Moderately religious			1.035	0.933	1.148	0.5190	
	Highly religious			0.987	0.888	1.098	0.8150	

Table S39: Results of the offspring zero-inflated Poisson regression models with 'total number of actions performed due to climate change' as the outcome for four religious exposures (belief, identity [both Christian denominations combined together and separate], attendance, and latent classes). Incidence rate ratios above 1 indicate an increased number of pro-environmental actions performed, while odds ratios above 1 indicate an increased probability of excess zeros. IRR = Incidence rate ratio; LCI = Lower 95% confidence interval; UCI = Upper 95% confidence interval; OR = Odds ratio.

**Exposure**	**Exposure level**	**Model**	***n***	**IRR**	**IRR LCI**	**IRR UCI**	**IRR *p***	**OR**	**OR LCI**	**OR UCI**	**OR *p***
Belief (ref = No)	Not sure	Unadjusted	983	0.976	0.915	1.040	0.4539	0.793	0.512	1.230	0.3005
	Yes			0.983	0.907	1.066	0.6810	1.077	0.653	1.776	0.7707
	Not sure	Adjusted	983	0.970	0.909	1.036	0.3624	0.835	0.522	1.337	0.4525
	Yes			0.978	0.902	1.061	0.5897	1.108	0.646	1.900	0.7090
	Not sure	Adjusted (politics)	983	0.981	0.919	1.048	0.5689	0.814	0.506	1.310	0.3968
	Yes			0.987	0.909	1.071	0.7505	1.105	0.639	1.913	0.7206
Identity (combined; ref = None)	Christian	Unadjusted	982	0.917	0.863	0.975	0.0056	1.137	0.775	1.669	0.5107
	Christian	Adjusted	982	0.919	0.864	0.978	0.0075	1.133	0.749	1.714	0.5556
	Christian	Adjusted (politics)	982	0.933	0.877	0.993	0.0298	1.089	0.714	1.662	0.6907
Identity (separate; ref = None)	C of E	Unadjusted	982	0.898	0.836	0.966	0.0036	1.299	0.847	1.993	0.2299
	Catholic			0.906	0.774	1.062	0.2233	1.270	0.505	3.193	0.6108
	Other			0.976	0.876	1.088	0.6648	0.611	0.252	1.484	0.2766
	C of E	Adjusted	982	0.905	0.841	0.974	0.0076	1.063	0.996	1.135	0.2592
	Catholic			0.932	0.795	1.094	0.3894	0.907	0.754	1.091	0.8665
	Other			0.952	0.853	1.063	0.3822	1.055	0.993	1.121	0.4010
	C of E	Adjusted (politics)	982	0.920	0.854	0.991	0.0271	1.042	0.975	1.113	0.3269
	Catholic			0.948	0.807	1.112	0.5102	0.907	0.754	1.092	0.9893
	Other			0.963	0.862	1.075	0.4970	1.058	0.996	1.124	0.3716
Attendance (ref = Occasional/None)	Regular	Unadjusted	975	1.036	0.917	1.171	0.5690	1.084	0.495	2.375	0.8394
	Regular	Adjusted	975	1.024	0.904	1.159	0.7145	1.232	0.536	2.833	0.6238
	Regular	Adjusted (politics)	975	1.034	0.913	1.172	0.5951	1.258	0.542	2.920	0.5930
Latent class (ref = "Atheist")	Agnostic	Unadjusted	940	0.945	0.880	1.015	0.1179	0.737	0.455	1.196	0.2167
	Moderately religious			1.005	0.906	1.113	0.9305	0.696	0.331	1.460	0.3373
	Highly religious			0.988	0.890	1.097	0.8212	0.878	0.442	1.741	0.7086
	Agnostic	Adjusted	940	0.937	0.871	1.008	0.0805	0.778	0.462	1.311	0.3455
	Moderately religious			0.980	0.883	1.088	0.7063	0.702	0.312	1.580	0.3931
	Highly religious			0.990	0.890	1.102	0.8597	0.932	0.448	1.937	0.8497
	Agnostic	Adjusted (politics)	940	0.947	0.880	1.020	0.1497	0.754	0.444	1.280	0.2961
	Moderately religious			0.988	0.890	1.097	0.8211	0.683	0.302	1.545	0.3598
	Highly religious			0.992	0.891	1.105	0.8861	0.955	0.456	2.001	0.9032

Table S40: Results of the offspring linear regression models with 'total number of actions performed due to climate change (excluding ones which may be prohibitively costly)' as the outcome for four religious exposures (belief, identity [both Christian denominations combined together and separate], attendance, and latent classes). Values above 0 indicate an increased number of pro-environmental actions performed. The '*p* total' column is the *p*-value for the overall association between the exposure and outcome (if three or more exposure levels). *b* = Mean difference; LCI = Lower 95% confidence interval; UCI = Upper 95% confidence interval.

**Exposure**	**Exposure level**	**Model**	***n***	***b***	**LCI**	**UCI**	***p*-value**	***p* total**
Belief (ref = No)	Not sure	Unadjusted	995	0.053	-0.407	0.514	0.8208	0.8442
	Yes			-0.133	-0.702	0.435	0.6461	
	Not sure	Adjusted	995	-0.035	-0.475	0.405	0.8756	0.7929
	Yes			-0.187	-0.726	0.352	0.4960	
	Not sure	Adjusted (politics)	995	0.028	-0.410	0.466	0.9006	0.8462
	Yes			-0.140	-0.678	0.398	0.6088	
Identity (combined; ref = None)	Christian	Unadjusted	994	-0.501	-0.926	-0.076	0.0209	NA
	Christian	Adjusted	994	-0.469	-0.873	-0.065	0.0230	NA
	Christian	Adjusted (politics)	994	-0.386	-0.790	0.018	0.0613	NA
Identity (separate; ref = None)	C of E	Unadjusted	994	-0.658	-1.150	-0.167	0.0087	0.0520
	Catholic			-0.574	-1.651	0.503	0.2955	
	Other			0.027	-0.769	0.822	0.9471	
	C of E	Adjusted	994	-0.590	-1.061	-0.120	0.0140	0.1034
	Catholic			-0.307	-1.329	0.715	0.5556	
	Other			-0.186	-0.944	0.572	0.6309	
	C of E	Adjusted (politics)	994	-0.508	-0.979	-0.037	0.0345	0.2127
	Catholic			-0.192	-1.209	0.824	0.7104	
	Other			-0.121	-0.874	0.633	0.7537	
Attendance (ref = Occasional/None)	Regular	Unadjusted	987	0.066	-0.806	0.938	0.8819	NA
	Regular	Adjusted	987	-0.003	-0.835	0.829	0.9946	NA
	Regular	Adjusted (politics)	987	0.033	-0.795	0.862	0.9374	NA
Latent class (ref = "Atheist")	Agnostic	Unadjusted	952	0.031	-0.475	0.537	0.9049	0.8402
	Moderately religious			0.348	-0.406	1.103	0.3652	
	Highly religious			-0.010	-0.756	0.736	0.9791	
	Agnostic	Adjusted	952	-0.101	-0.585	0.382	0.6812	0.8950
	Moderately religious			0.201	-0.516	0.919	0.5823	
	Highly religious			-0.081	-0.788	0.626	0.8217	
	Agnostic	Adjusted (politics)	952	-0.043	-0.525	0.438	0.8603	0.8927

	Moderately religious			0.248	-0.464	0.960	0.4949	
	Highly religious			-0.079	-0.784	0.626	0.8254	

Table S41: Results of the offspring Poisson regression models with 'total number of actions performed due to climate change (excluding ones which may be prohibitively costly)' as the outcome for four religious exposures (belief, identity [both Christian denominations combined together and separate], attendance, and latent classes). Incidence rate ratios above 1 indicate an increased number of pro-environmental actions performed. The '*p* total' column is the *p*-value for the overall association between the exposure and outcome (if three or more exposure levels). IRR = Incidence rate ratio; LCI = Lower 95% confidence interval; UCI = Upper 95% confidence interval.

**Exposure**	**Exposure level**	**Model**	***n***	**IRR**	**LCI**	**UCI**	***p*-value**	***p* total**
Belief (ref = No)	Not sure	Unadjusted	995	1.011	0.947	1.079	0.7443	0.7029
	Yes			0.973	0.896	1.056	0.5065	
	Not sure	Adjusted	995	0.994	0.930	1.062	0.8526	0.6392
	Yes			0.961	0.885	1.044	0.3447	
	Not sure	Adjusted (politics)	995	1.006	0.940	1.075	0.8711	0.6809
	Yes			0.967	0.890	1.051	0.4288	
Identity (combined; ref = None)	Christian	Unadjusted	994	0.900	0.846	0.958	0.0009	NA
	Christian	Adjusted	994	0.905	0.850	0.963	0.0017	NA
	Christian	Adjusted (politics)	994	0.918	0.862	0.978	0.0083	NA
Identity (separate; ref = None)	C of E	Unadjusted	994	0.868	0.807	0.934	0.0002	0.0012
	Catholic			0.885	0.754	1.040	0.1376	
	Other			1.005	0.899	1.124	0.9251	
	C of E	Adjusted	994	0.879	0.816	0.947	0.0007	0.0083
	Catholic			0.937	0.797	1.102	0.4341	
	Other			0.963	0.860	1.078	0.5131	
	C of E	Adjusted (politics)	994	0.893	0.828	0.962	0.0030	0.0312
	Catholic			0.957	0.814	1.126	0.5978	
	Other			0.973	0.869	1.090	0.6369	
Attendance (ref = Occasional/None)	Regular	Unadjusted	987	1.014	0.896	1.147	0.8303	NA
	Regular	Adjusted	987	0.998	0.880	1.132	0.9791	NA
	Regular	Adjusted (politics)	987	1.004	0.885	1.139	0.9533	NA
Latent class (ref = "Atheist")	Agnostic	Unadjusted	952	1.006	0.936	1.082	0.8627	0.6272
	Moderately religious			1.072	0.966	1.191	0.1916	
	Highly religious			0.998	0.897	1.111	0.9697	
	Agnostic	Adjusted	952	0.976	0.906	1.051	0.5139	0.7706
	Moderately religious			1.037	0.932	1.153	0.5074	
	Highly religious			0.982	0.881	1.094	0.7435	
	Agnostic	Adjusted (politics)	952	0.987	0.916	1.063	0.7223	0.7914

	Moderately religious			1.045	0.939	1.162	0.4208	
	Highly religious			0.980	0.879	1.093	0.7183	

Table S42: Results of the offspring zero-inflated Poisson regression models with 'total number of actions performed due to climate change (excluding ones which may be prohibitively costly)' as the outcome for four religious exposures (belief, identity [both Christian denominations combined together and separate], attendance, and latent classes). Incidence rate ratios above 1 indicate an increased number of pro-environmental actions performed, while odds ratios above 1 indicate an increased probability of excess zeros. IRR = Incidence rate ratio; LCI = Lower 95% confidence interval; UCI = Upper 95% confidence interval; OR = Odds ratio.

**Exposure**	**Exposure level**	**Model**	***n***	**IRR**	**IRR LCI**	**IRR UCI**	**IRR *p***	**OR**	**OR LCI**	**OR UCI**	**OR *p***
Belief (ref = No)	Not sure	Unadjusted	995	0.975	0.913	1.042	0.4566	0.765	0.492	1.188	0.2325
	Yes			0.977	0.900	1.062	0.5893	1.033	0.626	1.706	0.8987
	Not sure	Adjusted	995	0.974	0.910	1.042	0.4458	0.814	0.507	1.308	0.3955
	Yes			0.974	0.895	1.060	0.5402	1.069	0.621	1.840	0.8099
	Not sure	Adjusted (politics)	995	0.984	0.919	1.054	0.6474	0.793	0.491	1.281	0.3431
	Yes			0.983	0.903	1.070	0.6903	1.065	0.613	1.850	0.8236
Identity (combined; ref = None)	Christian	Unadjusted	994	0.921	0.865	0.981	0.0108	1.176	0.803	1.722	0.4043
	Christian	Adjusted	994	0.928	0.870	0.989	0.0218	1.187	0.785	1.794	0.4171
	Christian	Adjusted (politics)	994	0.934	0.876	0.996	0.0385	1.119	0.735	1.703	0.6002
Identity (separate; ref = None)	C of E	Unadjusted	994	0.912	0.847	0.982	0.0147	1.367	0.896	2.085	0.1468
	Catholic			0.918	0.780	1.081	0.3040	1.272	0.503	3.215	0.6114
	Other			0.950	0.848	1.063	0.3713	0.594	0.241	1.465	0.2583
	C of E	Adjusted	994	0.920	0.854	0.993	0.0314	1.082	1.012	1.158	0.1961
	Catholic			0.942	0.798	1.111	0.4762	0.928	0.770	1.118	0.8672
	Other			0.920	0.820	1.032	0.1544	1.036	0.973	1.103	0.3778
	C of E	Adjusted (politics)	994	0.934	0.865	1.008	0.0782	1.062	0.992	1.137	0.2465
	Catholic			0.955	0.810	1.127	0.5885	0.931	0.772	1.121	0.9980
	Other			0.927	0.826	1.040	0.1978	1.038	0.975	1.105	0.3521
Attendance (ref = Occasional/None)	Regular	Unadjusted	987	1.017	0.897	1.152	0.7940	1.022	0.465	2.245	0.9571
	Regular	Adjusted	987	1.017	0.893	1.157	0.8038	1.198	0.517	2.777	0.6728
	Regular	Adjusted (politics)	987	1.019	0.896	1.158	0.7752	1.151	0.493	2.685	0.7447
Latent class (ref = "Atheist")	Agnostic	Unadjusted	952	0.960	0.892	1.033	0.2764	0.712	0.438	1.156	0.1697
	Moderately religious			1.018	0.916	1.132	0.7440	0.680	0.323	1.433	0.3109
	Highly religious			0.971	0.871	1.082	0.5915	0.829	0.416	1.653	0.5948
	Agnostic	Adjusted	952	0.958	0.888	1.033	0.2620	0.758	0.448	1.282	0.3016
	Moderately religious			0.989	0.888	1.101	0.8422	0.682	0.301	1.546	0.3596
	Highly religious			0.980	0.877	1.096	0.7261	0.902	0.431	1.889	0.7840
	Agnostic	Adjusted (politics)	952	0.959	0.890	1.035	0.2824	0.732	0.430	1.246	0.2504

	Moderately religious			0.996	0.894	1.109	0.9384	0.669	0.294	1.520	0.3367
	Highly religious			0.977	0.874	1.091	0.6749	0.889	0.423	1.871	0.7573

Table S43: Results of the offspring multinomial regression models for each of the individual climate change action outcomes for four religious exposures (belief, identity [both Christian denominations combined together and separate], attendance, and latent classes). The '*p* total' column is the *p*-value for the overall association between the exposure and outcome. RRR = Relative risk ratio; LCI = Lower 95% confidence interval; UCI = Upper 95% confidence interval.

**Exposure**	**Exposure level**	**Outcome level (ref = No)**	**Model**	***n***	**RRR**	**LCI**	**UCI**	***p*-value**	***p* total**
Changed the way travel locally									
Belief (ref = No)	Not sure	For climate reasons	Unadjusted	1094	0.923	0.608	1.401	0.7059	0.5736
		For other reasons			1.048	0.719	1.527	0.8078	
		For climate and other			1.002	0.530	1.894	0.9960	
	Yes	For climate reasons			1.370	0.858	2.189	0.1872	
		For other reasons			1.314	0.843	2.049	0.2281	
		For climate and other			0.628	0.239	1.653	0.3461	
	Not sure	For climate reasons	Adjusted	1094	0.947	0.612	1.466	0.8085	0.5129
		For other reasons			1.078	0.729	1.594	0.7063	
		For climate and other			1.080	0.555	2.102	0.8198	
	Yes	For climate reasons			1.423	0.872	2.322	0.1580	
		For other reasons			1.363	0.862	2.157	0.1854	
		For climate and other			0.625	0.230	1.703	0.3583	
	Not sure	For climate reasons	Adjusted (politics)	1094	0.995	0.639	1.548	0.9814	0.4761
		For other reasons			1.099	0.742	1.628	0.6388	
		For climate and other			1.163	0.593	2.279	0.6606	
	Yes	For climate reasons			1.506	0.918	2.471	0.1054	
		For other reasons			1.399	0.882	2.220	0.1543	
		For climate and other			0.686	0.250	1.882	0.4646	
Identity (combined; ref = None)	Christian	For climate reasons	Unadjusted	1093	1.072	0.740	1.554	0.7129	0.7710
		For other reasons			1.130	0.803	1.590	0.4822	
		For climate and other			0.813	0.432	1.532	0.5223	
	Christian	For climate reasons	Adjusted	1093	1.096	0.744	1.614	0.6432	0.7910
		For other reasons			1.160	0.814	1.651	0.4113	
		For climate and other			0.879	0.457	1.692	0.6996	
	Christian	For climate reasons	Adjusted (politics)	1093	1.182	0.798	1.751	0.4051	0.6924
		For other reasons			1.186	0.829	1.695	0.3505	
		For climate and other			0.929	0.478	1.803	0.8268	
Identity (separate; ref = None)	C of E	For climate reasons	Unadjusted	1093	0.947	0.616	1.456	0.8033	0.1294
		For other reasons			0.892	0.593	1.341	0.5821	
		For climate and other			0.555	0.243	1.268	0.1623	

**Exposure**	**Exposure level**	**Outcome level (ref = No)**	**Model**	***n***	**RRR**	**LCI**	**UCI**	***p*-value**	***p* total**
	Catholic	For climate reasons			0.547	0.162	1.847	0.3313	
		For other reasons			1.533	0.717	3.275	0.2703	
		For climate and other			1.008	0.230	4.419	0.9911	
	Other	For climate reasons			2.074	1.087	3.956	0.0269	
		For other reasons			1.974	1.065	3.660	0.0308	
		For climate and other			1.909	0.704	5.174	0.2037	
	C of E	For climate reasons	Adjusted	1093	0.989	0.630	1.552	0.9608	0.3057
		For other reasons			0.898	0.588	1.371	0.6184	
		For climate and other			0.651	0.278	1.522	0.3220	
	Catholic	For climate reasons			0.626	0.180	2.175	0.4614	
		For other reasons			1.692	0.762	3.757	0.1965	
		For climate and other			1.133	0.246	5.226	0.8729	
	Other	For climate reasons			1.881	0.949	3.727	0.0701	
		For other reasons			2.079	1.085	3.985	0.0274	
		For climate and other			1.628	0.567	4.675	0.3655	
	C of E	For climate reasons	Adjusted (politics)	1093	1.065	0.674	1.683	0.7884	0.2924
		For other reasons			0.920	0.600	1.409	0.7008	
		For climate and other			0.694	0.294	1.638	0.4047	
	Catholic	For climate reasons			0.675	0.193	2.360	0.5384	
		For other reasons			1.720	0.774	3.825	0.1835	
		For climate and other			1.101	0.237	5.117	0.9023	
	Other	For climate reasons			2.030	1.023	4.031	0.0430	
		For other reasons			2.103	1.095	4.039	0.0257	
		For climate and other			1.728	0.597	5.006	0.3133	
Attendance (ref = Occasional/None)	Regular	For climate reasons	Unadjusted	1085	2.074	1.078	3.990	0.0288	0.2237
		For other reasons			1.150	0.555	2.385	0.7063	
		For climate and other			1.261	0.373	4.265	0.7091	
	Regular	For climate reasons	Adjusted	1085	1.918	0.956	3.851	0.0669	0.3544
		For other reasons			1.069	0.501	2.282	0.8629	
		For climate and other			1.084	0.292	4.026	0.9035	
	Regular	For climate reasons	Adjusted (politics)	1085	2.005	0.993	4.046	0.0522	0.3084
		For other reasons			1.091	0.510	2.335	0.8231	
		For climate and other			1.273	0.339	4.778	0.7205	
	Agnostic	For climate reasons	Unadjusted	1042	0.617	0.367	1.036	0.0678	0.1127

**Exposure**	**Exposure level**	**Outcome level (ref = No)**	**Model**	***n***	**RRR**	**LCI**	**UCI**	***p*-value**	***p* total**
		For other reasons			1.204	0.810	1.790	0.3578	
		For climate and other			0.860	0.413	1.787	0.6855	
	Moderately religious	For climate reasons			2.014	1.136	3.573	0.0166	
		For other reasons			1.574	0.875	2.829	0.1299	
		For climate and other			1.123	0.381	3.315	0.8333	
	Highly religious	For climate reasons			1.283	0.693	2.375	0.4281	
		For other reasons			1.039	0.555	1.944	0.9043	
		For climate and other			0.715	0.212	2.413	0.5892	
Latent class (ref = "Atheist")	Agnostic	For climate reasons	Adjusted	1042	0.550	0.319	0.948	0.0313	0.0825
		For other reasons			1.159	0.764	1.759	0.4866	
		For climate and other			0.793	0.367	1.714	0.5555	
	Moderately religious	For climate reasons			2.025	1.102	3.723	0.0231	
		For other reasons			1.619	0.879	2.980	0.1219	
		For climate and other			1.157	0.372	3.602	0.8008	
	Highly religious	For climate reasons			1.247	0.650	2.391	0.5065	
		For other reasons			1.066	0.557	2.040	0.8467	
		For climate and other			0.653	0.181	2.359	0.5157	
	Agnostic	For climate reasons	Adjusted (politics)	1042	0.569	0.329	0.986	0.0443	0.0929
		For other reasons			1.177	0.774	1.790	0.4456	
		For climate and other			0.851	0.391	1.856	0.6855	
	Moderately religious	For climate reasons			2.129	1.154	3.928	0.0156	
		For other reasons			1.641	0.891	3.022	0.1122	
		For climate and other			1.131	0.358	3.570	0.8342	
	Highly religious	For climate reasons			1.250	0.648	2.412	0.5064	
		For other reasons			1.071	0.558	2.055	0.8377	
		For climate and other			0.747	0.205	2.728	0.6590	
Reduced household waste									
Belief (ref = No)	Not sure	For climate reasons	Unadjusted	1096	1.175	0.839	1.647	0.3474	0.8743
		For other reasons			0.930	0.535	1.617	0.7971	
		For climate and other			1.096	0.592	2.027	0.7713	
	Yes	For climate reasons			1.123	0.744	1.696	0.5814	
		For other reasons			1.115	0.587	2.118	0.7398	
		For climate and other			0.780	0.339	1.798	0.5605	

Exposure	Exposure level	Outcome level (ref = No)	Model	*n*	RRR	LCI	UCI	*p*-value	*p* total
	Not sure	For climate reasons	Adjusted	1096	1.103	0.775	1.568	0.5872	0.8931
		For other reasons			0.844	0.477	1.495	0.5615	
		For climate and other			1.073	0.570	2.021	0.8276	
	Yes	For climate reasons			1.081	0.705	1.658	0.7211	
		For other reasons			1.169	0.605	2.259	0.6430	
		For climate and other			0.775	0.331	1.817	0.5581	
	Not sure	For climate reasons	Adjusted (politics)	1096	1.116	0.782	1.591	0.5461	0.8579
		For other reasons			0.828	0.466	1.470	0.5188	
		For climate and other			1.065	0.563	2.011	0.8471	
	Yes	For climate reasons			1.085	0.704	1.670	0.7116	
		For other reasons			1.163	0.599	2.260	0.6551	
		For climate and other			0.763	0.324	1.796	0.5361	
Identity (combined; ref = None)	Christian	For climate reasons	Unadjusted	1095	1.128	0.825	1.541	0.4502	0.0618
		For other reasons			1.533	0.950	2.474	0.0799	
		For climate and other			0.616	0.323	1.175	0.1413	
	Christian	For climate reasons	Adjusted	1095	1.097	0.792	1.517	0.5780	0.0794
		For other reasons			1.496	0.914	2.450	0.1092	
		For climate and other			0.603	0.312	1.164	0.1316	
	Christian	For climate reasons	Adjusted (politics)	1095	1.126	0.810	1.565	0.4809	0.0771
		For other reasons			1.481	0.899	2.440	0.1228	
		For climate and other			0.598	0.309	1.161	0.1287	
Identity (separate; ref = None)	C of E	For climate reasons	Unadjusted	1095	1.011	0.711	1.438	0.9510	0.1180
		For other reasons			1.398	0.814	2.402	0.2244	
		For climate and other			0.535	0.249	1.151	0.1098	
	Catholic	For climate reasons			1.565	0.670	3.657	0.3011	
		For other reasons			2.530	0.819	7.814	0.1067	
		For climate and other			NA	NA	NA	NA	
	Other	For climate reasons			1.404	0.740	2.665	0.2989	
		For other reasons			1.589	0.606	4.161	0.3461	
		For climate and other			1.303	0.445	3.818	0.6295	
	C of E	For climate reasons	Adjusted	1095	0.956	0.659	1.386	0.8118	0.1039
		For other reasons			1.272	0.723	2.236	0.4035	
		For climate and other			0.526	0.240	1.152	0.1083	
	Catholic	For climate reasons			1.843	0.763	4.452	0.1743	

Exposure	Exposure level	Outcome level (ref = No)	Model	*n*	RRR	LCI	UCI	*p*-value	*p* total
		For other reasons			2.928	0.909	9.435	0.0719	
		For climate and other			NA	NA	NA	NA	
	Other	For climate reasons			1.327	0.684	2.572	0.4026	
		For other reasons			1.762	0.653	4.753	0.2635	
		For climate and other			1.210	0.402	3.647	0.7347	
	C of E	For climate reasons	Adjusted (politics)	1095	0.976	0.670	1.421	0.8997	0.0985
		For other reasons			1.256	0.710	2.220	0.4332	
		For climate and other			0.517	0.235	1.139	0.1018	
	Catholic	For climate reasons			1.963	0.808	4.767	0.1363	
		For other reasons			2.871	0.890	9.264	0.0776	
		For climate and other			NA	NA	NA	NA	
	Other	For climate reasons			1.352	0.695	2.631	0.3743	
		For other reasons			1.754	0.648	4.752	0.2690	
		For climate and other			1.201	0.397	3.633	0.7462	
Attendance (ref = Occasional/None)	Regular	For climate reasons	Unadjusted	1087	0.902	0.484	1.681	0.7452	0.9599
		For other reasons			1.019	0.384	2.704	0.9698	
		For climate and other			0.745	0.209	2.650	0.6488	
	Regular	For climate reasons	Adjusted	1087	0.869	0.453	1.669	0.6740	0.9487
		For other reasons			1.031	0.376	2.831	0.9527	
		For climate and other			0.746	0.202	2.759	0.6611	
	Regular	For climate reasons	Adjusted (politics)	1087	0.843	0.436	1.627	0.6100	0.9212
		For other reasons			1.028	0.371	2.849	0.9570	
		For climate and other			0.712	0.191	2.653	0.6129	
Latent class (ref = "Atheist")	Agnostic	For climate reasons	Unadjusted	1044	1.185	0.817	1.719	0.3706	0.8680
		For other reasons			0.926	0.502	1.708	0.8050	
		For climate and other			1.008	0.511	1.987	0.9817	
	Moderately religious	For climate reasons			1.505	0.852	2.658	0.1594	
		For other reasons			1.059	0.419	2.676	0.9040	
		For climate and other			0.847	0.271	2.643	0.7748	
	Highly religious	For climate reasons			1.056	0.616	1.810	0.8442	
		For other reasons			0.979	0.412	2.326	0.9625	
		For climate and other			0.686	0.224	2.099	0.5087	
	Agnostic	For climate reasons	Adjusted	1044	1.105	0.747	1.635	0.6157	0.8895
		For other reasons			0.811	0.428	1.537	0.5204	

Exposure	Exposure level	Outcome level (ref = No)	Model	*n*	RRR	LCI	UCI	*p*-value	*p* total
		For climate and other			0.900	0.446	1.813	0.7673	
	Moderately religious	For climate reasons			1.502	0.828	2.724	0.1802	
		For other reasons			1.126	0.433	2.925	0.8079	
		For climate and other			0.848	0.263	2.732	0.7829	
	Highly religious	For climate reasons			0.955	0.544	1.678	0.8733	
		For other reasons			0.939	0.387	2.281	0.8901	
		For climate and other			0.655	0.208	2.063	0.4697	
	Agnostic	For climate reasons	Adjusted (politics)	1044	1.116	0.752	1.655	0.5859	0.8315
		For other reasons			0.792	0.417	1.505	0.4769	
		For climate and other			0.890	0.440	1.797	0.7444	
	Moderately religious	For climate reasons			1.549	0.852	2.815	0.1513	
		For other reasons			1.114	0.428	2.900	0.8246	
		For climate and other			0.865	0.269	2.786	0.8085	
	Highly religious	For climate reasons			0.921	0.521	1.629	0.7783	
		For other reasons			0.928	0.380	2.267	0.8699	
		For climate and other			0.626	0.197	1.986	0.4262	
Reduced energy use at home									
Belief (ref = No)	Not sure	For climate reasons	Unadjusted	1095	1.290	0.916	1.819	0.1451	0.0677
		For other reasons			1.679	1.141	2.472	0.0086	
		For climate and other			1.185	0.709	1.981	0.5169	
	Yes	For climate reasons			1.026	0.691	1.522	0.9001	
		For other reasons			1.002	0.623	1.613	0.9928	
		For climate and other			0.502	0.236	1.066	0.0730	
	Not sure	For climate reasons	Adjusted	1095	1.257	0.882	1.791	0.2051	0.0586
		For other reasons			1.746	1.171	2.604	0.0063	
		For climate and other			1.149	0.675	1.955	0.6097	
	Yes	For climate reasons			1.026	0.684	1.540	0.9007	
		For other reasons			0.981	0.602	1.600	0.9397	
		For climate and other			0.499	0.230	1.082	0.0785	
	Not sure	For climate reasons	Adjusted (politics)	1095	1.253	0.877	1.789	0.2148	0.0788
		For other reasons			1.700	1.137	2.540	0.0097	
		For climate and other			1.163	0.681	1.986	0.5800	
	Yes	For climate reasons			1.013	0.673	1.524	0.9512	

Exposure	Exposure level	Outcome level (ref = No)	Model	*n*	RRR	LCI	UCI	*p*-value	*p* total
		For other reasons			0.941	0.575	1.541	0.8094	
		For climate and other			0.507	0.233	1.101	0.0860	
Identity (combined; ref = None)	Christian	For climate reasons	Unadjusted	1094	0.917	0.677	1.243	0.5780	0.0227
		For other reasons			1.018	0.716	1.446	0.9224	
		For climate and other			0.459	0.266	0.793	0.0052	
	Christian	For climate reasons	Adjusted	1094	0.946	0.692	1.294	0.7287	0.0289
		For other reasons			1.023	0.713	1.468	0.9029	
		For climate and other			0.461	0.263	0.808	0.0068	
	Christian	For climate reasons	Adjusted (politics)	1094	0.948	0.691	1.300	0.7401	0.0381
		For other reasons			0.989	0.686	1.425	0.9511	
		For climate and other			0.465	0.264	0.818	0.0078	
Identity (separate; ref = None)	C of E	For climate reasons	Unadjusted	1094	0.934	0.662	1.319	0.6990	0.0209
		For other reasons			0.957	0.639	1.435	0.8333	
		For climate and other			0.348	0.172	0.703	0.0033	
	Catholic	For climate reasons			0.490	0.220	1.090	0.0804	
		For other reasons			1.087	0.505	2.339	0.8316	
		For climate and other			0.168	0.022	1.281	0.0852	
	Other	For climate reasons			1.227	0.668	2.254	0.5092	
		For other reasons			1.215	0.599	2.465	0.5888	
		For climate and other			1.202	0.507	2.849	0.6769	
	C of E	For climate reasons	Adjusted	1094	0.960	0.671	1.373	0.8232	0.0400
		For other reasons			0.951	0.625	1.448	0.8142	
		For climate and other			0.348	0.169	0.717	0.0042	
	Catholic	For climate reasons			0.527	0.231	1.203	0.1280	
		For other reasons			1.062	0.476	2.368	0.8833	
		For climate and other			0.166	0.021	1.321	0.0897	
	Other	For climate reasons			1.247	0.666	2.334	0.4907	
		For other reasons			1.282	0.619	2.656	0.5030	
		For climate and other			1.188	0.485	2.906	0.7065	
	C of E	For climate reasons	Adjusted (politics)	1094	0.957	0.667	1.374	0.8126	0.0523
		For other reasons			0.914	0.598	1.397	0.6781	
		For climate and other			0.349	0.169	0.722	0.0045	
	Catholic	For climate reasons			0.546	0.238	1.251	0.1526	
		For other reasons			1.063	0.475	2.380	0.8825	

Exposure	Exposure level	Outcome level (ref = No)	Model	*n*	RRR	LCI	UCI	*p*-value	*p* total
		For climate and other			0.172	0.022	1.370	0.0965	
	Other	For climate reasons			1.245	0.664	2.332	0.4946	
		For other reasons			1.235	0.595	2.563	0.5710	
		For climate and other			1.192	0.486	2.920	0.7015	
Attendance (ref = Occasional/None)	Regular	For climate reasons	Unadjusted	1086	0.992	0.528	1.866	0.9812	0.9948
		For other reasons			1.045	0.506	2.160	0.9049	
		For climate and other			0.905	0.329	2.486	0.8457	
	Regular	For climate reasons	Adjusted	1086	0.996	0.518	1.917	0.9911	0.9993
		For other reasons			1.004	0.472	2.136	0.9912	
		For climate and other			0.934	0.326	2.682	0.8995	
	Regular	For climate reasons	Adjusted (politics)	1086	0.951	0.493	1.836	0.8814	0.9982
		For other reasons			0.938	0.439	2.004	0.8696	
		For climate and other			0.938	0.325	2.706	0.9060	
Latent class (ref = "Atheist")	Agnostic	For climate reasons	Unadjusted	1044	1.163	0.795	1.701	0.4369	0.3822
		For other reasons			1.662	1.094	2.525	0.0174	
		For climate and other			1.183	0.676	2.068	0.5559	
	Moderately religious	For climate reasons			1.078	0.641	1.816	0.7764	
		For other reasons			1.048	0.561	1.955	0.8838	
		For climate and other			0.556	0.207	1.489	0.2426	
	Highly religious	For climate reasons			1.116	0.660	1.886	0.6830	
		For other reasons			0.903	0.466	1.749	0.7624	
		For climate and other			0.690	0.275	1.732	0.4291	
	Agnostic	For climate reasons	Adjusted	1044	1.086	0.732	1.611	0.6825	0.2221
		For other reasons			1.760	1.138	2.722	0.0111	
		For climate and other			1.090	0.610	1.947	0.7721	
	Moderately religious	For climate reasons			1.028	0.598	1.766	0.9215	
		For other reasons			1.023	0.534	1.957	0.9460	
		For climate and other			0.475	0.172	1.308	0.1498	
	Highly religious	For climate reasons			1.108	0.644	1.907	0.7119	
		For other reasons			0.849	0.429	1.677	0.6367	
		For climate and other			0.727	0.280	1.888	0.5125	
	Agnostic	For climate reasons	Adjusted (politics)	1044	1.085	0.730	1.614	0.6861	0.2564
		For other reasons			1.717	1.108	2.661	0.0156	
		For climate and other			1.097	0.612	1.967	0.7548	

Exposure	Exposure level	Outcome level (ref = No)	Model	*n*	RRR	LCI	UCI	*p*-value	*p* total
	Moderately religious	For climate reasons			1.051	0.611	1.810	0.8562	
		For other reasons			1.024	0.534	1.962	0.9428	
		For climate and other			0.478	0.173	1.320	0.1545	
	Highly religious	For climate reasons			1.057	0.611	1.827	0.8433	
		For other reasons			0.802	0.404	1.591	0.5282	
		For climate and other			0.724	0.277	1.888	0.5087	
Changed what buy									
Belief (ref = No)	Not sure	For climate reasons	Unadjusted	1092	1.273	0.928	1.746	0.1349	0.2176
		For other reasons			1.373	0.867	2.176	0.1765	
		For climate and other			1.597	0.935	2.728	0.0868	
	Yes	For climate reasons			1.255	0.858	1.836	0.2418	
		For other reasons			1.356	0.782	2.353	0.2785	
		For climate and other			0.675	0.289	1.577	0.3646	
	Not sure	For climate reasons	Adjusted	1092	1.251	0.894	1.750	0.1917	0.2086
		For other reasons			1.360	0.848	2.181	0.2015	
		For climate and other			1.722	0.983	3.017	0.0573	
	Yes	For climate reasons			1.267	0.846	1.899	0.2512	
		For other reasons			1.416	0.807	2.487	0.2254	
		For climate and other			0.709	0.297	1.690	0.4376	
	Not sure	For climate reasons	Adjusted (politics)	1092	1.324	0.943	1.859	0.1053	0.1043
		For other reasons			1.473	0.912	2.378	0.1133	
		For climate and other			1.860	1.053	3.285	0.0325	
	Yes	For climate reasons			1.347	0.893	2.029	0.1552	
		For other reasons			1.557	0.879	2.759	0.1289	
		For climate and other			0.764	0.318	1.833	0.5463	
Identity (combined; ref = None)	Christian	For climate reasons	Unadjusted	1091	1.065	0.799	1.419	0.6672	0.0681
		For other reasons			1.365	0.905	2.060	0.1381	
		For climate and other			0.585	0.324	1.055	0.0749	
	Christian	For climate reasons	Adjusted	1091	1.134	0.836	1.540	0.4191	0.0698
		For other reasons			1.403	0.920	2.138	0.1154	
		For climate and other			0.609	0.331	1.120	0.1108	
	Christian	For climate reasons	Adjusted (politics)		1.221	0.895	1.666	0.2066	0.0380
		For other reasons			1.583	1.029	2.437	0.0368	

Exposure	Exposure level	Outcome level (ref = No)	Model	*n*	RRR	LCI	UCI	*p*-value	*p* total
		For climate and other			0.669	0.361	1.241	0.2023	
Identity (separate; ref = None)	C of E	For climate reasons	Unadjusted	1091	1.038	0.750	1.437	0.8203	0.0189
		For other reasons			1.067	0.652	1.746	0.7961	
		For climate and other			0.510	0.251	1.038	0.0633	
	Catholic	For climate reasons			0.771	0.382	1.555	0.4676	
		For other reasons			1.350	0.541	3.366	0.5203	
		For climate and other			NA	NA	NA	NA	
	Other	For climate reasons			1.494	0.809	2.757	0.1994	
		For other reasons			2.892	1.384	6.039	0.0047	
		For climate and other			1.547	0.586	4.085	0.3782	
	C of E	For climate reasons	Adjusted	1091	1.139	0.803	1.615	0.4665	0.0186
		For other reasons			1.055	0.635	1.755	0.8354	
		For climate and other			0.558	0.267	1.164	0.1199	
	Catholic	For climate reasons			0.872	0.405	1.878	0.7271	
		For other reasons			1.498	0.584	3.847	0.4006	
		For climate and other			NA	NA	NA	NA	
	Other	For climate reasons			1.388	0.731	2.637	0.3164	
		For other reasons			3.088	1.450	6.579	0.0035	
		For climate and other			1.372	0.502	3.751	0.5371	
	C of E	For climate reasons	Adjusted (politics)	1091	1.224	0.859	1.743	0.2640	0.0111
		For other reasons			1.180	0.703	1.981	0.5312	
		For climate and other			0.612	0.291	1.287	0.1959	
	Catholic	For climate reasons			0.929	0.428	2.016	0.8522	
		For other reasons			1.740	0.672	4.508	0.2538	
		For climate and other			NA	NA	NA	NA	
	Other	For climate reasons			1.501	0.784	2.874	0.2203	
		For other reasons			3.491	1.621	7.518	0.0014	
		For climate and other			1.510	0.548	4.157	0.4254	
Attendance (ref = Occasional/None)	Regular	For climate reasons	Unadjusted	1083	1.263	0.688	2.319	0.4518	0.6771
		For other reasons			1.520	0.666	3.472	0.3201	
		For climate and other			0.824	0.237	2.869	0.7615	
	Regular	For climate reasons	Adjusted	1083	1.300	0.682	2.476	0.4257	0.6817
		For other reasons			1.445	0.614	3.401	0.3989	
		For climate and other			0.783	0.217	2.825	0.7086	

**Exposure**	**Exposure level**	**Outcome level (ref = No)**	**Model**	***n***	**RRR**	**LCI**	**UCI**	***p*-value**	***p* total**
	Regular	For climate reasons	Adjusted (politics)	1083	1.362	0.710	2.612	0.3527	0.6062
		For other reasons			1.552	0.655	3.678	0.3181	
		For climate and other			0.822	0.226	2.988	0.7658	
Latent class (ref = "Atheist")	Agnostic	For climate reasons	Unadjusted	1040	1.346	0.945	1.917	0.0991	0.2541
		For other reasons			1.600	0.977	2.619	0.0616	
		For climate and other			1.676	0.938	2.992	0.0810	
	Moderately religious	For climate reasons			1.591	0.960	2.637	0.0713	
		For other reasons			1.259	0.583	2.719	0.5573	
		For climate and other			0.804	0.269	2.403	0.6959	
	Highly religious	For climate reasons			1.509	0.899	2.533	0.1196	
		For other reasons			1.438	0.679	3.047	0.3424	
		For climate and other			1.043	0.381	2.855	0.9340	
	Agnostic	For climate reasons	Adjusted	1040	1.314	0.900	1.918	0.1579	0.3362
		For other reasons			1.643	0.985	2.738	0.0570	
		For climate and other			1.656	0.897	3.057	0.1069	
	Moderately religious	For climate reasons			1.470	0.854	2.531	0.1643	
		For other reasons			1.305	0.593	2.875	0.5081	
		For climate and other			0.788	0.255	2.431	0.6785	
	Highly religious	For climate reasons			1.622	0.937	2.808	0.0838	
		For other reasons			1.507	0.698	3.255	0.2966	
		For climate and other			1.158	0.411	3.260	0.7815	
	Agnostic	For climate reasons	Adjusted (politics)	1040	1.386	0.946	2.032	0.0941	0.2229
		For other reasons			1.758	1.048	2.947	0.0325	
		For climate and other			1.767	0.949	3.289	0.0725	
	Moderately religious	For climate reasons			1.523	0.880	2.635	0.1326	
		For other reasons			1.359	0.613	3.012	0.4501	
		For climate and other			0.826	0.267	2.559	0.7409	
	Highly religious	For climate reasons			1.672	0.960	2.914	0.0695	
		For other reasons			1.586	0.729	3.447	0.2445	
		For climate and other			1.199	0.423	3.399	0.7334	
Reduced air travel									
Belief (ref = No)	Not sure	For climate reasons	Unadjusted	1091	0.805	0.488	1.326	0.3941	0.1666
		For other reasons			1.266	0.912	1.757	0.1583	

Exposure	Exposure level	Outcome level (ref = No)	Model	*n*	RRR	LCI	UCI	*p*-value	*p* total
		For climate and other			0.565	0.242	1.321	0.1877	
	Yes	For climate reasons			0.935	0.511	1.708	0.8264	
		For other reasons			1.489	1.007	2.203	0.0463	
		For climate and other			0.600	0.205	1.754	0.3508	
	Not sure	For climate reasons	Adjusted	1091	0.823	0.491	1.380	0.4604	0.1572
		For other reasons			1.241	0.886	1.739	0.2091	
		For climate and other			0.486	0.203	1.165	0.1057	
	Yes	For climate reasons			0.970	0.521	1.804	0.9233	
		For other reasons			1.512	1.013	2.257	0.0430	
		For climate and other			0.650	0.215	1.963	0.4453	
	Not sure	For climate reasons	Adjusted (politics)	1091	0.880	0.521	1.489	0.6347	0.2381
		For other reasons			1.227	0.874	1.721	0.2377	
		For climate and other			0.497	0.206	1.199	0.1197	
	Yes	For climate reasons			1.038	0.554	1.946	0.9070	
		For other reasons			1.487	0.994	2.224	0.0536	
		For climate and other			0.672	0.221	2.041	0.4835	
Identity (combined; ref = None)	Christian	For climate reasons	Unadjusted	1090	0.758	0.475	1.209	0.2450	0.0391
		For other reasons			1.188	0.881	1.603	0.2575	
		For climate and other			0.405	0.167	0.984	0.0459	
	Christian	For climate reasons	Adjusted	1090	0.781	0.483	1.263	0.3127	0.0649
		For other reasons			1.192	0.878	1.619	0.2611	
		For climate and other			0.418	0.168	1.041	0.0610	
	Christian	For climate reasons	Adjusted (politics)	1090	0.862	0.529	1.407	0.5534	0.1325
		For other reasons			1.178	0.865	1.604	0.2993	
		For climate and other			0.437	0.174	1.099	0.0785	
Identity (separate; ref = None)	C of E	For climate reasons	Unadjusted	1090	0.526	0.284	0.973	0.0407	0.0637
		For other reasons			1.220	0.871	1.709	0.2481	
		For climate and other			0.292	0.088	0.967	0.0439	
	Catholic	For climate reasons			0.700	0.207	2.368	0.5660	
		For other reasons			1.050	0.496	2.224	0.8978	
		For climate and other			0.561	0.074	4.270	0.5770	
	Other	For climate reasons			1.668	0.820	3.391	0.1576	
		For other reasons			1.161	0.641	2.102	0.6229	
		For climate and other			0.730	0.169	3.157	0.6732	

Exposure	Exposure level	Outcome level (ref = No)	Model	*n*	RRR	LCI	UCI	*p*-value	*p* total
	C of E	For climate reasons	Adjusted	1090	0.552	0.293	1.040	0.0658	0.1589
		For other reasons			1.212	0.855	1.716	0.2798	
		For climate and other			0.297	0.087	1.016	0.0530	
	Catholic	For climate reasons			0.766	0.218	2.684	0.6764	
		For other reasons			1.153	0.534	2.492	0.7170	
		For climate and other			0.585	0.071	4.832	0.6183	
	Other	For climate reasons			1.527	0.728	3.200	0.2627	
		For other reasons			1.143	0.621	2.101	0.6680	
		For climate and other			0.770	0.170	3.484	0.7346	
	C of E	For climate reasons	Adjusted (politics)	1090	0.612	0.322	1.163	0.1338	0.2675
		For other reasons			1.192	0.839	1.694	0.3258	
		For climate and other			0.312	0.090	1.077	0.0654	
	Catholic	For climate reasons			0.857	0.241	3.042	0.8113	
		For other reasons			1.161	0.536	2.513	0.7049	
		For climate and other			0.626	0.076	5.188	0.6642	
	Other	For climate reasons			1.661	0.784	3.517	0.1853	
		For other reasons			1.136	0.617	2.093	0.6824	
		For climate and other			0.789	0.173	3.595	0.7597	
Attendance (ref = Occasional/None)	Regular	For climate reasons	Unadjusted	1082	1.835	0.817	4.123	0.1415	0.0453
		For other reasons			1.668	0.922	3.017	0.0910	
		For climate and other			NA	NA	NA	NA	
	Regular	For climate reasons	Adjusted	1082	1.804	0.773	4.210	0.1721	0.0721
		For other reasons			1.617	0.877	2.982	0.1238	
		For climate and other			NA	NA	NA	NA	
	Regular	For climate reasons	Adjusted (politics)	1082	1.896	0.805	4.465	0.1430	0.0737
		For other reasons			1.569	0.848	2.904	0.1513	
		For climate and other			NA	NA	NA	NA	
Latent class (ref = "Atheist")	Agnostic	For climate reasons	Unadjusted	1040	0.491	0.252	0.956	0.0365	0.0062
		For other reasons			1.392	0.983	1.973	0.0626	
		For climate and other			0.665	0.269	1.644	0.3770	
	Moderately religious	For climate reasons			0.546	0.211	1.414	0.2126	
		For other reasons			1.169	0.694	1.969	0.5564	
		For climate and other			1.085	0.366	3.215	0.8832	
	Highly religious	For climate reasons			1.360	0.654	2.828	0.4108	

Exposure	Exposure level	Outcome level (ref = No)	Model	*n*	RRR	LCI	UCI	*p*-value	*p* total
		For other reasons			1.771	1.065	2.946	0.0275	
		For climate and other			NA	NA	NA	NA	
	Agnostic	For climate reasons	Adjusted	1040	0.463	0.233	0.918	0.0275	0.0069
		For other reasons			1.316	0.918	1.886	0.1353	
		For climate and other			0.576	0.226	1.469	0.2485	
	Moderately religious	For climate reasons			0.486	0.182	1.300	0.1508	
		For other reasons			1.192	0.696	2.041	0.5225	
		For climate and other			0.991	0.319	3.075	0.9877	
	Highly religious	For climate reasons			1.479	0.687	3.184	0.3175	
		For other reasons			1.730	1.026	2.920	0.0399	
		For climate and other			NA	NA	NA	NA	
	Agnostic	For climate reasons	Adjusted (politics)	1040	0.501	0.250	1.002	0.0506	0.0138
		For other reasons			1.304	0.909	1.872	0.1496	
		For climate and other			0.568	0.221	1.457	0.2392	
	Moderately religious	For climate reasons			0.508	0.189	1.368	0.1804	
		For other reasons			1.196	0.698	2.049	0.5146	
		For climate and other			1.023	0.330	3.175	0.9684	
	Highly religious	For climate reasons			1.498	0.688	3.261	0.3082	
		For other reasons			1.695	1.001	2.869	0.0494	
		For climate and other			NA	NA	NA	NA	
Bought or hired an electric or hybrid vehicle									
Belief (ref = No)	Not sure	For climate reasons	Unadjusted	1099	0.762	0.351	1.654	0.4914	0.6295
		For other reasons			0.741	0.309	1.772	0.4999	
		For climate and other			1.270	0.301	5.350	0.7450	
	Yes	For climate reasons			0.591	0.203	1.724	0.3360	
		For other reasons			0.739	0.249	2.194	0.5862	
		For climate and other			2.957	0.785	11.141	0.1092	
	Not sure	For climate reasons	Adjusted	1099	0.700	0.307	1.594	0.3952	0.8000
		For other reasons			0.760	0.311	1.858	0.5475	
		For climate and other			1.389	0.290	6.644	0.6809	
	Yes	For climate reasons			0.597	0.198	1.800	0.3599	
		For other reasons			0.791	0.260	2.404	0.6794	
		For climate and other			2.399	0.533	10.786	0.2540	

Exposure	Exposure level	Outcome level (ref = No)	Model	*n*	RRR	LCI	UCI	*p*-value	*p* total
	Not sure	For climate reasons	Adjusted (politics)	1099	0.720	0.315	1.647	0.4370	0.8259
		For other reasons			0.727	0.296	1.784	0.4864	
		For climate and other			1.335	0.276	6.453	0.7193	
	Yes	For climate reasons			0.619	0.204	1.873	0.3957	
		For other reasons			0.731	0.237	2.251	0.5850	
		For climate and other			2.241	0.492	10.215	0.2969	
Identity (combined; ref = None)	Christian	For climate reasons	Unadjusted	1098	0.661	0.309	1.412	0.2849	0.6269
		For other reasons			0.741	0.328	1.674	0.4704	
		For climate and other			1.065	0.318	3.561	0.9190	
	Christian	For climate reasons	Adjusted	1098	0.627	0.285	1.378	0.2454	0.5441
		For other reasons			0.702	0.304	1.618	0.4063	
		For climate and other			0.850	0.214	3.380	0.8178	
	Christian	For climate reasons	Adjusted (politics)	1098	0.645	0.292	1.424	0.2775	0.5008
		For other reasons			0.649	0.277	1.519	0.3189	
		For climate and other			0.749	0.182	3.080	0.6890	
Identity (separate; ref = None)	C of E	For climate reasons	Unadjusted	1098	0.759	0.328	1.757	0.5201	0.8040
		For other reasons			0.821	0.330	2.041	0.6705	
		For climate and other			1.180	0.310	4.485	0.8083	
	Catholic	For climate reasons			0.612	0.081	4.610	0.6336	
		For other reasons			NA	NA	NA	NA	
		For climate and other			NA	NA	NA	NA	
	Other	For climate reasons			0.362	0.049	2.697	0.3211	
		For other reasons			0.912	0.210	3.953	0.9018	
		For climate and other			1.311	0.161	10.641	0.8000	
	C of E	For climate reasons	Adjusted	1098	0.640	0.267	1.533	0.3165	0.6579
		For other reasons			0.769	0.298	1.979	0.5853	
		For climate and other			1.235	0.268	5.697	0.7864	
	Catholic	For climate reasons			0.824	0.102	6.625	0.8552	
		For other reasons			NA	NA	NA	NA	
		For climate and other			NA	NA	NA	NA	
	Other	For climate reasons			0.458	0.059	3.571	0.4564	
		For other reasons			1.032	0.230	4.623	0.9669	
		For climate and other			0.697	0.056	8.622	0.7782	
	C of E	For climate reasons		1098	0.663	0.275	1.599	0.3604	0.6312

Exposure	Exposure level	Outcome level (ref = No)	Model	*n*	RRR	LCI	UCI	*p*-value	*p* total
		For other reasons	Adjusted (politics)		0.701	0.268	1.830	0.4677	
		For climate and other			1.089	0.229	5.189	0.9144	
	Catholic	For climate reasons			0.809	0.100	6.574	0.8432	
		For other reasons			NA	NA	NA	NA	
		For climate and other			NA	NA	NA	NA	
	Other	For climate reasons			0.470	0.060	3.654	0.4704	
		For other reasons			0.981	0.218	4.407	0.9801	
		For climate and other			0.592	0.043	8.124	0.6948	
Attendance (ref = Occasional/None)	Regular	For climate reasons	Unadjusted	1090	NA	NA	NA	NA	0.0987
		For other reasons			1.240	0.288	5.341	0.7726	
		For climate and other			3.473	0.743	16.237	0.1136	
	Regular	For climate reasons	Adjusted	1090	NA	NA	NA	NA	0.1664
		For other reasons			1.537	0.341	6.922	0.5755	
		For climate and other			2.718	0.392	18.820	0.3113	
	Regular	For climate reasons	Adjusted (politics)	1090	NA	NA	NA	NA	0.2209
		For other reasons			1.414	0.310	6.443	0.6540	
		For climate and other			2.276	0.300	17.300	0.4267	
Latent class (ref = “Atheist”)	Agnostic	For climate reasons	Unadjusted	1047	0.434	0.150	1.258	0.1243	0.1164
		For other reasons			0.672	0.250	1.804	0.4297	
		For climate and other			0.403	0.049	3.294	0.3965	
	Moderately religious	For climate reasons			0.541	0.126	2.321	0.4083	
		For other reasons			NA	NA	NA	NA	
		For climate and other			1.005	0.122	8.269	0.9963	
	Highly religious	For climate reasons			0.606	0.141	2.601	0.5000	
		For other reasons			1.499	0.501	4.483	0.4691	
		For climate and other			3.372	0.854	13.315	0.0828	
	Agnostic	For climate reasons	Adjusted	1047	0.420	0.140	1.264	0.1228	0.1022
		For other reasons			0.669	0.242	1.852	0.4391	
		For climate and other			0.314	0.034	2.900	0.3072	
	Moderately religious	For climate reasons			0.523	0.111	2.464	0.4126	
		For other reasons			NA	NA	NA	NA	
		For climate and other			0.632	0.064	6.265	0.6947	
	Highly religious	For climate reasons			0.502	0.111	2.264	0.3699	
		For other reasons			1.929	0.611	6.088	0.2628	

Exposure	Exposure level	Outcome level (ref = No)	Model	*n*	RRR	LCI	UCI	*p*-value	*p* total
		For climate and other			2.808	0.533	14.798	0.2233	
	Agnostic	For climate reasons	Adjusted (politics)	1047	0.424	0.140	1.285	0.1293	0.1160
		For other reasons			0.632	0.227	1.763	0.3807	
		For climate and other			0.294	0.031	2.742	0.2825	
	Moderately religious	For climate reasons			0.521	0.110	2.458	0.4101	
		For other reasons			NA	NA	NA	NA	
		For climate and other			0.654	0.066	6.517	0.7177	
	Highly religious	For climate reasons			0.498	0.110	2.257	0.3660	
		For other reasons			1.807	0.568	5.752	0.3167	
		For climate and other			2.455	0.447	13.484	0.3016	
Bought foods produced locally									
Belief (ref = No)	Not sure	For climate reasons	Unadjusted	1096	1.221	0.874	1.707	0.2419	0.3455
		For other reasons			1.500	1.035	2.175	0.0324	
		For climate and other			1.173	0.685	2.011	0.5607	
	Yes	For climate reasons			1.002	0.658	1.524	0.9930	
		For other reasons			1.474	0.947	2.294	0.0855	
		For climate and other			0.993	0.503	1.961	0.9833	
	Not sure	For climate reasons	Adjusted	1096	1.138	0.803	1.613	0.4675	0.4332
		For other reasons			1.426	0.974	2.089	0.0683	
		For climate and other			1.128	0.647	1.966	0.6701	
	Yes	For climate reasons			0.987	0.639	1.524	0.9525	
		For other reasons			1.534	0.976	2.411	0.0637	
		For climate and other			1.018	0.506	2.047	0.9602	
	Not sure	For climate reasons	Adjusted (politics)	1096	1.164	0.819	1.656	0.3970	0.4553
		For other reasons			1.428	0.972	2.097	0.0691	
		For climate and other			1.169	0.668	2.044	0.5840	
	Yes	For climate reasons			0.994	0.642	1.541	0.9800	
		For other reasons			1.520	0.963	2.397	0.0719	
		For climate and other			1.045	0.518	2.110	0.9016	
Identity (combined; ref = None)	Christian	For climate reasons	Unadjusted	1095	0.949	0.694	1.300	0.7457	0.0231
		For other reasons			1.527	1.094	2.132	0.0128	
		For climate and other			0.770	0.453	1.307	0.3327	
	Christian	For climate reasons	Adjusted	1095	0.927	0.669	1.284	0.6467	0.0363

Exposure	Exposure level	Outcome level (ref = No)	Model	*n*	RRR	LCI	UCI	*p*-value	*p* total
		For other reasons			1.503	1.068	2.115	0.0193	
		For climate and other			0.797	0.463	1.372	0.4124	
	Christian	For climate reasons	Adjusted (politics)	1095	0.954	0.686	1.326	0.7774	0.0485
		For other reasons			1.511	1.069	2.134	0.0193	
		For climate and other			0.820	0.474	1.418	0.4781	
Identity (separate; ref = None)	C of E	For climate reasons	Unadjusted	1095	0.843	0.582	1.221	0.3660	0.0208
		For other reasons			1.721	1.191	2.487	0.0038	
		For climate and other			0.583	0.296	1.148	0.1186	
	Catholic	For climate reasons			1.493	0.688	3.237	0.3104	
		For other reasons			1.701	0.719	4.025	0.2267	
		For climate and other			1.170	0.327	4.190	0.8093	
	Other	For climate reasons			1.055	0.593	1.877	0.8556	
		For other reasons			0.827	0.395	1.730	0.6139	
		For climate and other			1.194	0.505	2.825	0.6860	
	C of E	For climate reasons	Adjusted	1095	0.796	0.541	1.173	0.2486	0.0341
		For other reasons			1.664	1.136	2.437	0.0089	
		For climate and other			0.610	0.304	1.222	0.1633	
	Catholic	For climate reasons			1.793	0.790	4.070	0.1629	
		For other reasons			1.778	0.733	4.310	0.2028	
		For climate and other			1.286	0.344	4.806	0.7087	
	Other	For climate reasons			1.023	0.562	1.864	0.9402	
		For other reasons			0.856	0.403	1.819	0.6856	
		For climate and other			1.147	0.470	2.800	0.7627	
	C of E	For climate reasons	Adjusted (politics)	1095	0.814	0.550	1.203	0.3022	0.0397
		For other reasons			1.668	1.134	2.453	0.0094	
		For climate and other			0.628	0.312	1.264	0.1923	
	Catholic	For climate reasons			1.898	0.833	4.324	0.1270	
		For other reasons			1.823	0.751	4.426	0.1843	
		For climate and other			1.307	0.348	4.901	0.6917	
	Other	For climate reasons			1.057	0.578	1.933	0.8577	
		For other reasons			0.864	0.406	1.840	0.7045	
		For climate and other			1.186	0.485	2.901	0.7090	
Attendance (ref = Occasional/None)	Regular	For climate reasons	Unadjusted	1087	1.070	0.589	1.945	0.8238	0.4397
		For other reasons			0.612	0.275	1.361	0.2284	

Exposure	Exposure level	Outcome level (ref = No)	Model	*n*	RRR	LCI	UCI	*p*-value	*p* total
		For climate and other			0.586	0.174	1.970	0.3877	
	Regular	For climate reasons	Adjusted	1087	1.171	0.626	2.191	0.6217	0.5656
		For other reasons			0.700	0.309	1.589	0.3943	
		For climate and other			0.627	0.181	2.175	0.4623	
	Regular	For climate reasons	Adjusted (politics)	1087	1.143	0.608	2.150	0.6786	0.5734
		For other reasons			0.674	0.296	1.533	0.3468	
		For climate and other			0.650	0.187	2.261	0.4978	
Latent class (ref = "Atheist")	Agnostic	For climate reasons	Unadjusted	1044	1.069	0.734	1.556	0.7289	0.7034
		For other reasons			1.493	1.001	2.229	0.0496	
		For climate and other			1.141	0.636	2.049	0.6581	
	Moderately religious	For climate reasons			1.053	0.605	1.834	0.8548	
		For other reasons			1.567	0.885	2.776	0.1237	
		For climate and other			0.962	0.386	2.396	0.9329	
	Highly religious	For climate reasons			1.030	0.605	1.755	0.9125	
		For other reasons			0.897	0.471	1.711	0.7423	
		For climate and other			0.865	0.349	2.144	0.7548	
	Agnostic	For climate reasons	Adjusted	1044	0.984	0.664	1.459	0.9363	0.6725
		For other reasons			1.477	0.977	2.232	0.0641	
		For climate and other			1.056	0.574	1.940	0.8618	
	Moderately religious	For climate reasons			0.944	0.529	1.688	0.8470	
		For other reasons			1.556	0.863	2.806	0.1415	
		For climate and other			0.783	0.304	2.019	0.6132	
	Highly religious	For climate reasons			1.059	0.607	1.848	0.8391	
		For other reasons			0.968	0.500	1.872	0.9227	
		For climate and other			0.972	0.381	2.483	0.9528	
	Agnostic	For climate reasons	Adjusted (politics)	1044	1.001	0.673	1.489	0.9960	0.6987
		For other reasons			1.461	0.965	2.212	0.0733	
		For climate and other			1.083	0.587	1.999	0.7975	
	Moderately religious	For climate reasons			0.970	0.542	1.738	0.9196	
		For other reasons			1.567	0.868	2.831	0.1363	
		For climate and other			0.780	0.301	2.019	0.6084	
	Highly religious	For climate reasons			1.016	0.579	1.783	0.9550	
		For other reasons			0.920	0.474	1.786	0.8054	
		For climate and other			0.993	0.387	2.548	0.9892	

Exposure	Exposure level	Outcome level (ref = No)	Model	*n*	RRR	LCI	UCI	*p*-value	*p* total
Recycled or upcycled more									
Belief (ref = No)	Not sure	For climate reasons	Unadjusted	1095	1.382	0.913	2.092	0.1264	0.1859
		For other reasons			1.339	0.795	2.254	0.2719	
		For climate and other			1.631	0.940	2.828	0.0816	
	Yes	For climate reasons			1.559	0.934	2.605	0.0897	
		For other reasons			1.152	0.592	2.243	0.6769	
		For climate and other			0.932	0.430	2.020	0.8582	
	Not sure	For climate reasons	Adjusted	1095	1.277	0.829	1.969	0.2674	0.2109
		For other reasons			1.293	0.755	2.213	0.3495	
		For climate and other			1.517	0.859	2.679	0.1513	
	Yes	For climate reasons			1.614	0.947	2.750	0.0785	
		For other reasons			1.157	0.585	2.290	0.6747	
		For climate and other			0.922	0.417	2.037	0.8409	
	Not sure	For climate reasons	Adjusted (politics)	1095	1.294	0.837	2.000	0.2462	0.2122
		For other reasons			1.331	0.775	2.286	0.3006	
		For climate and other			1.549	0.874	2.744	0.1337	
	Yes	For climate reasons			1.629	0.951	2.791	0.0755	
		For other reasons			1.209	0.607	2.408	0.5892	
		For climate and other			0.940	0.423	2.088	0.8791	
Identity (combined; ref = None)	Christian	For climate reasons	Unadjusted	1094	1.252	0.858	1.826	0.2442	0.3063
		For other reasons			1.235	0.766	1.992	0.3859	
		For climate and other			0.868	0.505	1.492	0.6081	
	Christian	For climate reasons	Adjusted	1094	1.306	0.880	1.938	0.1850	0.3013
		For other reasons			1.236	0.755	2.024	0.3997	
		For climate and other			0.913	0.523	1.595	0.7493	
	Christian	For climate reasons	Adjusted (politics)	1094	1.337	0.897	1.993	0.1543	0.2658
		For other reasons			1.296	0.786	2.136	0.3099	
		For climate and other			0.940	0.535	1.650	0.8284	
Identity (separate; ref = None)	C of E	For climate reasons	Unadjusted	1094	1.134	0.741	1.735	0.5618	0.7154
		For other reasons			1.180	0.689	2.020	0.5466	
		For climate and other			0.769	0.411	1.440	0.4122	
	Catholic	For climate reasons			1.426	0.538	3.784	0.4758	
		For other reasons			1.337	0.396	4.510	0.6395	

**Exposure**	**Exposure level**	**Outcome level (ref = No)**	**Model**	***n***	**RRR**	**LCI**	**UCI**	***p*-value**	***p* total**
		For climate and other			0.551	0.104	2.906	0.4821	
	Other	For climate reasons			1.698	0.746	3.862	0.2069	
		For other reasons			1.433	0.515	3.982	0.4907	
		For climate and other			1.573	0.549	4.505	0.3988	
	C of E	For climate reasons	Adjusted	1094	1.180	0.753	1.849	0.4710	0.7263
		For other reasons			1.134	0.647	1.987	0.6617	
		For climate and other			0.809	0.423	1.548	0.5218	
	Catholic	For climate reasons			1.786	0.642	4.969	0.2664	
		For other reasons			1.465	0.418	5.137	0.5506	
		For climate and other			0.638	0.116	3.507	0.6057	
	Other	For climate reasons			1.582	0.681	3.677	0.2864	
		For other reasons			1.550	0.546	4.399	0.4103	
		For climate and other			1.536	0.518	4.549	0.4389	
	C of E	For climate reasons	Adjusted (politics)	1094	1.202	0.764	1.892	0.4261	0.6935
		For other reasons			1.189	0.674	2.098	0.5505	
		For climate and other			0.830	0.432	1.597	0.5774	
	Catholic	For climate reasons			1.898	0.679	5.307	0.2221	
		For other reasons			1.553	0.442	5.462	0.4925	
		For climate and other			0.677	0.123	3.732	0.6542	
	Other	For climate reasons			1.607	0.689	3.748	0.2720	
		For other reasons			1.613	0.566	4.595	0.3712	
		For climate and other			1.567	0.527	4.657	0.4191	
Attendance (ref = Occasional/None)	Regular	For climate reasons	Unadjusted	1086	1.481	0.651	3.367	0.3492	0.4907
		For other reasons			1.059	0.363	3.095	0.9159	
		For climate and other			0.806	0.230	2.825	0.7361	
	Regular	For climate reasons	Adjusted	1086	1.385	0.591	3.246	0.4531	0.5588
		For other reasons			1.070	0.353	3.240	0.9054	
		For climate and other			0.723	0.197	2.649	0.6243	
	Regular	For climate reasons	Adjusted (politics)	1086	1.357	0.577	3.193	0.4847	0.5980
		For other reasons			1.094	0.359	3.333	0.8738	
		For climate and other			0.718	0.195	2.641	0.6179	
Latent class (ref = "Atheist")	Agnostic	For climate reasons	Unadjusted	1043	1.105	0.709	1.722	0.6604	0.1651
		For other reasons			0.978	0.556	1.722	0.9395	
		For climate and other			1.313	0.726	2.374	0.3686	

**Exposure**	**Exposure level**	**Outcome level (ref = No)**	**Model**	***n***	**RRR**	**LCI**	**UCI**	***p*-value**	***p* total**
	Moderately religious	For climate reasons			2.351	1.044	5.297	0.0391	
		For other reasons			1.587	0.592	4.257	0.3590	
		For climate and other			1.714	0.594	4.947	0.3189	
	Highly religious	For climate reasons			1.496	0.759	2.949	0.2441	
		For other reasons			0.643	0.240	1.724	0.3799	
		For climate and other			0.818	0.289	2.317	0.7056	
	Agnostic	For climate reasons	Adjusted	1043	0.994	0.621	1.589	0.9786	0.2064
		For other reasons			0.971	0.539	1.751	0.9222	
		For climate and other			1.209	0.651	2.245	0.5486	
	Moderately religious	For climate reasons			2.323	0.999	5.406	0.0504	
		For other reasons			1.577	0.573	4.338	0.3778	
		For climate and other			1.626	0.546	4.841	0.3823	
	Highly religious	For climate reasons			1.524	0.752	3.089	0.2420	
		For other reasons			0.648	0.237	1.776	0.3995	
		For climate and other			0.836	0.286	2.445	0.7436	
	Agnostic	For climate reasons	Adjusted (politics)	1043	1.008	0.628	1.617	0.9745	0.2233
		For other reasons			0.993	0.549	1.798	0.9827	
		For climate and other			1.231	0.660	2.293	0.5134	
	Moderately religious	For climate reasons			2.374	1.018	5.534	0.0452	
		For other reasons			1.607	0.583	4.431	0.3595	
		For climate and other			1.658	0.556	4.945	0.3644	
	Highly religious	For climate reasons			1.483	0.728	3.018	0.2774	
		For other reasons			0.653	0.237	1.797	0.4092	
		For climate and other			0.823	0.280	2.417	0.7232	
Reduced the amount of plastic used									
Belief (ref = No)	Not sure	For climate reasons	Unadjusted	1097	1.341	0.940	1.913	0.1057	0.5085
		For other reasons			1.079	0.596	1.953	0.8028	
		For climate and other			1.844	0.977	3.482	0.0592	
	Yes	For climate reasons			1.170	0.766	1.788	0.4676	
		For other reasons			1.189	0.601	2.350	0.6190	
		For climate and other			1.084	0.459	2.562	0.8543	
	Not sure	For climate reasons	Adjusted	1097	1.302	0.898	1.889	0.1642	0.6903
		For other reasons			1.084	0.590	1.995	0.7944	

Exposure	Exposure level	Outcome level (ref = No)	Model	*n*	RRR	LCI	UCI	*p*-value	*p* total
		For climate and other			1.753	0.909	3.380	0.0936	
	Yes	For climate reasons			1.145	0.736	1.783	0.5475	
		For other reasons			1.179	0.586	2.370	0.6440	
		For climate and other			1.099	0.458	2.639	0.8327	
	Not sure	For climate reasons	Adjusted (politics)	1097	1.340	0.921	1.949	0.1259	0.6418
		For other reasons			1.126	0.610	2.080	0.7047	
		For climate and other			1.769	0.915	3.421	0.0902	
	Yes	For climate reasons			1.180	0.754	1.845	0.4686	
		For other reasons			1.234	0.609	2.499	0.5600	
		For climate and other			1.120	0.464	2.703	0.8009	
Identity (combined; ref = None)	Christian	For climate reasons	Unadjusted	1096	1.144	0.828	1.580	0.4149	0.1999
		For other reasons			1.679	1.009	2.791	0.0459	
		For climate and other			0.898	0.475	1.700	0.7418	
	Christian	For climate reasons	Adjusted	1096	1.177	0.839	1.652	0.3446	0.1821
		For other reasons			1.711	1.014	2.886	0.0442	
		For climate and other			0.894	0.466	1.716	0.7361	
	Christian	For climate reasons	Adjusted (politics)	1096	1.235	0.876	1.741	0.2285	0.1108
		For other reasons			1.840	1.080	3.136	0.0250	
		For climate and other			0.911	0.472	1.756	0.7802	
Identity (separate; ref = None)	C of E	For climate reasons	Unadjusted	1096	1.127	0.775	1.638	0.5304	0.5780
		For other reasons			1.861	1.056	3.279	0.0318	
		For climate and other			0.996	0.486	2.040	0.9913	
	Catholic	For climate reasons			1.432	0.616	3.330	0.4042	
		For other reasons			0.906	0.183	4.493	0.9035	
		For climate and other			1.091	0.219	5.436	0.9155	
	Other	For climate reasons			1.060	0.577	1.948	0.8510	
		For other reasons			1.479	0.573	3.820	0.4187	
		For climate and other			0.509	0.112	2.310	0.3815	
	C of E	For climate reasons	Adjusted	1096	1.195	0.804	1.777	0.3778	0.4417
		For other reasons			1.901	1.054	3.430	0.0329	
		For climate and other			0.973	0.465	2.036	0.9413	
	Catholic	For climate reasons			1.726	0.710	4.199	0.2285	
		For other reasons			0.913	0.179	4.657	0.9131	
		For climate and other			1.423	0.274	7.389	0.6745	

**Exposure**	**Exposure level**	**Outcome level (ref = No)**	**Model**	***n***	**RRR**	**LCI**	**UCI**	***p*-value**	***p* total**
	Other	For climate reasons			0.913	0.485	1.718	0.7774	
		For other reasons			1.532	0.578	4.065	0.3911	
		For climate and other			0.474	0.102	2.207	0.3415	
	C of E	For climate reasons	Adjusted (politics)	1096	1.253	0.839	1.872	0.2710	0.3295
		For other reasons			2.048	1.123	3.735	0.0193	
		For climate and other			0.989	0.470	2.082	0.9768	
	Catholic	For climate reasons			1.857	0.758	4.550	0.1757	
		For other reasons			0.996	0.194	5.098	0.9958	
		For climate and other			1.475	0.284	7.678	0.6439	
	Other	For climate reasons			0.945	0.500	1.787	0.8625	
		For other reasons			1.626	0.610	4.336	0.3314	
		For climate and other			0.482	0.103	2.247	0.3525	
Attendance (ref = Occasional/None)	Regular	For climate reasons	Unadjusted	1088	1.321	0.670	2.605	0.4223	0.3599
		For other reasons			0.982	0.304	3.168	0.9752	
		For climate and other			0.345	0.044	2.727	0.3133	
	Regular	For climate reasons	Adjusted	1088	1.313	0.645	2.672	0.4527	0.4156
		For other reasons			1.009	0.305	3.337	0.9885	
		For climate and other			0.352	0.043	2.858	0.3286	
	Regular	For climate reasons	Adjusted (politics)	1088	1.318	0.644	2.695	0.4496	0.4119
		For other reasons			1.038	0.312	3.456	0.9520	
		For climate and other			0.348	0.043	2.840	0.3245	
Latent class (ref = "Atheist")	Agnostic	For climate reasons	Unadjusted	1045	1.360	0.915	2.022	0.1280	0.3539
		For other reasons			0.931	0.479	1.810	0.8336	
		For climate and other			2.032	1.038	3.979	0.0387	
	Moderately religious	For climate reasons			1.096	0.631	1.903	0.7458	
		For other reasons			0.784	0.299	2.060	0.6218	
		For climate and other			1.426	0.529	3.846	0.4834	
	Highly religious	For climate reasons			1.217	0.696	2.129	0.4906	
		For other reasons			0.690	0.245	1.942	0.4820	
		For climate and other			0.502	0.111	2.268	0.3702	
	Agnostic	For climate reasons	Adjusted	1045	1.365	0.898	2.073	0.1449	0.4506
		For other reasons			0.935	0.471	1.857	0.8472	
		For climate and other			2.002	0.992	4.039	0.0527	
		For climate reasons			1.013	0.566	1.815	0.9642	

**Exposure**	**Exposure level**	**Outcome level (ref = No)**	**Model**	***n***	**RRR**	**LCI**	**UCI**	***p*-value**	***p* total**
	Moderately religious	For other reasons			0.736	0.271	1.998	0.5474	
		For climate and other			1.277	0.457	3.566	0.6406	
	Highly religious	For climate reasons			1.181	0.657	2.122	0.5780	
		For other reasons			0.649	0.225	1.872	0.4237	
		For climate and other			0.499	0.108	2.305	0.3729	
	Agnostic	For climate reasons	Adjusted (politics)	1045	1.413	0.927	2.153	0.1080	0.4341
		For other reasons			0.958	0.481	1.909	0.9035	
		For climate and other			2.027	1.003	4.099	0.0492	
	Moderately religious	For climate reasons			1.043	0.581	1.873	0.8874	
		For other reasons			0.752	0.276	2.043	0.5756	
		For climate and other			1.292	0.462	3.610	0.6256	
	Highly religious	For climate reasons			1.183	0.655	2.137	0.5778	
		For other reasons			0.659	0.227	1.912	0.4430	
		For climate and other			0.499	0.107	2.319	0.3750	
Chosen sustainably sourced items									
Belief (ref = No)	Not sure	For climate reasons	Unadjusted	1094	0.861	0.638	1.162	0.3291	0.2850
		For other reasons			1.258	0.681	2.322	0.4631	
		For climate and other			1.316	0.760	2.279	0.3277	
	Yes	For climate reasons			0.889	0.619	1.277	0.5240	
		For other reasons			1.344	0.655	2.757	0.4207	
		For climate and other			0.534	0.217	1.314	0.1722	
	Not sure	For climate reasons	Adjusted	1094	0.782	0.568	1.075	0.1301	0.1670
		For other reasons			1.304	0.694	2.447	0.4094	
		For climate and other			1.295	0.731	2.292	0.3752	
	Yes	For climate reasons			0.837	0.572	1.226	0.3612	
		For other reasons			1.295	0.620	2.707	0.4918	
		For climate and other			0.530	0.212	1.322	0.1732	
	Not sure	For climate reasons	Adjusted (politics)	1094	0.815	0.590	1.125	0.2141	0.2000
		For other reasons			1.371	0.727	2.586	0.3293	
		For climate and other			1.344	0.757	2.388	0.3130	
	Yes	For climate reasons			0.872	0.593	1.283	0.4872	
		For other reasons			1.383	0.657	2.913	0.3937	
		For climate and other			0.552	0.220	1.381	0.2040	

**Exposure**	**Exposure level**	**Outcome level (ref = No)**	**Model**	***n***	**RRR**	**LCI**	**UCI**	***p*-value**	***p* total**
Identity (combined; ref = None)	Christian	For climate reasons	Unadjusted	1093	0.755	0.574	0.992	0.0435	0.0207
		For other reasons			1.250	0.725	2.158	0.4222	
		For climate and other			0.505	0.275	0.924	0.0267	
	Christian	For climate reasons	Adjusted	1093	0.732	0.548	0.978	0.0349	0.0209
		For other reasons			1.231	0.703	2.155	0.4669	
		For climate and other			0.495	0.267	0.919	0.0258	
	Christian	For climate reasons	Adjusted (politics)	1093	0.777	0.579	1.042	0.0921	0.0370
		For other reasons			1.345	0.760	2.380	0.3095	
		For climate and other			0.518	0.278	0.965	0.0384	
Identity (separate; ref = None)	C of E	For climate reasons	Unadjusted	1093	0.713	0.523	0.973	0.0327	0.0630
		For other reasons			0.832	0.418	1.657	0.6013	
		For climate and other			0.420	0.200	0.880	0.0215	
	Catholic	For climate reasons			1.009	0.501	2.033	0.9793	
		For other reasons			2.100	0.656	6.721	0.2111	
		For climate and other			0.707	0.156	3.197	0.6520	
	Other	For climate reasons			0.785	0.456	1.351	0.3820	
		For other reasons			2.450	1.070	5.613	0.0341	
		For climate and other			0.733	0.247	2.178	0.5757	
	C of E	For climate reasons	Adjusted	1093	0.699	0.502	0.974	0.0346	0.0287
		For other reasons			0.769	0.378	1.567	0.4701	
		For climate and other			0.412	0.193	0.878	0.0216	
	Catholic	For climate reasons			1.149	0.545	2.422	0.7147	
		For other reasons			2.090	0.628	6.958	0.2295	
		For climate and other			0.674	0.141	3.214	0.6207	
	Other	For climate reasons			0.679	0.383	1.202	0.1838	
		For other reasons			2.748	1.155	6.538	0.0223	
		For climate and other			0.734	0.240	2.249	0.5883	
	C of E	For climate reasons	Adjusted (politics)	1093	0.741	0.529	1.037	0.0804	0.0441
		For other reasons			0.838	0.407	1.725	0.6317	
		For climate and other			0.429	0.200	0.919	0.0294	
	Catholic	For climate reasons			1.244	0.586	2.645	0.5697	
		For other reasons			2.288	0.680	7.695	0.1810	
		For climate and other			0.718	0.150	3.438	0.6789	
	Other	For climate reasons			0.717	0.403	1.275	0.2573	

Exposure	Exposure level	Outcome level (ref = No)	Model	*n*	RRR	LCI	UCI	*p*-value	*p* total
		For other reasons			2.942	1.226	7.058	0.0156	
		For climate and other			0.763	0.248	2.347	0.6374	
Attendance (ref = Occasional/None)	Regular	For climate reasons	Unadjusted	1086	0.859	0.493	1.496	0.5907	0.9071
		For other reasons			0.795	0.233	2.716	0.7147	
		For climate and other			0.700	0.206	2.385	0.5688	
	Regular	For climate reasons	Adjusted	1086	0.877	0.488	1.576	0.6599	0.9744
		For other reasons			0.858	0.241	3.055	0.8128	
		For climate and other			0.869	0.246	3.068	0.8274	
	Regular	For climate reasons	Adjusted (politics)	1086	0.899	0.497	1.625	0.7235	0.9873
		For other reasons			0.906	0.253	3.249	0.8798	
		For climate and other			0.896	0.252	3.183	0.8656	
Latent class (ref = "Atheist")	Agnostic	For climate reasons	Unadjusted	1043	0.910	0.654	1.265	0.5730	0.8740
		For other reasons			1.112	0.583	2.120	0.7479	
		For climate and other			0.960	0.509	1.812	0.8999	
	Moderately religious	For climate reasons			0.991	0.612	1.607	0.9722	
		For other reasons			1.368	0.565	3.311	0.4877	
		For climate and other			0.844	0.312	2.279	0.7374	
	Highly religious	For climate reasons			0.957	0.595	1.537	0.8541	
		For other reasons			0.368	0.085	1.595	0.1817	
		For climate and other			0.636	0.215	1.880	0.4134	
	Agnostic	For climate reasons	Adjusted	1043	0.838	0.588	1.194	0.3272	0.8241
		For other reasons			1.171	0.600	2.285	0.6431	
		For climate and other			0.931	0.481	1.803	0.8330	
	Moderately religious	For climate reasons			0.886	0.531	1.479	0.6441	
		For other reasons			1.343	0.537	3.354	0.5283	
		For climate and other			0.713	0.257	1.975	0.5151	
	Highly religious	For climate reasons			0.936	0.566	1.545	0.7948	
		For other reasons			0.381	0.086	1.684	0.2031	
		For climate and other			0.747	0.247	2.262	0.6059	
	Agnostic	For climate reasons	Adjusted (politics)	1043	0.876	0.613	1.253	0.4685	0.8676
		For other reasons			1.227	0.626	2.405	0.5505	
		For climate and other			0.961	0.495	1.866	0.9073	
	Moderately religious	For climate reasons			0.921	0.549	1.544	0.7542	
		For other reasons			1.386	0.553	3.477	0.4861	

Exposure	Exposure level	Outcome level (ref = No)	Model	*n*	RRR	LCI	UCI	*p*-value	*p* total
		For climate and other			0.734	0.264	2.039	0.5531	
	Highly religious	For climate reasons			0.942	0.567	1.567	0.8186	
		For other reasons			0.394	0.089	1.750	0.2207	
		For climate and other			0.757	0.249	2.301	0.6231	
Improved home insulation									
Belief (ref = No)	Not sure	For climate reasons	Unadjusted	1099	1.187	0.633	2.225	0.5935	0.7625
		For other reasons			0.786	0.455	1.357	0.3873	
		For climate and other			1.434	0.579	3.552	0.4360	
	Yes	For climate reasons			1.490	0.726	3.057	0.2769	
		For other reasons			1.133	0.619	2.073	0.6854	
		For climate and other			1.637	0.567	4.723	0.3623	
	Not sure	For climate reasons	Adjusted	1099	1.208	0.637	2.290	0.5628	0.7494
		For other reasons			0.799	0.454	1.407	0.4376	
		For climate and other			1.344	0.532	3.390	0.5318	
	Yes	For climate reasons			1.480	0.715	3.066	0.2911	
		For other reasons			1.234	0.661	2.305	0.5086	
		For climate and other			1.746	0.592	5.152	0.3128	
	Not sure	For climate reasons	Adjusted (politics)	1099	1.319	0.689	2.527	0.4037	0.6745
		For other reasons			0.774	0.439	1.366	0.3770	
		For climate and other			1.373	0.542	3.475	0.5040	
	Yes	For climate reasons			1.567	0.747	3.288	0.2345	
		For other reasons			1.167	0.622	2.189	0.6310	
		For climate and other			1.774	0.598	5.263	0.3012	
Identity (combined; ref = None)	Christian	For climate reasons	Unadjusted	1098	1.063	0.596	1.894	0.8362	0.7441
		For other reasons			1.265	0.797	2.007	0.3187	
		For climate and other			1.263	0.552	2.891	0.5810	
	Christian	For climate reasons	Adjusted	1098	1.028	0.572	1.847	0.9257	0.7282
		For other reasons			1.287	0.799	2.073	0.2994	
		For climate and other			1.269	0.545	2.955	0.5808	
	Christian	For climate reasons	Adjusted (politics)	1098	1.122	0.617	2.040	0.7068	0.7685
		For other reasons			1.241	0.766	2.012	0.3806	
		For climate and other			1.290	0.553	3.009	0.5564	
	C of E	For climate reasons	Unadjusted	1098	1.154	0.602	2.210	0.6668	0.5316

Exposure	Exposure level	Outcome level (ref = No)	Model	*n*	RRR	LCI	UCI	*p*-value	*p* total
		For other reasons			1.533	0.930	2.526	0.0939	
		For climate and other			1.265	0.488	3.277	0.6287	
	Catholic	For climate reasons			0.471	0.063	3.527	0.4634	
		For other reasons			0.976	0.291	3.271	0.9684	
		For climate and other			NA	NA	NA	NA	
	Other	For climate reasons			1.130	0.390	3.274	0.8215	
		For other reasons			0.585	0.178	1.928	0.3786	
		For climate and other			2.012	0.570	7.102	0.2776	
Identity (separate; ref = None)	C of E	For climate reasons	Adjusted	1098	1.103	0.567	2.144	0.7725	0.5282
		For other reasons			1.630	0.963	2.761	0.0690	
		For climate and other			1.246	0.467	3.326	0.6608	
	Catholic	For climate reasons			0.467	0.061	3.573	0.4630	
		For other reasons			0.842	0.239	2.961	0.7886	
		For climate and other			NA	NA	NA	NA	
	Other	For climate reasons			1.118	0.380	3.294	0.8393	
		For other reasons			0.590	0.175	1.997	0.3966	
		For climate and other			1.995	0.540	7.378	0.3004	
	C of E	For climate reasons	Adjusted (politics)	1098	1.200	0.609	2.365	0.5988	0.5601
		For other reasons			1.563	0.917	2.664	0.1008	
		For climate and other			1.284	0.480	3.432	0.6183	
	Catholic	For climate reasons			0.539	0.070	4.159	0.5530	
		For other reasons			0.858	0.242	3.043	0.8122	
		For climate and other			NA	NA	NA	NA	
	Other	For climate reasons			1.204	0.406	3.574	0.7377	
		For other reasons			0.561	0.165	1.908	0.3548	
		For climate and other			2.013	0.541	7.485	0.2966	
Attendance (ref = Occasional/None)	Regular	For climate reasons	Unadjusted	1090	0.948	0.287	3.136	0.9305	0.4953
		For other reasons			0.399	0.095	1.666	0.2077	
		For climate and other			1.457	0.334	6.346	0.6165	
	Regular	For climate reasons	Adjusted	1090	0.945	0.278	3.215	0.9278	0.6128
		For other reasons			0.467	0.108	2.013	0.3070	
		For climate and other			1.755	0.380	8.094	0.4710	
	Regular	For climate reasons	Adjusted (politics)	1090	0.943	0.274	3.245	0.9253	0.5070
		For other reasons			0.420	0.097	1.822	0.2466	

**Exposure**	**Exposure level**	**Outcome level (ref = No)**	**Model**	***n***	**RRR**	**LCI**	**UCI**	***p*-value**	***p* total**
		For climate and other			1.872	0.401	8.745	0.4252	
Latent class (ref = "Atheist")	Agnostic	For climate reasons	Unadjusted	1047	1.125	0.551	2.297	0.7456	0.4138
		For other reasons			1.120	0.652	1.923	0.6814	
		For climate and other			0.741	0.245	2.246	0.5969	
	Moderately religious	For climate reasons			1.328	0.498	3.541	0.5702	
		For other reasons			1.309	0.620	2.766	0.4805	
		For climate and other			0.482	0.063	3.688	0.4819	
	Highly religious	For climate reasons			1.572	0.632	3.915	0.3308	
		For other reasons			0.287	0.069	1.203	0.0878	
		For climate and other			1.898	0.617	5.836	0.2634	
	Agnostic	For climate reasons	Adjusted	1047	1.215	0.586	2.522	0.6005	0.4317
		For other reasons			1.241	0.706	2.179	0.4531	
		For climate and other			0.706	0.228	2.187	0.5465	
	Moderately religious	For climate reasons			1.308	0.477	3.587	0.6021	
		For other reasons			1.360	0.621	2.982	0.4423	
		For climate and other			0.551	0.070	4.333	0.5712	
	Highly religious	For climate reasons			1.514	0.593	3.864	0.3855	
		For other reasons			0.301	0.070	1.286	0.1052	
		For climate and other			2.005	0.627	6.414	0.2412	
	Agnostic	For climate reasons	Adjusted (politics)	1047	1.311	0.626	2.745	0.4730	0.3473
		For other reasons			1.191	0.677	2.095	0.5448	
		For climate and other			0.742	0.238	2.314	0.6074	
	Moderately religious	For climate reasons			1.374	0.499	3.786	0.5391	
		For other reasons			1.389	0.634	3.043	0.4120	
		For climate and other			0.528	0.066	4.207	0.5461	
	Highly religious	For climate reasons			1.491	0.576	3.859	0.4107	
		For other reasons			0.276	0.064	1.184	0.0831	
		For climate and other			2.186	0.674	7.093	0.1929	
Started growing vegetables									
Belief (ref = No)	Not sure	For climate reasons	Unadjusted	1096	0.956	0.529	1.727	0.8808	0.4023
		For other reasons			1.168	0.797	1.711	0.4258	
		For climate and other			0.822	0.376	1.799	0.6241	
	Yes	For climate reasons			0.797	0.363	1.750	0.5720	

Exposure	Exposure level	Outcome level (ref = No)	Model	*n*	RRR	LCI	UCI	*p*-value	*p* total
		For other reasons			1.521	0.986	2.345	0.0579	
		For climate and other			0.486	0.144	1.640	0.2447	
	Not sure	For climate reasons	Adjusted	1096	0.935	0.507	1.722	0.8283	0.4283
		For other reasons			1.155	0.780	1.710	0.4716	
		For climate and other			0.805	0.357	1.813	0.6002	
	Yes	For climate reasons			0.801	0.358	1.794	0.5901	
		For other reasons			1.539	0.989	2.396	0.0562	
		For climate and other			0.505	0.145	1.757	0.2827	
	Not sure	For climate reasons	Adjusted (politics)	1096	0.921	0.497	1.707	0.7933	0.3870
		For other reasons			1.180	0.795	1.752	0.4112	
		For climate and other			0.877	0.386	1.995	0.7547	
	Yes	For climate reasons			0.768	0.340	1.736	0.5256	
		For other reasons			1.587	1.016	2.479	0.0423	
		For climate and other			0.530	0.151	1.860	0.3214	
Identity (combined; ref = None)	Christian	For climate reasons	Unadjusted	1095	0.889	0.505	1.564	0.6827	0.2020
		For other reasons			1.384	0.987	1.941	0.0598	
		For climate and other			0.757	0.351	1.633	0.4779	
	Christian	For climate reasons	Adjusted	1095	0.892	0.499	1.595	0.7007	0.2473
		For other reasons			1.388	0.982	1.961	0.0635	
		For climate and other			0.828	0.374	1.836	0.6427	
	Christian	For climate reasons	Adjusted (politics)	1095	0.881	0.489	1.588	0.6730	0.2145
		For other reasons			1.422	1.002	2.016	0.0485	
		For climate and other			0.884	0.395	1.978	0.7647	
Identity (separate; ref = None)	C of E	For climate reasons	Unadjusted	1095	0.852	0.441	1.644	0.6322	0.4632
		For other reasons			1.335	0.908	1.965	0.1422	
		For climate and other			0.484	0.167	1.401	0.1808	
	Catholic	For climate reasons			0.411	0.055	3.087	0.3877	
		For other reasons			1.205	0.516	2.812	0.6665	
		For climate and other			1.402	0.318	6.176	0.6549	
	Other	For climate reasons			1.342	0.509	3.543	0.5521	
		For other reasons			1.684	0.908	3.122	0.0981	
		For climate and other			1.372	0.401	4.694	0.6145	
	C of E	For climate reasons	Adjusted	1095	0.854	0.433	1.683	0.6481	0.6348
		For other reasons			1.325	0.889	1.974	0.1672	

**Exposure**	**Exposure level**	**Outcome level (ref = No)**	**Model**	***n***	**RRR**	**LCI**	**UCI**	***p*-value**	***p* total**
		For climate and other			0.579	0.193	1.733	0.3283	
	Catholic	For climate reasons			0.436	0.055	3.433	0.4306	
		For other reasons			1.162	0.486	2.779	0.7358	
		For climate and other			1.369	0.281	6.661	0.6971	
	Other	For climate reasons			1.286	0.474	3.490	0.6217	
		For other reasons			1.774	0.940	3.349	0.0768	
		For climate and other			1.202	0.329	4.394	0.7806	
	C of E	For climate reasons	Adjusted (politics)	1095	0.833	0.419	1.656	0.6029	0.5964
		For other reasons			1.363	0.911	2.038	0.1318	
		For climate and other			0.616	0.205	1.856	0.3897	
	Catholic	For climate reasons			0.436	0.054	3.532	0.4371	
		For other reasons			1.169	0.488	2.801	0.7264	
		For climate and other			1.381	0.285	6.682	0.6884	
	Other	For climate reasons			1.286	0.470	3.518	0.6239	
		For other reasons			1.808	0.955	3.421	0.0688	
		For climate and other			1.335	0.363	4.911	0.6636	
Attendance (ref = Occasional/None)	Regular	For climate reasons	Unadjusted	1087	1.650	0.627	4.337	0.3102	0.5421
		For other reasons			1.368	0.703	2.659	0.3561	
		For climate and other			0.537	0.072	4.020	0.5452	
	Regular	For climate reasons	Adjusted	1087	1.701	0.618	4.677	0.3035	0.4020
		For other reasons			1.541	0.774	3.069	0.2189	
		For climate and other			0.474	0.059	3.782	0.4807	
	Regular	For climate reasons	Adjusted (politics)	1087	1.549	0.556	4.310	0.4024	0.4537
		For other reasons			1.600	0.800	3.198	0.1837	
		For climate and other			0.542	0.067	4.357	0.5648	
Latent class (ref = "Atheist")	Agnostic	For climate reasons	Unadjusted	1044	0.881	0.439	1.767	0.7214	0.5666
		For other reasons			1.126	0.745	1.702	0.5728	
		For climate and other			0.864	0.366	2.043	0.7400	
	Moderately religious	For climate reasons			0.583	0.175	1.943	0.3796	
		For other reasons			1.079	0.593	1.964	0.8044	
		For climate and other			0.599	0.139	2.592	0.4931	
	Highly religious	For climate reasons			1.682	0.716	3.953	0.2330	
		For other reasons			1.867	1.081	3.226	0.0252	
		For climate and other			0.741	0.170	3.220	0.6890	

Exposure	Exposure level	Outcome level (ref = No)	Model	*n*	RRR	LCI	UCI	*p*-value	*p* total
	Agnostic	For climate reasons	Adjusted	1044	0.800	0.391	1.639	0.5428	0.5181
		For other reasons			1.106	0.722	1.694	0.6427	
		For climate and other			0.812	0.329	2.003	0.6507	
	Moderately religious	For climate reasons			0.608	0.177	2.092	0.4305	
		For other reasons			1.100	0.595	2.035	0.7615	
		For climate and other			0.494	0.109	2.225	0.3581	
	Highly religious	For climate reasons			1.726	0.709	4.201	0.2288	
		For other reasons			1.895	1.078	3.329	0.0263	
		For climate and other			0.752	0.164	3.437	0.7130	
	Agnostic	For climate reasons	Adjusted (politics)	1044	0.793	0.385	1.633	0.5288	0.5322
		For other reasons			1.134	0.738	1.741	0.5659	
		For climate and other			0.902	0.361	2.255	0.8260	
	Moderately religious	For climate reasons			0.649	0.189	2.231	0.4924	
		For other reasons			1.104	0.596	2.045	0.7531	
		For climate and other			0.460	0.100	2.118	0.3188	
	Highly religious	For climate reasons			1.574	0.639	3.876	0.3242	
		For other reasons			1.958	1.110	3.456	0.0204	
		For climate and other			0.835	0.181	3.846	0.8171	
Planted trees									
Belief (ref = No)	Not sure	For climate reasons	Unadjusted	1095	0.543	0.283	1.040	0.0655	0.3415
		For other reasons			1.356	0.838	2.194	0.2143	
		For climate and other			1.395	0.596	3.268	0.4434	
	Yes	For climate reasons			0.784	0.387	1.589	0.4995	
		For other reasons			1.332	0.743	2.389	0.3353	
		For climate and other			1.075	0.348	3.318	0.8996	
	Not sure	For climate reasons	Adjusted	1095	0.553	0.284	1.077	0.0817	0.4121
		For other reasons			1.356	0.821	2.237	0.2340	
		For climate and other			1.394	0.577	3.364	0.4605	
	Yes	For climate reasons			0.763	0.371	1.570	0.4627	
		For other reasons			1.301	0.709	2.386	0.3955	
		For climate and other			1.049	0.330	3.338	0.9351	
	Not sure	For climate reasons	Adjusted (politics)	1095	0.558	0.285	1.092	0.0885	0.4275
		For other reasons			1.335	0.807	2.210	0.2604	

Exposure	Exposure level	Outcome level (ref = No)	Model	*n*	RRR	LCI	UCI	*p*-value	*p* total
		For climate and other			1.502	0.614	3.672	0.3724	
	Yes	For climate reasons			0.763	0.369	1.576	0.4643	
		For other reasons			1.286	0.699	2.367	0.4184	
		For climate and other			1.203	0.373	3.877	0.7575	
Identity (combined; ref = None)	Christian	For climate reasons	Unadjusted	1094	0.781	0.449	1.360	0.3823	0.1548
		For other reasons			1.571	1.019	2.422	0.0406	
		For climate and other			0.950	0.411	2.197	0.9049	
	Christian	For climate reasons	Adjusted	1094	0.762	0.432	1.343	0.3467	0.1908
		For other reasons			1.535	0.980	2.402	0.0611	
		For climate and other			0.925	0.392	2.182	0.8583	
	Christian	For climate reasons	Adjusted (politics)	1094	0.774	0.437	1.372	0.3806	0.2337
		For other reasons			1.513	0.963	2.378	0.0724	
		For climate and other			0.984	0.412	2.349	0.9711	
Identity (separate; ref = None)	C of E	For climate reasons	Unadjusted	1094	0.780	0.408	1.492	0.4520	0.1460
		For other reasons			1.689	1.043	2.736	0.0332	
		For climate and other			1.245	0.515	3.007	0.6263	
	Catholic	For climate reasons			1.002	0.298	3.363	0.9979	
		For other reasons			0.310	0.042	2.304	0.2525	
		For climate and other			NA	NA	NA	NA	
	Other	For climate reasons			0.644	0.195	2.128	0.4704	
		For other reasons			1.993	0.964	4.120	0.0628	
		For climate and other			0.587	0.077	4.470	0.6074	
	C of E	For climate reasons	Adjusted	1094	0.798	0.410	1.552	0.5058	0.1348
		For other reasons			1.611	0.969	2.676	0.0659	
		For climate and other			1.143	0.457	2.860	0.7748	
	Catholic	For climate reasons			0.952	0.272	3.331	0.9383	
		For other reasons			0.261	0.034	2.025	0.1989	
		For climate and other			NA	NA	NA	NA	
	Other	For climate reasons			0.552	0.163	1.866	0.3393	
		For other reasons			2.276	1.058	4.895	0.0353	
		For climate and other			0.703	0.090	5.506	0.7370	
	C of E	For climate reasons	Adjusted (politics)	1094	0.810	0.414	1.584	0.5379	0.1383
		For other reasons			1.590	0.952	2.654	0.0762	
		For climate and other			1.252	0.494	3.176	0.6355	

**Exposure**	**Exposure level**	**Outcome level (ref = No)**	**Model**	***n***	**RRR**	**LCI**	**UCI**	***p*-value**	***p* total**
	Catholic	For climate reasons			0.993	0.283	3.491	0.9917	
		For other reasons			0.257	0.033	1.984	0.1923	
		For climate and other			NA	NA	NA	NA	
	Other	For climate reasons			0.555	0.164	1.879	0.3440	
		For other reasons			2.255	1.047	4.861	0.0379	
		For climate and other			0.716	0.090	5.701	0.7520	
Attendance (ref = Occasional/None)	Regular	For climate reasons	Unadjusted	1086	0.507	0.121	2.131	0.3539	0.1369
		For other reasons			1.586	0.727	3.457	0.2462	
		For climate and other			NA	NA	NA	NA	
	Regular	For climate reasons	Adjusted	1086	0.441	0.101	1.917	0.2748	0.0995
		For other reasons			1.794	0.783	4.110	0.1668	
		For climate and other			NA	NA	NA	NA	
	Regular	For climate reasons	Adjusted (politics)	1086	0.431	0.098	1.890	0.2647	0.1134
		For other reasons			1.808	0.784	4.168	0.1645	
		For climate and other			NA	NA	NA	NA	
Latent class (ref = "Atheist")	Agnostic	For climate reasons	Unadjusted	1044	0.501	0.233	1.081	0.0781	0.1134
		For other reasons			1.167	0.685	1.988	0.5701	
		For climate and other			2.104	0.919	4.819	0.0786	
	Moderately religious	For climate reasons			1.124	0.489	2.583	0.7822	
		For other reasons			0.997	0.436	2.278	0.9945	
		For climate and other			1.618	0.454	5.768	0.4583	
	Highly religious	For climate reasons			0.642	0.225	1.836	0.4088	
		For other reasons			1.425	0.694	2.926	0.3351	
		For climate and other			NA	NA	NA	NA	
	Agnostic	For climate reasons	Adjusted	1044	0.481	0.219	1.058	0.0688	0.1374
		For other reasons			1.225	0.701	2.143	0.4762	
		For climate and other			2.473	1.032	5.928	0.0423	
	Moderately religious	For climate reasons			1.065	0.447	2.536	0.8864	
		For other reasons			0.839	0.351	2.004	0.6930	
		For climate and other			1.496	0.397	5.641	0.5520	
	Highly religious	For climate reasons			0.585	0.199	1.718	0.3291	
		For other reasons			1.426	0.671	3.034	0.3565	
		For climate and other			NA	NA	NA	NA	
	Agnostic	For climate reasons		1044	0.484	0.219	1.067	0.0721	0.0772

Exposure	Exposure level	Outcome level (ref = No)	Model	*n*	RRR	LCI	UCI	*p*-value	*p* total
		For other reasons	Adjusted (politics)		1.206	0.688	2.114	0.5141	
		For climate and other			2.800	1.141	6.873	0.0246	
	Moderately religious	For climate reasons			1.082	0.454	2.578	0.8588	
		For other reasons			0.834	0.349	1.993	0.6825	
		For climate and other			1.573	0.418	5.921	0.5030	
	Highly religious	For climate reasons			0.566	0.191	1.679	0.3050	
		For other reasons			1.412	0.661	3.018	0.3728	
		For climate and other			NA	NA	NA	NA	
Avoided organisations that support fossil fuels									
Belief (ref = No)	Not sure	For climate reasons	Unadjusted	1097	0.647	0.421	0.996	0.0478	0.0746
		For other reasons			1.034	0.256	4.167	0.9629	
		For climate and other			1.608	0.592	4.367	0.3514	
	Yes	For climate reasons			0.632	0.370	1.077	0.0917	
		For other reasons			NA	NA	NA	NA	
		For climate and other			0.386	0.049	3.071	0.3682	
	Not sure	For climate reasons	Adjusted	1097	0.631	0.406	0.981	0.0410	0.0443
		For other reasons			1.287	0.290	5.708	0.7398	
		For climate and other			1.492	0.512	4.343	0.4631	
	Yes	For climate reasons			0.590	0.342	1.020	0.0590	
		For other reasons			NA	NA	NA	NA	
		For climate and other			0.284	0.030	2.664	0.2707	
	Not sure	For climate reasons	Adjusted (politics)	1097	0.680	0.434	1.064	0.0915	0.0722
		For other reasons			1.644	0.350	7.718	0.5287	
		For climate and other			2.423	0.710	8.269	0.1578	
	Yes	For climate reasons			0.621	0.357	1.082	0.0925	
		For other reasons			NA	NA	NA	NA	
		For climate and other			0.357	0.032	3.937	0.4007	
Identity (combined; ref = None)	Christian	For climate reasons	Unadjusted	1096	0.493	0.321	0.757	0.0012	0.0063
		For other reasons			1.618	0.431	6.070	0.4754	
		For climate and other			0.843	0.294	2.414	0.7502	
	Christian	For climate reasons	Adjusted	1096	0.483	0.312	0.747	0.0011	0.0050
		For other reasons			1.811	0.438	7.482	0.4118	
		For climate and other			0.725	0.236	2.233	0.5756	

Exposure	Exposure level	Outcome level (ref = No)	Model	*n*	RRR	LCI	UCI	*p*-value	*p* total
	Christian	For climate reasons	Adjusted (politics)	1096	0.527	0.338	0.821	0.0046	0.0164
		For other reasons			2.319	0.531	10.125	0.2635	
		For climate and other			1.115	0.308	4.034	0.8683	
Identity (separate; ref = None)	C of E	For climate reasons	Unadjusted	1096	0.444	0.264	0.747	0.0022	0.0767
		For other reasons			1.763	0.418	7.441	0.4402	
		For climate and other			0.490	0.109	2.206	0.3525	
	Catholic	For climate reasons			0.593	0.207	1.698	0.3300	
		For other reasons			NA	NA	NA	NA	
		For climate and other			1.469	0.186	11.621	0.7155	
	Other	For climate reasons			0.605	0.270	1.356	0.2222	
		For other reasons			2.057	0.236	17.897	0.5135	
		For climate and other			1.714	0.375	7.838	0.4873	
	C of E	For climate reasons	Adjusted	1096	0.444	0.261	0.755	0.0027	0.0728
		For other reasons			2.598	0.544	12.397	0.2312	
		For climate and other			0.487	0.099	2.395	0.3763	
	Catholic	For climate reasons			0.617	0.209	1.819	0.3814	
		For other reasons			NA	NA	NA	NA	
		For climate and other			1.261	0.120	13.238	0.8465	
	Other	For climate reasons			0.537	0.235	1.227	0.1403	
		For other reasons			1.464	0.130	16.436	0.7575	
		For climate and other			1.043	0.193	5.625	0.9610	
	C of E	For climate reasons	Adjusted (politics)	1096	0.485	0.283	0.831	0.0084	0.1178
		For other reasons			3.878	0.721	20.852	0.1143	
		For climate and other			0.850	0.146	4.963	0.8565	
	Catholic	For climate reasons			0.698	0.235	2.077	0.5186	
		For other reasons			NA	NA	NA	NA	
		For climate and other			0.660	0.041	10.633	0.7692	
	Other	For climate reasons			0.568	0.247	1.307	0.1834	
		For other reasons			2.131	0.167	27.146	0.5601	
		For climate and other			2.119	0.337	13.346	0.4237	
Attendance (ref = Occasional/None)	Regular	For climate reasons	Unadjusted	1088	1.290	0.638	2.607	0.4781	0.3321
		For other reasons			NA	NA	NA	NA	
		For climate and other			NA	NA	NA	NA	
	Regular	For climate reasons	Adjusted	1088	1.179	0.569	2.443	0.6574	0.1731

Exposure	Exposure level	Outcome level (ref = No)	Model	*n*	RRR	LCI	UCI	*p*-value	*p* total
		For other reasons			NA	NA	NA	NA	
		For climate and other			NA	NA	NA	NA	
	Regular	For climate reasons	Adjusted (politics)	1088	1.245	0.595	2.603	0.5604	0.4309
		For other reasons			NA	NA	NA	NA	
		For climate and other			NA	NA	NA	NA	
Latent class (ref = "Atheist")	Agnostic	For climate reasons	Unadjusted	1045	0.834	0.527	1.320	0.4389	0.5932
		For other reasons			2.155	0.478	9.716	0.3178	
		For climate and other			0.442	0.099	1.977	0.2854	
	Moderately religious	For climate reasons			0.599	0.280	1.281	0.1861	
		For other reasons			NA	NA	NA	NA	
		For climate and other			0.536	0.069	4.150	0.5500	
	Highly religious	For climate reasons			0.892	0.456	1.746	0.7388	
		For other reasons			NA	NA	NA	NA	
		For climate and other			0.580	0.075	4.501	0.6024	
	Agnostic	For climate reasons	Adjusted	1045	0.807	0.503	1.295	0.3740	0.2532
		For other reasons			4.020	0.677	23.873	0.1259	
		For climate and other			0.362	0.076	1.722	0.2014	
	Moderately religious	For climate reasons			0.528	0.242	1.151	0.1080	
		For other reasons			NA	NA	NA	NA	
		For climate and other			0.474	0.055	4.050	0.4951	
	Highly religious	For climate reasons			0.866	0.433	1.730	0.6828	
		For other reasons			NA	NA	NA	NA	
		For climate and other			0.430	0.046	4.048	0.4607	
	Agnostic	For climate reasons	Adjusted (politics)	1045	0.870	0.538	1.407	0.5714	0.3014
		For other reasons			6.329	0.919	43.585	0.0609	
		For climate and other			0.431	0.076	2.455	0.3430	
	Moderately religious	For climate reasons			0.547	0.249	1.202	0.1331	
		For other reasons			NA	NA	NA	NA	
		For climate and other			0.432	0.041	4.553	0.4850	
	Highly religious	For climate reasons			0.894	0.443	1.804	0.7541	
		For other reasons			NA	NA	NA	NA	
		For climate and other			0.725	0.060	8.784	0.8003	
Chosen to have fewer or no children									

Exposure	Exposure level	Outcome level (ref = No)	Model	*n*	RRR	LCI	UCI	*p*-value	*p* total
Belief (ref = No)	Not sure	For climate reasons	Unadjusted	1093	0.631	0.295	1.353	0.2370	0.0038
		For other reasons			0.740	0.410	1.336	0.3175	
		For climate and other			0.593	0.252	1.395	0.2313	
	Yes	For climate reasons			0.239	0.056	1.012	0.0518	
		For other reasons			1.023	0.537	1.947	0.9459	
		For climate and other			NA	NA	NA	NA	
	Not sure	For climate reasons	Adjusted	1093	0.600	0.273	1.319	0.2038	0.0031
		For other reasons			0.759	0.414	1.391	0.3715	
		For climate and other			0.623	0.257	1.510	0.2951	
	Yes	For climate reasons			0.219	0.050	0.950	0.0426	
		For other reasons			0.981	0.507	1.896	0.9544	
		For climate and other			NA	NA	NA	NA	
	Not sure	For climate reasons	Adjusted (politics)	1093	0.632	0.285	1.400	0.2581	0.0074
		For other reasons			0.817	0.441	1.511	0.5188	
		For climate and other			0.675	0.274	1.664	0.3936	
	Yes	For climate reasons			0.227	0.052	0.994	0.0491	
		For other reasons			1.055	0.540	2.064	0.8750	
		For climate and other			NA	NA	NA	NA	
Identity (combined; ref = None)	Christian	For climate reasons	Unadjusted	1092	0.353	0.147	0.849	0.0201	<0.0001
		For other reasons			0.808	0.478	1.365	0.4248	
		For climate and other			0.067	0.009	0.491	0.0079	
	Christian	For climate reasons	Adjusted	1092	0.357	0.146	0.874	0.0241	0.0001
		For other reasons			0.834	0.488	1.425	0.5062	
		For climate and other			0.073	0.010	0.548	0.0109	
	Christian	For climate reasons	Adjusted (politics)	1092	0.386	0.156	0.954	0.0392	0.0009
		For other reasons			0.928	0.537	1.603	0.7888	
		For climate and other			0.085	0.011	0.647	0.0172	
Identity (separate; ref = None)	C of E	For climate reasons	Unadjusted	1092	0.340	0.119	0.968	0.0433	0.0003
		For other reasons			0.666	0.349	1.272	0.2183	
		For climate and other			NA	NA	NA	NA	
	Catholic	For climate reasons			NA	NA	NA	NA	
		For other reasons			0.658	0.155	2.805	0.5720	
		For climate and other			NA	NA	NA	NA	
	Other	For climate reasons			0.620	0.145	2.644	0.5184	

**Exposure**	**Exposure level**	**Outcome level (ref = No)**	**Model**	***n***	**RRR**	**LCI**	**UCI**	***p*-value**	***p* total**
		For other reasons			1.418	0.617	3.260	0.4106	
		For climate and other			0.351	0.047	2.620	0.3075	
	C of E	For climate reasons	Adjusted	1092	0.367	0.126	1.069	0.0660	0.0019
		For other reasons			0.749	0.386	1.451	0.3912	
		For climate and other			NA	NA	NA	NA	
	Catholic	For climate reasons			NA	NA	NA	NA	
		For other reasons			0.525	0.118	2.341	0.3986	
		For climate and other			NA	NA	NA	NA	
	Other	For climate reasons			0.533	0.120	2.373	0.4090	
		For other reasons			1.305	0.550	3.099	0.5462	
		For climate and other			0.265	0.033	2.120	0.2106	
	C of E	For climate reasons	Adjusted (politics)	1092	0.400	0.136	1.175	0.0956	0.0080
		For other reasons			0.837	0.427	1.641	0.6047	
		For climate and other			NA	NA	NA	NA	
	Catholic	For climate reasons			NA	NA	NA	NA	
		For other reasons			0.578	0.127	2.628	0.4779	
		For climate and other			NA	NA	NA	NA	
	Other	For climate reasons			0.561	0.125	2.511	0.4497	
		For other reasons			1.441	0.602	3.446	0.4120	
		For climate and other			0.322	0.040	2.575	0.2856	
Attendance (ref = Occasional/None)	Regular	For climate reasons	Unadjusted	1084	0.469	0.063	3.486	0.4594	0.0994
		For other reasons			1.890	0.825	4.332	0.1326	
		For climate and other			NA	NA	NA	NA	
	Regular	For climate reasons	Adjusted	1084	0.445	0.058	3.422	0.4367	0.0702
		For other reasons			1.757	0.731	4.221	0.2075	
		For climate and other			NA	NA	NA	NA	
	Regular	For climate reasons	Adjusted (politics)	1084	0.467	0.060	3.614	0.4660	0.0896
		For other reasons			1.863	0.767	4.530	0.1697	
		For climate and other			NA	NA	NA	NA	
Latent class (ref = "Atheist")	Agnostic	For climate reasons	Unadjusted	1043	0.587	0.240	1.435	0.2425	0.0113
		For other reasons			0.923	0.494	1.725	0.8023	
		For climate and other			0.827	0.351	1.950	0.6645	
	Moderately religious	For climate reasons			0.477	0.112	2.036	0.3173	
		For other reasons			0.804	0.309	2.089	0.6541	

Exposure	Exposure level	Outcome level (ref = No)	Model	*n*	RRR	LCI	UCI	*p*-value	*p* total
		For climate and other			NA	NA	NA	NA	
	Highly religious	For climate reasons			NA	NA	NA	NA	
		For other reasons			1.336	0.606	2.948	0.4729	
		For climate and other			NA	NA	NA	NA	
	Agnostic	For climate reasons	Adjusted	1043	0.550	0.218	1.388	0.2057	0.0069
		For other reasons			0.989	0.519	1.885	0.9741	
		For climate and other			0.854	0.347	2.105	0.7320	
	Moderately religious	For climate reasons			0.458	0.102	2.048	0.3069	
		For other reasons			0.729	0.275	1.932	0.5249	
		For climate and other			NA	NA	NA	NA	
	Highly religious	For climate reasons			NA	NA	NA	NA	
		For other reasons			1.348	0.593	3.066	0.4760	
		For climate and other			NA	NA	NA	NA	
	Agnostic	For climate reasons	Adjusted (politics)	1043	0.578	0.227	1.470	0.2498	0.0111
		For other reasons			1.102	0.572	2.122	0.7715	
		For climate and other			0.959	0.383	2.400	0.9283	
	Moderately religious	For climate reasons			0.471	0.105	2.112	0.3253	
		For other reasons			0.738	0.274	1.987	0.5480	
		For climate and other			NA	NA	NA	NA	
	Highly religious	For climate reasons			NA	NA	NA	NA	
		For other reasons			1.416	0.614	3.263	0.4147	
		For climate and other			NA	NA	NA	NA	
Taken action to eat less or no meat and/or dairy									
Belief (ref = No)	Not sure	For climate reasons	Unadjusted	1039	0.966	0.654	1.427	0.8615	0.3574
		For other reasons			1.023	0.679	1.543	0.9117	
		For climate and other			1.059	0.726	1.545	0.7659	
	Yes	For climate reasons			0.837	0.526	1.333	0.4531	
		For other reasons			0.679	0.399	1.155	0.1533	
		For climate and other			0.574	0.343	0.961	0.0349	
	Not sure	For climate reasons	Adjusted	1039	0.964	0.637	1.459	0.8642	0.3766
		For other reasons			1.048	0.686	1.600	0.8287	
		For climate and other			0.988	0.663	1.472	0.9528	
	Yes	For climate reasons			0.796	0.487	1.301	0.3620	

Exposure	Exposure level	Outcome level (ref = No)	Model	*n*	RRR	LCI	UCI	*p*-value	*p* total
		For other reasons			0.696	0.405	1.199	0.1915	
		For climate and other			0.542	0.316	0.929	0.0260	
	Not sure	For climate reasons	Adjusted (politics)	1039	1.038	0.681	1.580	0.8629	0.4534
		For other reasons			1.083	0.707	1.658	0.7139	
		For climate and other			1.053	0.703	1.578	0.8032	
	Yes	For climate reasons			0.841	0.509	1.387	0.4971	
		For other reasons			0.721	0.418	1.246	0.2418	
		For climate and other			0.568	0.329	0.981	0.0424	
Identity (combined; ref = None)	Christian	For climate reasons	Unadjusted	1038	0.791	0.556	1.125	0.1917	0.0005
		For other reasons			0.729	0.497	1.068	0.1049	
		For climate and other			0.451	0.306	0.664	0.0001	
	Christian	For climate reasons	Adjusted	1038	0.790	0.544	1.148	0.2165	0.0008
		For other reasons			0.701	0.473	1.039	0.0767	
		For climate and other			0.446	0.298	0.670	0.0001	
	Christian	For climate reasons	Adjusted (politics)	1038	0.858	0.587	1.254	0.4293	0.0039
		For other reasons			0.731	0.492	1.086	0.1209	
		For climate and other			0.483	0.321	0.729	0.0005	
Identity (separate; ref = None)	C of E	For climate reasons	Unadjusted	1038	0.803	0.536	1.202	0.2866	0.0055
		For other reasons			0.840	0.550	1.284	0.4214	
		For climate and other			0.399	0.249	0.640	0.0001	
	Catholic	For climate reasons			0.505	0.187	1.362	0.1771	
		For other reasons			0.598	0.221	1.617	0.3112	
		For climate and other			0.432	0.160	1.161	0.0961	
	Other	For climate reasons			0.926	0.490	1.748	0.8125	
		For other reasons			0.439	0.180	1.069	0.0698	
		For climate and other			0.633	0.320	1.251	0.1881	
	C of E	For climate reasons	Adjusted	1038	0.818	0.531	1.259	0.3611	0.0194
		For other reasons			0.783	0.505	1.214	0.2746	
		For climate and other			0.403	0.246	0.661	0.0003	
	Catholic	For climate reasons			0.586	0.208	1.649	0.3116	
		For other reasons			0.658	0.237	1.829	0.4225	
		For climate and other			0.511	0.180	1.448	0.2064	
	Other	For climate reasons			0.814	0.415	1.597	0.5494	
		For other reasons			0.445	0.180	1.100	0.0796	

Exposure	Exposure level	Outcome level (ref = No)	Model	*n*	RRR	LCI	UCI	*p*-value	*p* total
		For climate and other			0.537	0.262	1.101	0.0896	
	C of E	For climate reasons	Adjusted (politics)	1038	0.895	0.577	1.388	0.6199	0.0646
		For other reasons			0.815	0.523	1.268	0.3639	
		For climate and other			0.440	0.267	0.726	0.0013	
	Catholic	For climate reasons			0.642	0.226	1.821	0.4045	
		For other reasons			0.698	0.250	1.950	0.4933	
		For climate and other			0.553	0.193	1.582	0.2692	
	Other	For climate reasons			0.862	0.434	1.713	0.6722	
		For other reasons			0.464	0.187	1.151	0.0976	
		For climate and other			0.568	0.275	1.173	0.1264	
Attendance (ref = Occasional/None)	Regular	For climate reasons	Unadjusted	1030	1.278	0.655	2.492	0.4723	0.4901
		For other reasons			0.746	0.318	1.747	0.4998	
		For climate and other			0.688	0.307	1.542	0.3642	
	Regular	For climate reasons	Adjusted	1030	1.191	0.580	2.446	0.6334	0.4555
		For other reasons			0.769	0.322	1.837	0.5542	
		For climate and other			0.597	0.255	1.398	0.2346	
	Regular	For climate reasons	Adjusted (politics)	1030	1.259	0.604	2.623	0.5391	0.4584
		For other reasons			0.779	0.324	1.871	0.5767	
		For climate and other			0.621	0.263	1.466	0.2769	
Latent class (ref = "Atheist")	Agnostic	For climate reasons	Unadjusted	992	0.761	0.488	1.186	0.2280	0.0860
		For other reasons			0.704	0.440	1.124	0.1415	
		For climate and other			0.921	0.610	1.390	0.6960	
	Moderately religious	For climate reasons			0.941	0.529	1.673	0.8358	
		For other reasons			0.268	0.104	0.693	0.0066	
		For climate and other			0.556	0.285	1.085	0.0853	
	Highly religious	For climate reasons			0.989	0.539	1.817	0.9722	
		For other reasons			0.631	0.305	1.307	0.2150	
		For climate and other			0.762	0.400	1.452	0.4089	
	Agnostic	For climate reasons	Adjusted	992	0.729	0.454	1.170	0.1908	0.0709
		For other reasons			0.700	0.432	1.133	0.1464	
		For climate and other			0.798	0.514	1.240	0.3153	
	Moderately religious	For climate reasons			0.956	0.514	1.777	0.8860	
		For other reasons			0.264	0.100	0.693	0.0069	
		For climate and other			0.480	0.238	0.969	0.0406	

**Exposure**	**Exposure level**	**Outcome level (ref = No)**	**Model**	***n***	**RRR**	**LCI**	**UCI**	***p*-value**	***p* total**
	Highly religious	For climate reasons			0.841	0.440	1.606	0.5990	
		For other reasons			0.658	0.311	1.392	0.2735	
		For climate and other			0.720	0.365	1.421	0.3431	
	Agnostic	For climate reasons	Adjusted (politics)	992	0.770	0.476	1.245	0.2862	0.1023
		For other reasons			0.712	0.438	1.156	0.1695	
		For climate and other			0.830	0.531	1.297	0.4130	
	Moderately religious	For climate reasons			0.990	0.528	1.857	0.9760	
		For other reasons			0.275	0.104	0.723	0.0089	
		For climate and other			0.494	0.242	1.009	0.0528	
	Highly religious	For climate reasons			0.839	0.433	1.628	0.6041	
		For other reasons			0.644	0.302	1.371	0.2535	
		For climate and other			0.713	0.358	1.423	0.3373	

Figure S1: Scree plot for the parallel analysis for the nine political ideology variables in G1 offspring used to inform the creation of the 'political ideology' principal components. As the variables were binary, tetrachoric correlations were used. Based on inspection of the scree plot, and the interpretability of the resulting components, we decided that a two-factor solution was the optimal fit to the data.

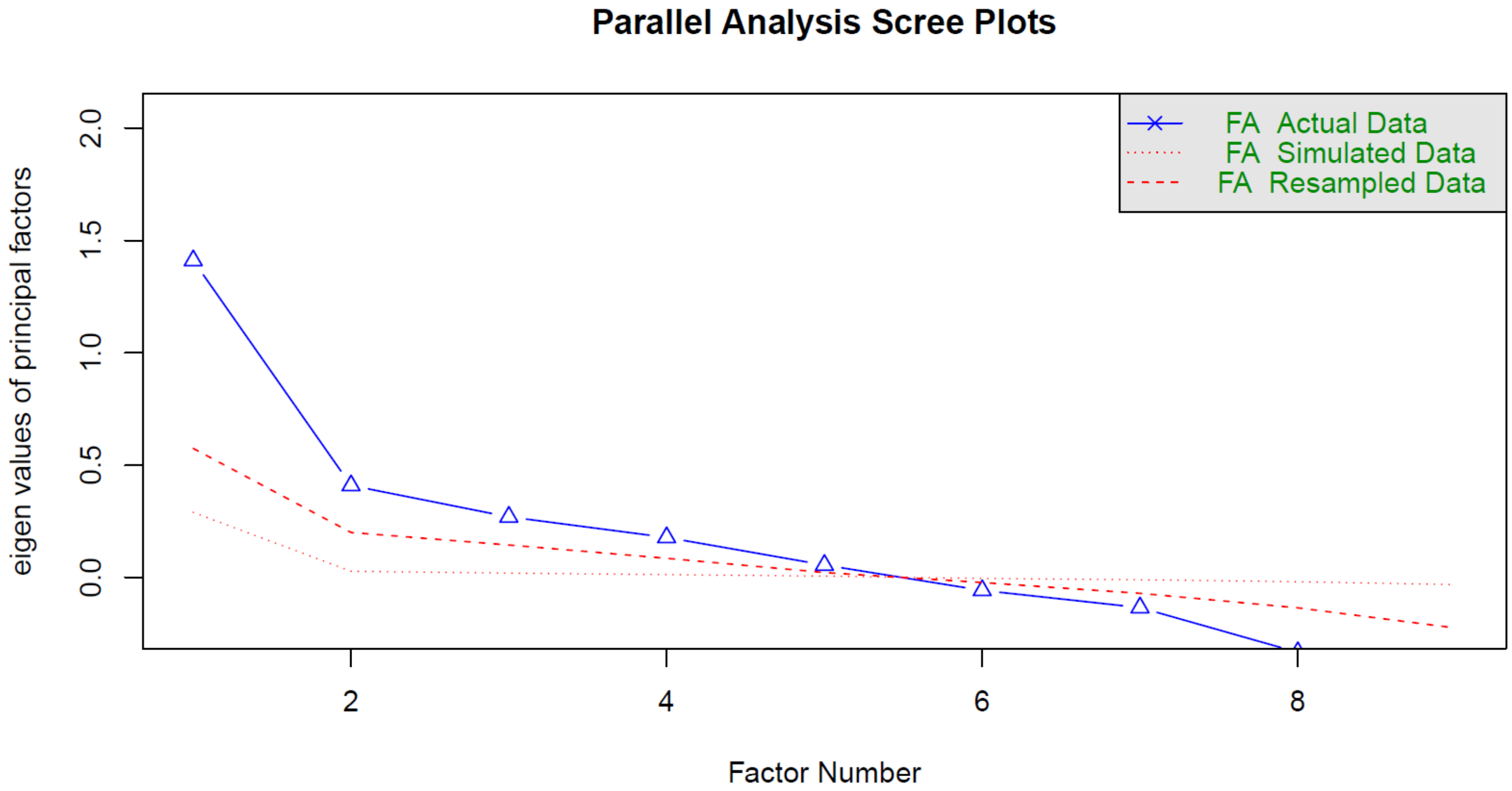

Figure S2: Histogram of total number of pro-environmental actions performed for climate change reasons in G0 mothers (max = 16).

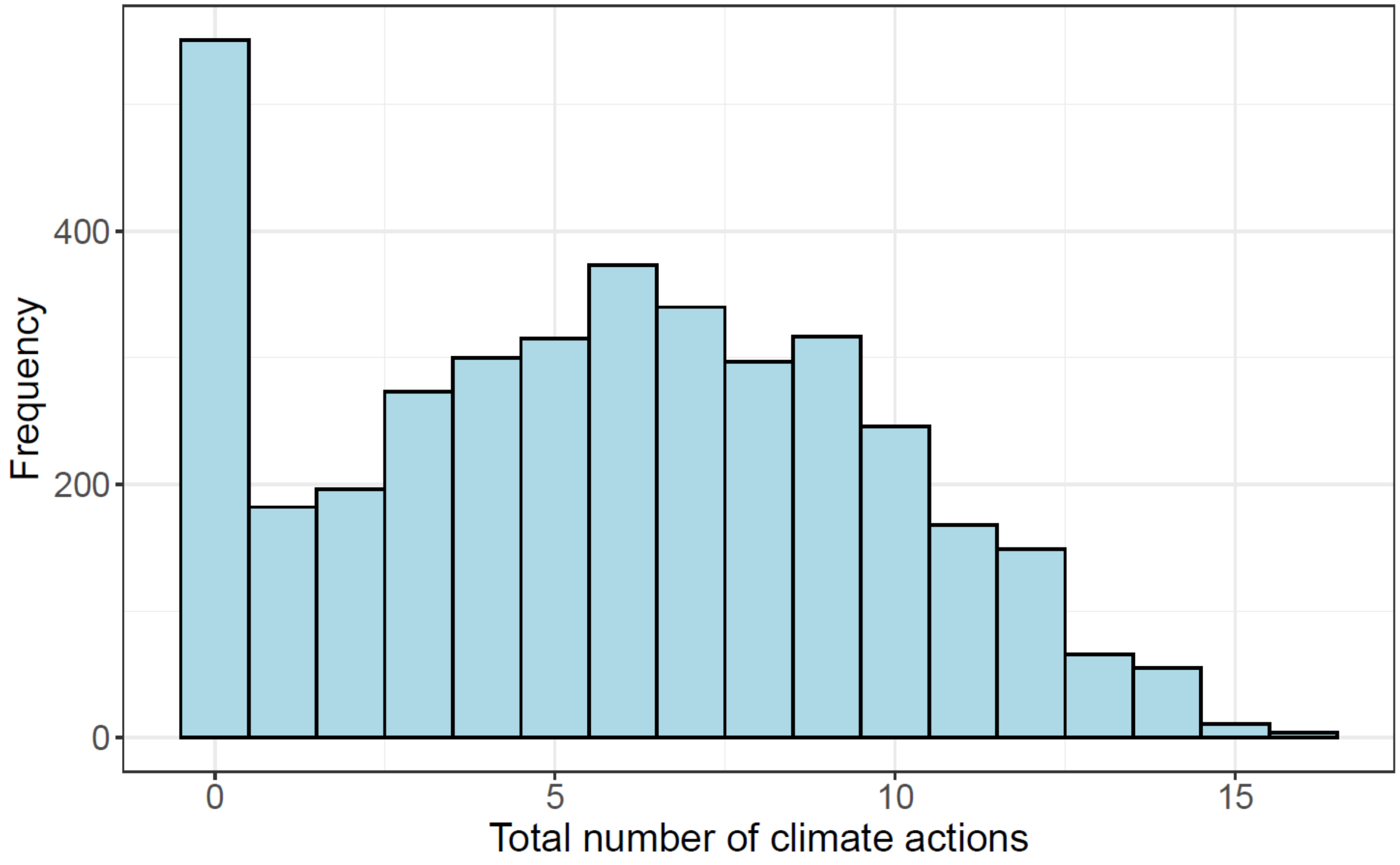

Figure S3: Predicted probabilities of the mothers ordinal regression models with 'belief that the climate is changing' as the outcome for four religious exposures (belief, identity, attendance and latent classes).

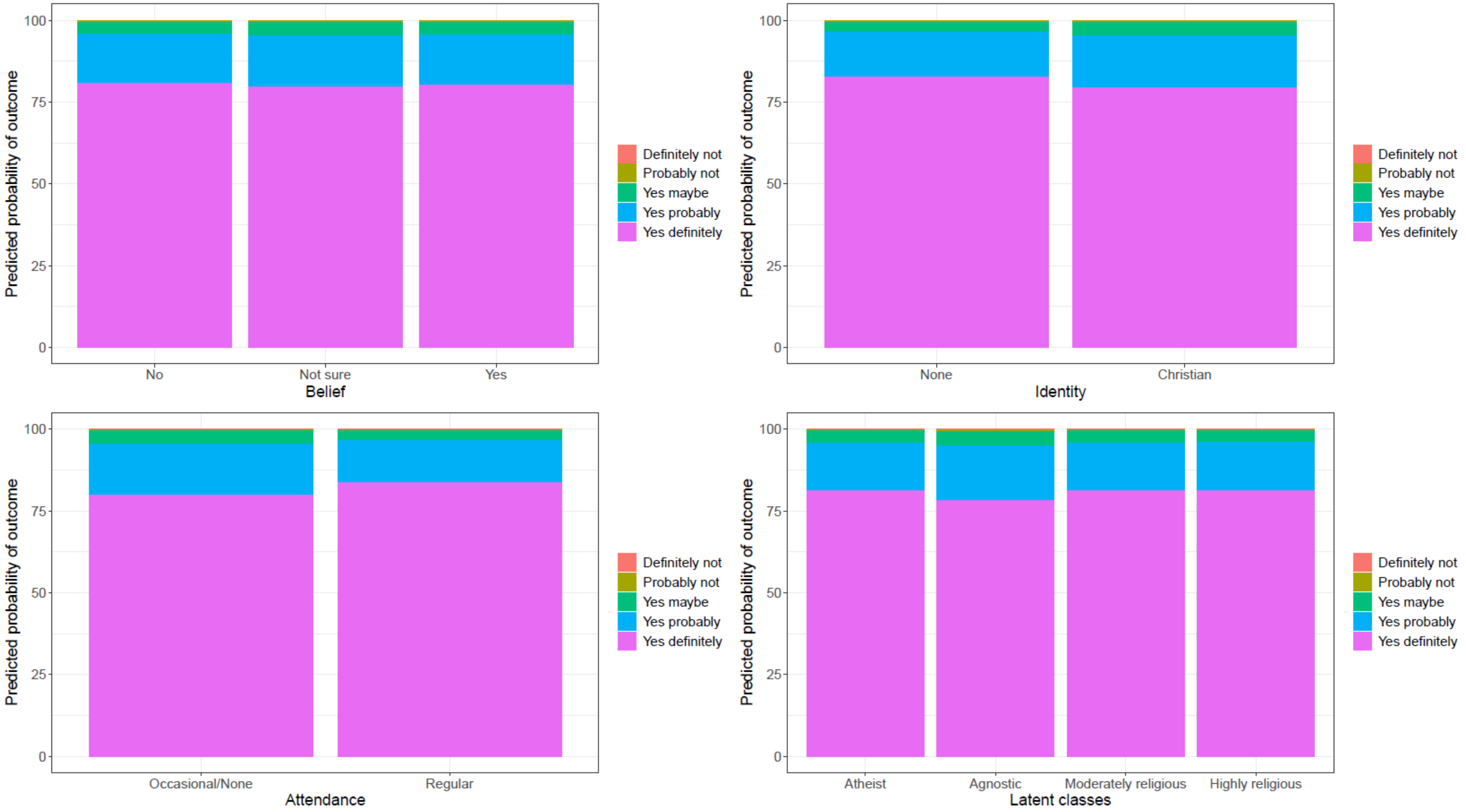

Figure S4: Predicted probabilities of the mothers ordinal regression models with ‘belief that the climate is changing’ as the outcome and the religious identity (with the Christian denominations separated) as the exposure.

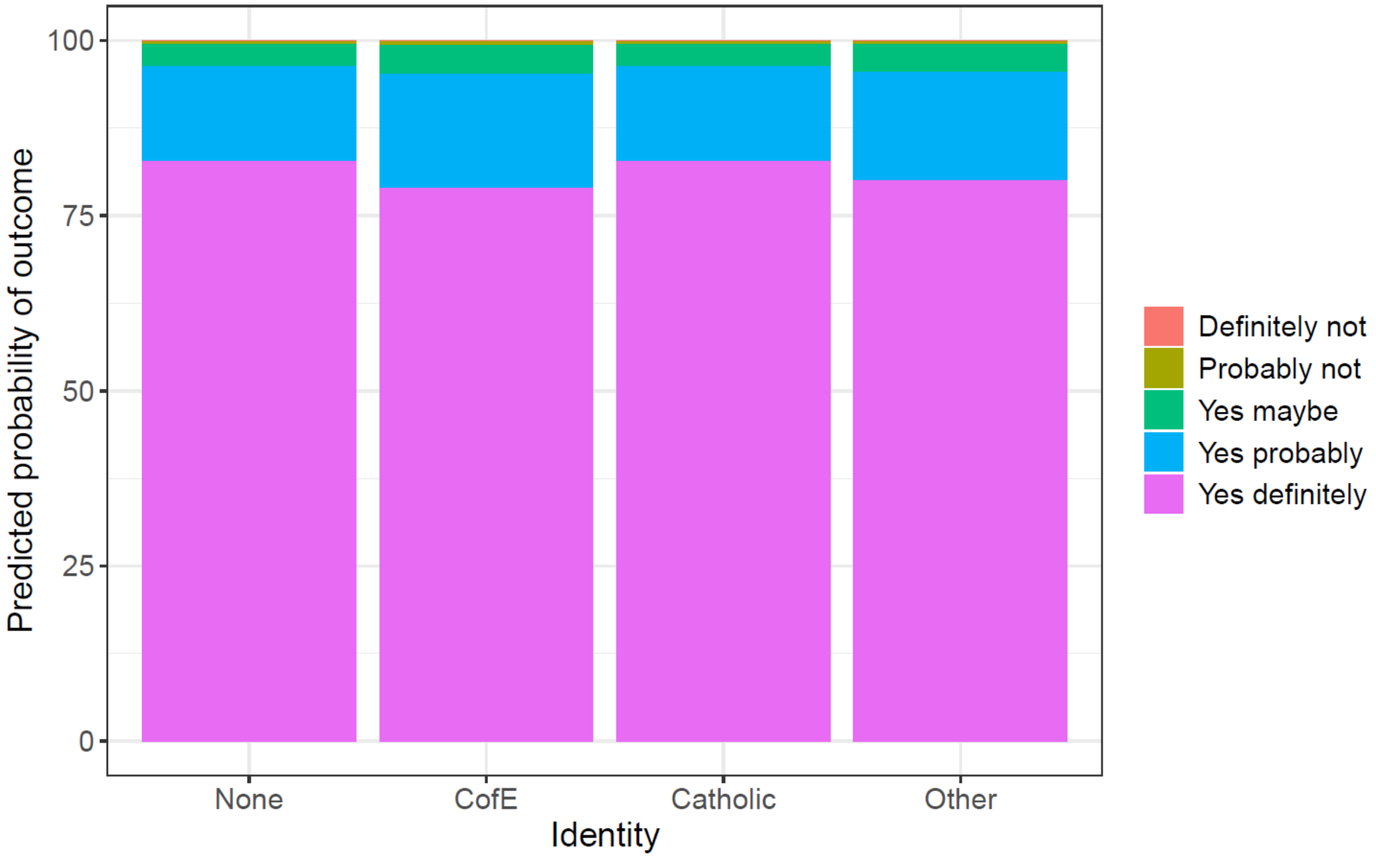

Figure S5: Results of the mothers ordinal regression models with 'concerned about the impact of climate change' as the outcome for four religious exposures (belief [*n* = 2,560], identity [*n* = 2,534], attendance [*n* = 2,534], and latent classes [*n* = 2,566]; models are separated by dashed horizontal lines). Odds ratios above 1 indicate an increased concern regarding climate change. See table S10 for full results.

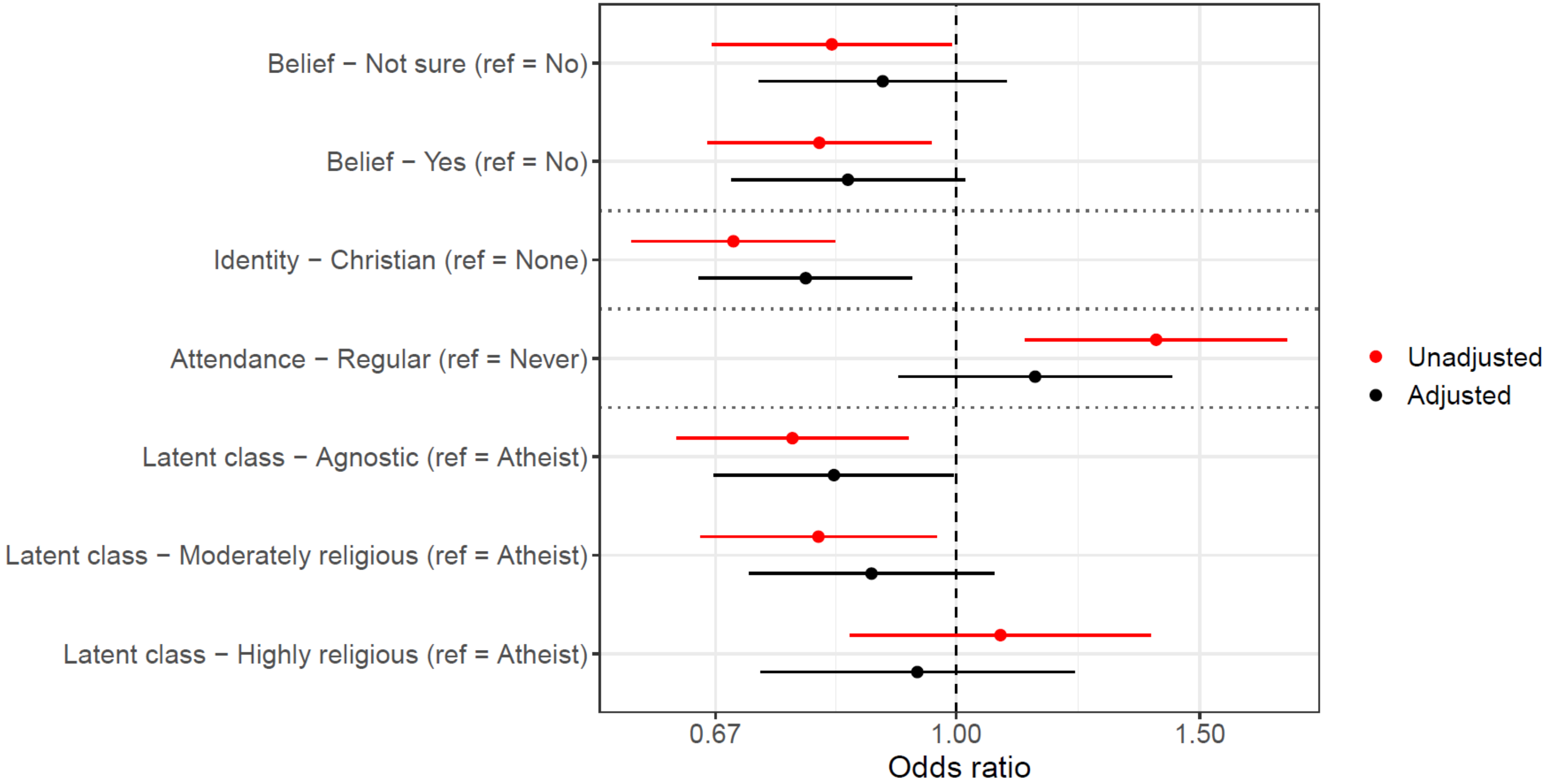

Figure S6: Predicted probabilities of the mothers ordinal regression models with 'concerned about the impact of climate change' as the outcome for four religious exposures (belief, identity, attendance and latent classes).

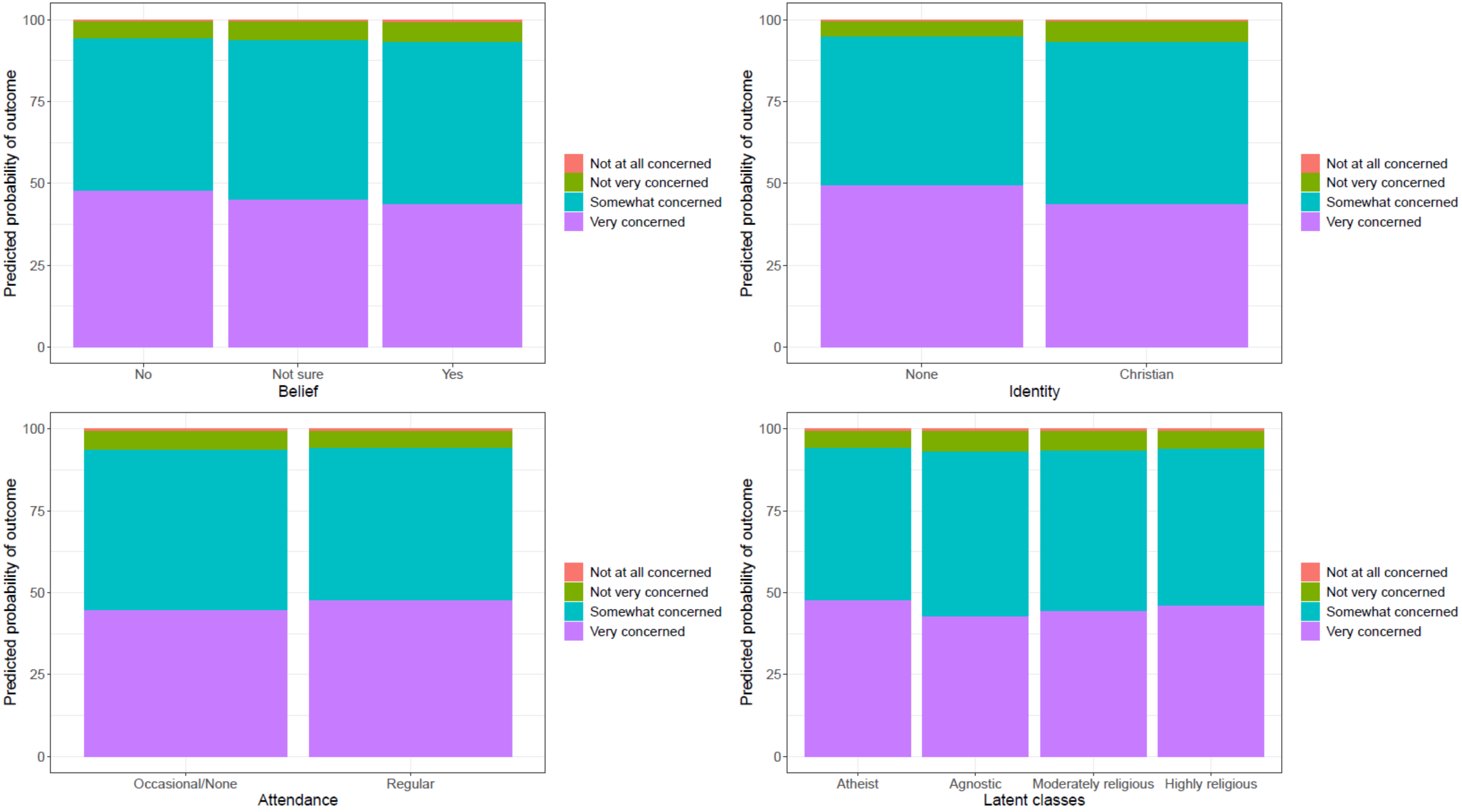

Figure S7: Predicted probabilities of the mothers ordinal regression models with 'concerned about the impact of climate change' as the outcome and the religious identity (with the Christian denominations separated) as the exposure.

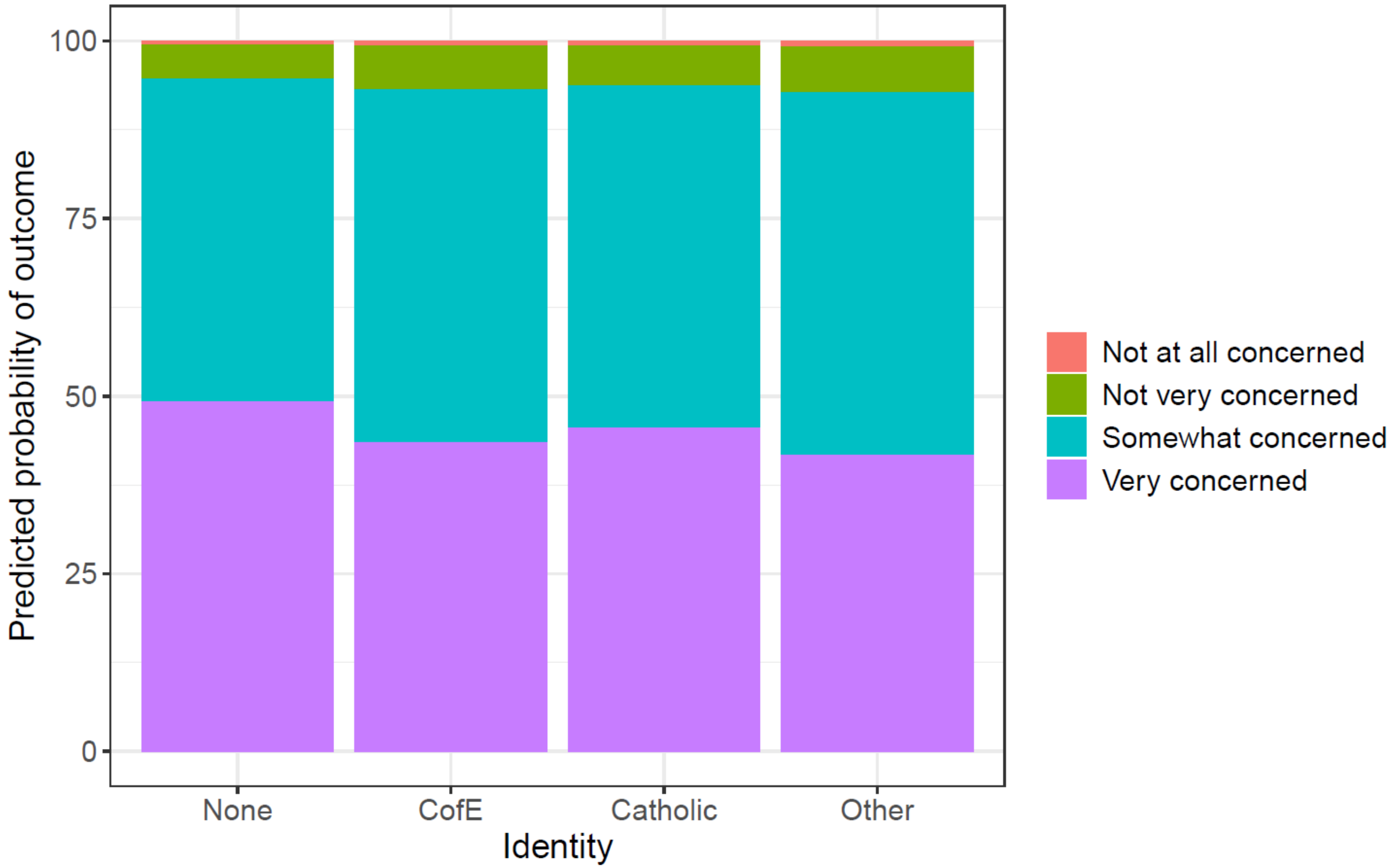

Figure S8: Predicted probabilities of the mothers ordinal regression models with 'believes that humans are to blame for climate change' as the outcome for four religious exposures (belief, identity, attendance and latent classes).

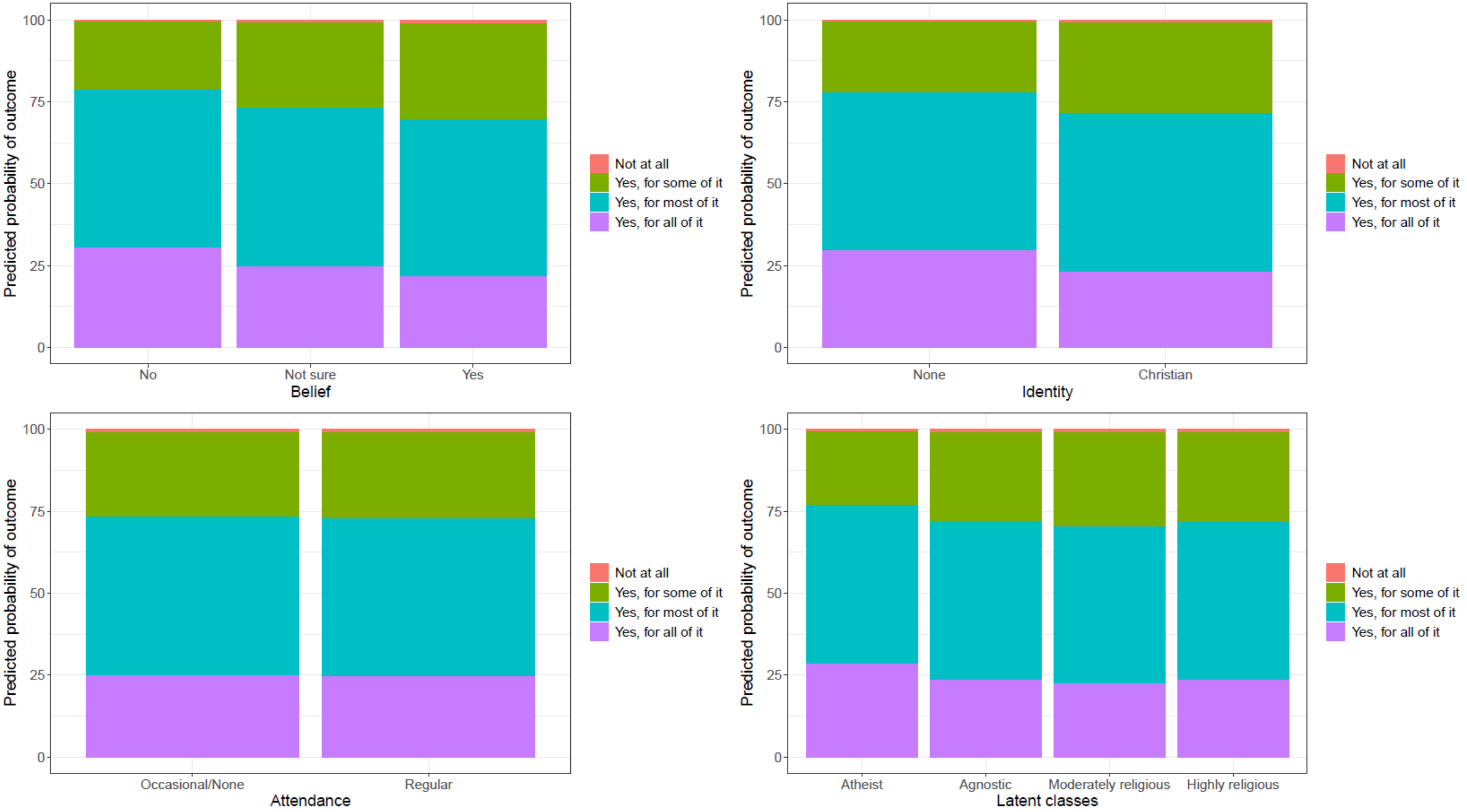

Figure S9: Predicted probabilities of the mothers ordinal regression models with 'believes that humans are to blame for climate change' as the outcome and the religious identity (with the Christian denominations separated) as the exposure.

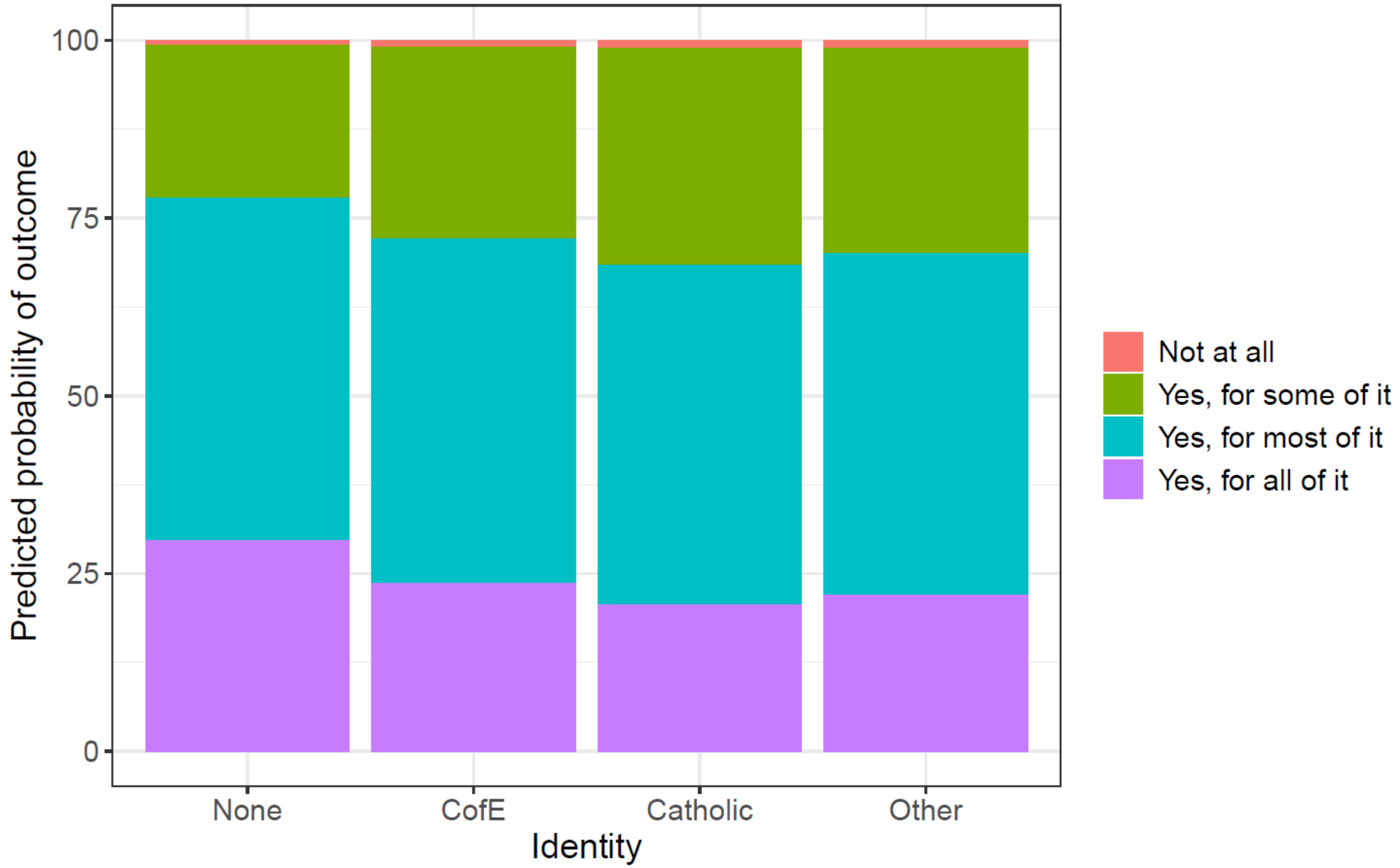

Figure S10: Results of the mothers multinomial regression models with 'thinks that personal actions will make a difference to long-term climate change' as the outcome for four religious exposures (belief [*n* = 2,560], identity [*n* = 2,534], attendance [*n* = 2,534], and latent classes [*n* = 2,566]; models are separated by dashed horizontal lines). See table S12 for full results.

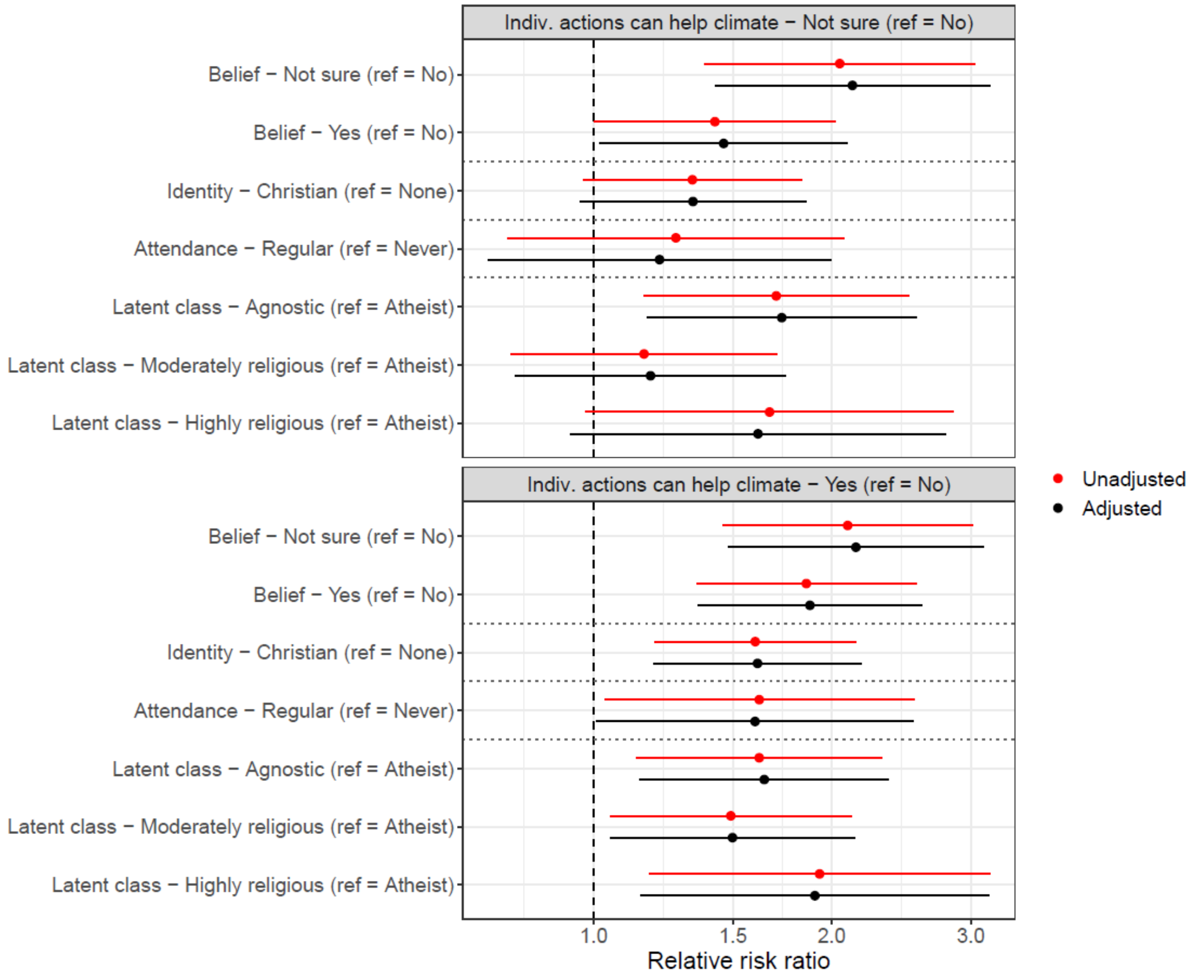

Figure S11: Predicted probabilities of the mothers multinomial regression models with 'thinks that personal actions will make a difference to long-term climate change' as the outcome for four religious exposures (belief, identity, attendance and latent classes).

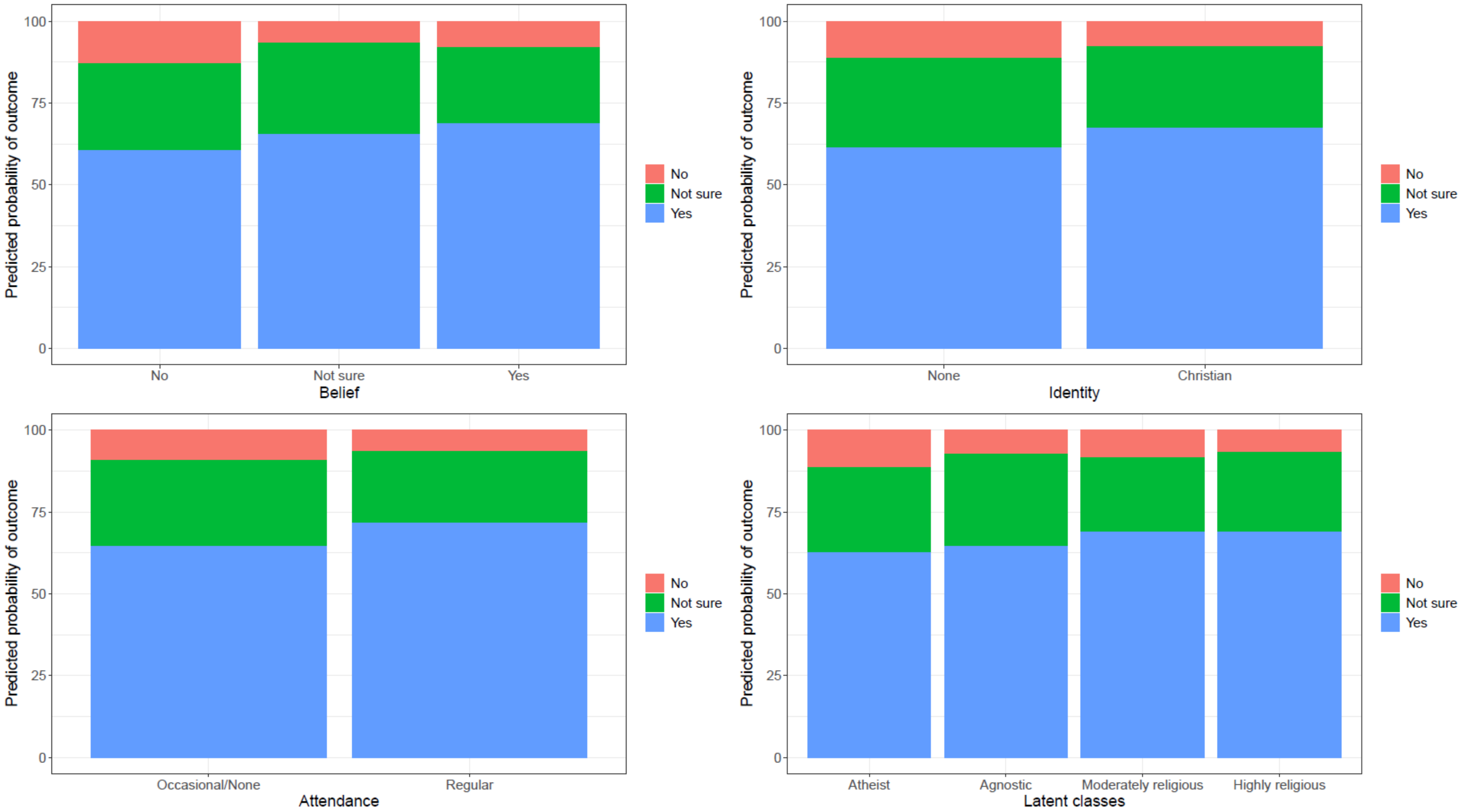

Figure S12: Predicted probabilities of the mothers multinomial regression models with 'thinks that personal actions will make a difference to long-term climate change' as the outcome and the religious identity (with the Christian denominations separated) as the exposure.

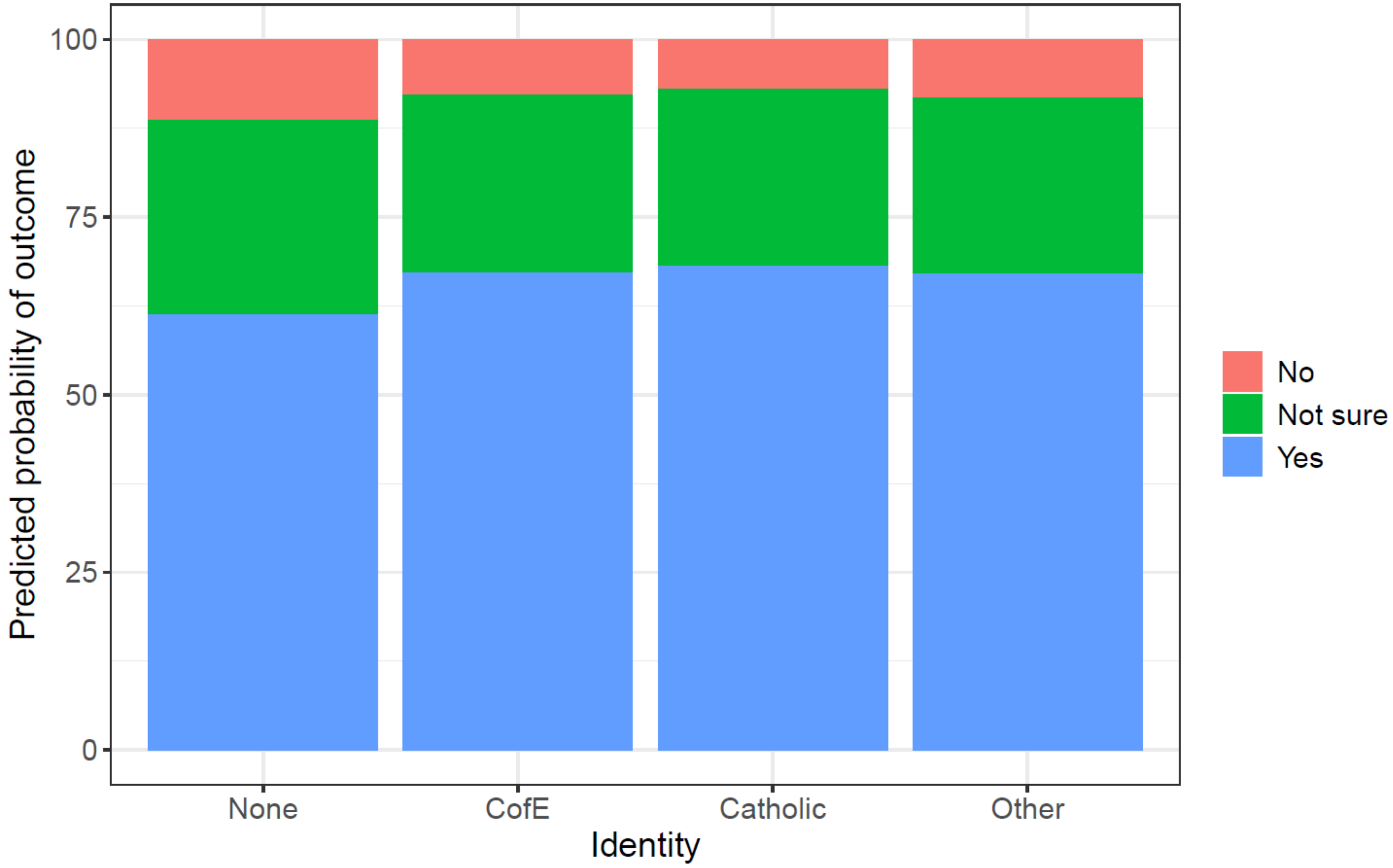

Figure S13: Predicted total number of actions performed due to climate change for four religious exposures (belief, identity, attendance and latent classes) based on the mothers linear regression models.

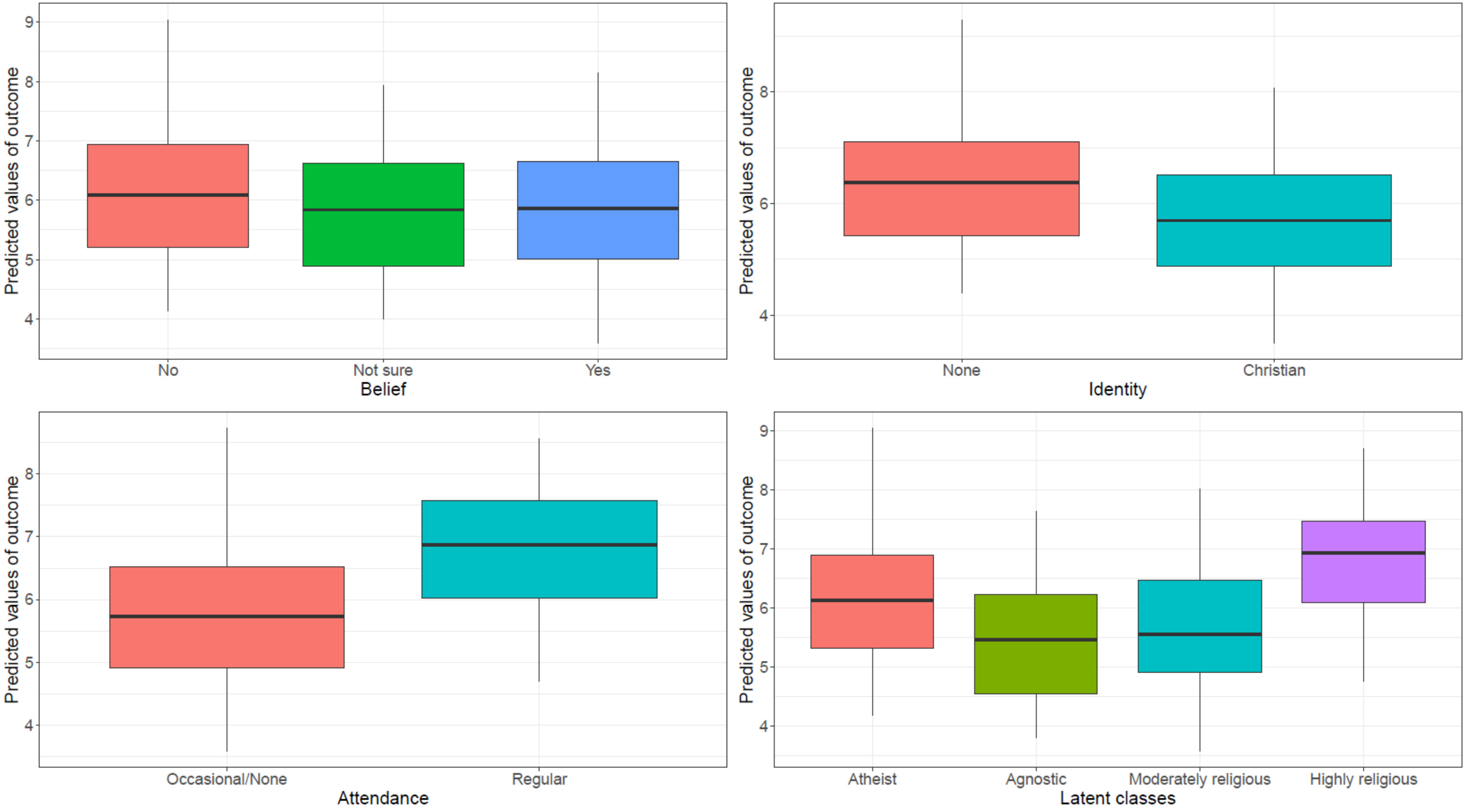

Figure S14: Predicted total number of actions performed due to climate change for the religious identity (with the Christian denominations separated) as the exposure based on the mothers linear regression models.

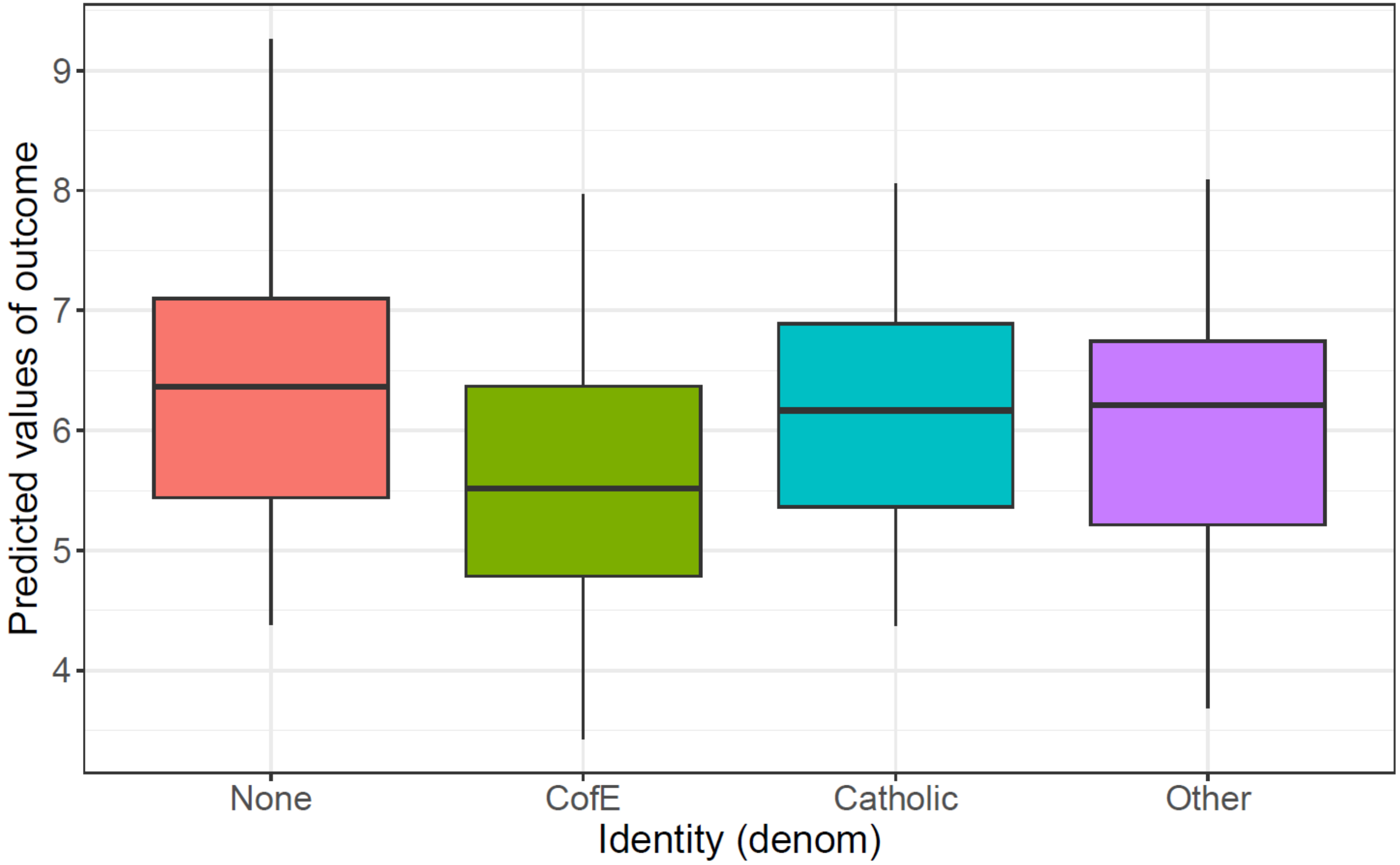

Figure S15: Results of the mothers Poisson regression models with 'total number of actions performed due to climate change' as the outcome for four religious exposures (belief [*n* = 2,218], identity [*n* = 2,195], attendance [*n* = 2,197], and latent classes [*n* = 2,224]; models are separated by dashed horizontal lines). Incidence rate ratios above 1 indicate an increased number of pro-environmental actions performed. See table S14 for full results.

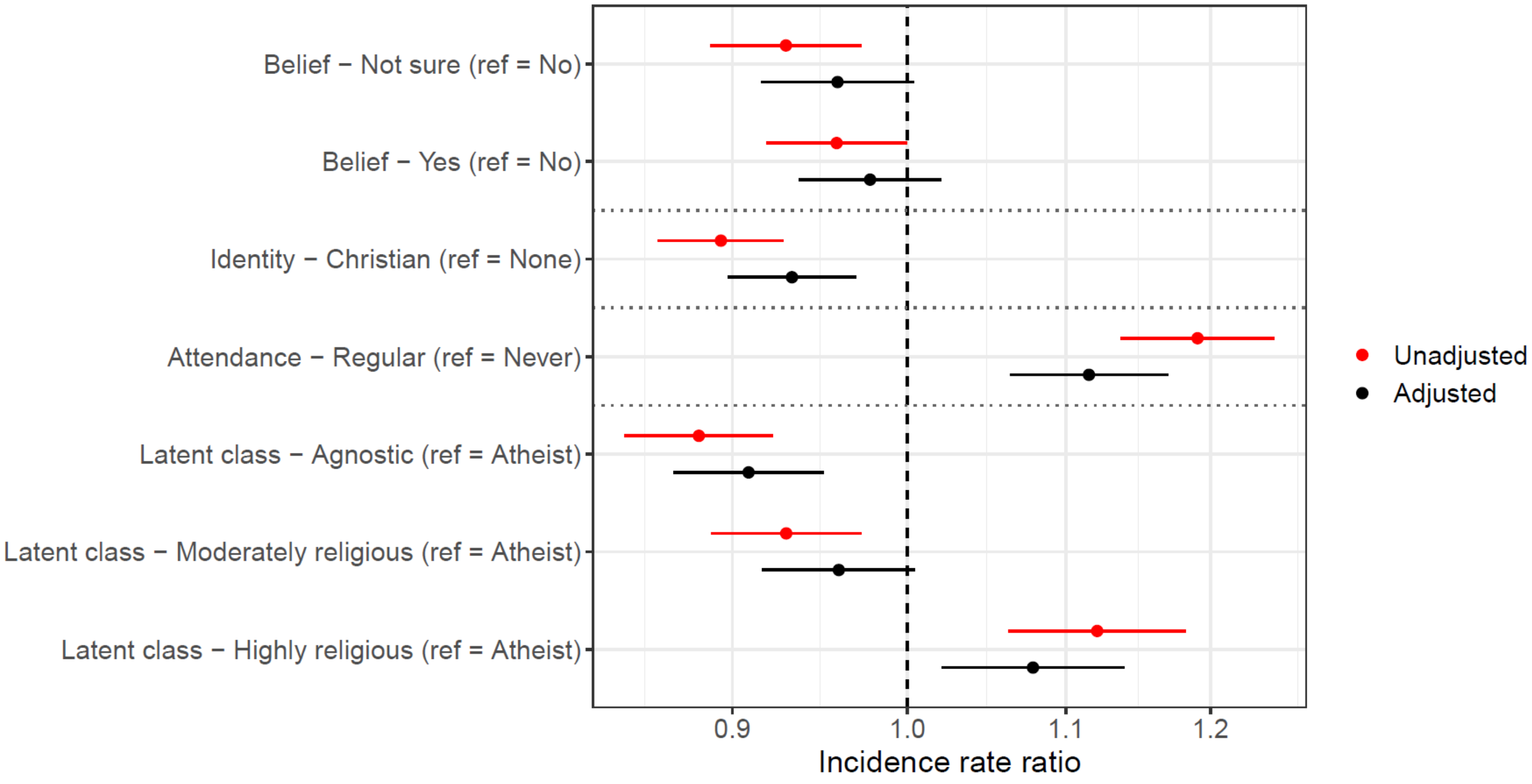

Figure S16: Predicted total number of actions performed due to climate change for four religious exposures (belief, identity, attendance and latent classes) based on the mothers Poisson regression models.

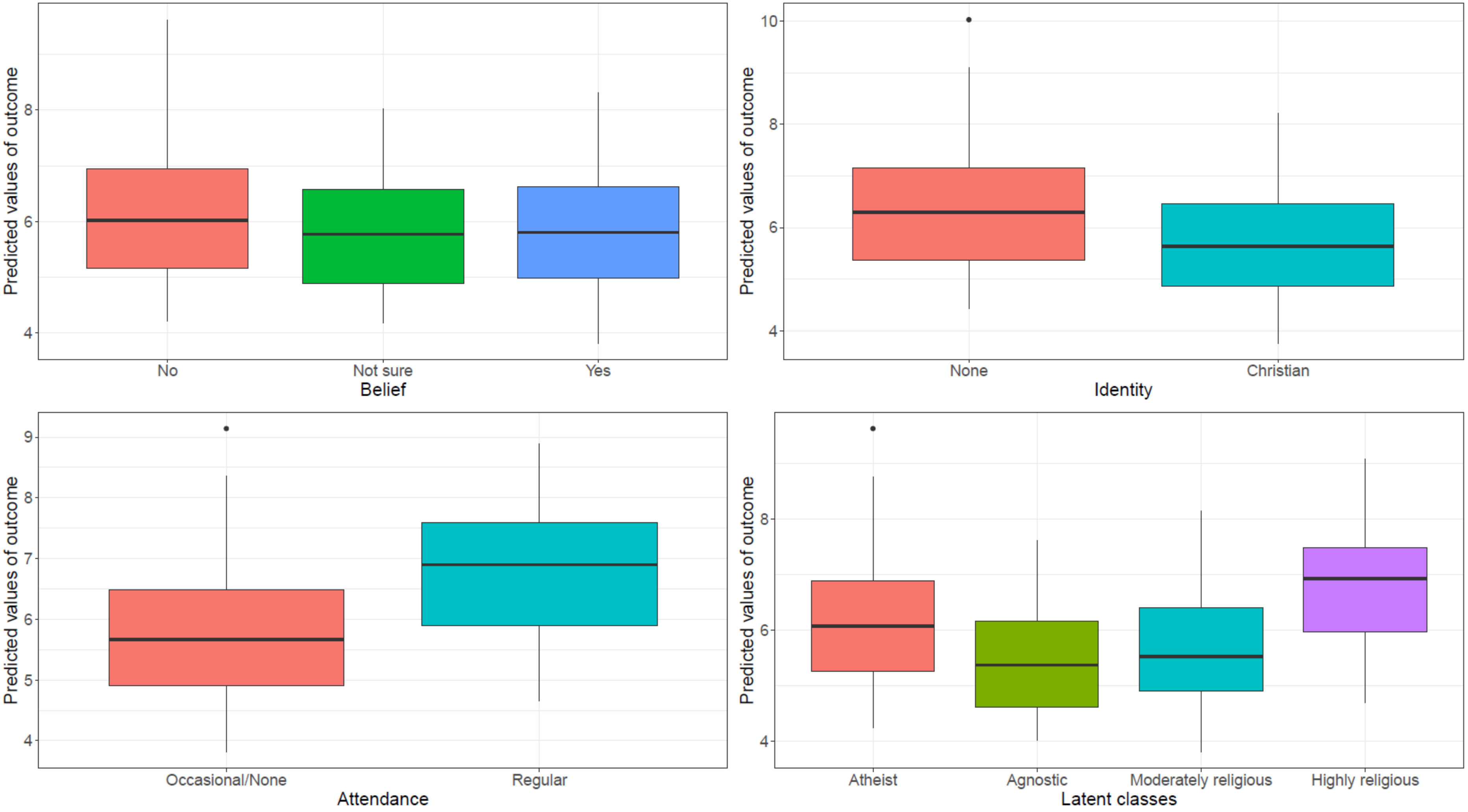

Figure S17: Predicted total number of actions performed due to climate change for the religious identity (with the Christian denominations separated) as the exposure based on the mothers Poisson regression models.

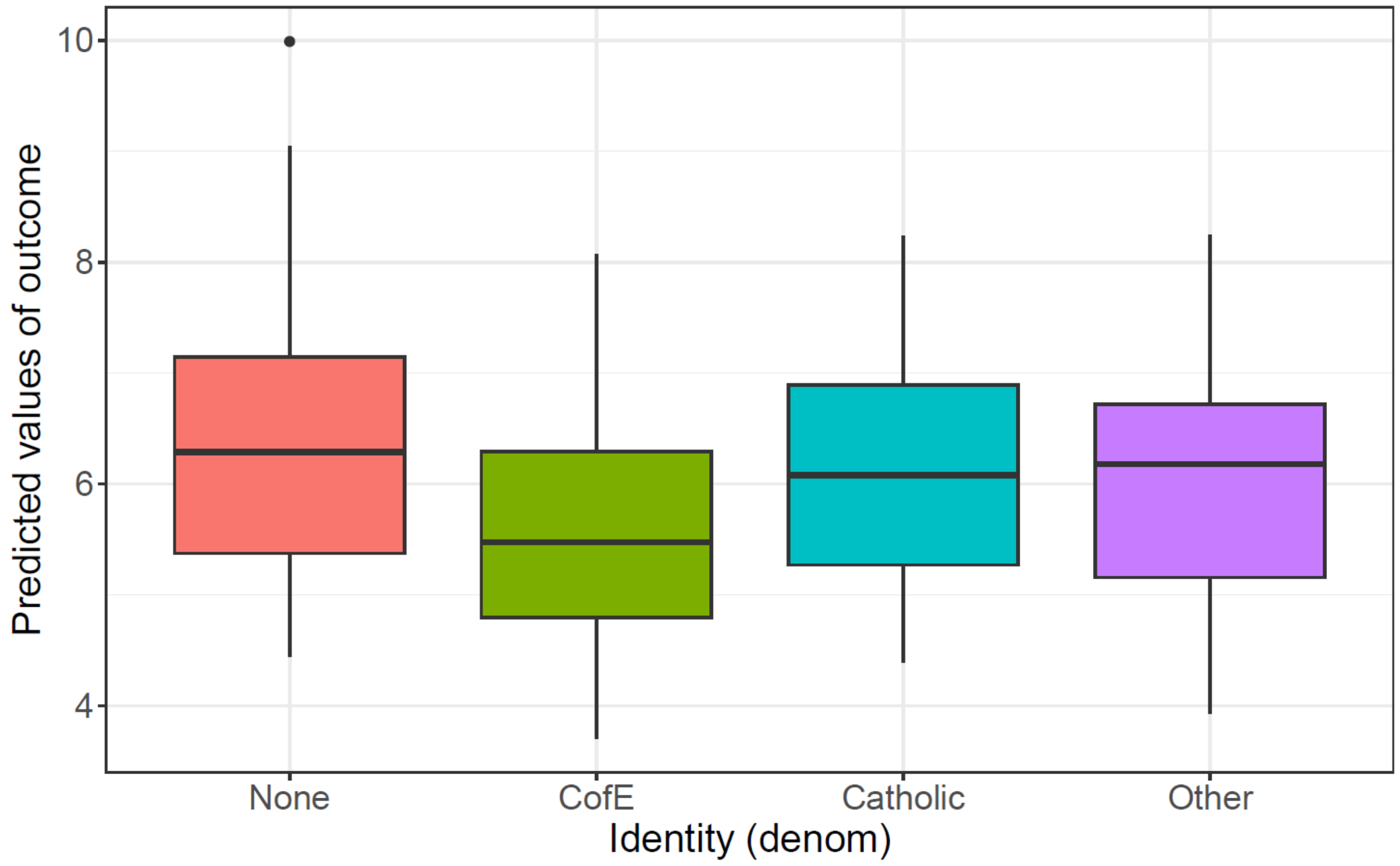

Figure S18: Results of the mothers zero-inflated Poisson regression models with 'total number of actions performed due to climate change' as the outcome for four religious exposures (belief [*n* = 2,218], identity [*n* = 2,195], attendance [*n* = 2,197], and latent classes [*n* = 2,224]; models are separated by dashed horizontal lines). Incidence rate ratios above 1 indicate an increased number of pro-environmental actions performed, while odds ratios above 1 indicate an excess of zeros. See table S15 for full results.

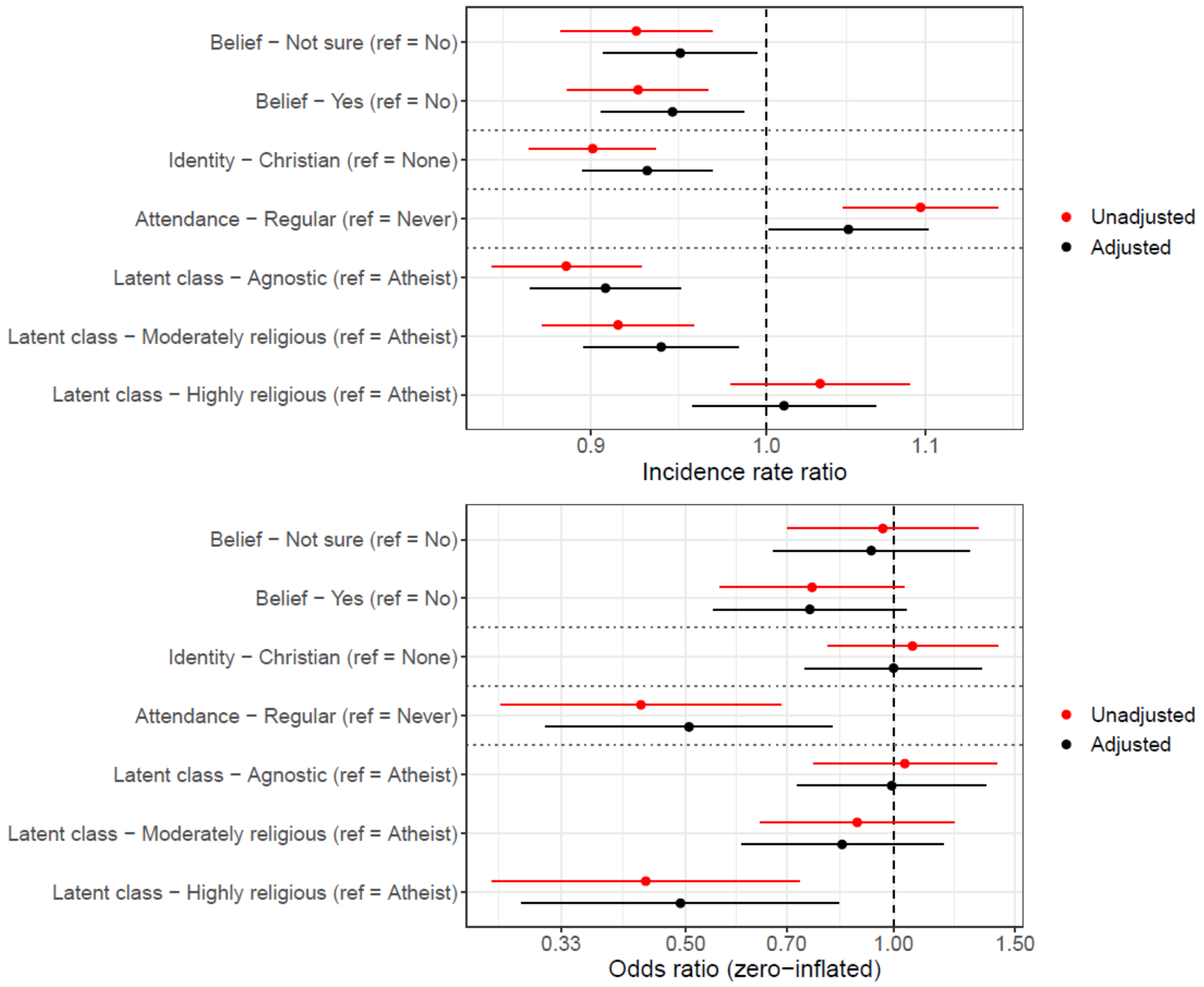

Figure S19: Predicted total number of actions performed due to climate change for four religious exposures (belief, identity, attendance and latent classes) based on the mothers zero-inflated Poisson regression models.

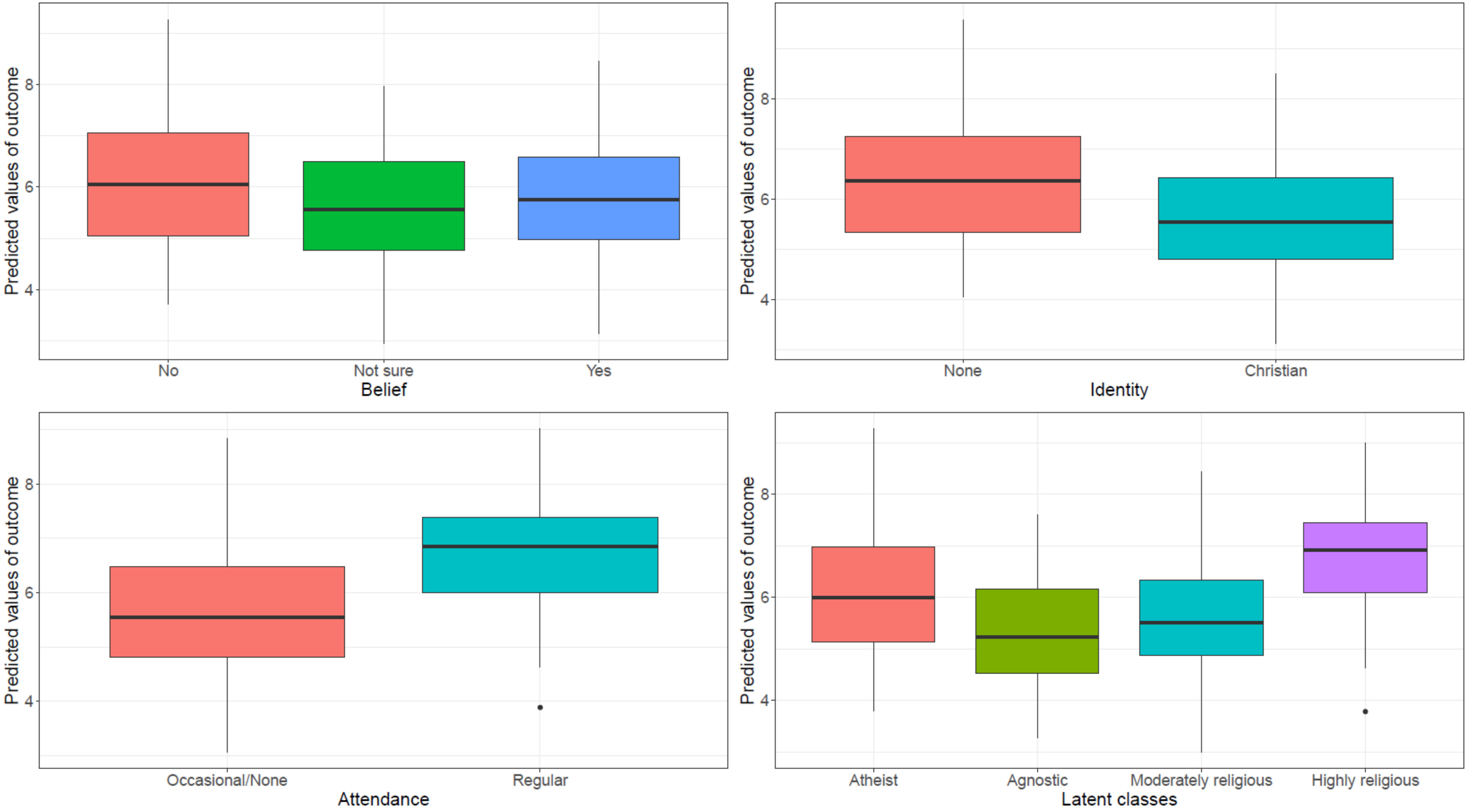

Figure S20: Predicted total number of actions performed due to climate change for the religious identity (with the Christian denominations separated) as the exposure based on the mothers zero-inflated Poisson regression models.

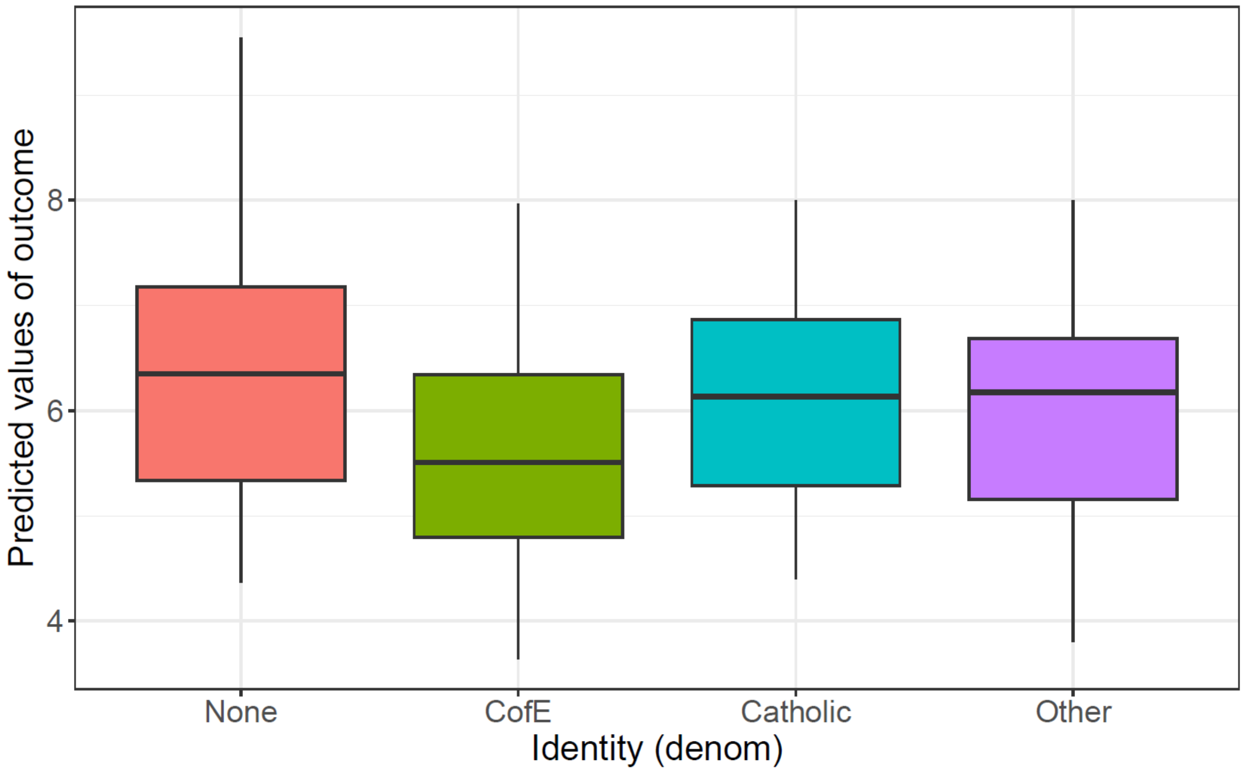

Figure S21: Results of the mothers linear regression models with 'total number of actions performed due to climate change (excluding ones which may be prohibitively costly)' as the outcome for four religious exposures (belief [*n* = 2,268], identity [*n* = 2,244], attendance [*n* = 2,246], and latent classes [*n* = 2,274]; models are separated by dashed horizontal lines). Values above 0 indicate an increased number of pro-environmental actions performed. See table S16 for full results.

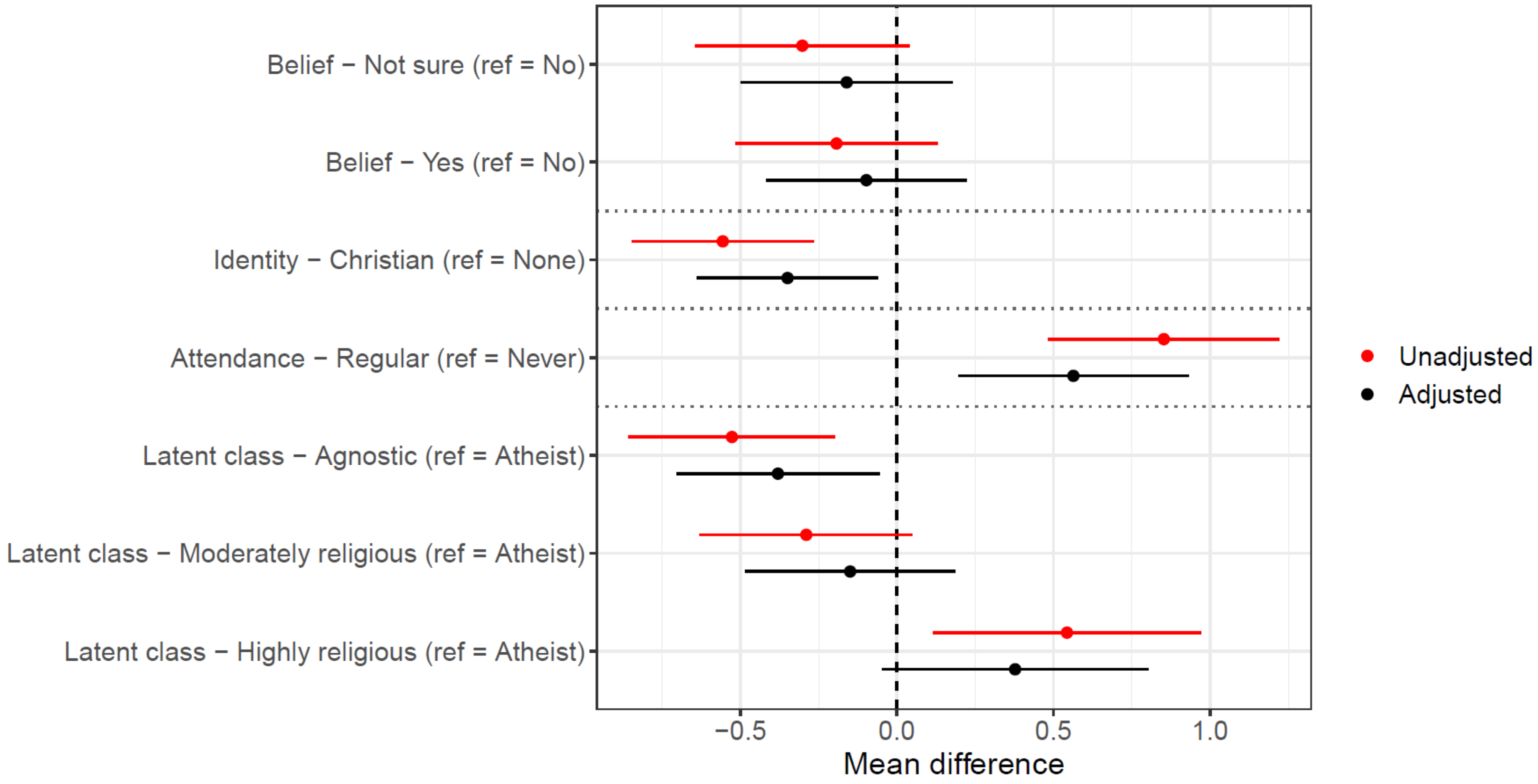

Figure S22: Predicted total number of actions (excluding ones which may be prohibitively costly) performed due to climate change for four religious exposures (belief, identity, attendance and latent classes) based on the mothers linear regression models.

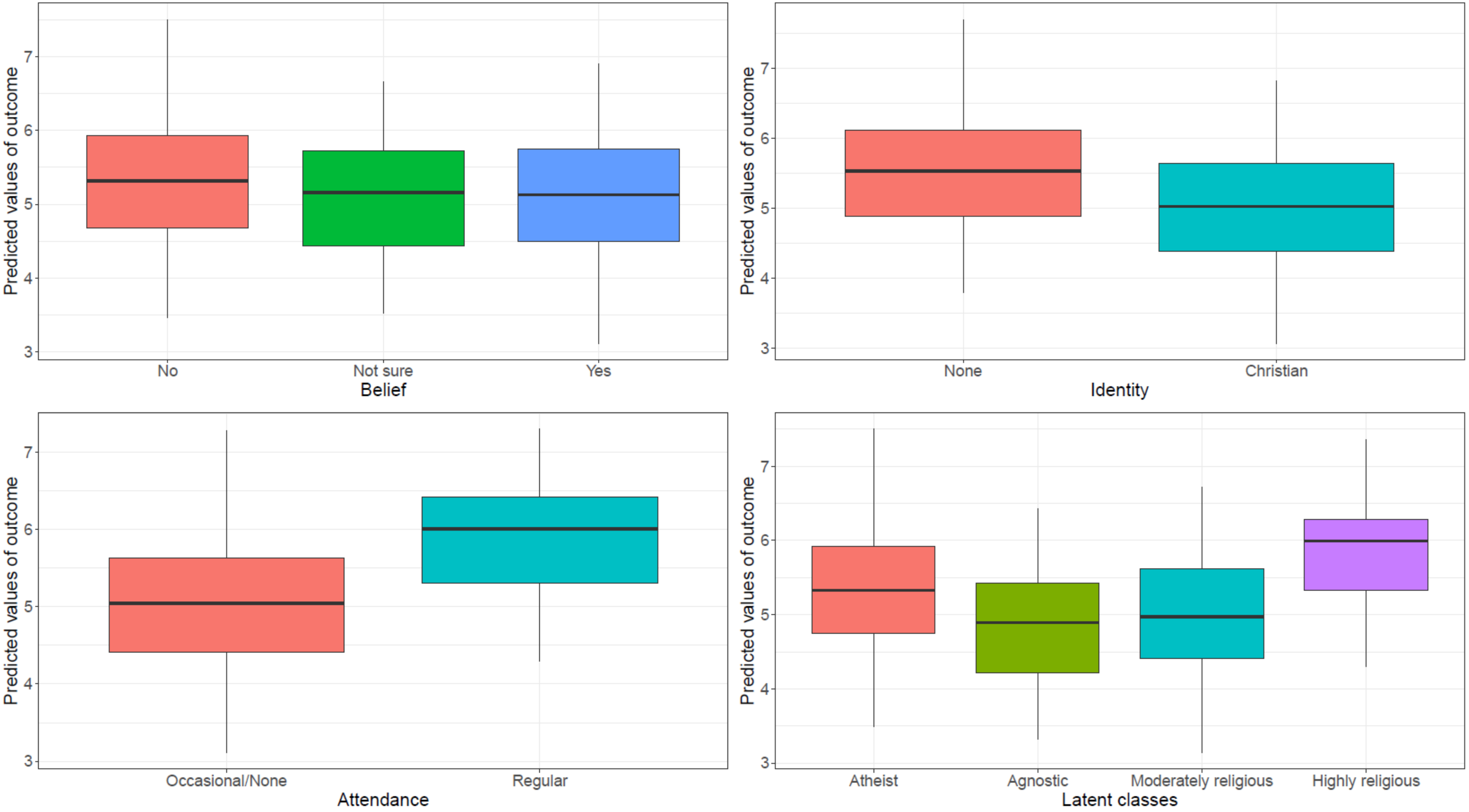

Figure S23: Predicted total number of actions (excluding ones which may be prohibitively costly) performed due to climate change for the religious identity (with the Christian denominations separated) as the exposure based on the mothers linear regression models.

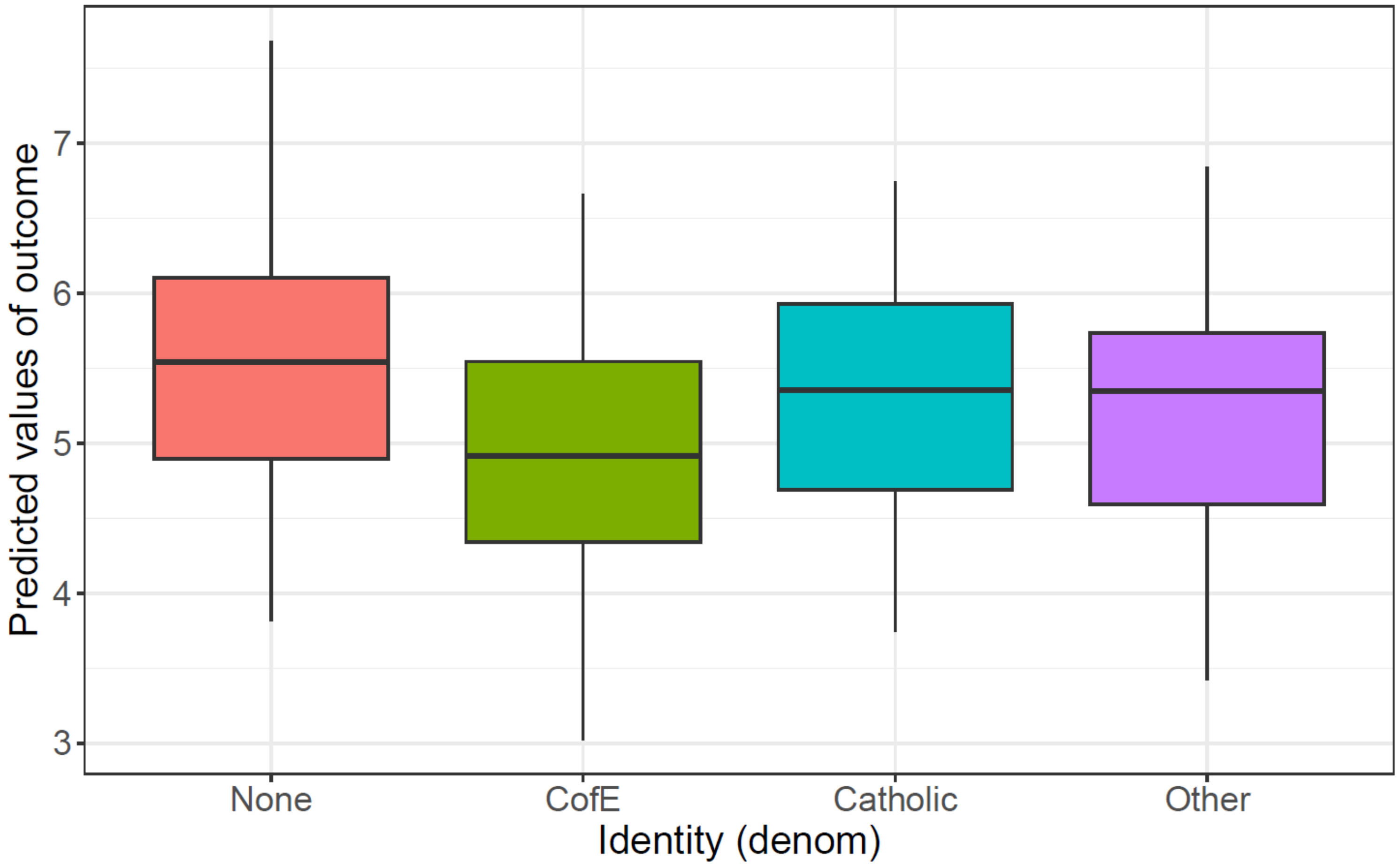

Figure S24: Results of the mothers Poisson regression models with ‘total number of actions performed due to climate change (excluding ones which may be prohibitively costly)’ as the outcome for four religious exposures (belief [*n* = 2,268], identity [*n* = 2,244], attendance [*n* = 2,246], and latent classes [*n* = 2,274]; models are separated by dashed horizontal lines). Incidence rate ratios above 1 indicate an increased number of pro-environmental actions performed. See table S17 for full results.

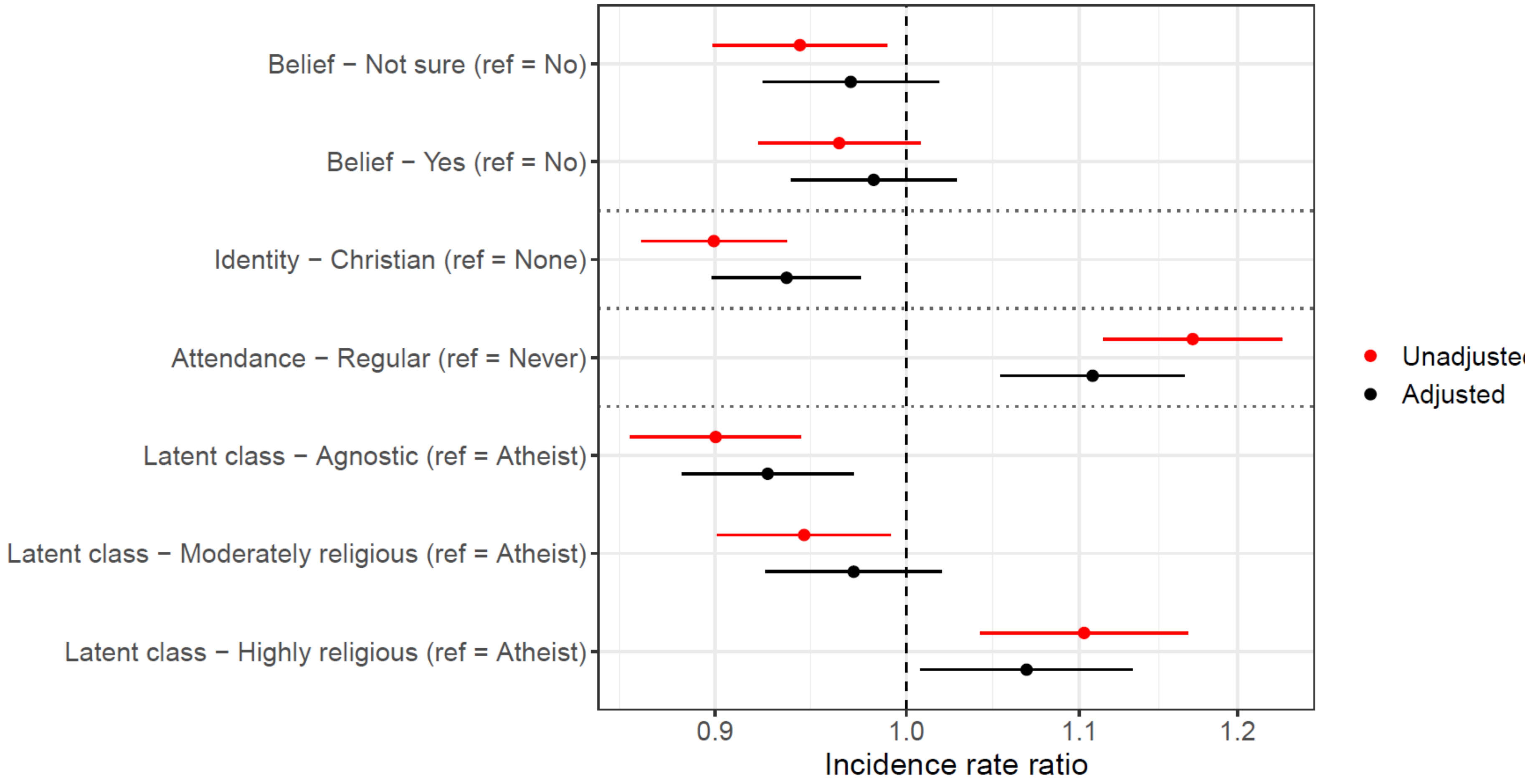

Figure S25: Predicted total number of actions (excluding ones which may be prohibitively costly) performed due to climate change for four religious exposures (belief, identity, attendance and latent classes) based on the mothers Poisson regression models.

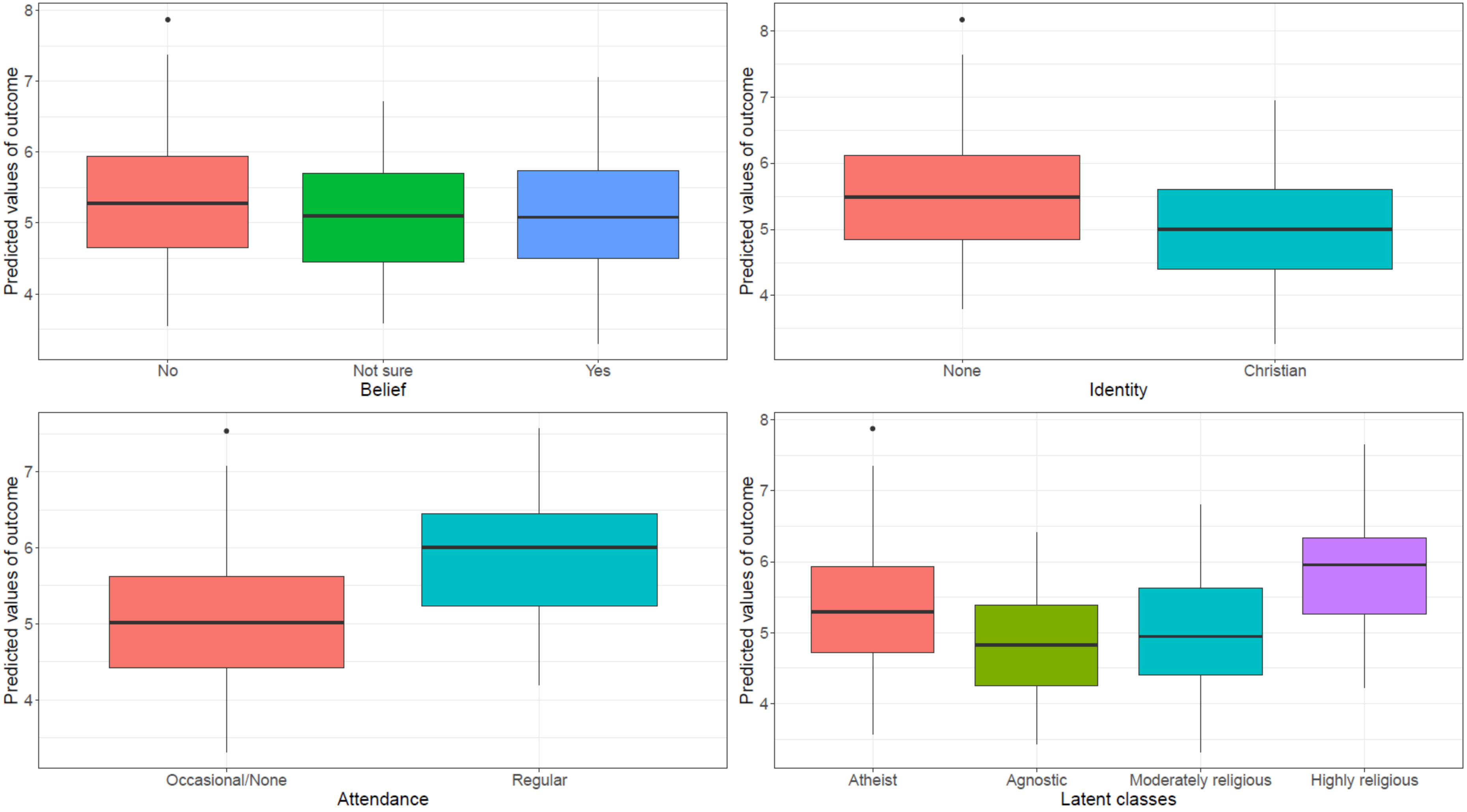

Figure S26: Predicted total number of actions (excluding ones which may be prohibitively costly) performed due to climate change for the religious identity (with the Christian denominations separated) as the exposure based on the mothers Poisson regression models.

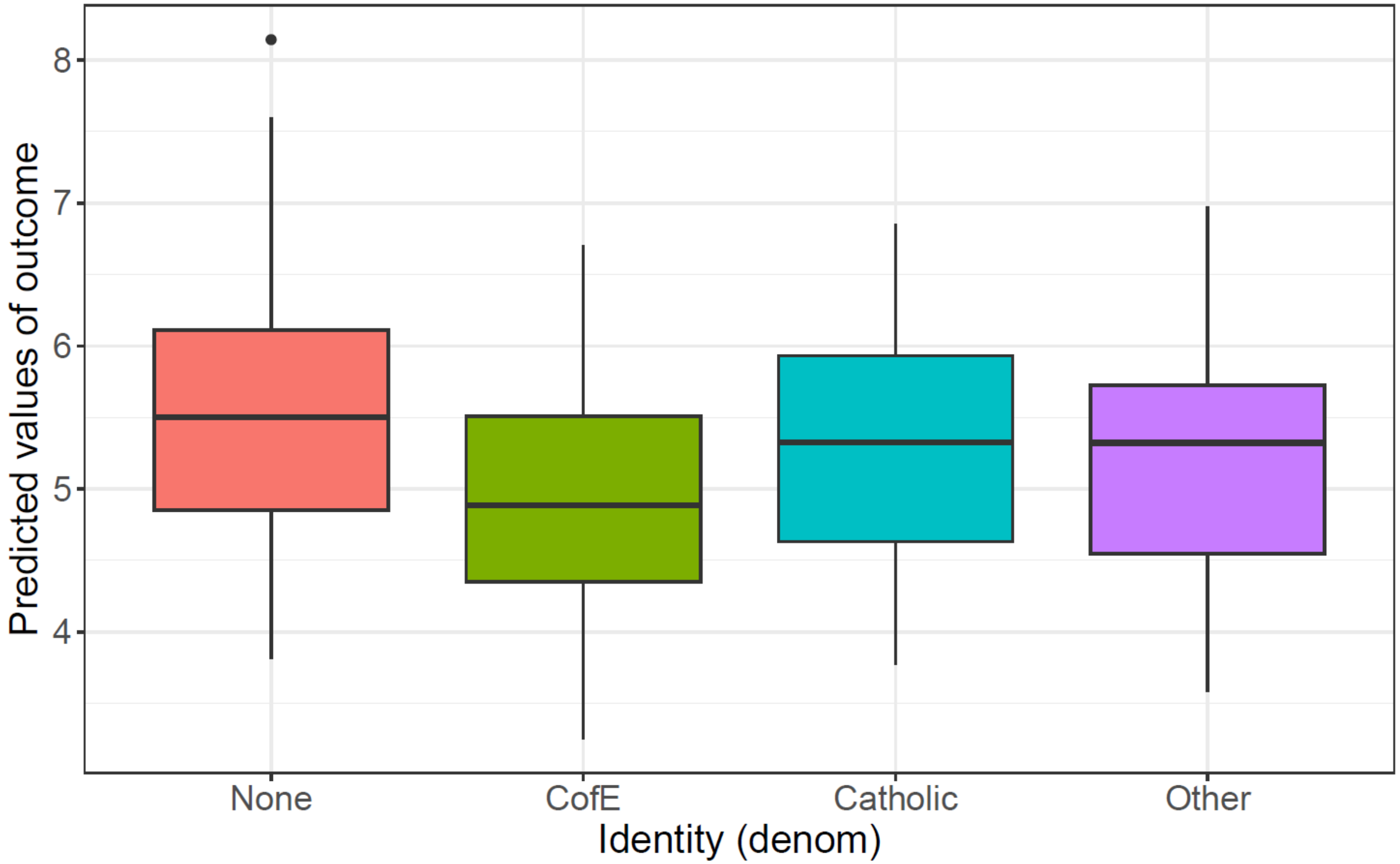

Figure S27: Results of the mothers zero-inflated Poisson regression models with 'total number of actions performed due to climate change (excluding ones which may be prohibitively costly)' as the outcome for four religious exposures (belief [*n* = 2,268], identity [*n* = 2,244], attendance [*n* = 2,246], and latent classes [*n* = 2,274]; models are separated by dashed horizontal lines). Incidence rate ratios above 1 indicate an increased number of pro-environmental actions performed, while odds ratios above 1 indicate an excess of zeros. See table S18 for full results.

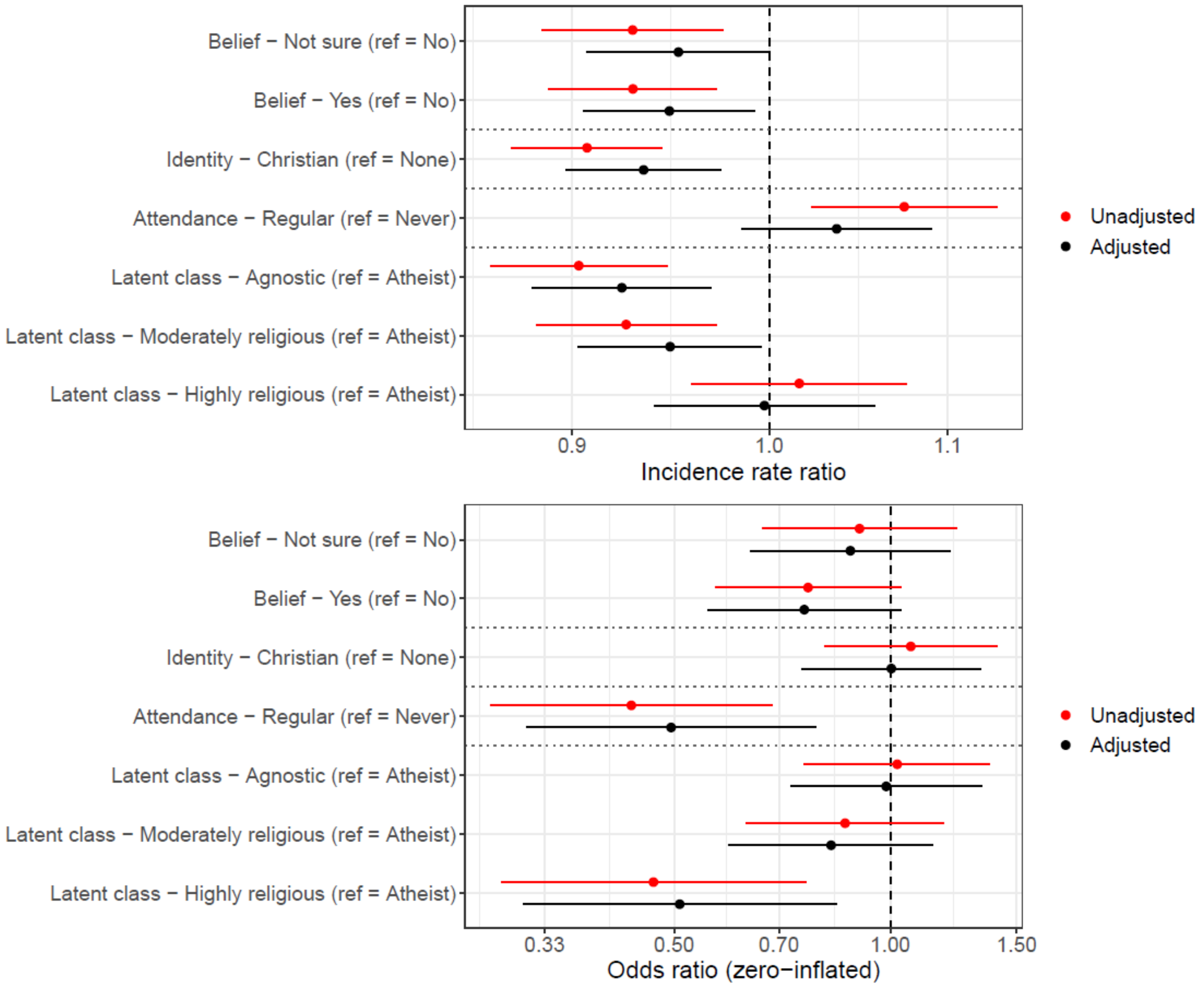

Figure S28: Predicted total number of actions (excluding ones which may be prohibitively costly) performed due to climate change for four religious exposures (belief, identity, attendance and latent classes) based on the mothers zero-inflated Poisson regression models.

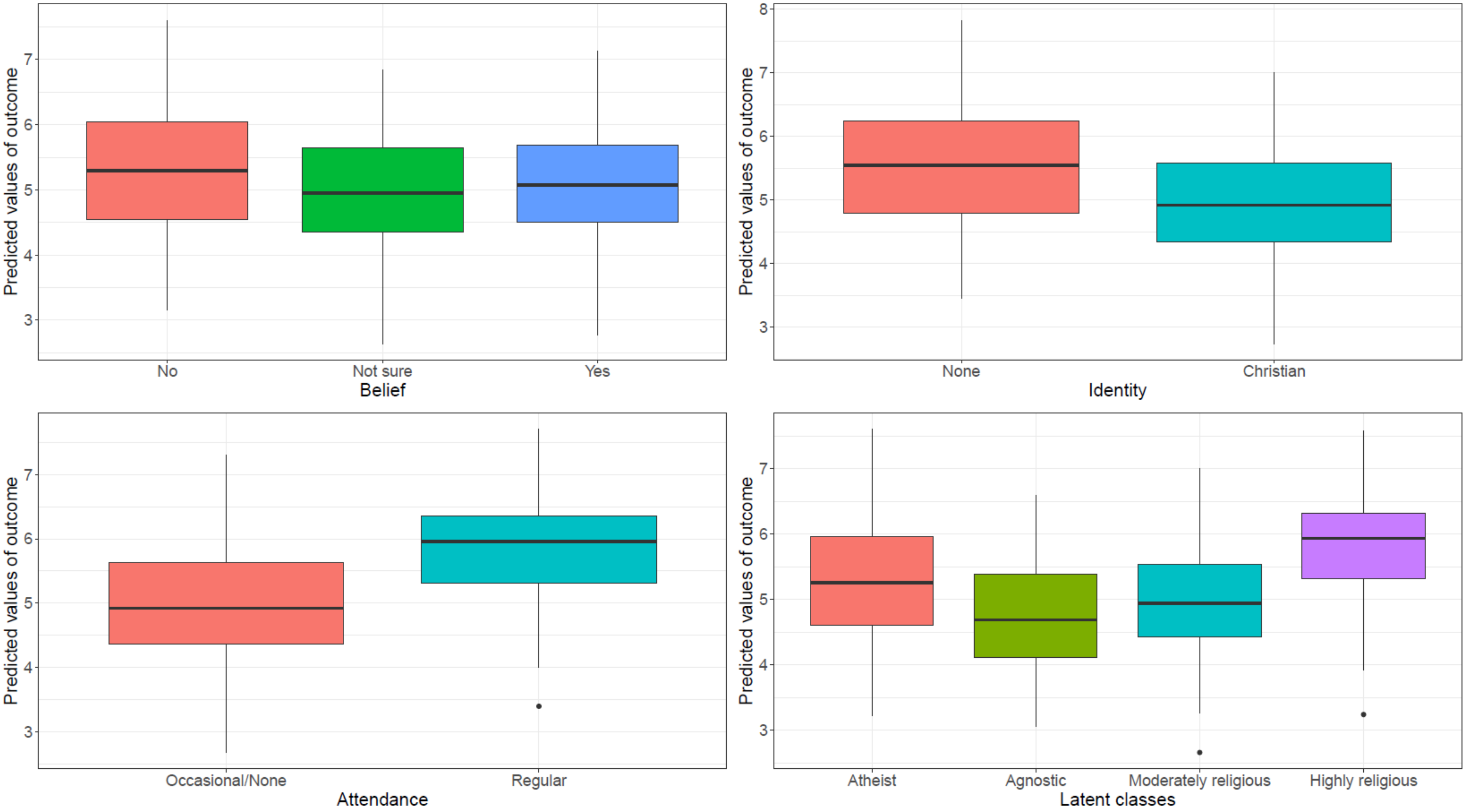

Figure S29: Predicted total number of actions (excluding ones which may be prohibitively costly) performed due to climate change for the religious identity (with the Christian denominations separated) as the exposure based on the mothers zero-inflated Poisson regression models.

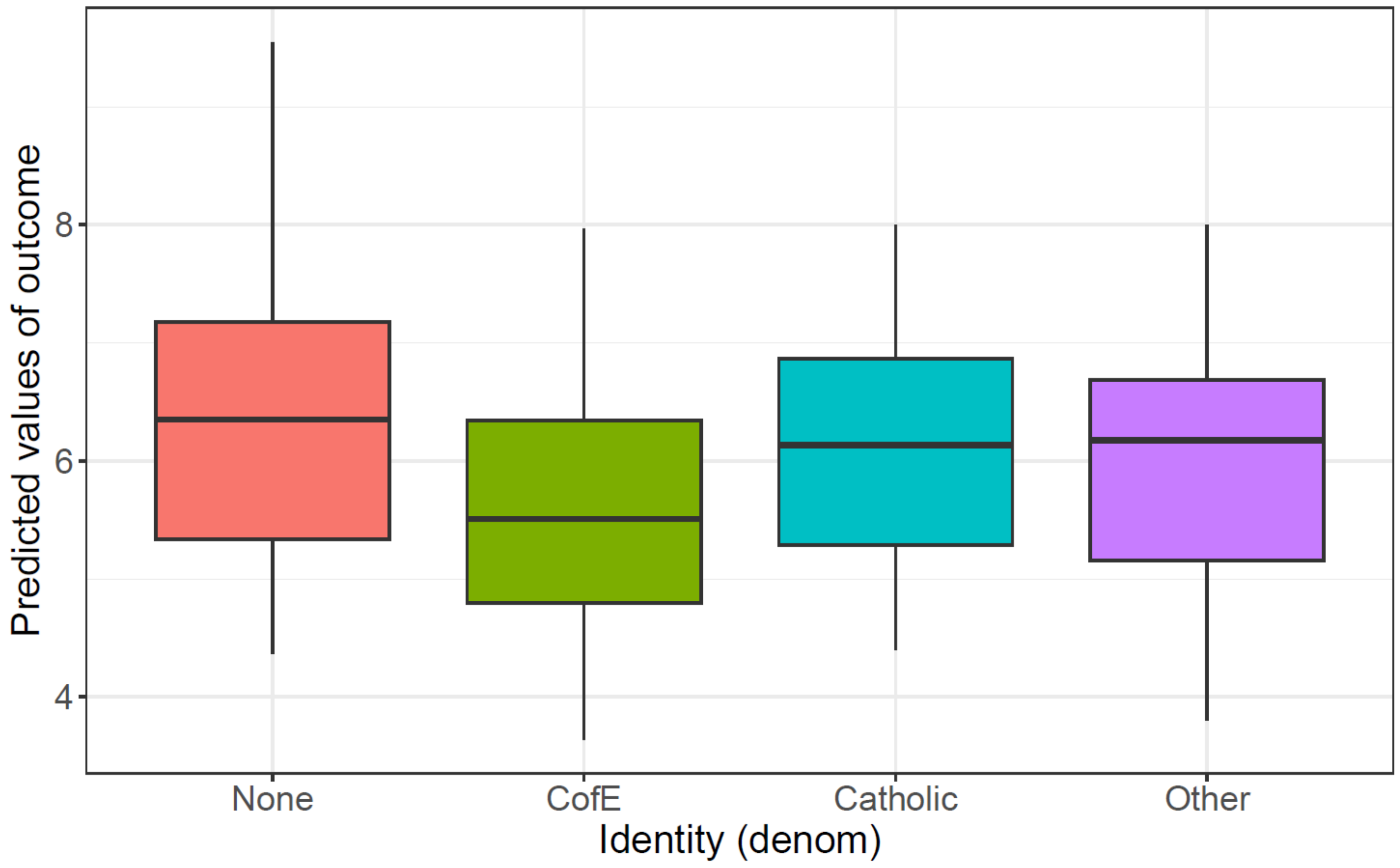

Figure S30: Results of the mothers multinomial regression models with 'changed the way travel locally' as the outcome for four religious exposures (belief [*n* = 2,552], identity [*n* = 2,526], attendance [*n* = 2,525], and latent classes [*n* = 2,558]; models are separated by dashed horizontal lines). See table S19 for full results.

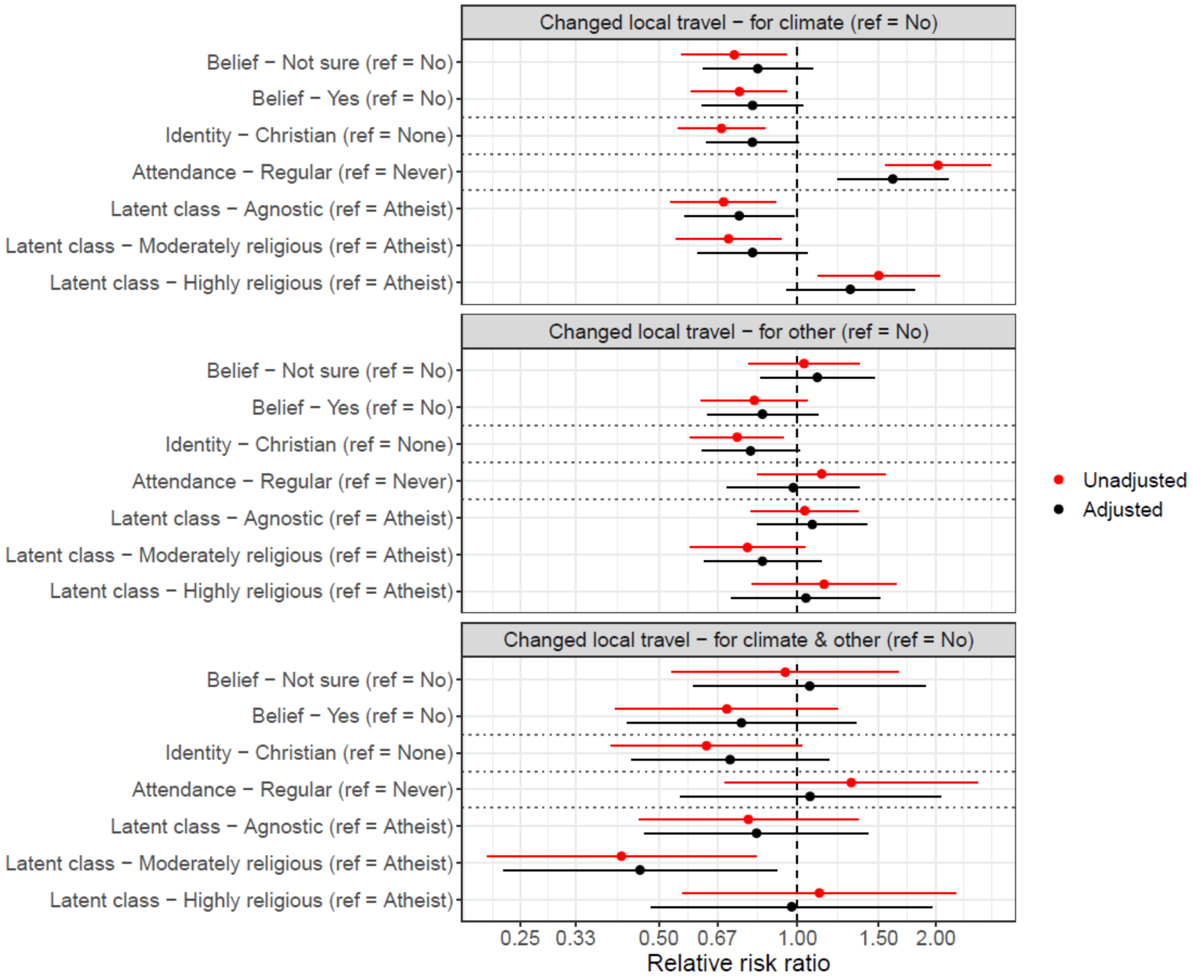

Figure S31: Predicted probabilities of the mothers multinomial regression models with 'changed the way travel locally' as the outcome for four religious exposures (belief, identity, attendance and latent classes).

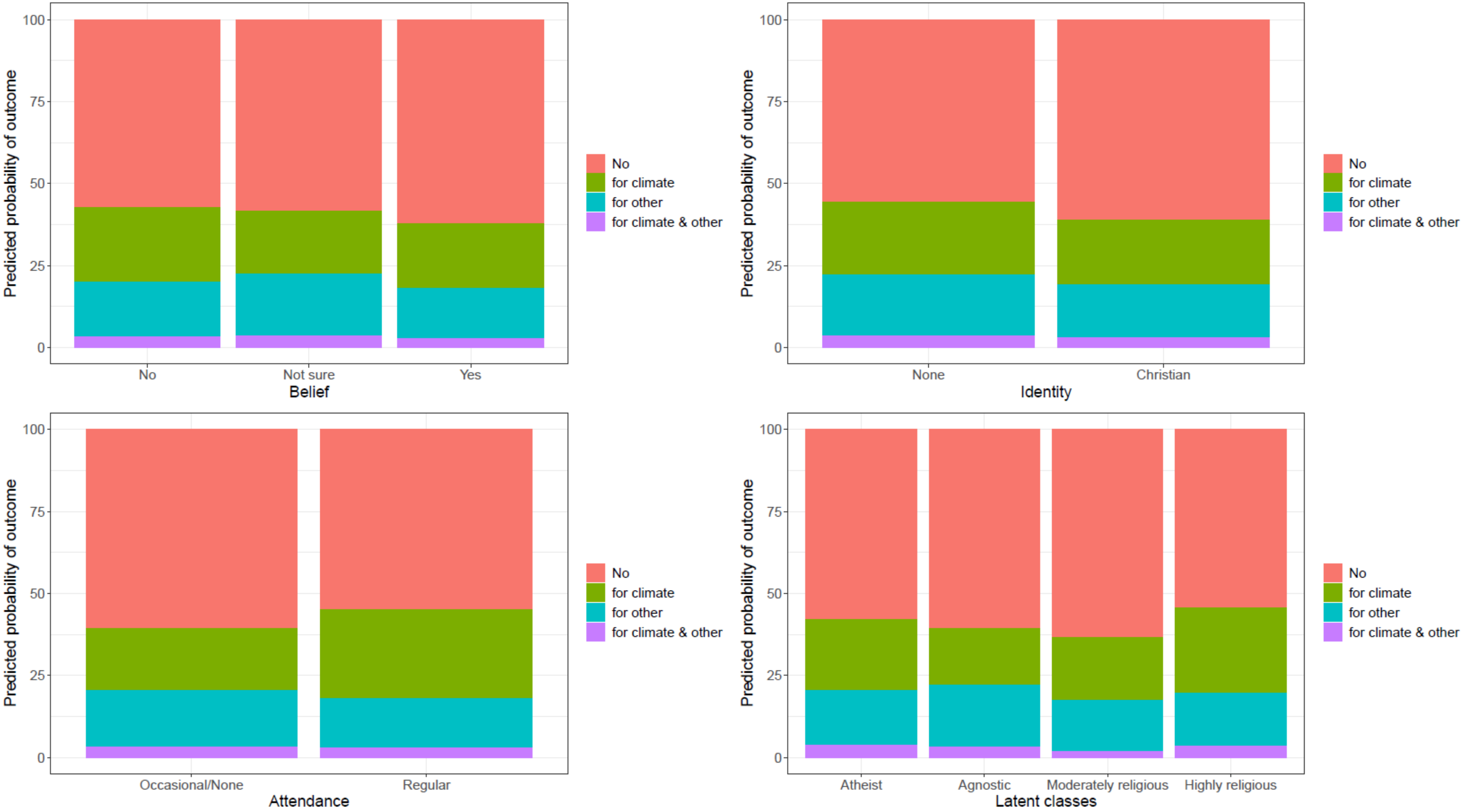

Figure S32: Predicted probabilities of the mothers multinomial regression models with 'changed the way travel locally' as the outcome and the religious identity (with the Christian denominations separated) as the exposure.

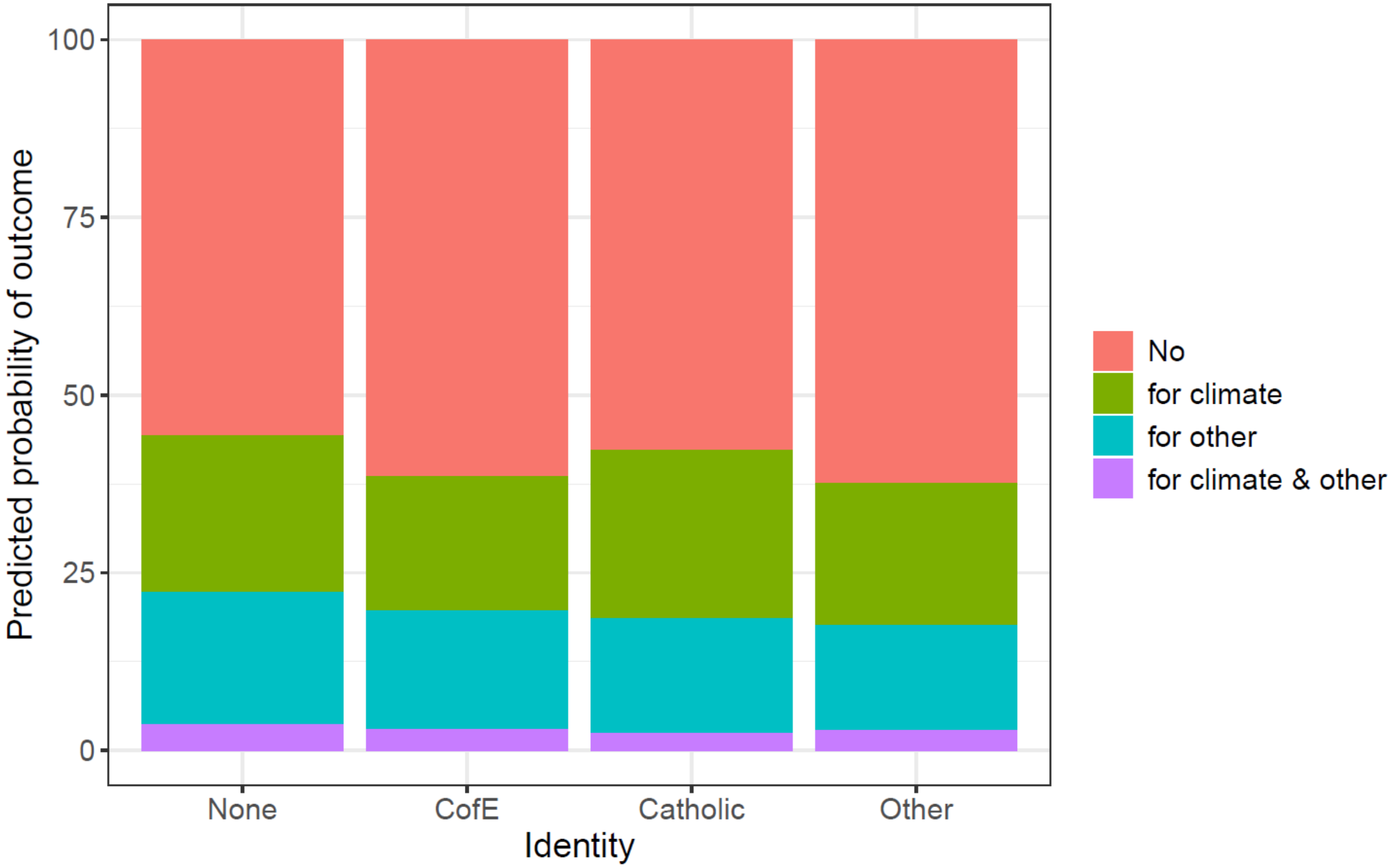

Figure S33: Results of the mothers multinomial regression models with 'reduced household waste' as the outcome for four religious exposures (belief [*n* = 2,560], identity [*n* = 2,534], attendance [*n* = 2,534], and latent classes [*n* = 2,566]; models are separated by dashed horizontal lines). See table S19 for full results.

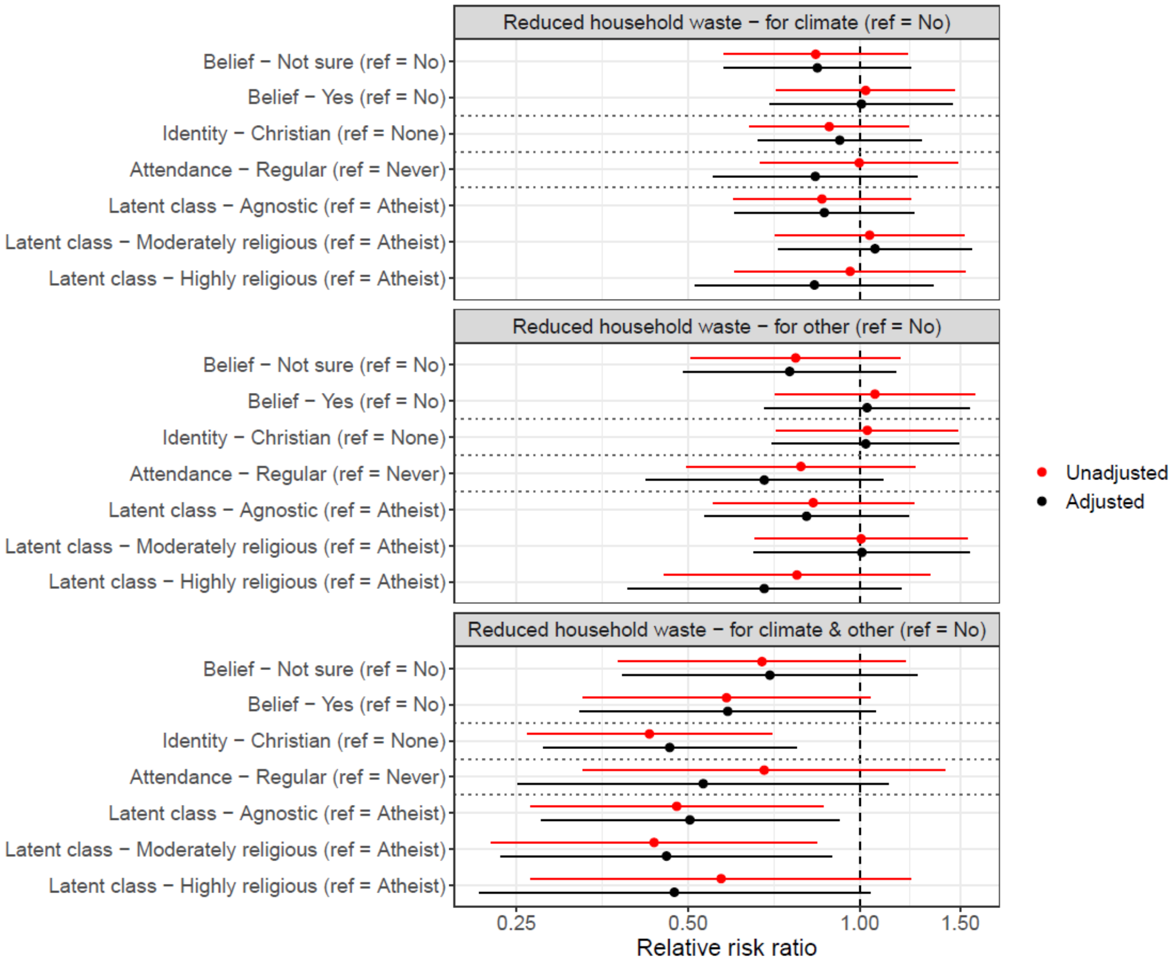

Figure S34: Predicted probabilities of the mothers multinomial regression models with 'reduced household waste' as the outcome for four religious exposures (belief, identity, attendance and latent classes).

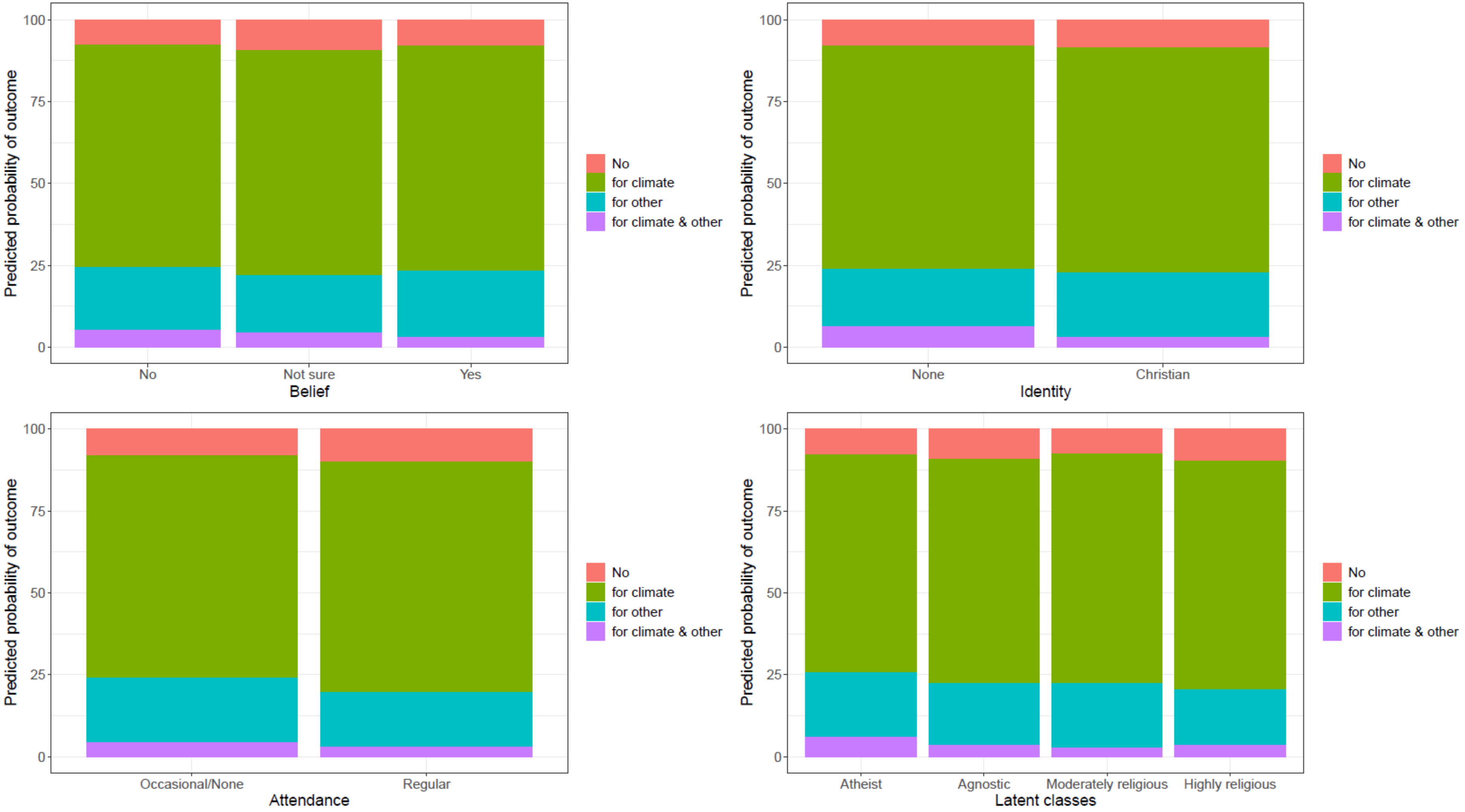

Figure S35: Predicted probabilities of the mothers multinomial regression models with 'reduced household waste' as the outcome and the religious identity (with the Christian denominations separated) as the exposure.

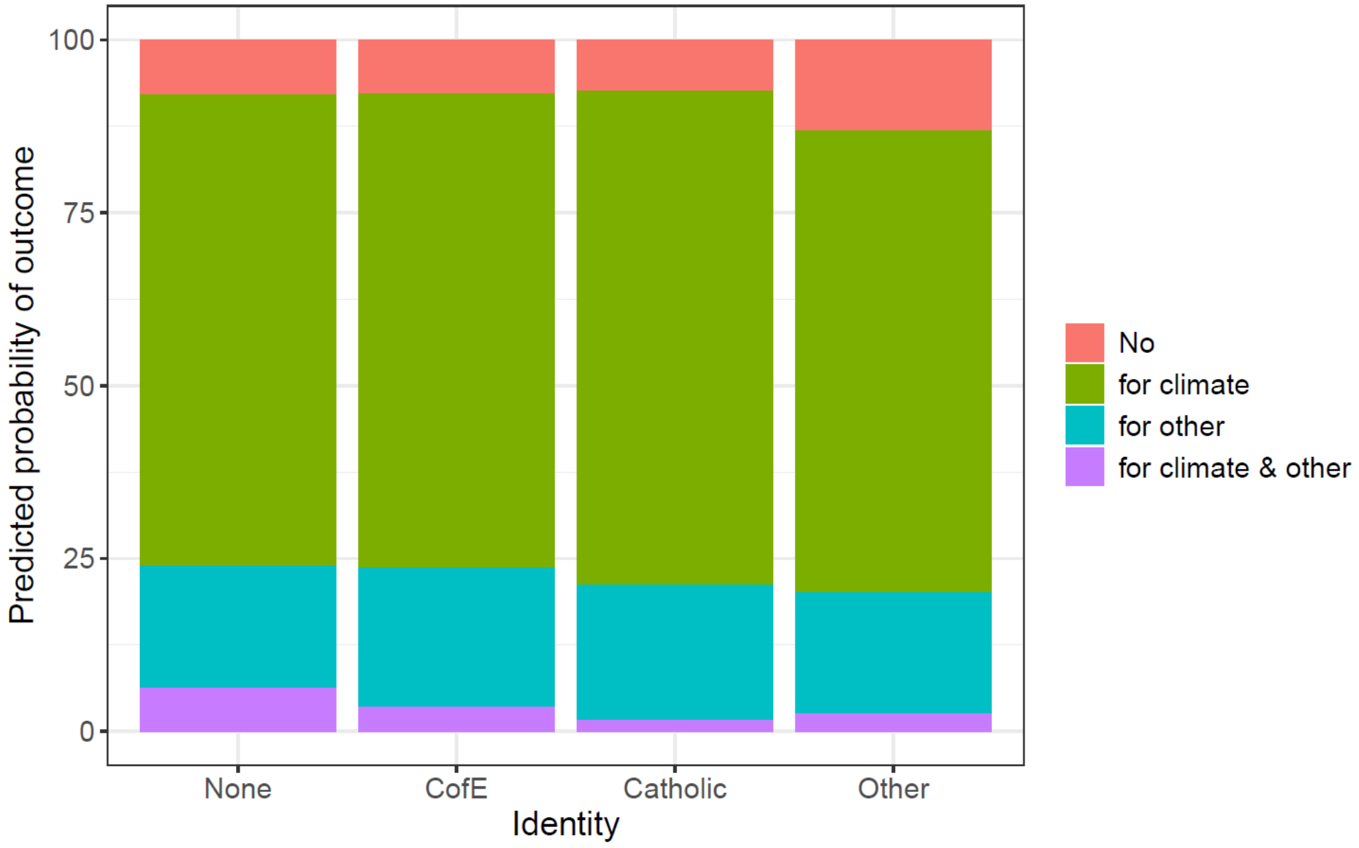

Figure S36: Results of the mothers multinomial regression models with 'reduced energy use at home' as the outcome for four religious exposures (belief [*n* = 2,562], identity [*n* = 2,535], attendance [*n* = 2,535], and latent classes [*n* = 2,568]; models are separated by dashed horizontal lines). See table S19 for full results.

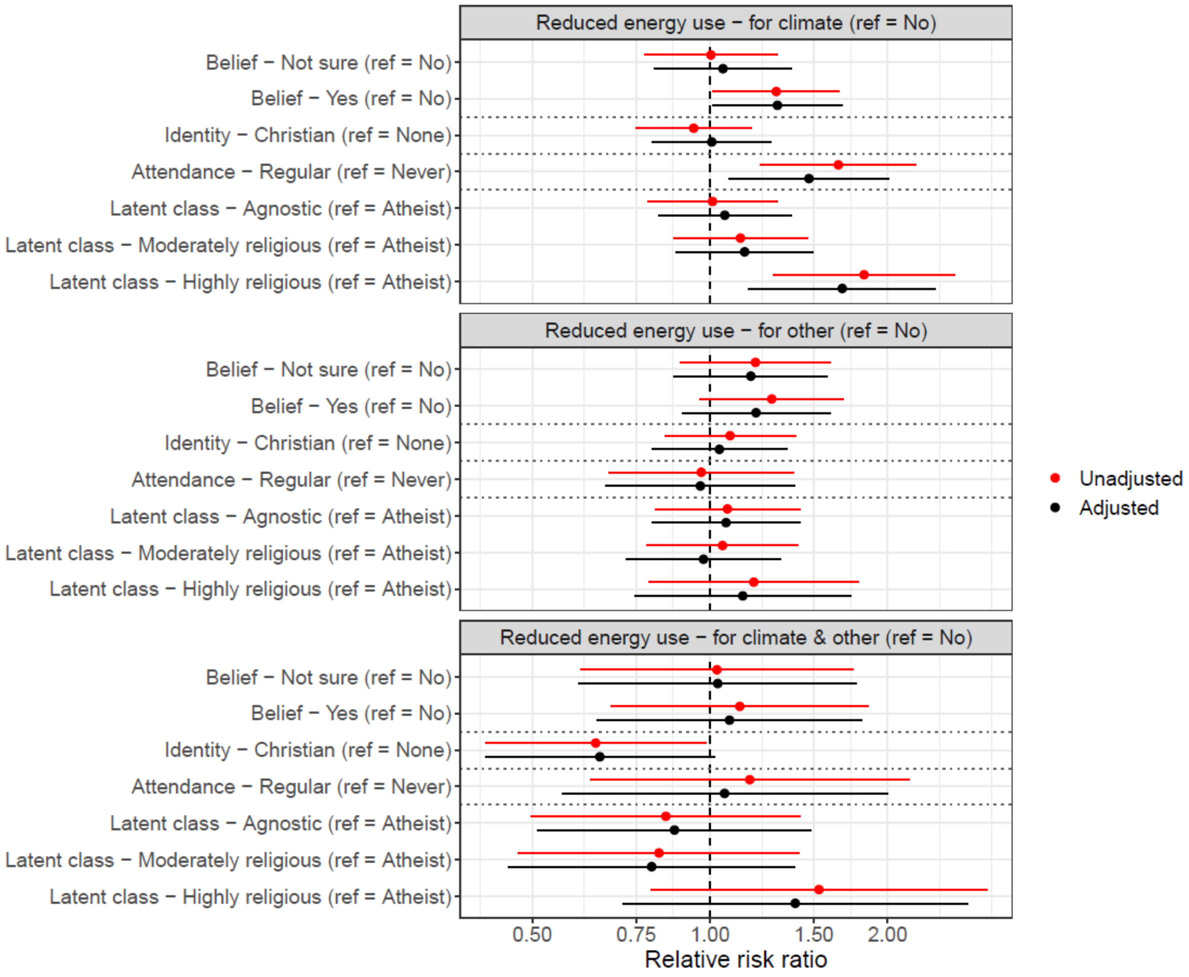

Figure S37: Predicted probabilities of the mothers multinomial regression models with 'reduced energy use at home' as the outcome for four religious exposures (belief, identity, attendance and latent classes).

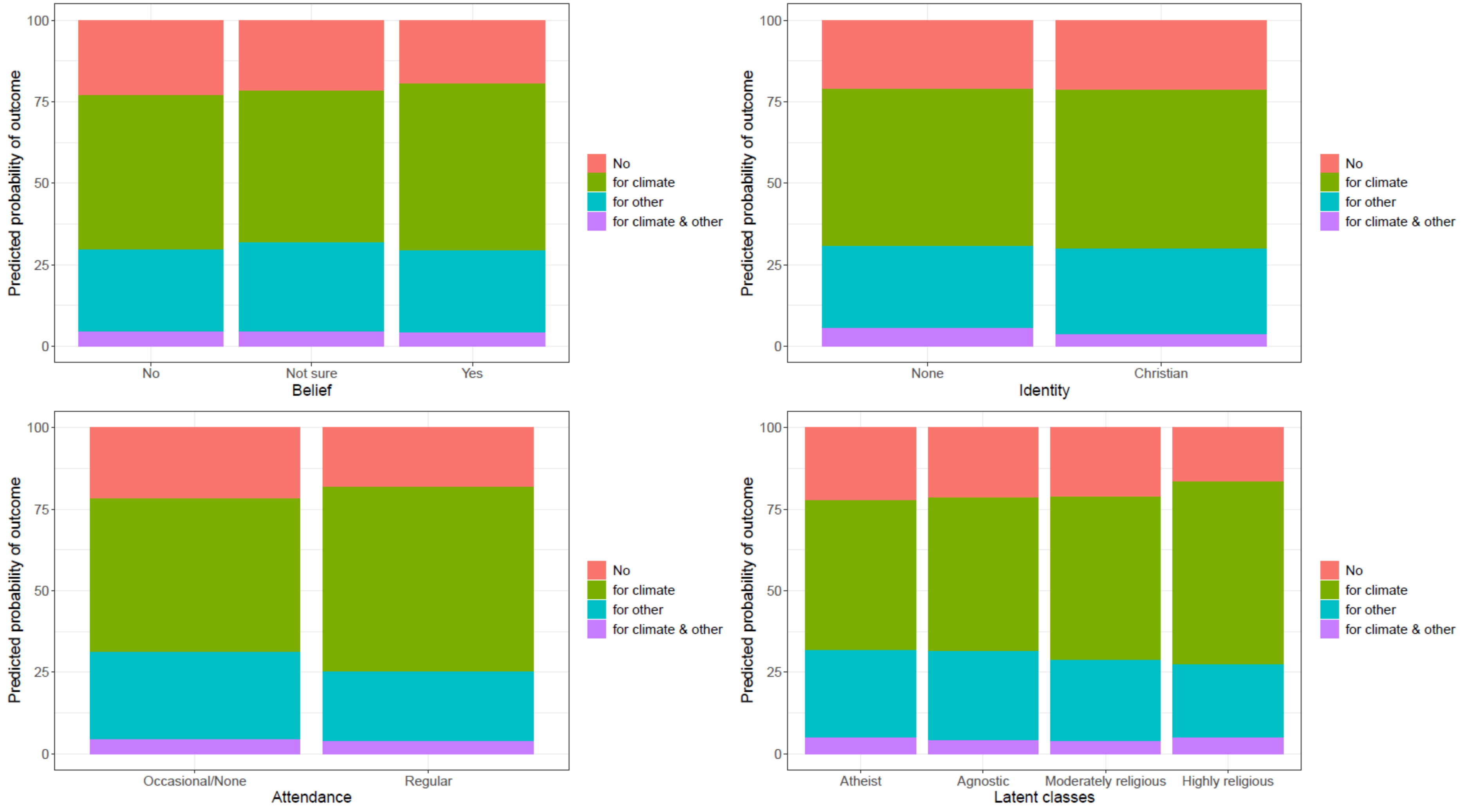

Figure S38: Predicted probabilities of the mothers multinomial regression models with 'reduced energy use at home' as the outcome and the religious identity (with the Christian denominations separated) as the exposure.

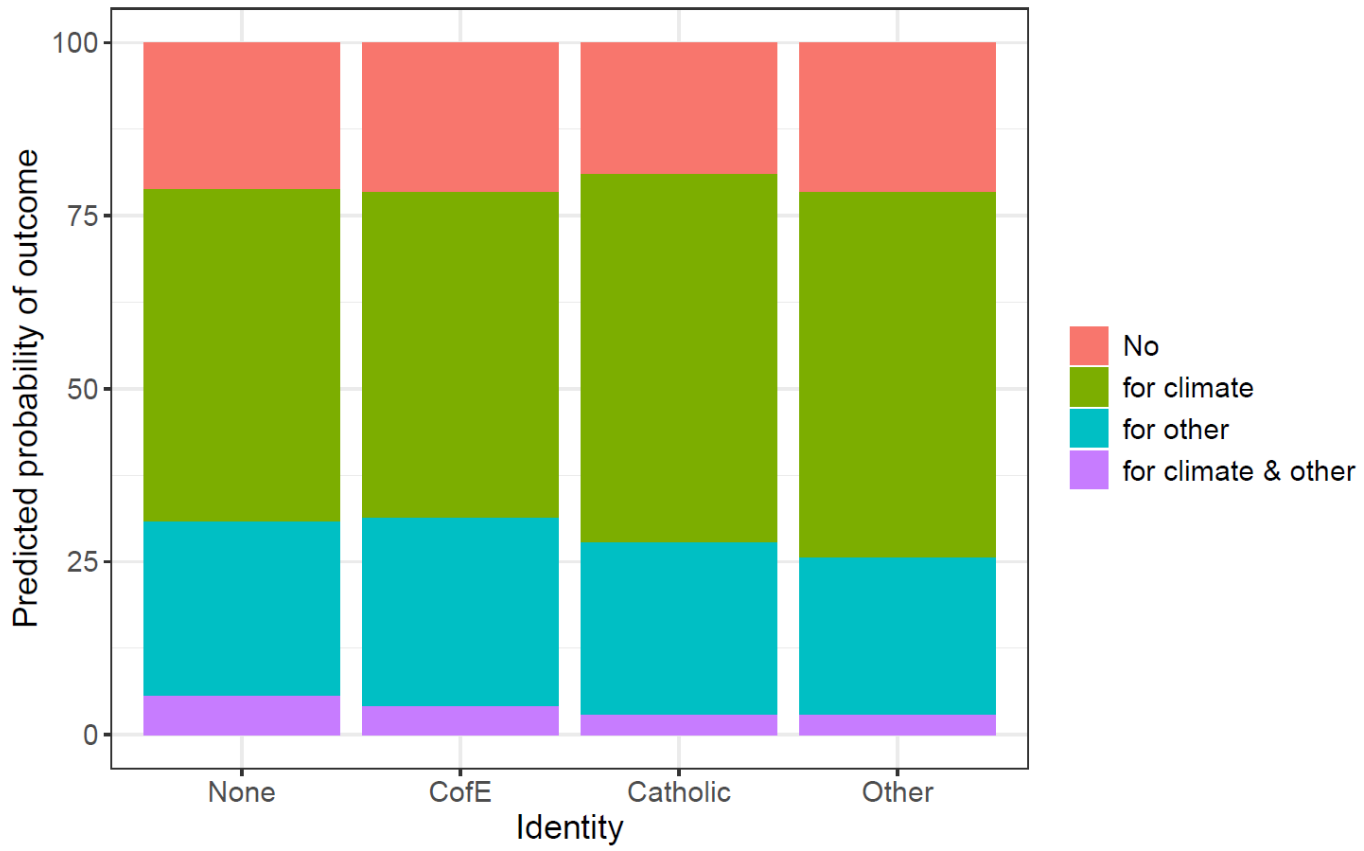

Figure S39: Results of the mothers multinomial regression models with 'changed what buy' as the outcome for four religious exposures (belief [*n* = 2,545], identity [*n* = 2,518], attendance [*n* = 2,518], and latent classes [*n* = 2,551]; models are separated by dashed horizontal lines). See table S19 for full results.

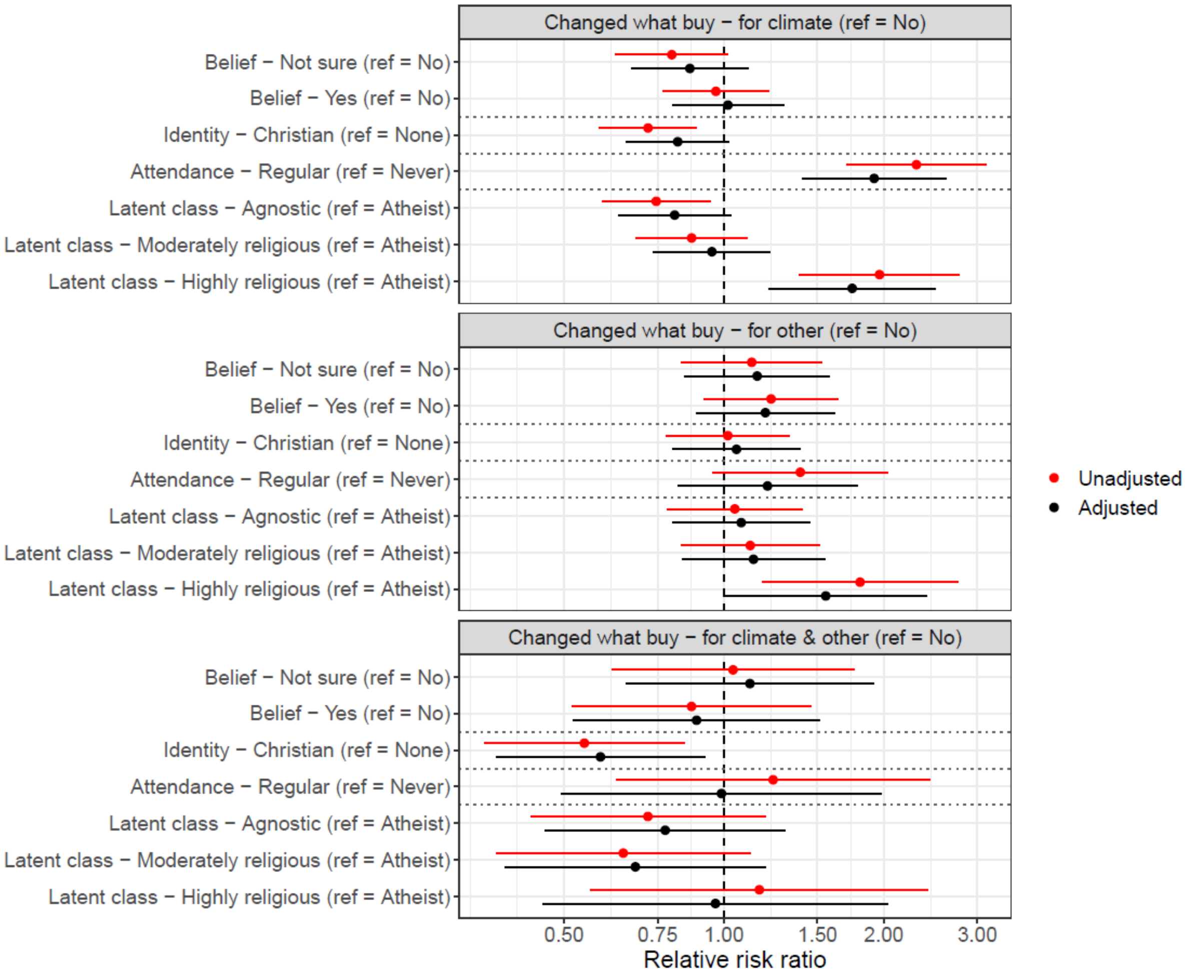

Figure S40: Predicted probabilities of the mothers multinomial regression models with 'changed what buy' as the outcome for four religious exposures (belief, identity, attendance and latent classes).

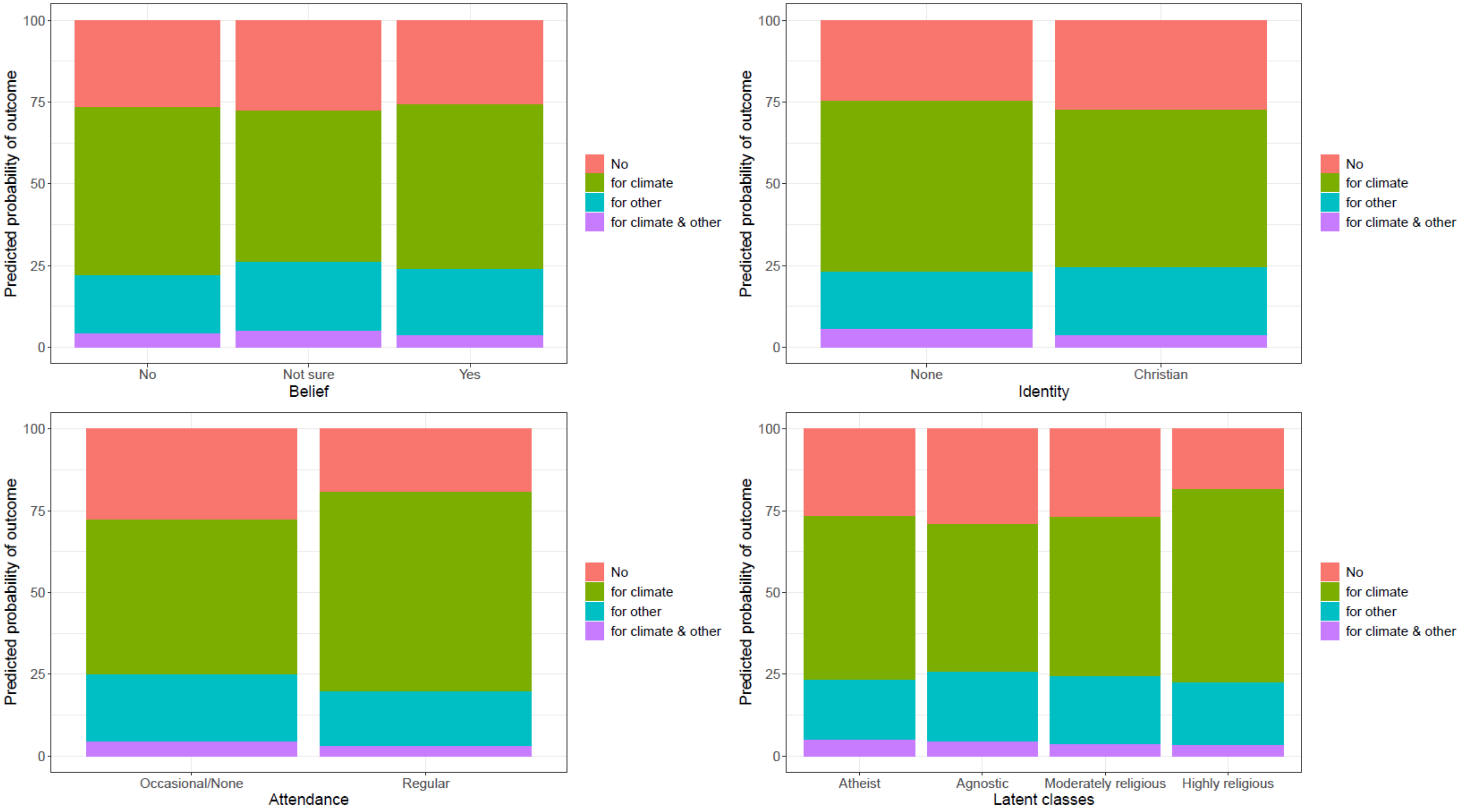

Figure S41: Predicted probabilities of the mothers multinomial regression models with ‘changed what buy’ as the outcome and the religious identity (with the Christian denominations separated) as the exposure.

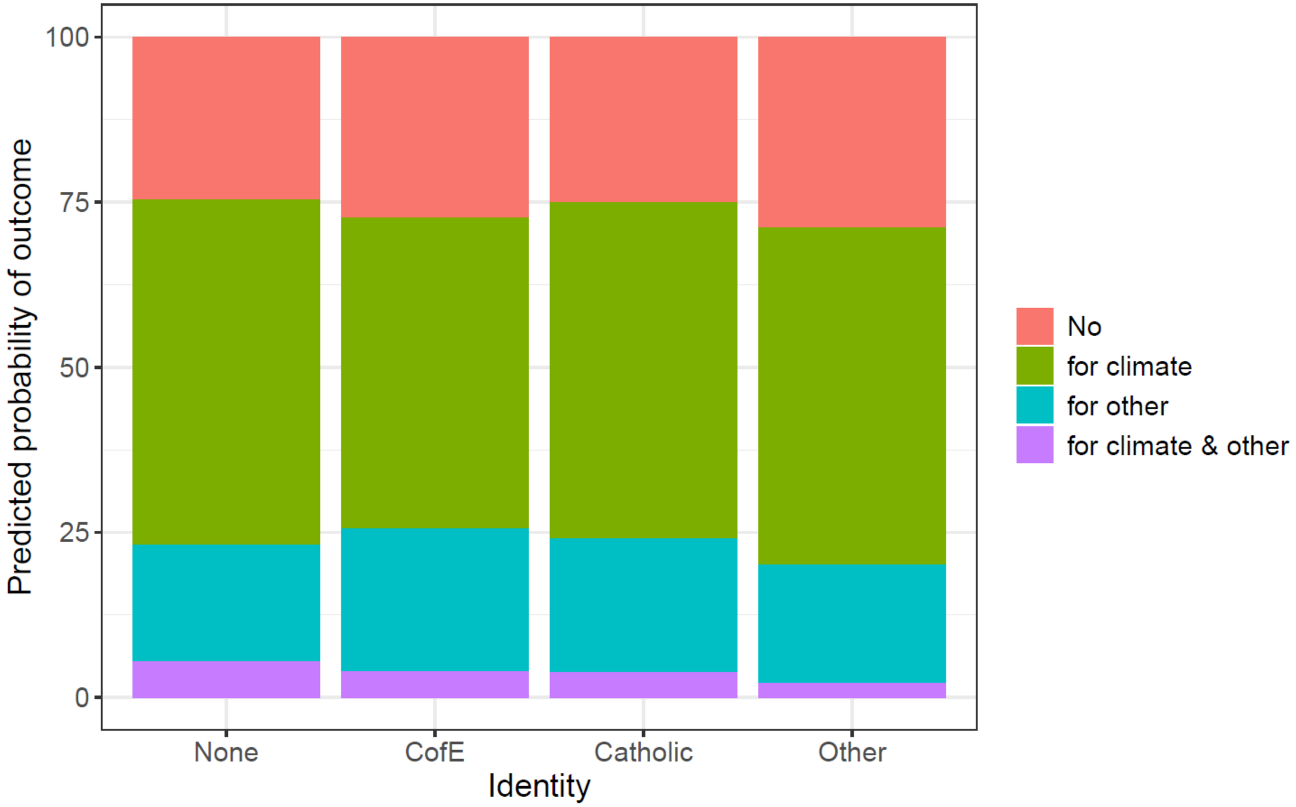

Figure S42: Results of the mothers multinomial regression models with 'reduced air travel' as the outcome for four religious exposures (belief [*n* = 2,537], identity [*n* = 2,511], attendance [*n* = 2,511], and latent classes [*n* = 2,543]; models are separated by dashed horizontal lines). See table S19 for full results.

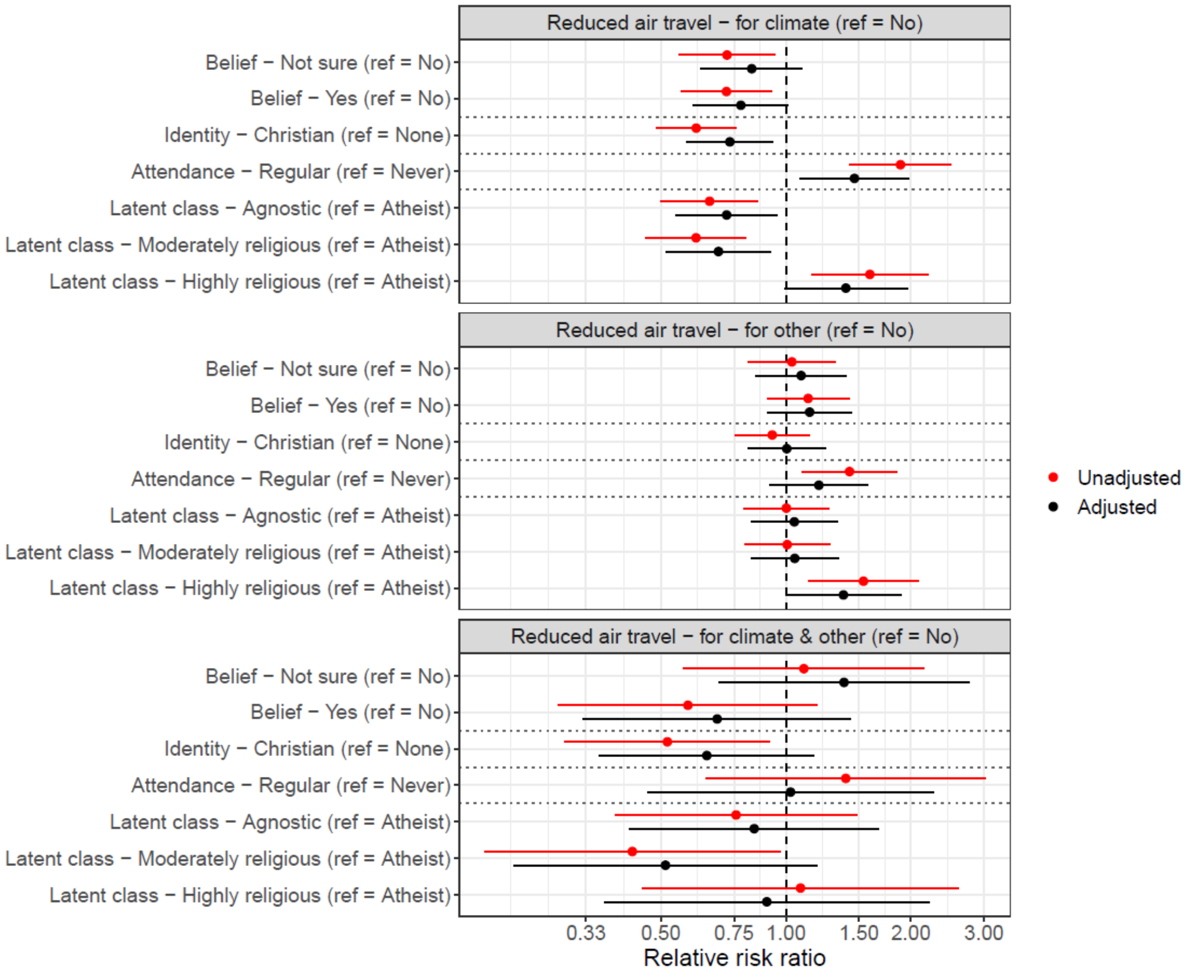

Figure S43: Predicted probabilities of the mothers multinomial regression models with 'reduced air travel' as the outcome for four religious exposures (belief, identity, attendance and latent classes).

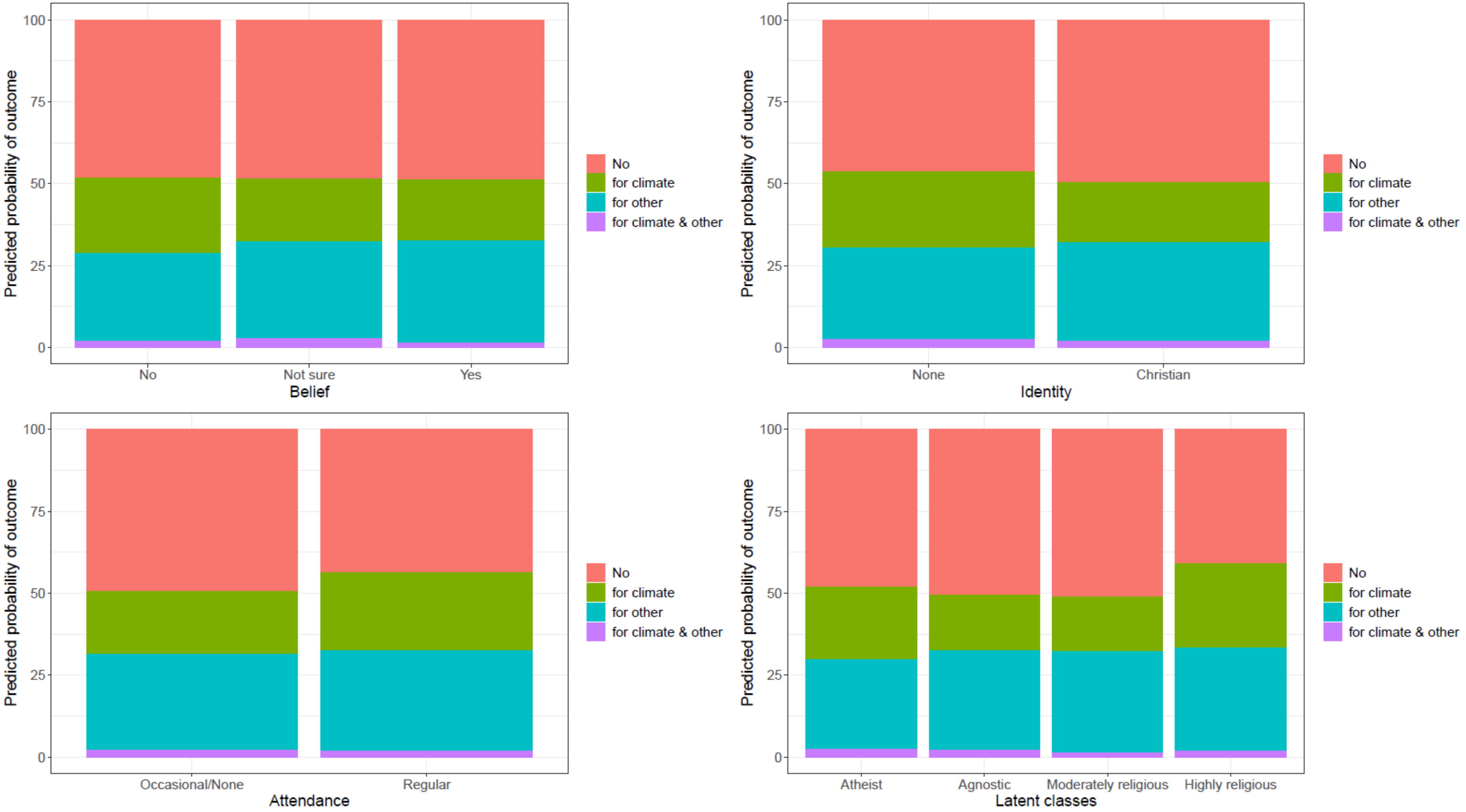

Figure S44: Predicted probabilities of the mothers multinomial regression models with 'reduced air travel' as the outcome and the religious identity (with the Christian denominations separated) as the exposure.

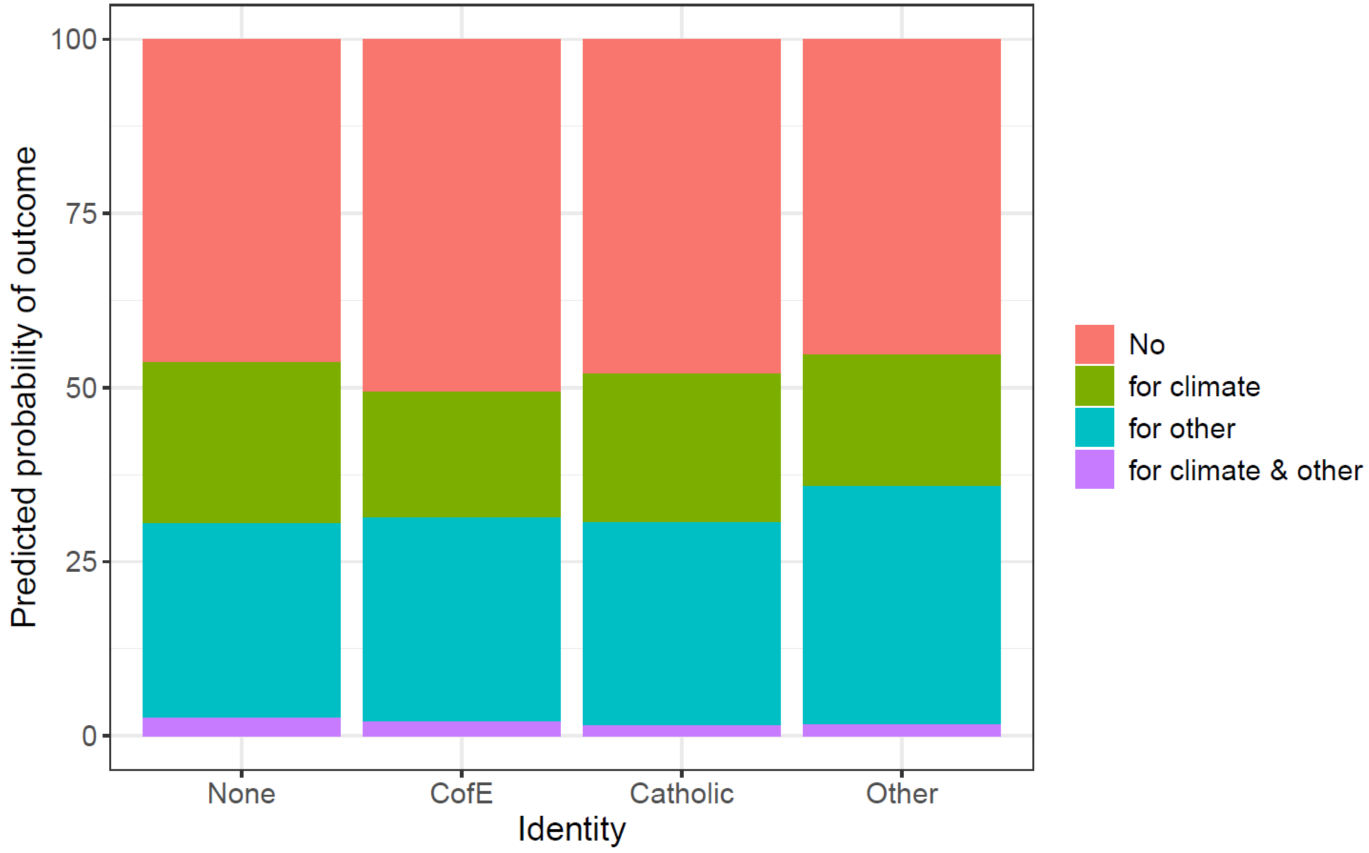

Figure S45: Results of the mothers multinomial regression models with 'bought or hired an electric or hybrid vehicle' as the outcome for four religious exposures (belief [*n* = 2,557], identity [*n* = 2,530], attendance [*n* = 2,530], and latent classes [*n* = 2,563]; models are separated by dashed horizontal lines). See table S19 for full results.

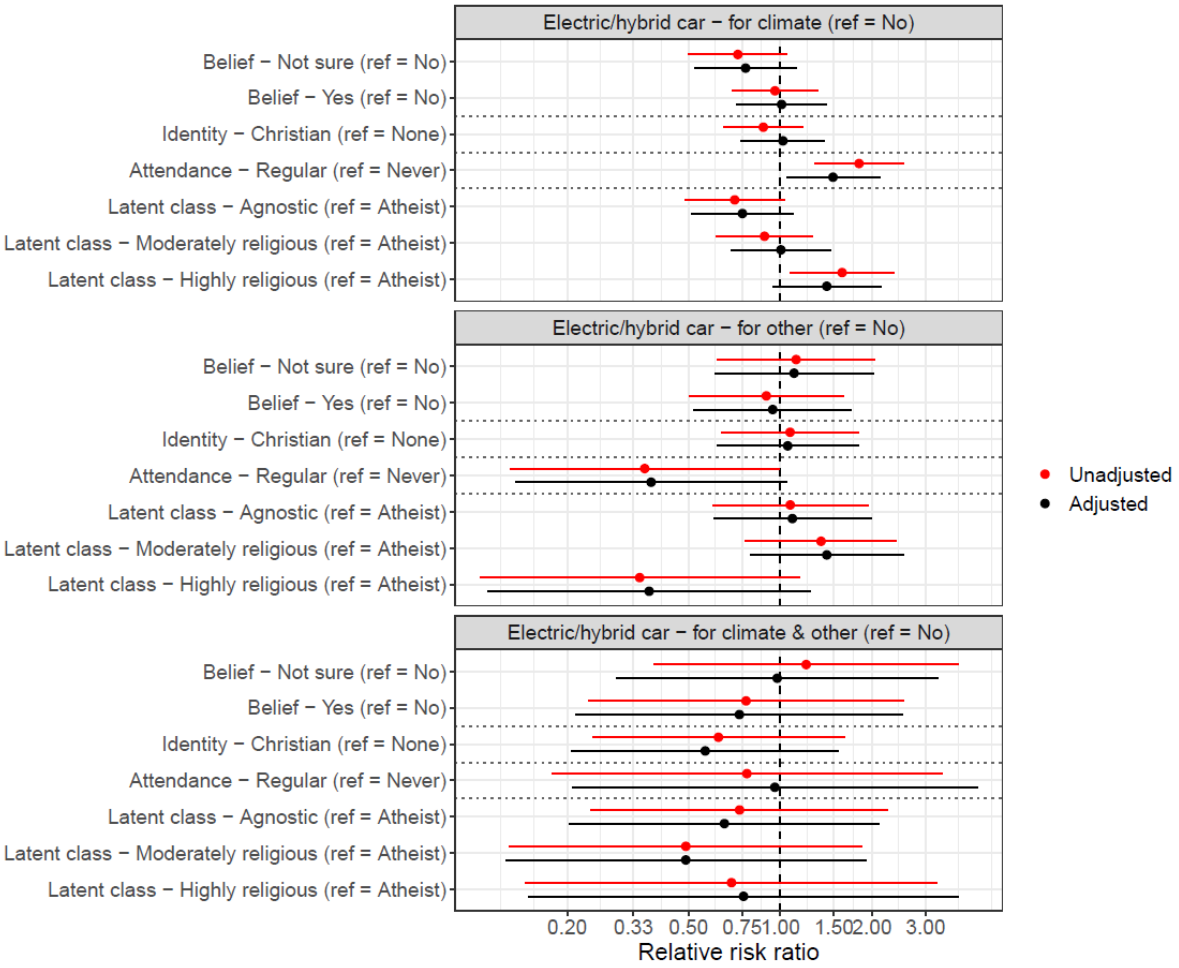

Figure S46: Predicted probabilities of the mothers multinomial regression models with 'bought or hired an electric or hybrid vehicle' as the outcome for four religious exposures (belief, identity, attendance and latent classes).

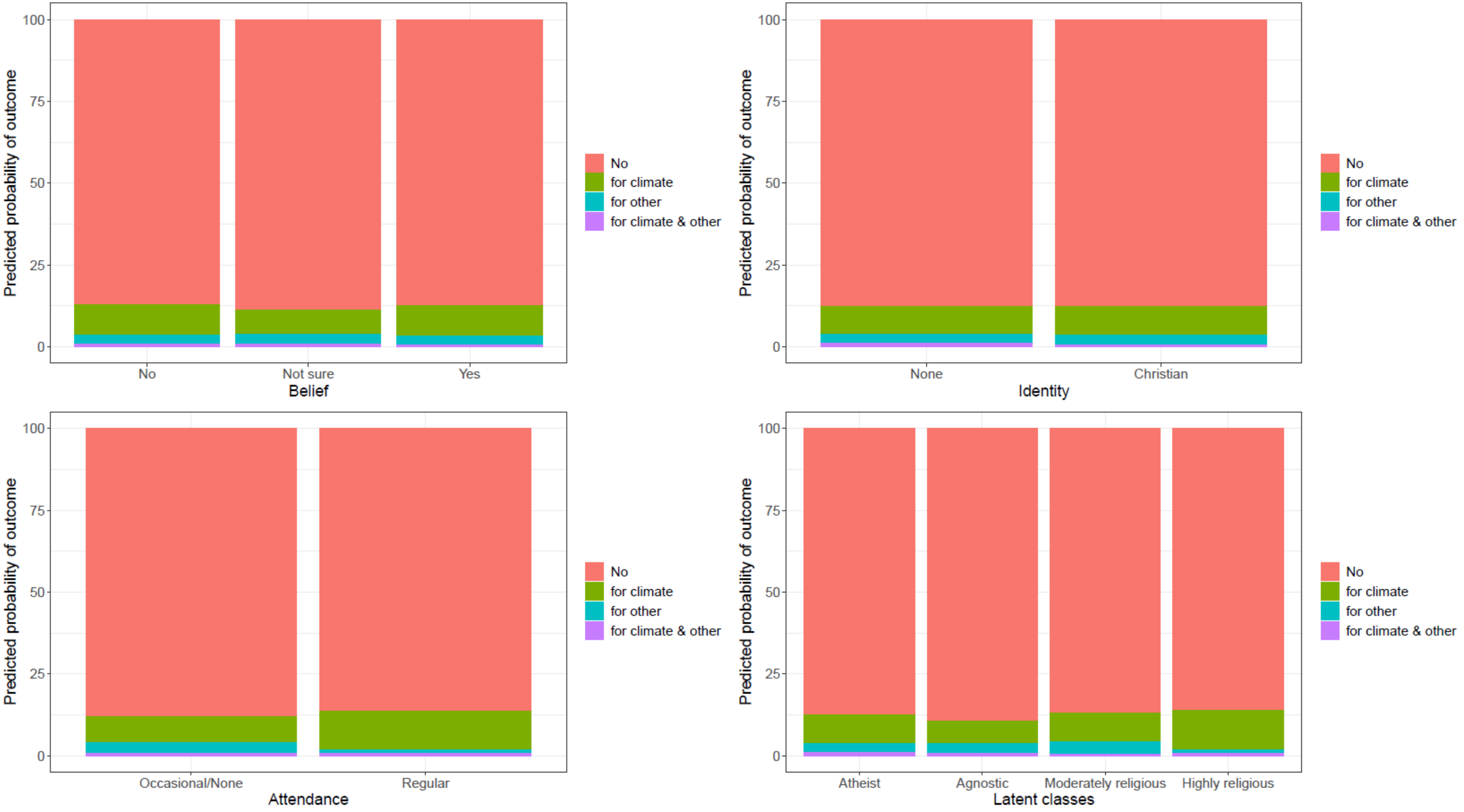

Figure S47: Predicted probabilities of the mothers multinomial regression models with 'bought or hired an electric or hybrid vehicle' as the outcome and the religious identity (with the Christian denominations separated) as the exposure.

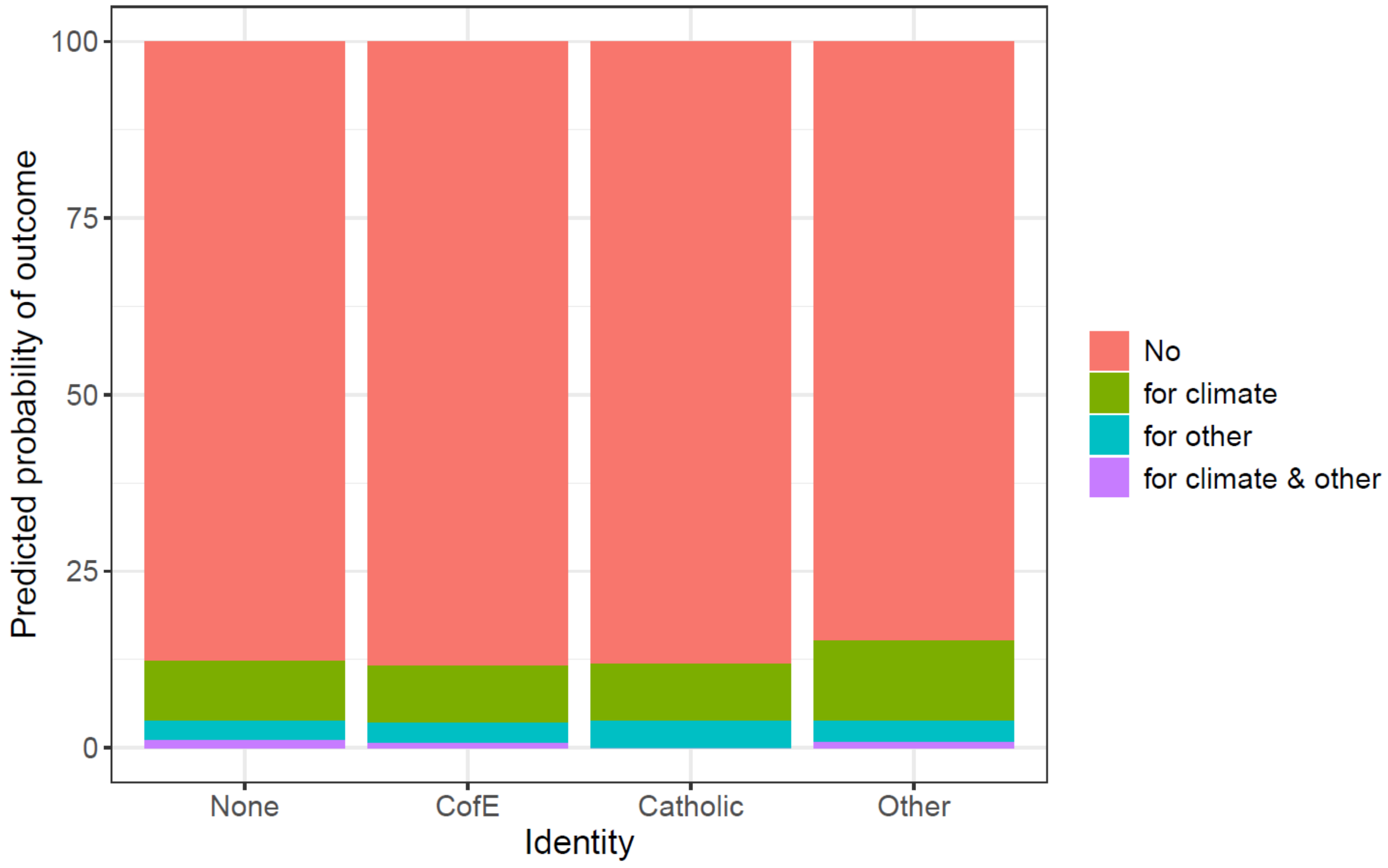

Figure S48: Results of the mothers multinomial regression models with 'bought foods produced locally' as the outcome for four religious exposures (belief [*n* = 2,544], identity [*n* = 2,517], attendance [*n* = 2,517], and latent classes [*n* = 2,550]; models are separated by dashed horizontal lines). See table S19 for full results.

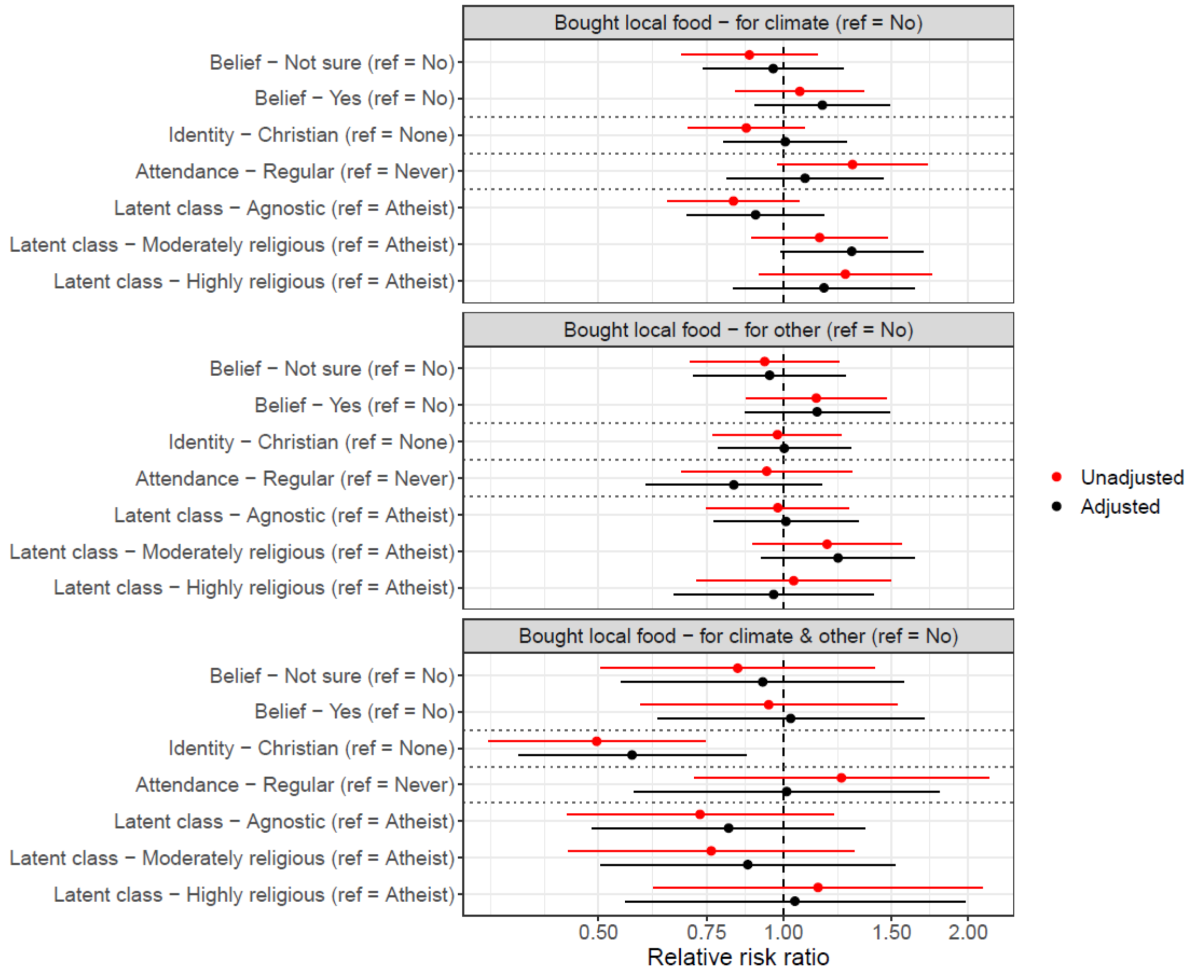

Figure S49: Predicted probabilities of the mothers multinomial regression models with 'bought foods produced locally' as the outcome for four religious exposures (belief, identity, attendance and latent classes).

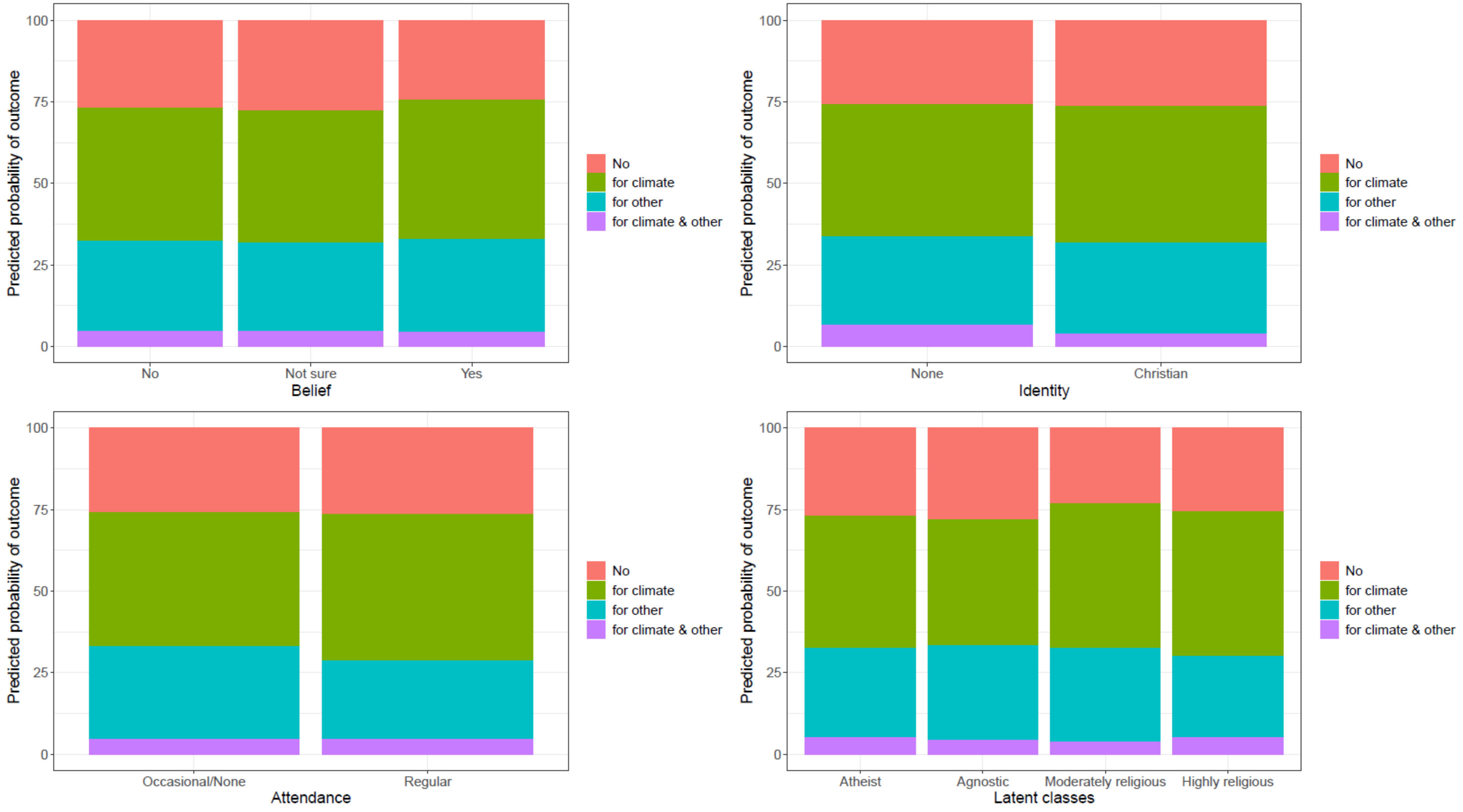

Figure S50: Predicted probabilities of the mothers multinomial regression models with 'bought foods produced locally' as the outcome and the religious identity (with the Christian denominations separated) as the exposure.

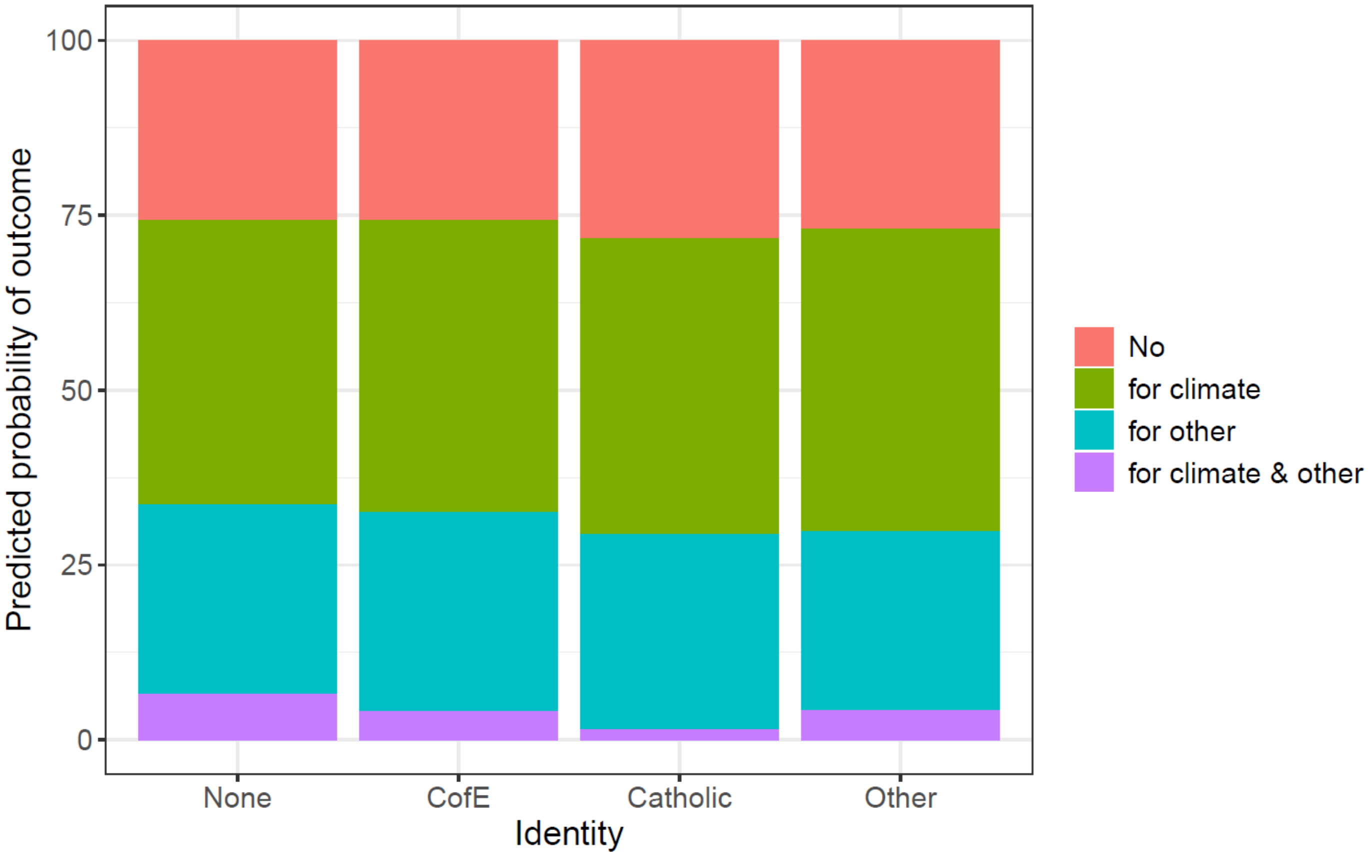

Figure S51: Results of the mothers multinomial regression models with 'recycled or upcycled more' as the outcome for four religious exposures (belief [*n* = 2,555], identity [*n* = 2,529], attendance [*n* = 2,528], and latent classes [*n* = 2,561]; models are separated by dashed horizontal lines). See table S19 for full results.

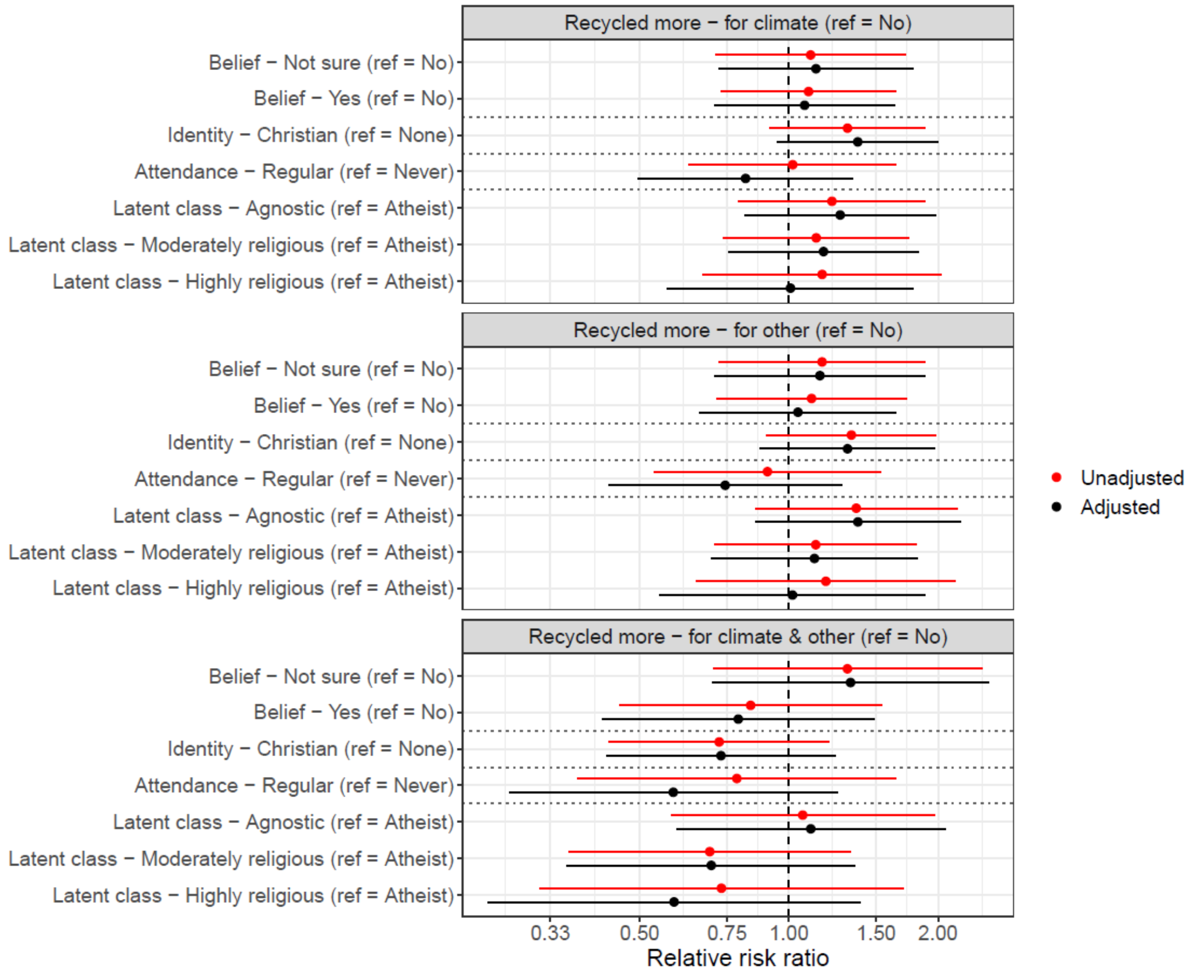

Figure S52: Predicted probabilities of the mothers multinomial regression models with ‘recycled or upcycled more’ as the outcome for four religious exposures (belief, identity, attendance and latent classes).

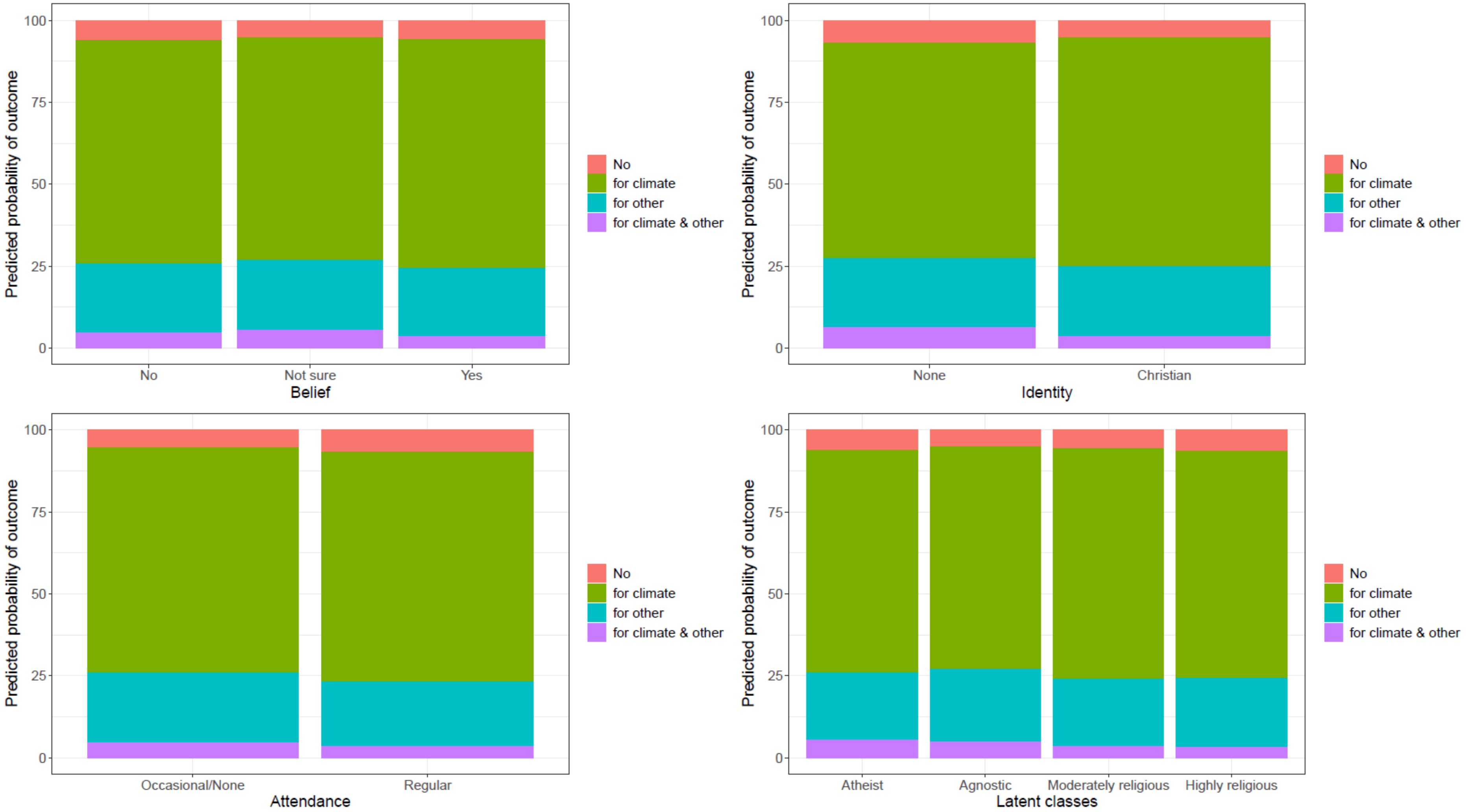

Figure S53: Predicted probabilities of the mothers multinomial regression models with ‘recycled or upcycled more’ as the outcome and the religious identity (with the Christian denominations separated) as the exposure.

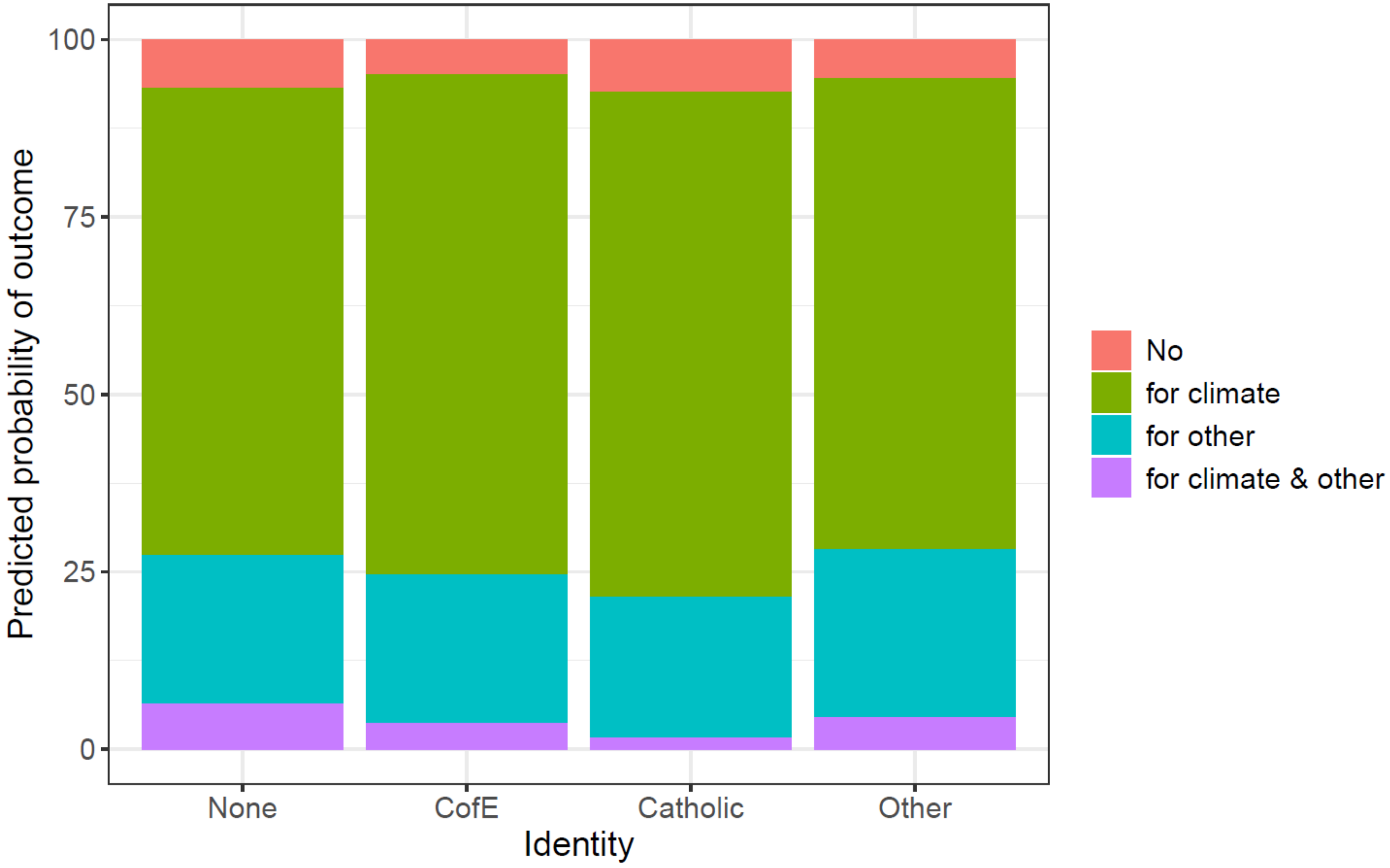

Figure S54: Results of the mothers multinomial regression models with 'reduced the amount of plastic used' as the outcome for four religious exposures (belief [*n* = 2,562], identity [*n* = 2,535], attendance [*n* = 2,535], and latent classes [*n* = 2,568]; models are separated by dashed horizontal lines). See table S19 for full results.

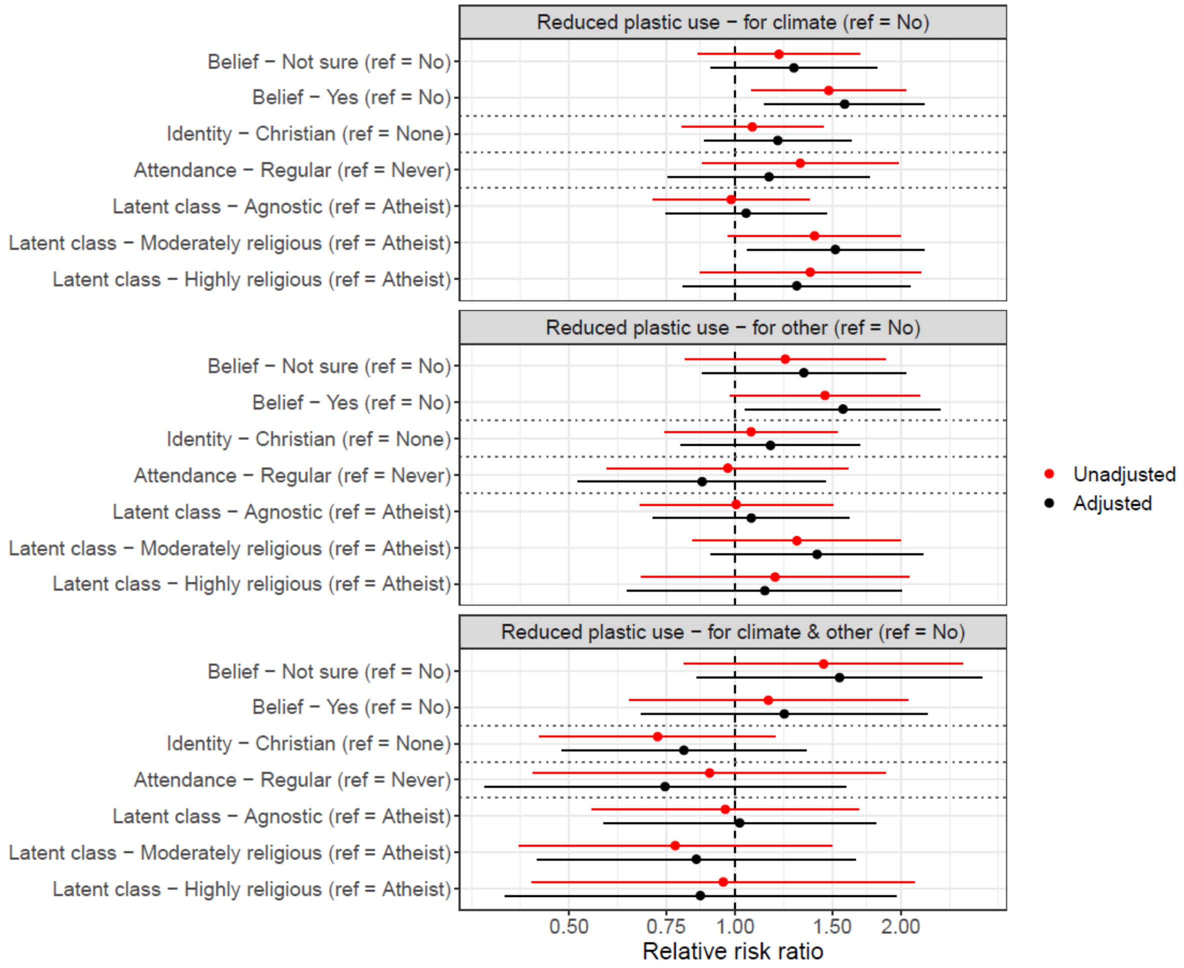

Figure S55: Predicted probabilities of the mothers multinomial regression models with 'reduced the amount of plastic used' as the outcome for four religious exposures (belief, identity, attendance and latent classes).

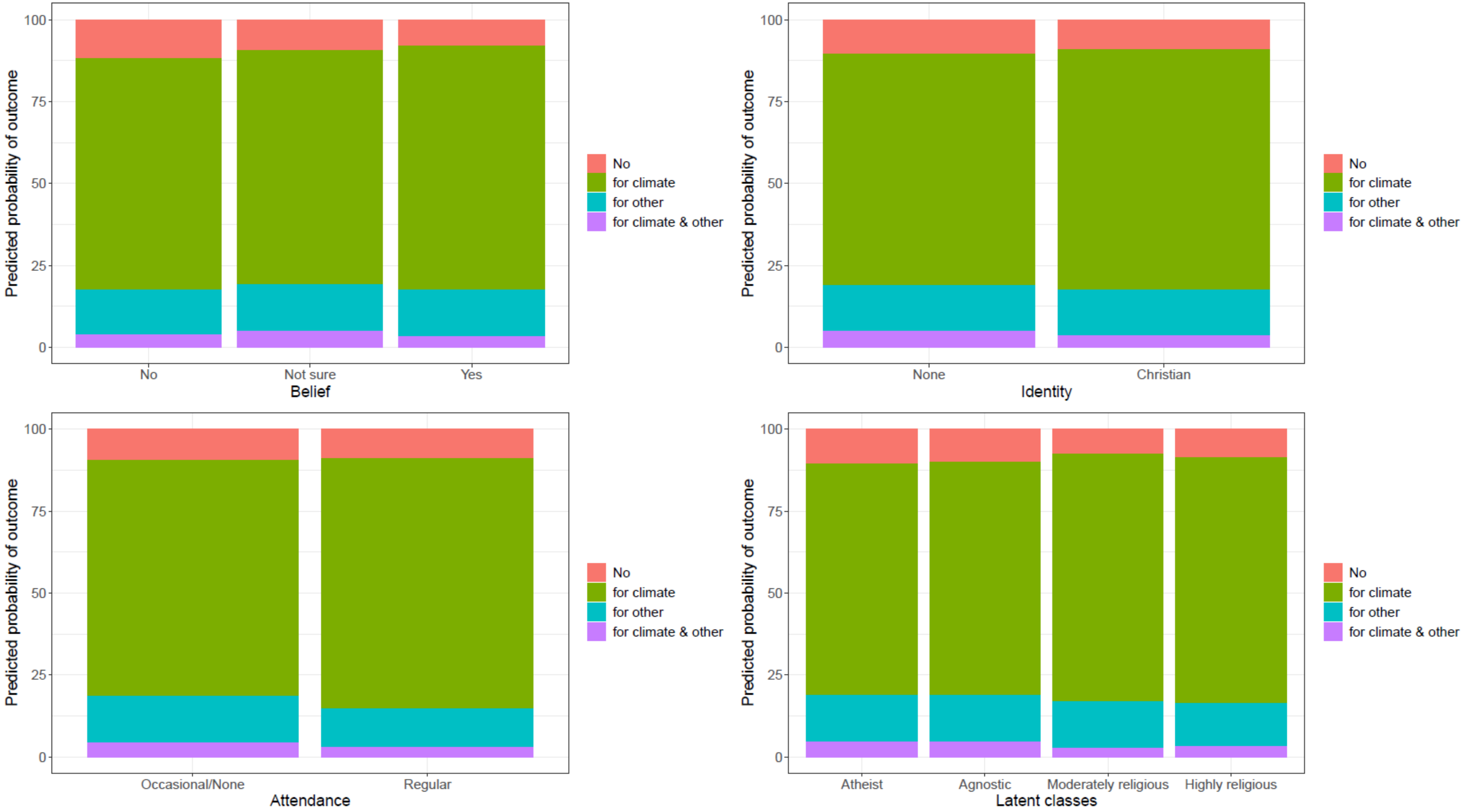

Figure S56: Predicted probabilities of the mothers multinomial regression models with 'reduced the amount of plastic used' as the outcome and the religious identity (with the Christian denominations separated) as the exposure.

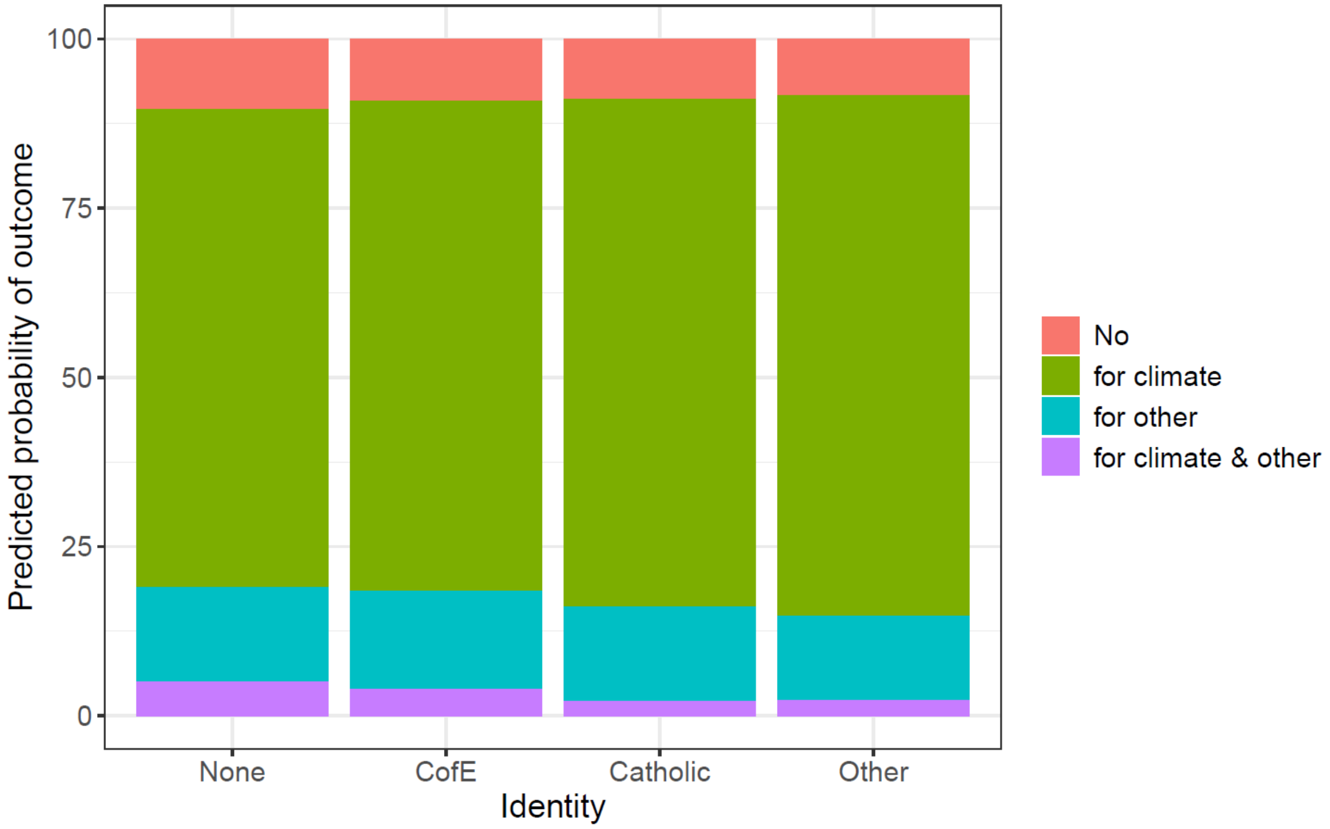

Figure S57: Results of the mothers multinomial regression models with ‘chosen sustainably sourced items’ as the outcome for four religious exposures (belief [*n* = 2,538], identity [*n* = 2,511], attendance [*n* = 2,511], and latent classes [*n* = 2,544]; models are separated by dashed horizontal lines). See table S19 for full results.

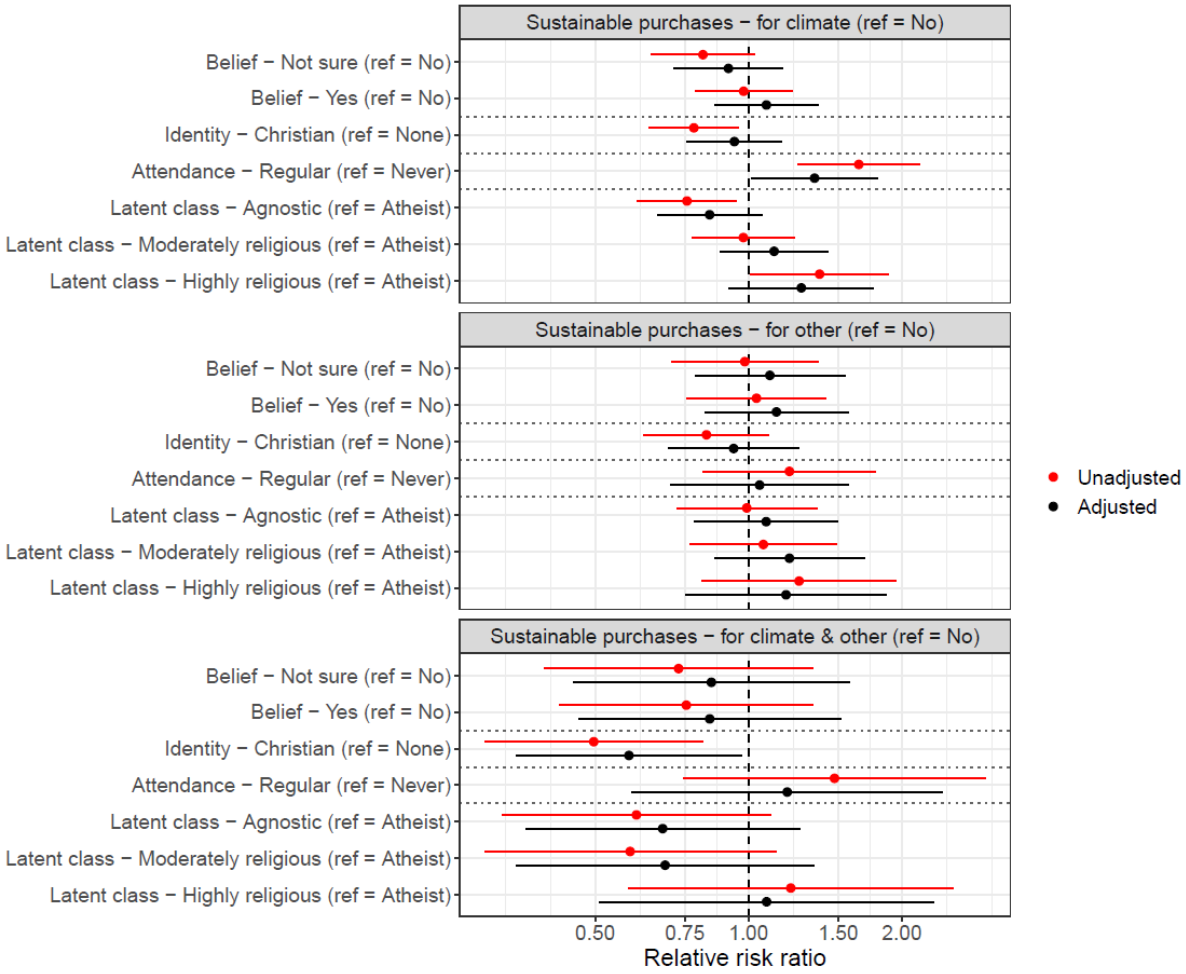

Figure S58: Predicted probabilities of the mothers multinomial regression models with 'chosen sustainably sourced items' as the outcome for four religious exposures (belief, identity, attendance and latent classes).

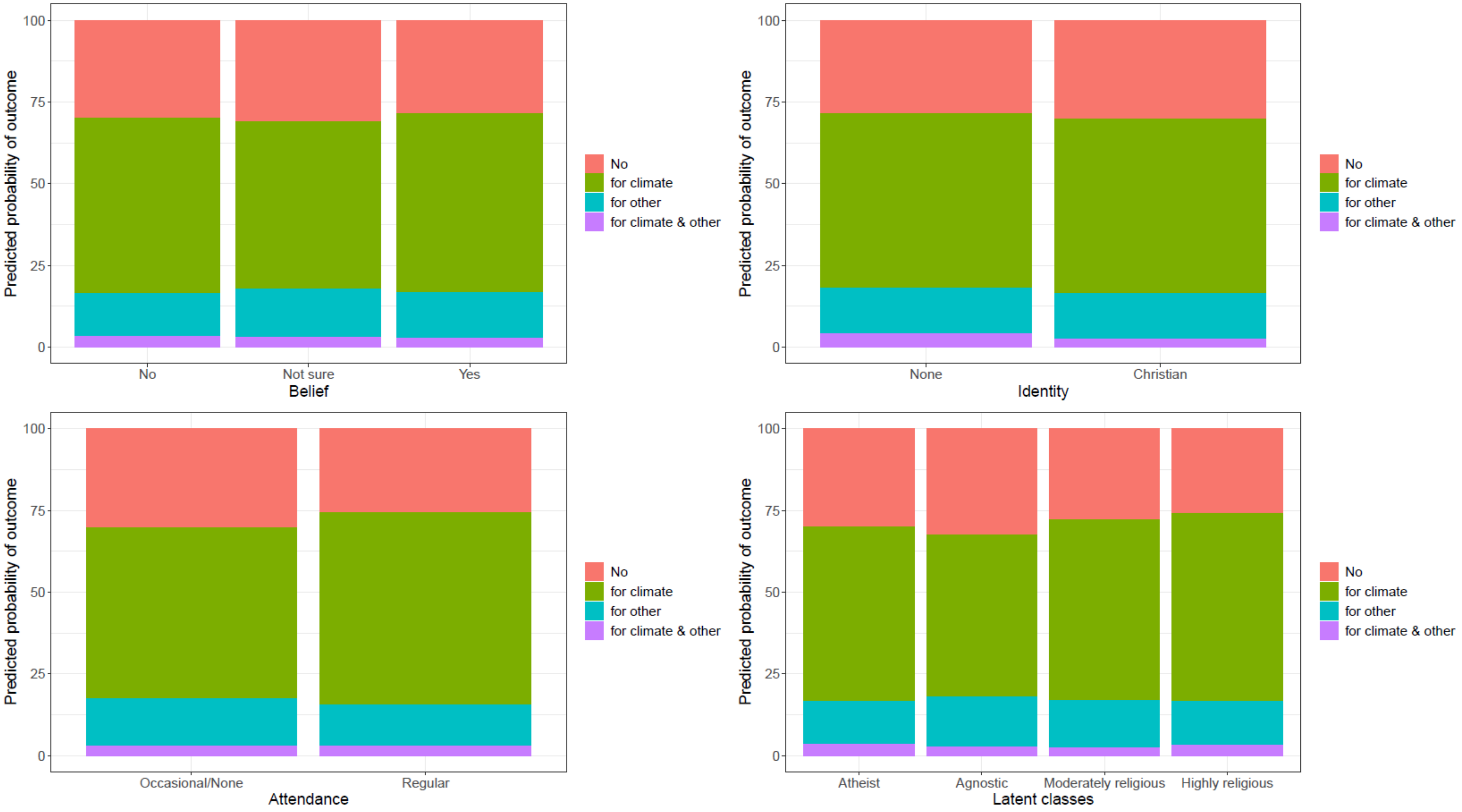

Figure S59: Predicted probabilities of the mothers multinomial regression models with ‘chosen sustainably sourced items’ as the outcome and the religious identity (with the Christian denominations separated) as the exposure.

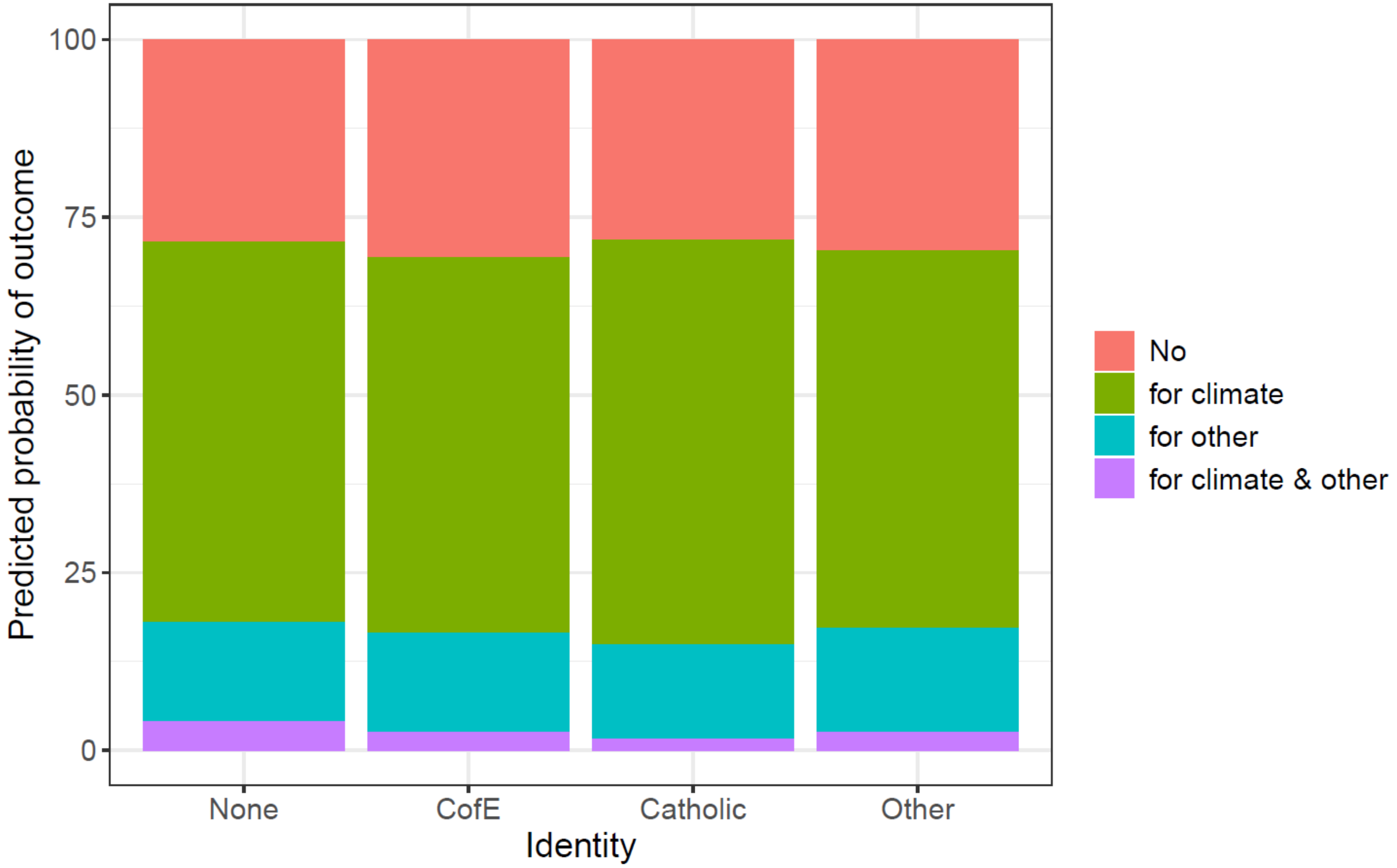

Figure S60: Results of the mothers multinomial regression models with 'improved home insulation' as the outcome for four religious exposures (belief [*n* = 2,559], identity [*n* = 2,532], attendance [*n* = 2,532], and latent classes [*n* = 2,565]; models are separated by dashed horizontal lines). See table S19 for full results.

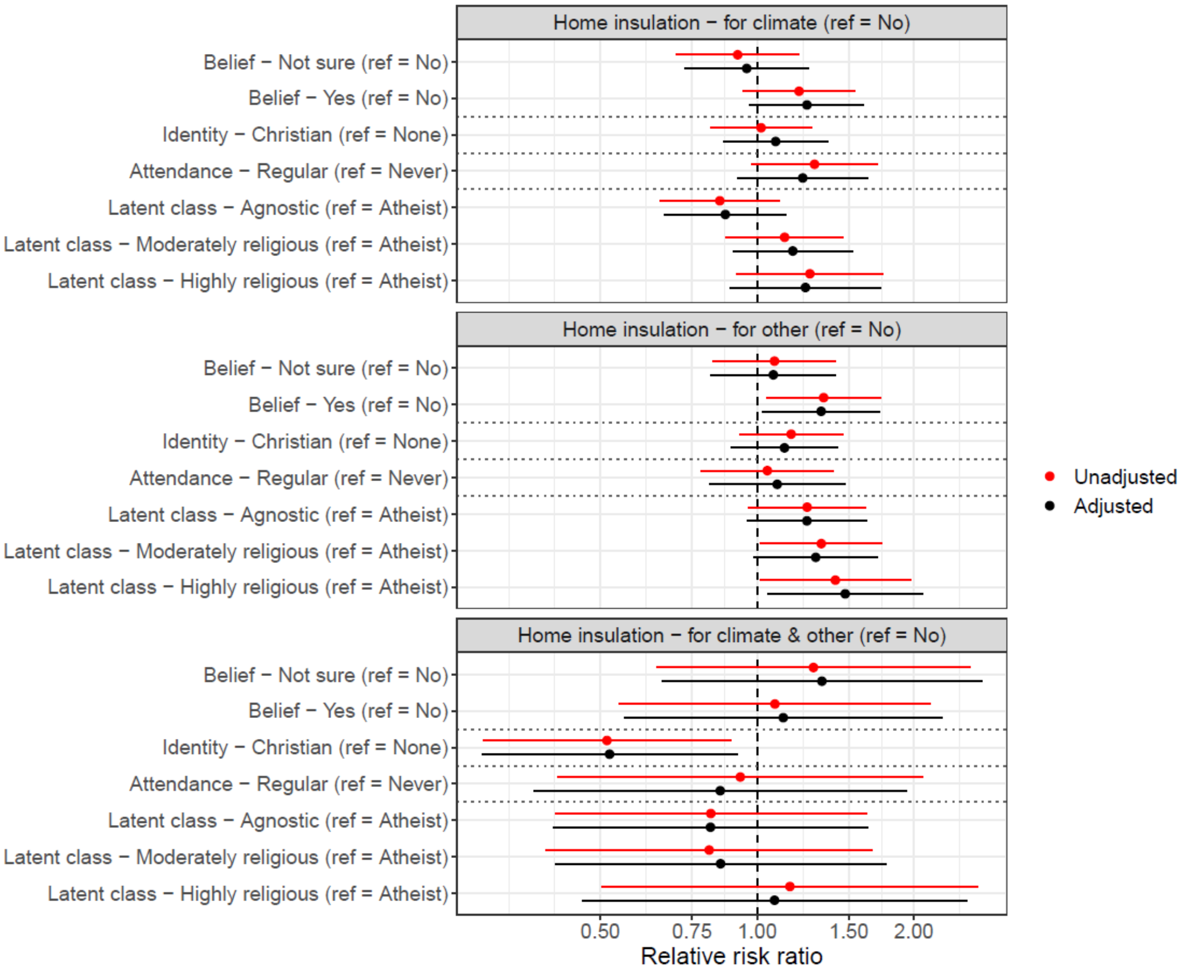

Figure S61: Predicted probabilities of the mothers multinomial regression models with 'improved home insulation' as the outcome for four religious exposures (belief, identity, attendance and latent classes).

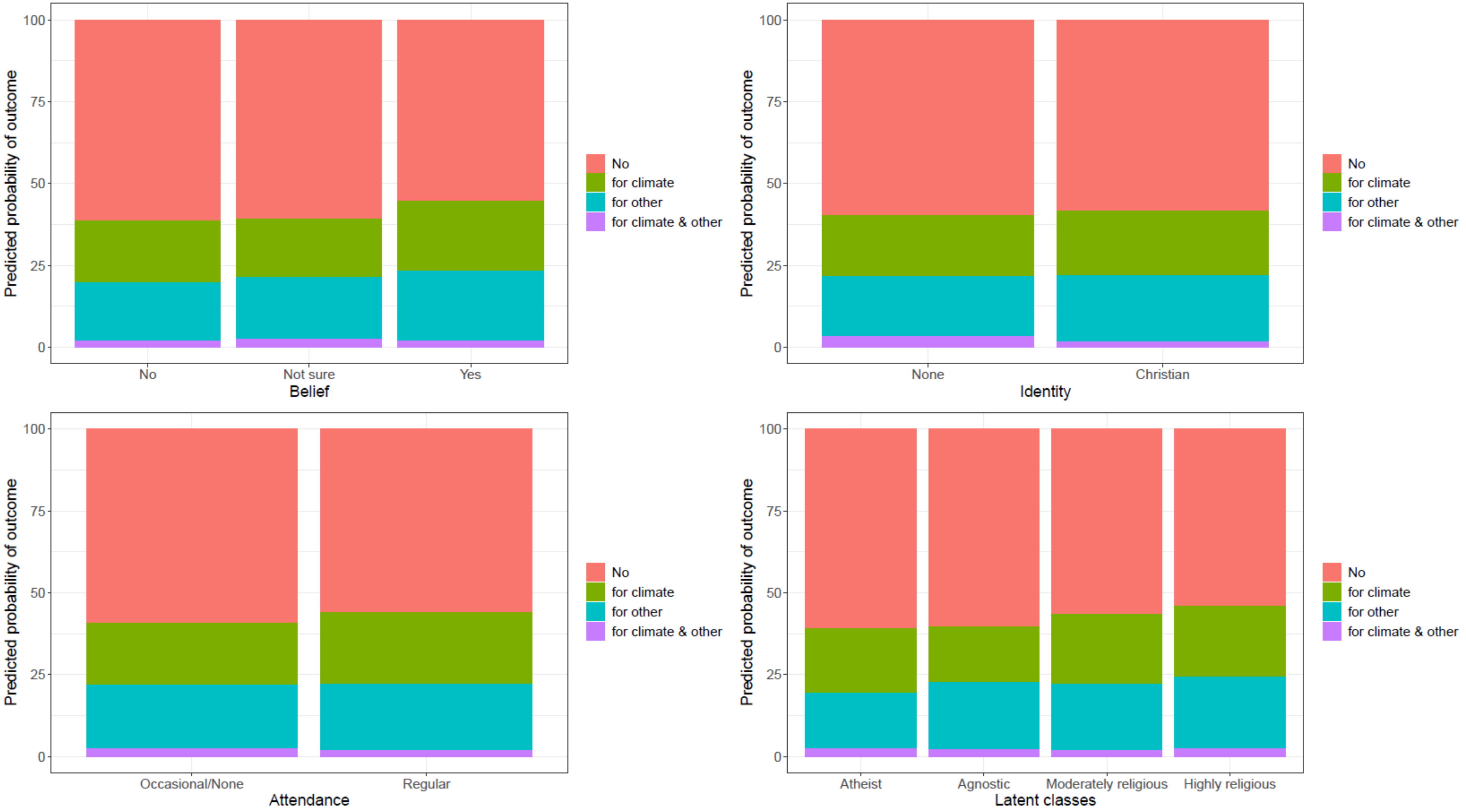

Figure S62: Predicted probabilities of the mothers multinomial regression models with 'improved home insulation' as the outcome and the religious identity (with the Christian denominations separated) as the exposure.

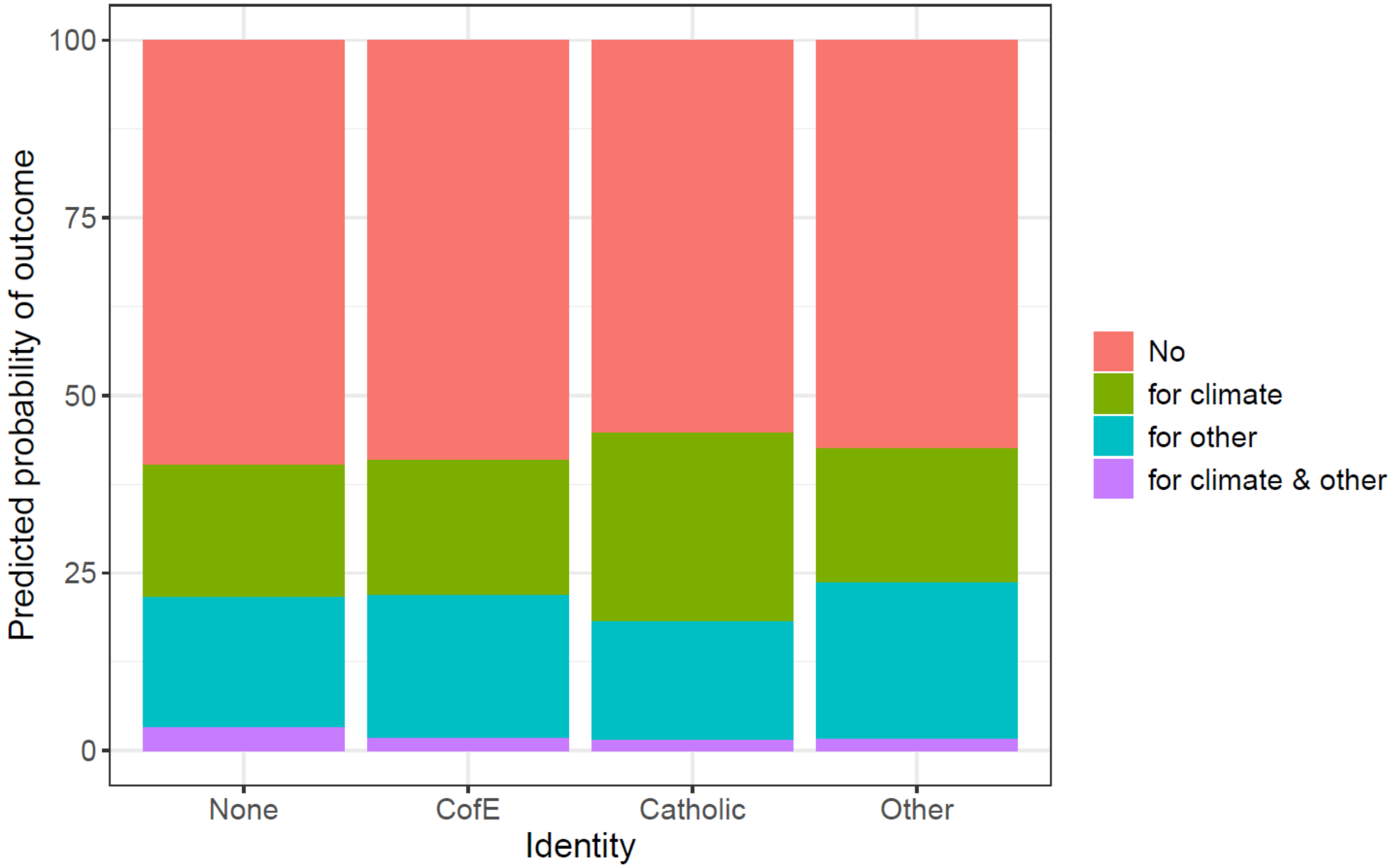

Figure S63: Results of the mothers multinomial regression models with ‘installed solar panels’ as the outcome for four religious exposures (belief [*n* = 2,549], identity [*n* = 2,522], attendance [*n* = 2,522], and latent classes [*n* = 2,555]; models are separated by dashed horizontal lines). See table S19 for full results.

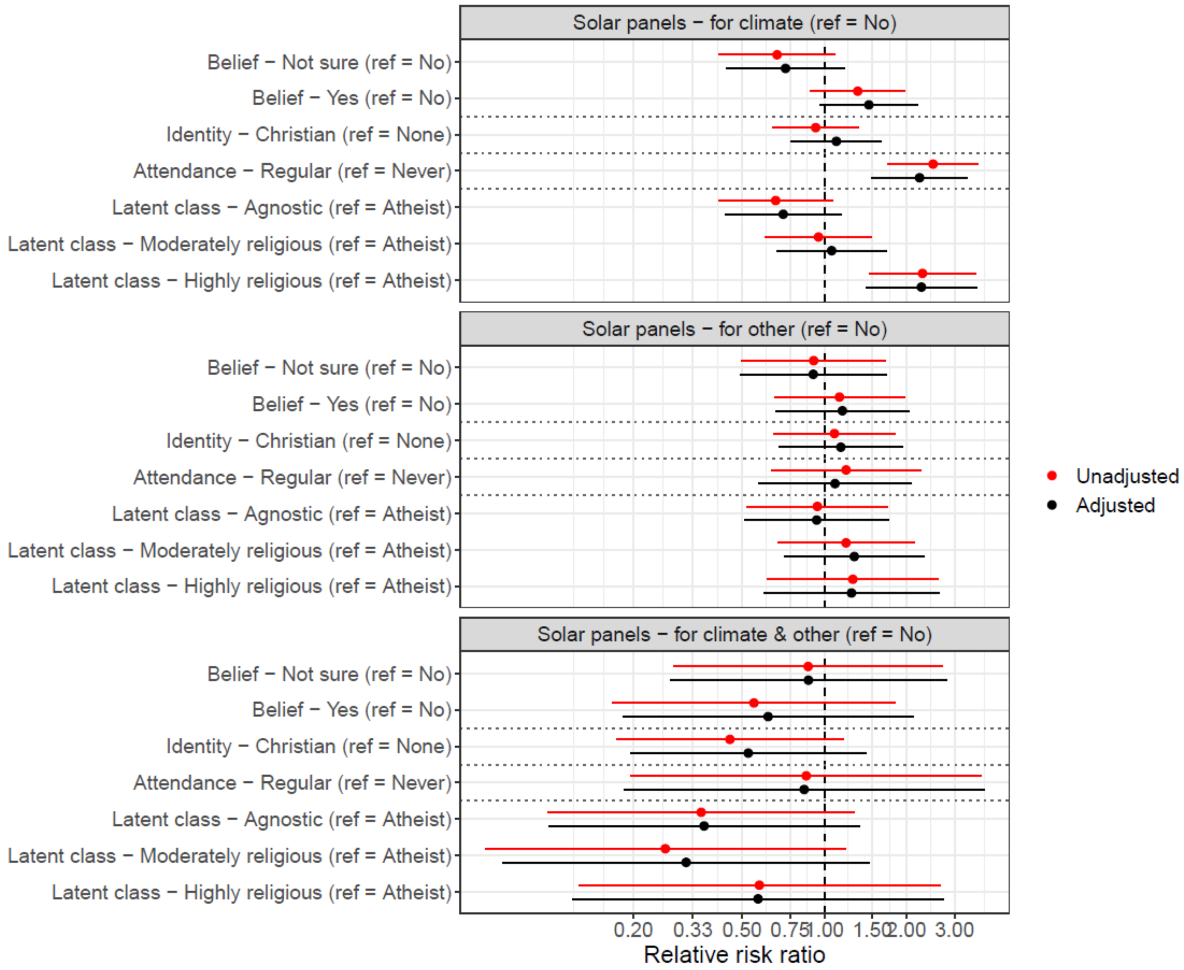

Figure S64: Predicted probabilities of the mothers multinomial regression models with 'installed solar panels' as the outcome for four religious exposures (belief, identity, attendance and latent classes).

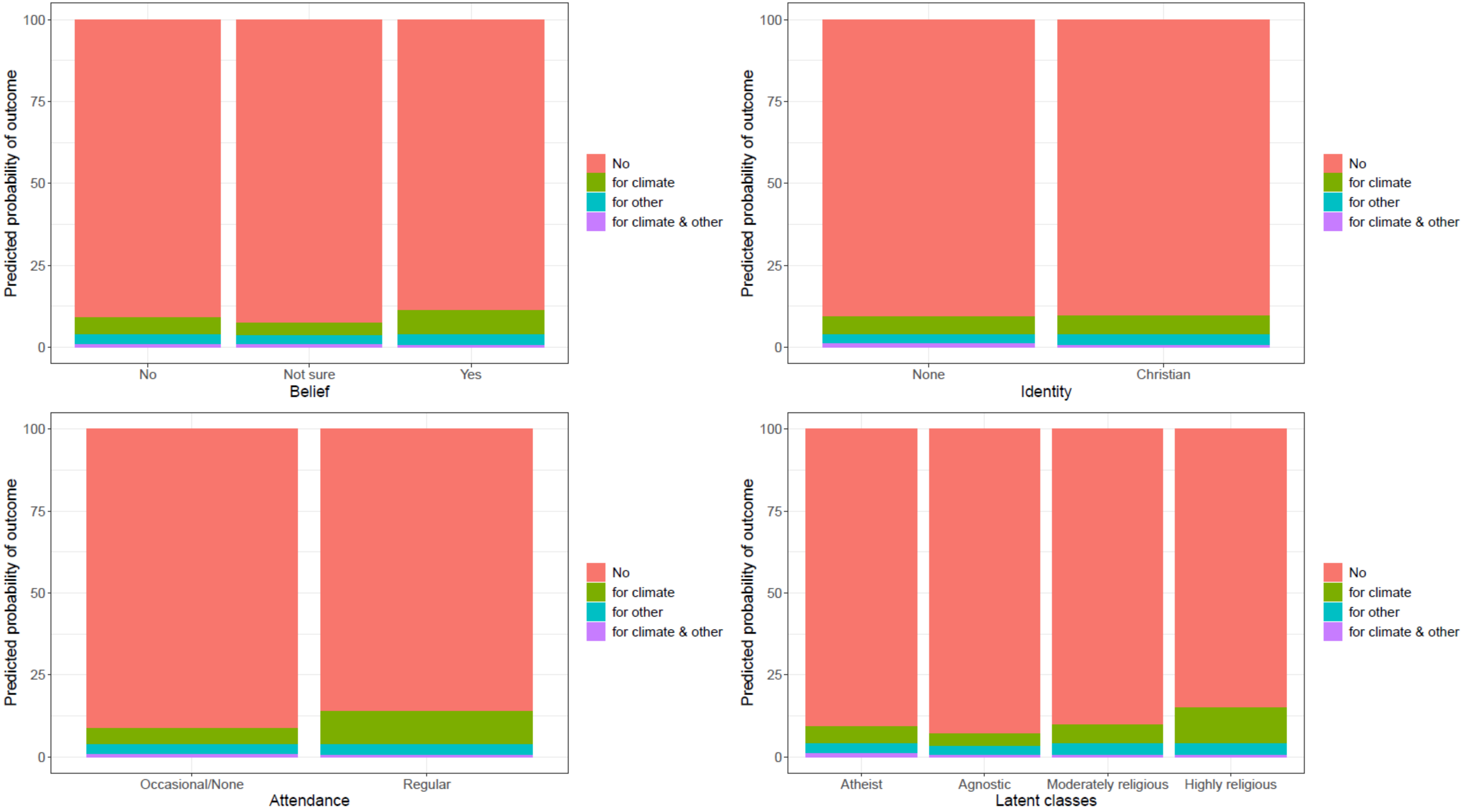

Figure S65: Predicted probabilities of the mothers multinomial regression models with ‘installed solar panels’ as the outcome and the religious identity (with the Christian denominations separated) as the exposure.

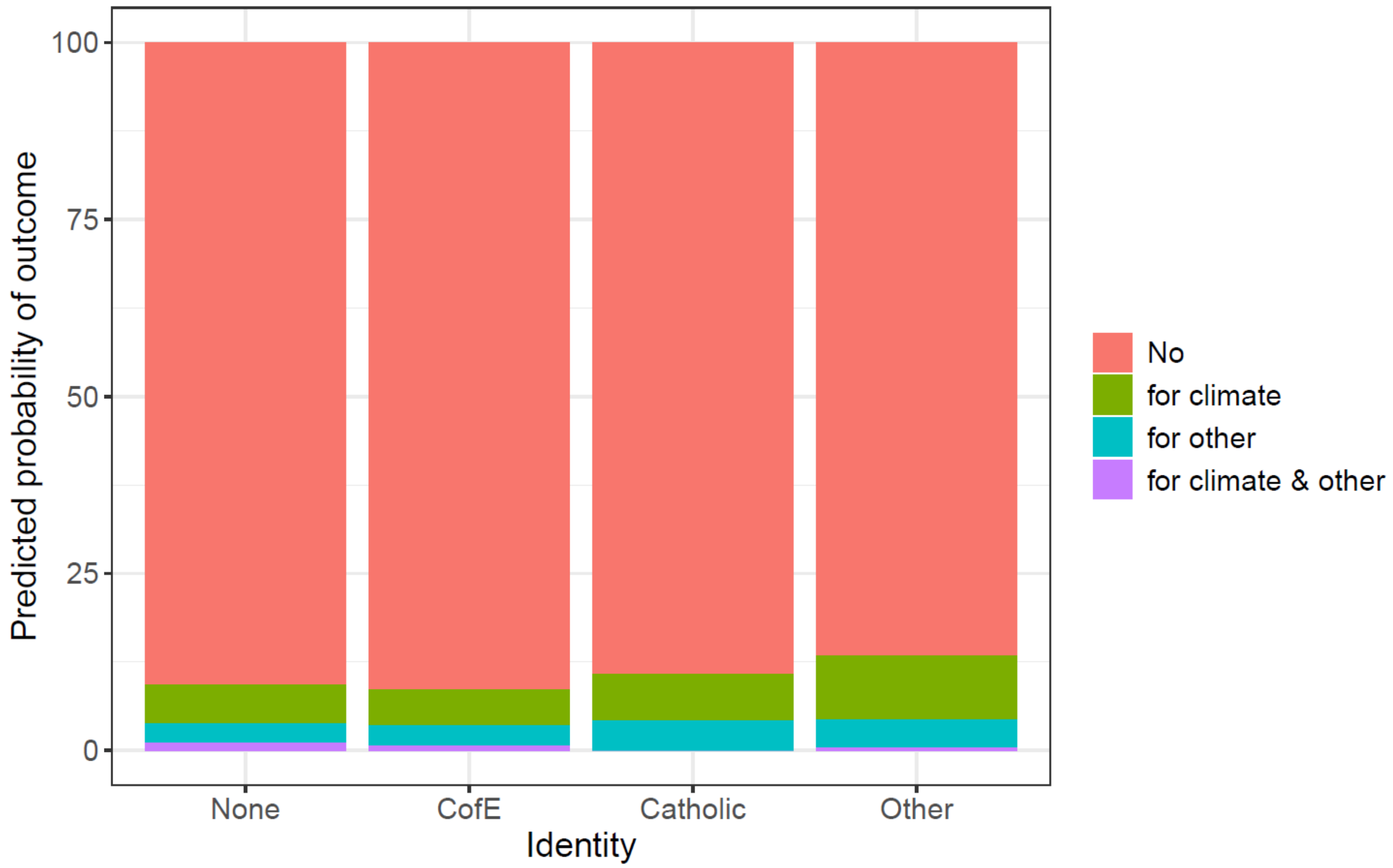

Figure S66: Results of the mothers multinomial regression models with 'started growing vegetables' as the outcome for four religious exposures (belief [*n* = 2,558], identity [*n* = 2,531], attendance [*n* = 2,531], and latent classes [*n* = 2,564]; models are separated by dashed horizontal lines). See table S19 for full results.

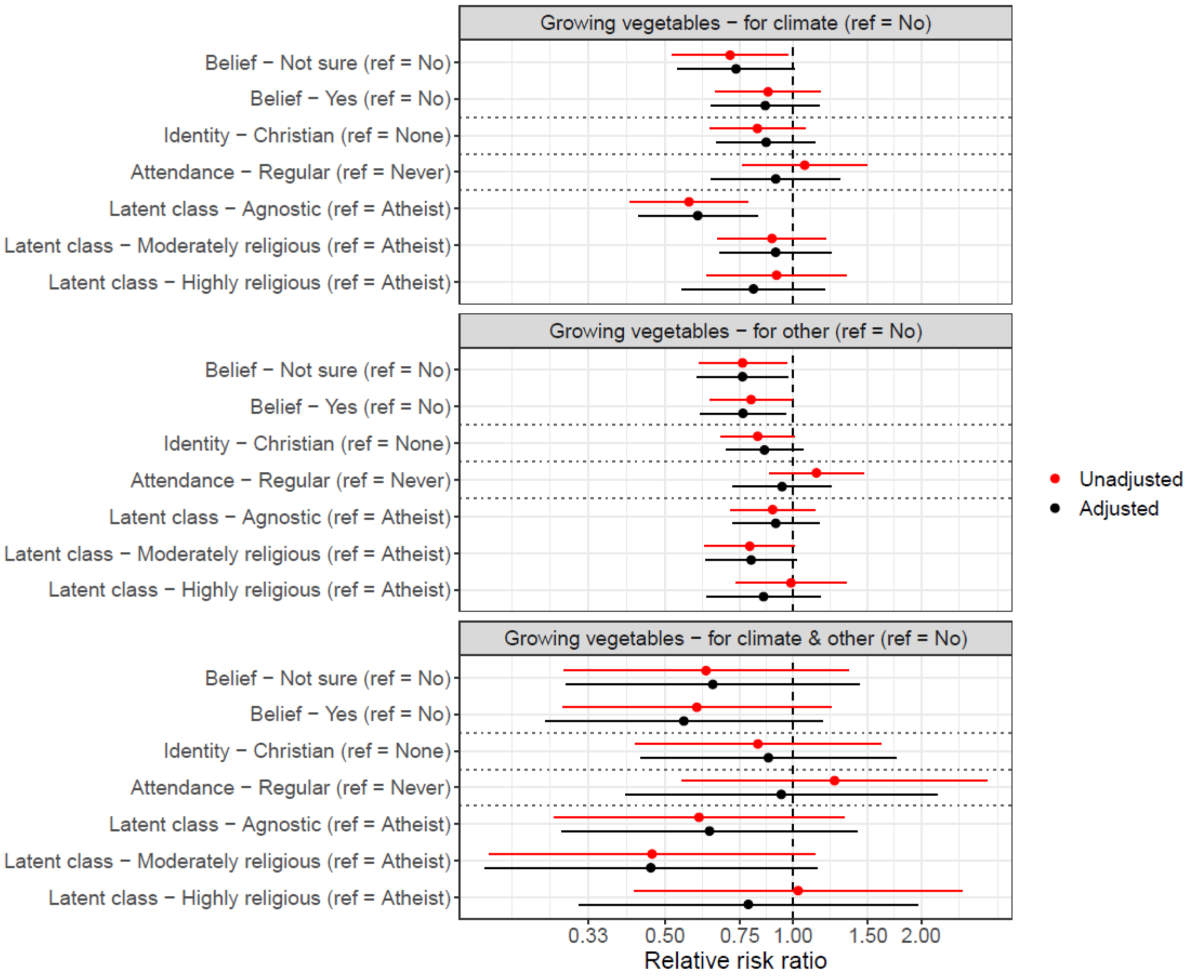

Figure S67: Predicted probabilities of the mothers multinomial regression models with ‘started growing vegetables’ as the outcome for four religious exposures (belief, identity, attendance and latent classes).

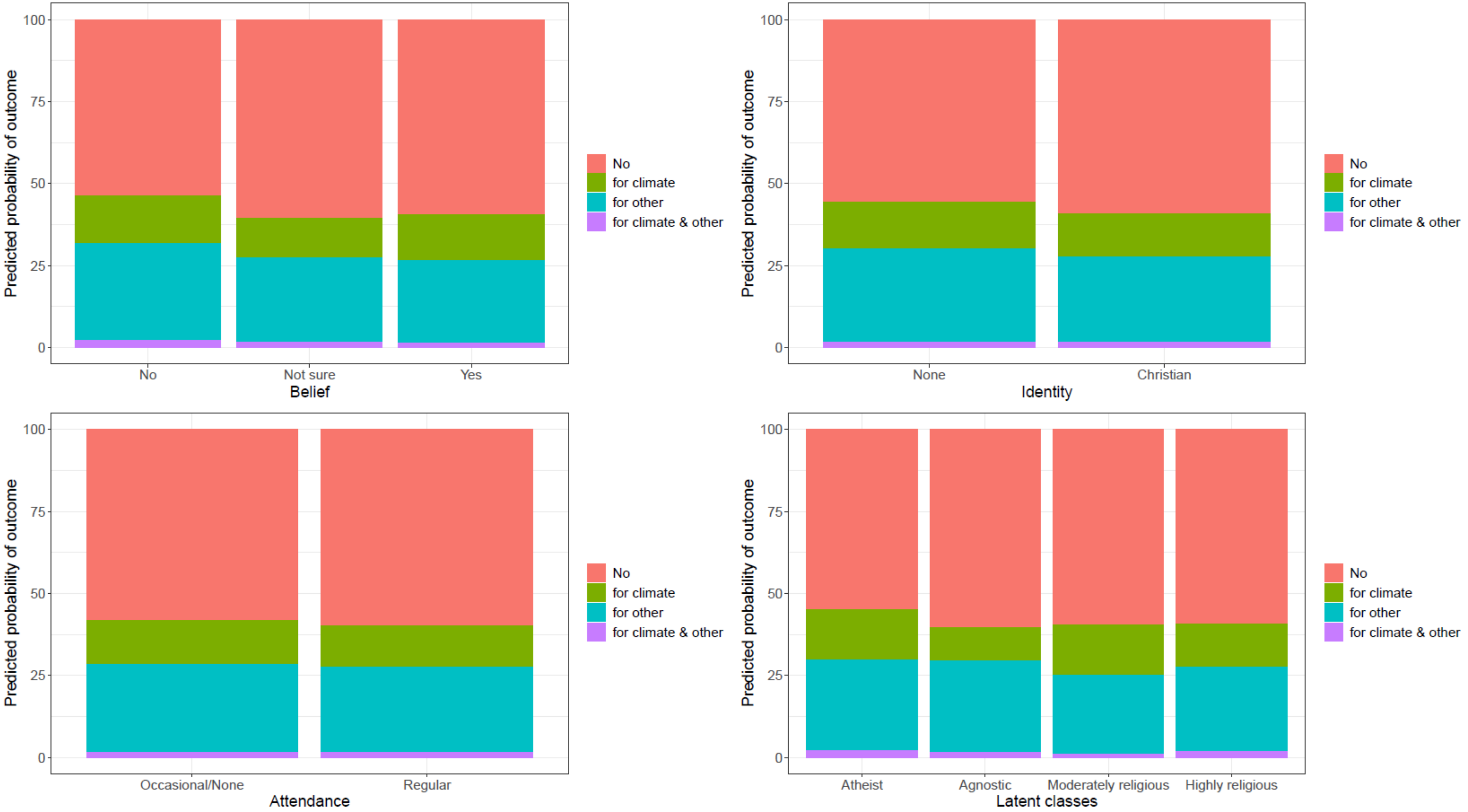

Figure S68: Predicted probabilities of the mothers multinomial regression models with 'started growing vegetables' as the outcome and the religious identity (with the Christian denominations separated) as the exposure.

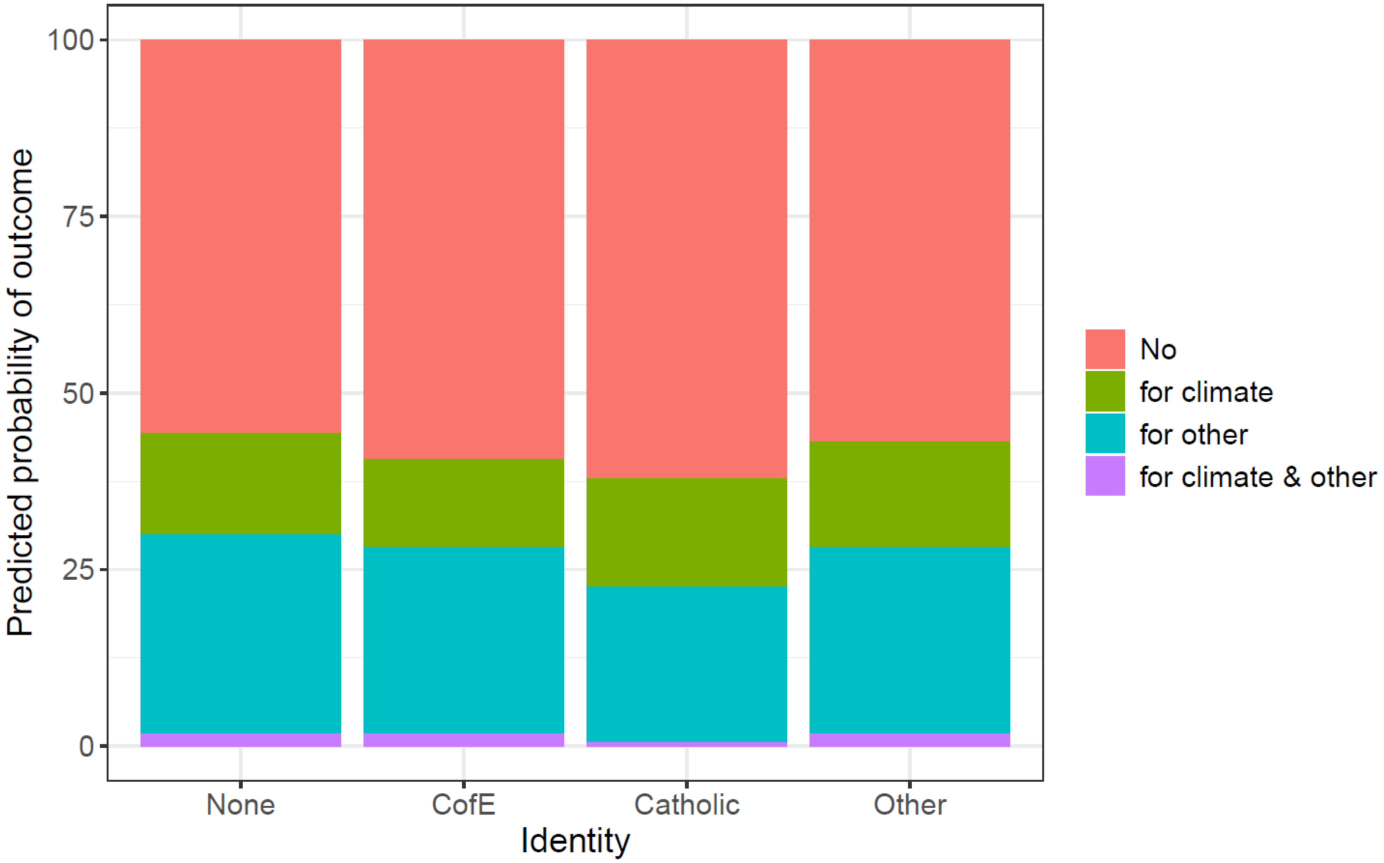

Figure S69: Results of the mothers multinomial regression models with ‘planted trees’ as the outcome for four religious exposures (belief [*n* = 2,552], identity [*n* = 2,526], attendance [*n* = 2,525], and latent classes [*n* = 2,558]; models are separated by dashed horizontal lines). See table S19 for full results.

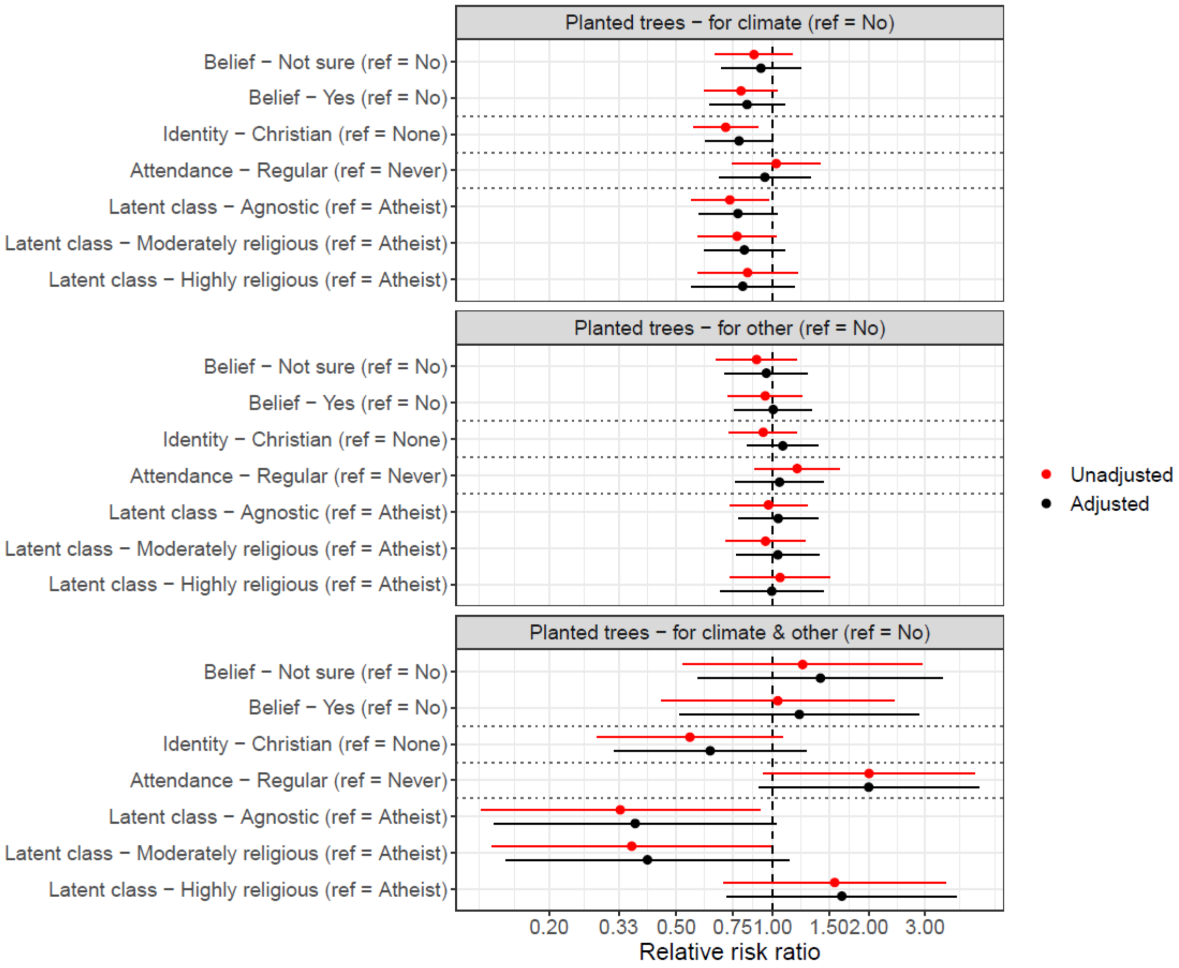

Figure S70: Predicted probabilities of the mothers multinomial regression models with ‘planted trees’ as the outcome for four religious exposures (belief, identity, attendance and latent classes).

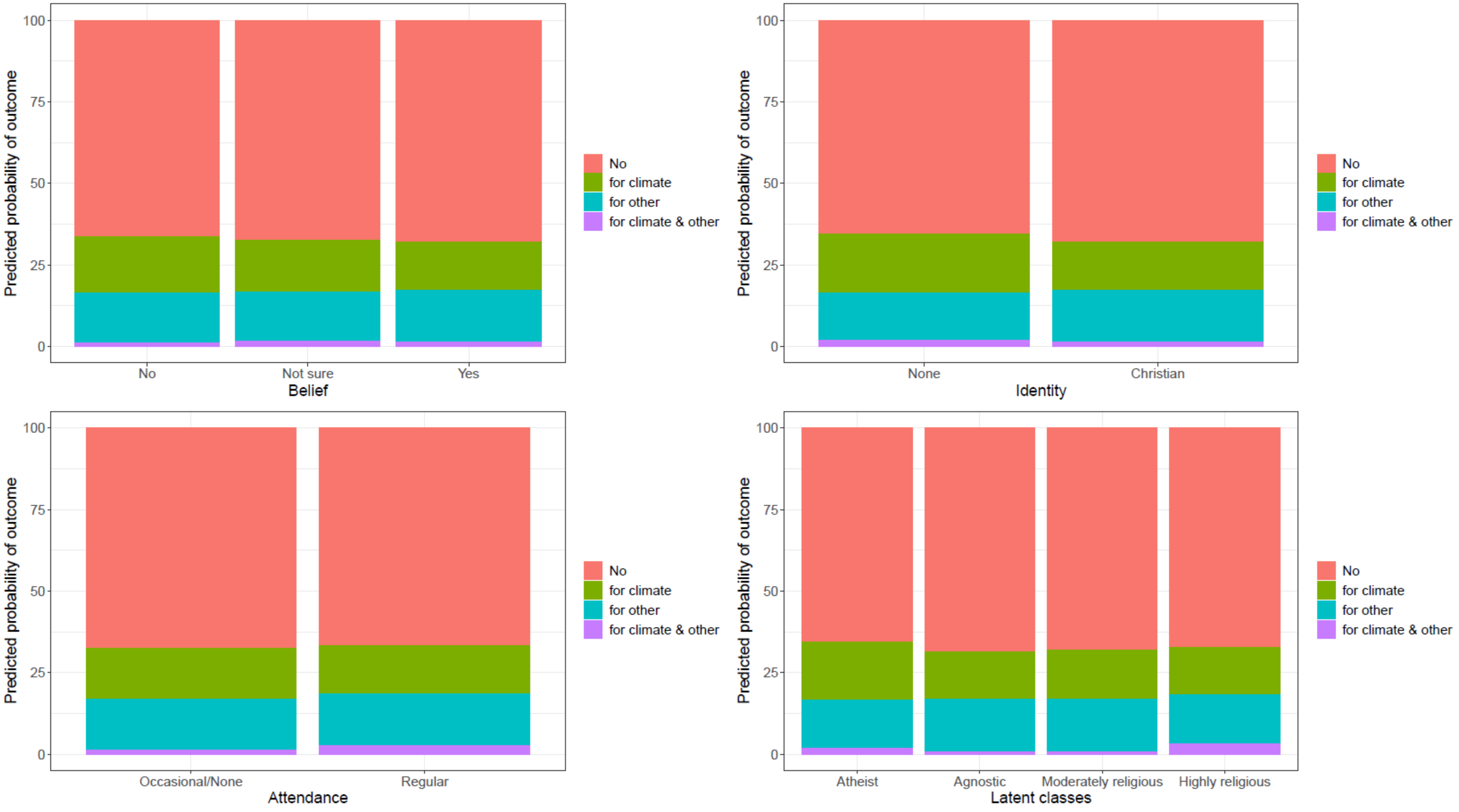

Figure S71: Predicted probabilities of the mothers multinomial regression models with 'planted trees' as the outcome and the religious identity (with the Christian denominations separated) as the exposure.

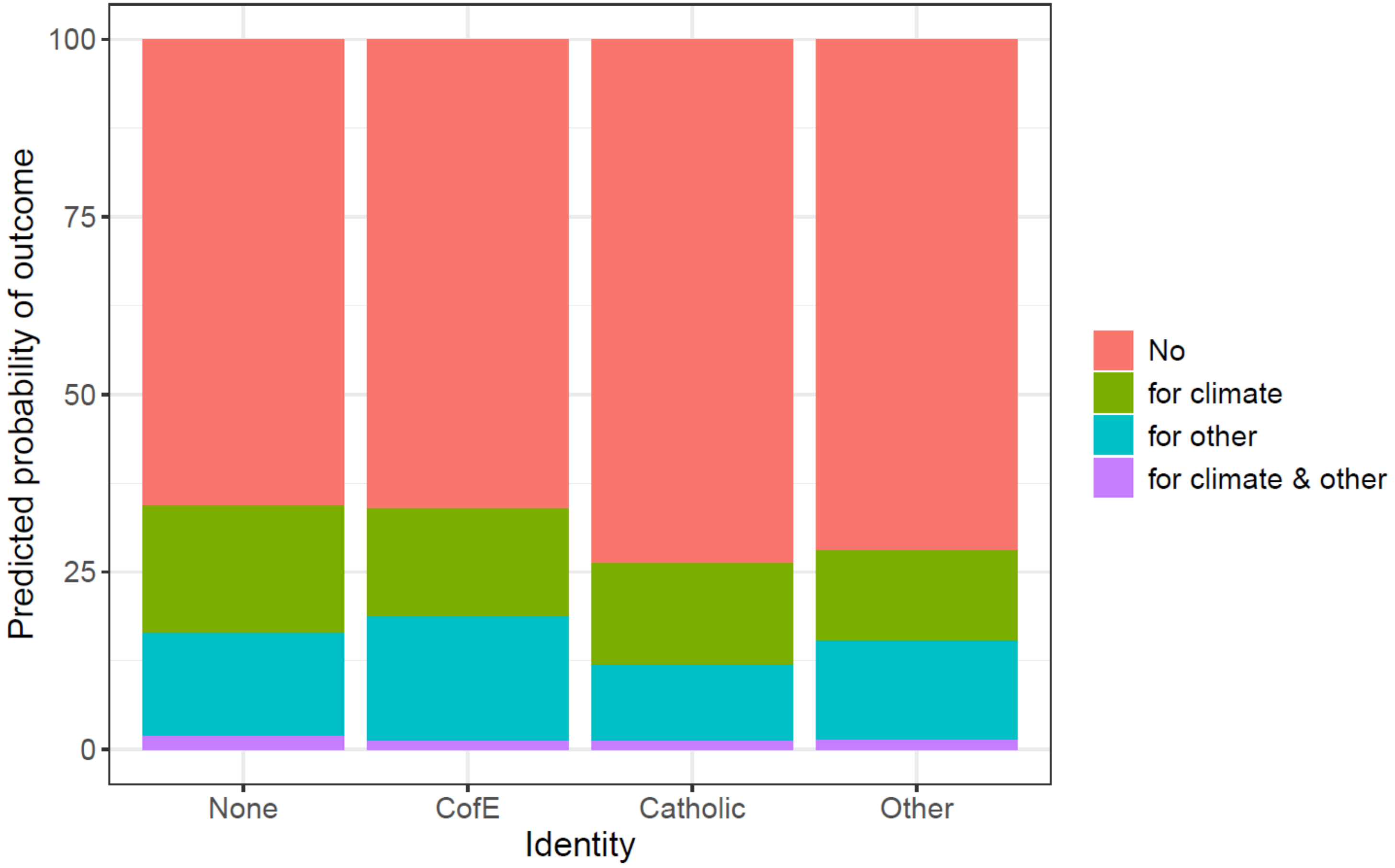

Figure S72: Results of the mothers multinomial regression models with 'avoided organisations that support fossil fuels' as the outcome for four religious exposures (belief [*n* = 2,534], identity [*n* = 2,508], attendance [*n* = 2,507], and latent classes [*n* = 2,540]; models are separated by dashed horizontal lines). See table S19 for full results.

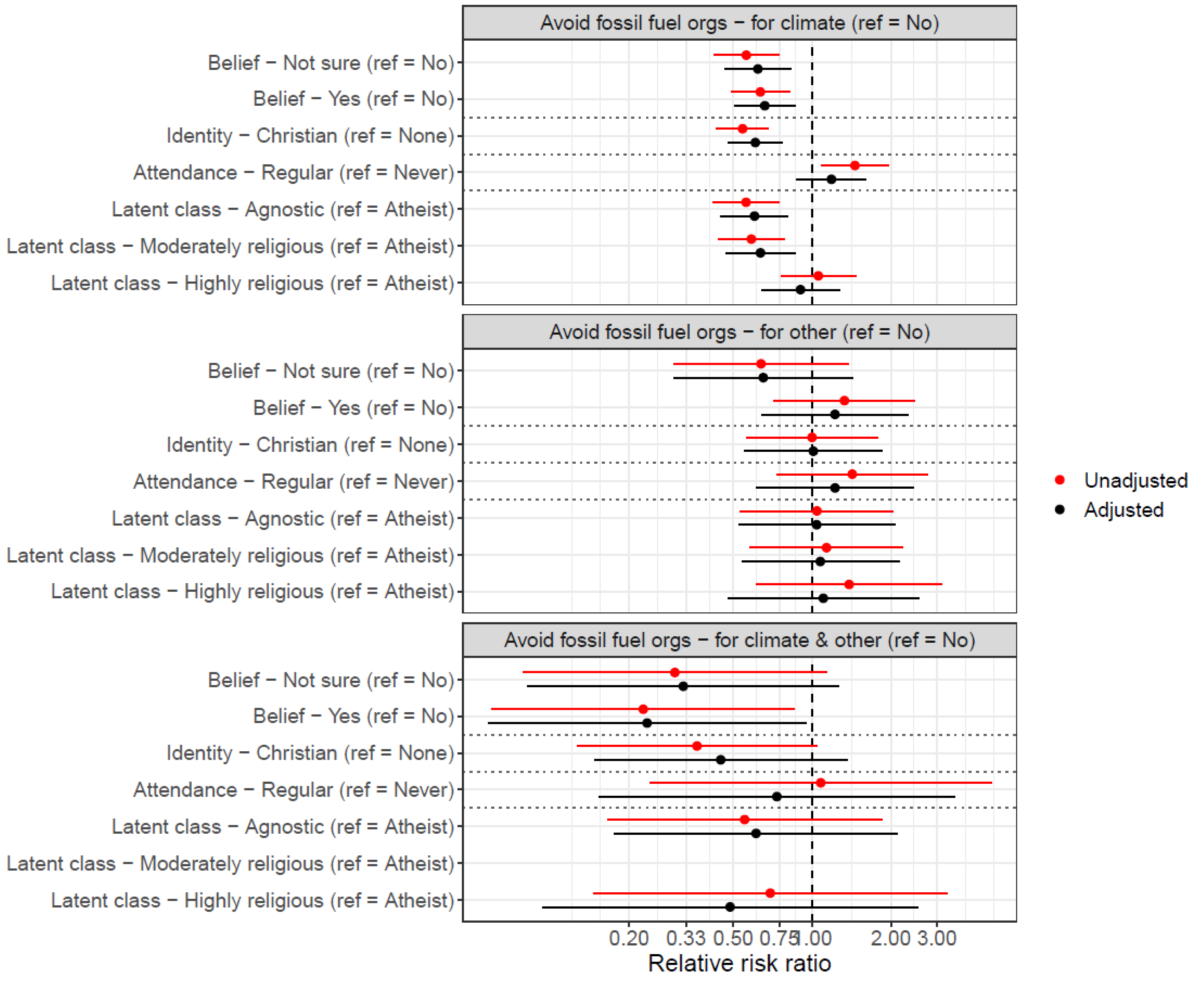

Figure S73: Predicted probabilities of the mothers multinomial regression models with 'avoided organisations that support fossil fuels' as the outcome for four religious exposures (belief, identity, attendance and latent classes).

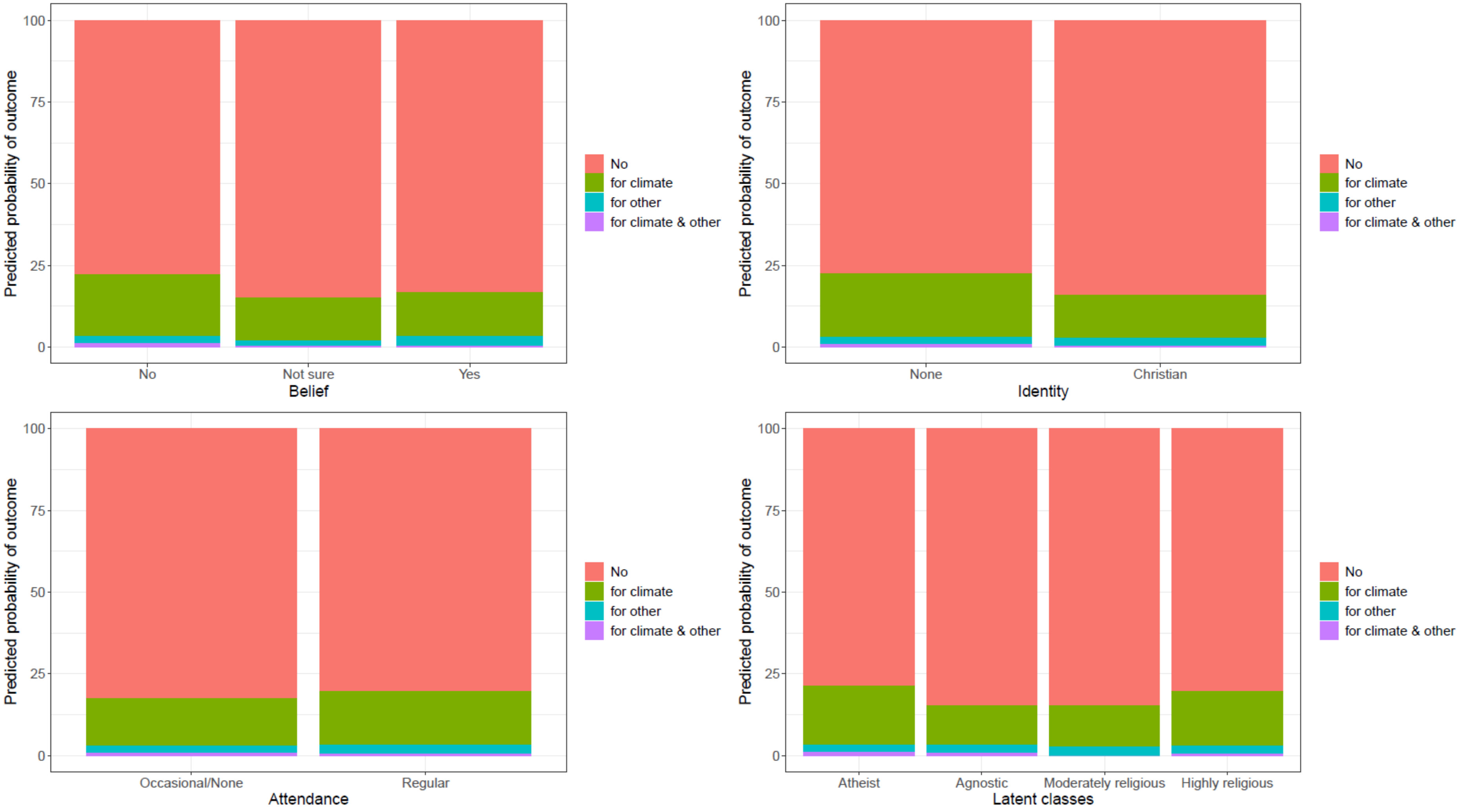

Figure S74: Predicted probabilities of the mothers multinomial regression models with ‘avoided organisations that support fossil fuels’ as the outcome and the religious identity (with the Christian denominations separated) as the exposure.

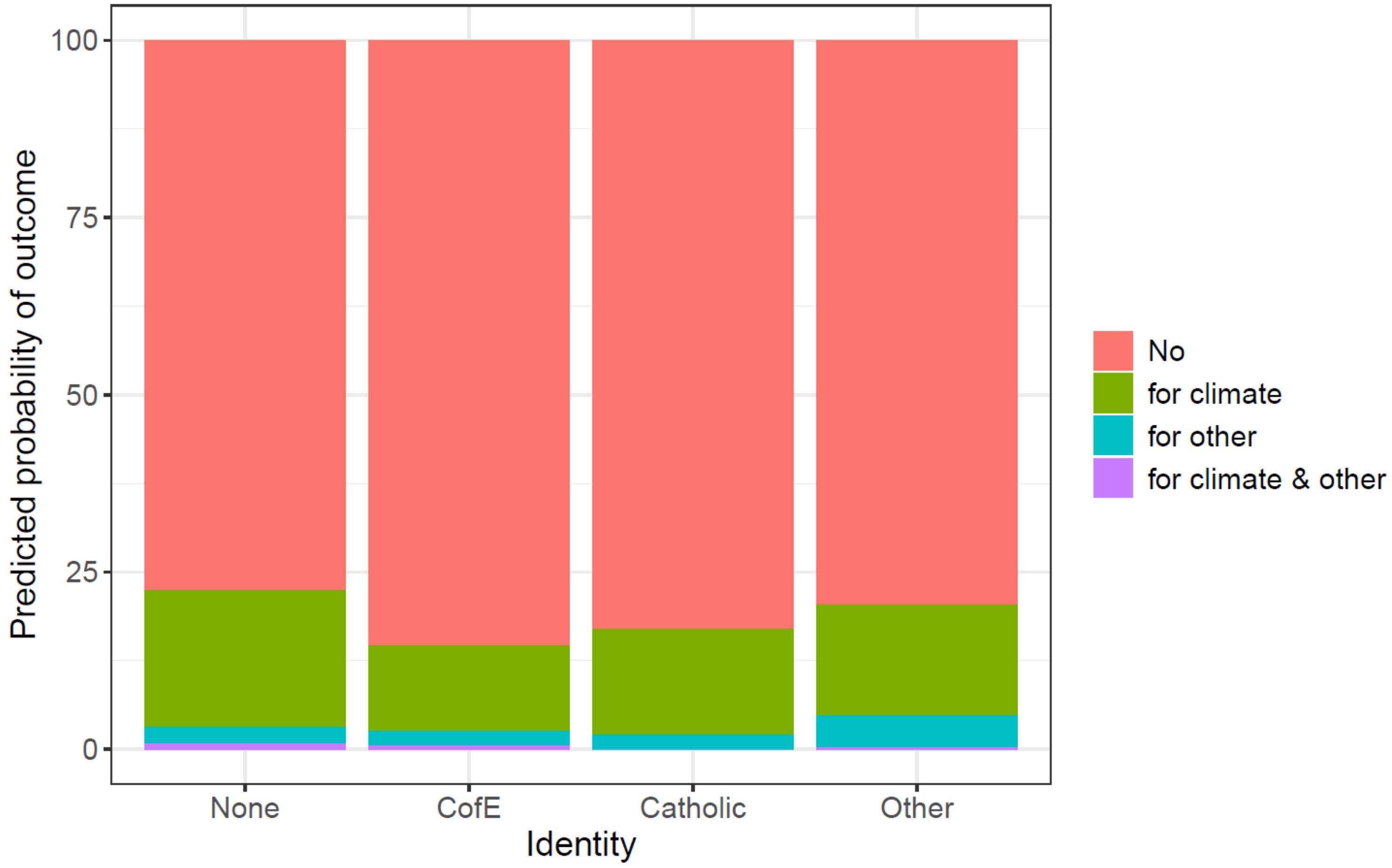

Figure S75: Results of the mothers multinomial regression models with 'taken action to eat less or no meat and/or dairy' as the outcome for four religious exposures (belief [*n* = 2,405], identity [*n* = 2,378], attendance [*n* = 2,382], and latent classes [*n* = 2,411]; models are separated by dashed horizontal lines). See table S19 for full results.

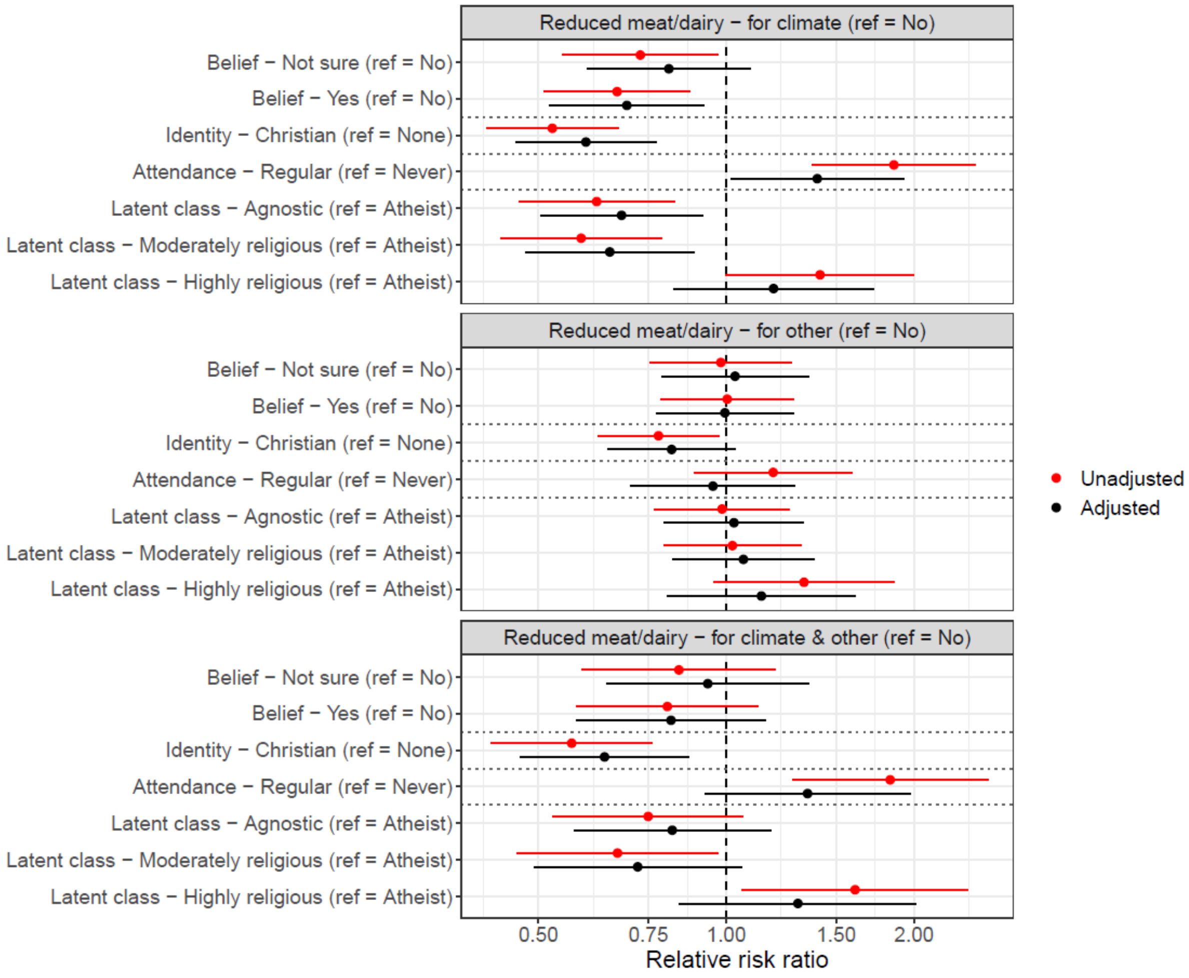

Figure S76: Predicted probabilities of the mothers multinomial regression models with 'taken action to eat less or no meat and/or dairy' as the outcome for four religious exposures (belief, identity, attendance and latent classes).

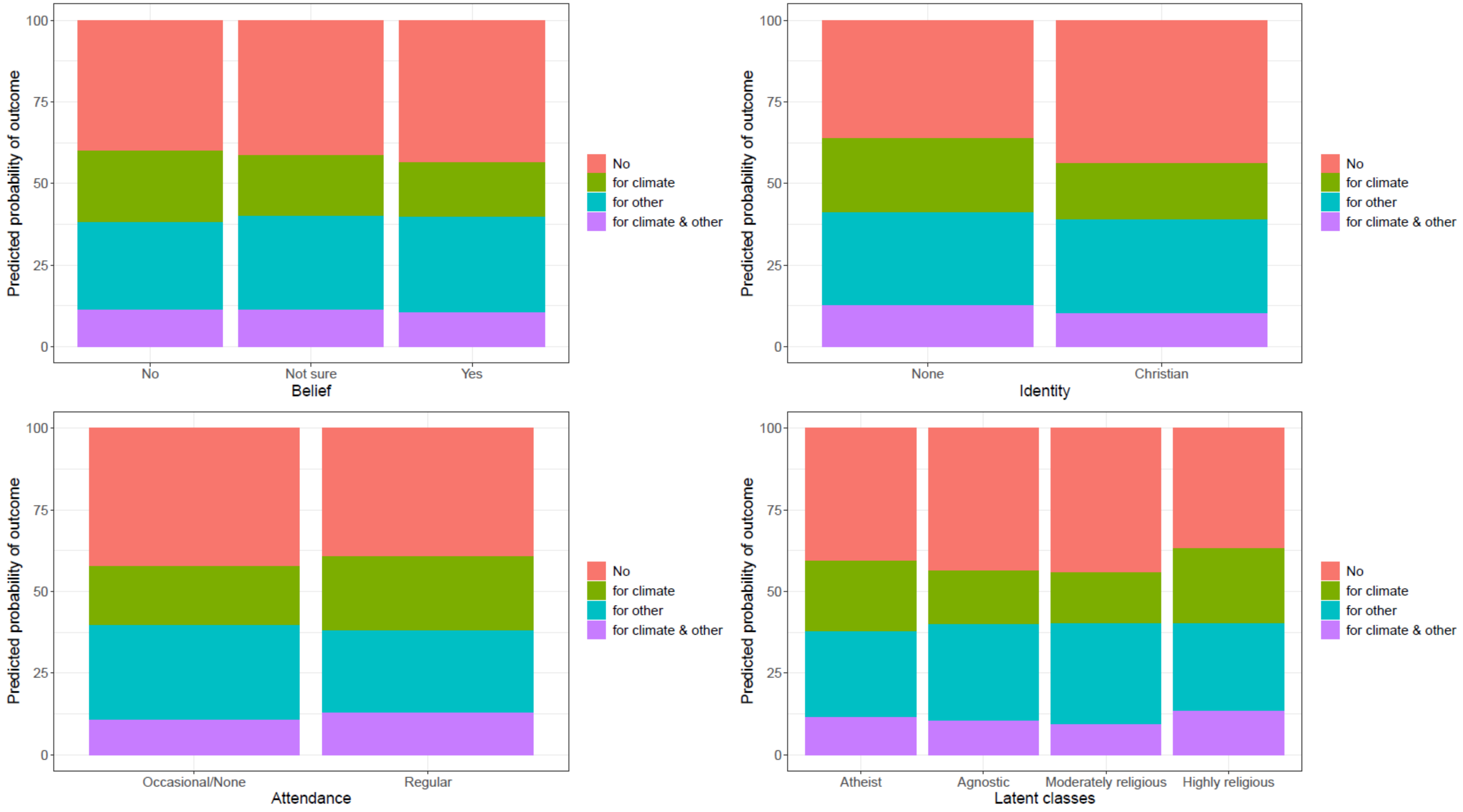

Figure S77: Predicted probabilities of the mothers multinomial regression models with ‘taken action to eat less or no meat and/or dairy’ as the outcome and the religious identity (with the Christian denominations separated) as the exposure.

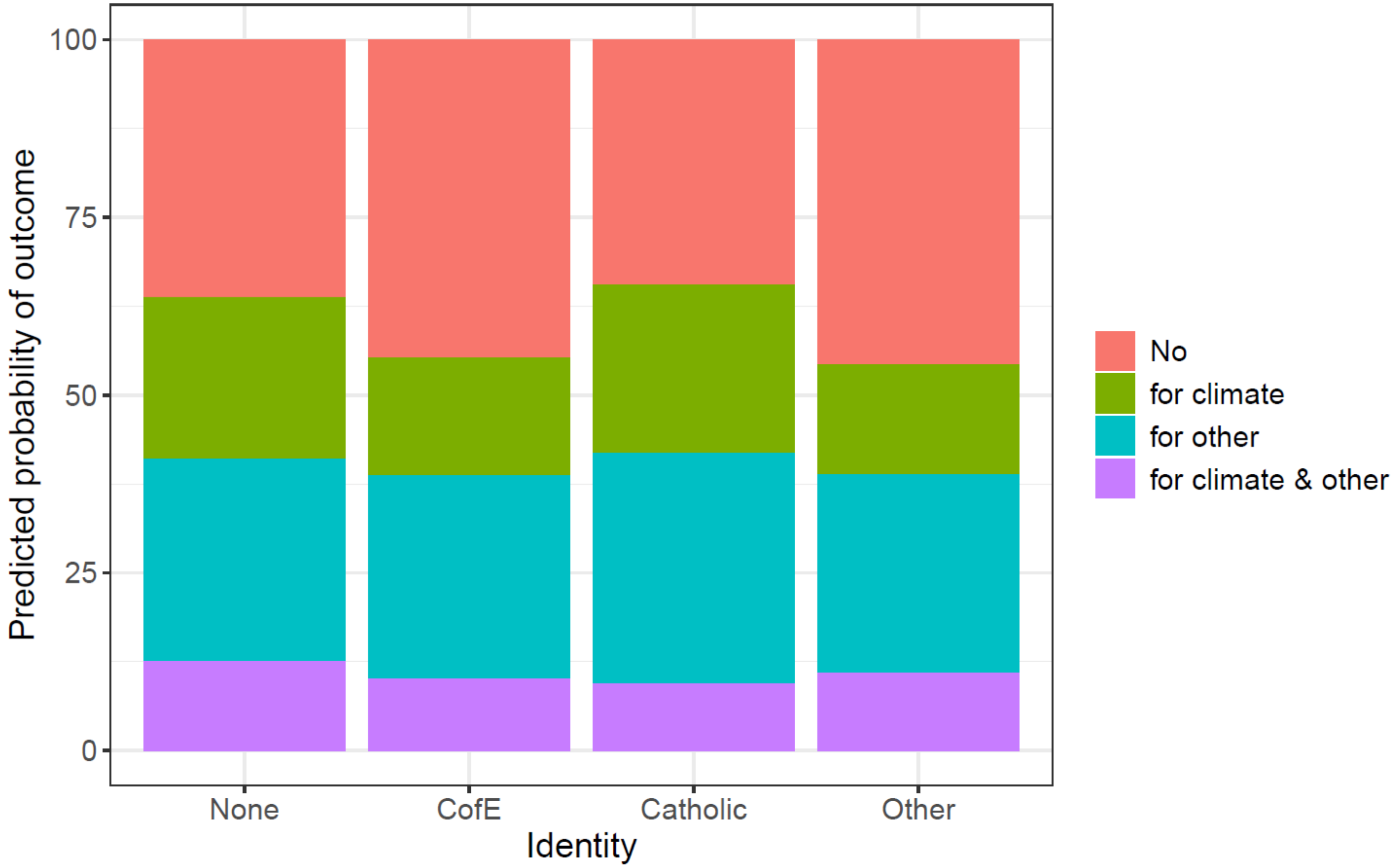

Figure S78: Histogram of total number of pro-environmental actions performed for climate change reasons in G0 partners (max = 16).

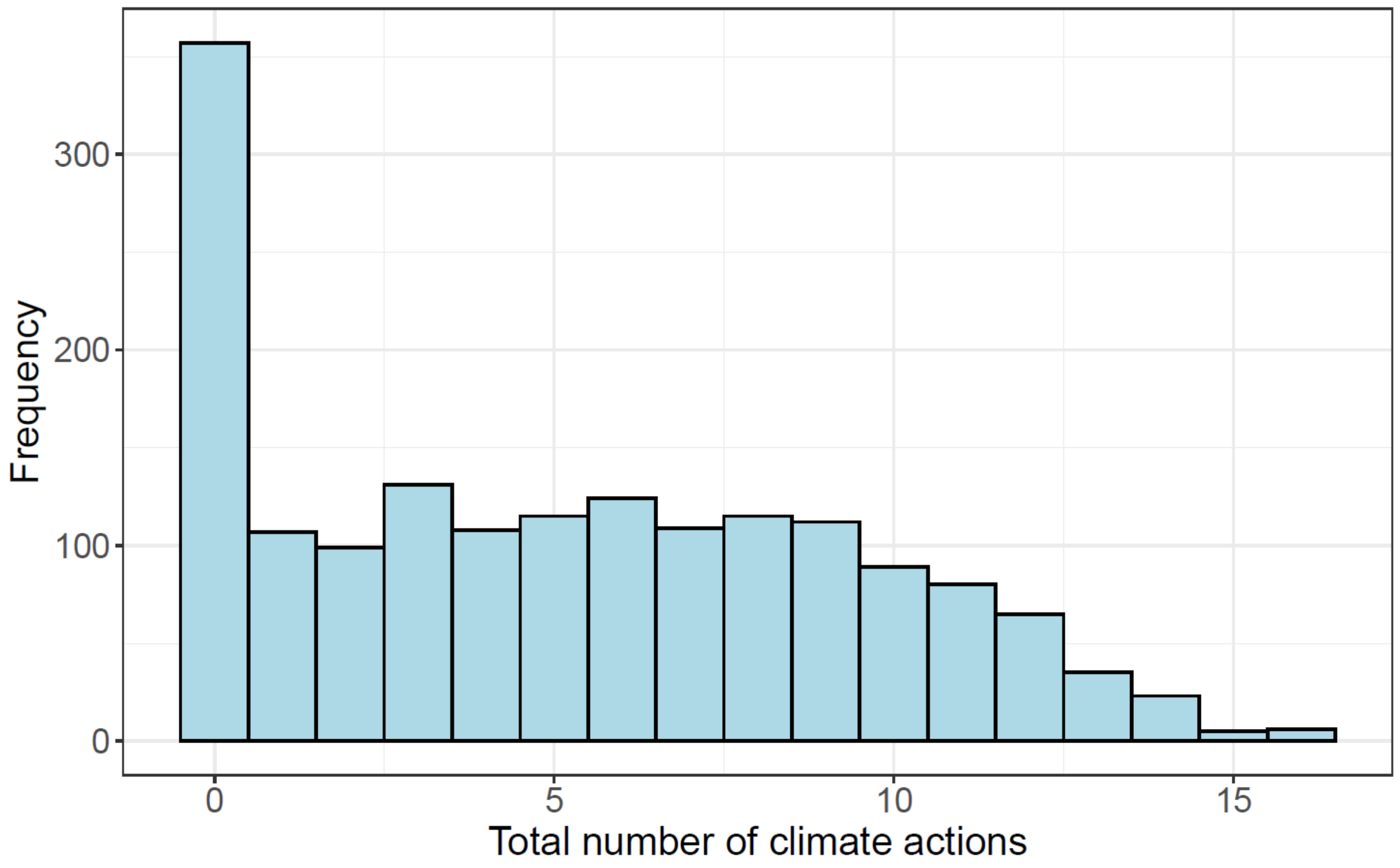

Figure S79: Results of the partners ordinal regression models with 'belief that the climate is changing' as the outcome for four religious exposures (belief [*n* = 1,115], identity [*n* = 1,104], attendance [*n* = 1,117], and latent classes [*n* = 1,125]; models are separated by dashed horizontal lines). Odds ratios above 1 indicate an increased belief in climate change. See table S21 for full results.

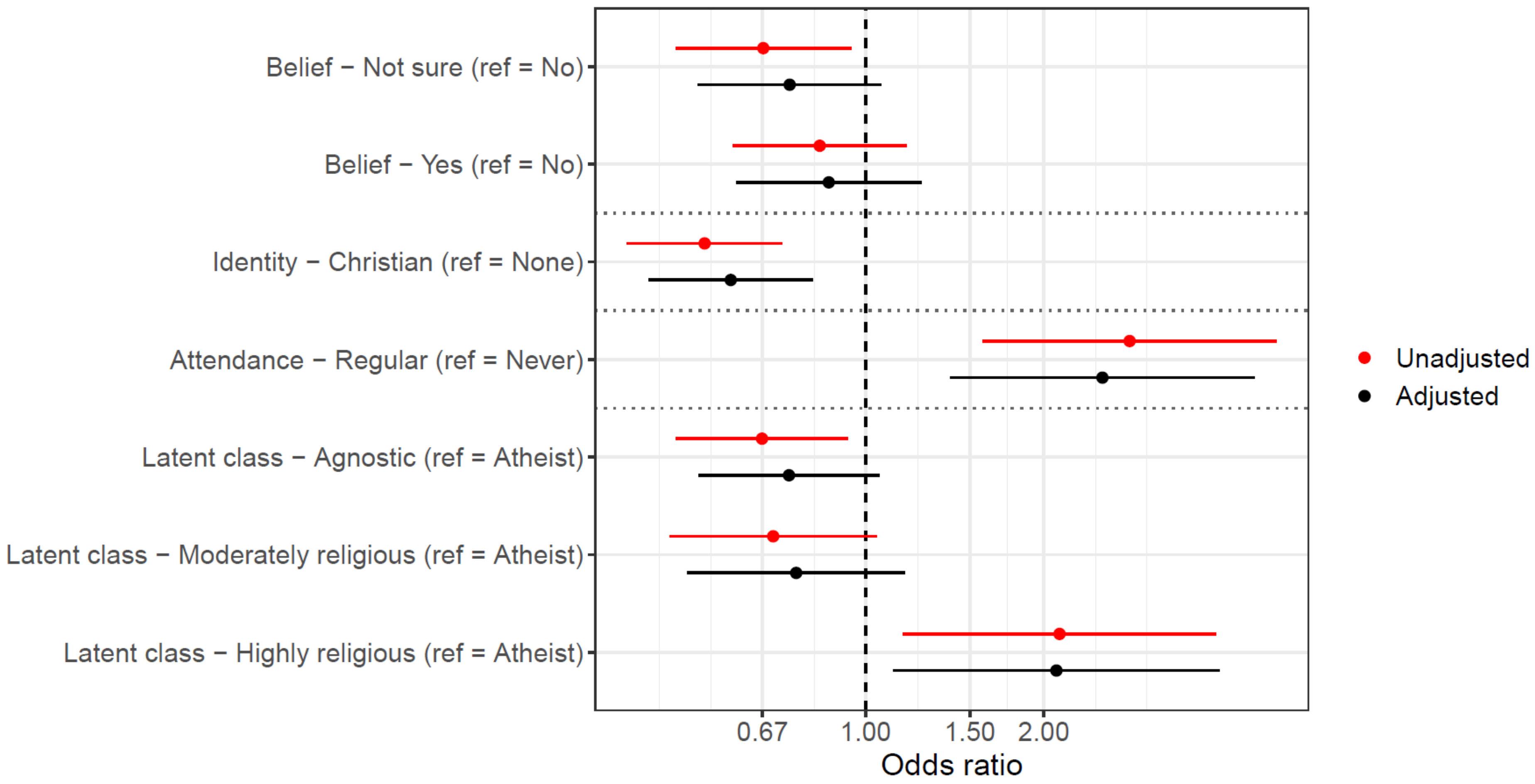

Figure S80: Predicted probabilities of the partners ordinal regression models with ‘belief that the climate is changing’ as the outcome for four religious exposures (belief, identity, attendance and latent classes).

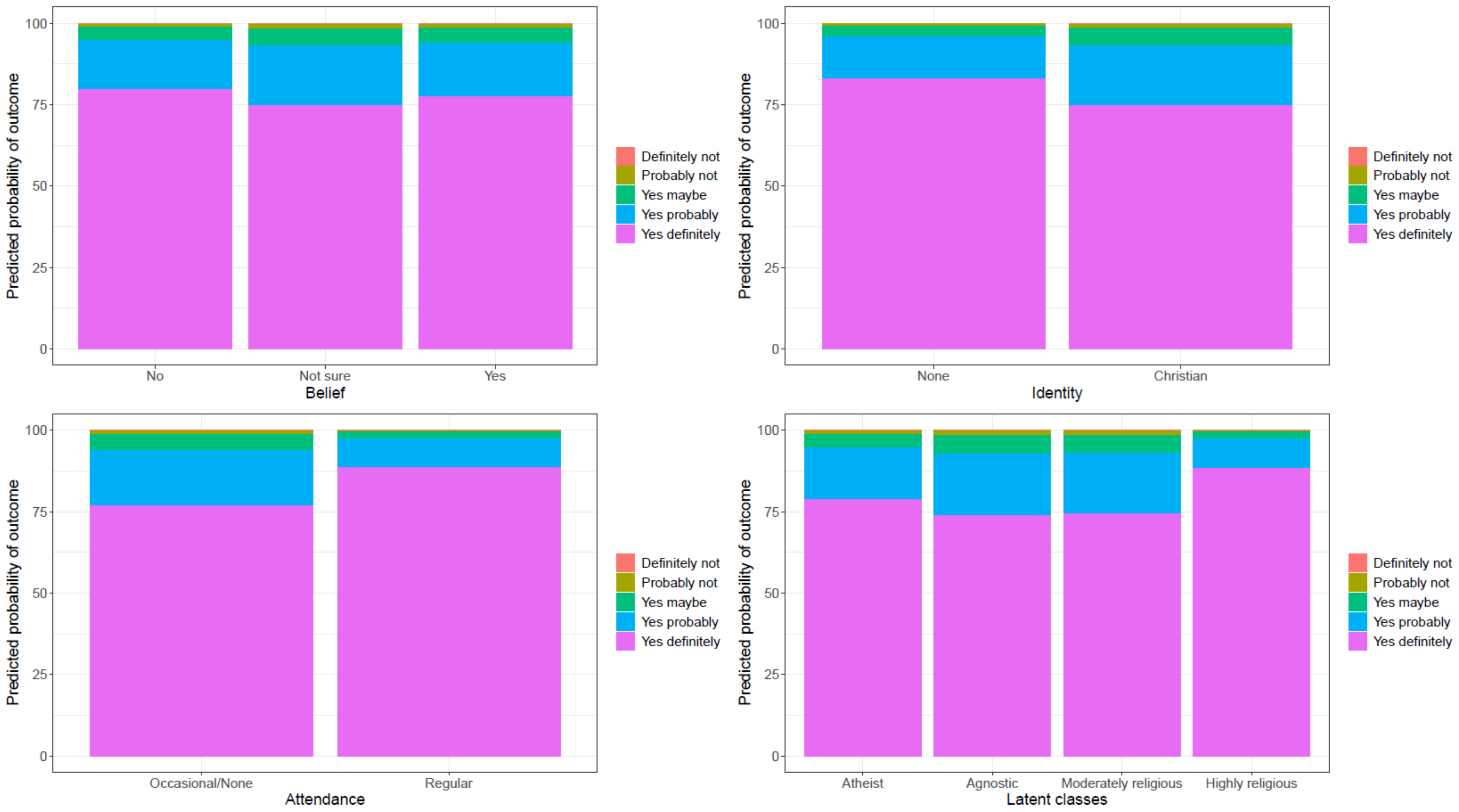

Figure S81: Predicted probabilities of the partners ordinal regression models with 'belief that the climate is changing' as the outcome and the religious identity (with the Christian denominations separated) as the exposure.

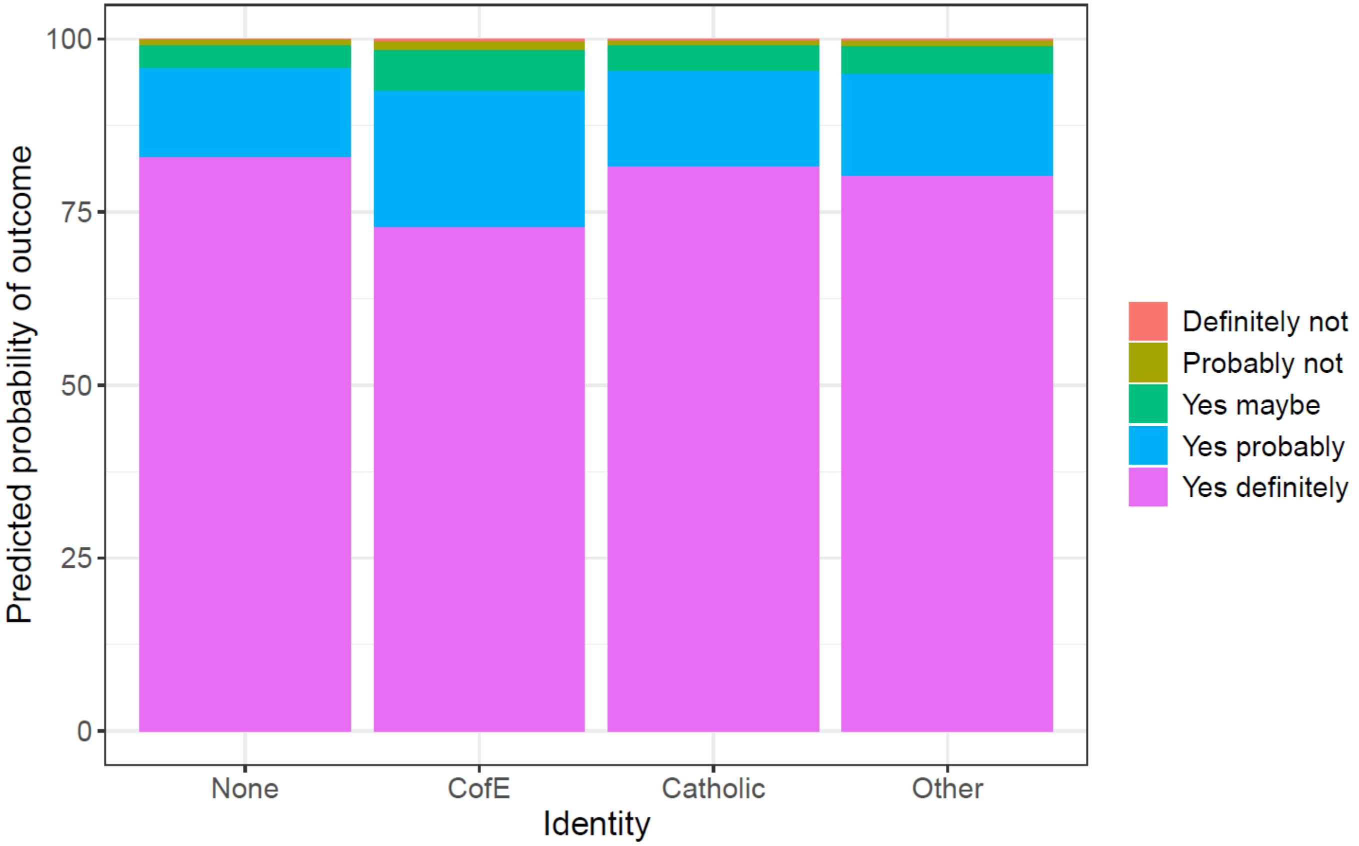

Figure S82: Results of the partners ordinal regression models with 'concerned about the impact of climate change' as the outcome for four religious exposures (belief [*n* = 1,110], identity [*n* = 1,100], attendance [*n* = 1,112], and latent classes [*n* = 1,120]; models are separated by dashed horizontal lines). Odds ratios above 1 indicate an increased concern regarding climate change. See table S22 for full results.

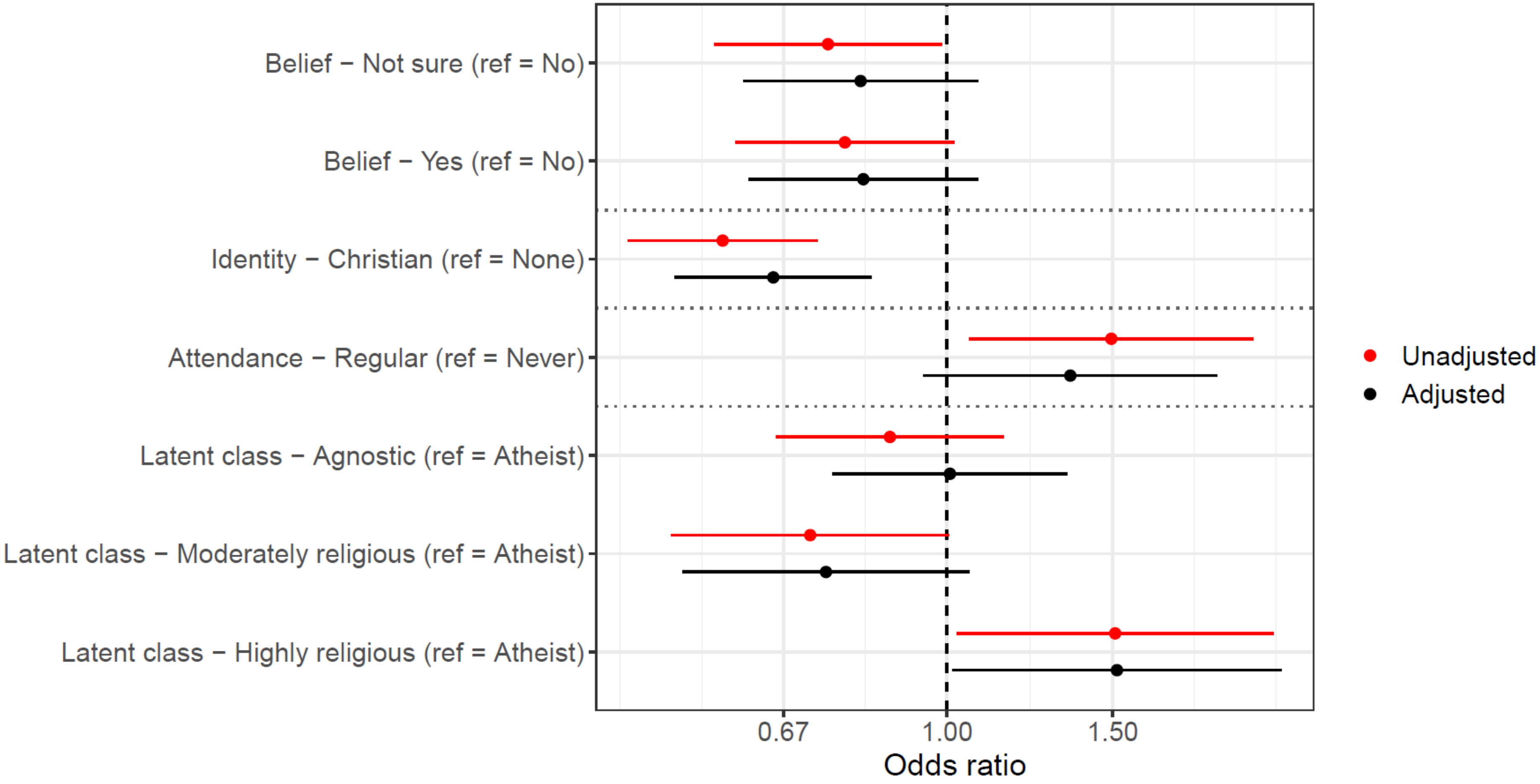

Figure S83: Predicted probabilities of the partners ordinal regression models with 'concerned about the impact of climate change' as the outcome for four religious exposures (belief, identity, attendance and latent classes).

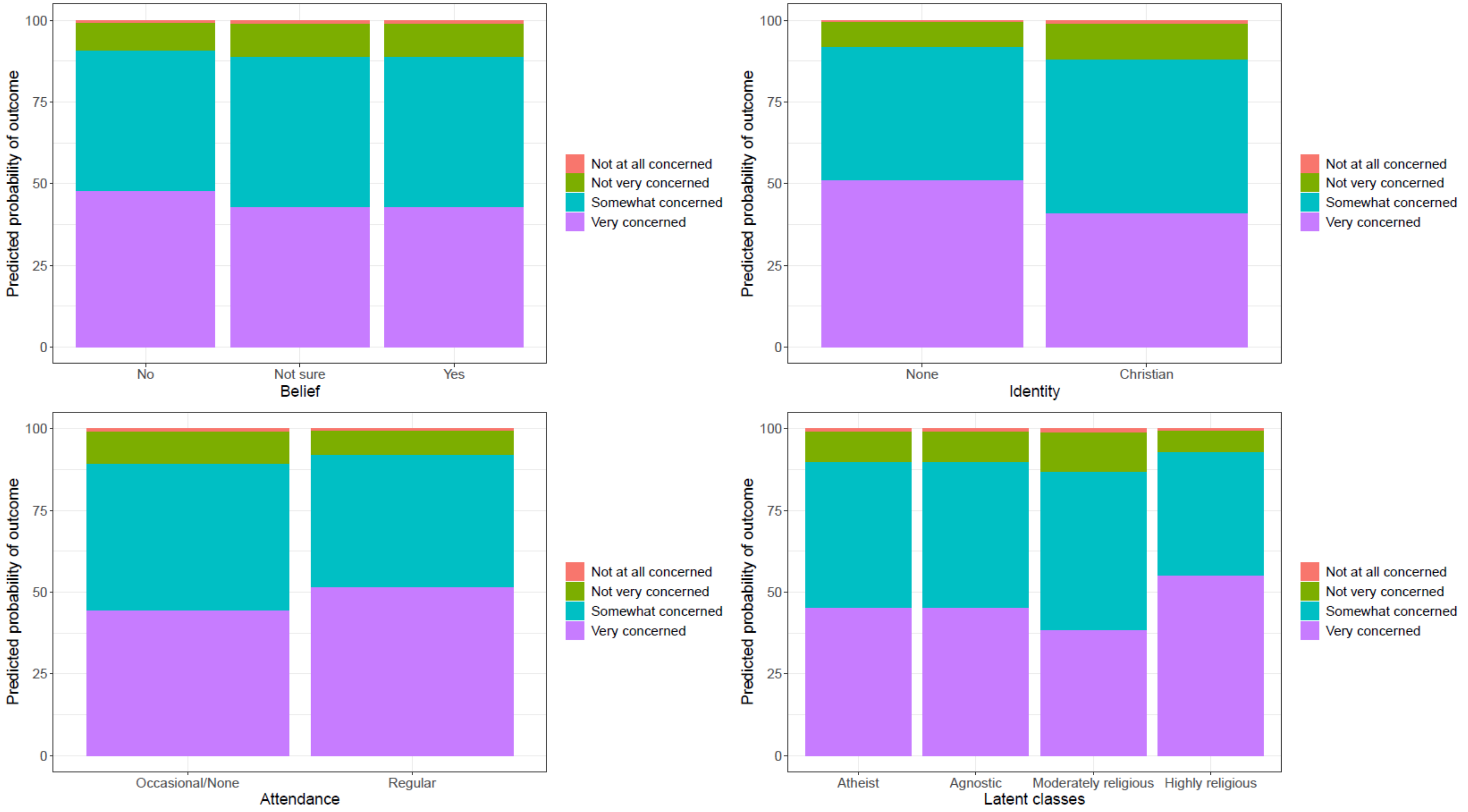

Figure S84: Predicted probabilities of the partners ordinal regression models with 'concerned about the impact of climate change' as the outcome and the religious identity (with the Christian denominations separated) as the exposure.

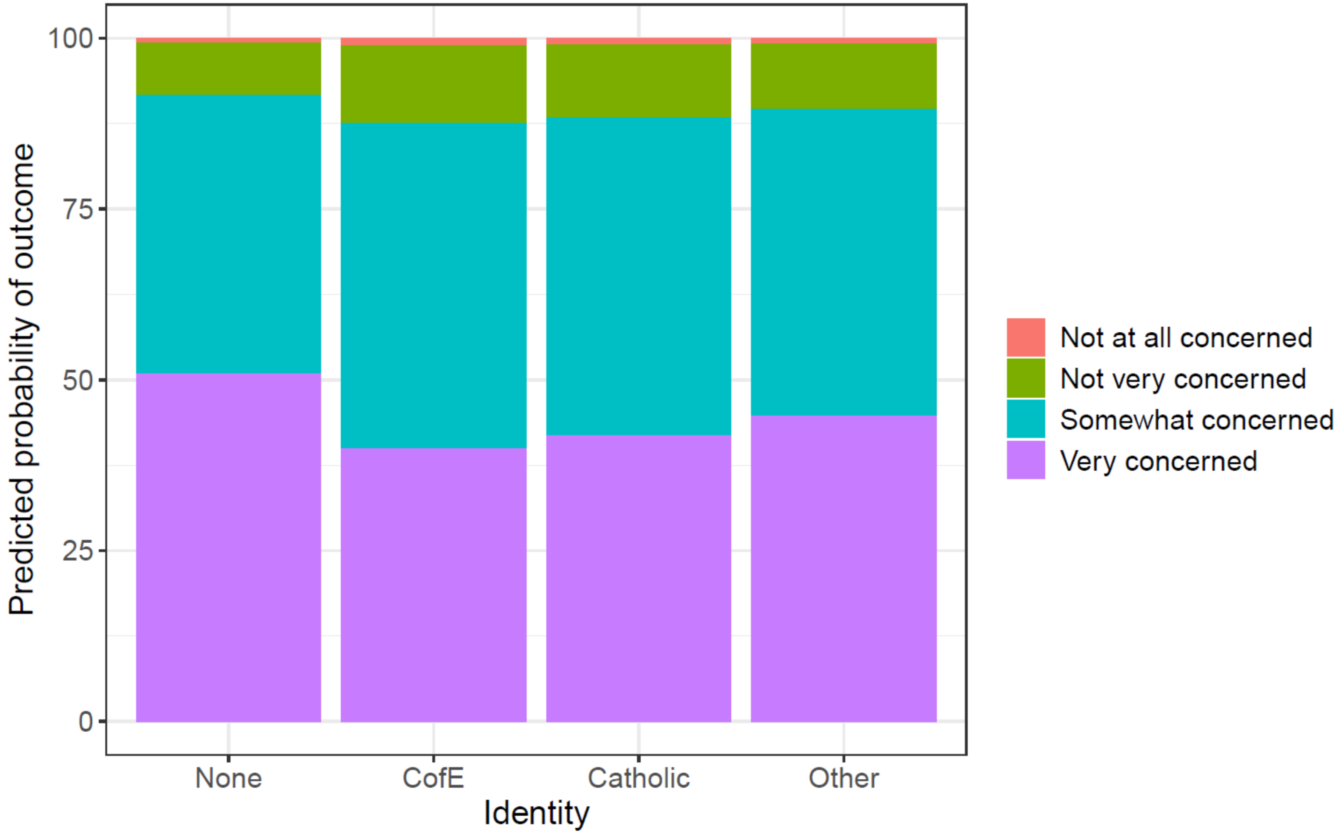

Figure S85: Results of the partners ordinal regression models with 'believes that humans are to blame for climate change' as the outcome for four religious exposures (belief [*n* = 1,111], identity [*n* = 1,100], attendance [*n* = 1,113], and latent classes [*n* = 1,121]; models are separated by dashed horizontal lines). Odds ratios above 1 indicate an increased belief that humans are to blame for climate change. See table S23 for full results.

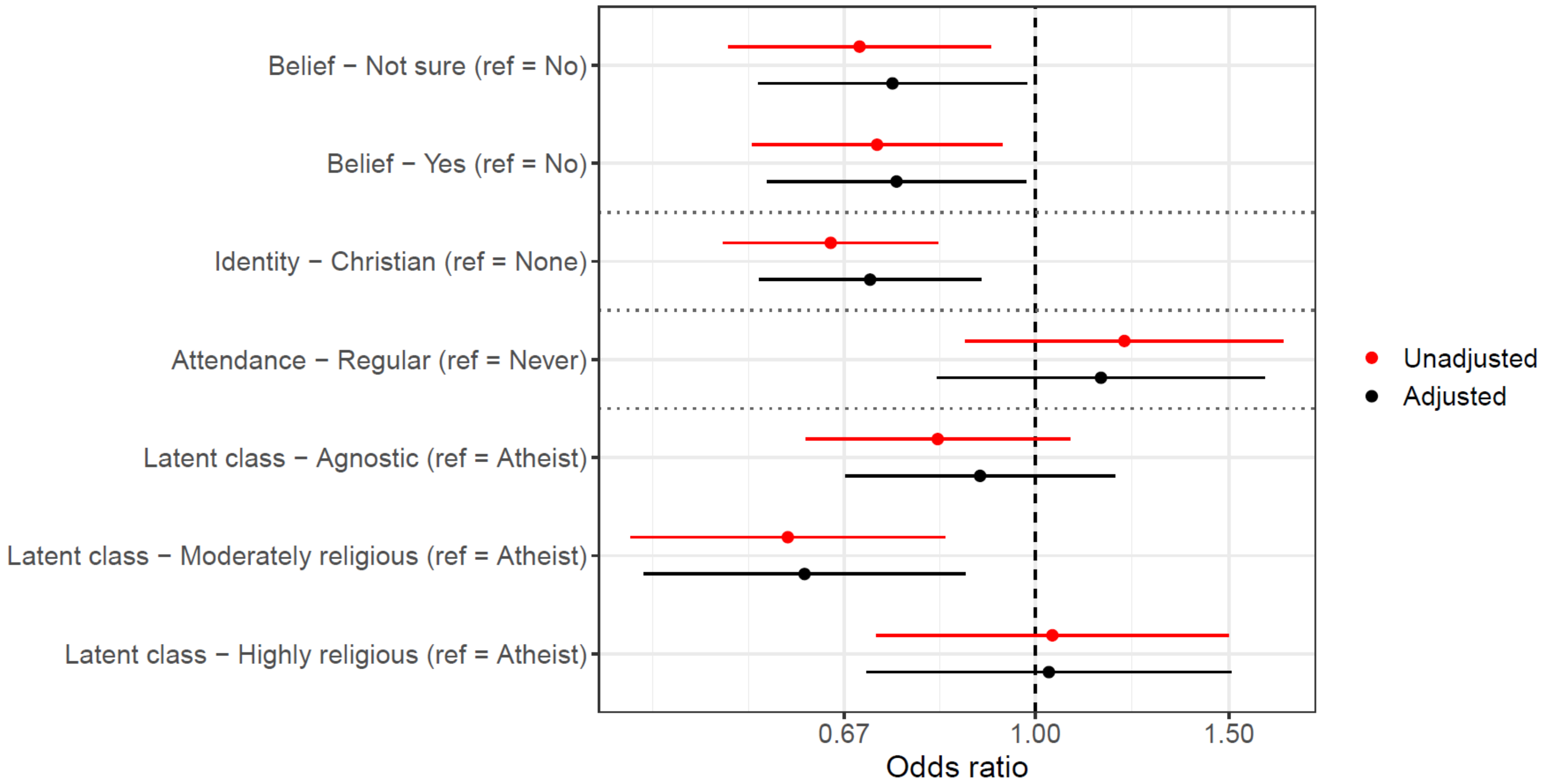

Figure S86: Predicted probabilities of the partners ordinal regression models with 'believes that humans are to blame for climate change' as the outcome for four religious exposures (belief, identity, attendance and latent classes).

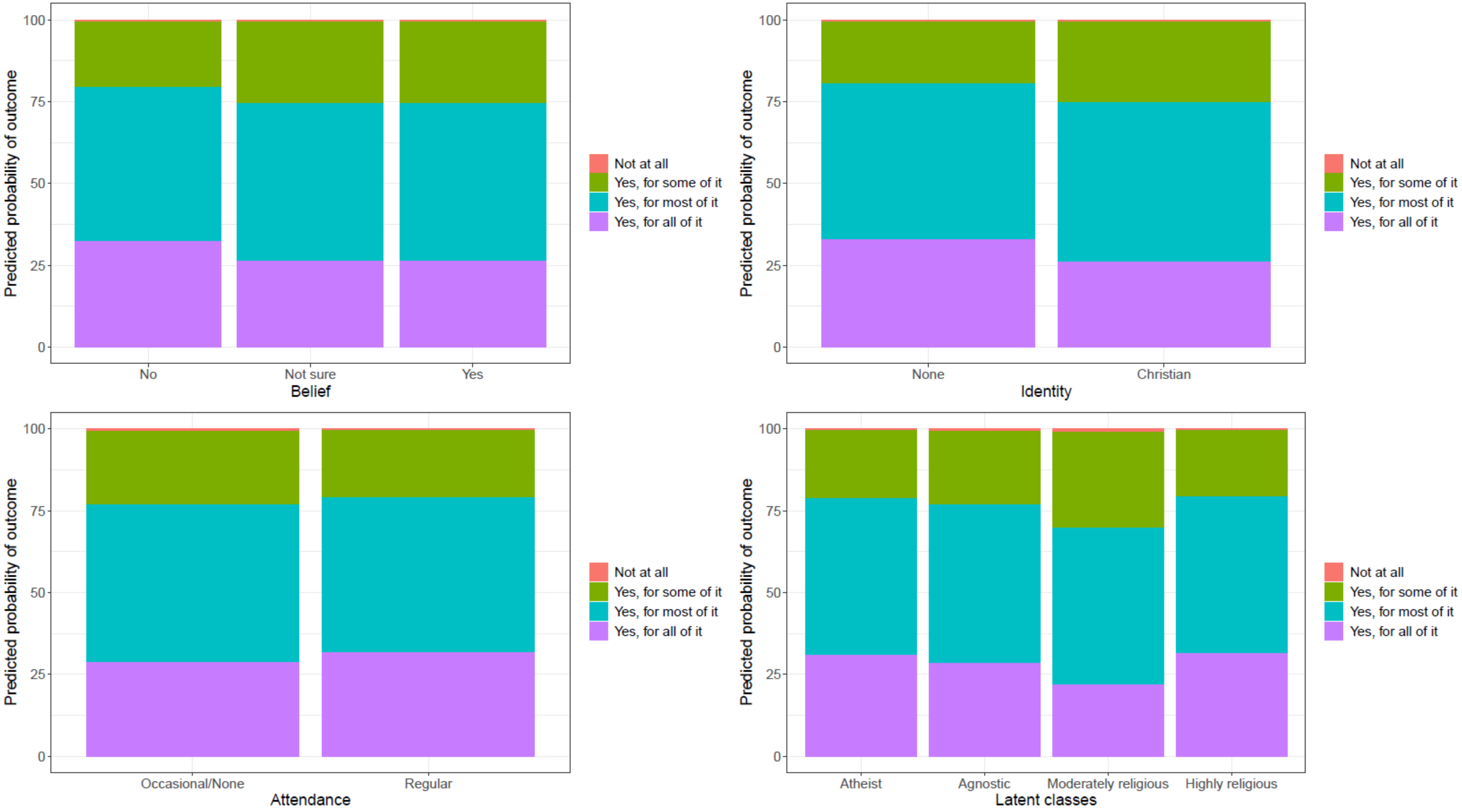

Figure S87: Predicted probabilities of the partners ordinal regression models with 'believes that humans are to blame for climate change' as the outcome and the religious identity (with the Christian denominations separated) as the exposure.

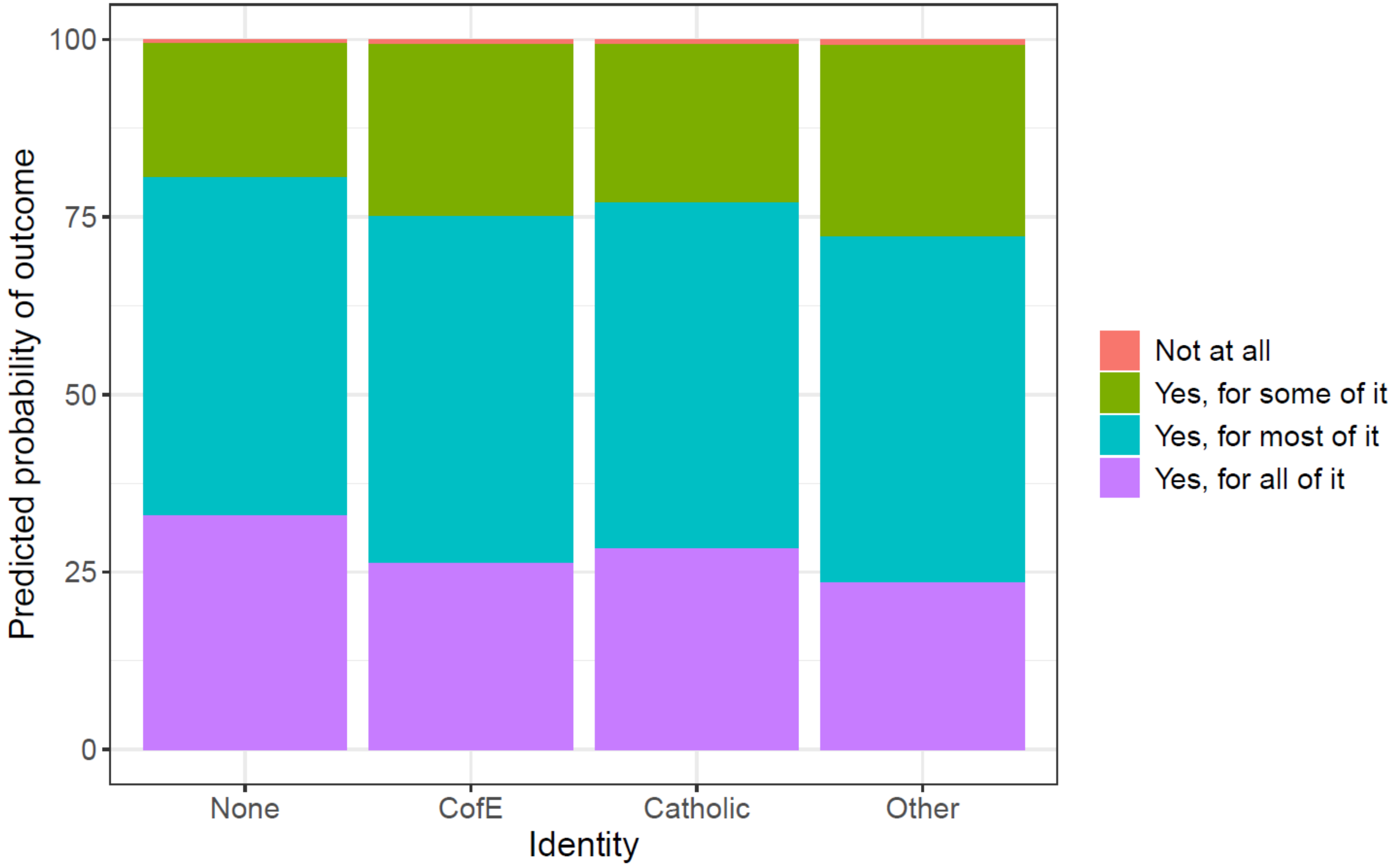

Figure S88: Results of the partners multinomial regression models with ‘thinks that personal actions will make a difference to long-term climate change’ as the outcome for four religious exposures (belief [*n* = 1,110], identity [*n* = 1,099], attendance [*n* = 1,112], and latent classes [*n* = 1,120]; models are separated by dashed horizontal lines). See table S24 for full results.

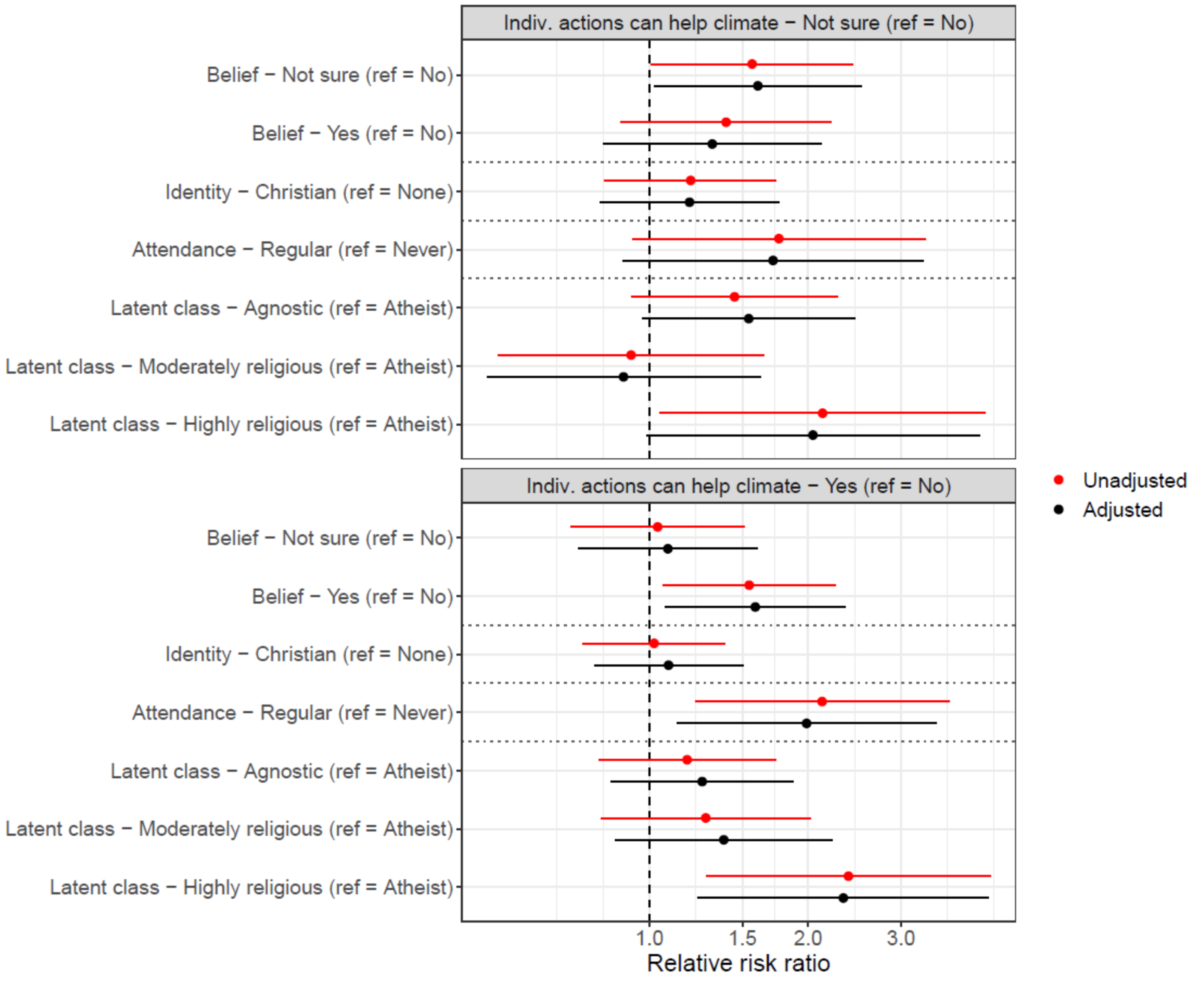

Figure S89: Predicted probabilities of the partners multinomial regression models with 'thinks that personal actions will make a difference to long-term climate change' as the outcome for four religious exposures (belief, identity, attendance and latent classes).

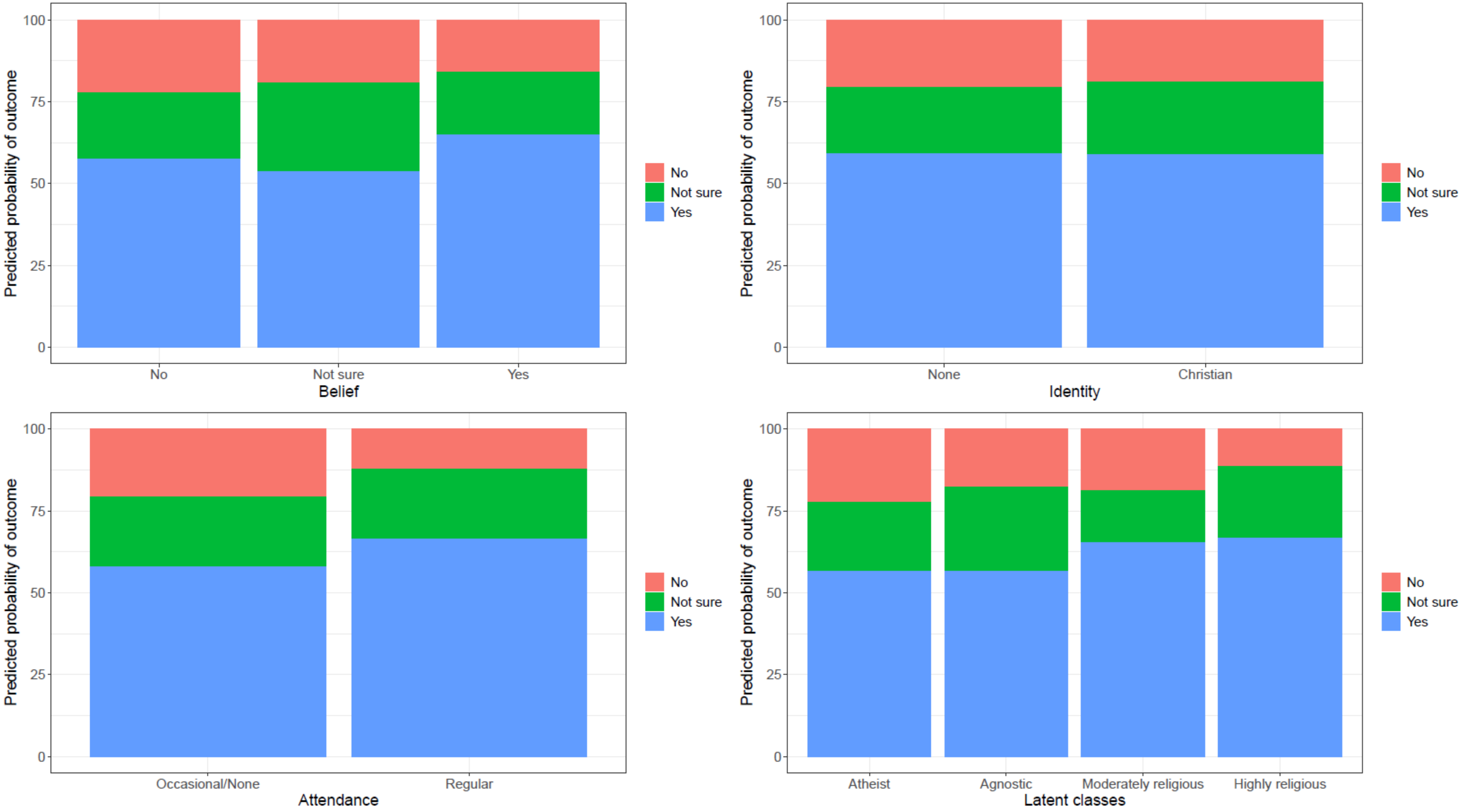

Figure S90: Predicted probabilities of the partners multinomial regression models with 'thinks that personal actions will make a difference to long-term climate change' as the outcome and the religious identity (with the Christian denominations separated) as the exposure.

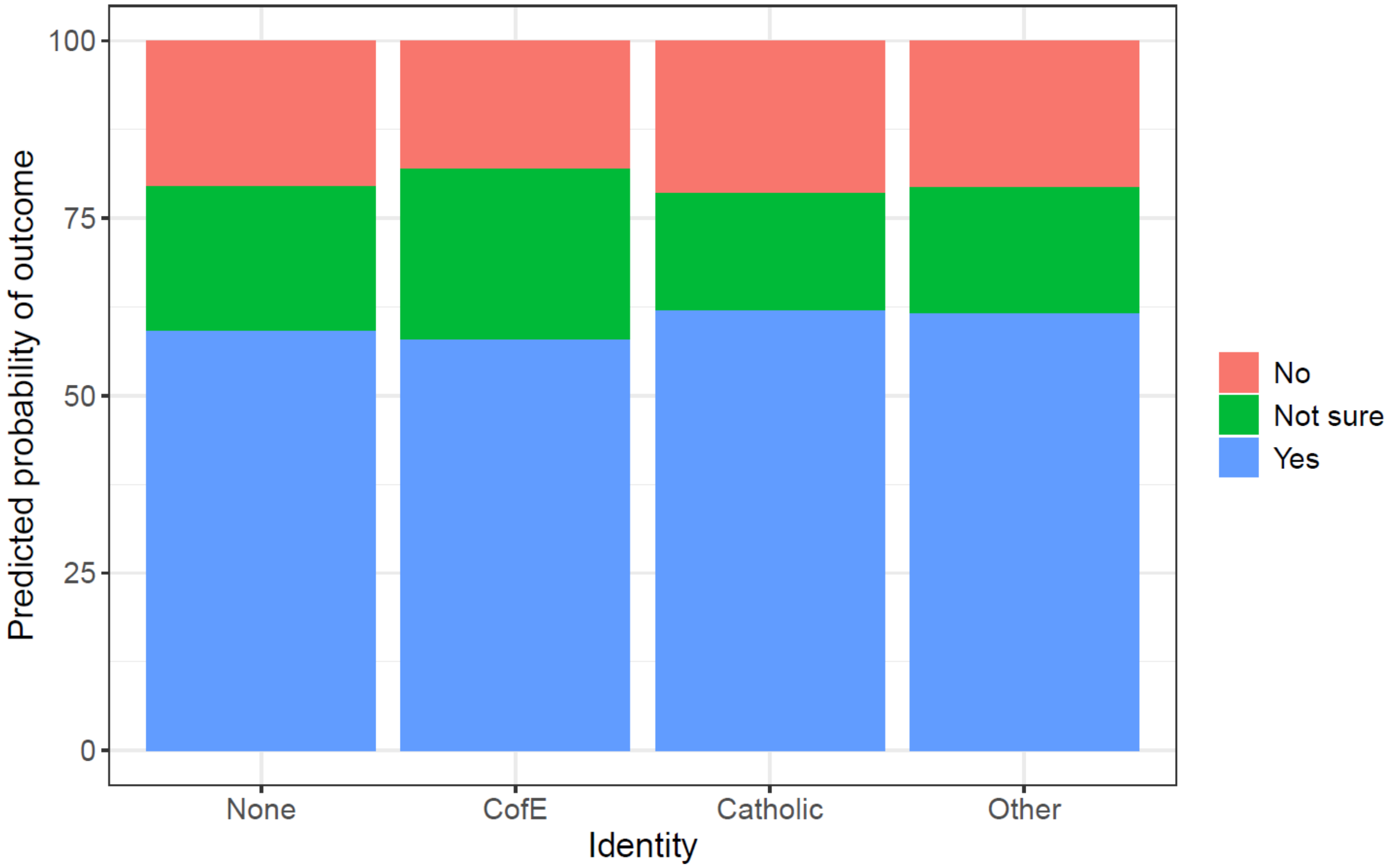

Figure S91: Results of the partners linear regression models with 'total number of actions performed due to climate change' as the outcome for four religious exposures (belief [*n* = 984], identity [*n* = 975], attendance [*n* = 989], and latent classes [*n* = 993]; models are separated by dashed horizontal lines). Values above 0 indicate an increased number of pro-environmental actions performed. See table S25 for full results.

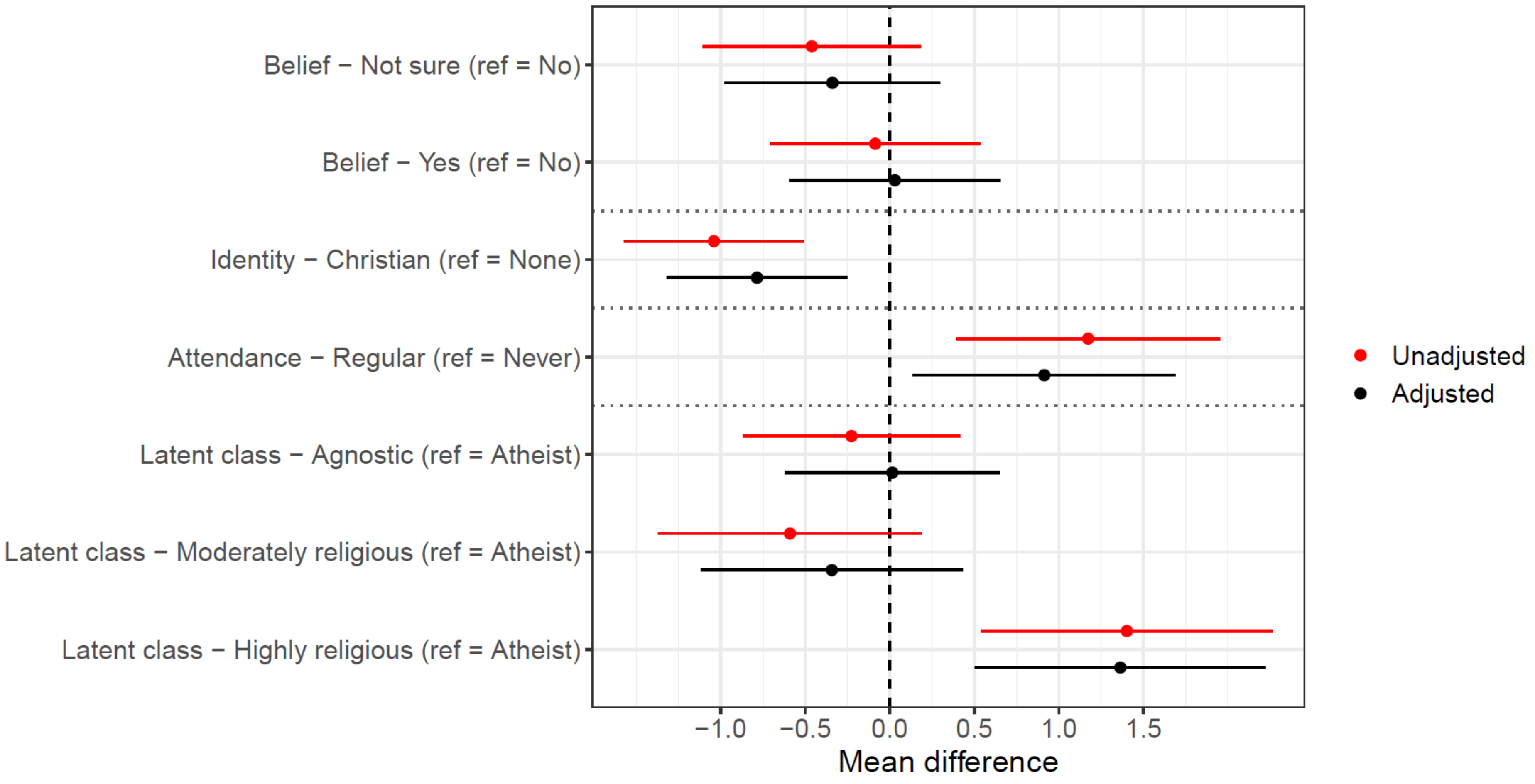

Figure S92: Predicted total number of actions performed due to climate change for four religious exposures (belief, identity, attendance and latent classes) based on the partners linear regression models.

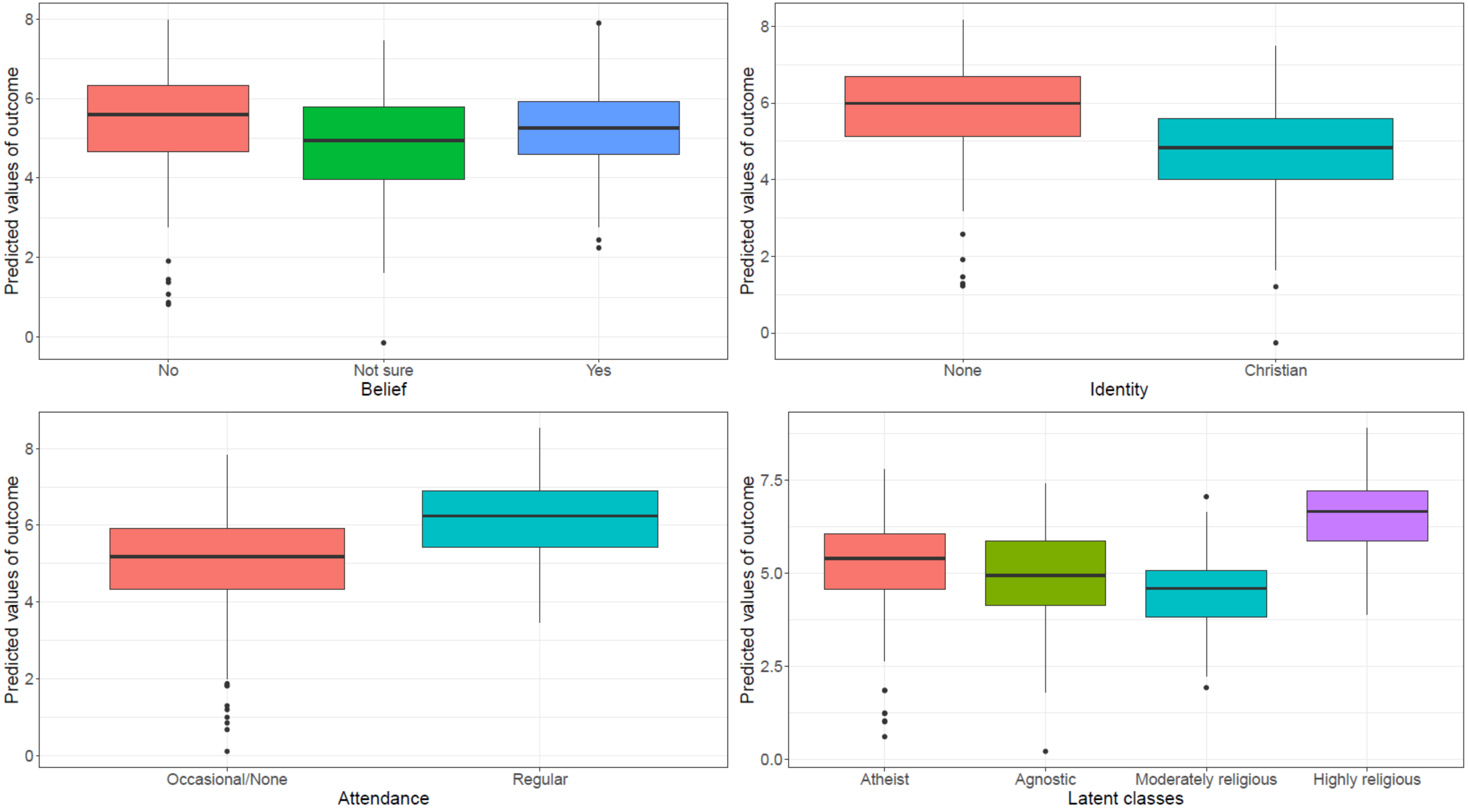

Figure S93: Predicted total number of actions performed due to climate change for the religious identity (with the Christian denominations separated) as the exposure based on the partners linear regression models.

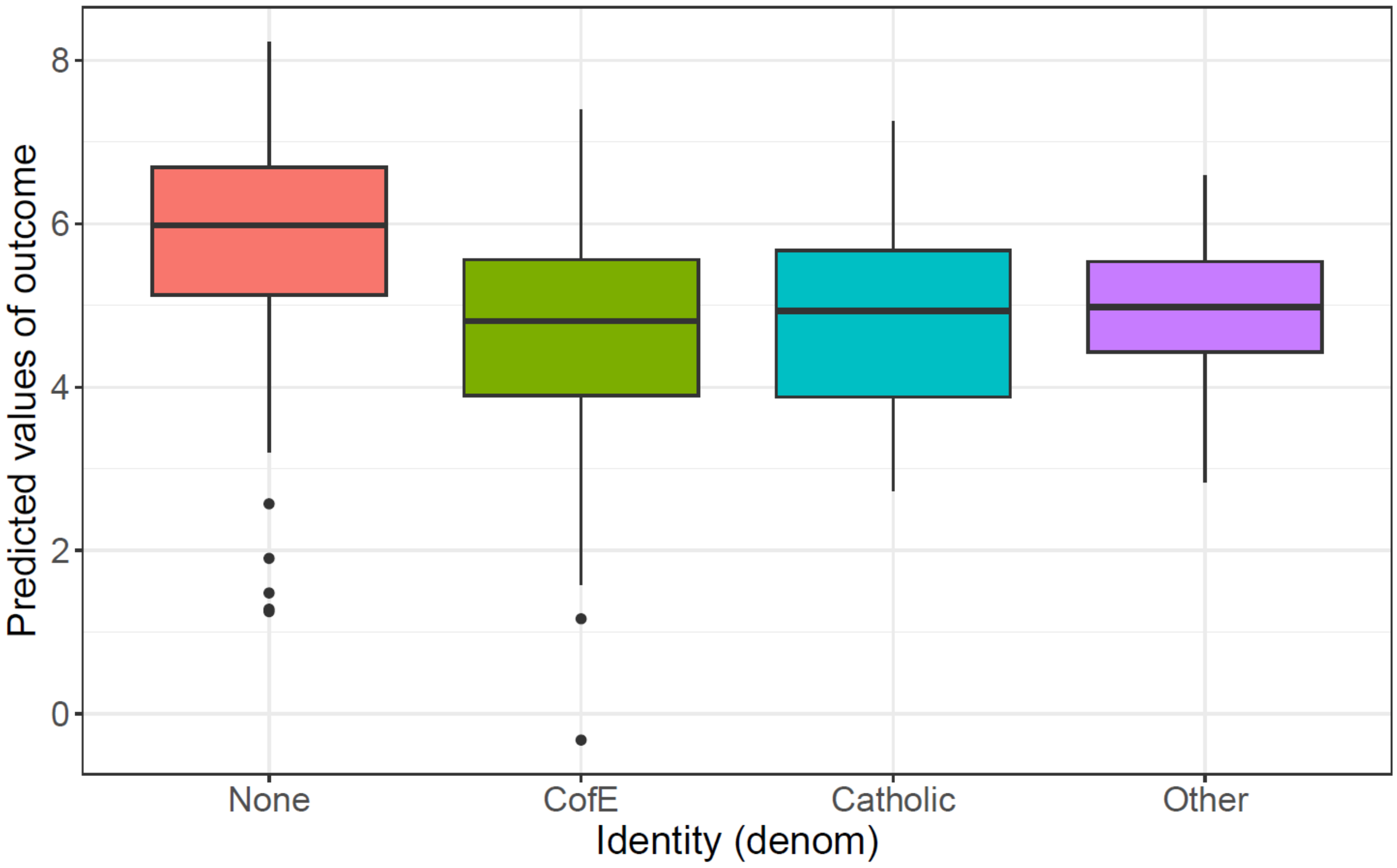

Figure S94: Results of the partners Poisson regression models with 'total number of actions performed due to climate change' as the outcome for four religious exposures (belief [*n* = 984], identity [*n* = 975], attendance [*n* = 989], and latent classes [*n* = 993]; models are separated by dashed horizontal lines). Incidence rate ratios above 1 indicate an increased number of pro-environmental actions performed. See table S26 for full results.

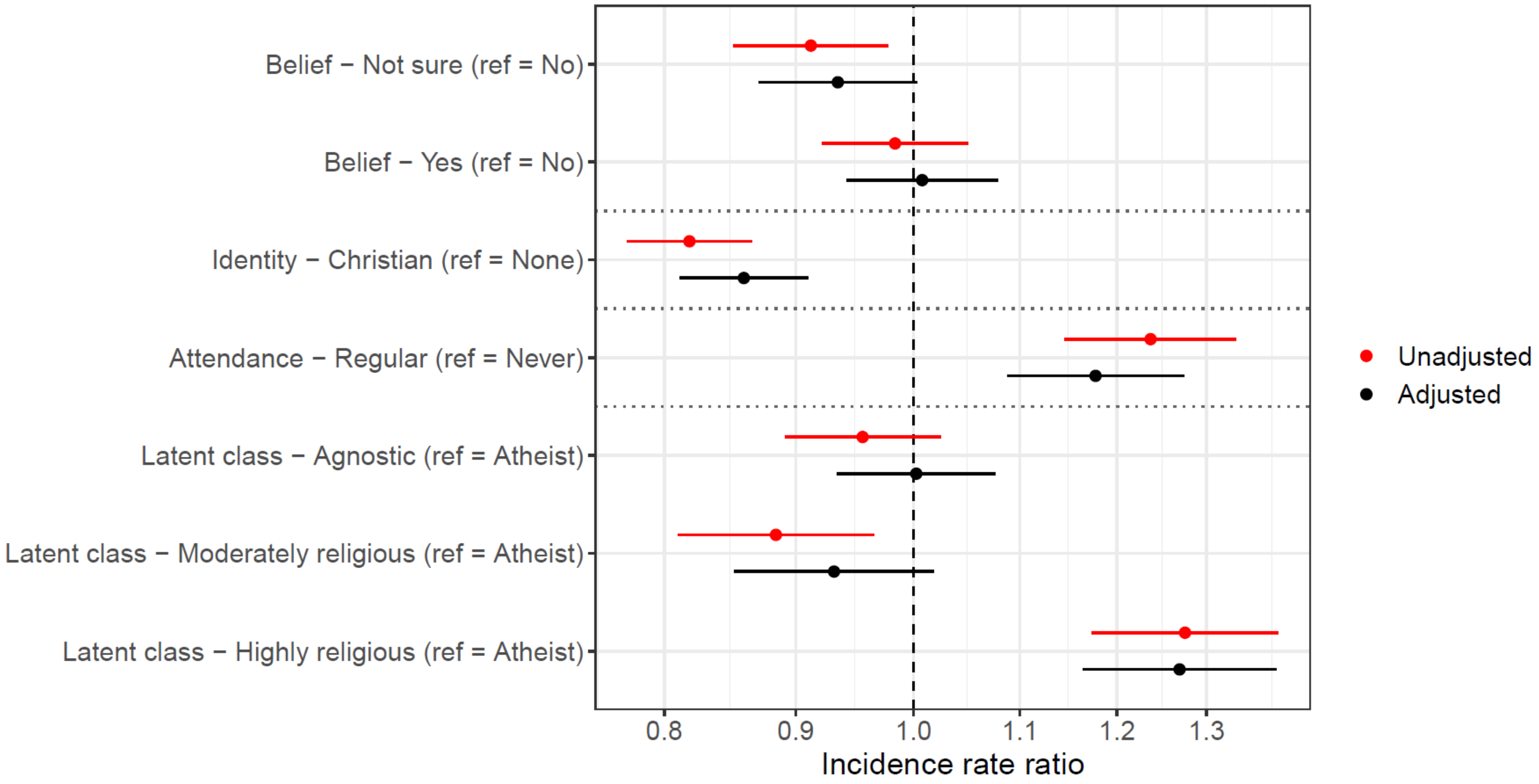

Figure S95: Predicted total number of actions performed due to climate change for four religious exposures (belief, identity, attendance and latent classes) based on the partners Poisson regression models.

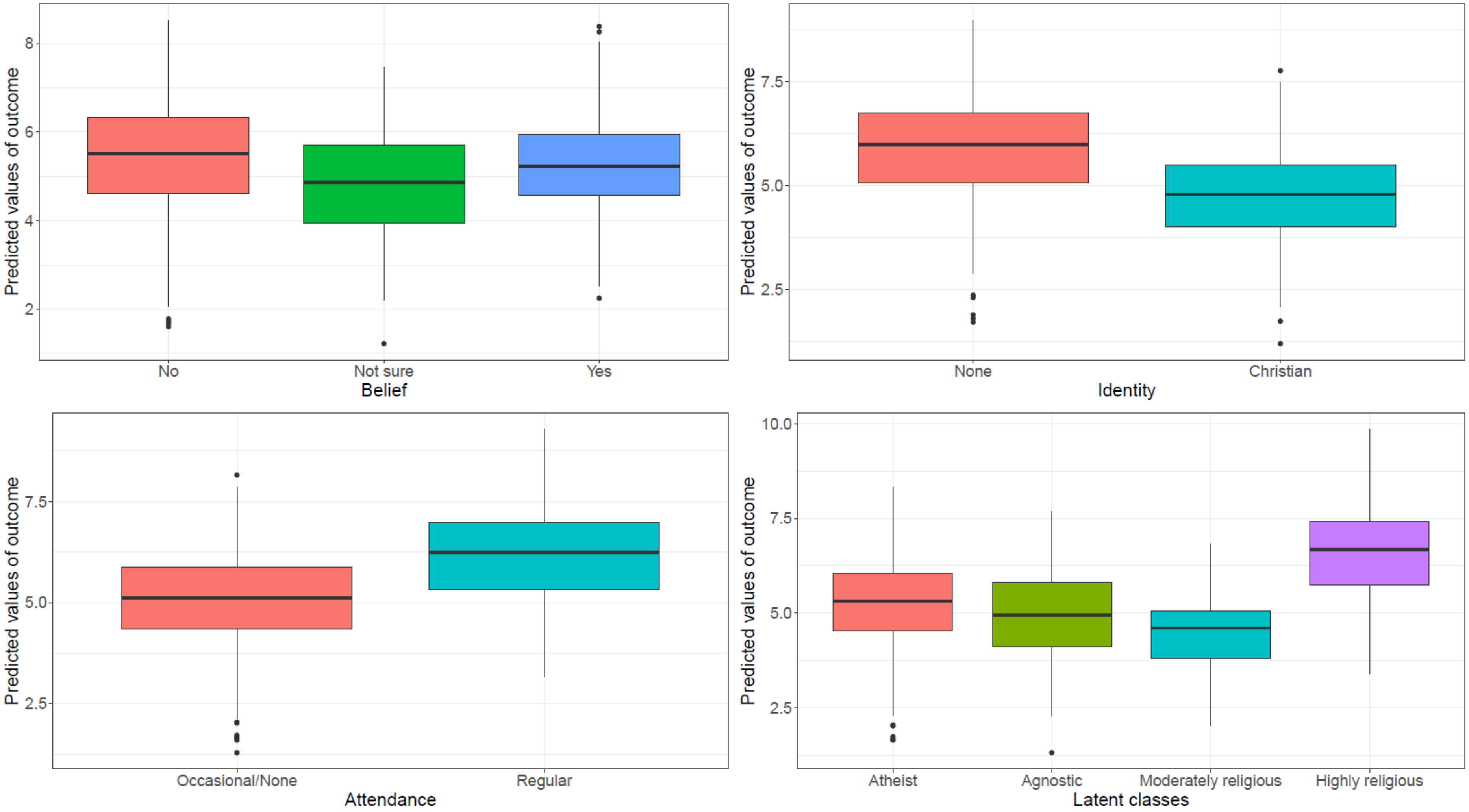

Figure S96: Predicted total number of actions performed due to climate change for the religious identity (with the Christian denominations separated) as the exposure based on the partners Poisson regression models.

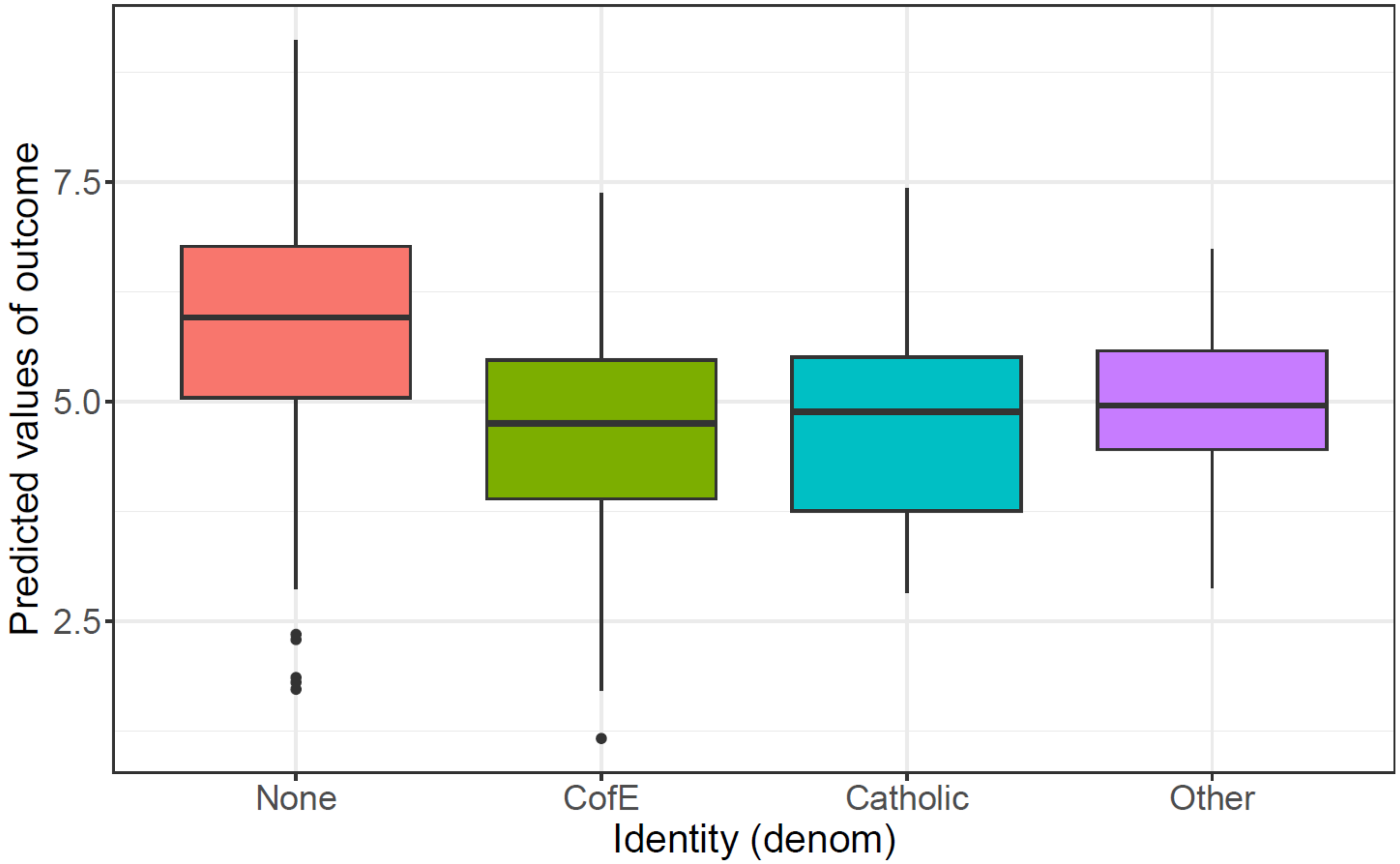

Figure S97: Results of the partners zero-inflated Poisson regression models with 'total number of actions performed due to climate change' as the outcome for four religious exposures (belief [*n* = 984], identity [*n* = 975], attendance [*n* = 989], and latent classes [*n* = 993]; models are separated by dashed horizontal lines). Incidence rate ratios above 1 indicate an increased number of pro-environmental actions performed, while odds ratios above 1 indicate an excess of zeros. See table S27 for full results.

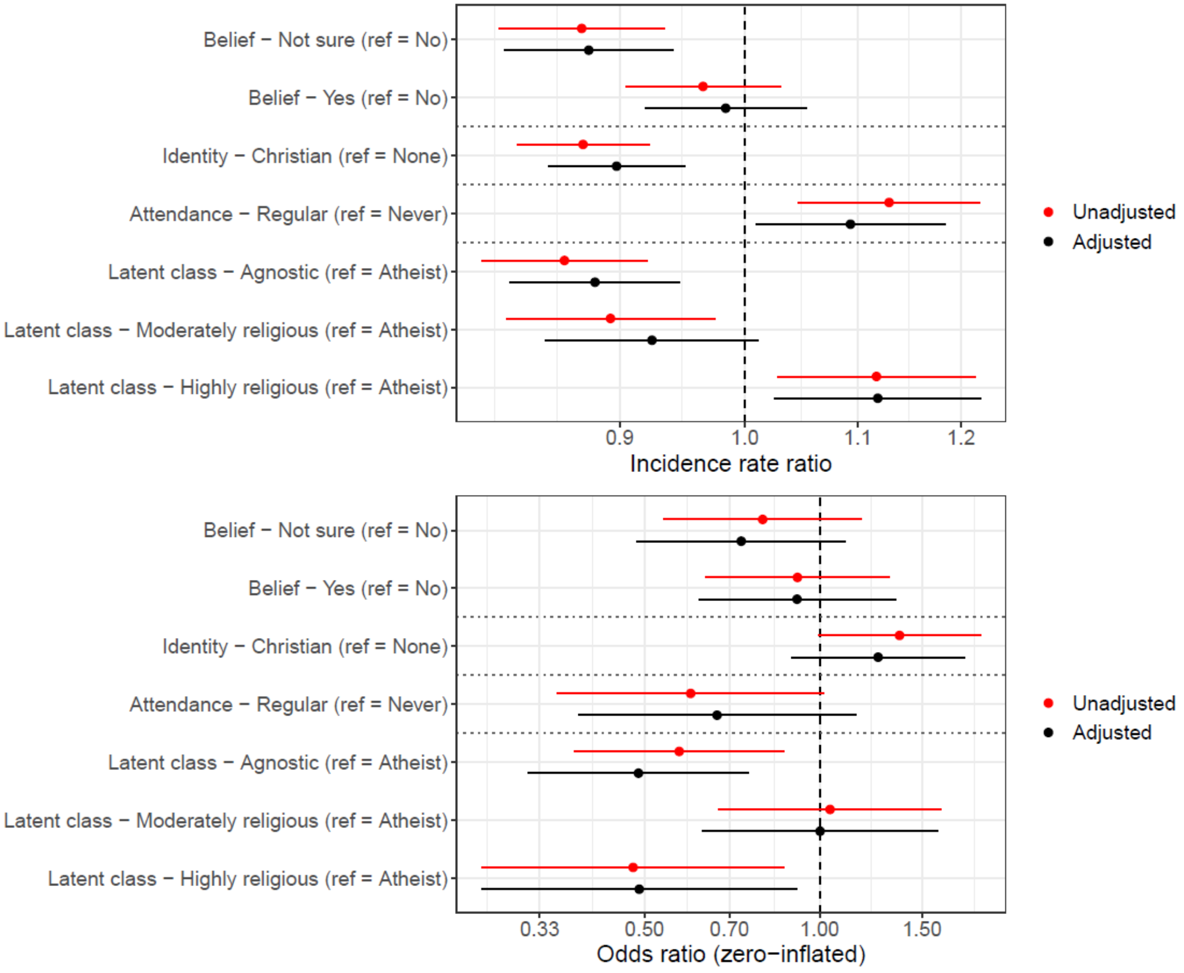

Figure S98: Predicted total number of actions performed due to climate change for four religious exposures (belief, identity, attendance and latent classes) based on the partners zero-inflated Poisson regression models.

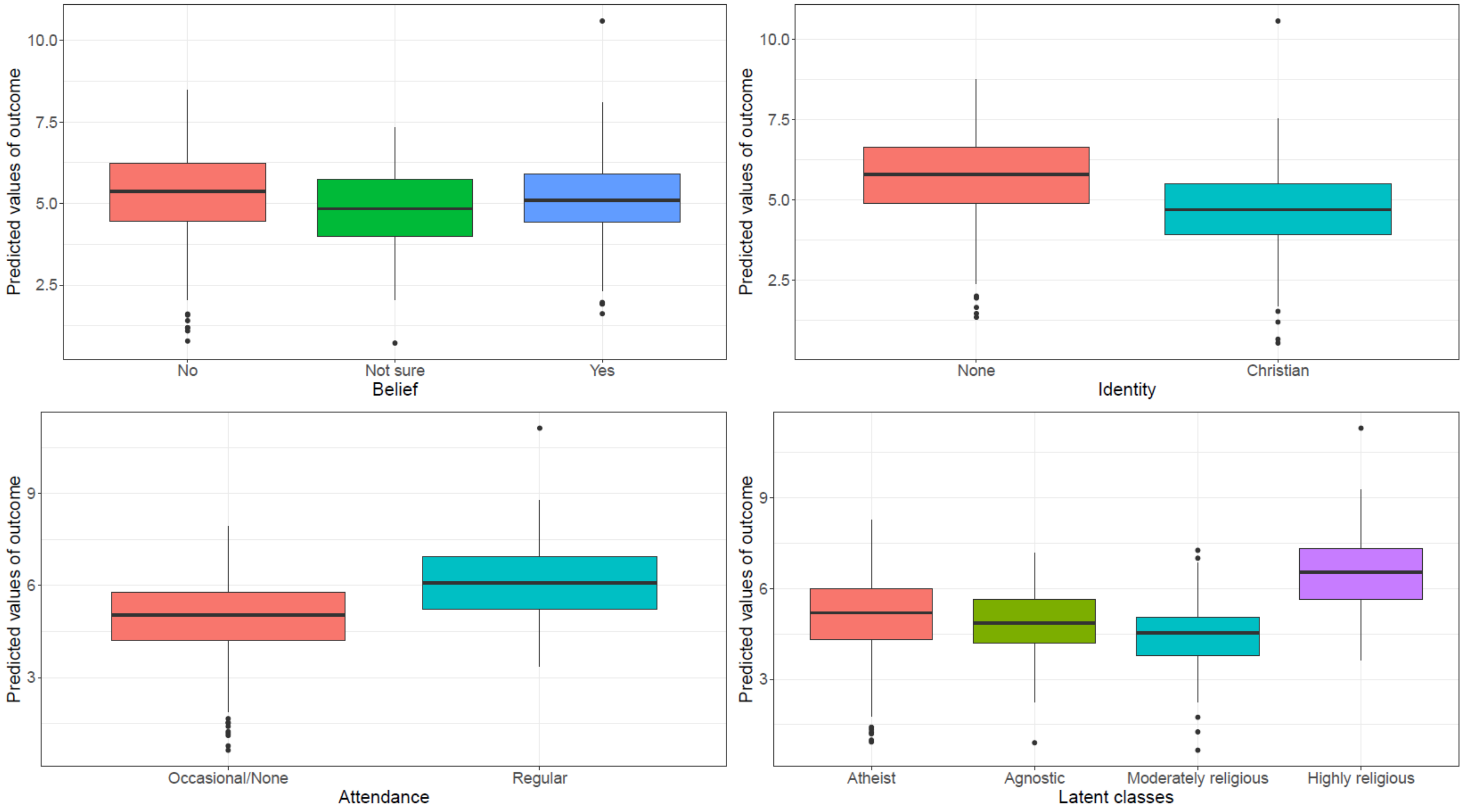

Figure S99: Predicted total number of actions performed due to climate change for the religious identity (with the Christian denominations separated) as the exposure based on the partners zero-inflated Poisson regression models.

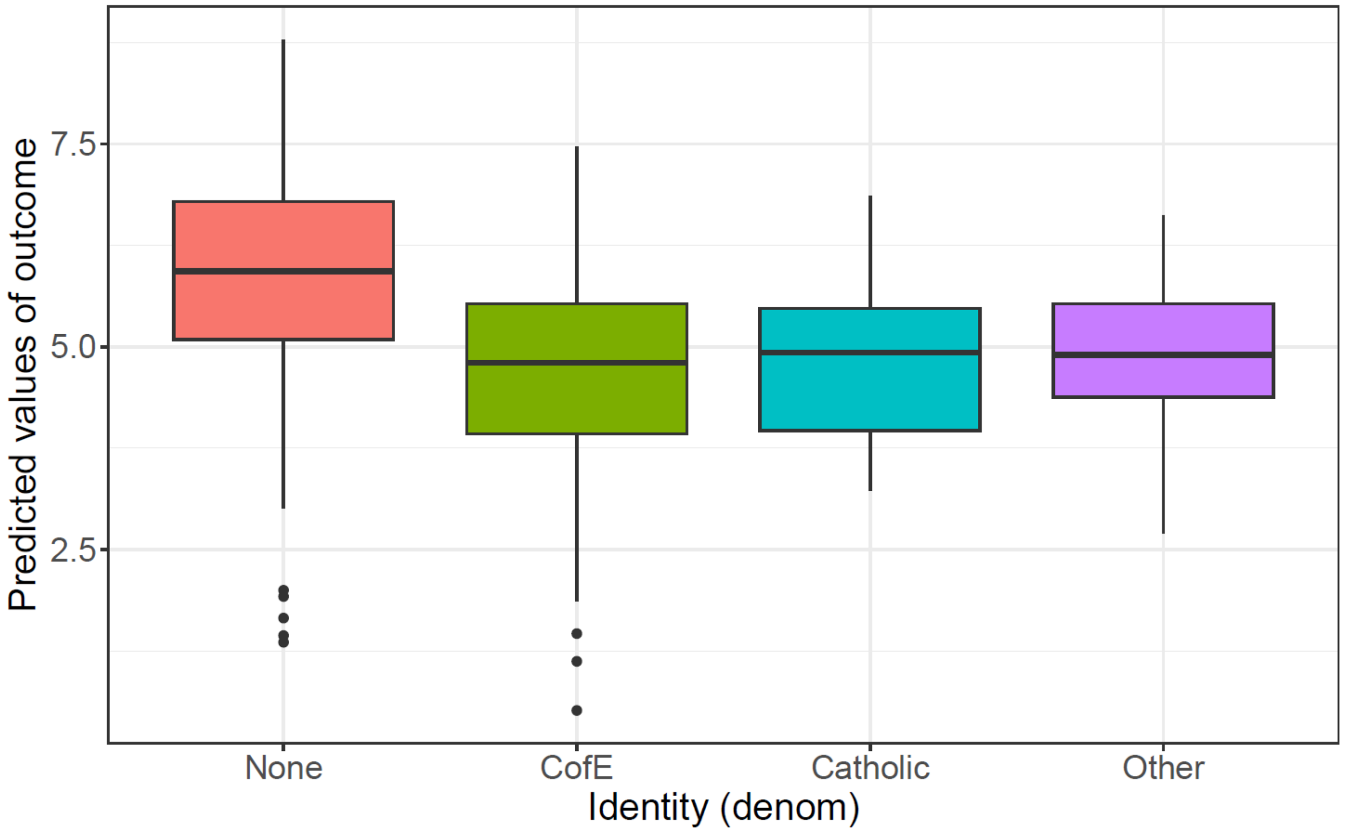

Figure S100: Results of the partners linear regression models with 'total number of actions performed due to climate change (excluding ones which may be prohibitively costly)' as the outcome for four religious exposures (belief [*n* = 1,002], identity [*n* = 993], attendance [*n* = 1,007], and latent classes [*n* = 1,011]; models are separated by dashed horizontal lines). Values above 0 indicate an increased number of pro-environmental actions performed. See table S28 for full results.

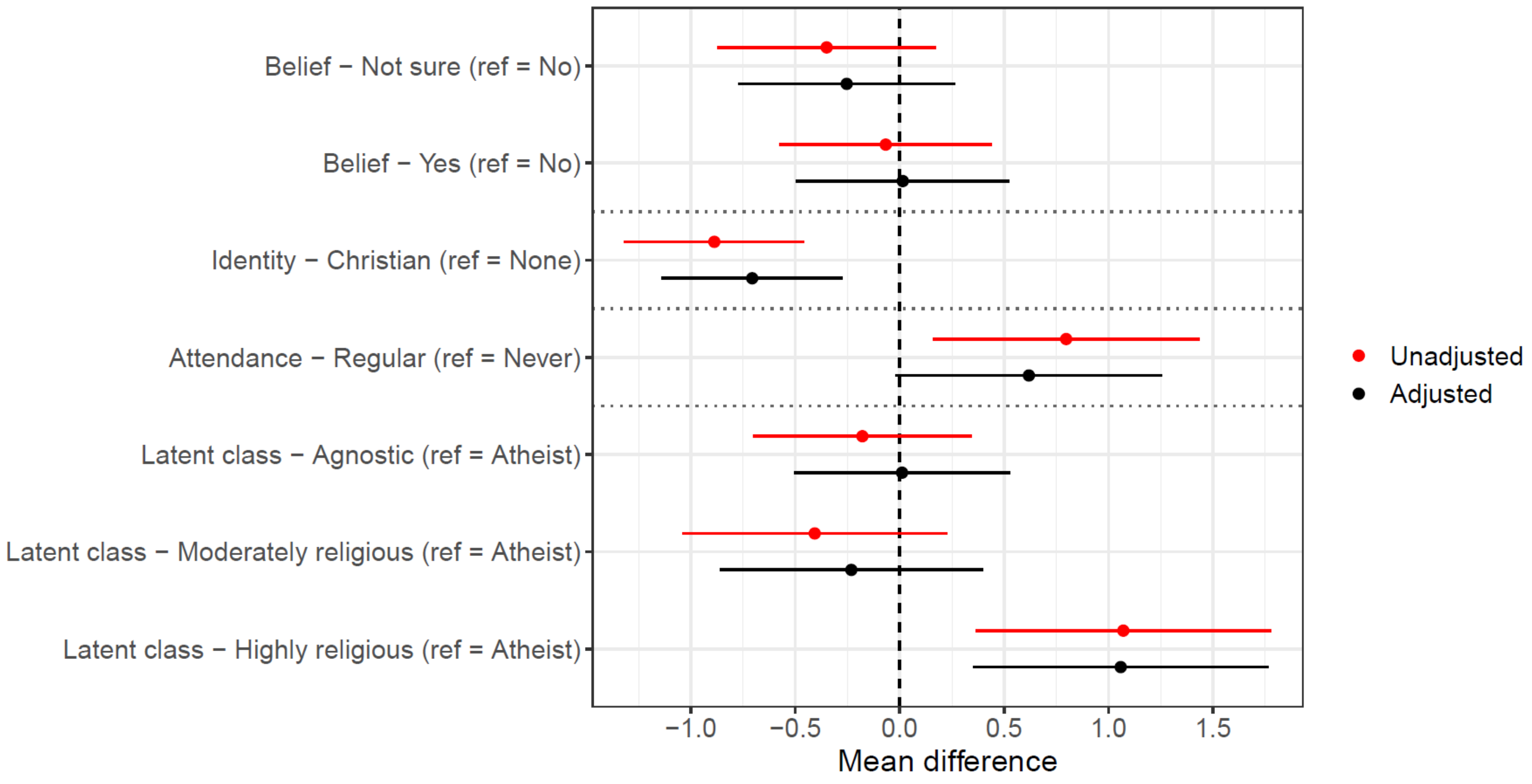

Figure S101: Predicted total number of actions (excluding ones which may be prohibitively costly) performed due to climate change for four religious exposures (belief, identity, attendance and latent classes) based on the partners linear regression models.

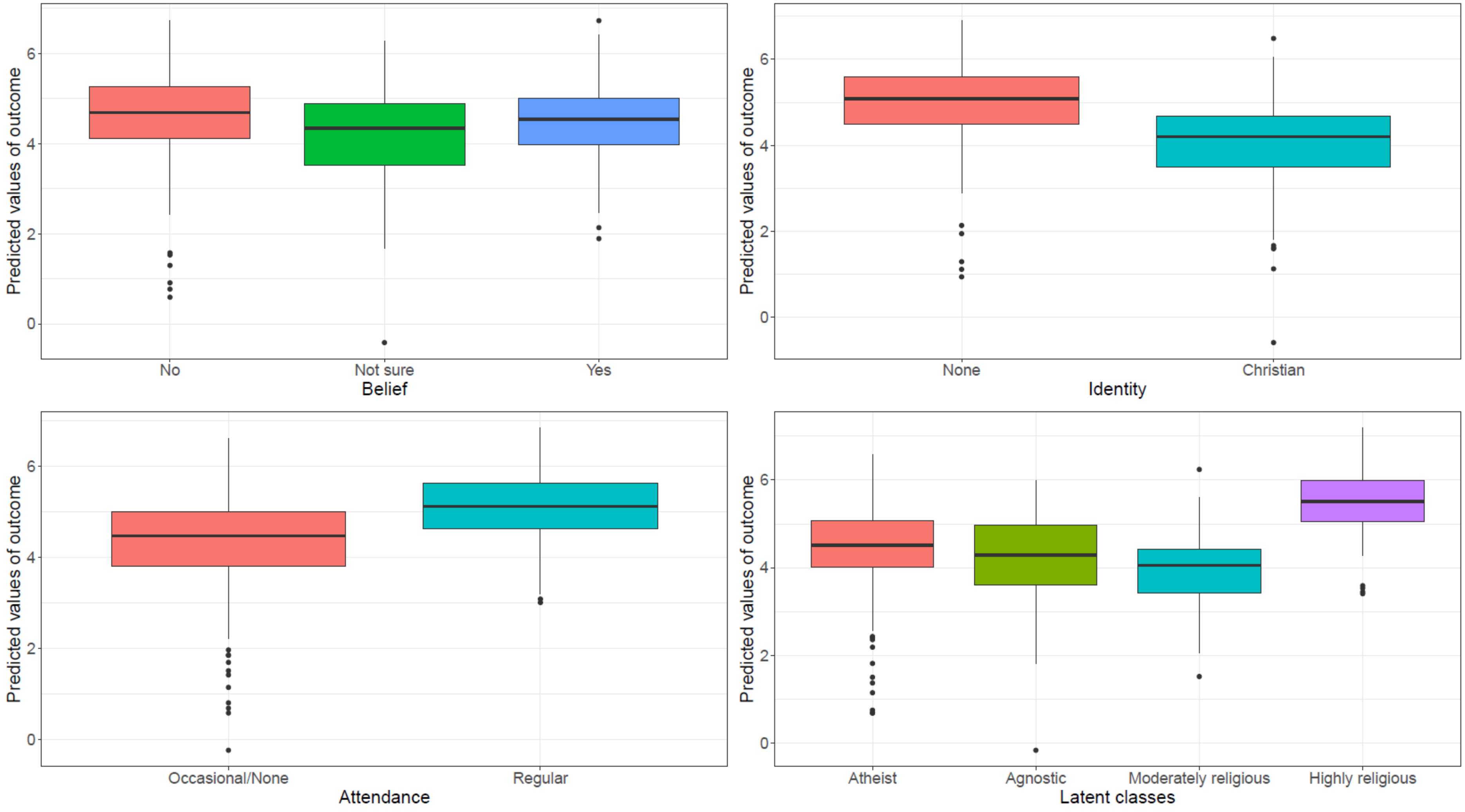

Figure S102: Predicted total number of actions (excluding ones which may be prohibitively costly) performed due to climate change for the religious identity (with the Christian denominations separated) as the exposure based on the partners linear regression models.

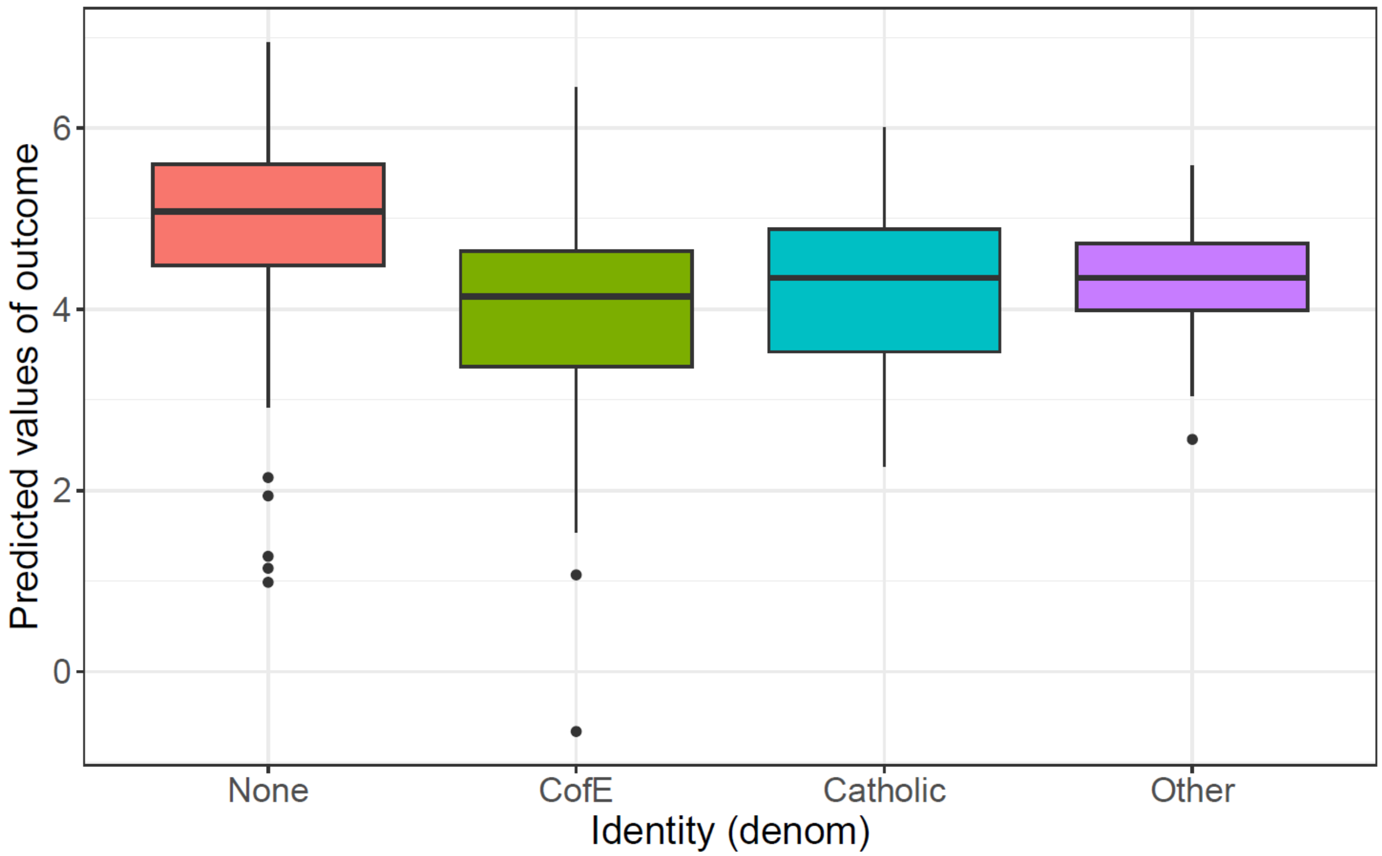

Figure S103: Results of the partners Poisson regression models with 'total number of actions performed due to climate change (excluding ones which may be prohibitively costly)' as the outcome for four religious exposures (belief [*n* = 1,002], identity [*n* = 993], attendance [*n* = 1,007], and latent classes [*n* = 1,011]; models are separated by dashed horizontal lines). Incidence rate ratios above 1 indicate an increased number of pro-environmental actions performed. See table S29 for full results.

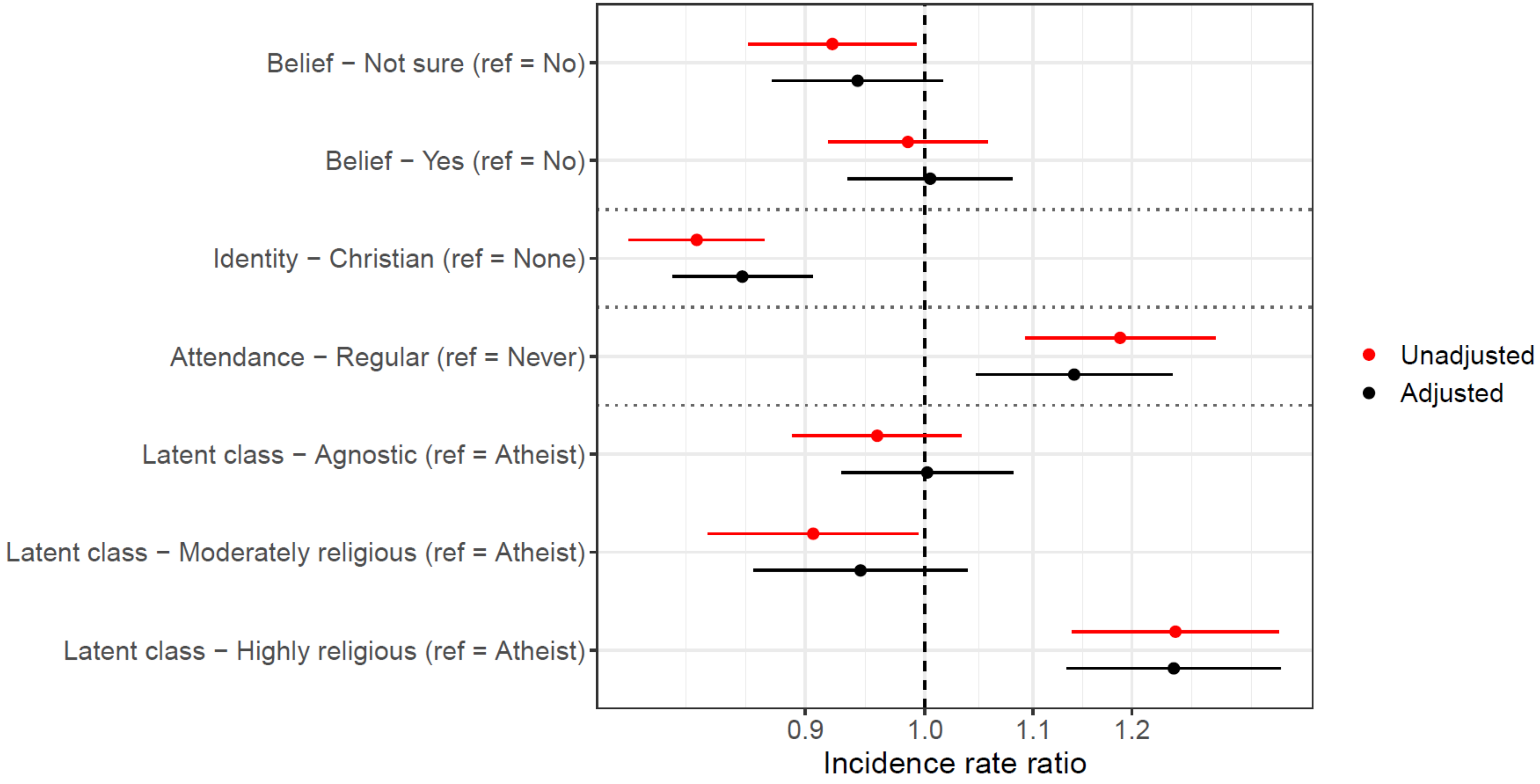

Figure S104: Predicted total number of actions (excluding ones which may be prohibitively costly) performed due to climate change for four religious exposures (belief, identity, attendance and latent classes) based on the partners Poisson regression models.

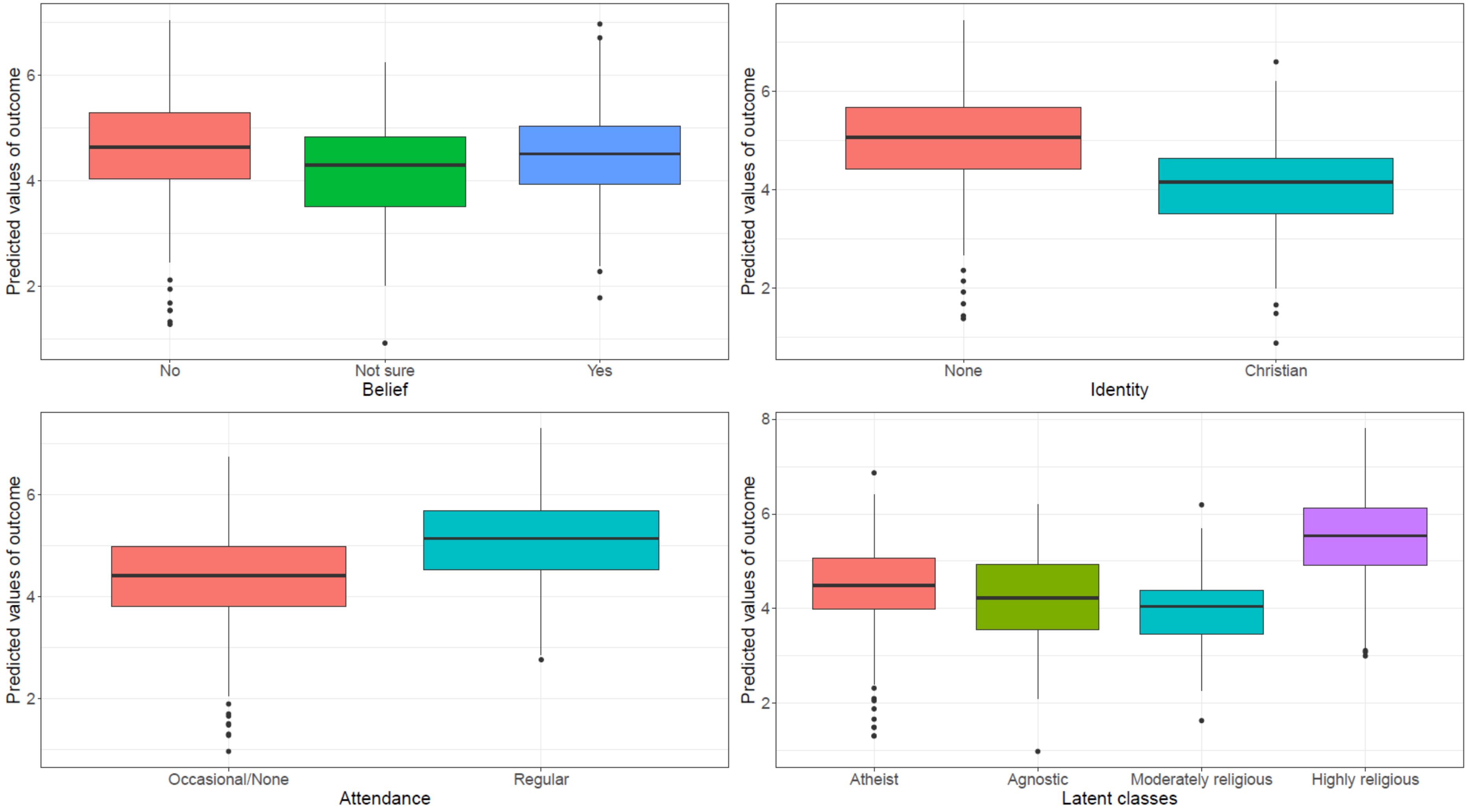

Figure S105: Predicted total number of actions (excluding ones which may be prohibitively costly) performed due to climate change for the religious identity (with the Christian denominations separated) as the exposure based on the partners Poisson regression models.

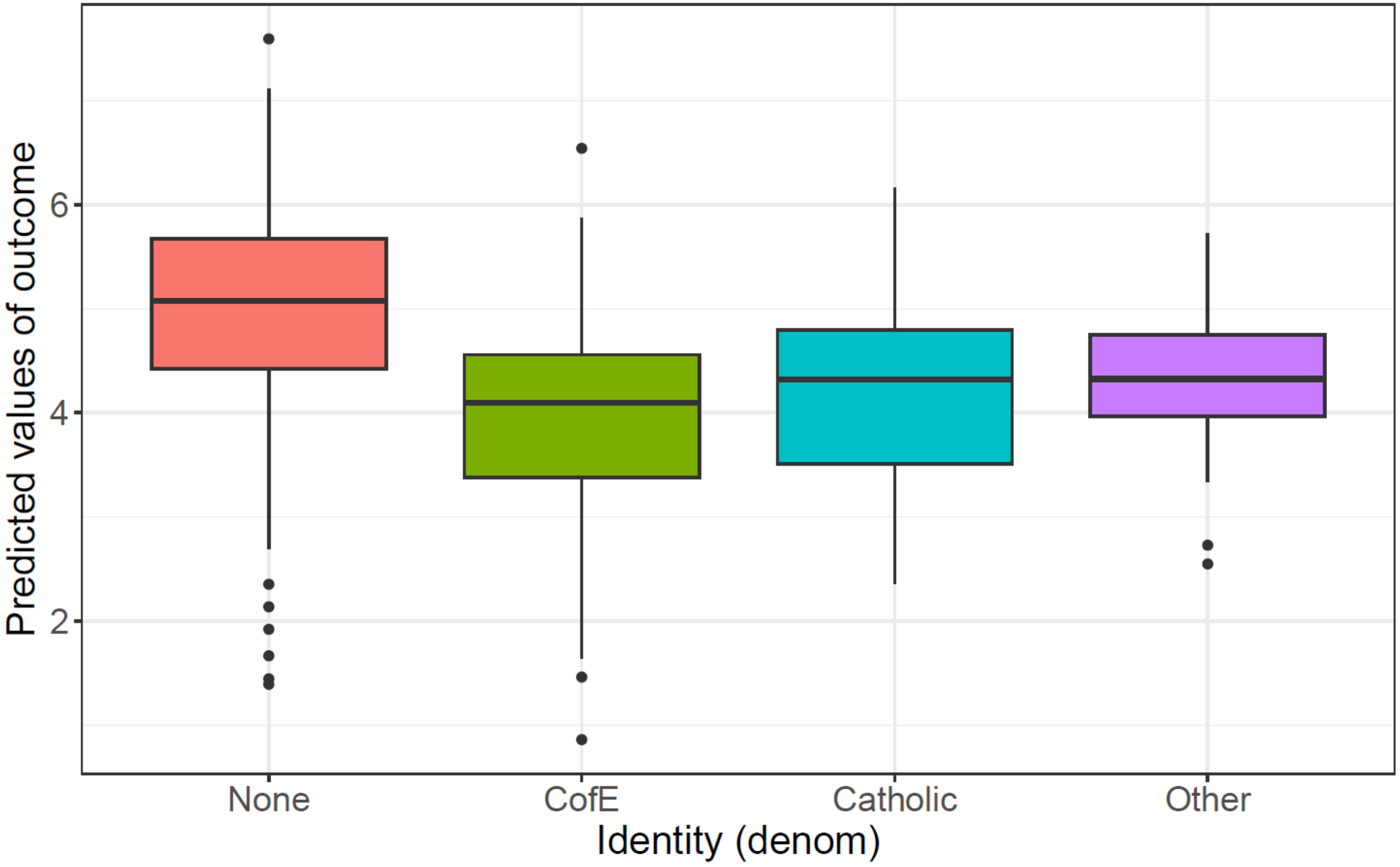

Figure S106: Results of the partners zero-inflated Poisson regression models with ‘total number of actions performed due to climate change (excluding ones which may be prohibitively costly)’ as the outcome for four religious exposures (belief [*n* = 1,002], identity [*n* = 993], attendance [*n* = 1,007], and latent classes [*n* = 1,011]; models are separated by dashed horizontal lines). Incidence rate ratios above 1 indicate an increased number of pro-environmental actions performed, while odds ratios above 1 indicate an excess of zeros. See table S30 for full results.

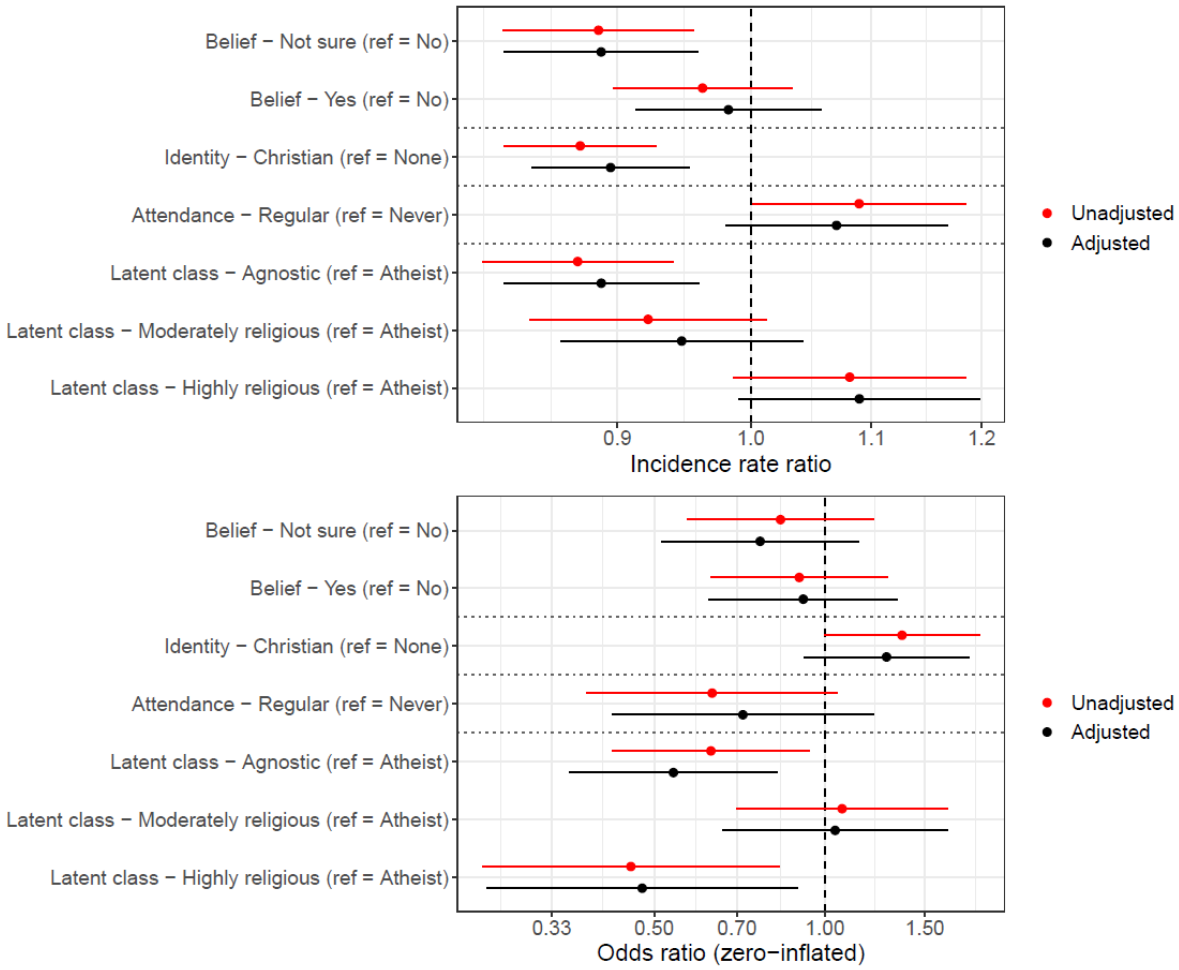

Figure S107: Predicted total number of actions (excluding ones which may be prohibitively costly) performed due to climate change for four religious exposures (belief, identity, attendance and latent classes) based on the partners zero-inflated Poisson regression models.

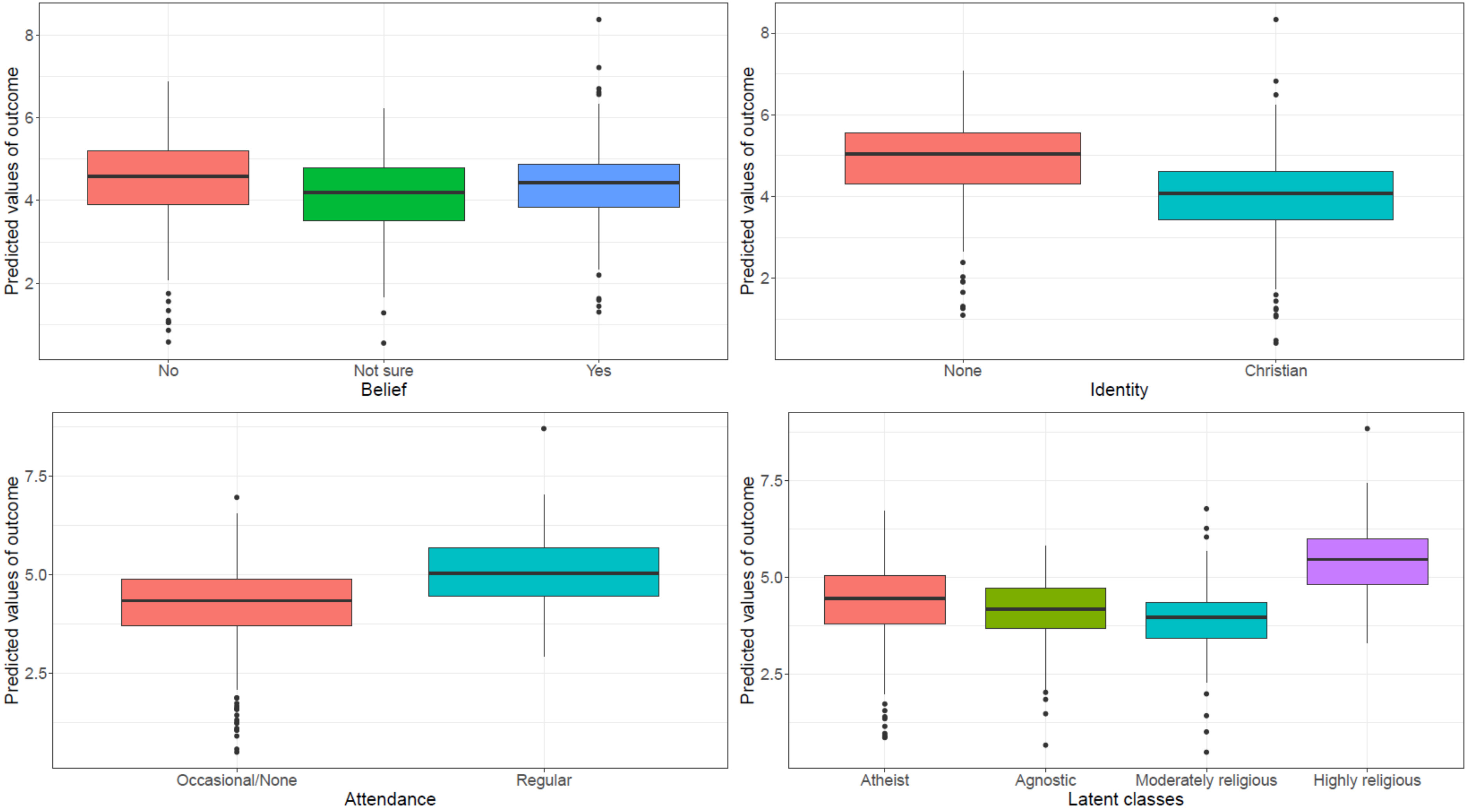

Figure S108: Predicted total number of actions (excluding ones which may be prohibitively costly) performed due to climate change for the religious identity (with the Christian denominations separated) as the exposure based on the partners zero-inflated Poisson regression models.

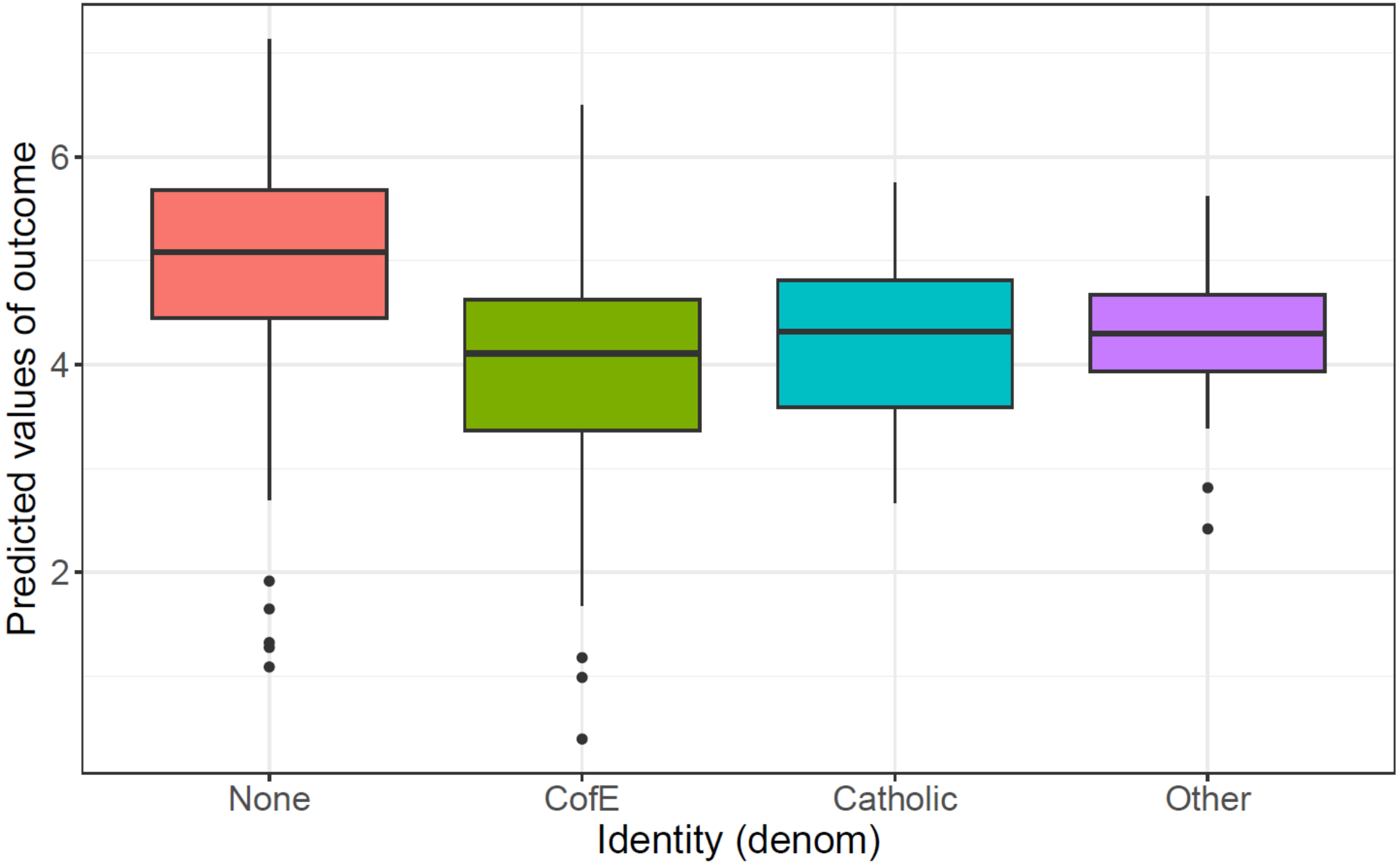

Figure S109: Results of the partners multinomial regression models with 'changed the way travel locally' as the outcome for four religious exposures (belief [*n* = 1,108], identity [*n* = 1,096], attendance [*n* = 1,110] and latent classes [*n* = 1,117]; models are separated by dashed horizontal lines). See table S31 for full results.

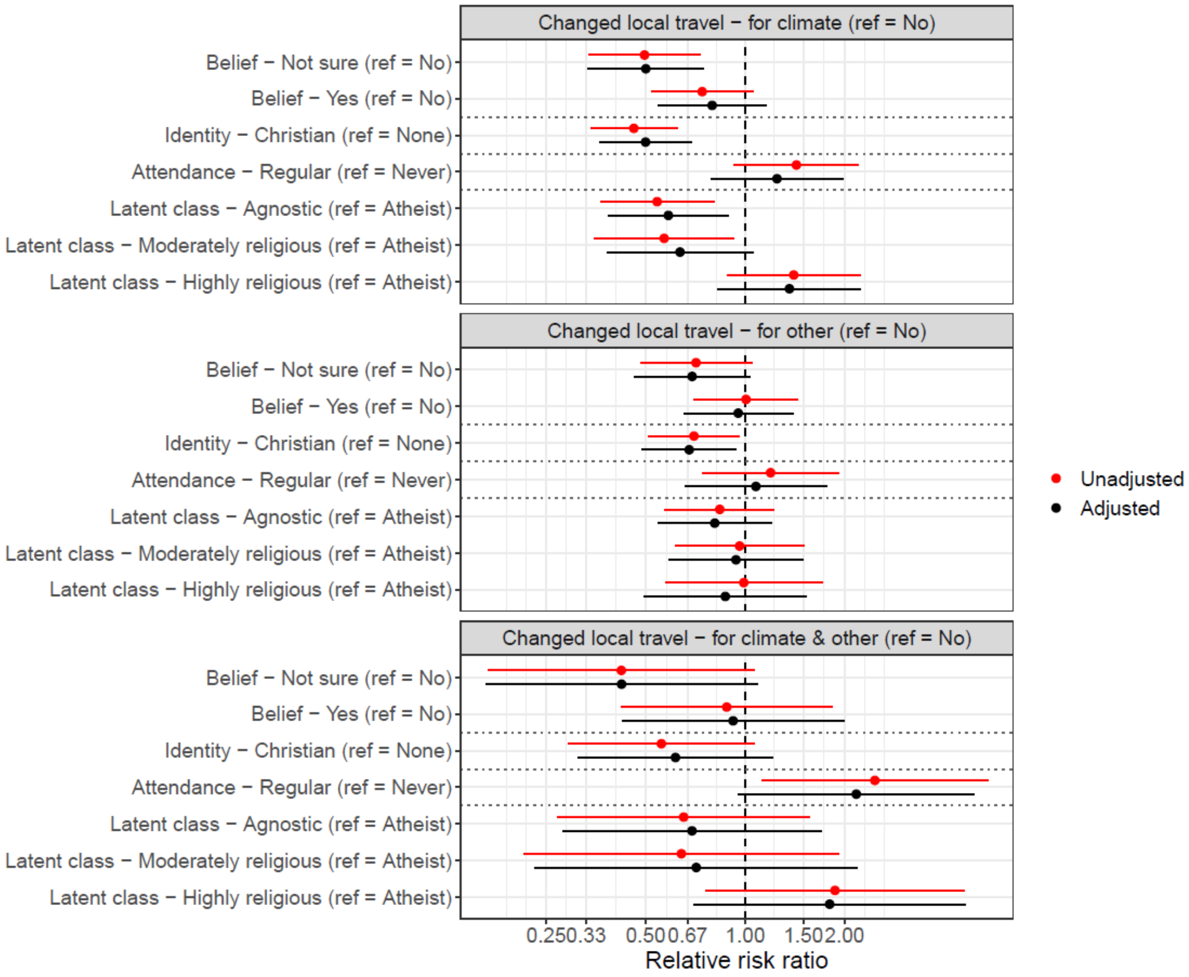

Figure S110: Predicted probabilities of the partners multinomial regression models with ‘changed the way travel locally’ as the outcome for four religious exposures (belief, identity, attendance and latent classes).

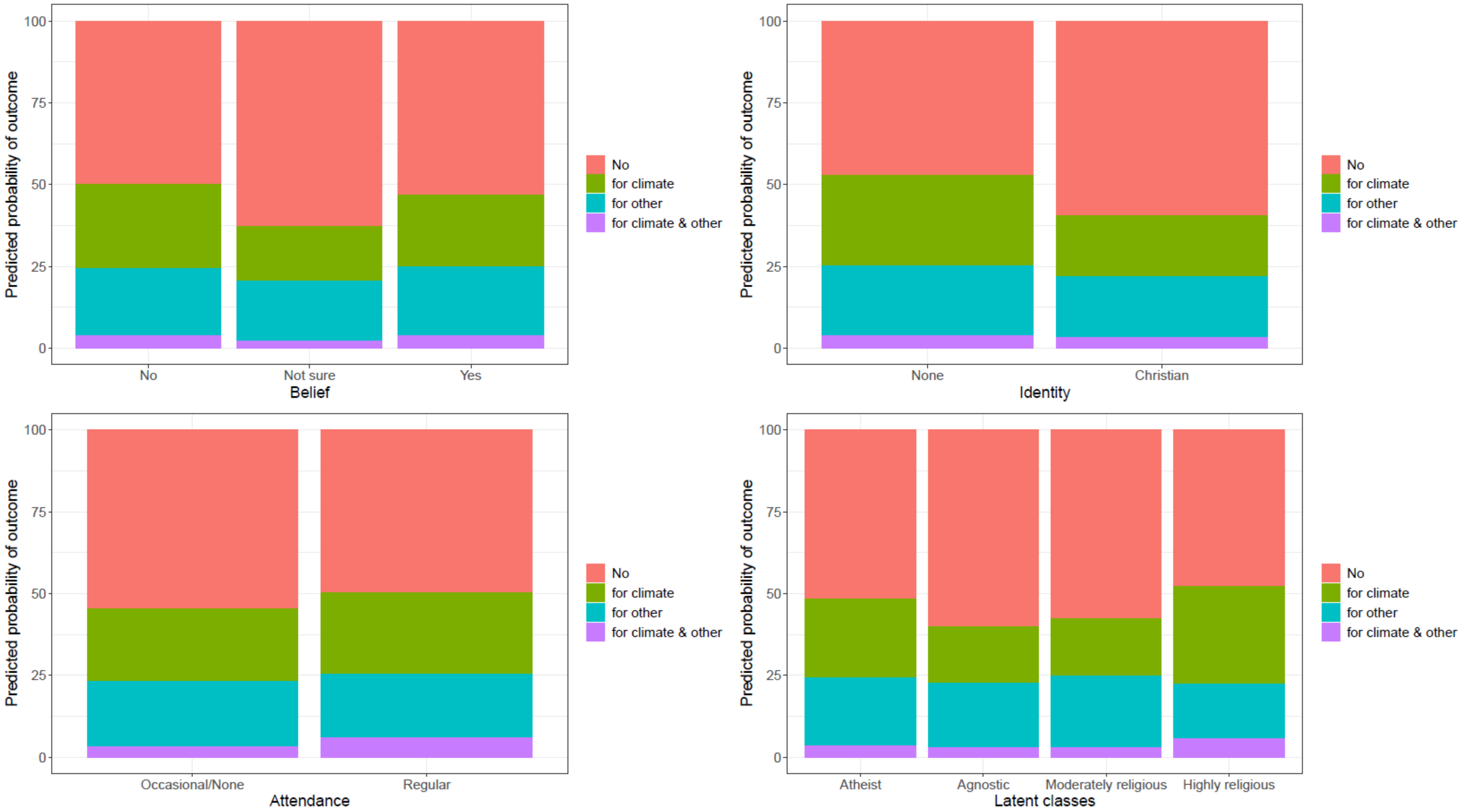

Figure S111: Predicted probabilities of the partners multinomial regression models with ‘changed the way travel locally’ as the outcome and the religious identity (with the Christian denominations separated) as the exposure.

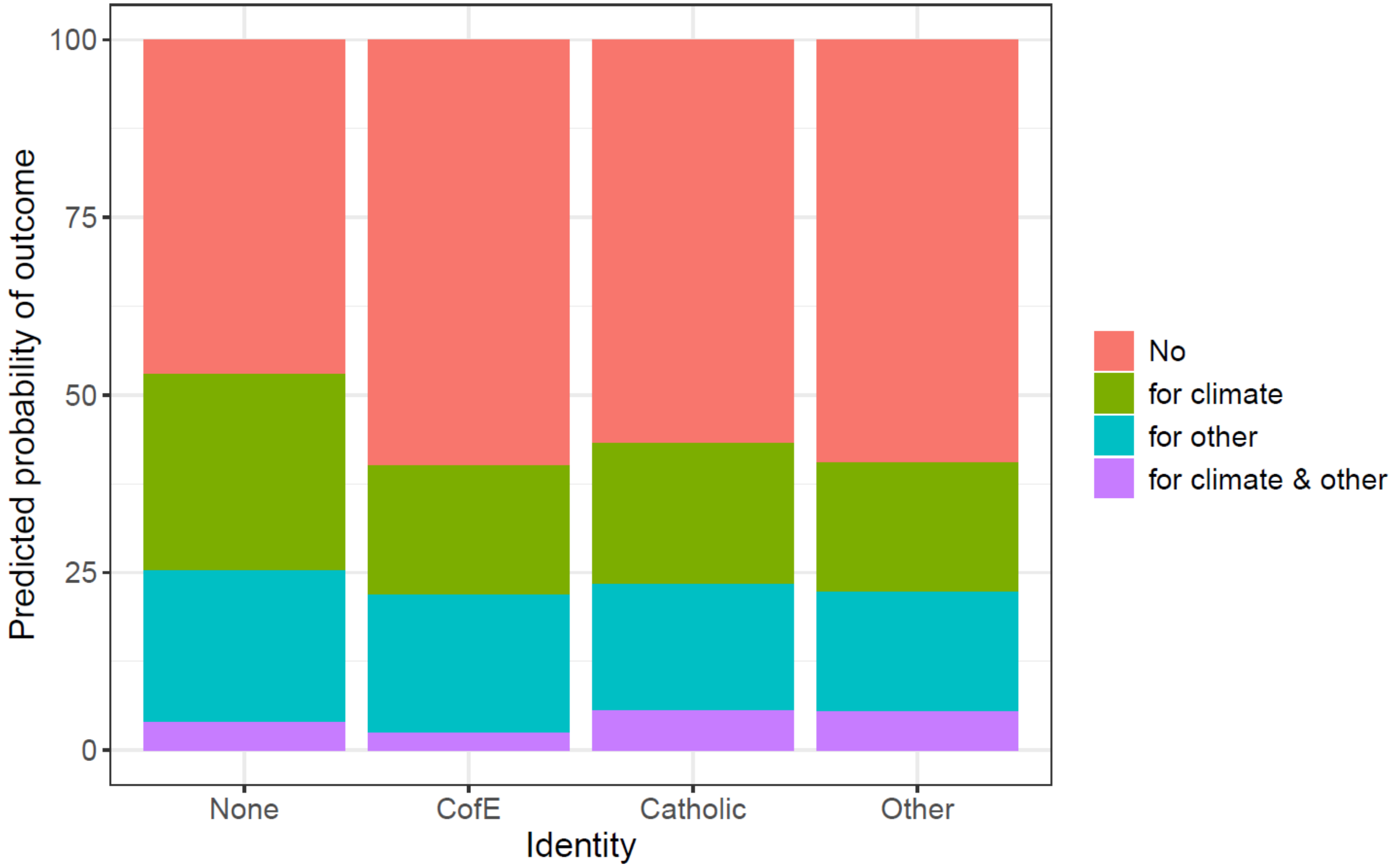

Figure S112: Results of the partners multinomial regression models with 'reduced household waste' as the outcome for four religious exposures (belief [*n* = 1,109], identity [*n* = 1,098], attendance [*n* = 1,111], and latent classes [*n* = 1,119]; models are separated by dashed horizontal lines). See table S31 for full results.

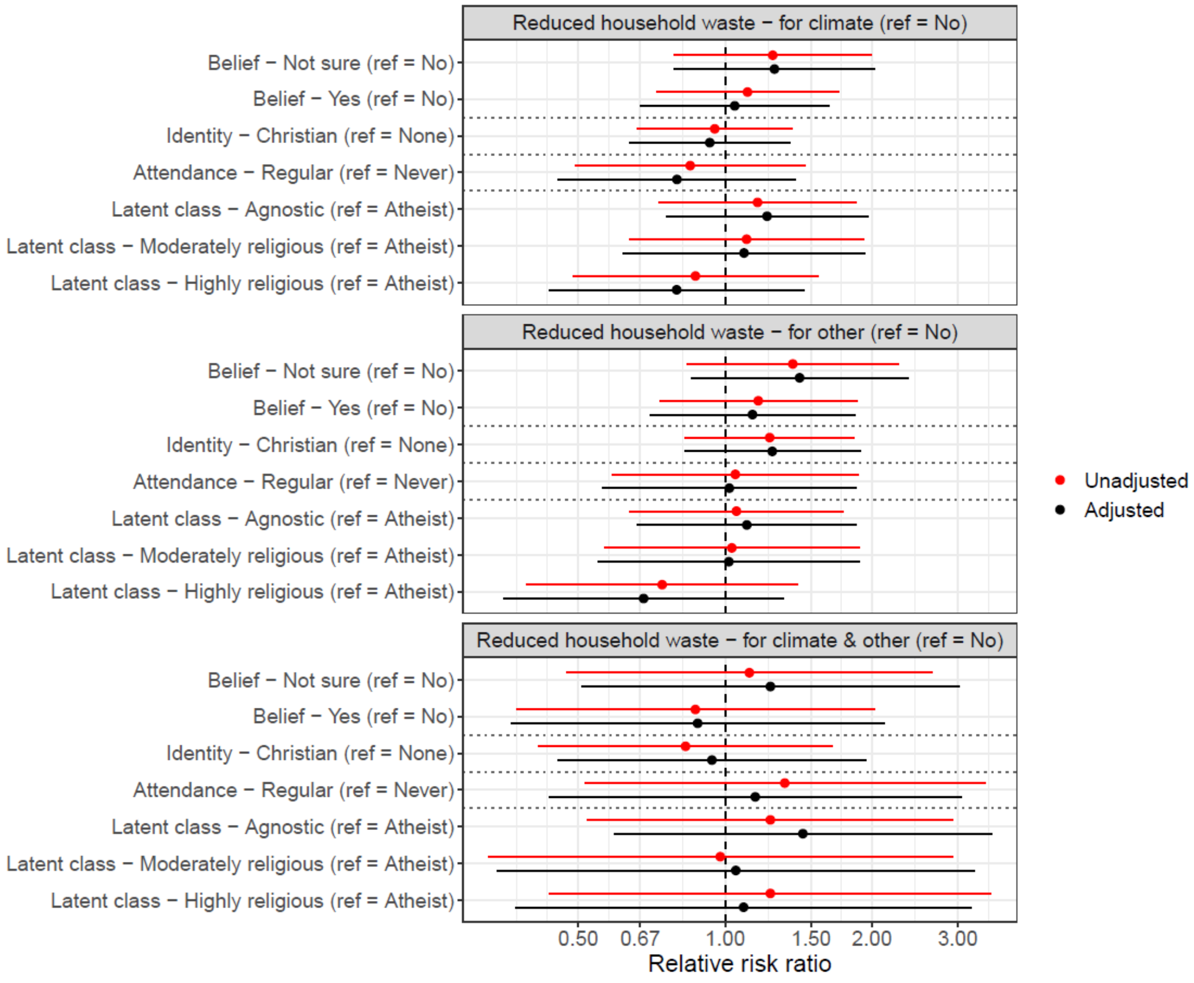

Figure S113: Predicted probabilities of the partners multinomial regression models with ‘reduced household waste’ as the outcome for four religious exposures (belief, identity, attendance and latent classes).

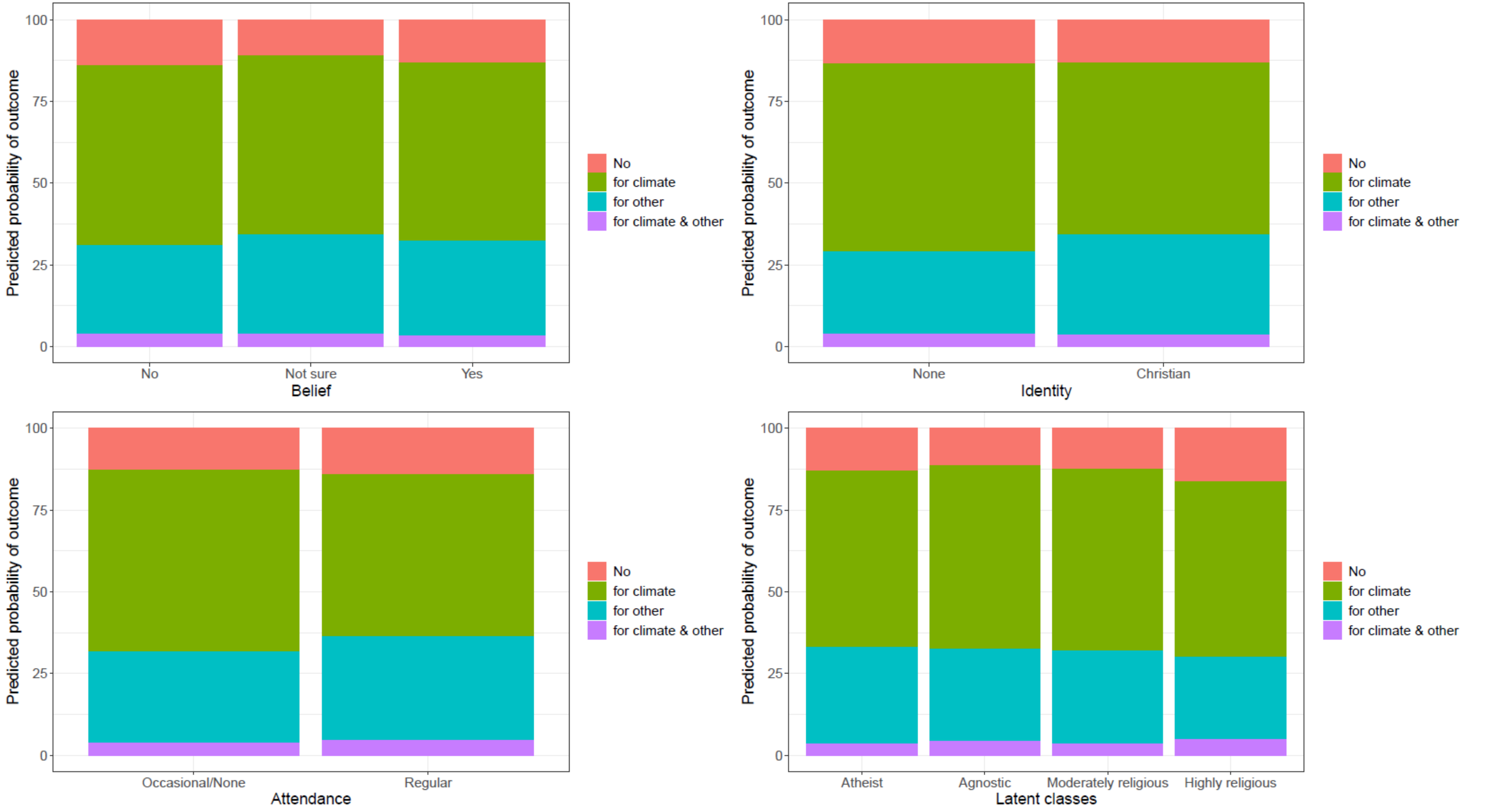

Figure S114: Predicted probabilities of the partners multinomial regression models with 'reduced household waste' as the outcome and the religious identity (with the Christian denominations separated) as the exposure.

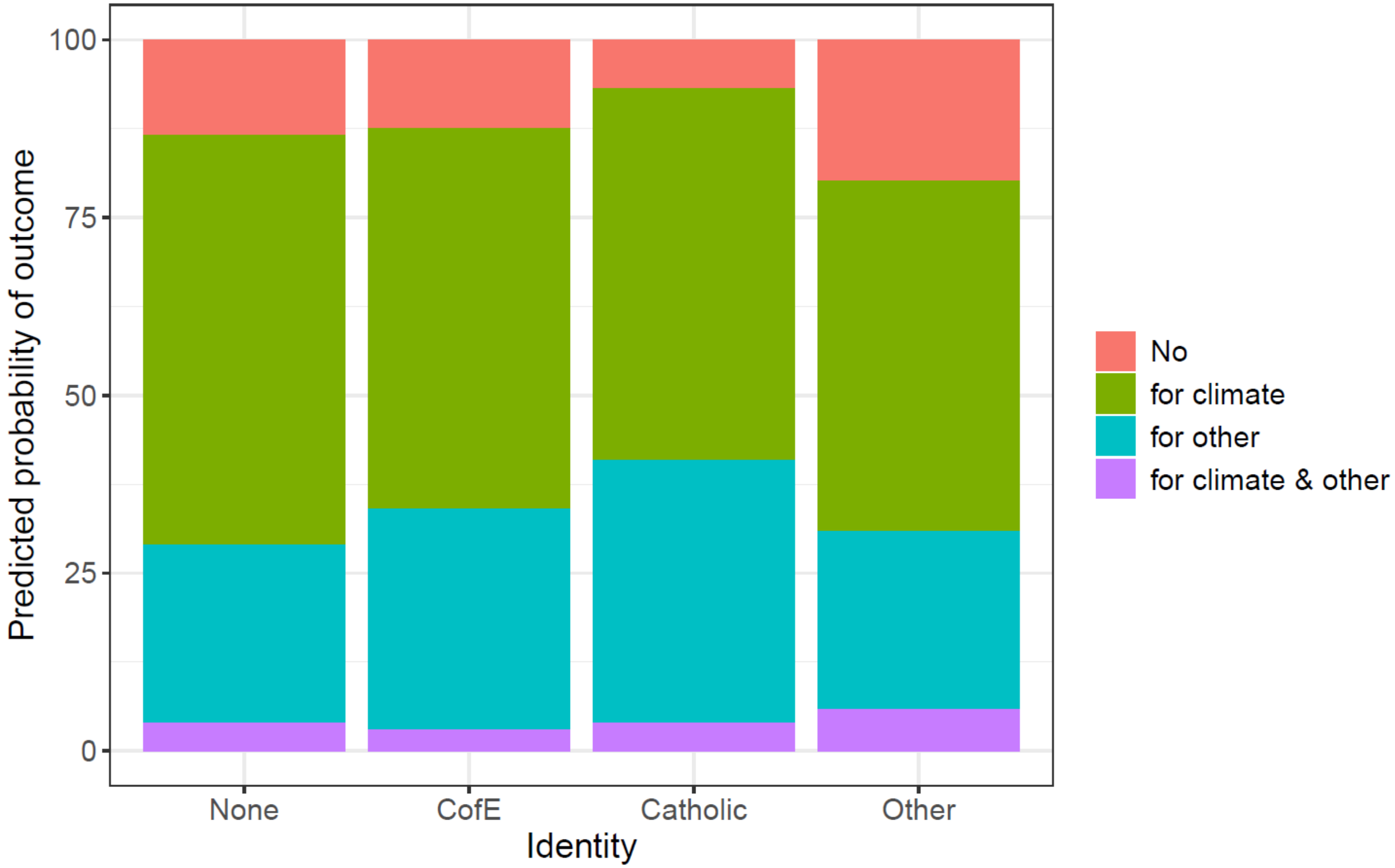

Figure S115: Results of the partners multinomial regression models with 'reduced energy use at home' as the outcome for four religious exposures (belief [*n* = 1,105], identity [*n* = 1,094], attendance [*n* = 1,107], and latent classes [*n* = 1,115]; models are separated by dashed horizontal lines). See table S31 for full results.

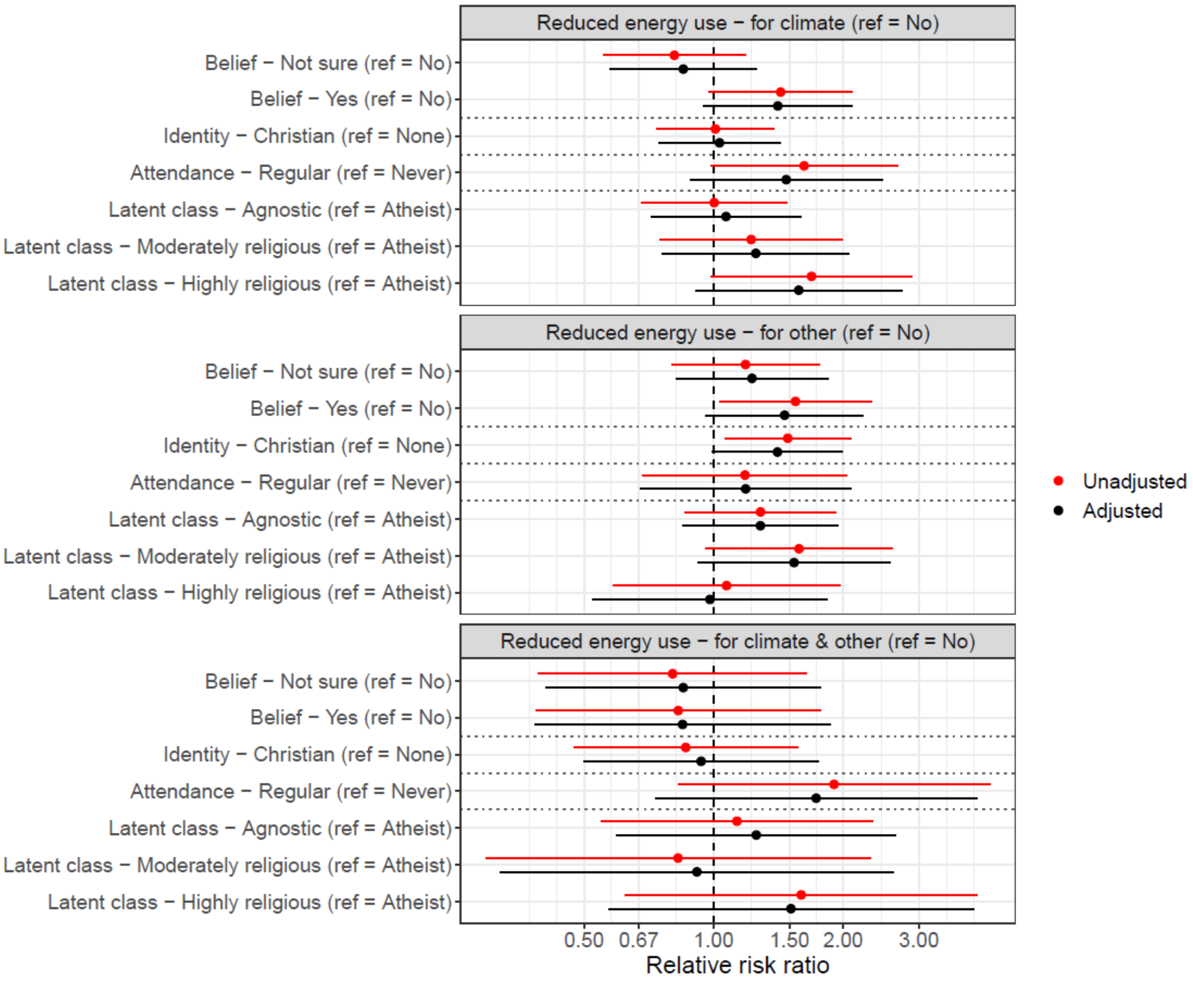

Figure S116: Predicted probabilities of the partners multinomial regression models with 'reduced energy use at home' as the outcome for four religious exposures (belief, identity, attendance and latent classes).

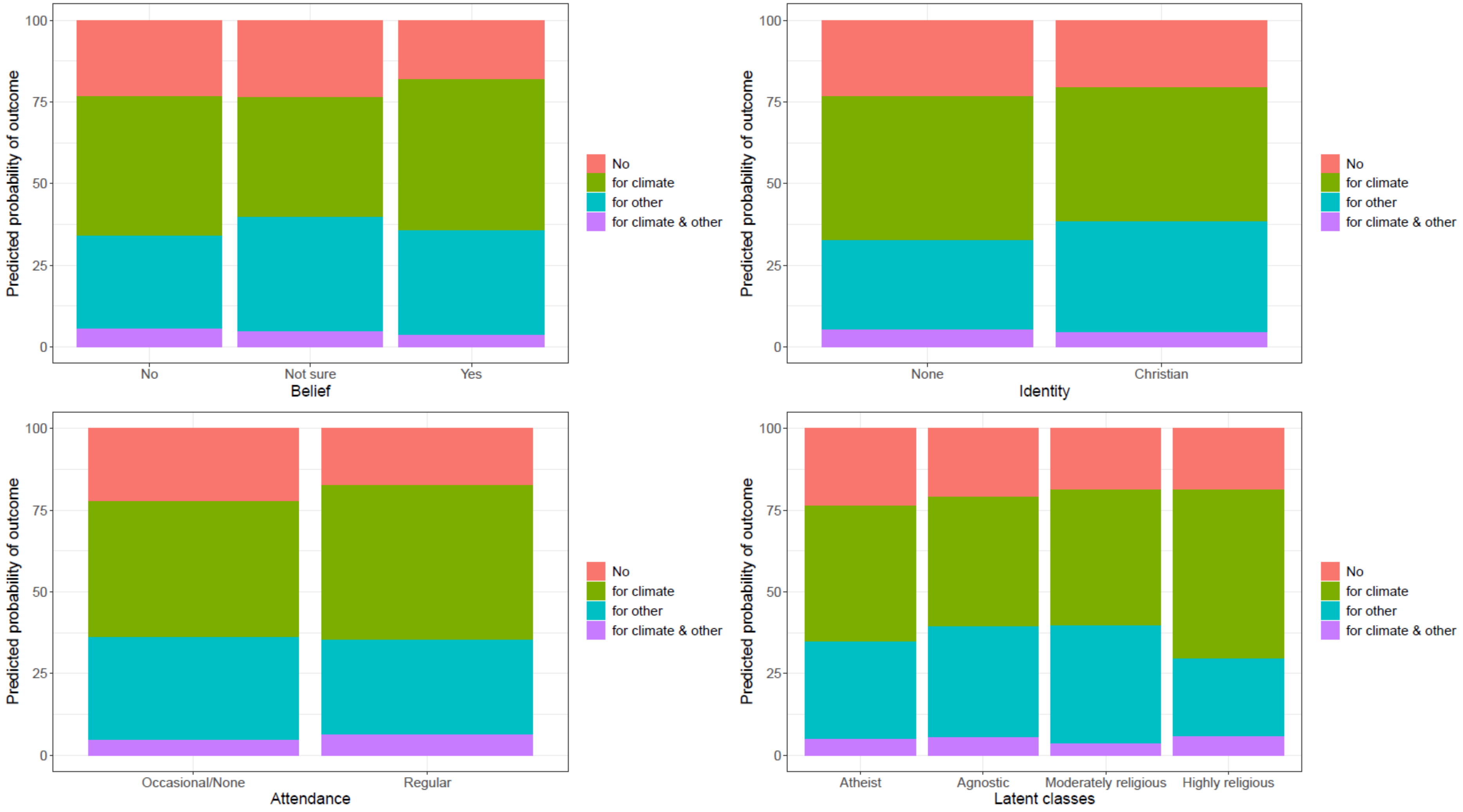

Figure S117: Predicted probabilities of the partners multinomial regression models with 'reduced energy use at home' as the outcome and the religious identity (with the Christian denominations separated) as the exposure.

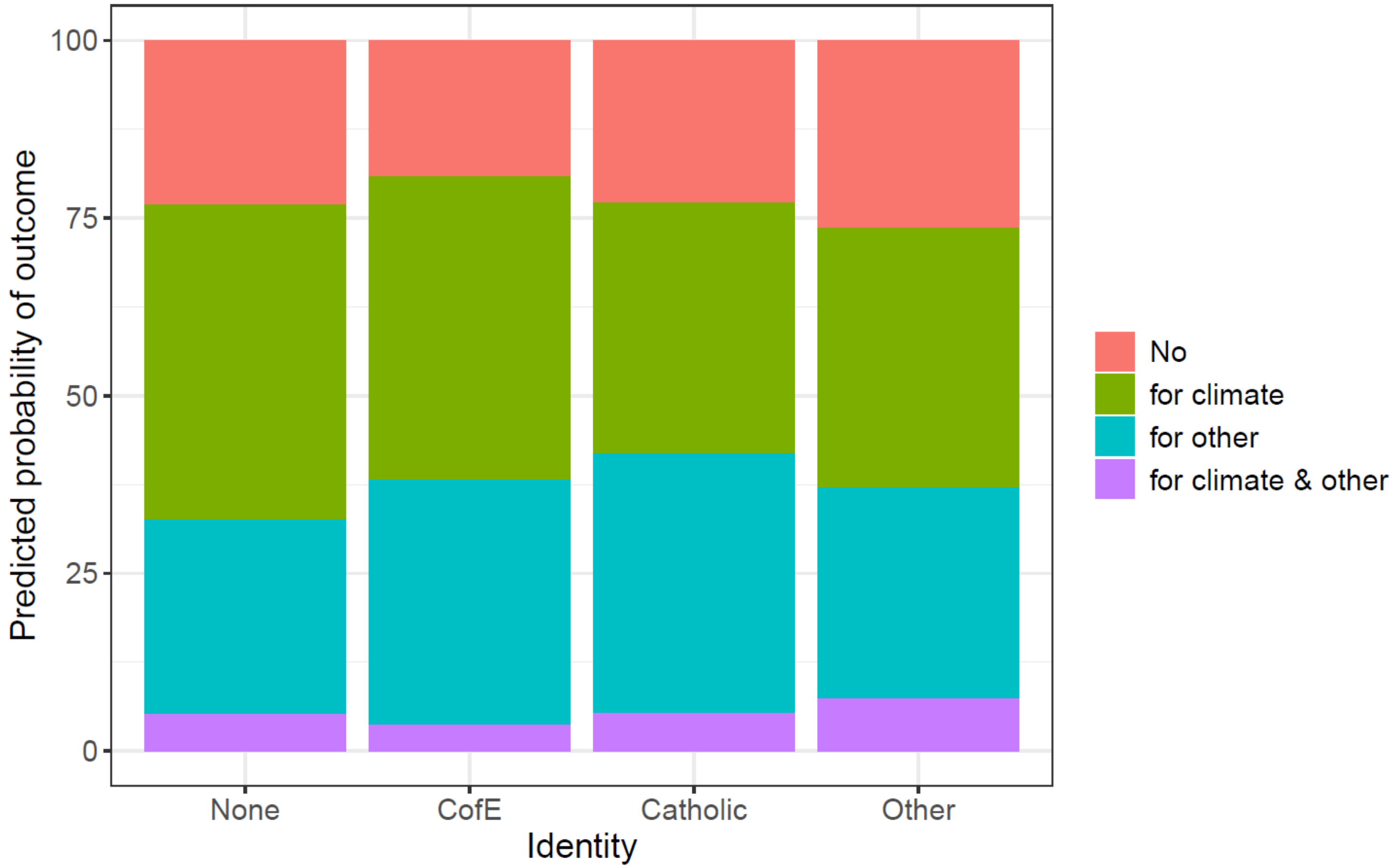

Figure S118: Results of the partners multinomial regression models with ‘changed what buy’ as the outcome for four religious exposures (belief [*n* = 1,101], identity [*n* = 1,090], attendance [*n* = 1,103], and latent classes [*n* = 1,111]; models are separated by dashed horizontal lines). See table S31 for full results.

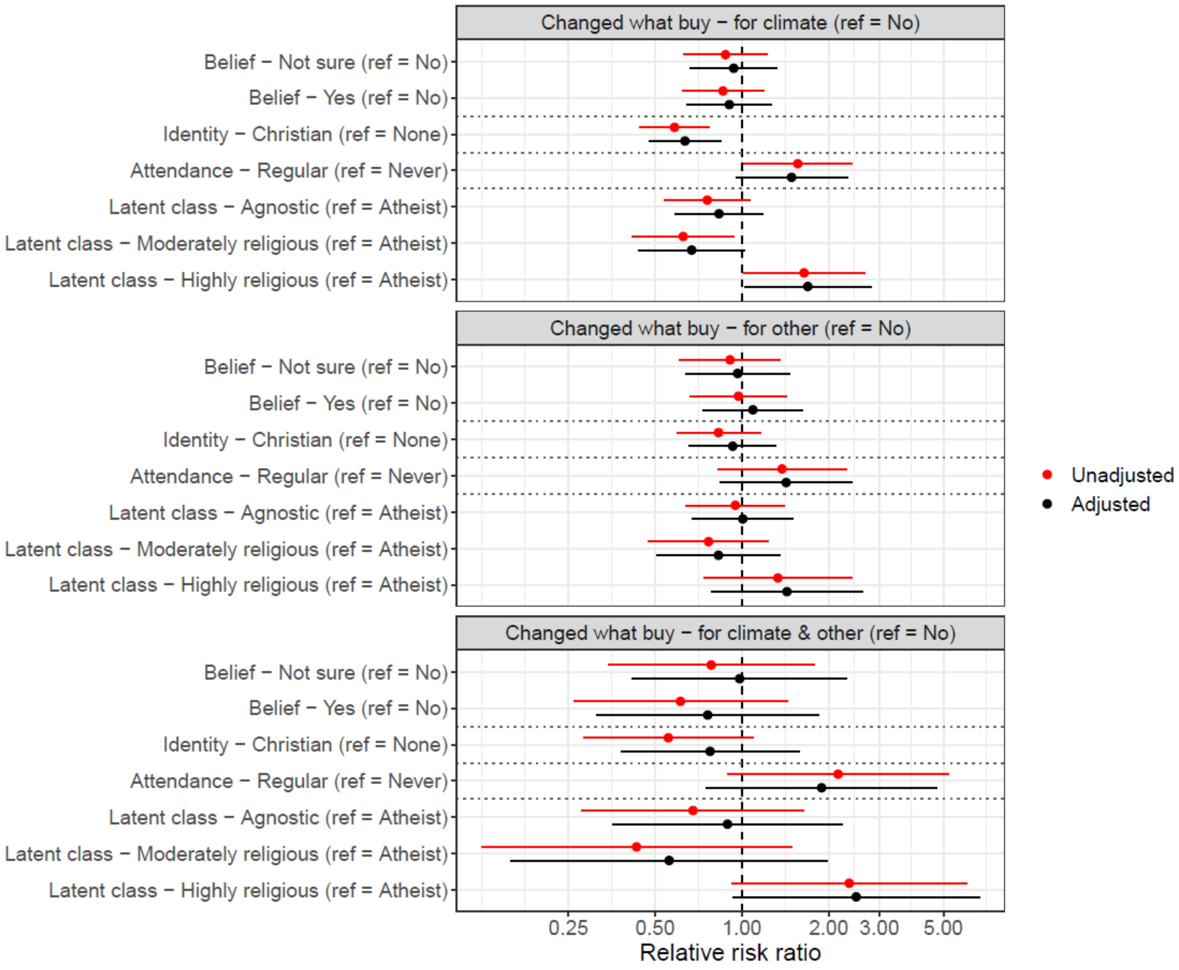

Figure S119: Predicted probabilities of the partners multinomial regression models with ‘changed what buy’ as the outcome for four religious exposures (belief, identity, attendance and latent classes).

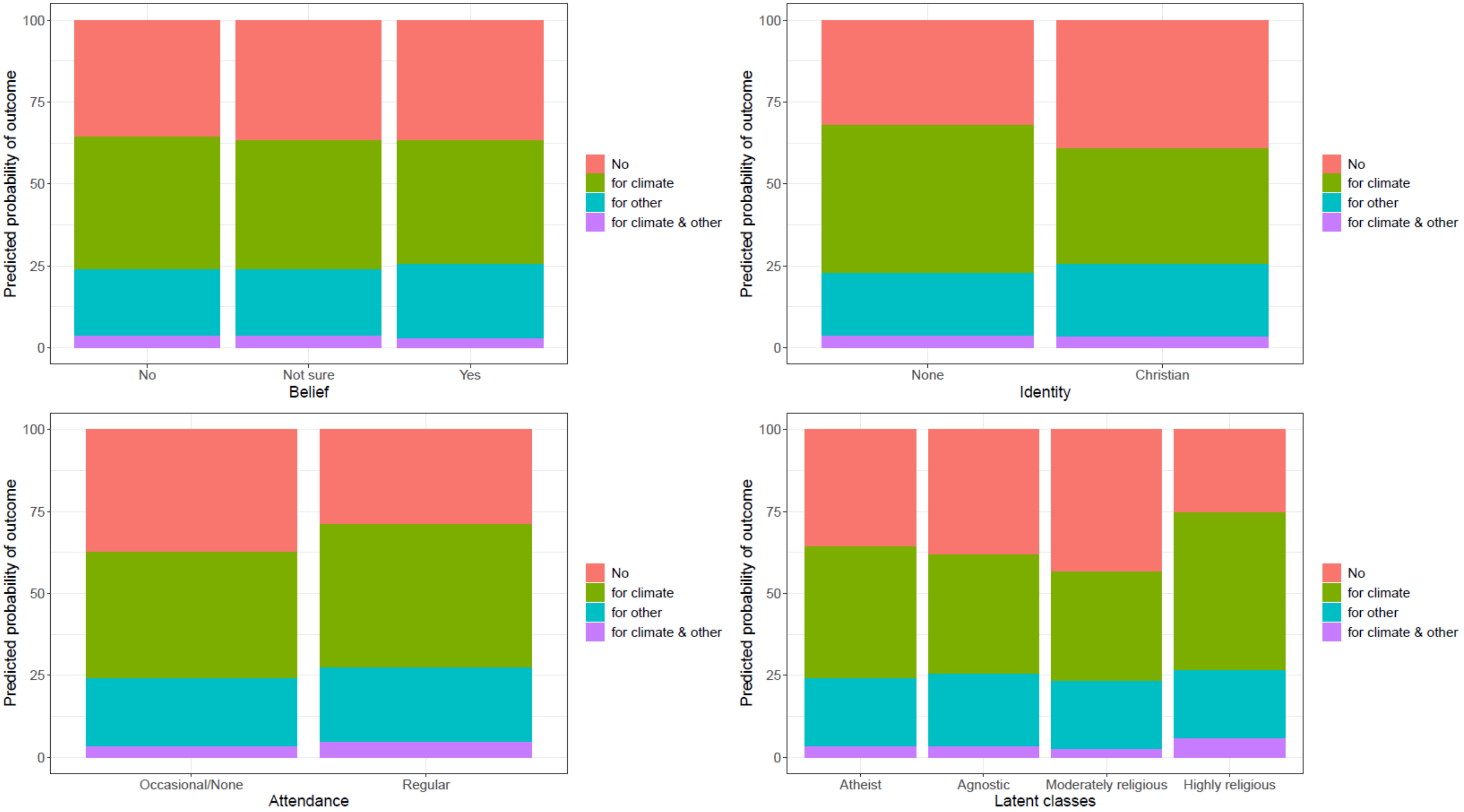

Figure S120: Predicted probabilities of the partners multinomial regression models with 'changed what buy' as the outcome and the religious identity (with the Christian denominations separated) as the exposure.

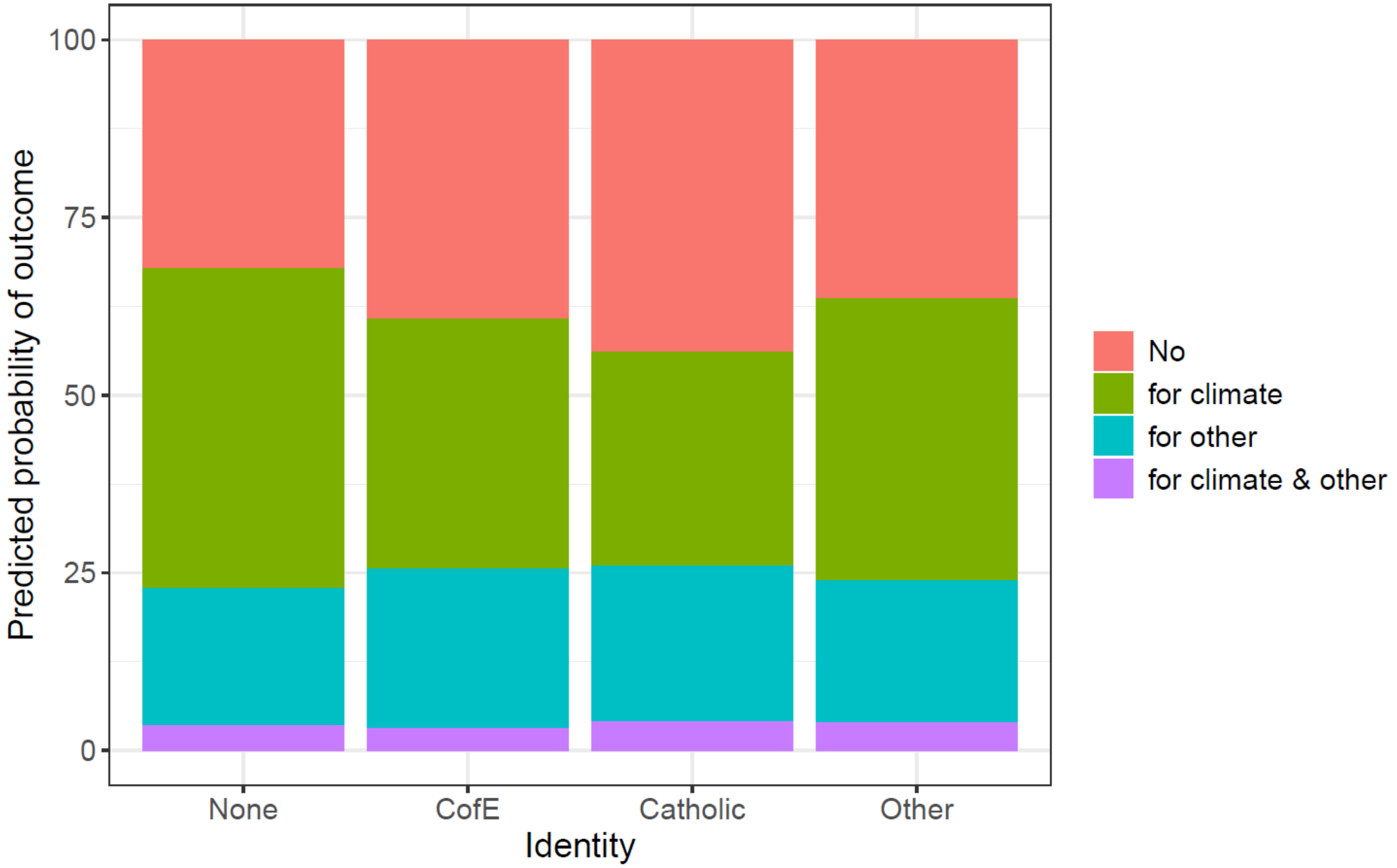

Figure S121: Results of the partners multinomial regression models with ‘reduced air travel’ as the outcome for four religious exposures (belief [*n* = 1,103], identity [*n* = 1,092], attendance [*n* = 1,105], and latent classes [*n* = 1,113]; models are separated by dashed horizontal lines). See table S31 for full results.

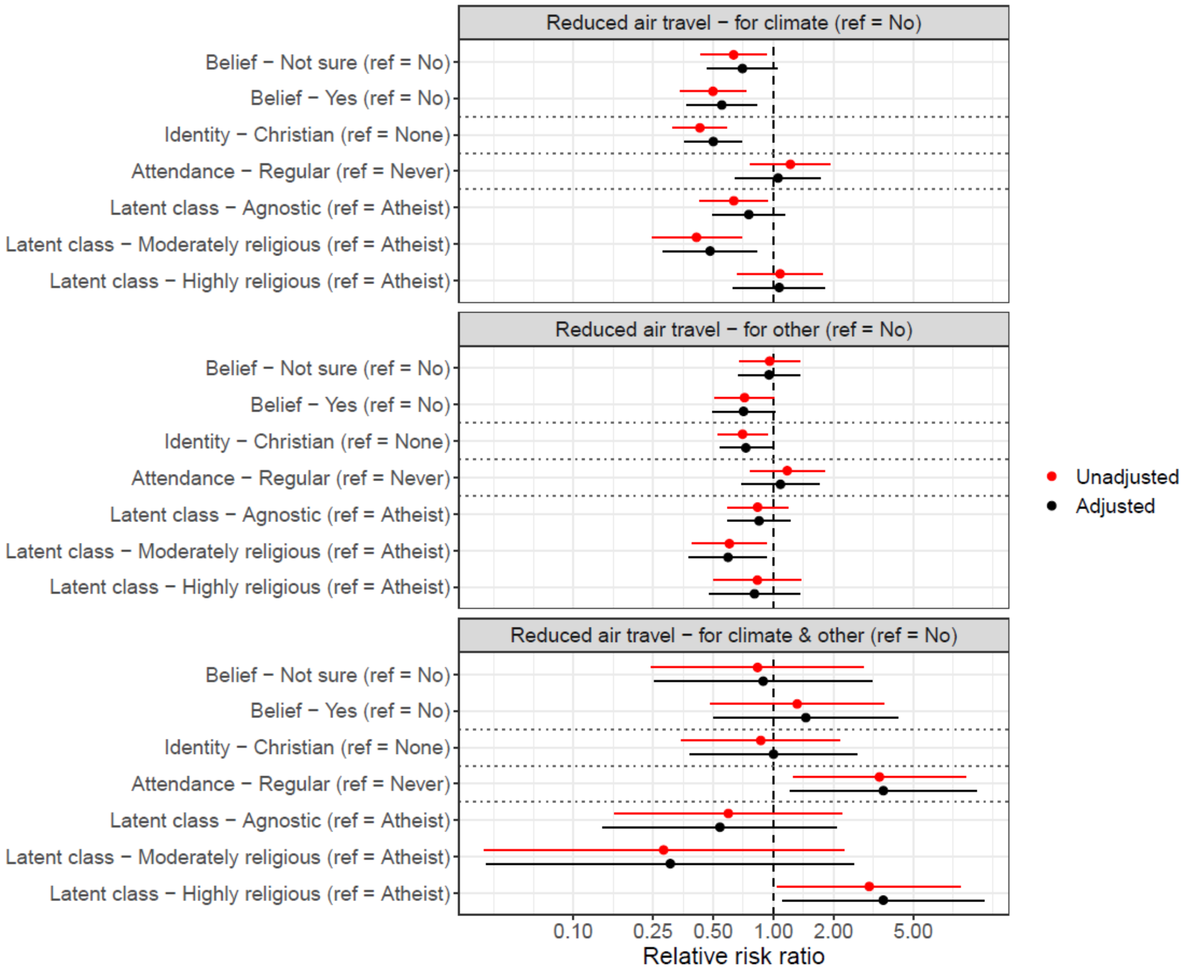

Figure S122: Predicted probabilities of the partners multinomial regression models with 'reduced air travel' as the outcome for four religious exposures (belief, identity, attendance and latent classes).

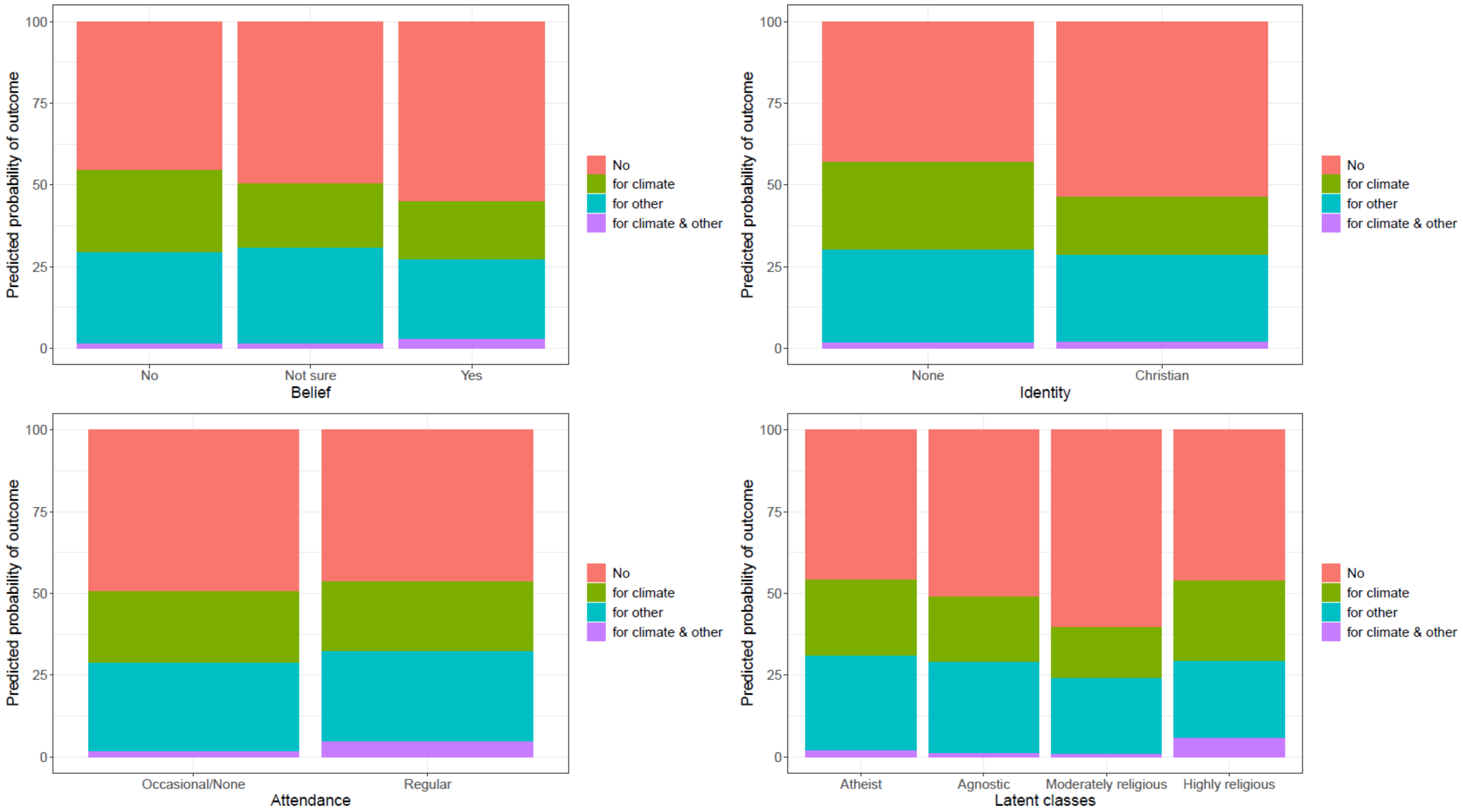

Figure S123: Predicted probabilities of the partners multinomial regression models with 'reduced air travel' as the outcome and the religious identity (with the Christian denominations separated) as the exposure.

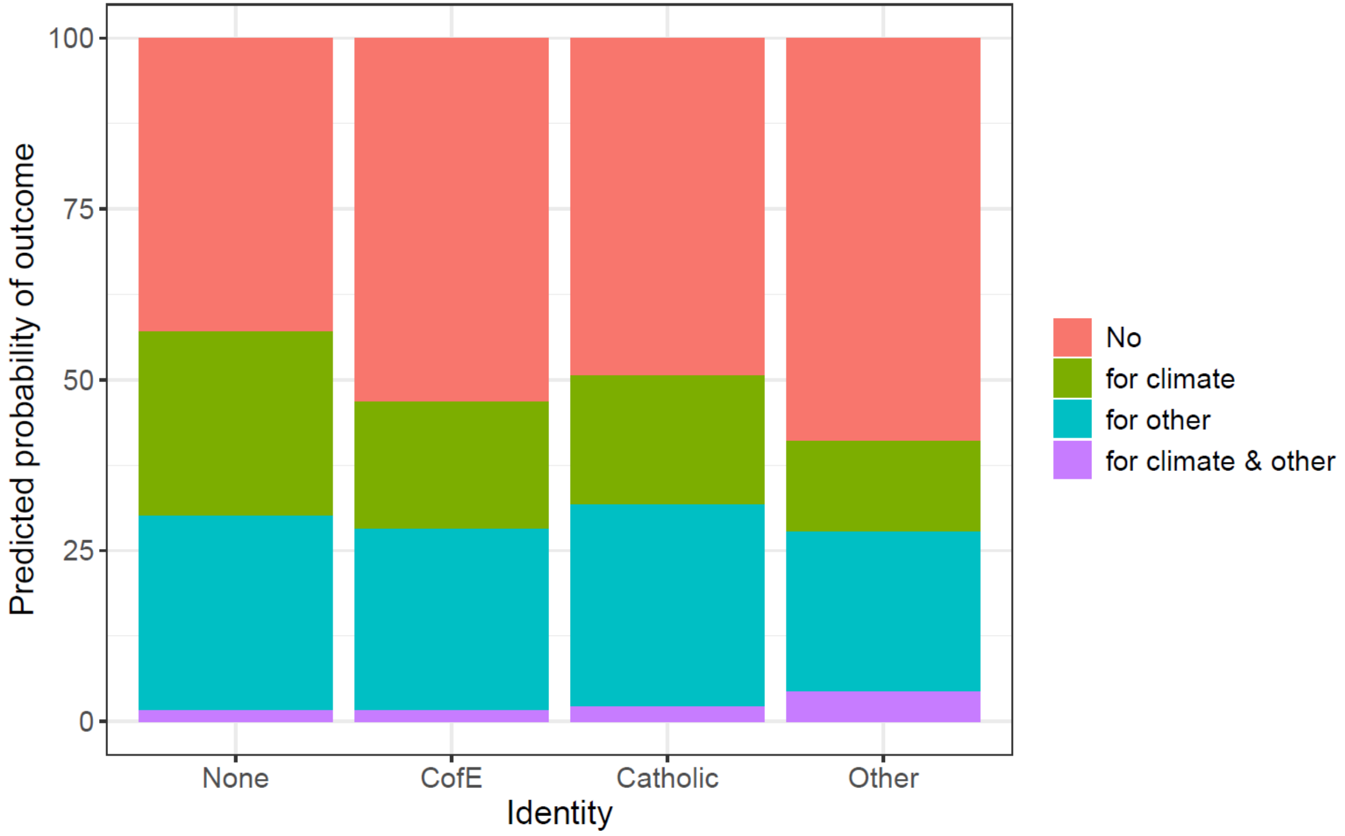

Figure S124: Results of the partners multinomial regression models with 'bought or hired an electric or hybrid vehicle' as the outcome for four religious exposures (belief [*n* = 1,107], identity [*n* = 1,096], attendance [*n* = 1,109], and latent classes [*n* = 1,117]; models are separated by dashed horizontal lines). See table S31 for full results.

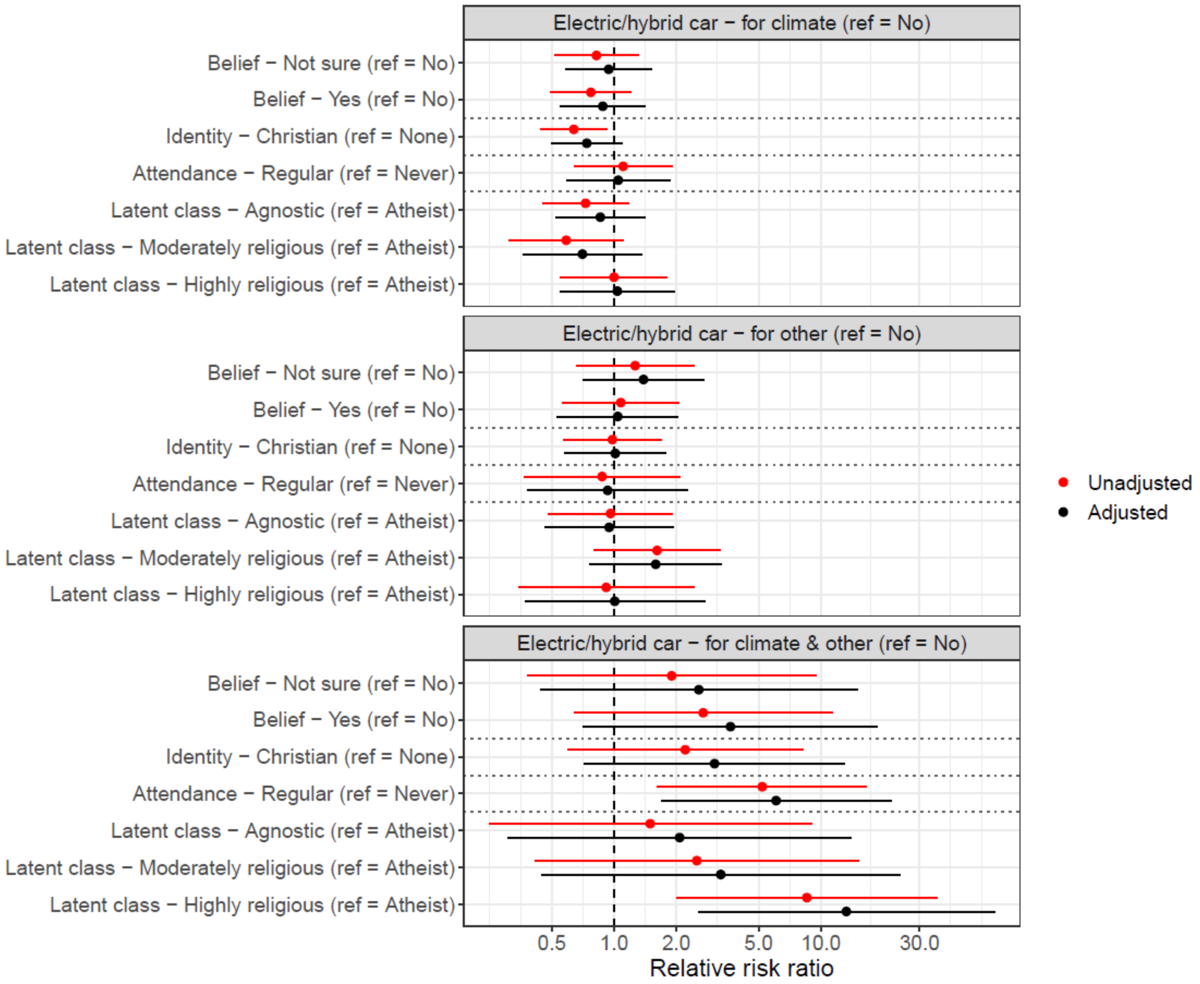

Figure S125: Predicted probabilities of the partners multinomial regression models with ‘bought or hired an electric or hybrid vehicle’ as the outcome for four religious exposures (belief, identity, attendance and latent classes).

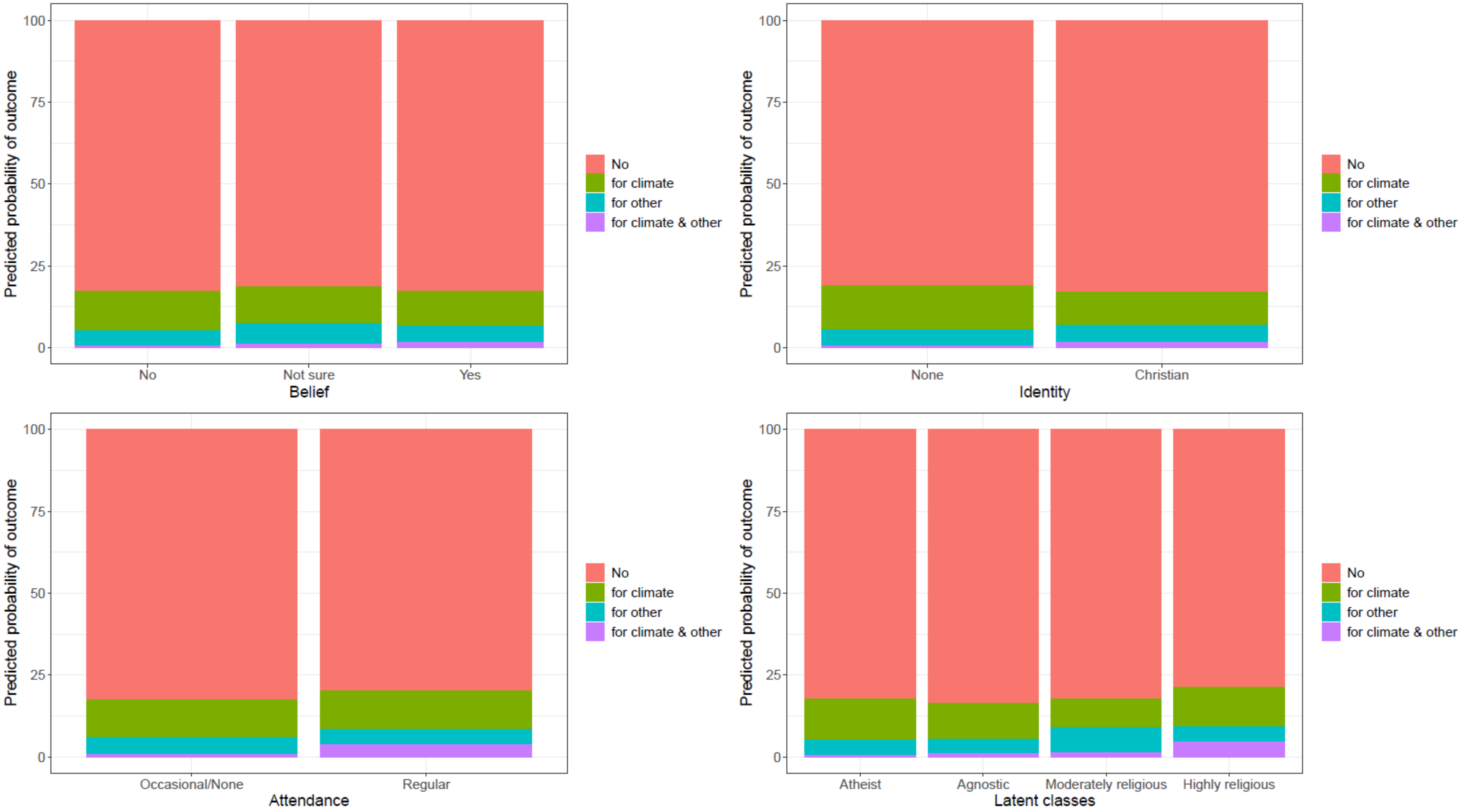

Figure S126: Predicted probabilities of the partners multinomial regression models with ‘bought or hired an electric or hybrid vehicle’ as the outcome and the religious identity (with the Christian denominations separated) as the exposure.

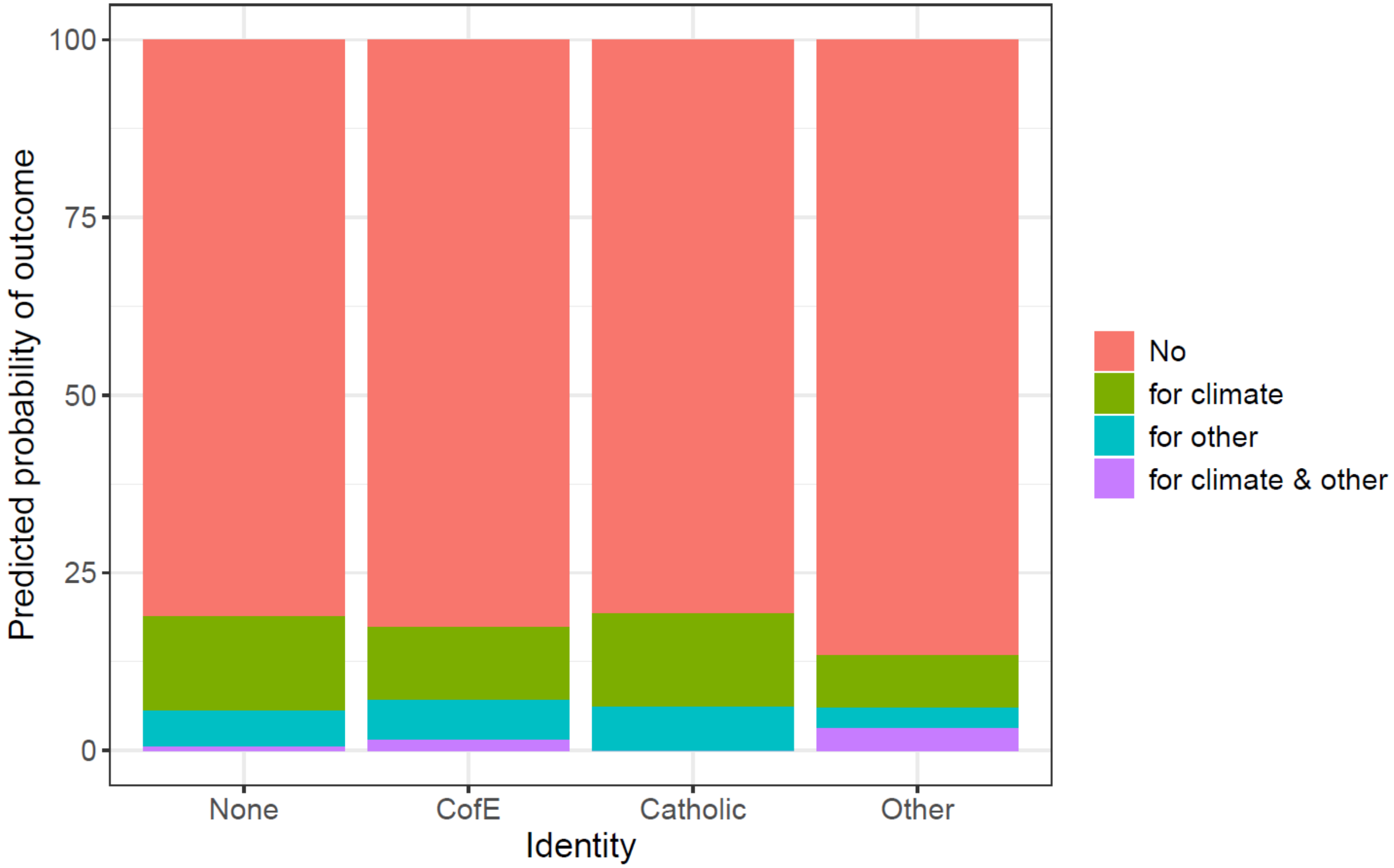

Figure S127: Results of the partners multinomial regression models with 'bought foods produced locally' as the outcome for four religious exposures (belief [*n* = 1,108], identity [*n* = 1,096], attendance [*n* = 1,110], and latent classes [*n* = 1,118]; models are separated by dashed horizontal lines). See table S31 for full results.

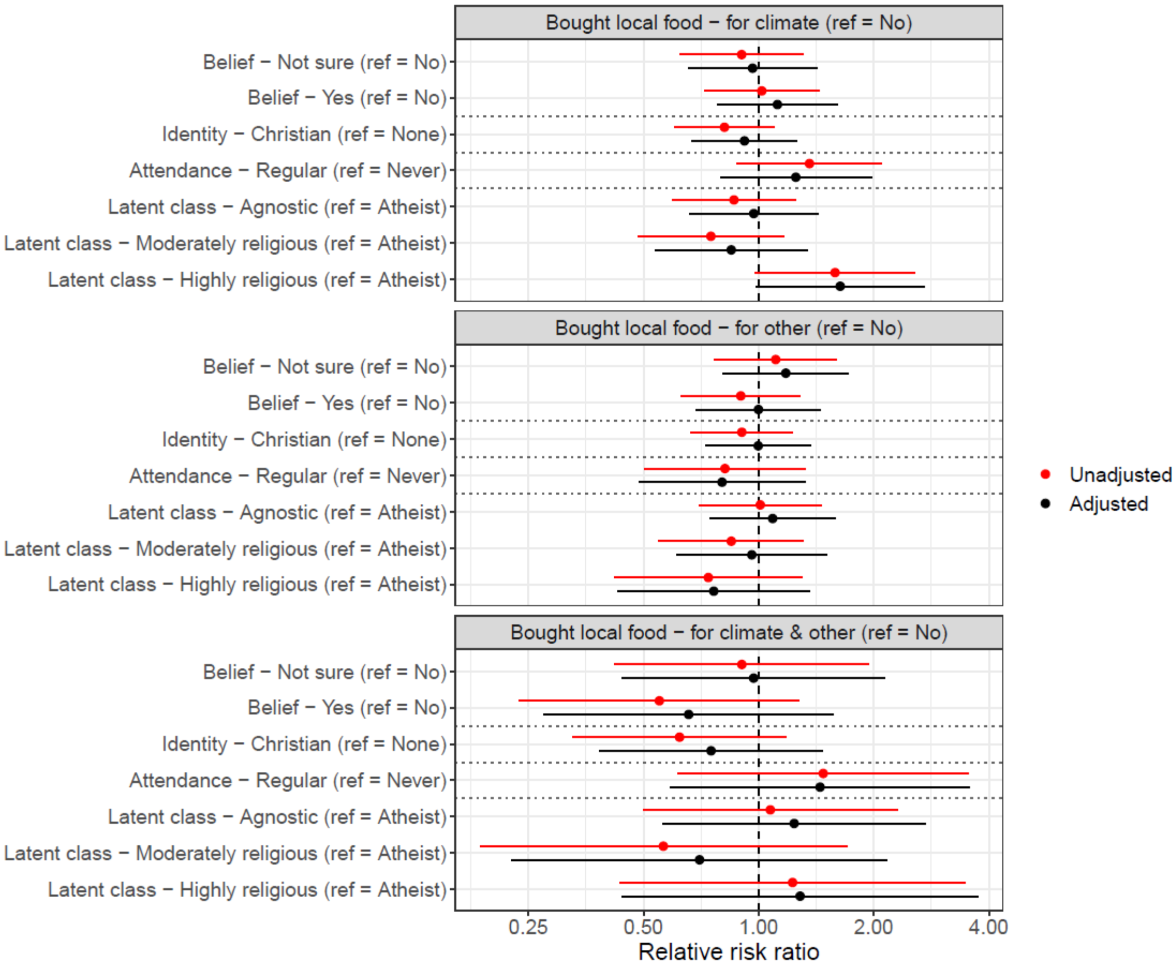

Figure S128: Predicted probabilities of the partners multinomial regression models with 'bought foods produced locally' as the outcome for four religious exposures (belief, identity, attendance and latent classes).

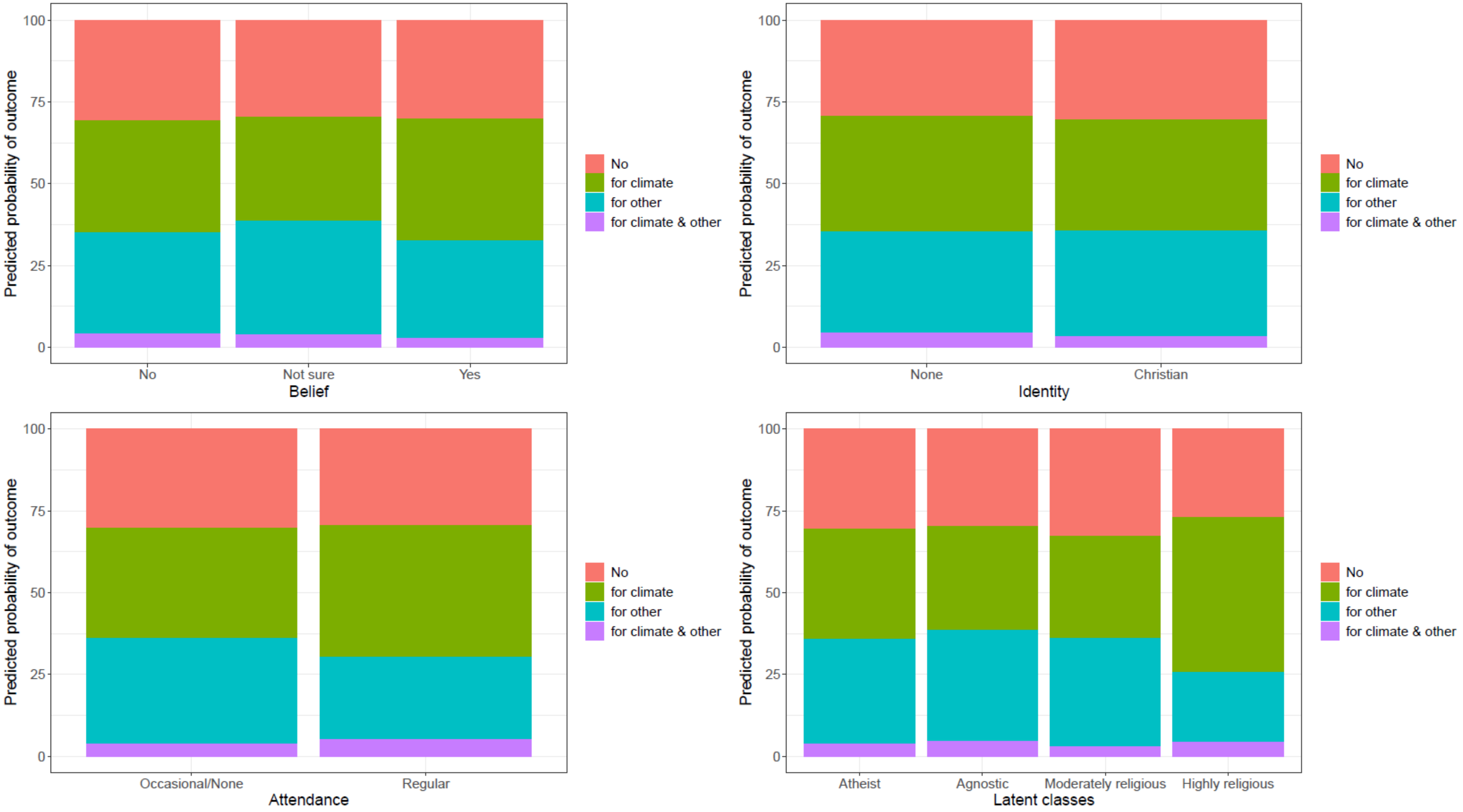

Figure S129: Predicted probabilities of the partners multinomial regression models with 'bought foods produced locally' as the outcome and the religious identity (with the Christian denominations separated) as the exposure.

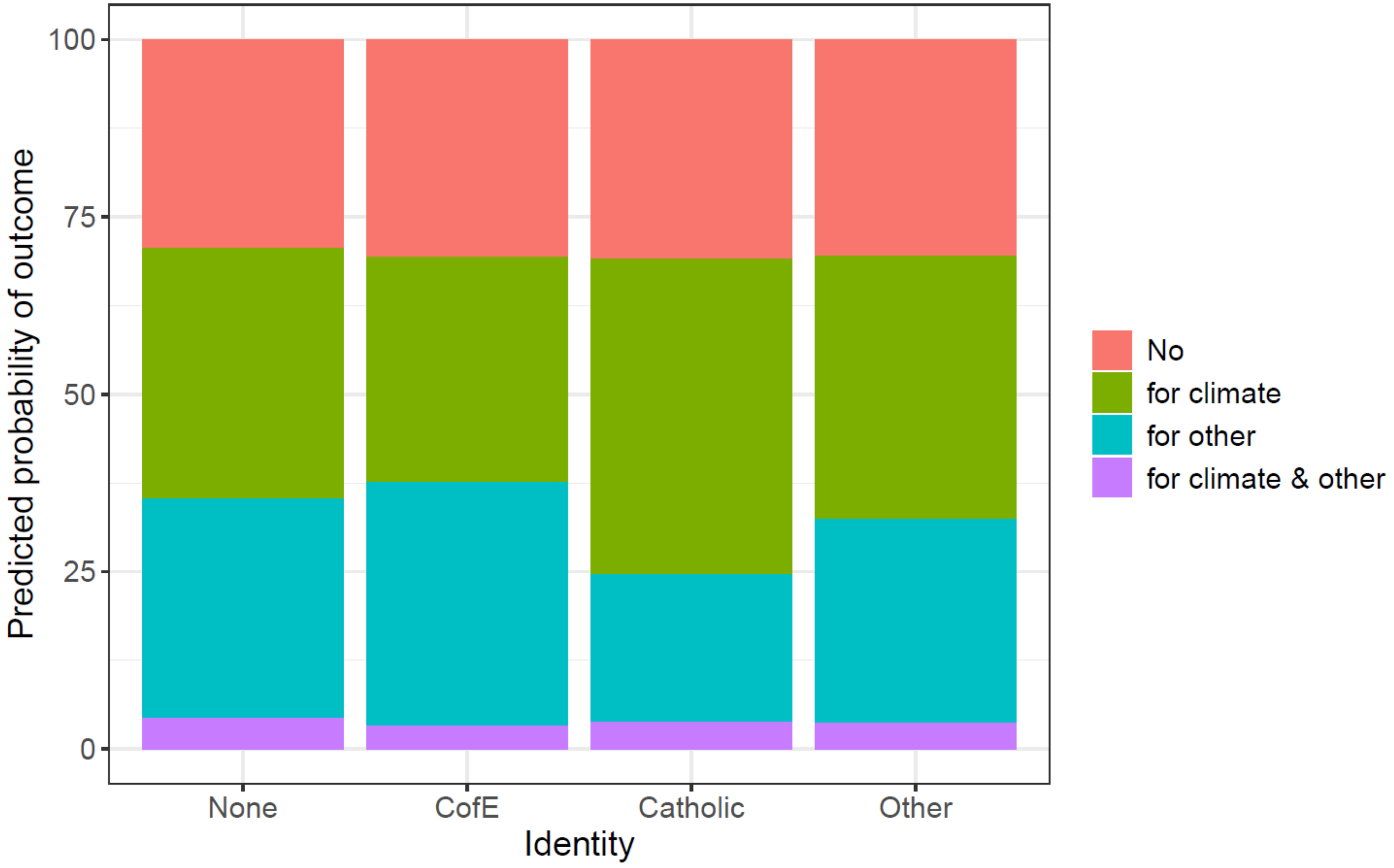

Figure S130: Results of the partners multinomial regression models with 'recycled or upcycled more' as the outcome for four religious exposures (belief [*n* = 1,107], identity [*n* = 1,096], attendance [*n* = 1,109], and latent classes [*n* = 1,117]; models are separated by dashed horizontal lines). See table S31 for full results.

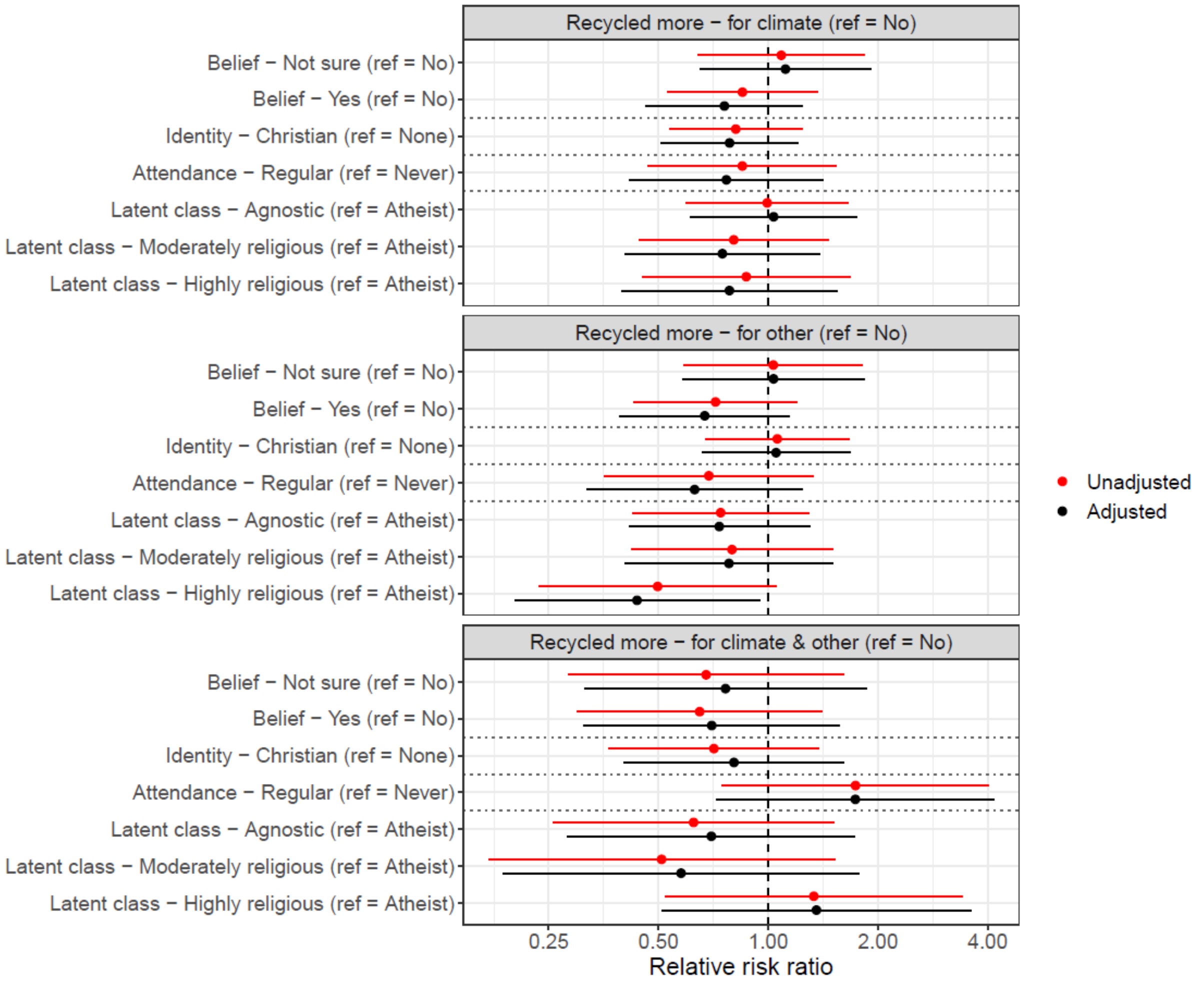

Figure S131: Predicted probabilities of the partners multinomial regression models with 'recycled or upcycled more' as the outcome for four religious exposures (belief, identity, attendance and latent classes).

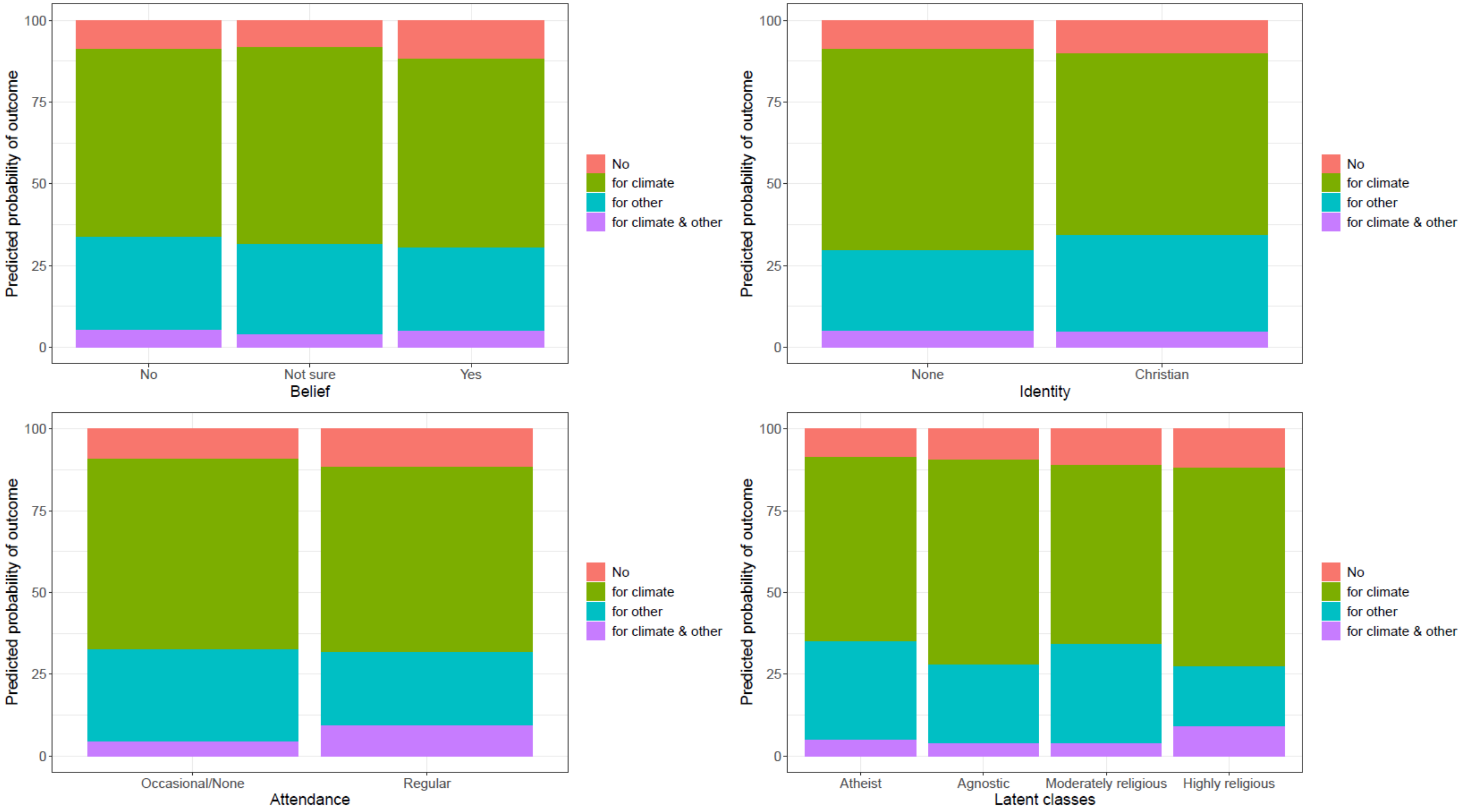

Figure S132: Predicted probabilities of the partners multinomial regression models with 'recycled or upcycled more' as the outcome and the religious identity (with the Christian denominations separated) as the exposure.

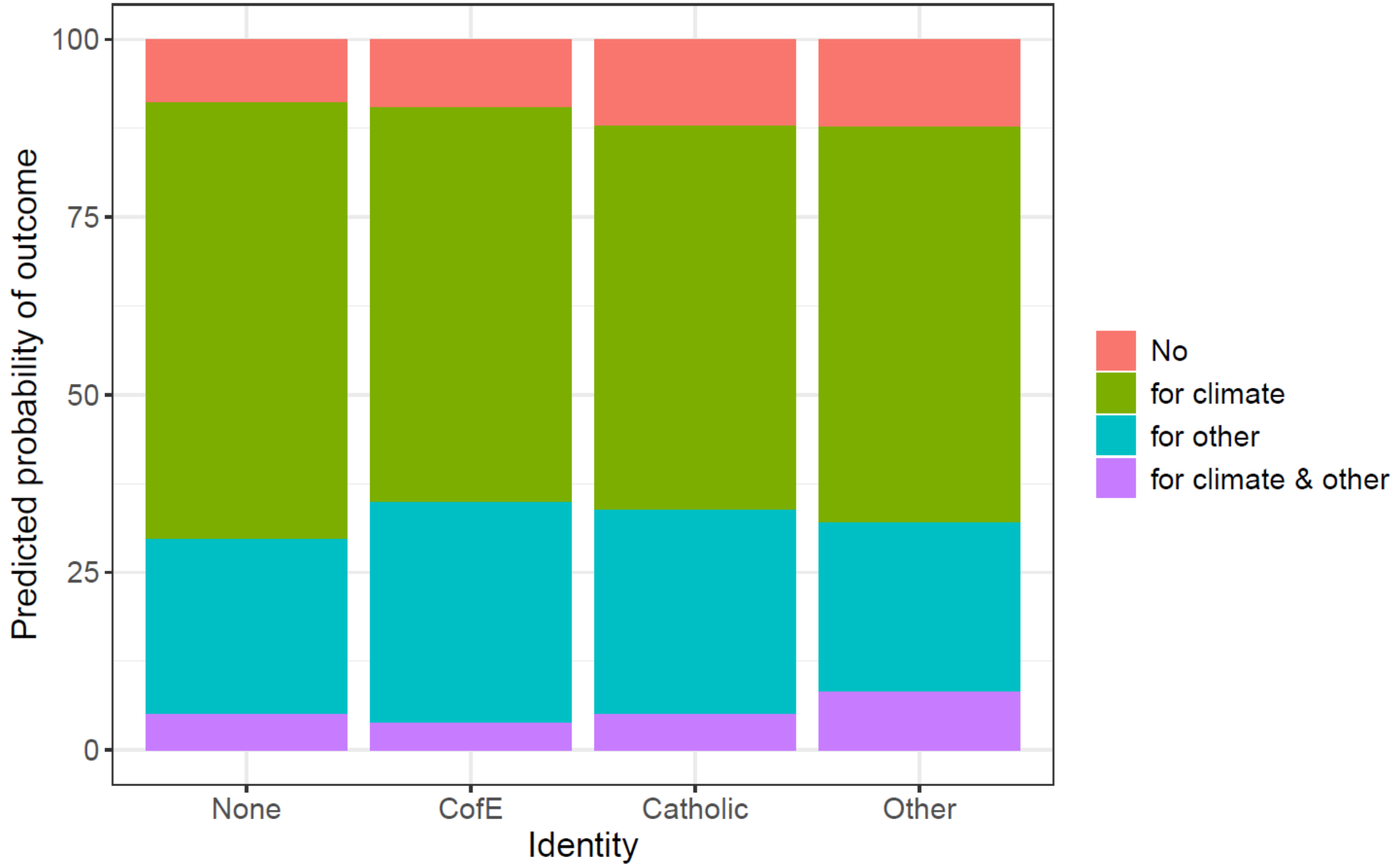

Figure S133: Results of the partners multinomial regression models with 'reduced the amount of plastic used' as the outcome for four religious exposures (belief [*n* = 1,111], identity [*n* = 1,100], attendance [*n* = 1,113], and latent classes [*n* = 1,121]; models are separated by dashed horizontal lines). See table S31 for full results.

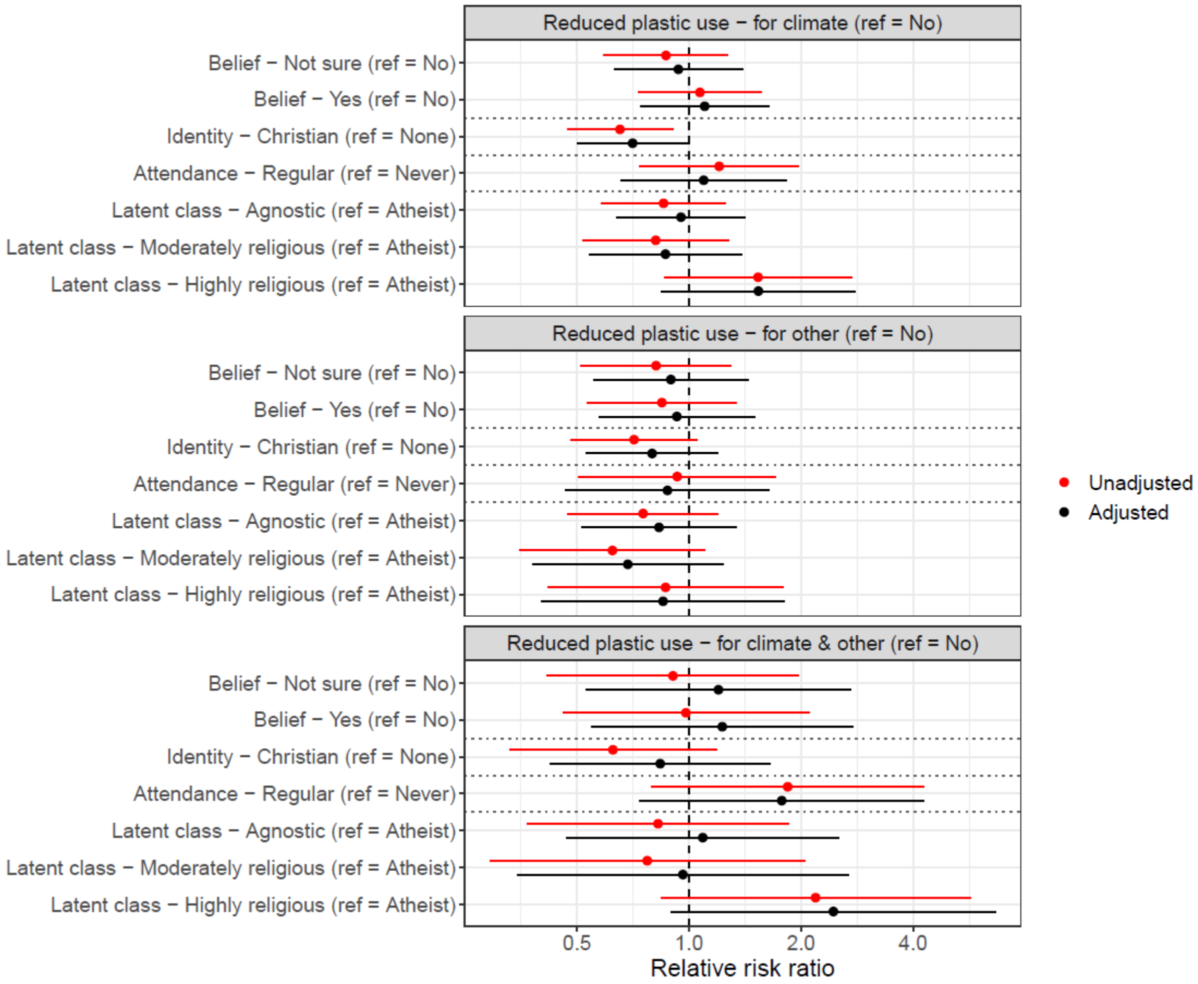

Figure S134: Predicted probabilities of the partners multinomial regression models with 'reduced the amount of plastic used' as the outcome for four religious exposures (belief, identity, attendance and latent classes).

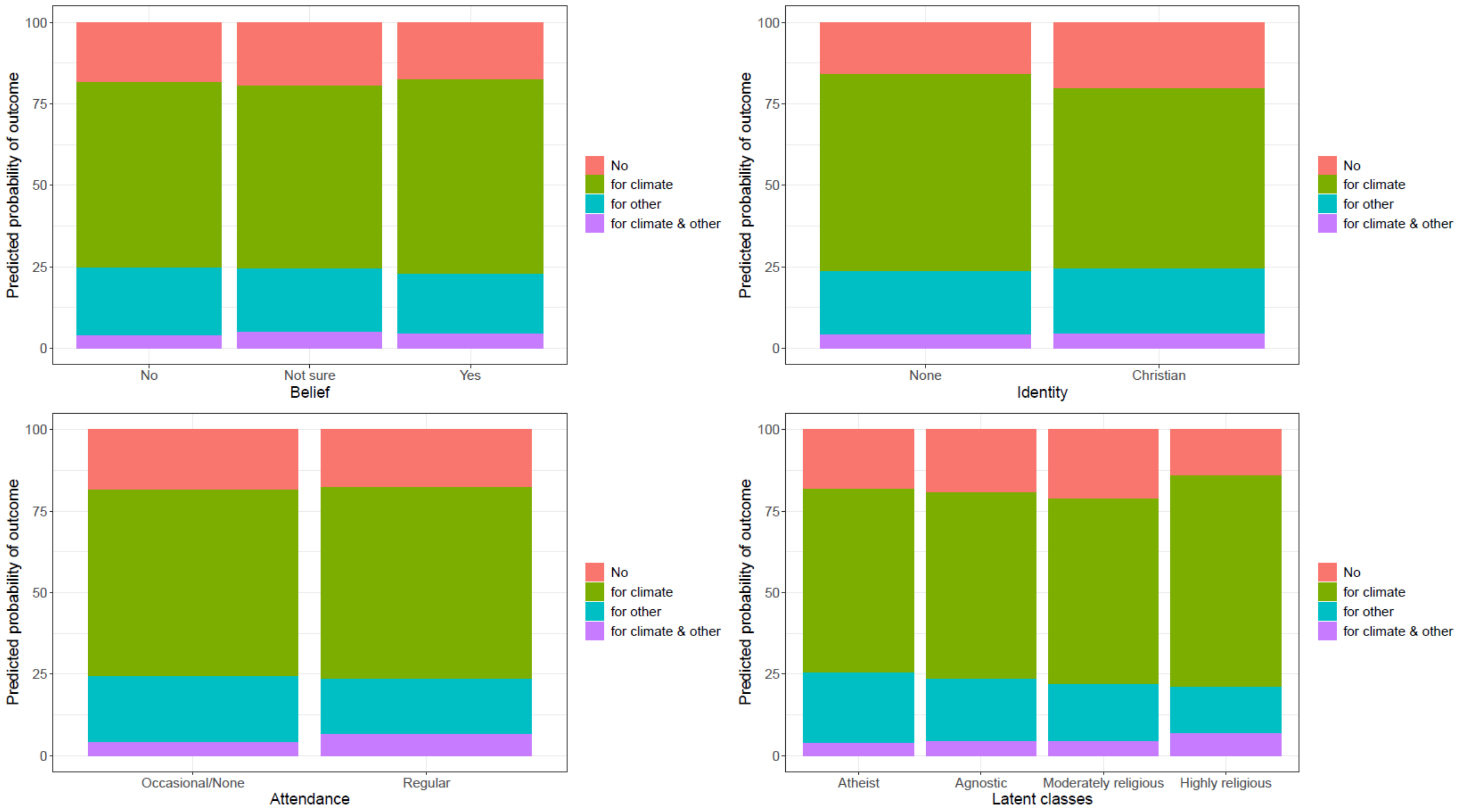

Figure S135: Predicted probabilities of the partners multinomial regression models with ‘reduced the amount of plastic used’ as the outcome and the religious identity (with the Christian denominations separated) as the exposure.

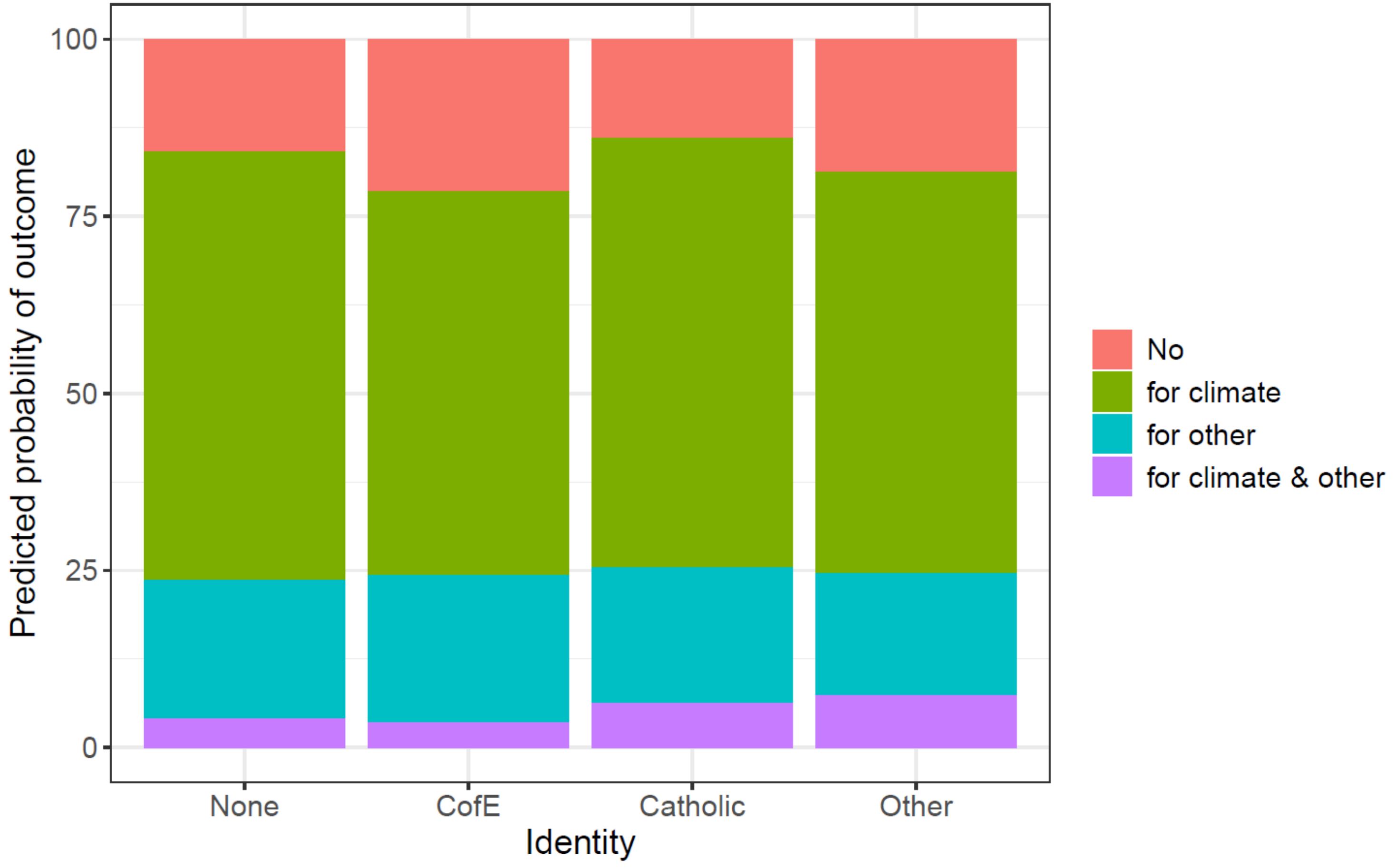

Figure S136: Results of the partners multinomial regression models with 'chosen sustainably sourced items' as the outcome for four religious exposures (belief [*n* = 1,103], identity [*n* = 1,093], attendance [*n* = 1,106], and latent classes [*n* = 1,113]; models are separated by dashed horizontal lines). See table S31 for full results.

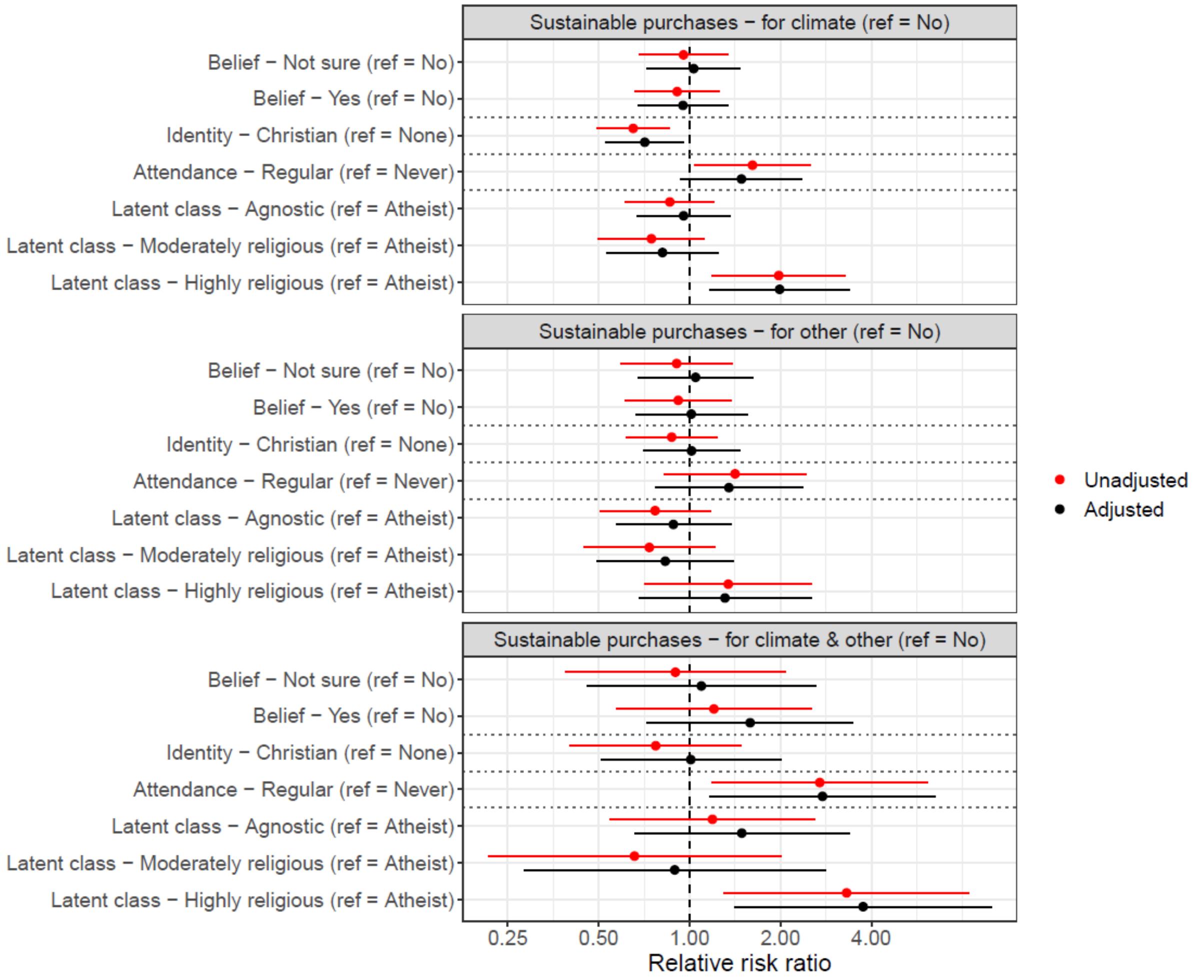

Figure S137: Predicted probabilities of the partners multinomial regression models with 'chosen sustainably sourced items' as the outcome for four religious exposures (belief, identity, attendance and latent classes).

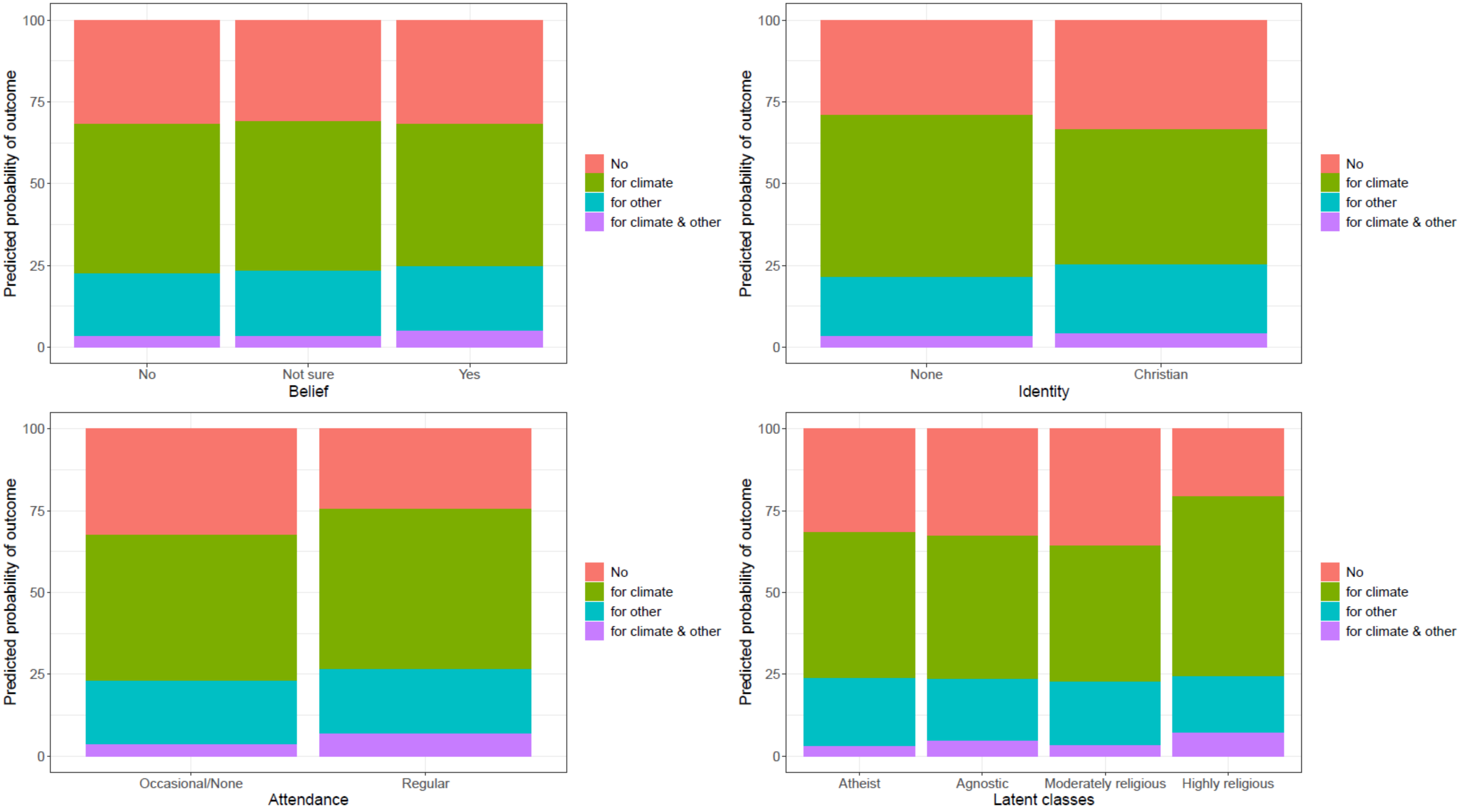

Figure S138: Predicted probabilities of the partners multinomial regression models with 'chosen sustainably sourced items' as the outcome and the religious identity (with the Christian denominations separated) as the exposure.

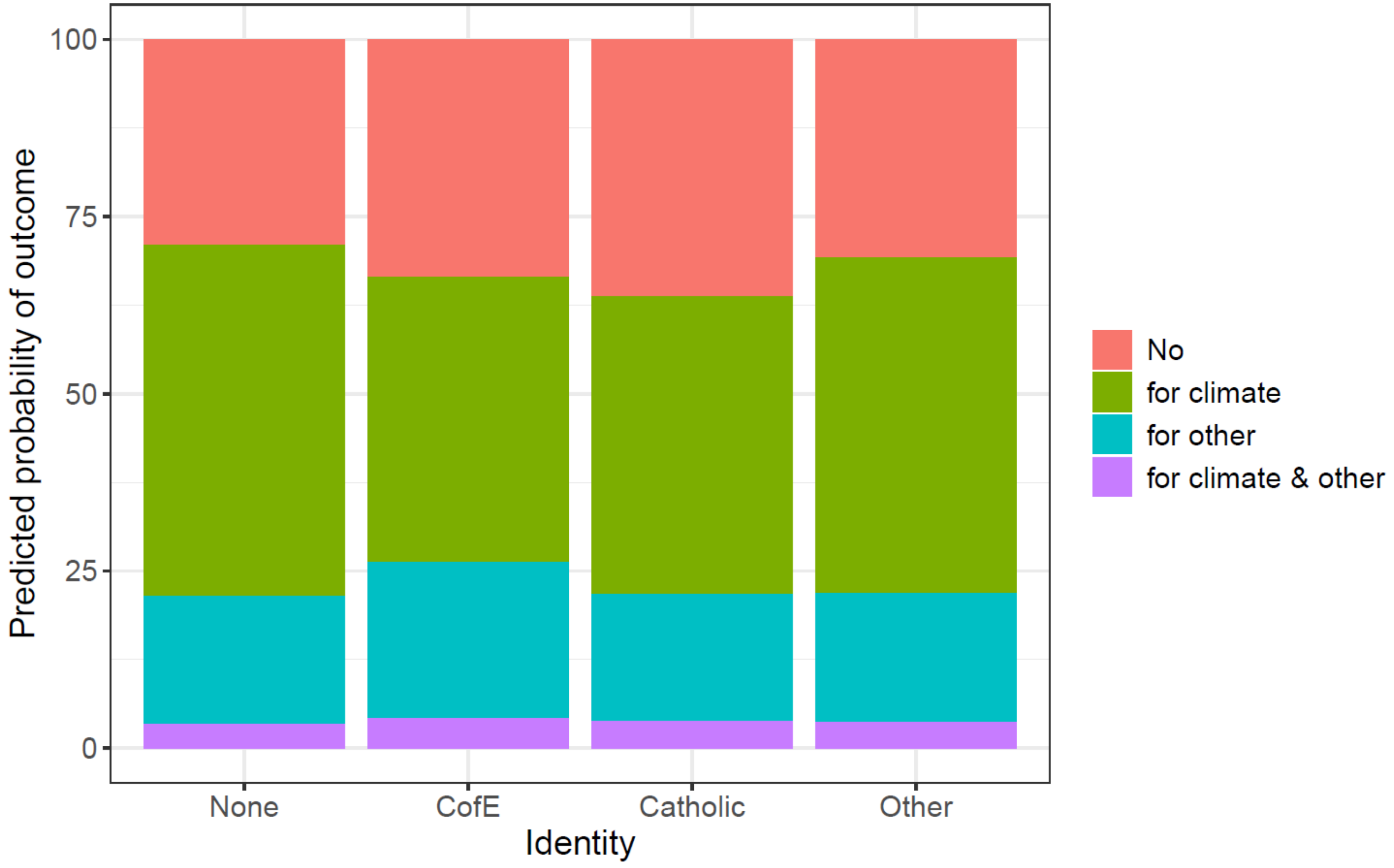

Figure S139: Results of the partners multinomial regression models with 'improved home insulation' as the outcome for four religious exposures (belief [*n* = 1,111], identity [*n* = 1,100], attendance [*n* = 1,113], and latent classes [*n* = 1,121]; models are separated by dashed horizontal lines). See table S31 for full results.

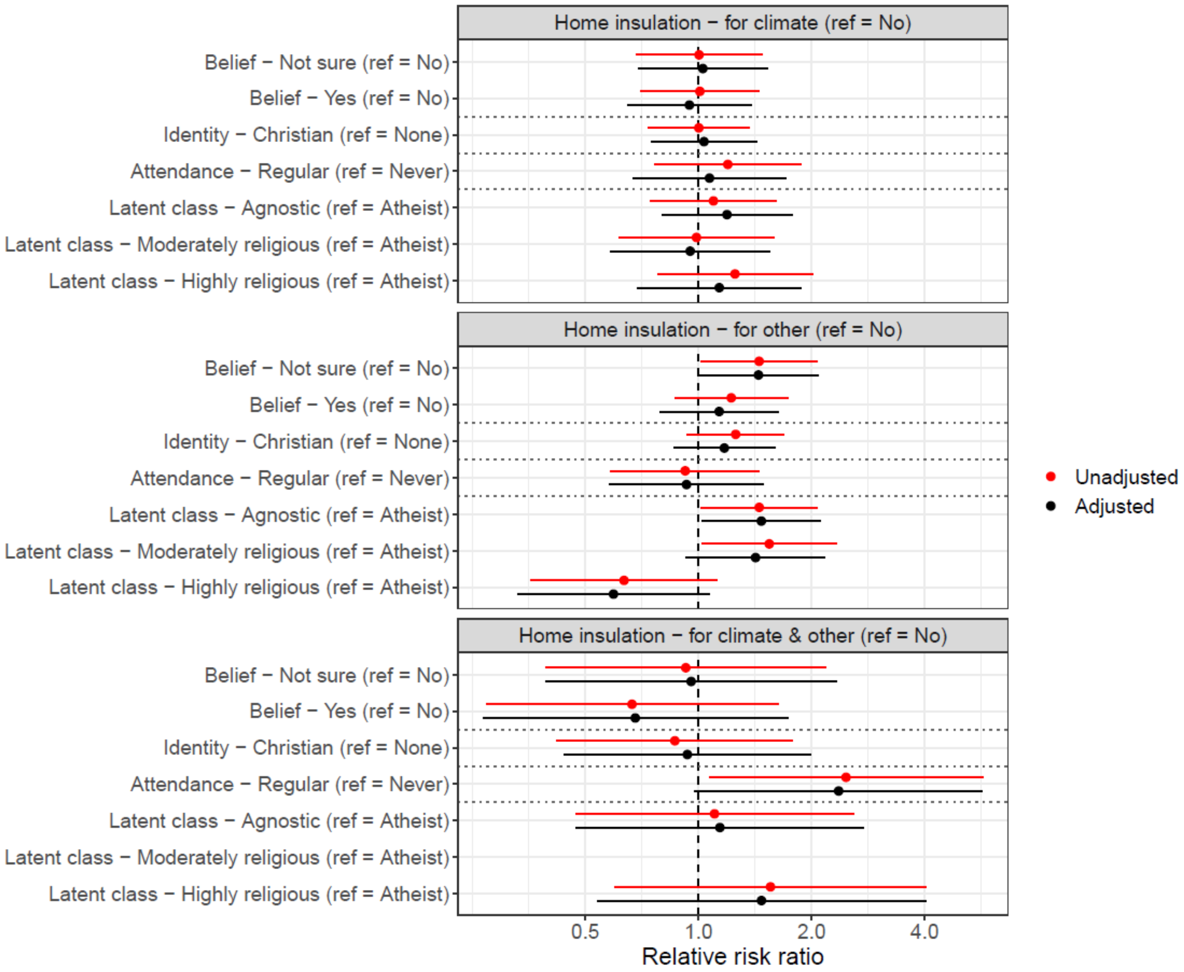

Figure S140: Predicted probabilities of the partners multinomial regression models with 'improved home insulation' as the outcome for four religious exposures (belief, identity, attendance and latent classes).

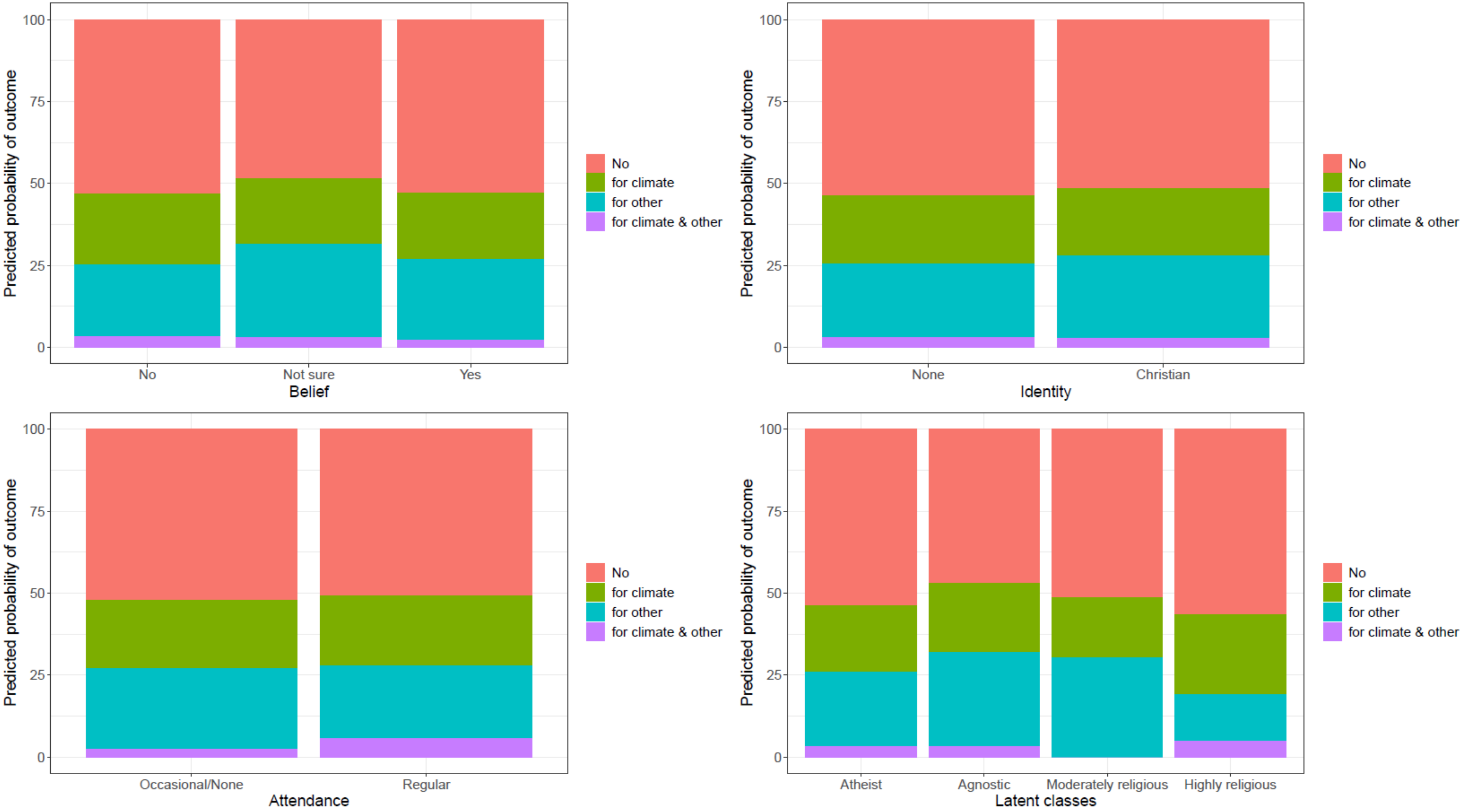

Figure S141: Predicted probabilities of the partners multinomial regression models with 'improved home insulation' as the outcome and the religious identity (with the Christian denominations separated) as the exposure.

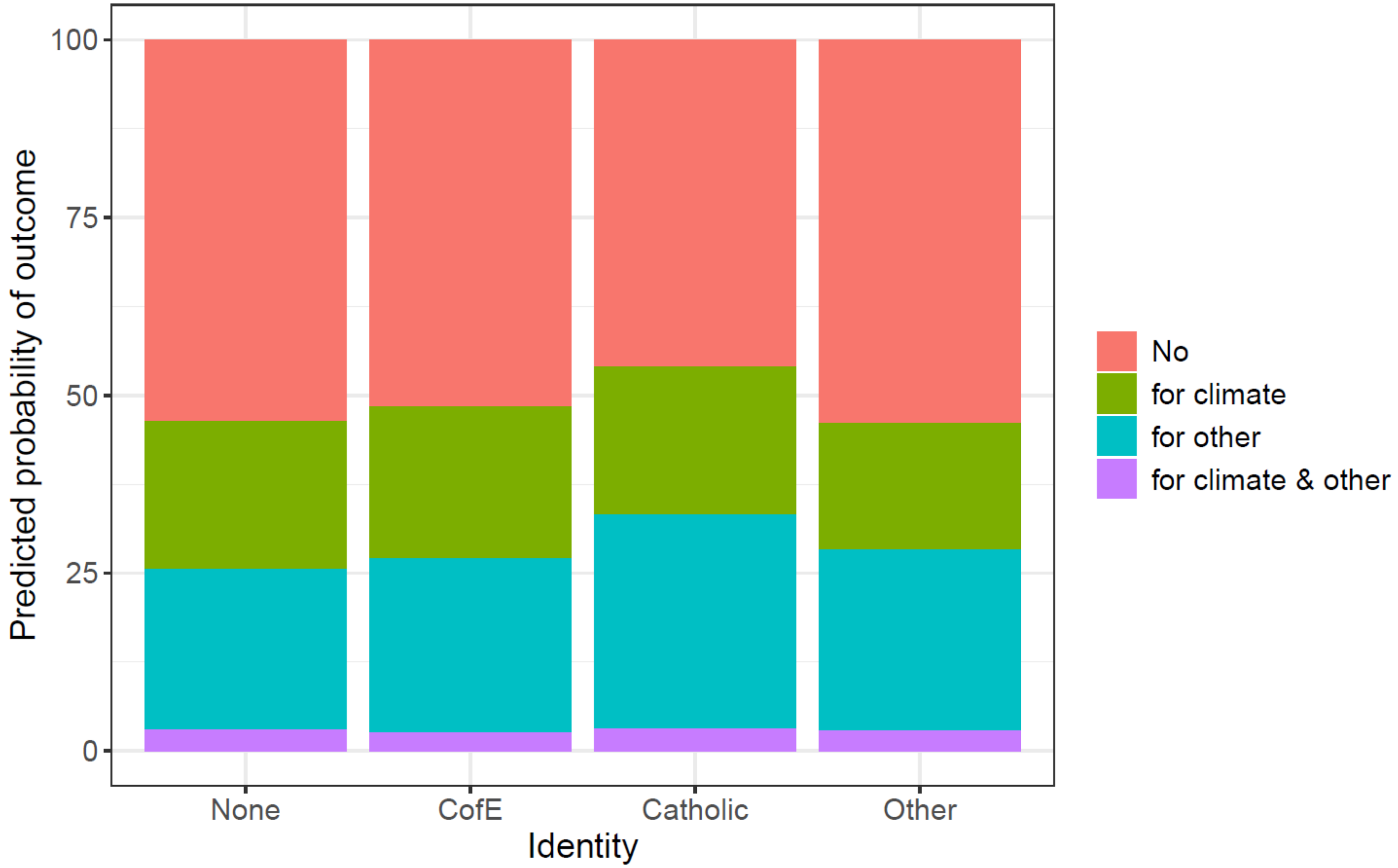

Figure S142: Results of the partners multinomial regression models with 'installed solar panels' as the outcome for four religious exposures (belief [*n* = 1,103], identity [*n* = 1,092], attendance [*n* = 1,105], and latent classes [*n* = 1,113]; models are separated by dashed horizontal lines). See table S31 for full results.

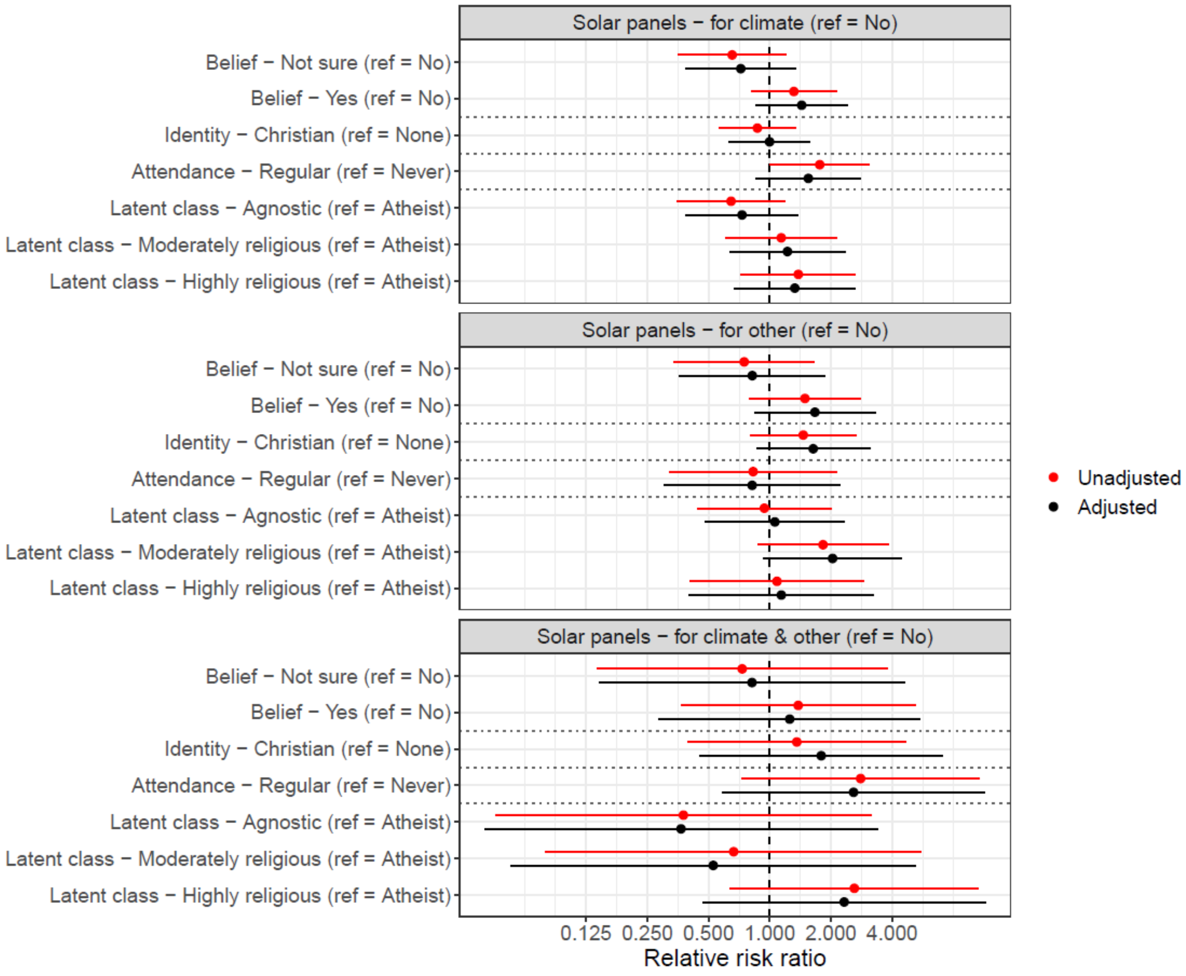

Figure S143: Predicted probabilities of the partners multinomial regression models with ‘installed solar panels’ as the outcome for four religious exposures (belief, identity, attendance and latent classes).

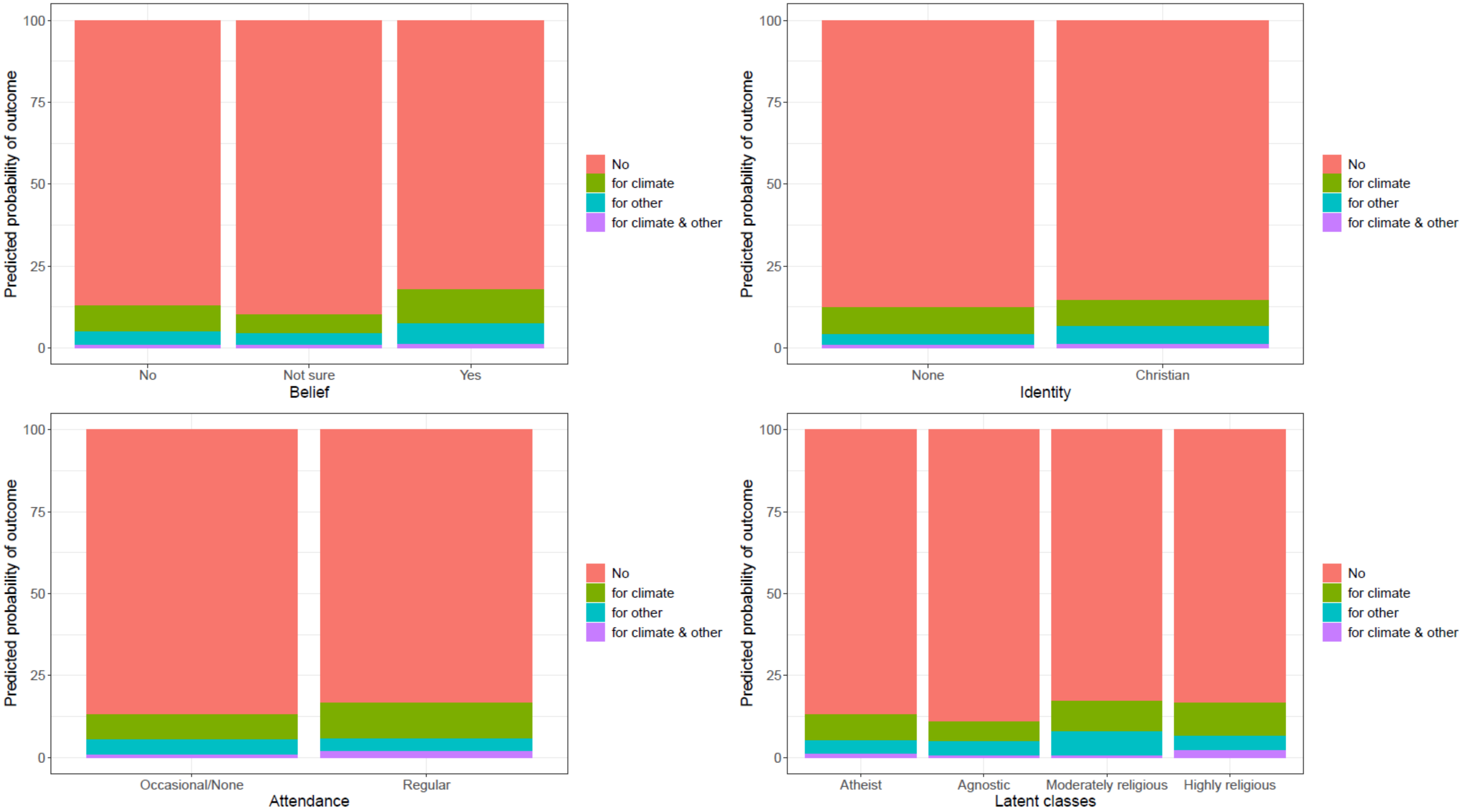

Figure S144: Predicted probabilities of the partners multinomial regression models with 'installed solar panels' as the outcome and the religious identity (with the Christian denominations separated) as the exposure.

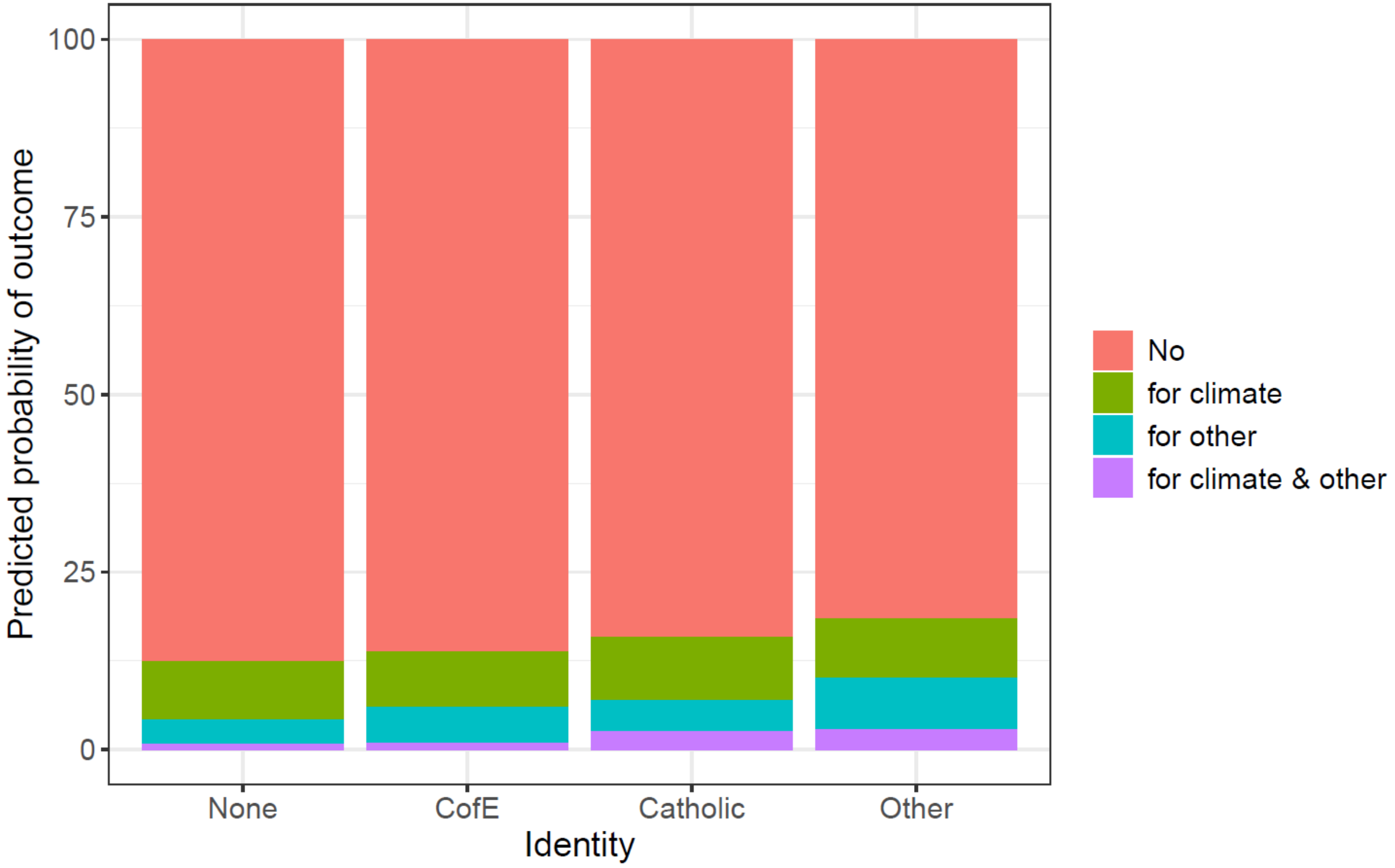

Figure S145: Results of the partners multinomial regression models with 'started growing vegetables' as the outcome for four religious exposures (belief [*n* = 1,110], identity [*n* = 1,099], attendance [*n* = 1,112], and latent classes [*n* = 1,120]; models are separated by dashed horizontal lines). See table S31 for full results.

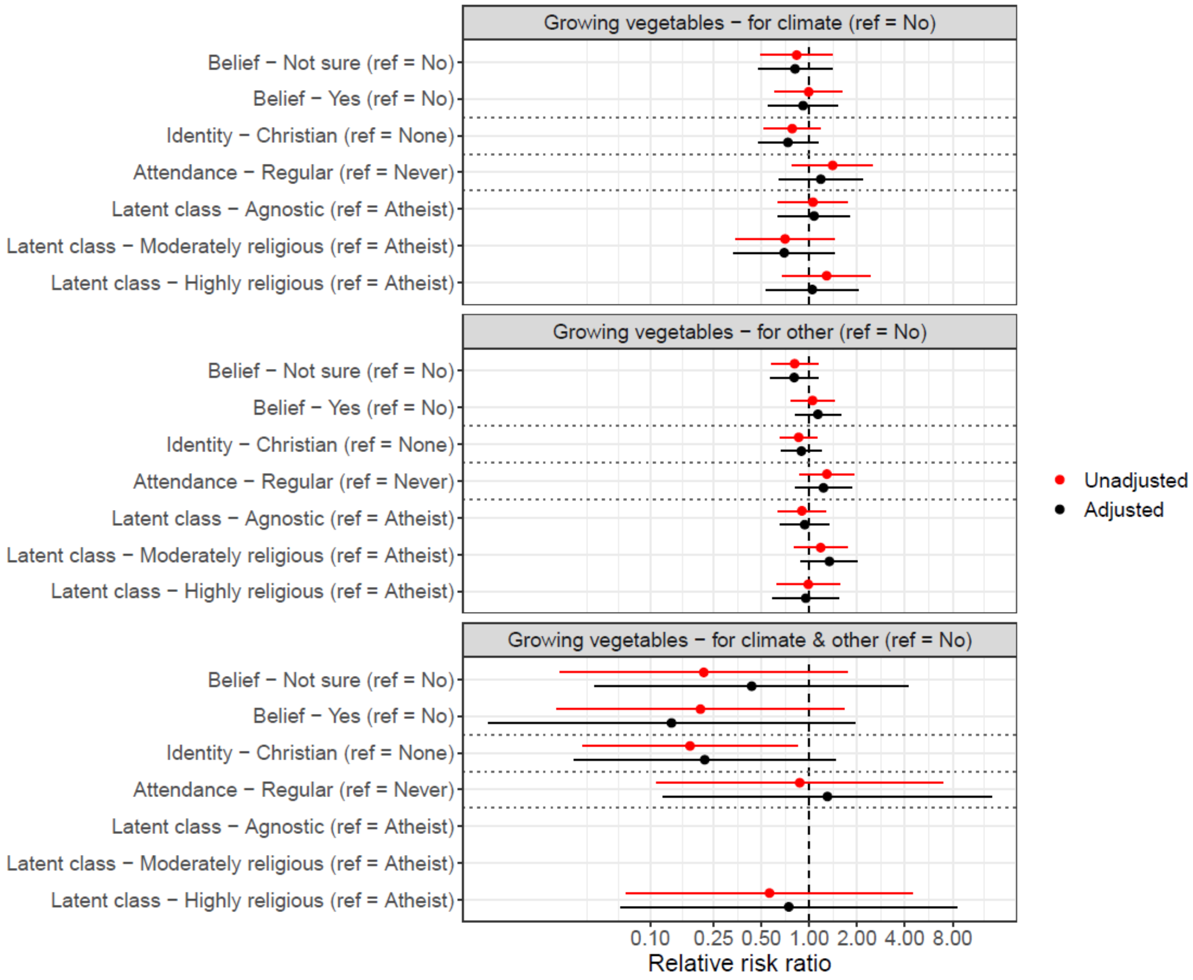

Figure S146: Predicted probabilities of the partners multinomial regression models with ‘started growing vegetables’ as the outcome for four religious exposures (belief, identity, attendance and latent classes).

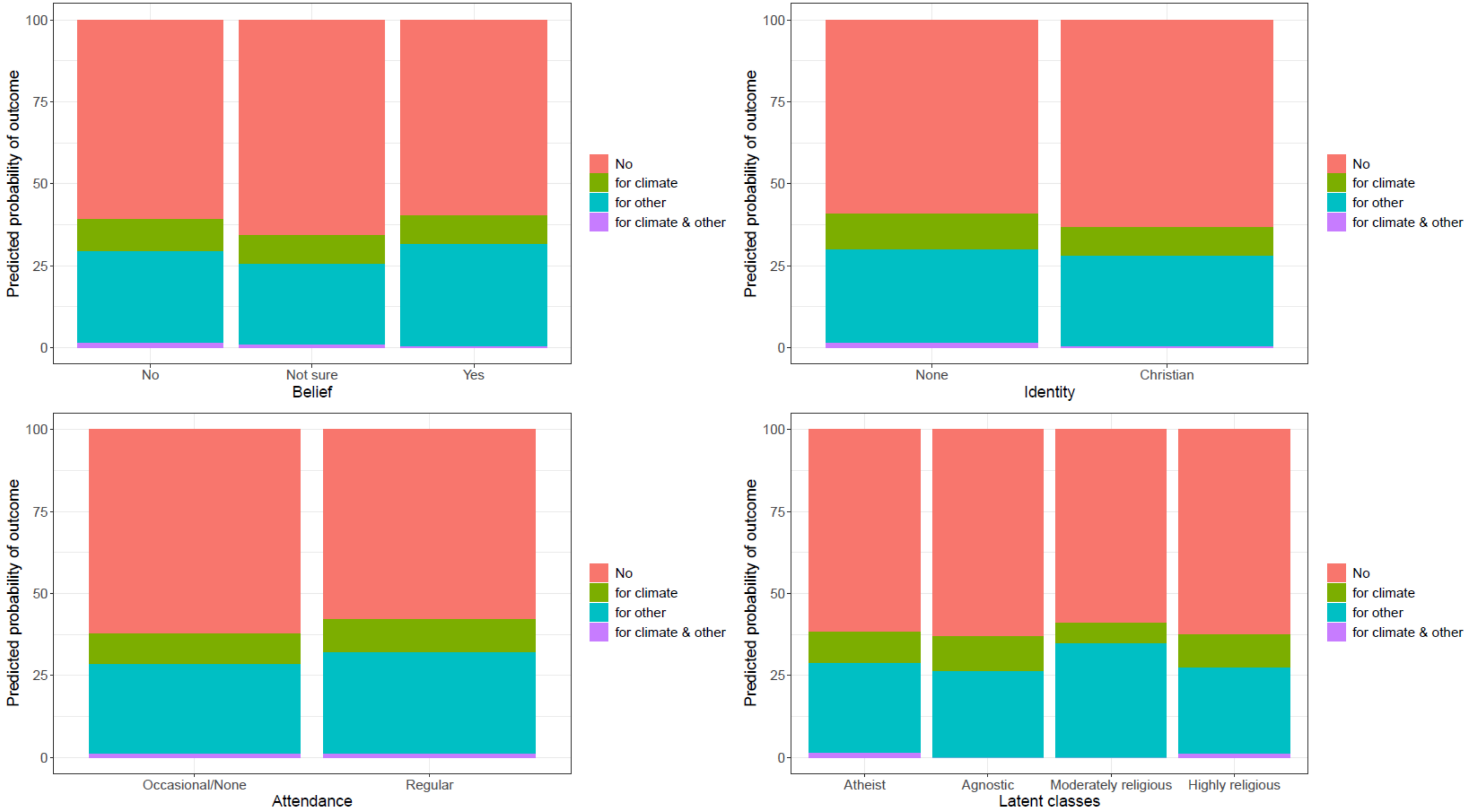

Figure S147: Predicted probabilities of the partners multinomial regression models with 'started growing vegetables' as the outcome and the religious identity (with the Christian denominations separated) as the exposure.

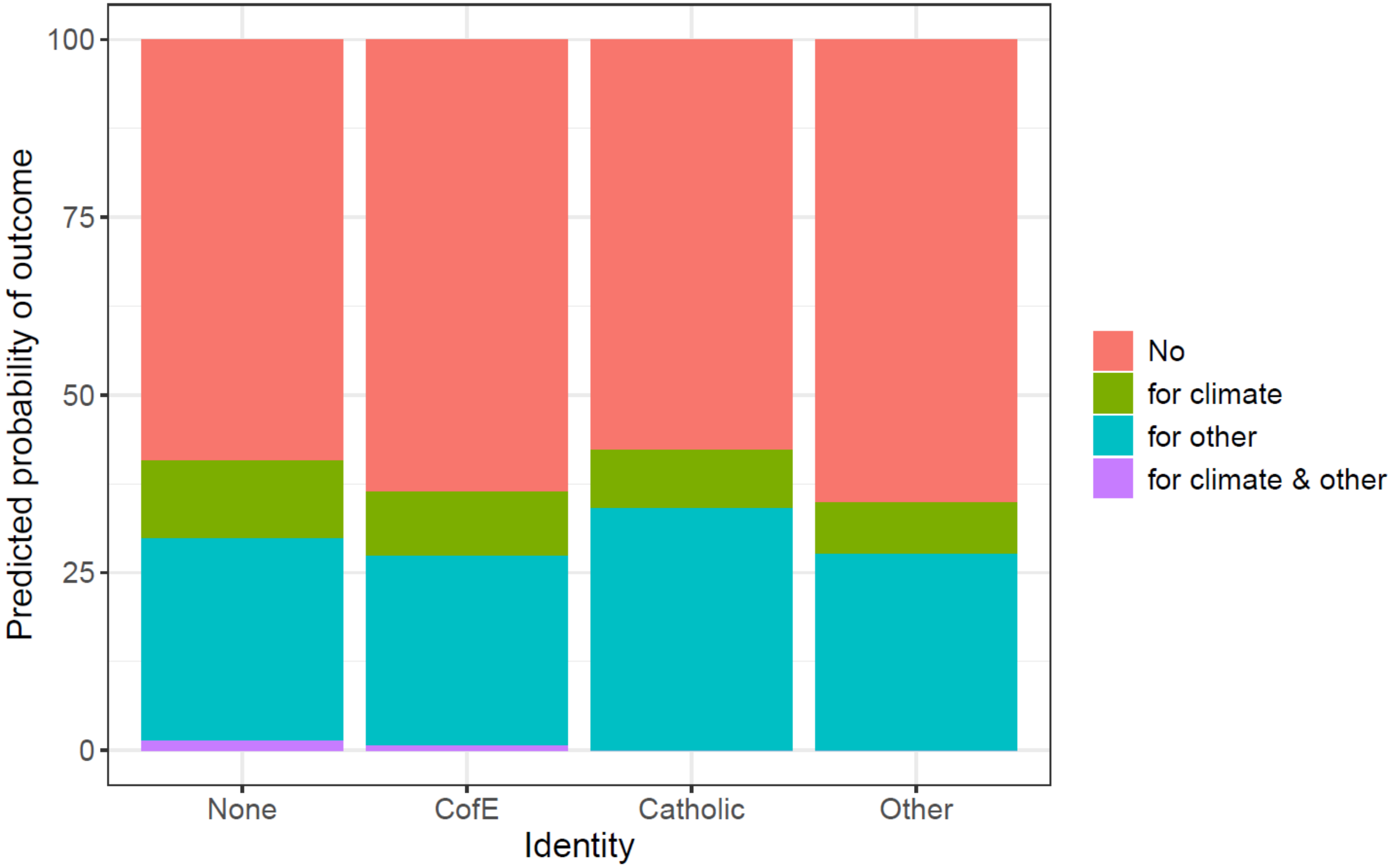

Figure S148: Results of the partners multinomial regression models with 'planted trees' as the outcome for four religious exposures (belief [*n* = 1,110], identity [*n* = 1,089], attendance [*n* = 1,102], and latent classes [*n* = 1,110]; models are separated by dashed horizontal lines). See table S31 for full results.

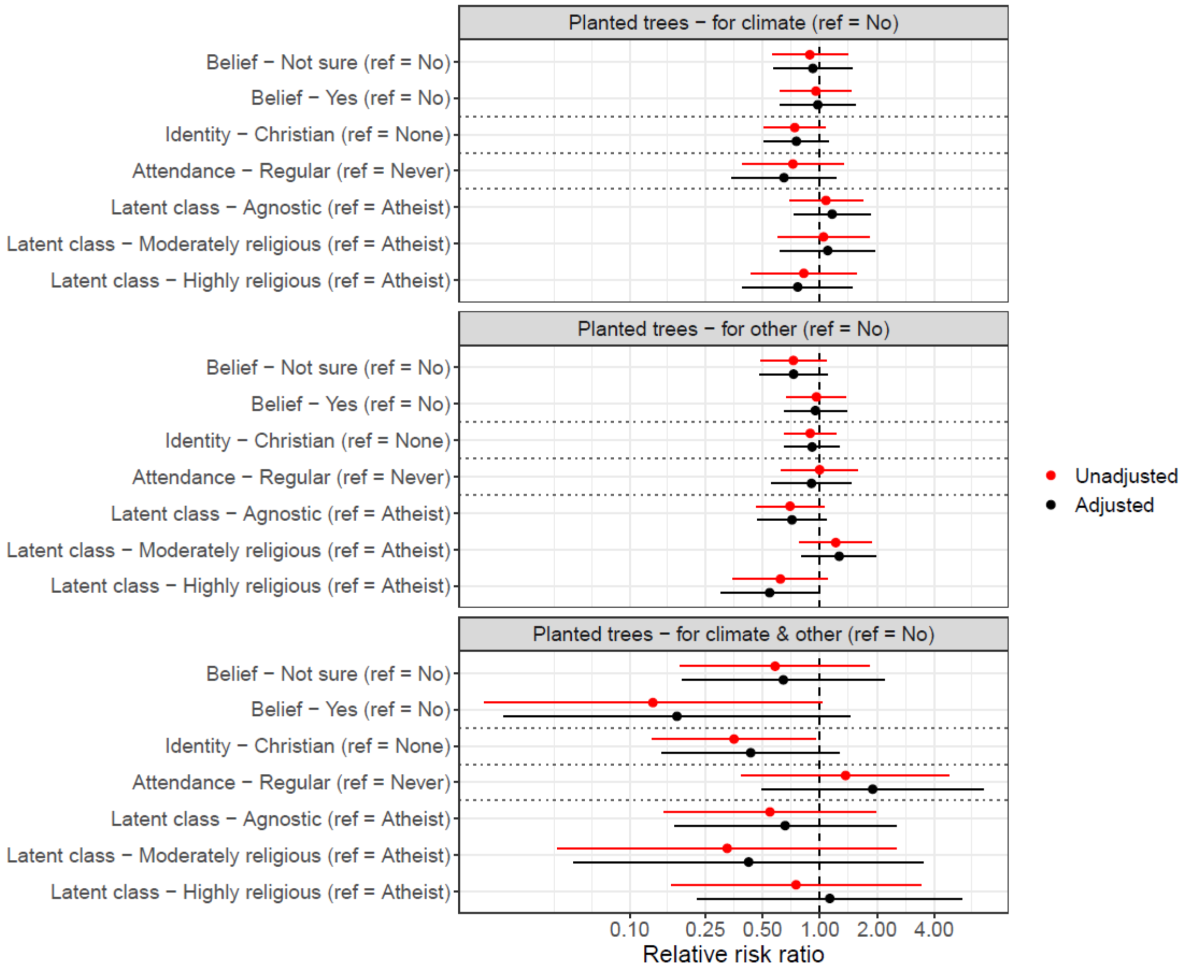

Figure S149: Predicted probabilities of the partners multinomial regression models with 'planted trees' as the outcome for four religious exposures (belief, identity, attendance and latent classes).

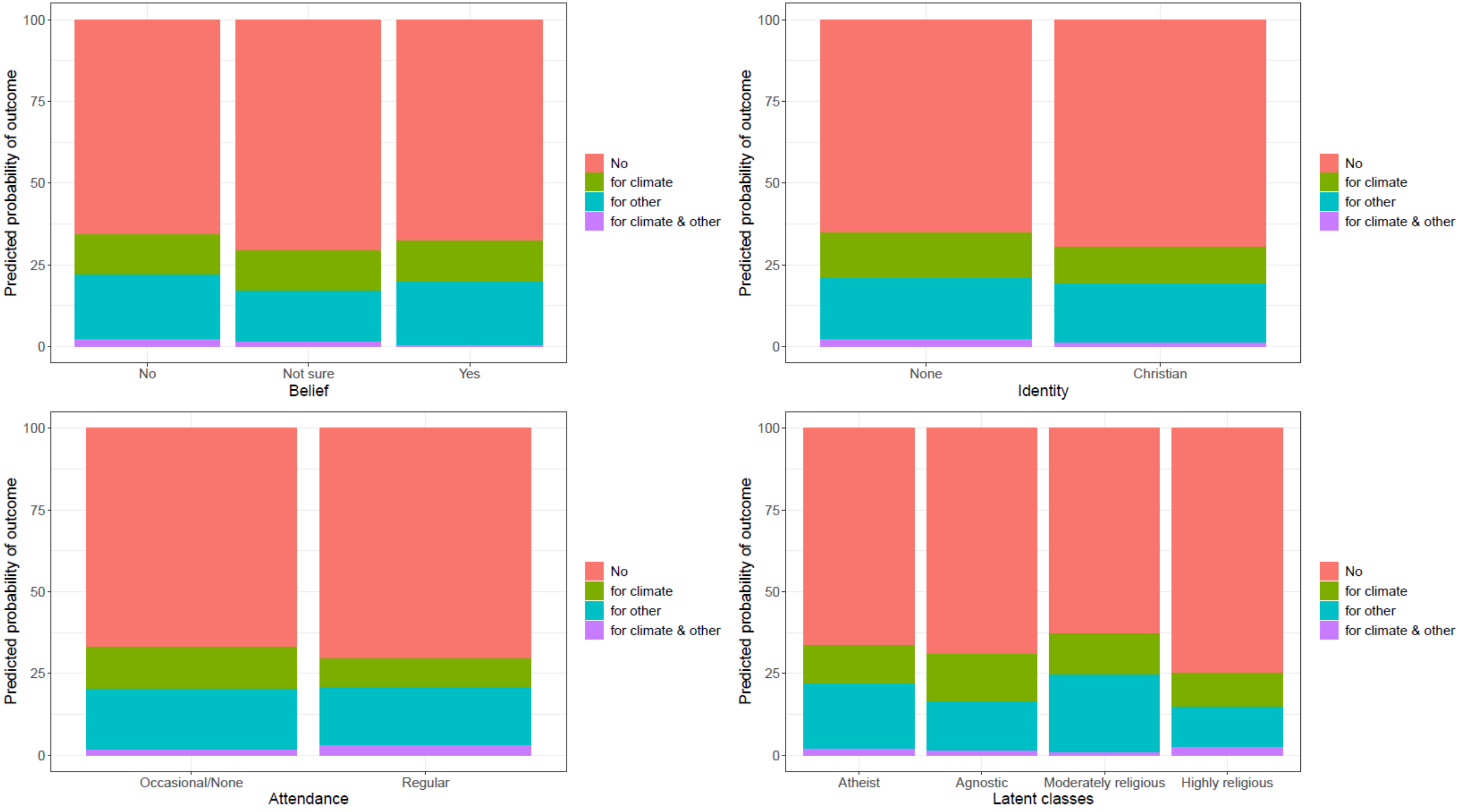

Figure S150: Predicted probabilities of the partners multinomial regression models with 'planted trees' as the outcome and the religious identity (with the Christian denominations separated) as the exposure.

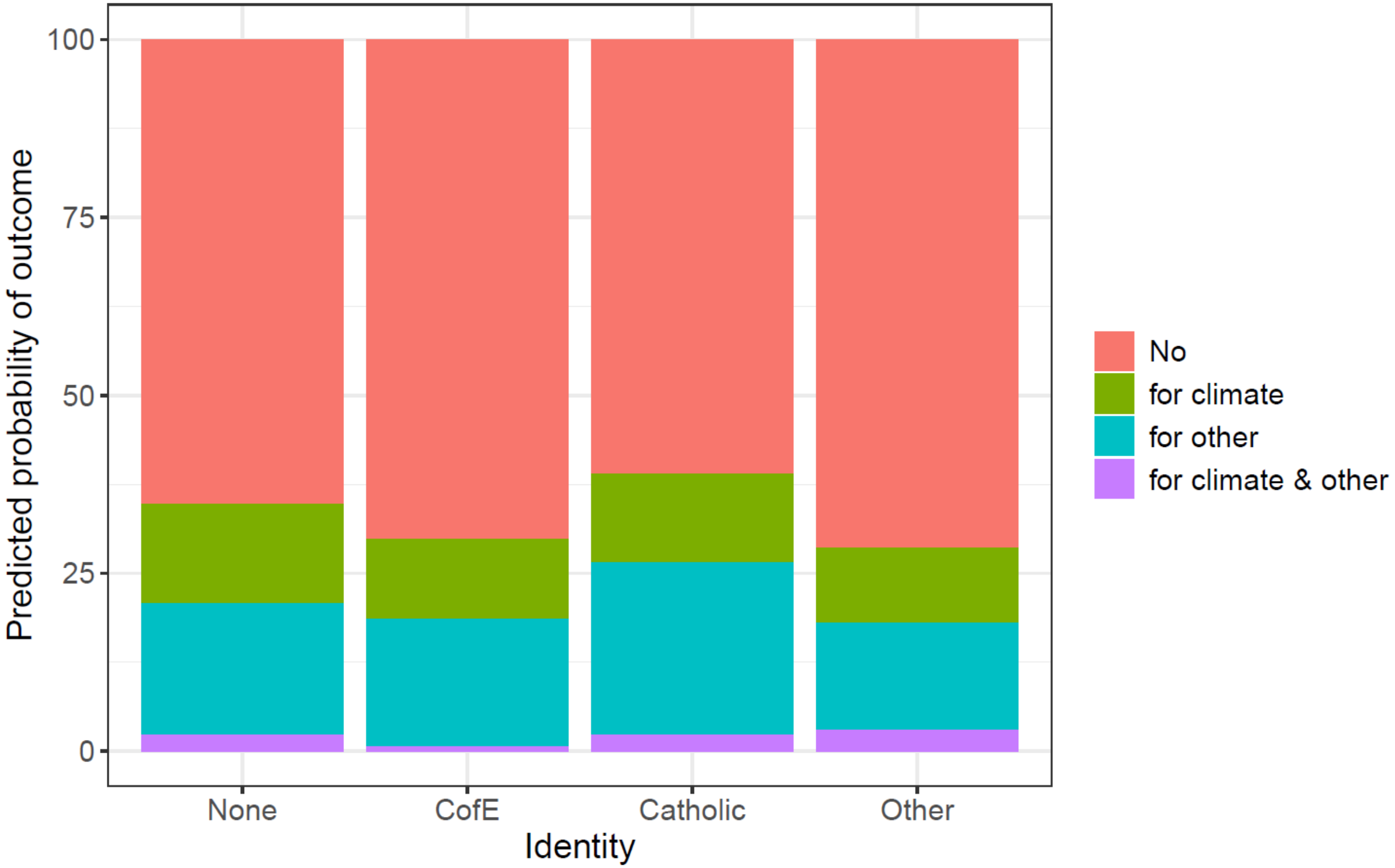

Figure S151: Results of the partners multinomial regression models with 'avoided organisations that support fossil fuels' as the outcome for four religious exposures (belief [*n* = 1,105], identity [*n* = 1,094], attendance [*n* = 1,107], and latent classes [*n* = 1,115]; models are separated by dashed horizontal lines). See table S31 for full results.

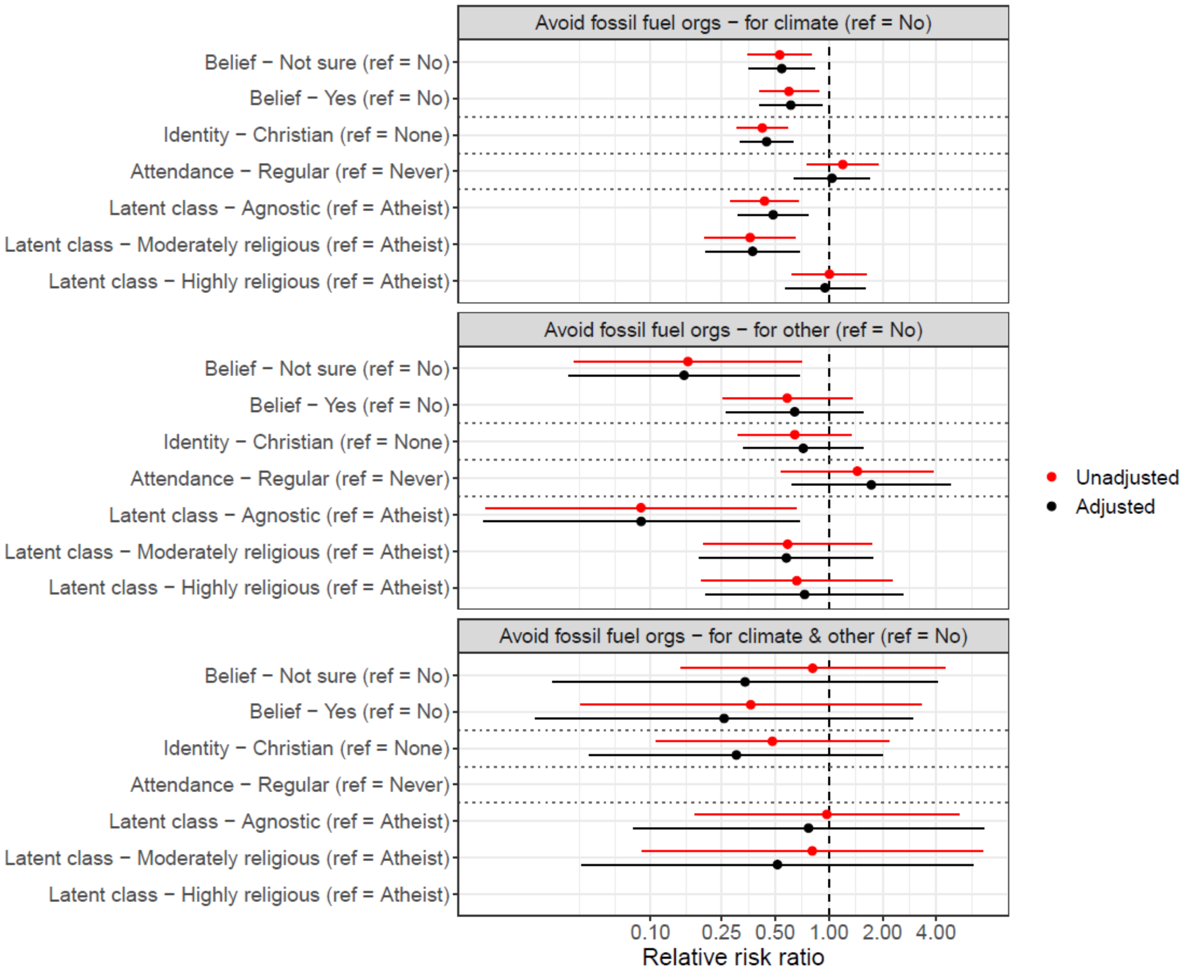

Figure S152: Predicted probabilities of the partners multinomial regression models with ‘avoided organisations that support fossil fuels’ as the outcome for four religious exposures (belief, identity, attendance and latent classes).

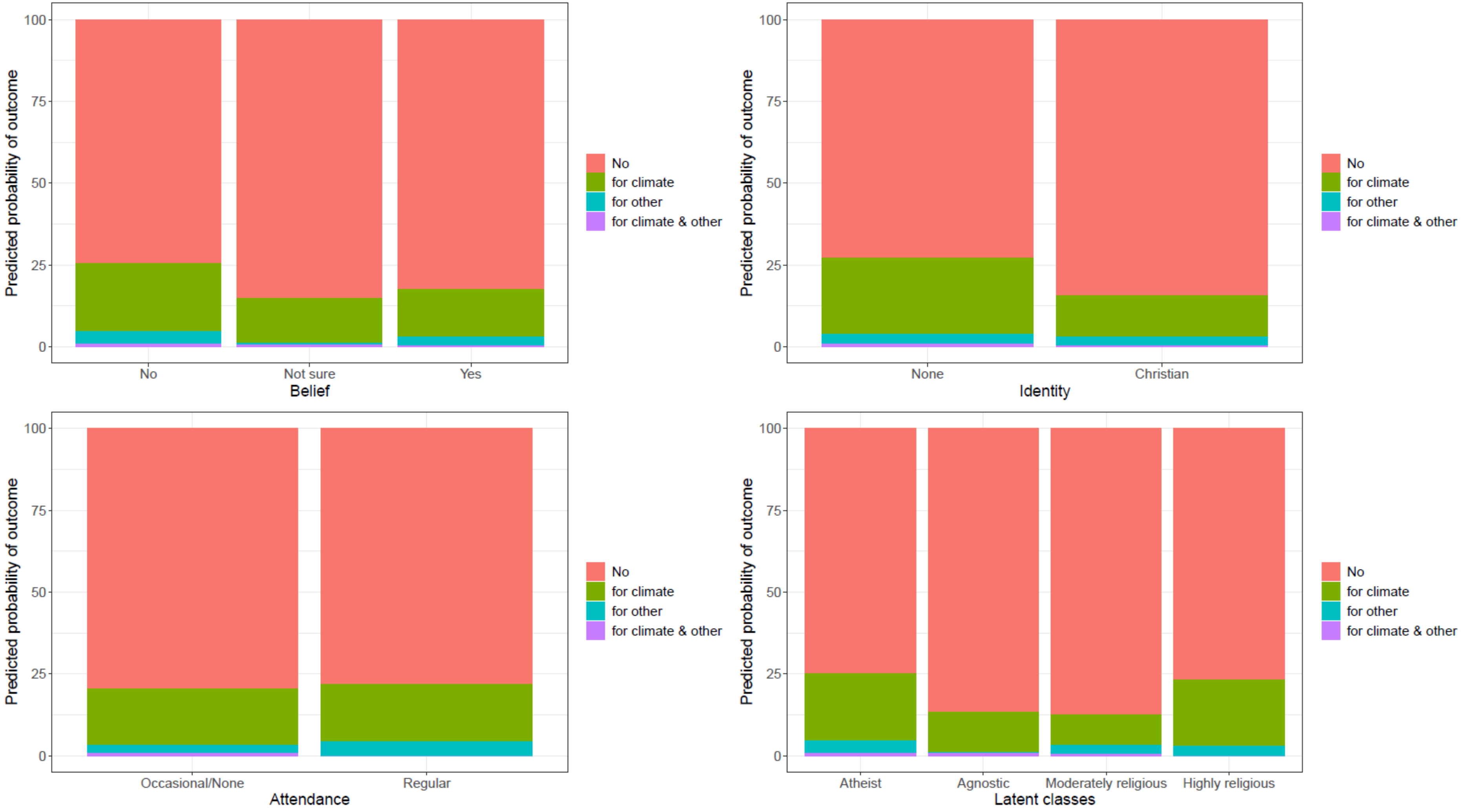

Figure S153: Predicted probabilities of the partners multinomial regression models with 'avoided organisations that support fossil fuels' as the outcome and the religious identity (with the Christian denominations separated) as the exposure.

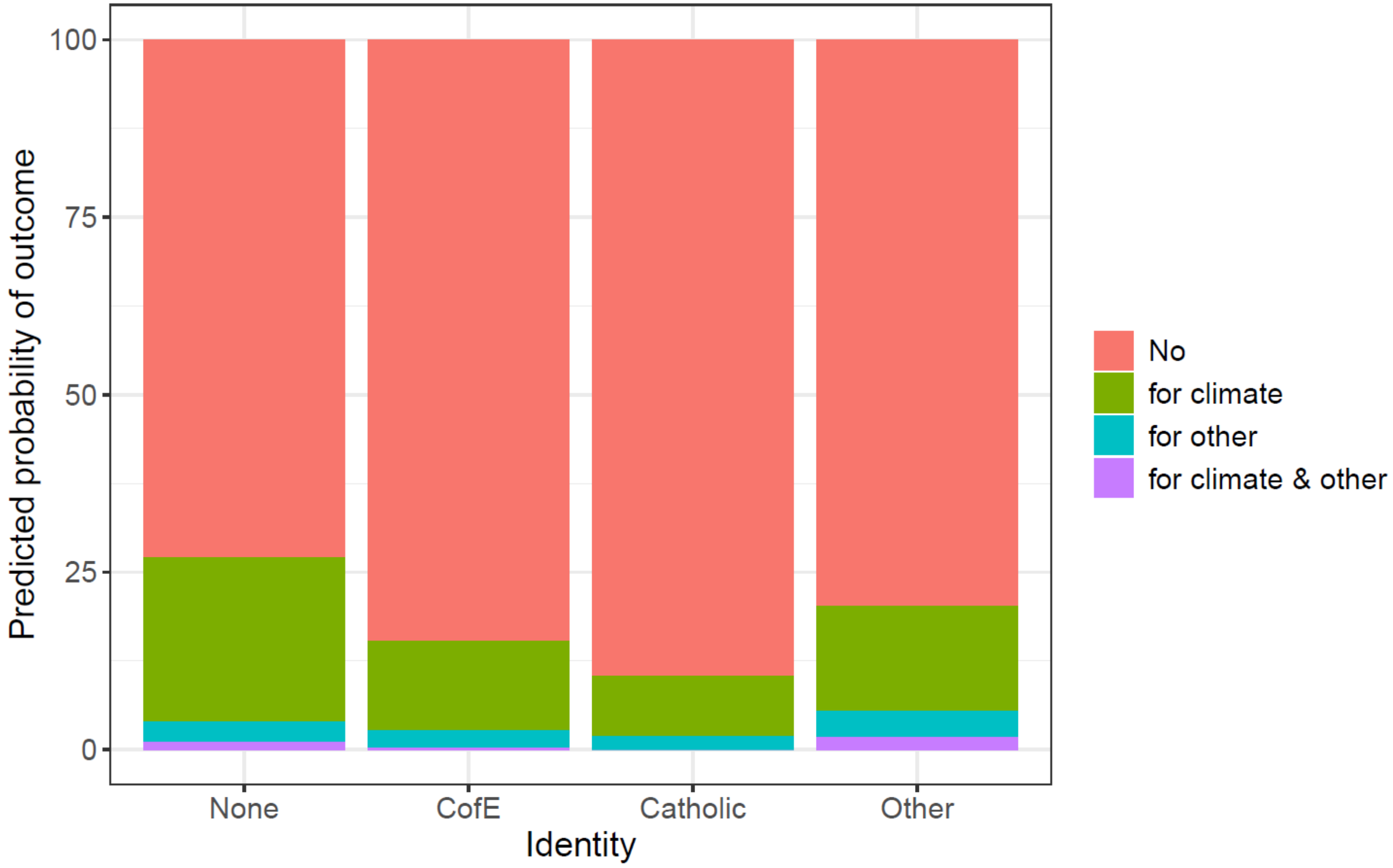

Figure S154: Results of the partners multinomial regression models with 'taken action to eat less or no meat and/or dairy' as the outcome for four religious exposures (belief [*n* = 1,070], identity [*n* = 1,061], attendance [*n* = 1,074], and latent classes [*n* = 1,080]; models are separated by dashed horizontal lines). See table S31 for full results.

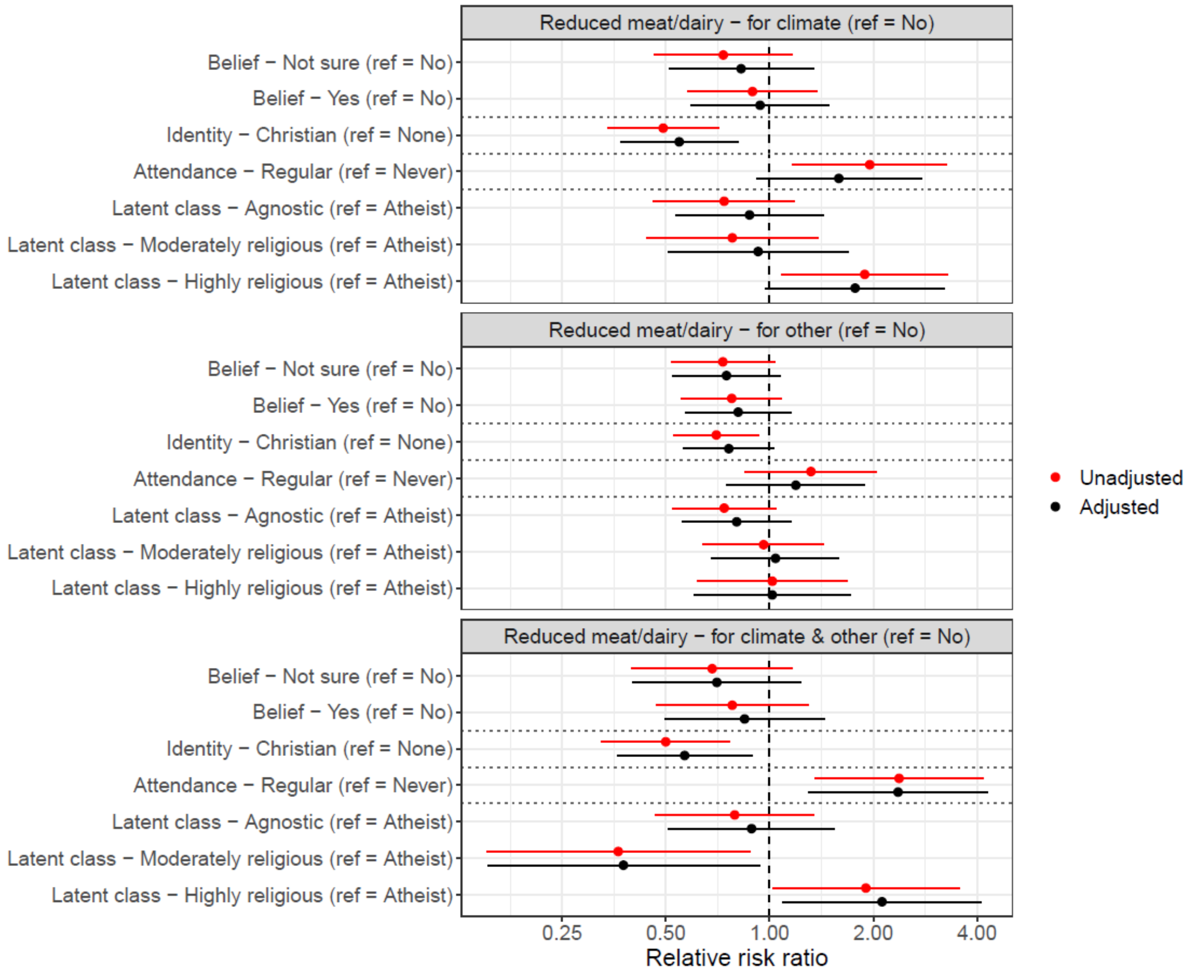

Figure S155: Predicted probabilities of the partners multinomial regression models with 'taken action to eat less or no meat and/or dairy' as the outcome for four religious exposures (belief, identity, attendance and latent classes).

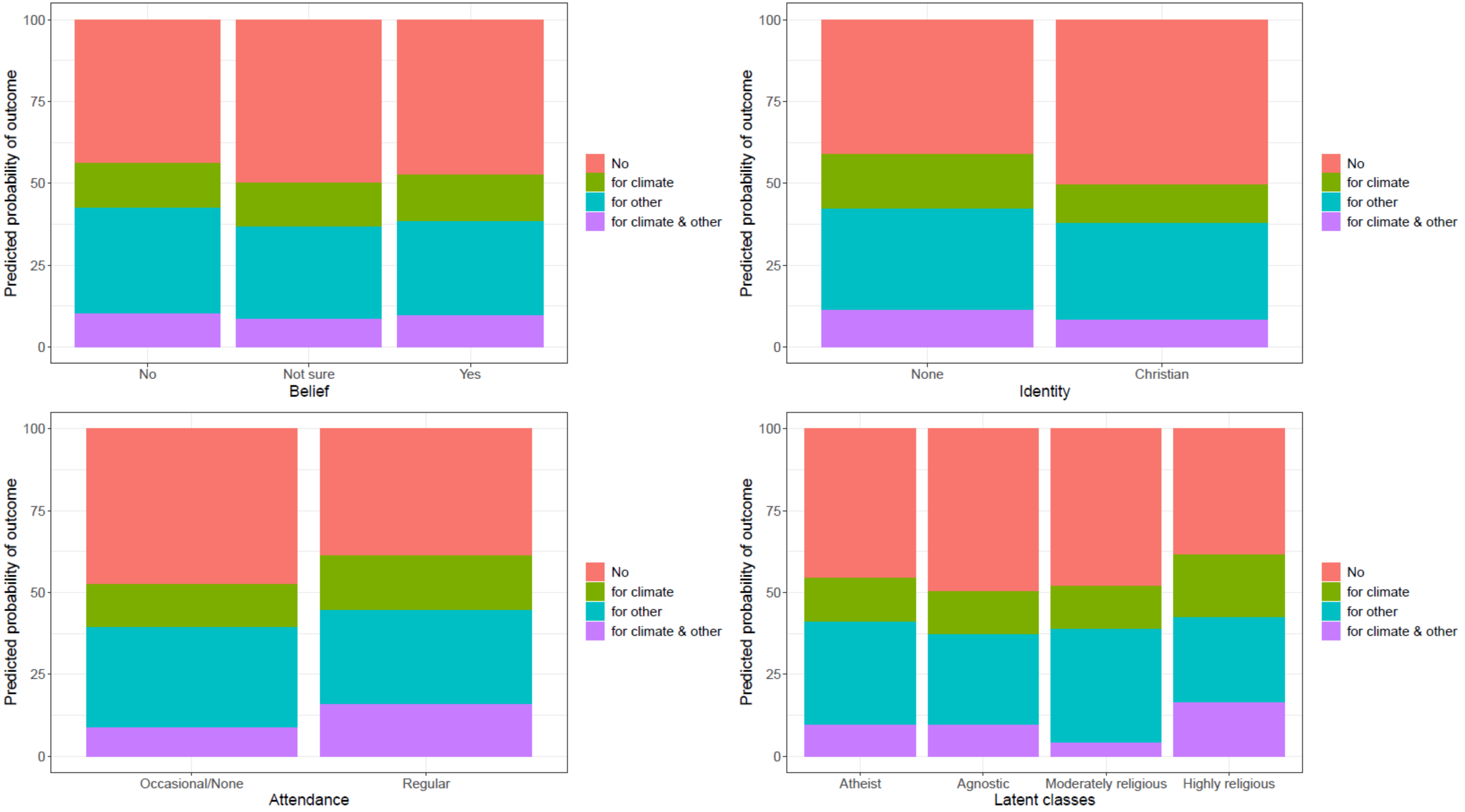

Figure S156: Predicted probabilities of the partners multinomial regression models with ‘taken action to eat less or no meat and/or dairy’ as the outcome and the religious identity (with the Christian denominations separated) as the exposure.

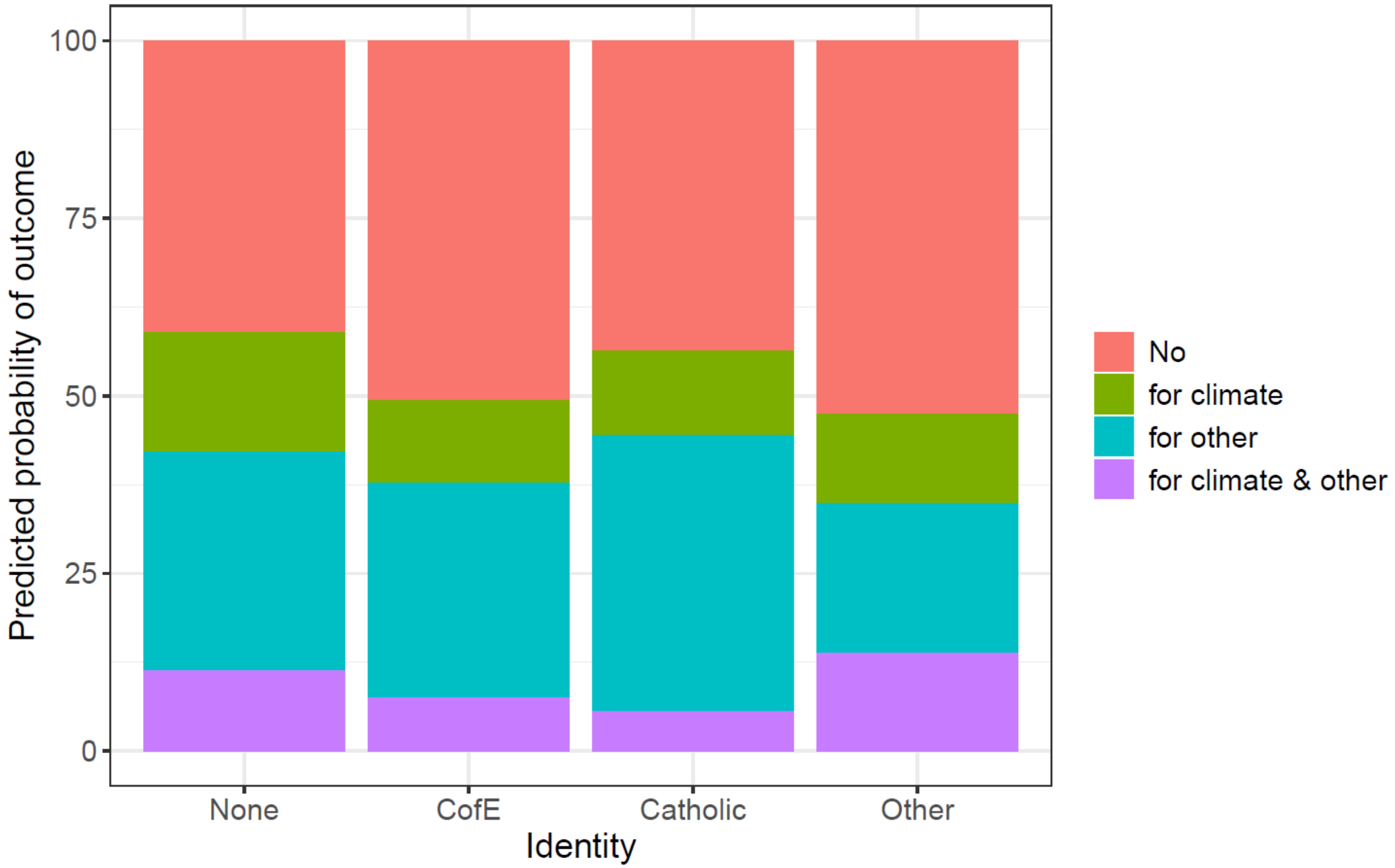

Figure S157: Histogram of total number of pro-environmental actions performed for climate change reasons in G1 offspring (max = 17).

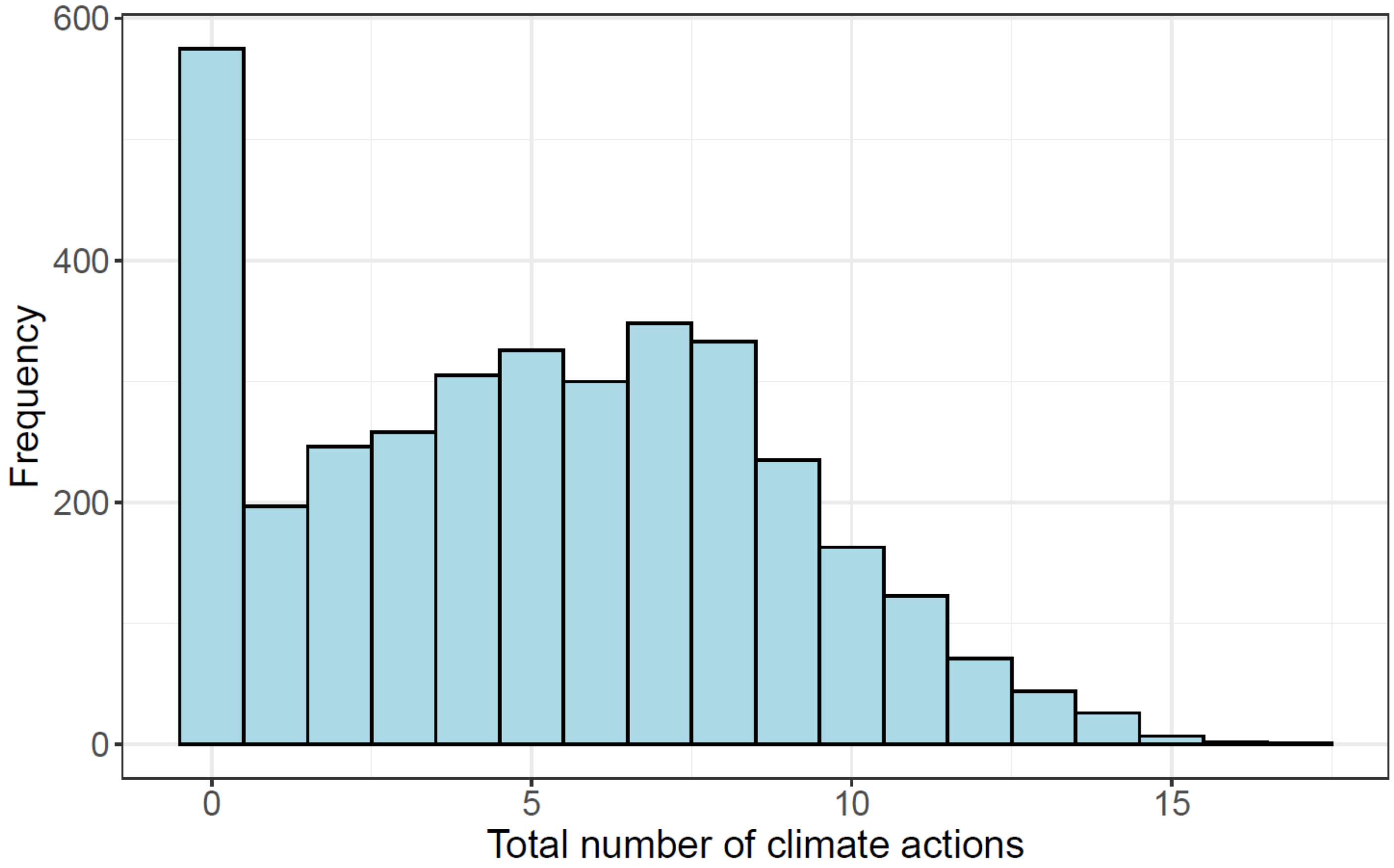

Figure S158: Results of the offspring ordinal regression models with 'belief that the climate is changing' as the outcome for four religious exposures (belief [*n* = 1,100], identity [*n* = 1,099], attendance [*n* = 1,117091 and latent classes [*n* = 1,048]; models are separated by dashed horizontal lines). Odds ratios above 1 indicate an increased belief in climate change. See table S33 for full results.

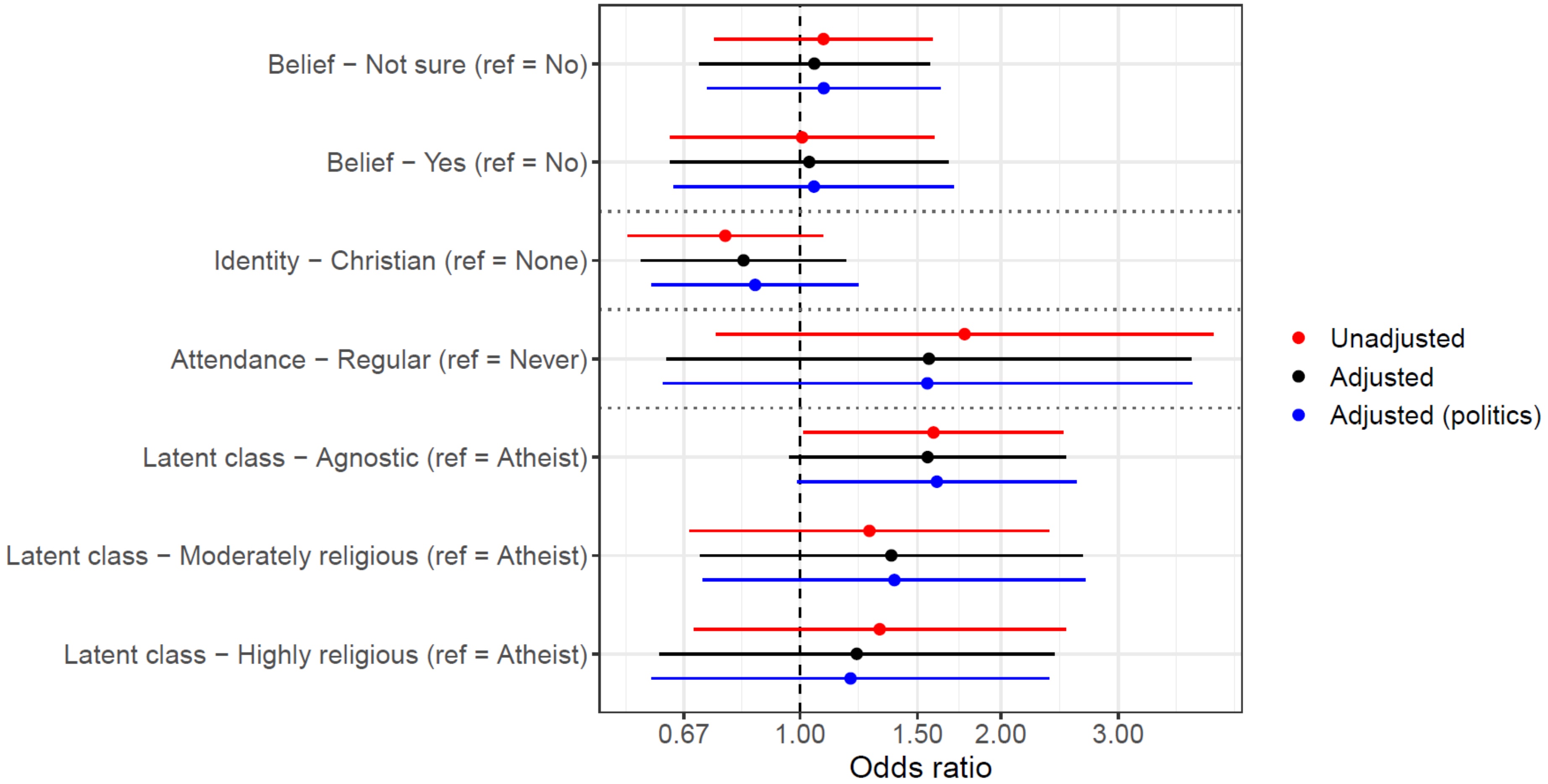

Figure 159: Predicted probabilities of the offspring ordinal regression models with 'belief that the climate is changing' as the outcome for four religious exposures (belief, identity, attendance and latent classes). Results are for the adjusted models excluding political ideology (results including political ideology are practically identical).

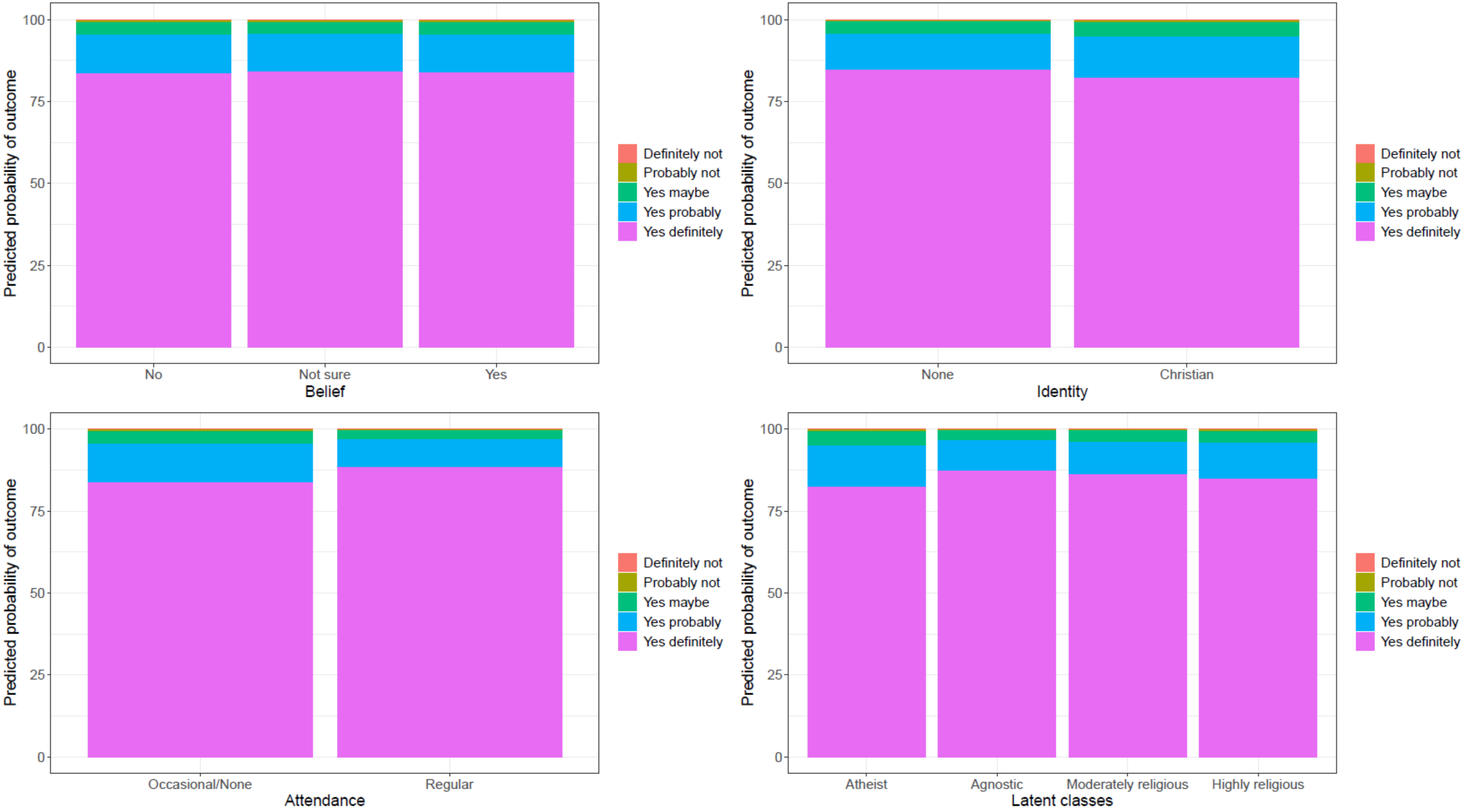

Figure S160: Predicted probabilities of the offspring ordinal regression models with ‘belief that the climate is changing’ as the outcome and the religious identity (with the Christian denominations separated) as the exposure. Results are for the adjusted models excluding political ideology (results including political ideology are practically identical).

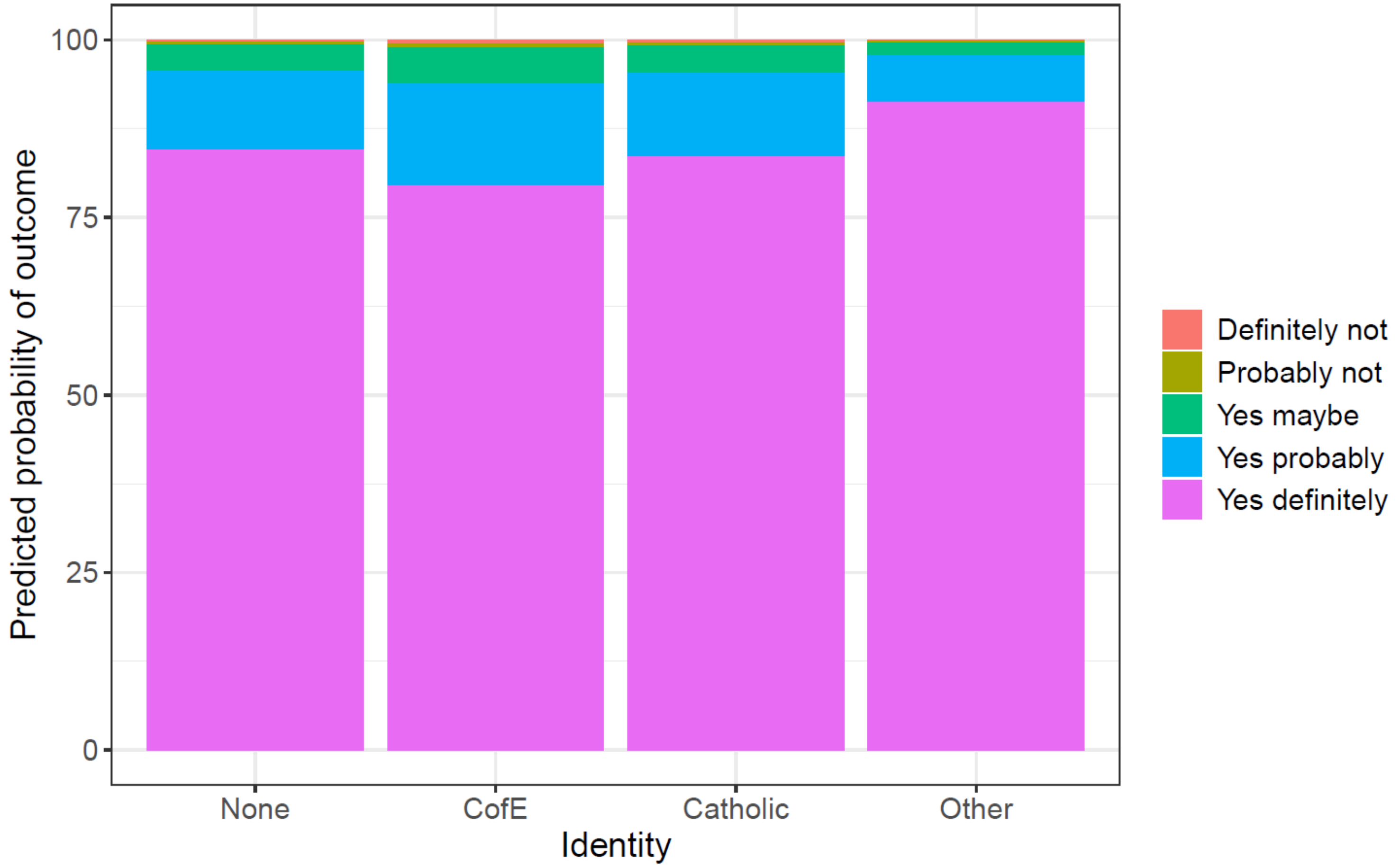

Figure S161: Results of the offspring ordinal regression models with 'concerned about the impact of climate change' as the outcome for four religious exposures (belief [*n* = 1,096], identity [*n* = 1,095], attendance [*n* = 1,088], and latent classes [*n* = 1,045]; models are separated by dashed horizontal lines). Odds ratios above 1 indicate an increased concern regarding climate change. See table S34 for full results.

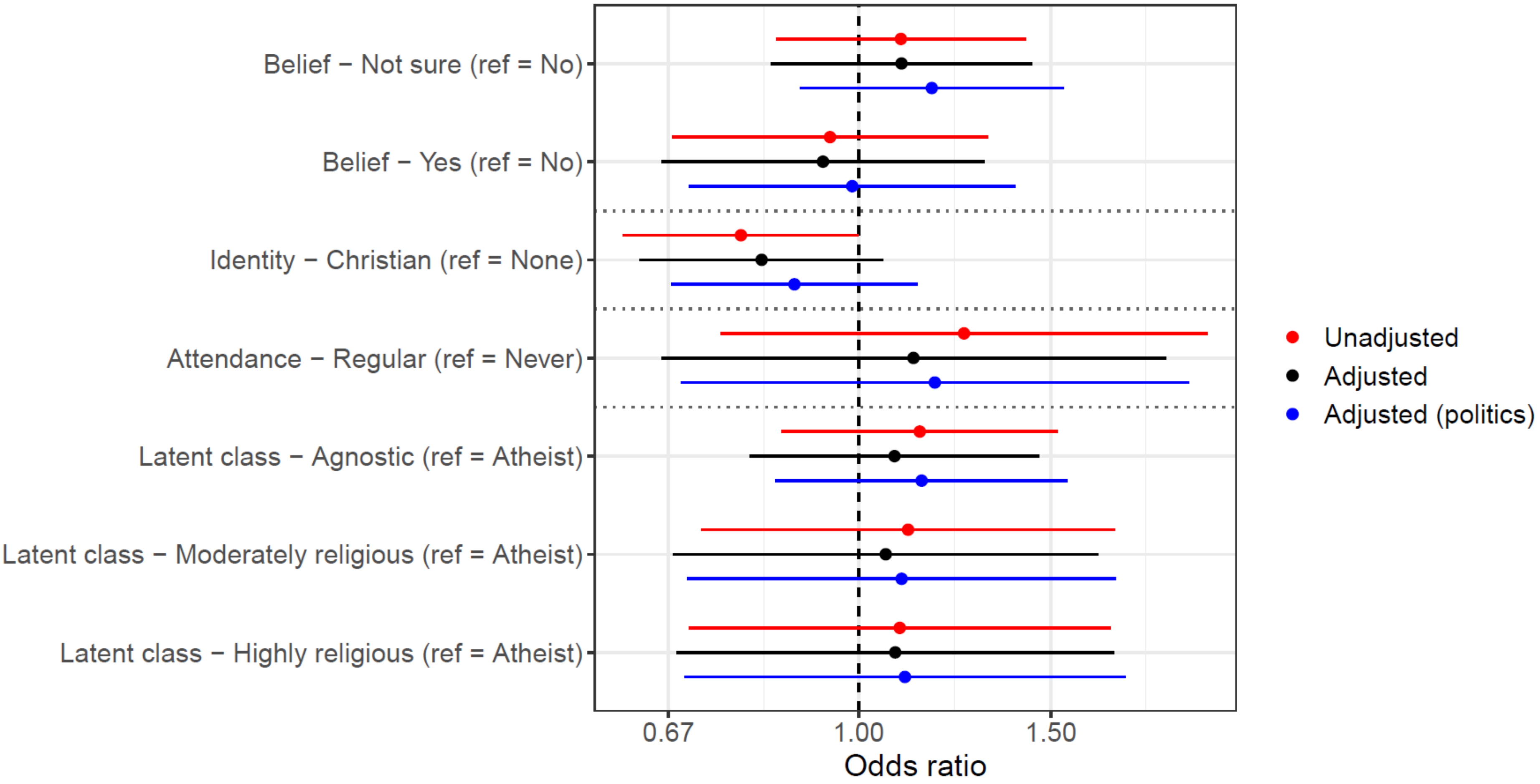

Figure S162: Predicted probabilities of the offspring ordinal regression models with 'concerned about the impact of climate change' as the outcome for four religious exposures (belief, identity, attendance and latent classes). Results are for the adjusted models excluding political ideology (results including political ideology are practically identical).

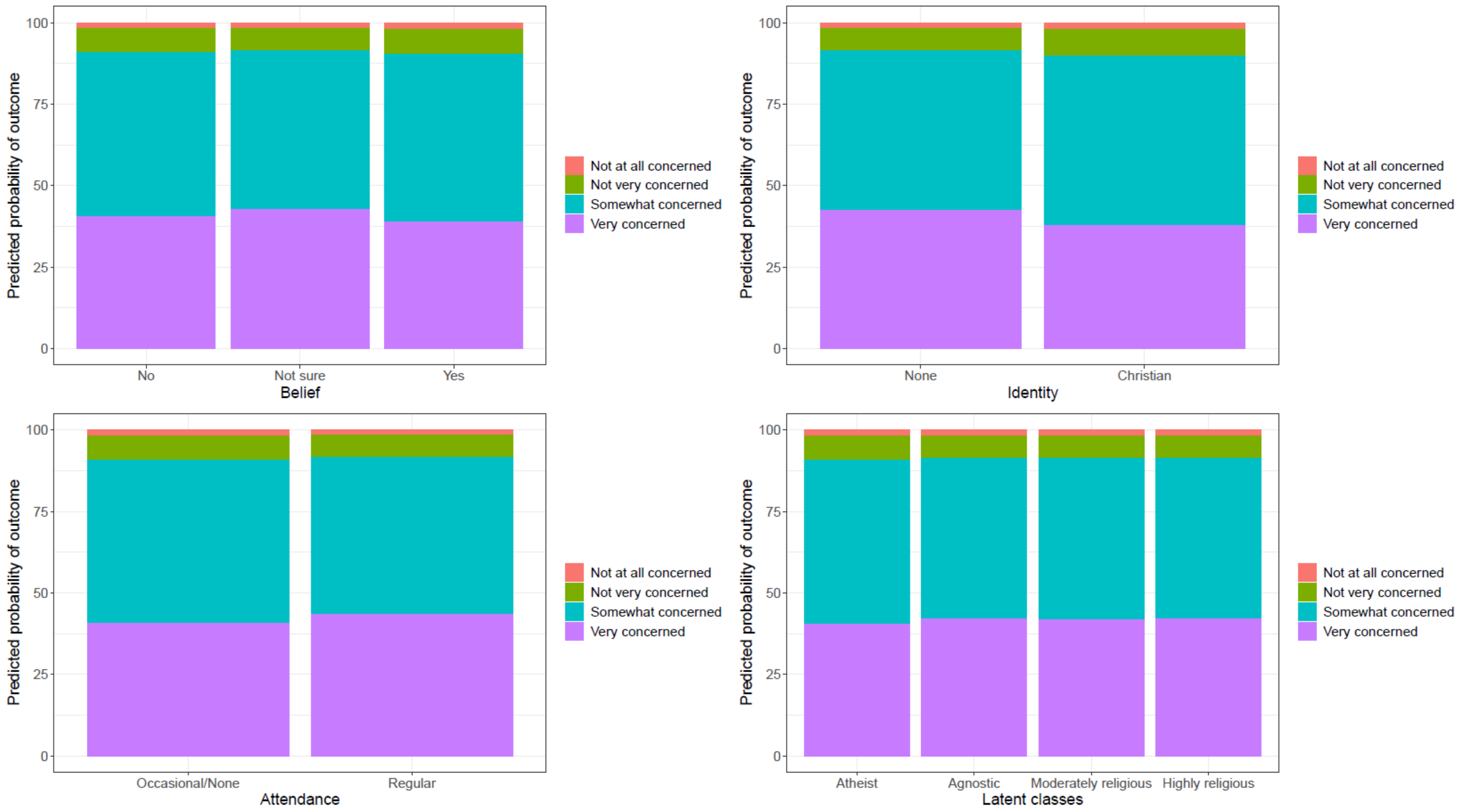

Figure S163: Predicted probabilities of the offspring ordinal regression models with 'concerned about the impact of climate change' as the outcome and the religious identity (with the Christian denominations separated) as the exposure. Results are for the adjusted models excluding political ideology (results including political ideology are practically identical).

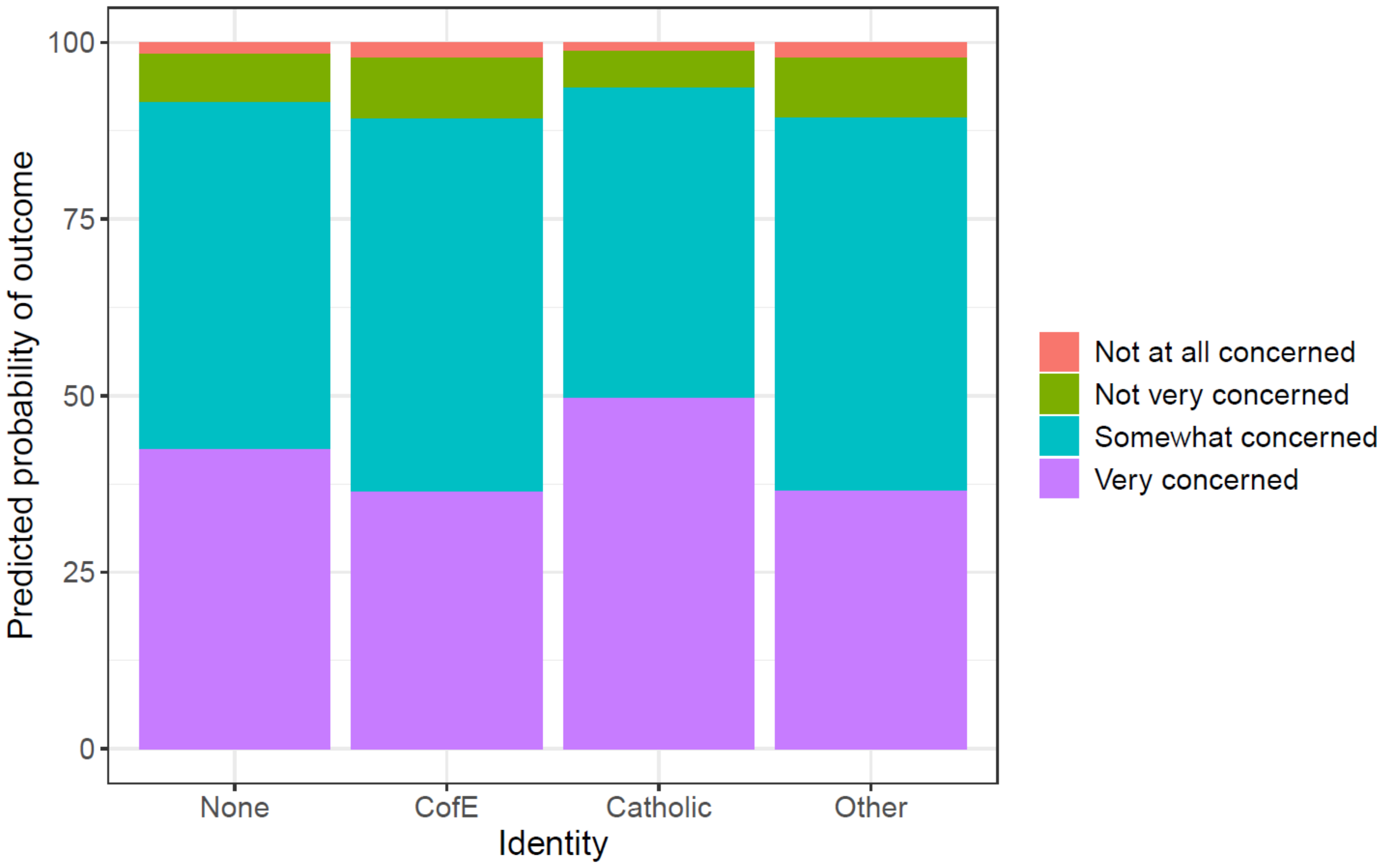

Figure S164: Results of the offspring ordinal regression models with 'believes that humans are to blame for climate change' as the outcome for four religious exposures (belief [*n* = 1,095], identity [*n* = 1,094], attendance [*n* = 1,087], and latent classes [*n* = 1,045]; models are separated by dashed horizontal lines). Odds ratios above 1 indicate an increased belief that humans are to blame for climate change. See table S35 for full results.

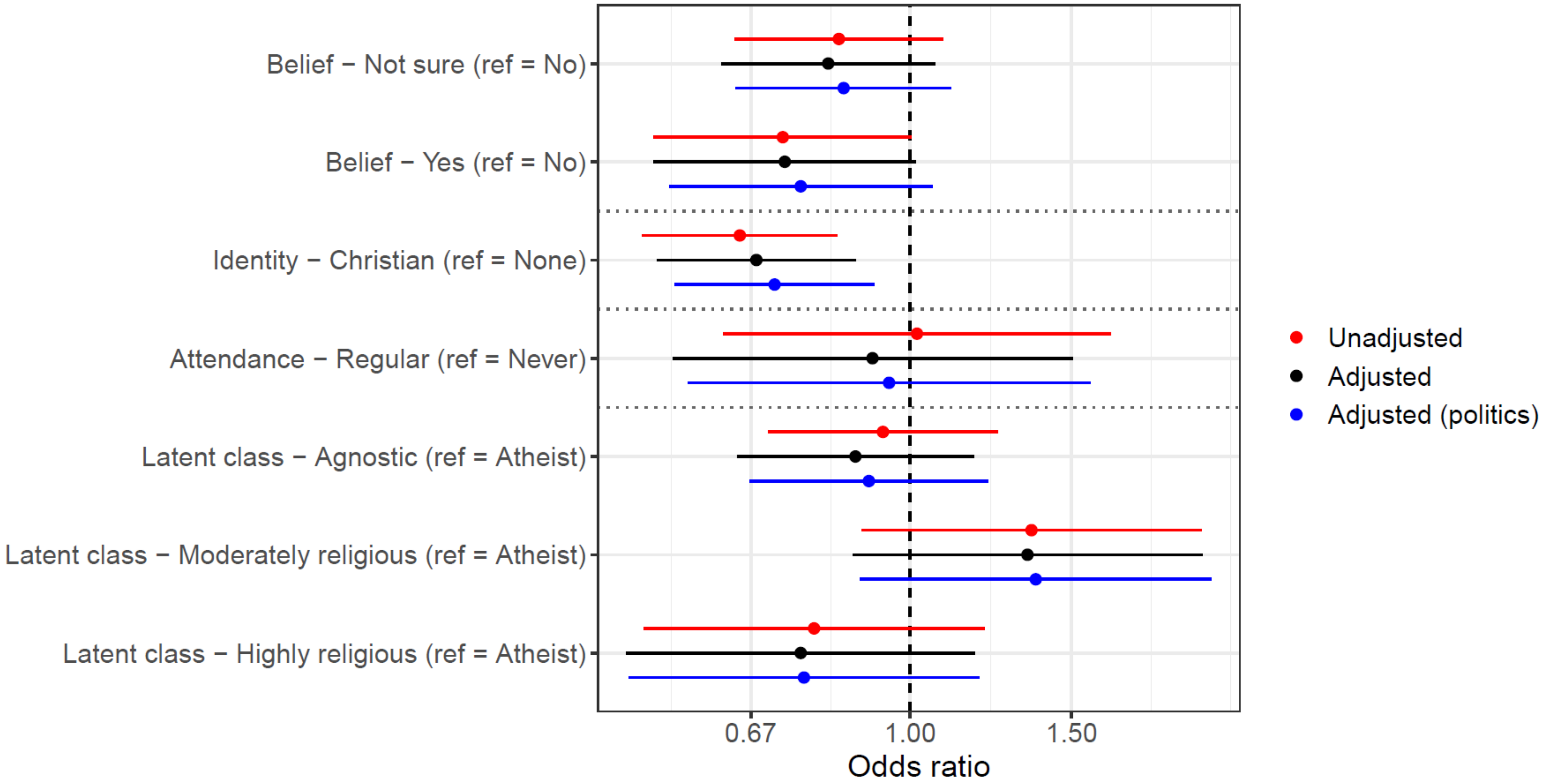

Figure S165: Predicted probabilities of the offspring ordinal regression models with 'believes that humans are to blame for climate change' as the outcome for four religious exposures (belief, identity, attendance and latent classes). Results are for the adjusted models excluding political ideology (results including political ideology are practically identical).

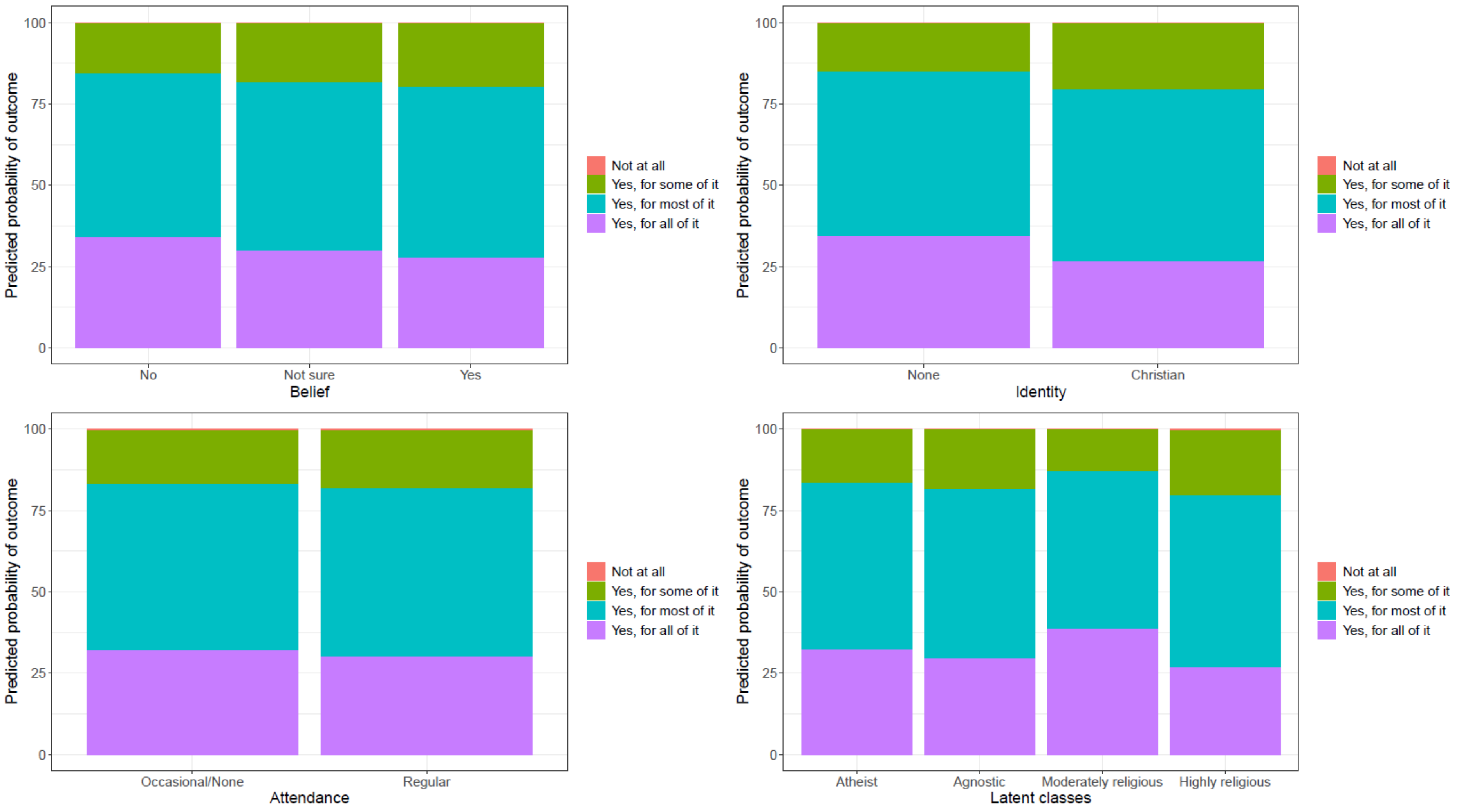

Figure S166: Predicted probabilities of the offspring ordinal regression models with 'believes that humans are to blame for climate change' as the outcome and the religious identity (with the Christian denominations separated) as the exposure. Results are for the adjusted models excluding political ideology (results including political ideology are practically identical).

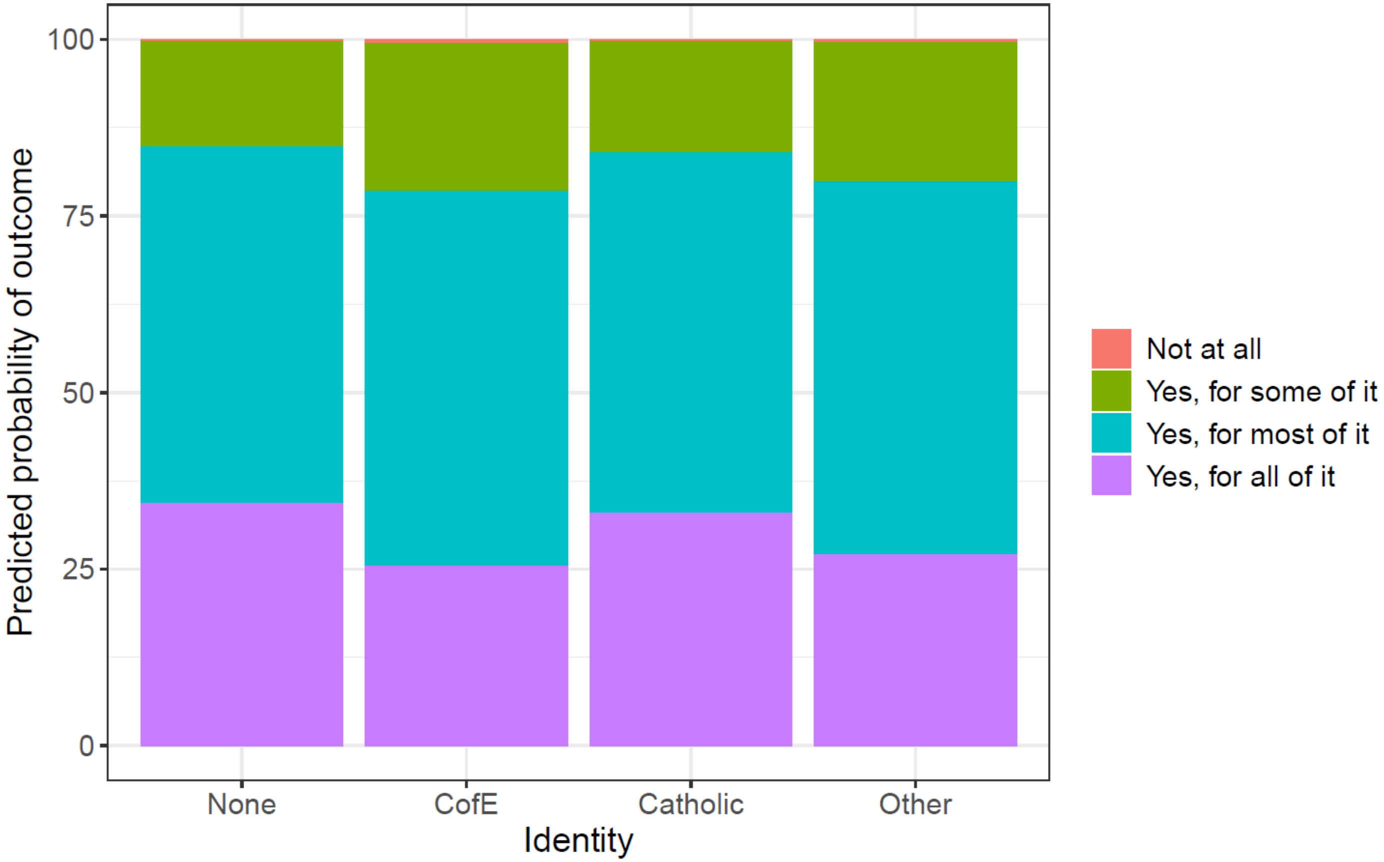

Figure S167: Results of the offspring multinomial regression models with 'thinks that personal actions will make a difference to long-term climate change' as the outcome for four religious exposures (belief [*n* = 1,095], identity [*n* = 1,094], attendance [*n* = 1,087], and latent classes [*n* = 1,045]; models are separated by dashed horizontal lines). See table S36 for full results.

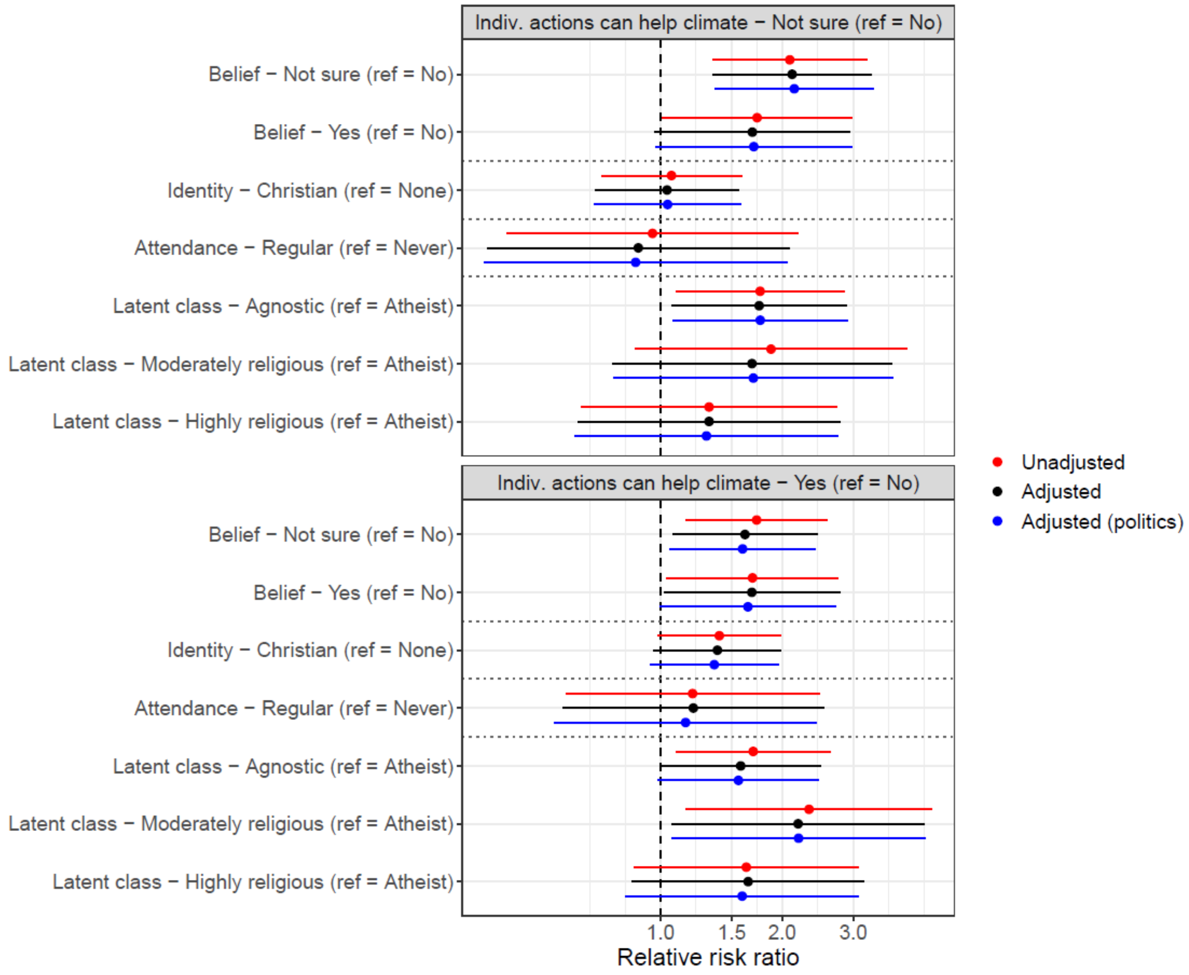

Figure S168: Predicted probabilities of the offspring multinomial regression models with ‘thinks that personal actions will make a difference to long-term climate change’ as the outcome for four religious exposures (belief, identity, attendance and latent classes). Results are for the adjusted models excluding political ideology (results including political ideology are practically identical).

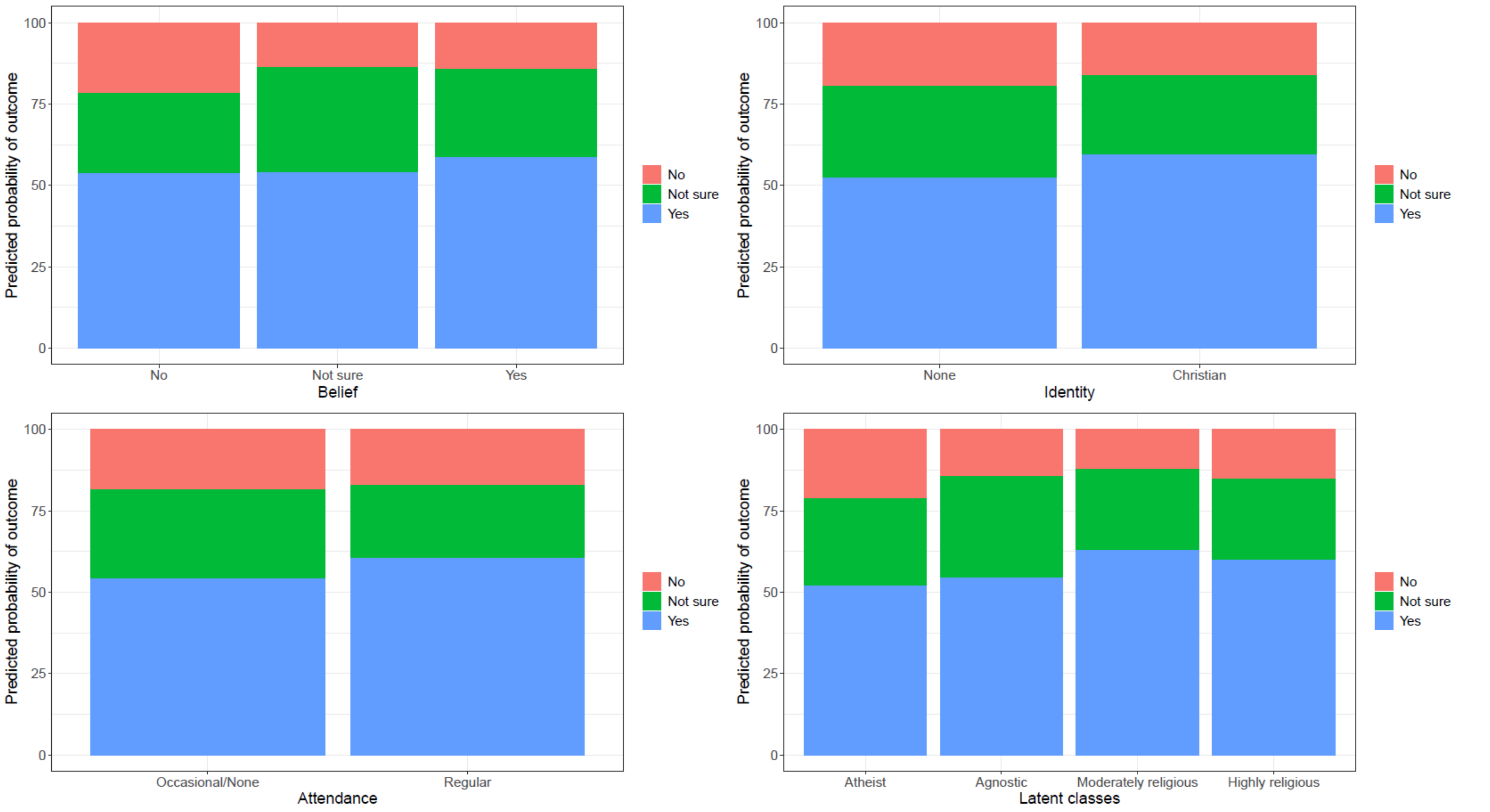

Figure S169: Predicted probabilities of the offspring multinomial regression models with 'thinks that personal actions will make a difference to long-term climate change' as the outcome and the religious identity (with the Christian denominations separated) as the exposure. Results are for the adjusted models excluding political ideology (results including political ideology are practically identical).

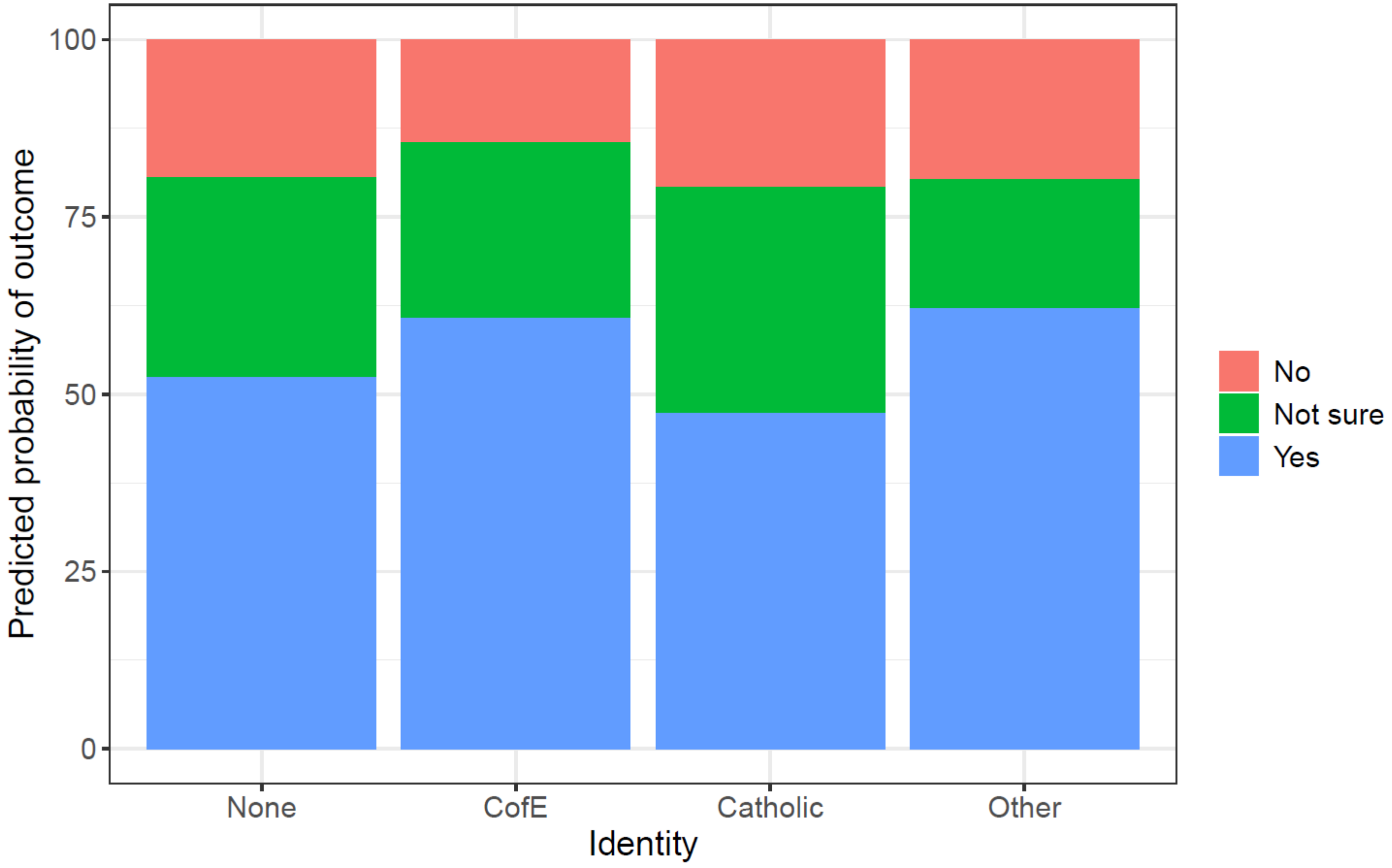

Figure S170: Results of the offspring linear regression models with 'total number of actions performed due to climate change' as the outcome for four religious exposures (belief [*n* = 983], identity [*n* = 982], attendance [*n* = 975], and latent classes [*n* = 940]; models are separated by dashed horizontal lines). Values above 0 indicate an increased number of pro-environmental actions performed. See table S37 for full results.

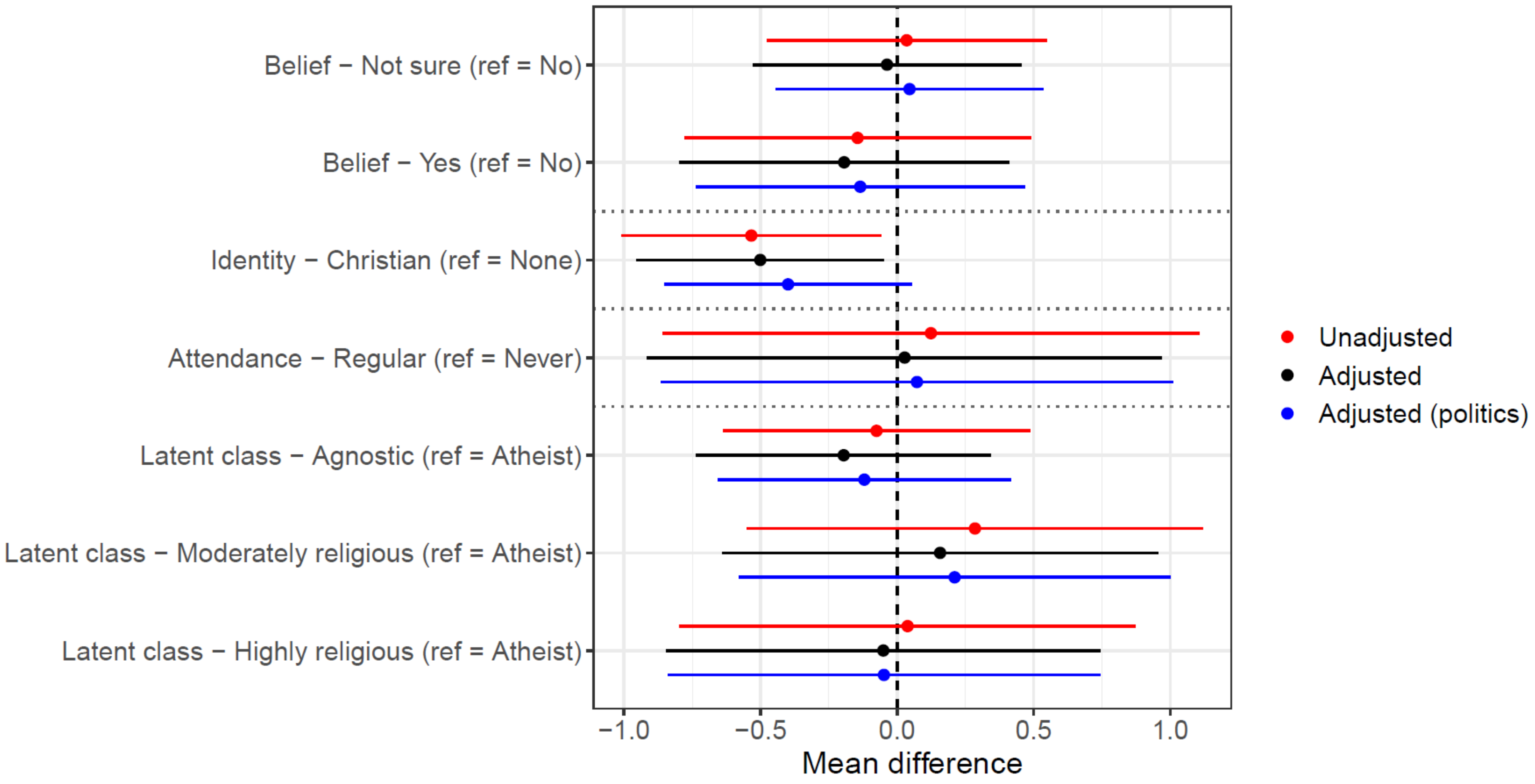

Figure S171: Predicted total number of actions performed due to climate change for four religious exposures (belief, identity, attendance and latent classes) based on the offspring linear regression models. Results are for the adjusted models excluding political ideology (results including political ideology are practically identical).

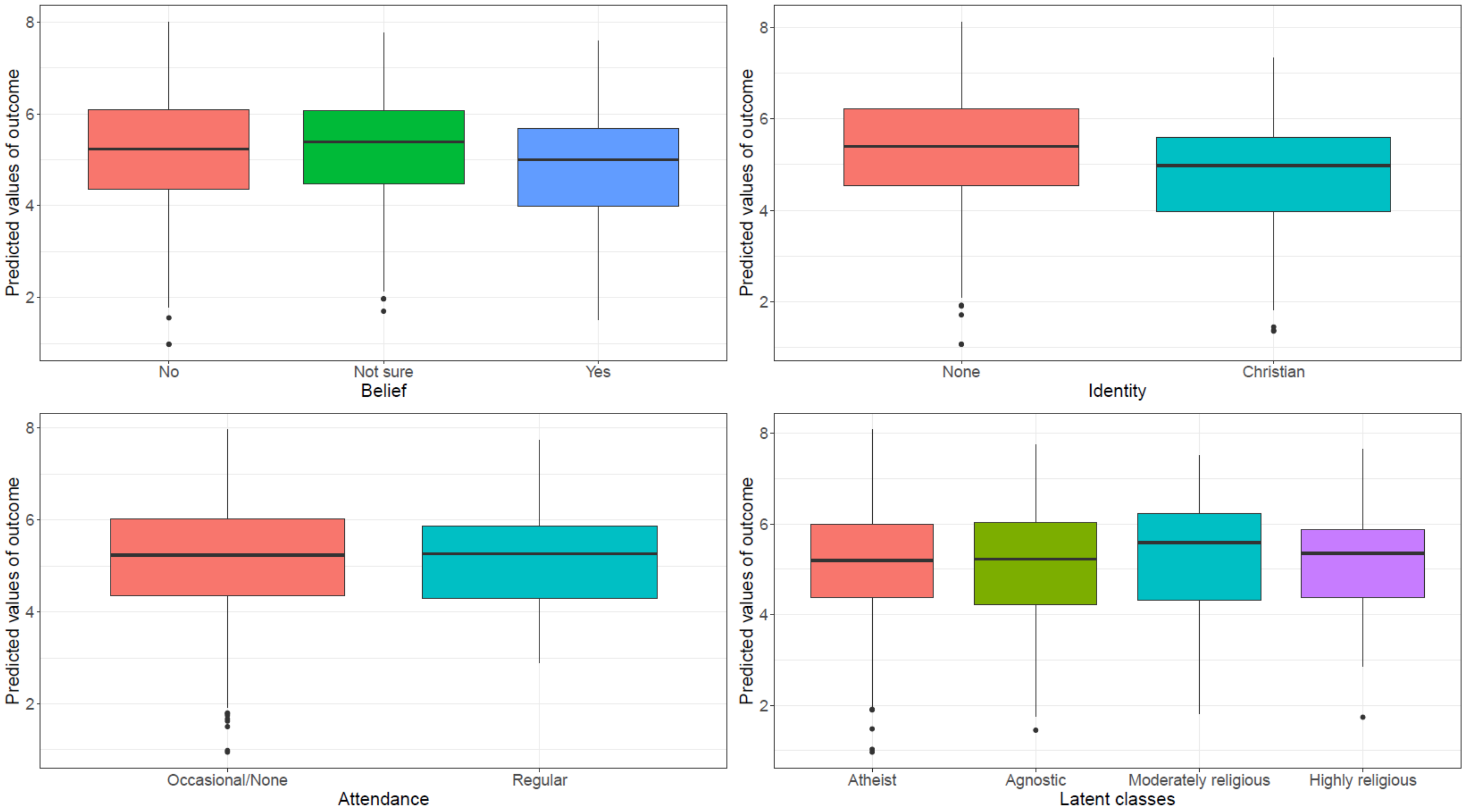

Figure S172: Predicted total number of actions performed due to climate change for the religious identity (with the Christian denominations separated) as the exposure based on the offspring linear regression models. Results are for the adjusted models excluding political ideology (results including political ideology are practically identical).

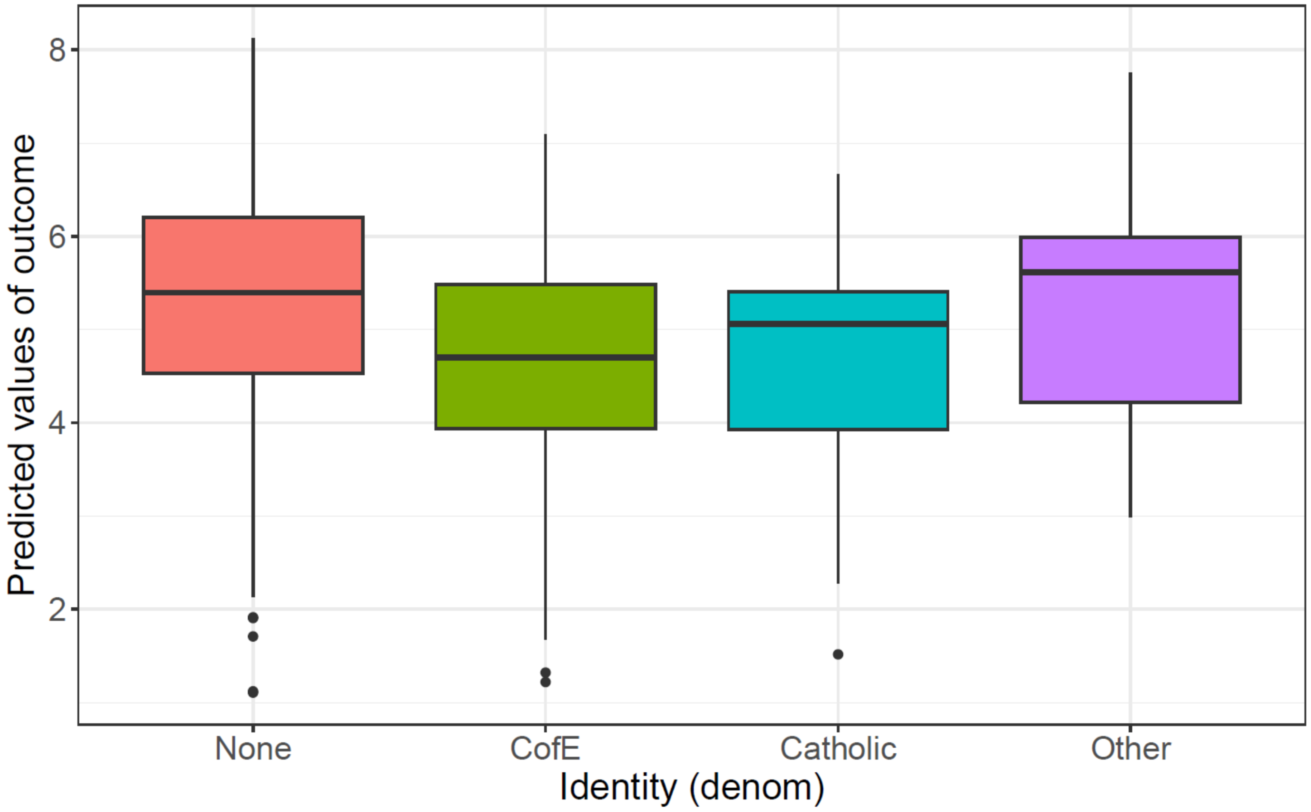

Figure S173: Results of the offspring Poisson regression models with 'total number of actions performed due to climate change' as the outcome for four religious exposures (belief [*n* = 983], identity [*n* = 982], attendance [*n* = 975], and latent classes [*n* = 940]; models are separated by dashed horizontal lines). Incidence rate ratios above 1 indicate an increased number of pro-environmental actions performed. See table S38 for full results.

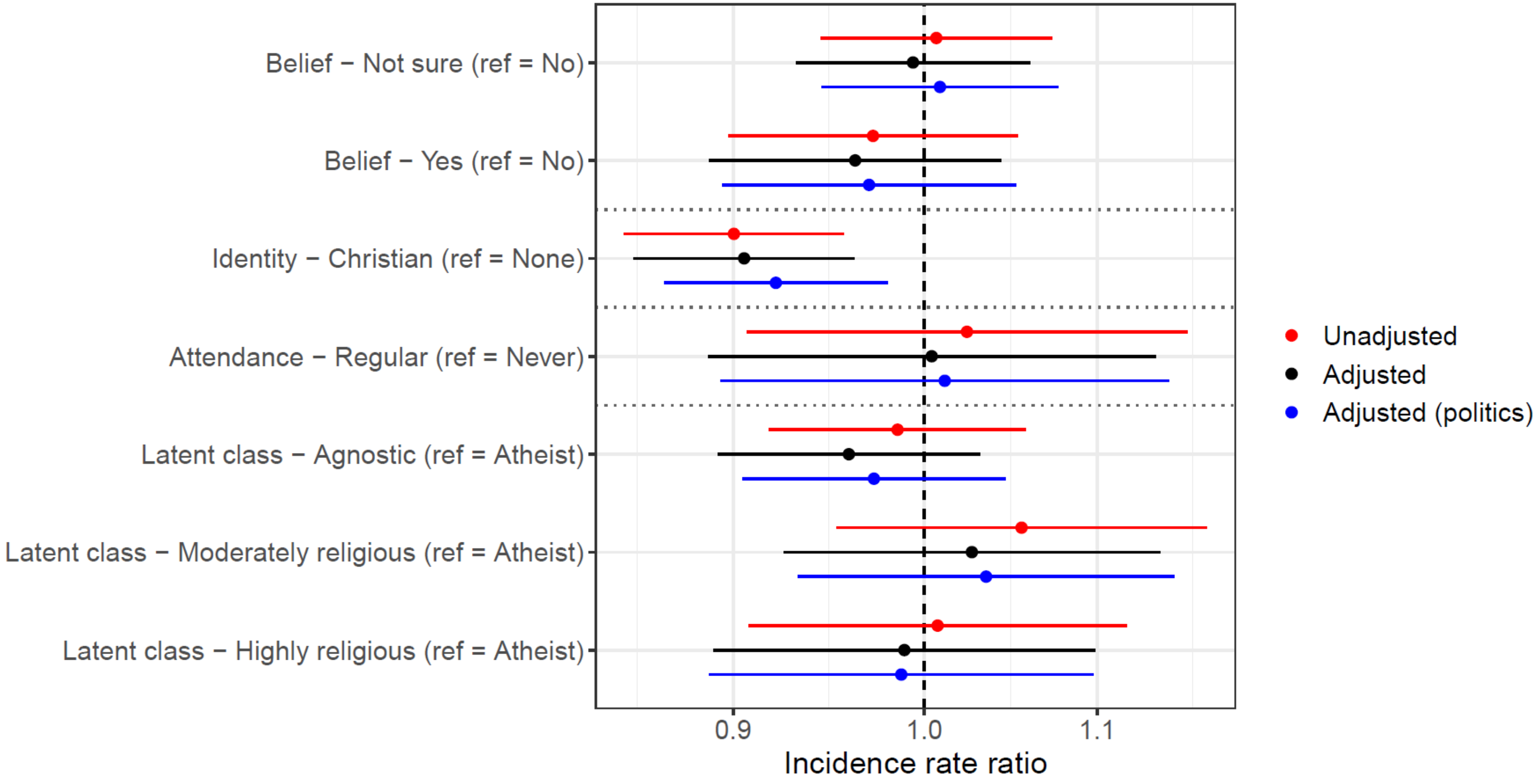

Figure S174: Predicted total number of actions performed due to climate change for four religious exposures (belief, identity, attendance and latent classes) based on the offspring Poisson regression models. Results are for the adjusted models excluding political ideology (results including political ideology are practically identical).

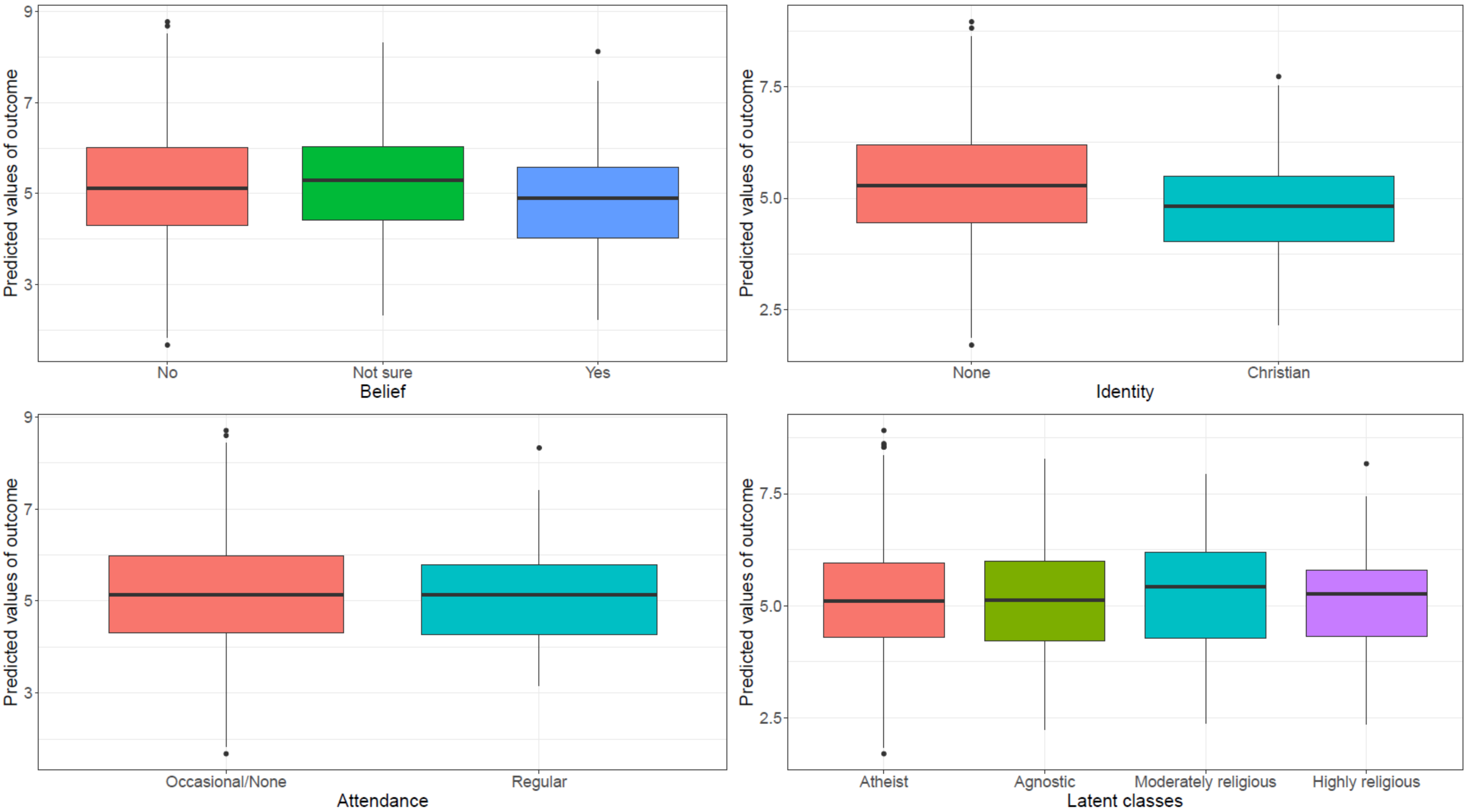

Figure S175: Predicted total number of actions performed due to climate change for the religious identity (with the Christian denominations separated) as the exposure based on the offspring Poisson regression models. Results are for the adjusted models excluding political ideology (results including political ideology are practically identical).

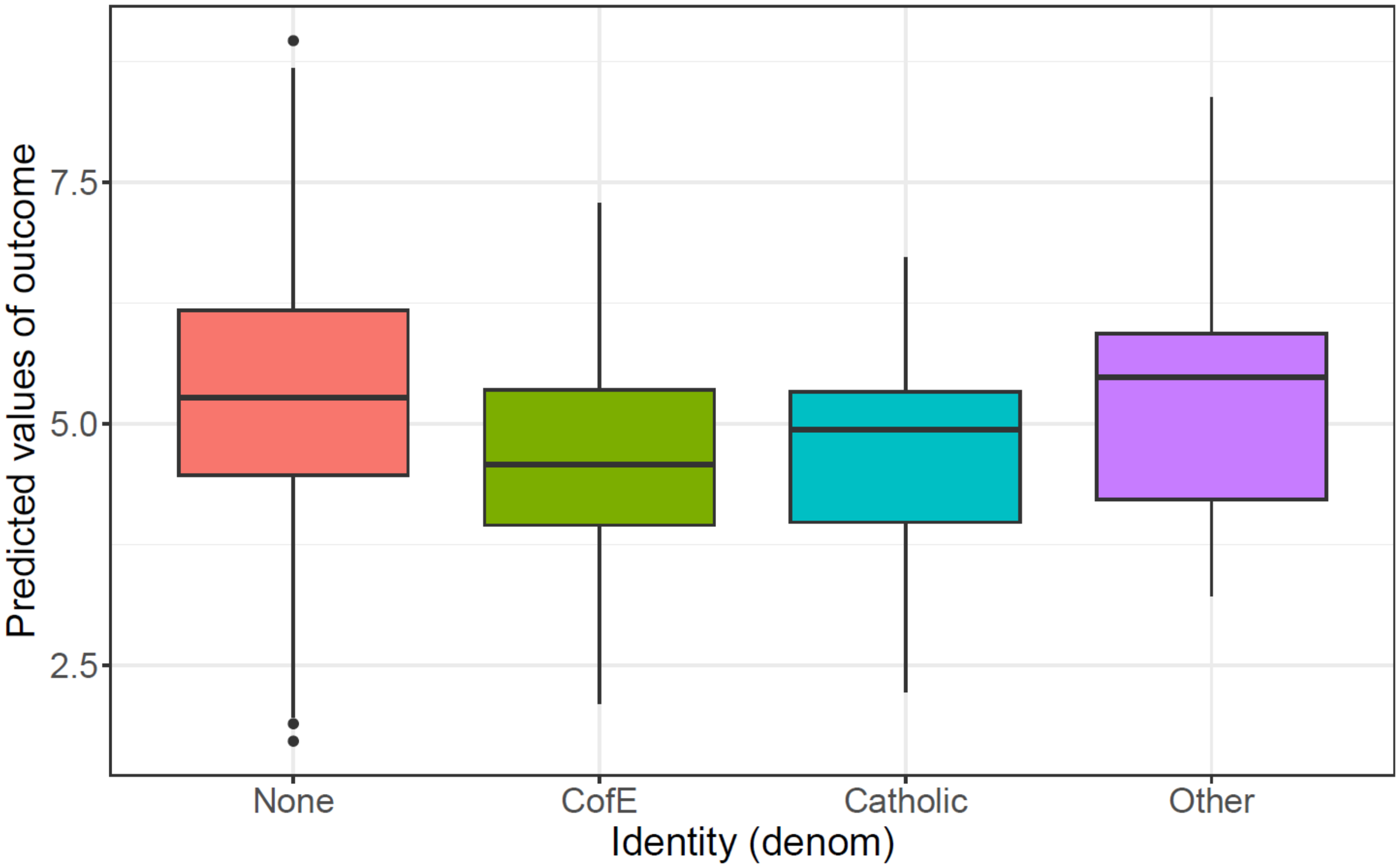

Figure S176: Results of the offspring zero-inflated Poisson regression models with ‘total number of actions performed due to climate change’ as the outcome for four religious exposures (belief [*n* = 983], identity [*n* = 982], attendance [*n* = 975], and latent classes [*n* = 940]; models are separated by dashed horizontal lines). Incidence rate ratios above 1 indicate an increased number of pro-environmental actions performed, while odds ratios above 1 indicate an excess of zeros. See table S39 for full results.

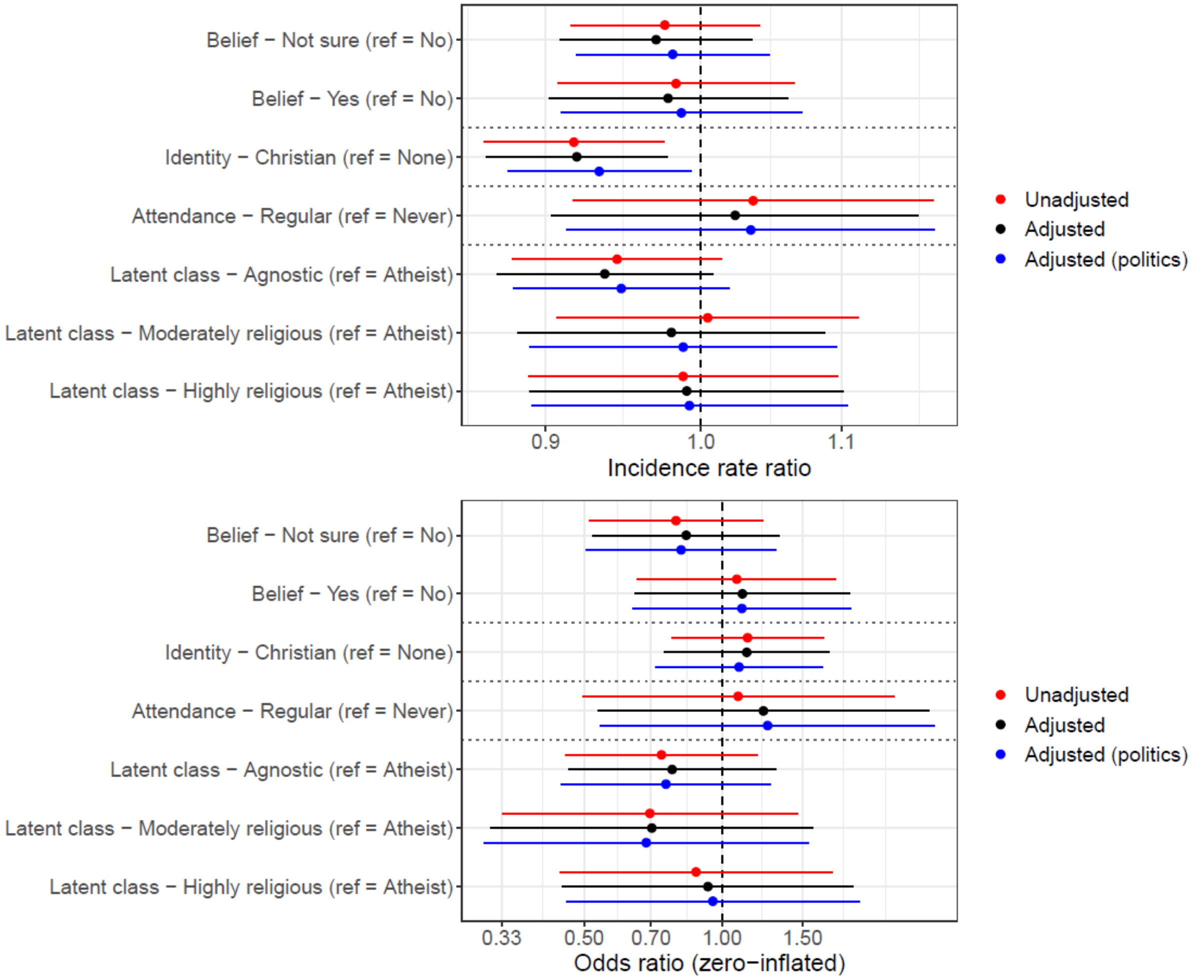

Figure S177: Predicted total number of actions performed due to climate change for four religious exposures (belief, identity, attendance and latent classes) based on the offspring zero-inflated Poisson regression models. Results are for the adjusted models excluding political ideology (results including political ideology are practically identical).

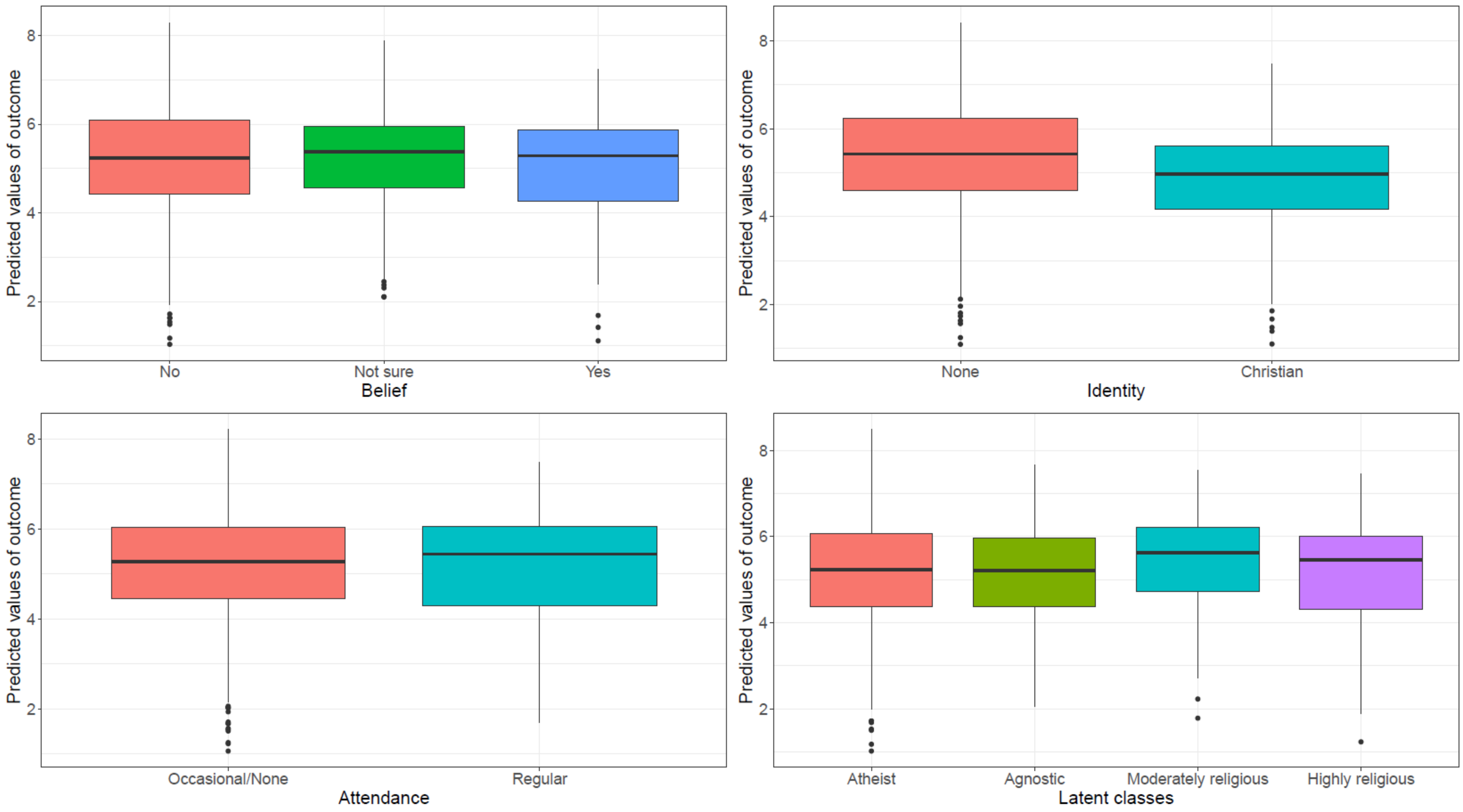

Figure S178: Predicted total number of actions performed due to climate change for the religious identity (with the Christian denominations separated) as the exposure based on the offspring zero-inflated Poisson regression models. Results are for the adjusted models excluding political ideology (results including political ideology are practically identical).

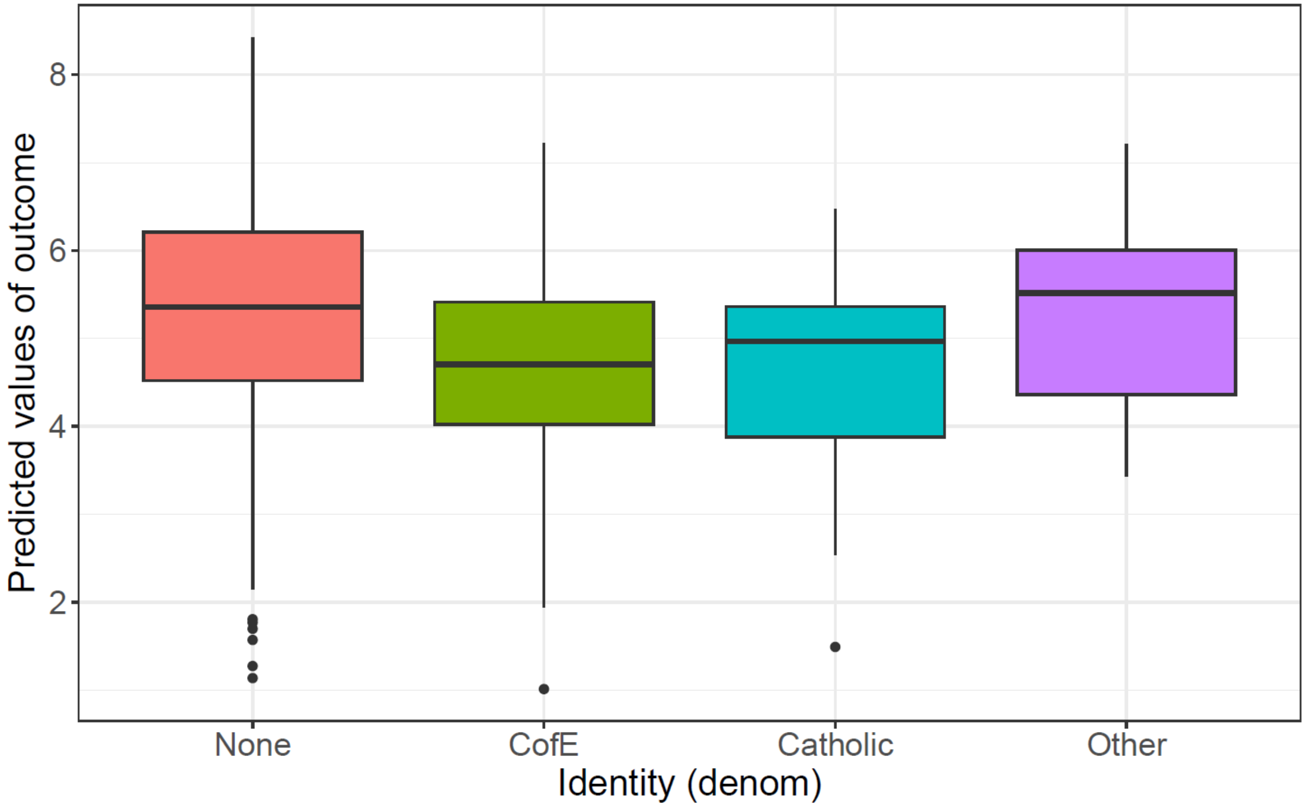

Figure S179: Results of the offspring linear regression models with 'total number of actions performed due to climate change (excluding ones which may be prohibitively costly)' as the outcome for four religious exposures (belief [*n* = 995], identity [*n* = 994], attendance [*n* = 987], and latent classes [*n* = 952]; models are separated by dashed horizontal lines). Values above 0 indicate an increased number of pro-environmental actions performed. See table S40 for full results.

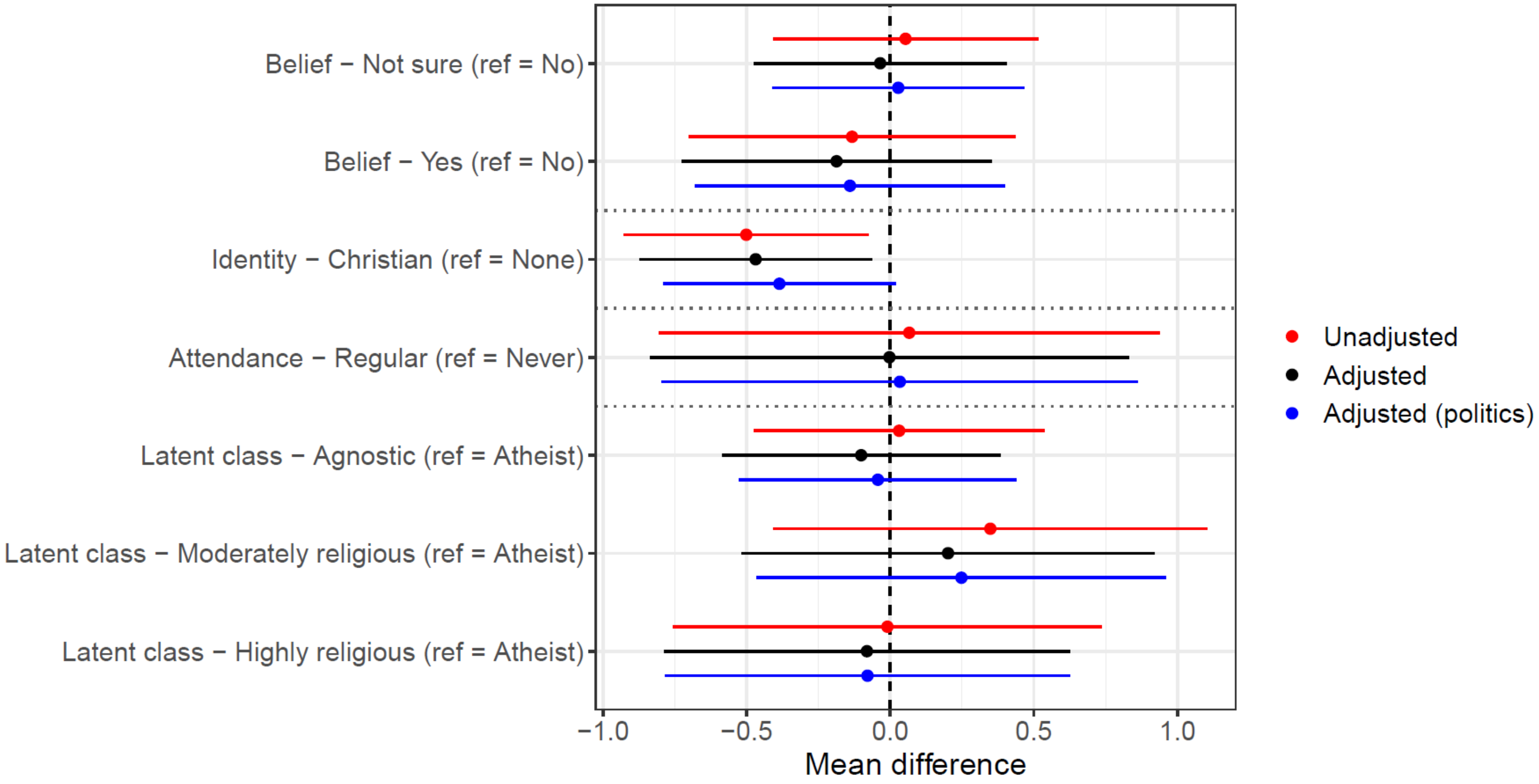

Figure S180: Predicted total number of actions (excluding ones which may be prohibitively costly) performed due to climate change for four religious exposures (belief, identity, attendance and latent classes) based on the offspring linear regression models. Results are for the adjusted models excluding political ideology (results including political ideology are practically identical).

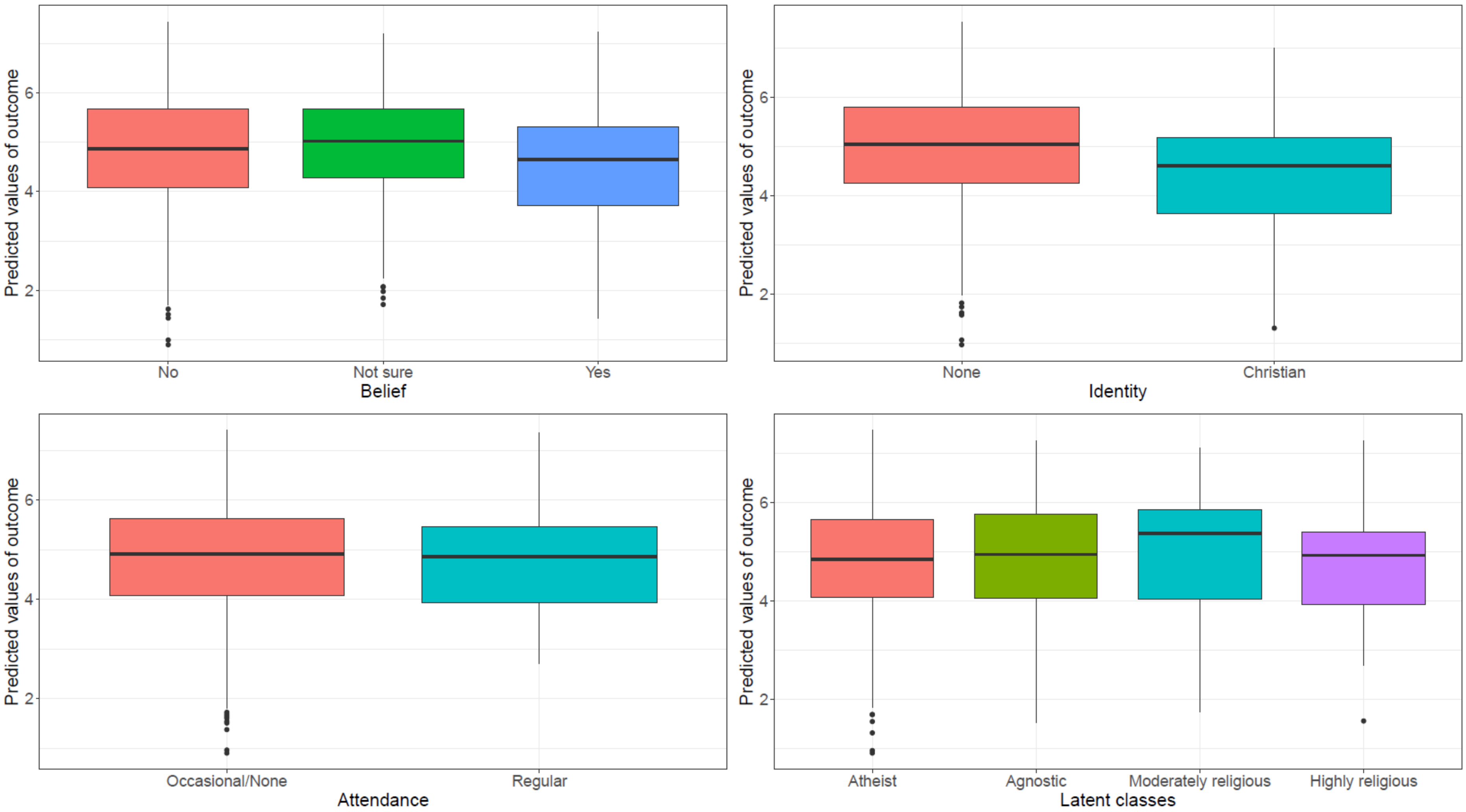

Figure S181: Predicted total number of actions (excluding ones which may be prohibitively costly) performed due to climate change for the religious identity (with the Christian denominations separated) as the exposure based on the offspring linear regression models. Results are for the adjusted models excluding political ideology (results including political ideology are practically identical).

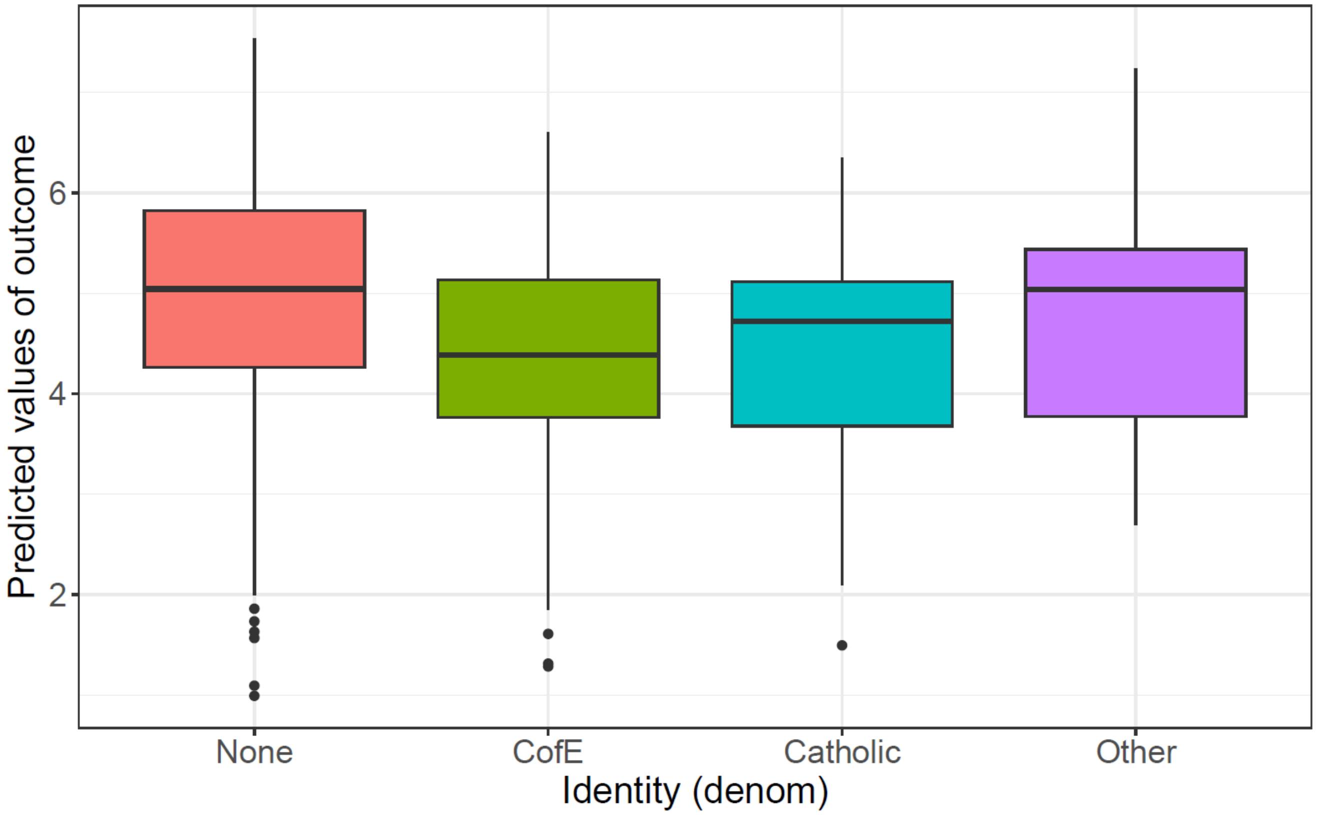

Figure S182: Results of the offspring Poisson regression models with 'total number of actions performed due to climate change (excluding ones which may be prohibitively costly)' as the outcome for four religious exposures (belief [*n* = 995], identity [*n* = 994], attendance [*n* = 987], and latent classes [*n* = 952]; models are separated by dashed horizontal lines). Incidence rate ratios above 1 indicate an increased number of pro-environmental actions performed. See table S41 for full results.

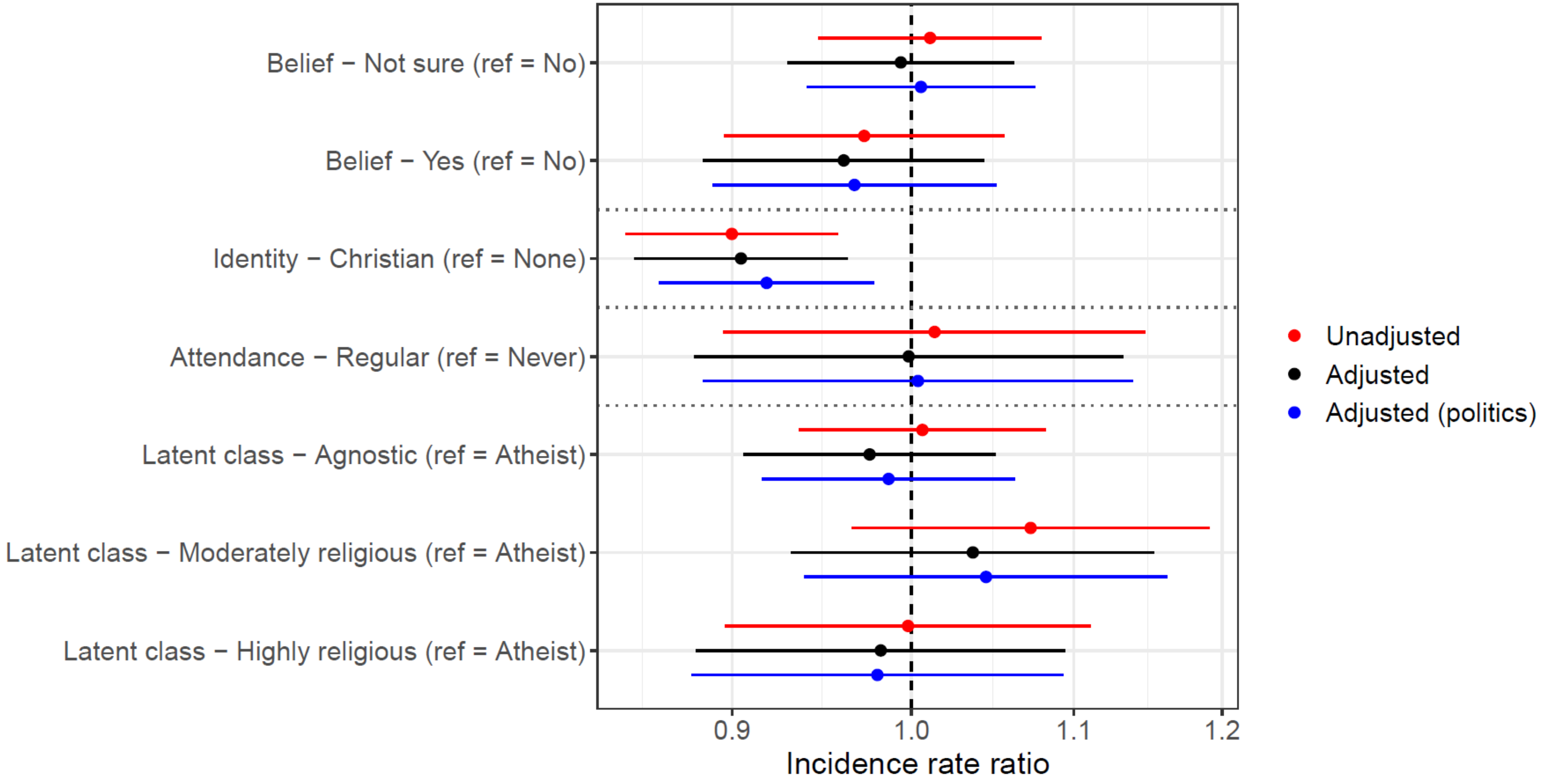

Figure S183: Predicted total number of actions (excluding ones which may be prohibitively costly) performed due to climate change for four religious exposures (belief, identity, attendance and latent classes) based on the offspring Poisson regression models. Results are for the adjusted models excluding political ideology (results including political ideology are practically identical).

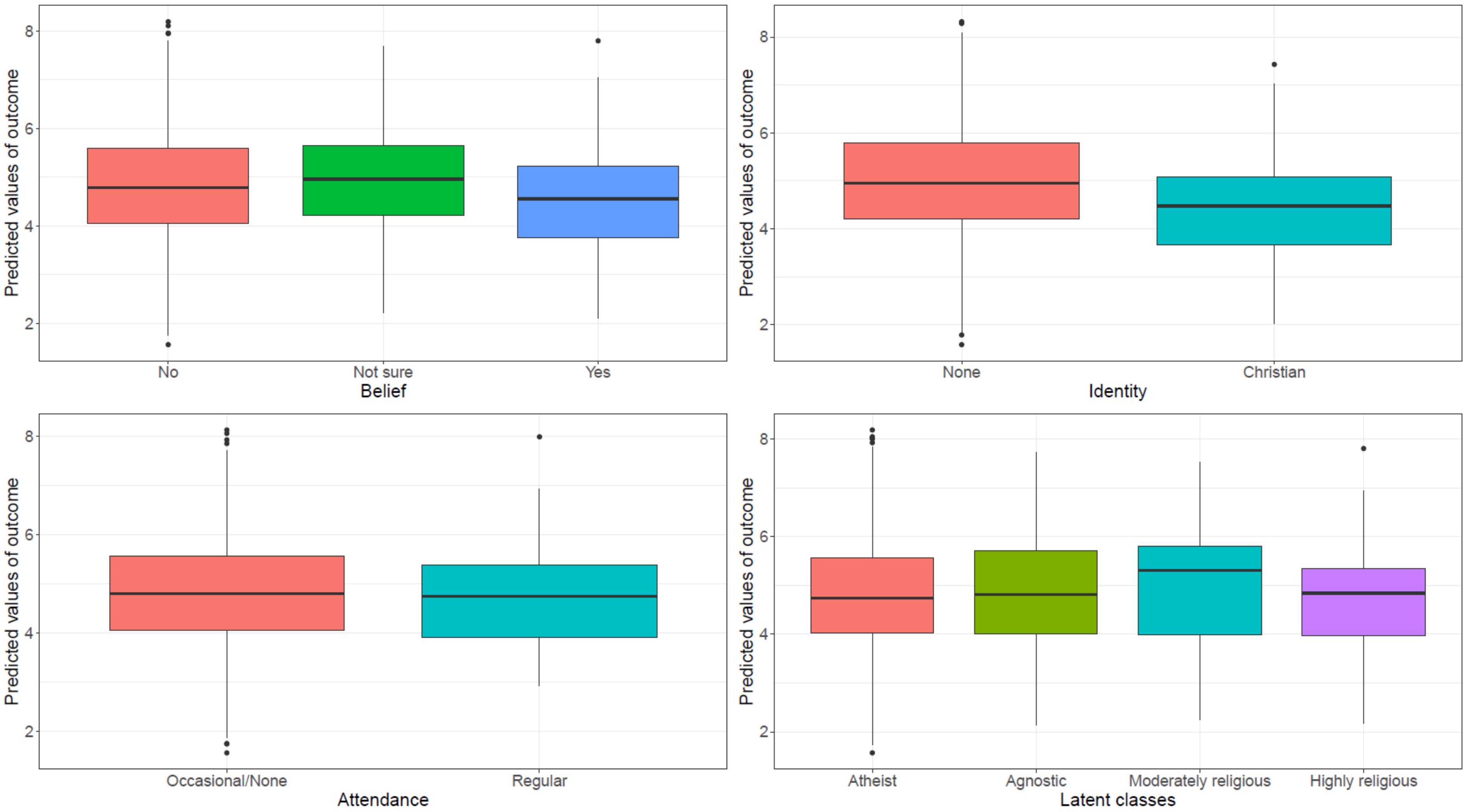

Figure S184: Predicted total number of actions (excluding ones which may be prohibitively costly) performed due to climate change for the religious identity (with the Christian denominations separated) as the exposure based on the offspring Poisson regression models. Results are for the adjusted models excluding political ideology (results including political ideology are practically identical).

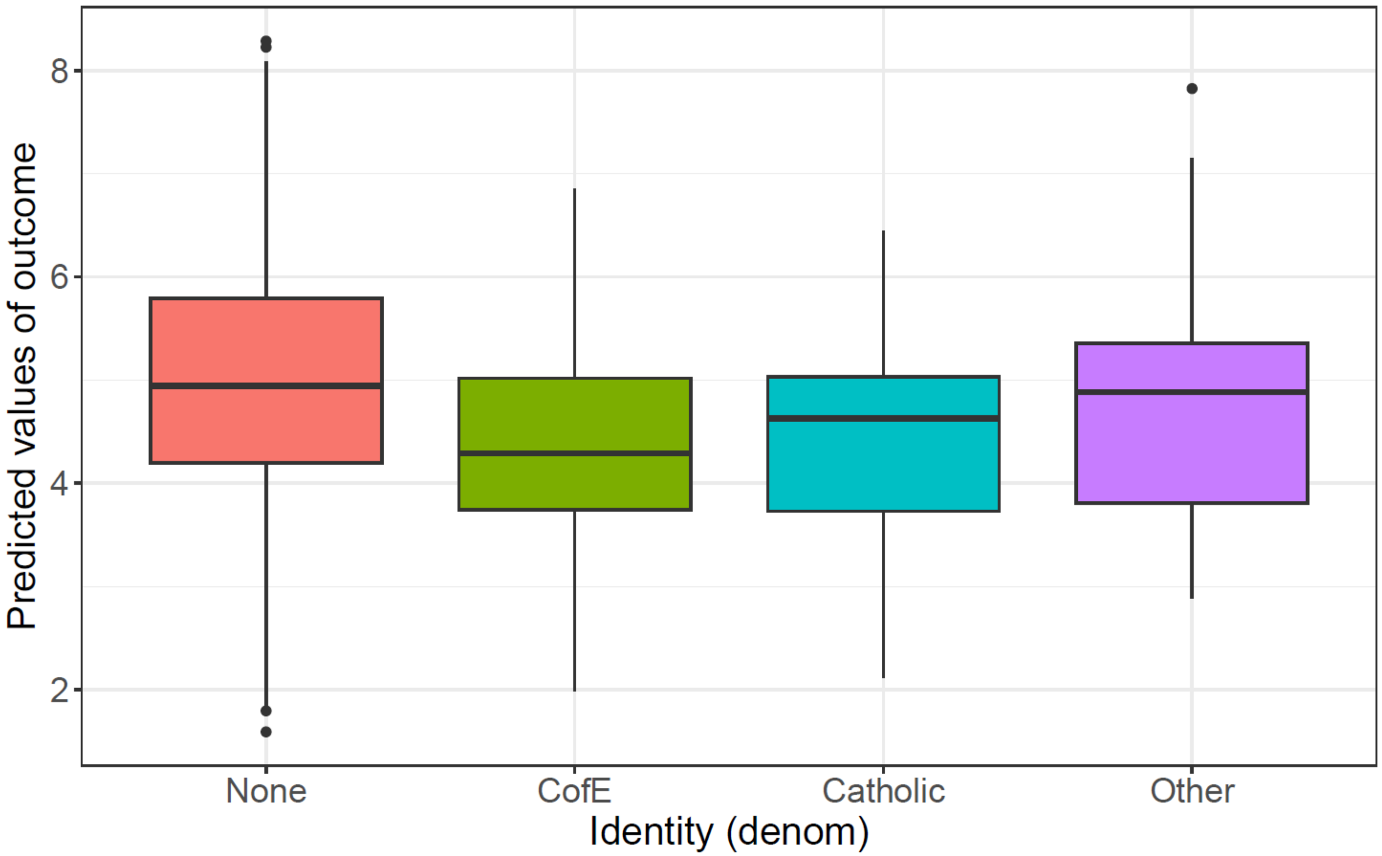

Figure S185: Results of the offspring zero-inflated Poisson regression models with 'total number of actions performed due to climate change (excluding ones which may be prohibitively costly)' as the outcome for four religious exposures (belief [*n* = 995], identity [*n* = 994], attendance [*n* = 987], and latent classes [*n* = 952]; models are separated by dashed horizontal lines). Incidence rate ratios above 1 indicate an increased number of pro-environmental actions performed, while odds ratios above 1 indicate an excess of zeros. See table S42 for full results.

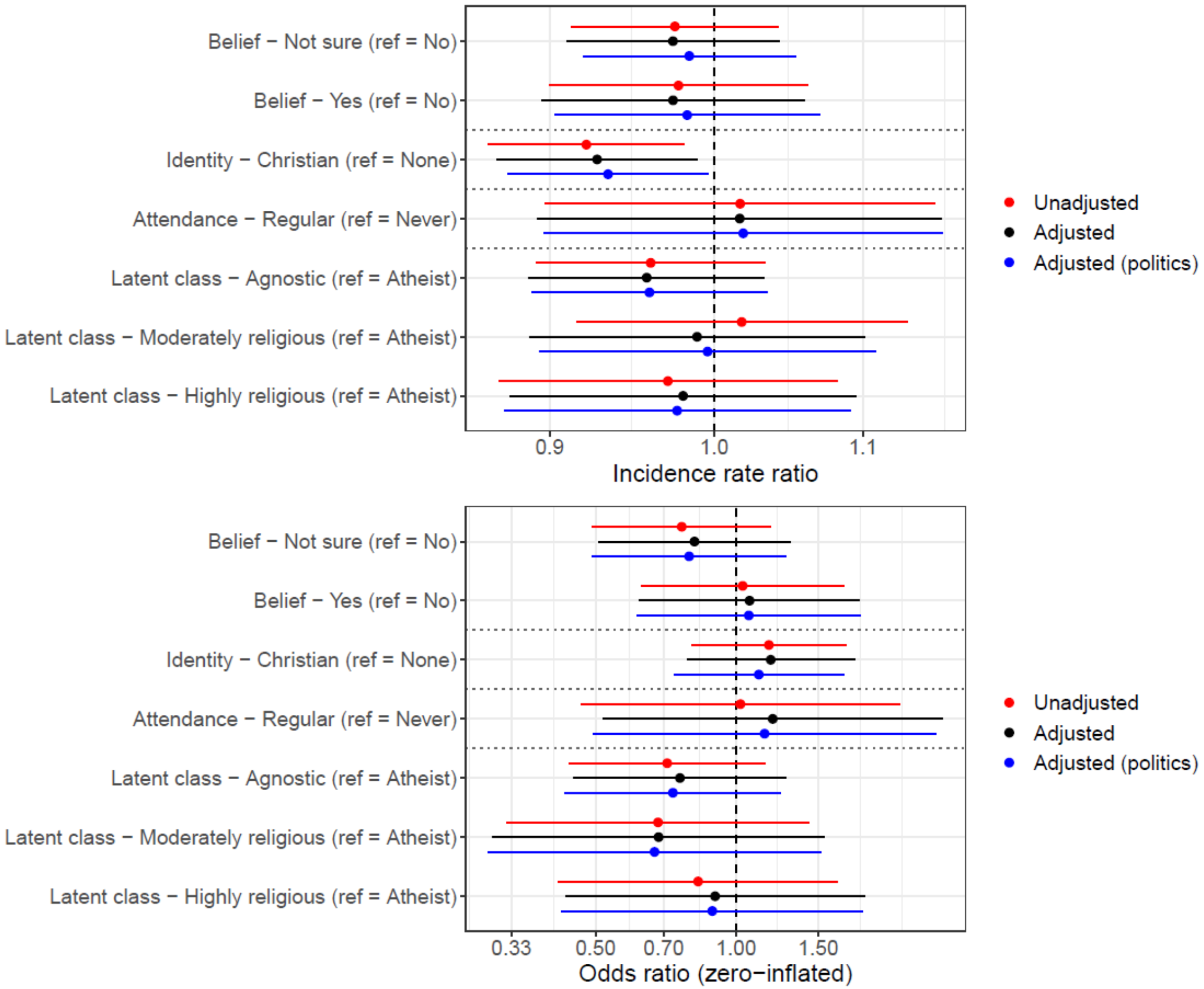

Figure S186: Predicted total number of actions (excluding ones which may be prohibitively costly) performed due to climate change for four religious exposures (belief, identity, attendance and latent classes) based on the offspring zero-inflated Poisson regression models. Results are for the adjusted models excluding political ideology (results including political ideology are practically identical).

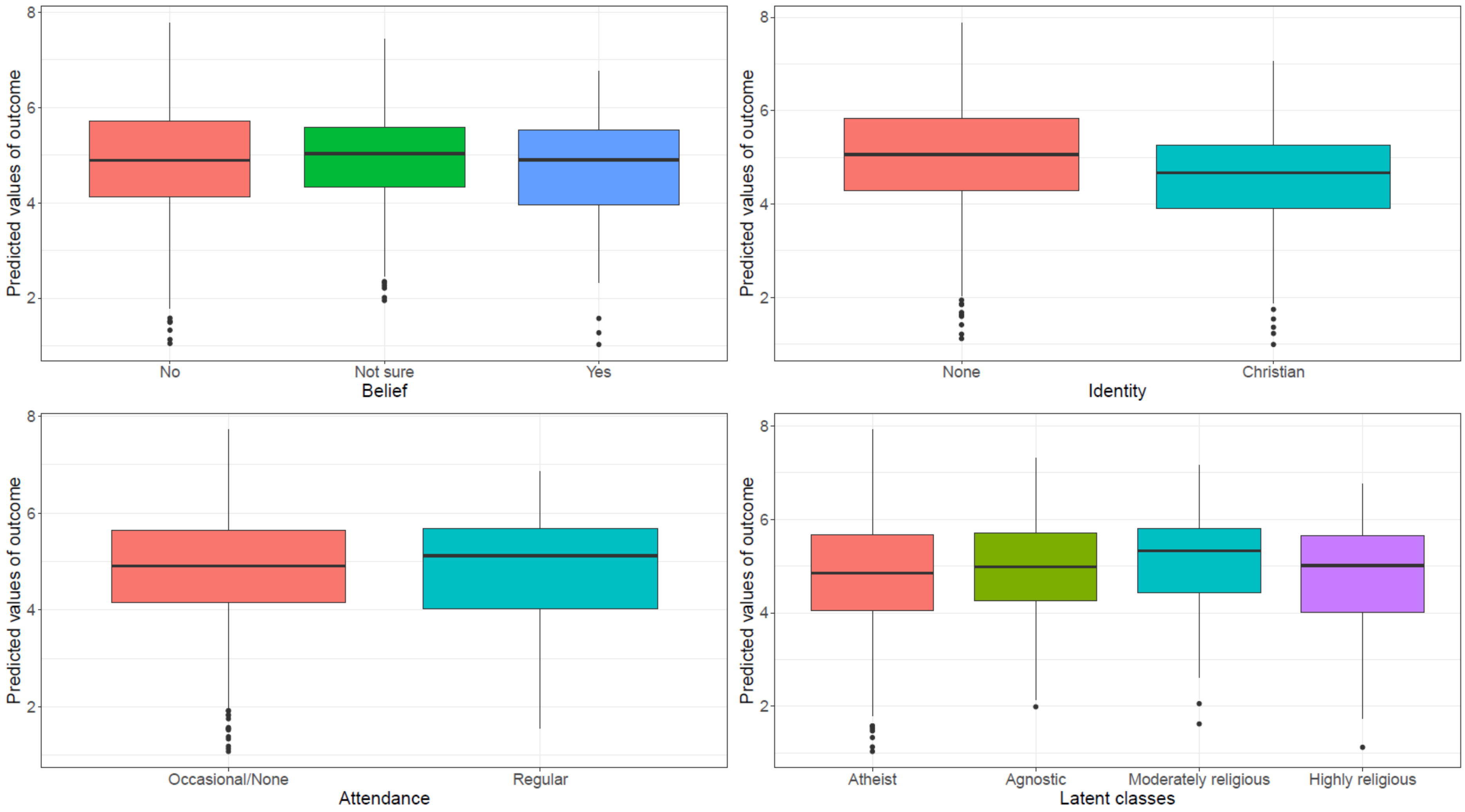

Figure S187: Predicted total number of actions (excluding ones which may be prohibitively costly) performed due to climate change for the religious identity (with the Christian denominations separated) as the exposure based on the offspring zero-inflated Poisson regression models. Results are for the adjusted models excluding political ideology (results including political ideology are practically identical).

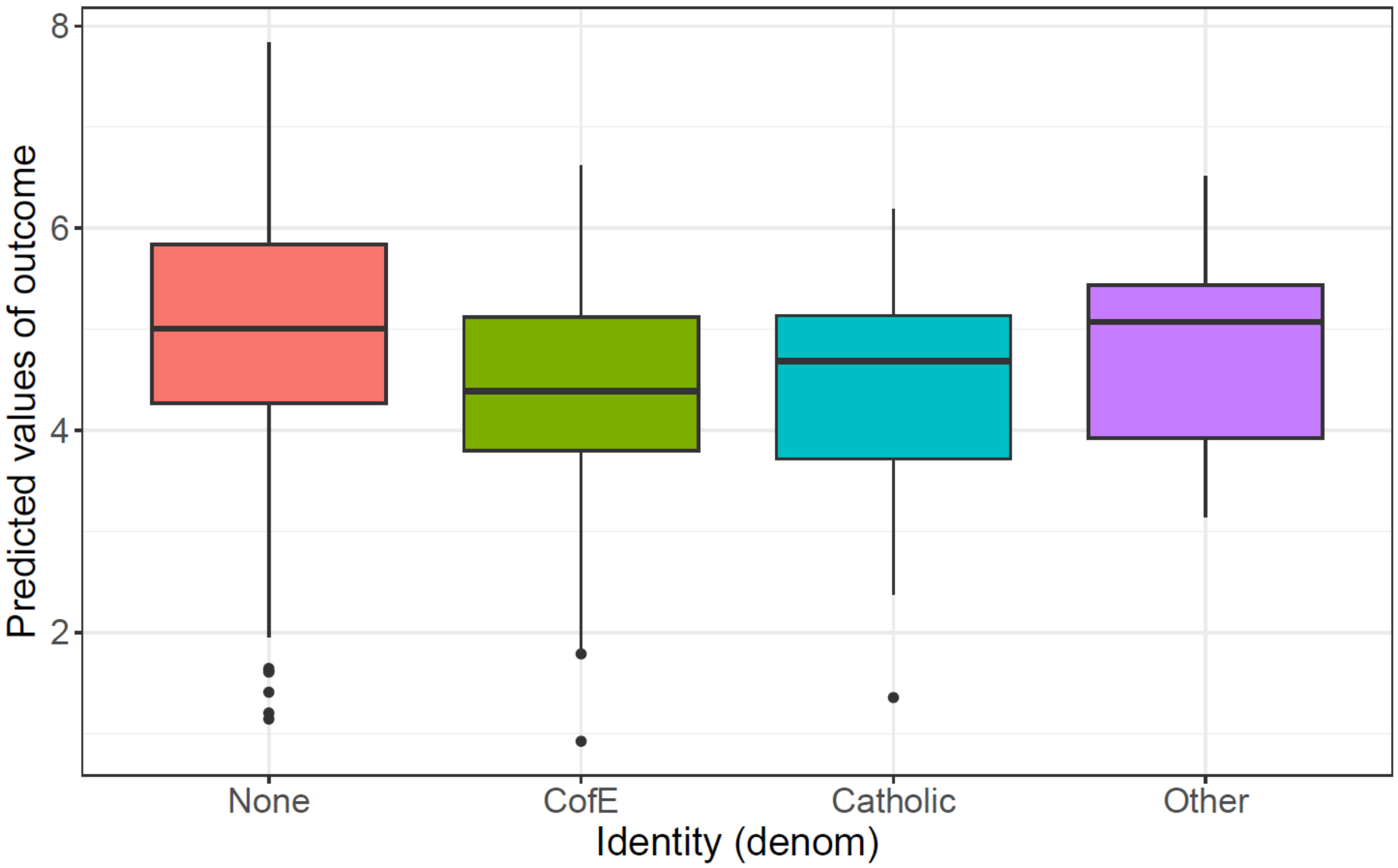

Figure S188: Results of the offspring multinomial regression models with 'changed the way travel locally' as the outcome for four religious exposures (belief [*n* = 1,094], identity [*n* = 1,093], attendance [*n* = 1,085] and latent classes [*n* = 1,042]; models are separated by dashed horizontal lines). See table S43 for full results.

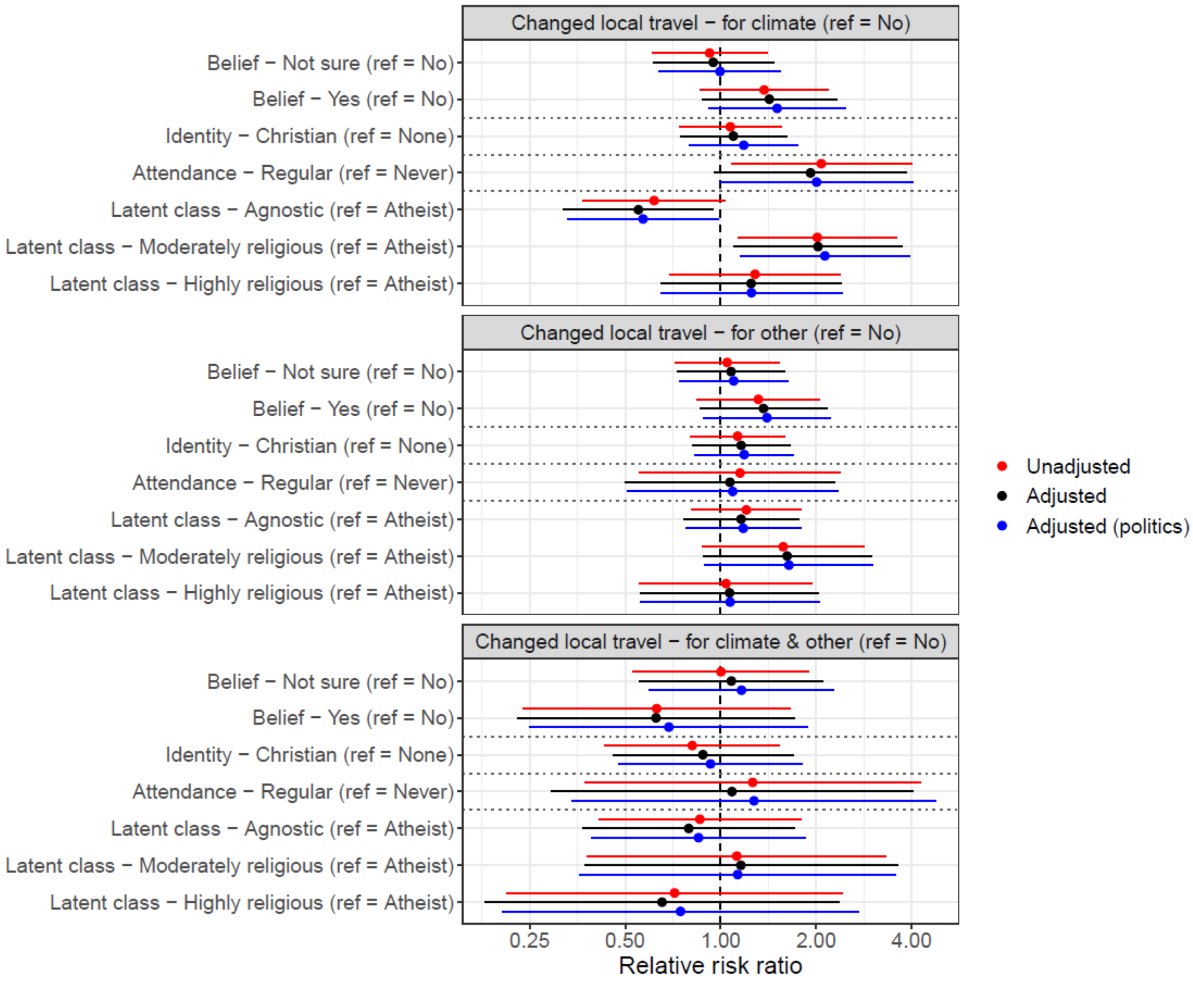

Figure S189: Predicted probabilities of the offspring multinomial regression models with ‘changed the way travel locally’ as the outcome for four religious exposures (belief, identity, attendance and latent classes). Results are for the adjusted models excluding political ideology (results including political ideology are practically identical).

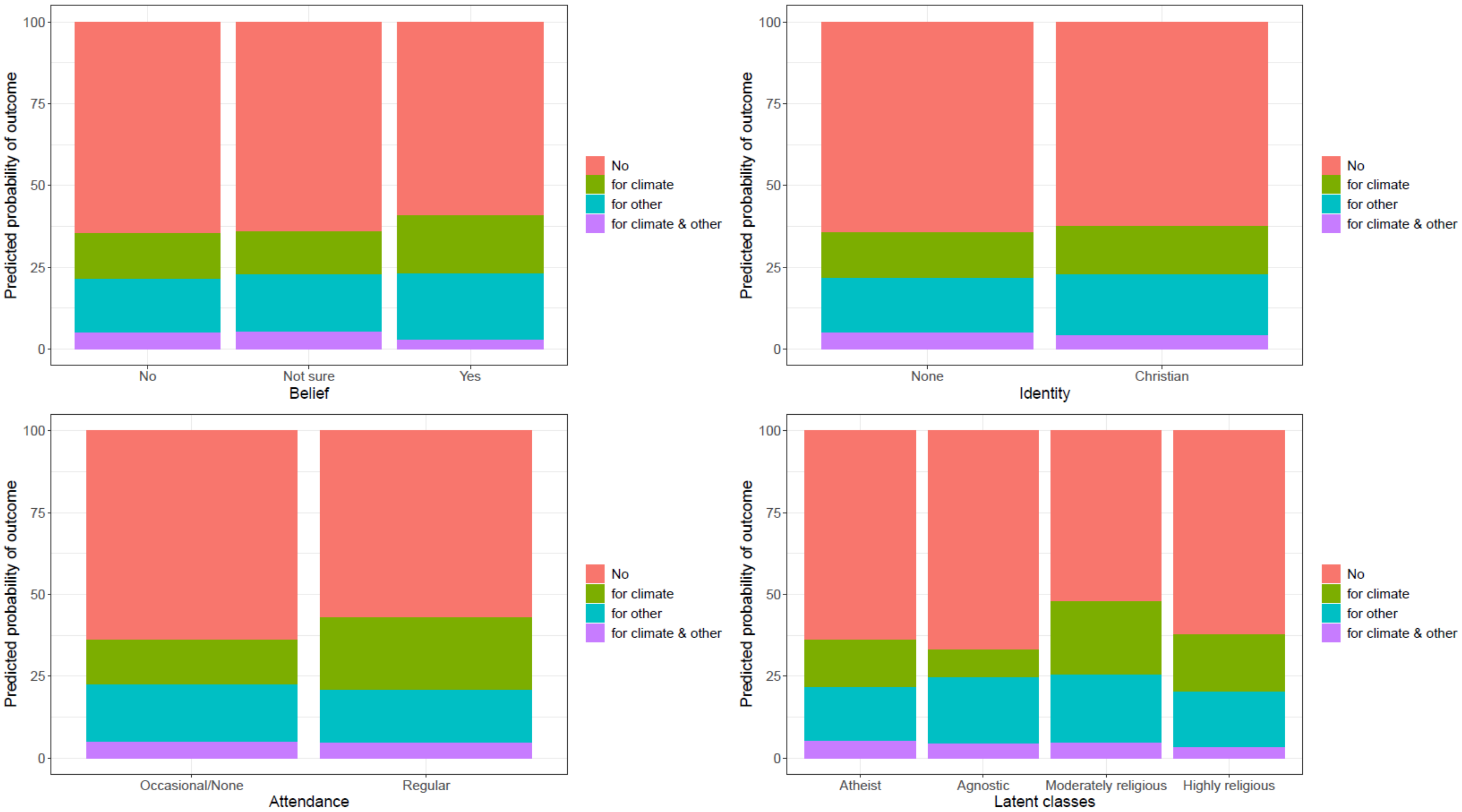

Figure S190: Predicted probabilities of the offspring multinomial regression models with 'changed the way travel locally' as the outcome and the religious identity (with the Christian denominations separated) as the exposure. Results are for the adjusted models excluding political ideology (results including political ideology are practically identical).

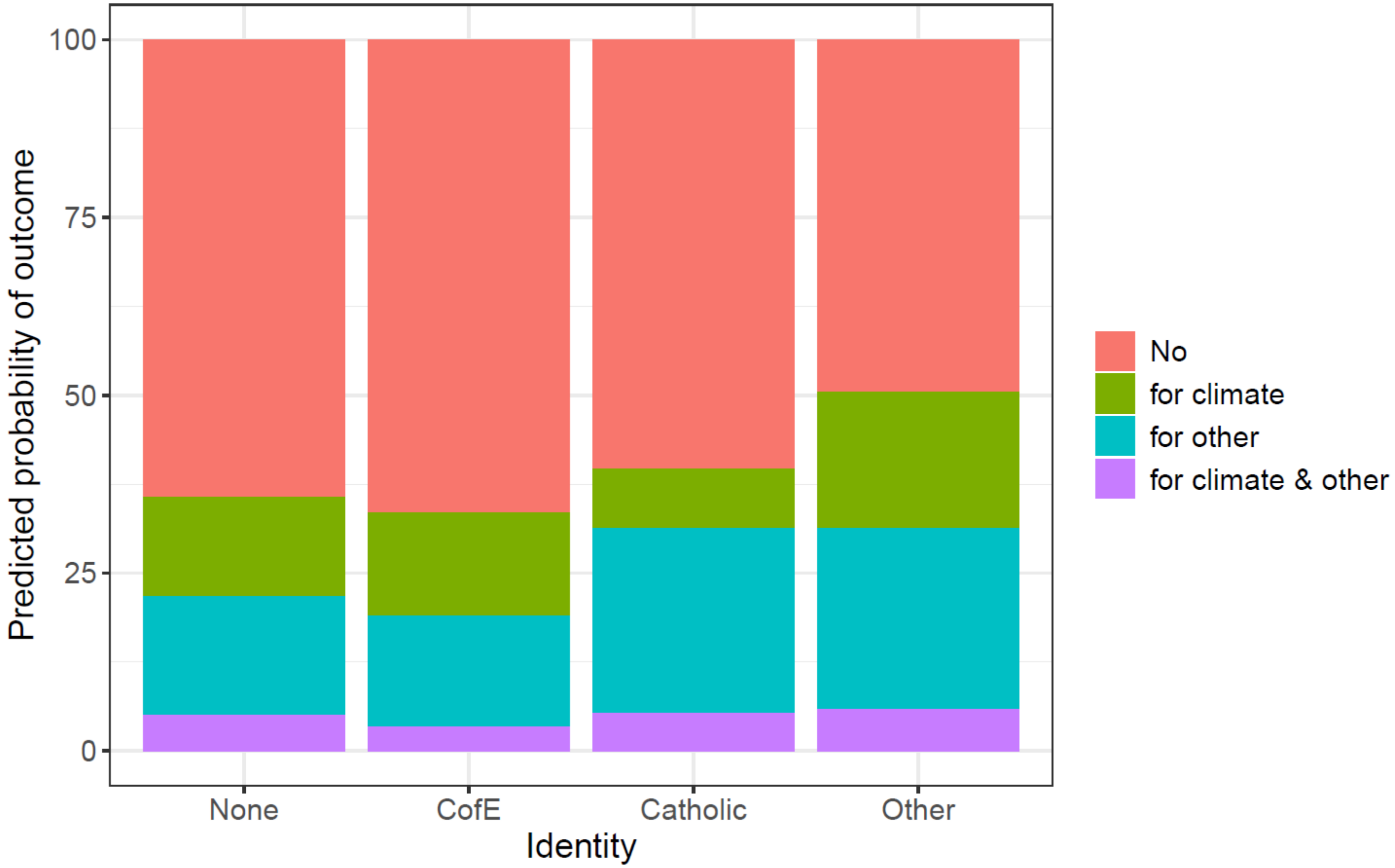

Figure S191: Results of the offspring multinomial regression models with 'reduced household waste' as the outcome for four religious exposures (belief [*n* = 1,109096 identity [*n* = 1,095], attendance [*n* = 1,087], and latent classes [*n* = 1,044]; models are separated by dashed horizontal lines). See table S43 for full results.

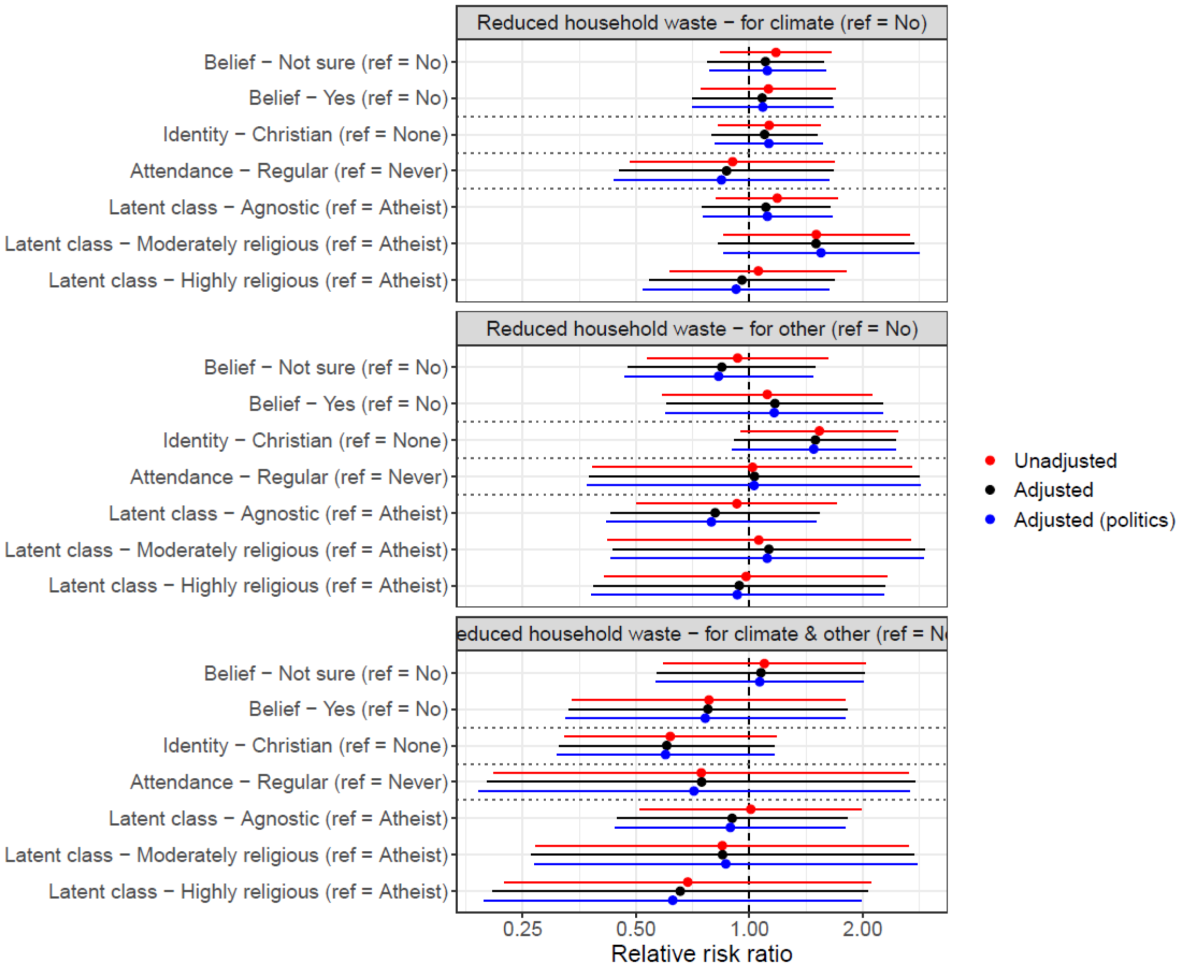

Figure S192: Predicted probabilities of the offspring multinomial regression models with 'reduced household waste' as the outcome for four religious exposures (belief, identity, attendance and latent classes). Results are for the adjusted models excluding political ideology (results including political ideology are practically identical).

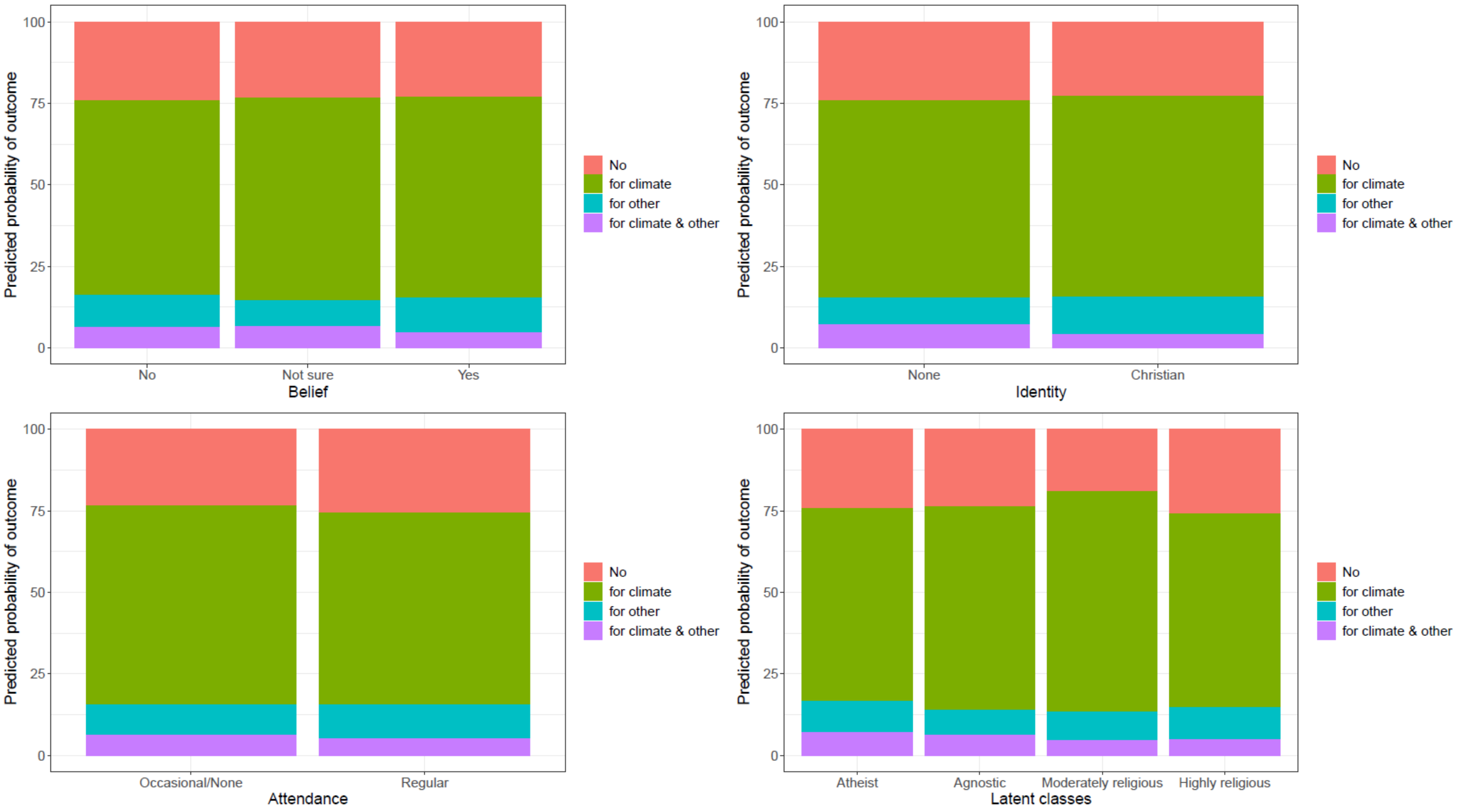

Figure S193: Predicted probabilities of the offspring multinomial regression models with 'reduced household waste' as the outcome and the religious identity (with the Christian denominations separated) as the exposure. Results are for the adjusted models excluding political ideology (results including political ideology are practically identical).

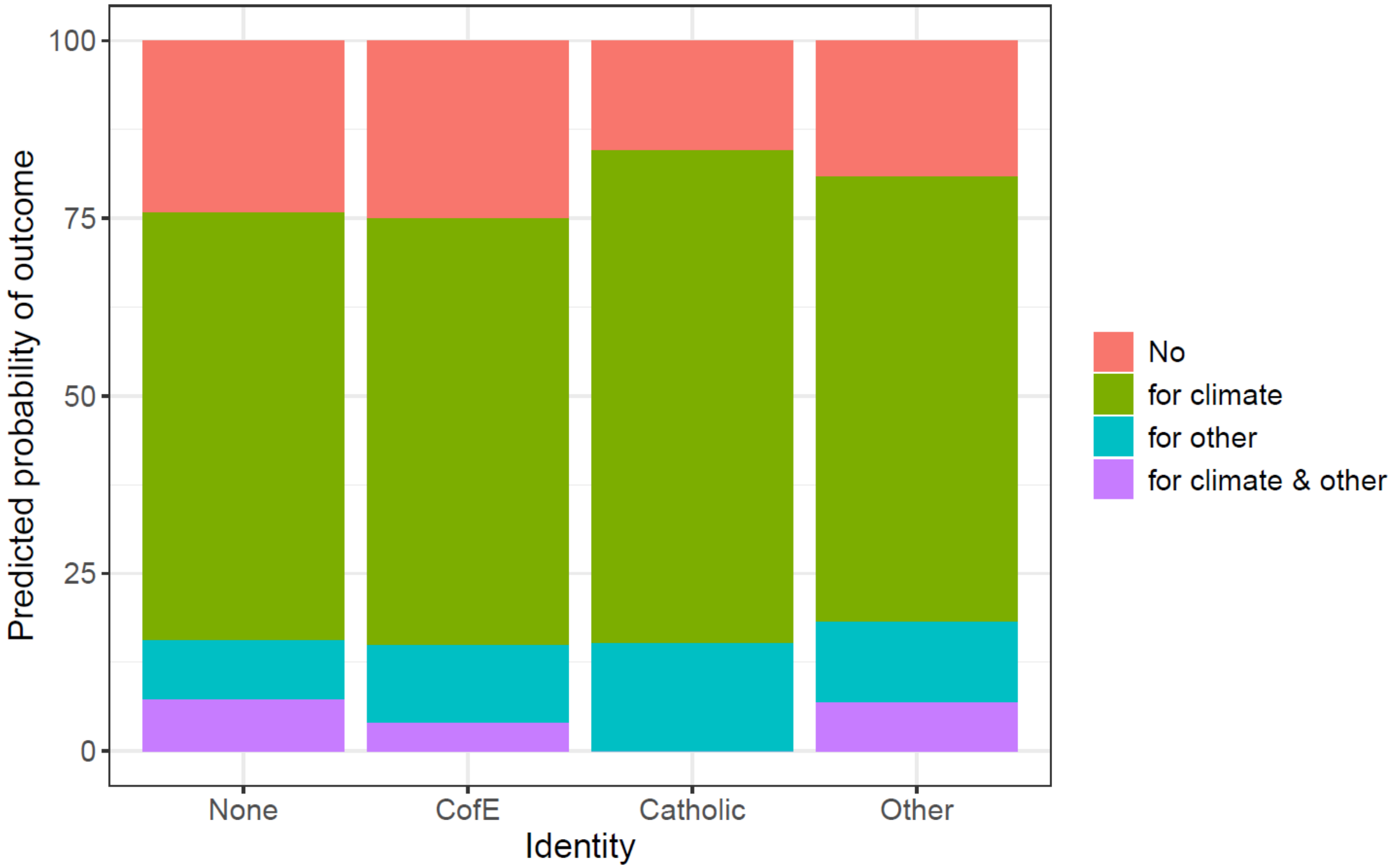

Figure S194: Results of the offspring multinomial regression models with 'reduced energy use at home' as the outcome for four religious exposures (belief [*n* = 1,095], identity [*n* = 1,094], attendance [*n* = 1,086], and latent classes [*n* = 1,044]; models are separated by dashed horizontal lines). See table S43 for full results.

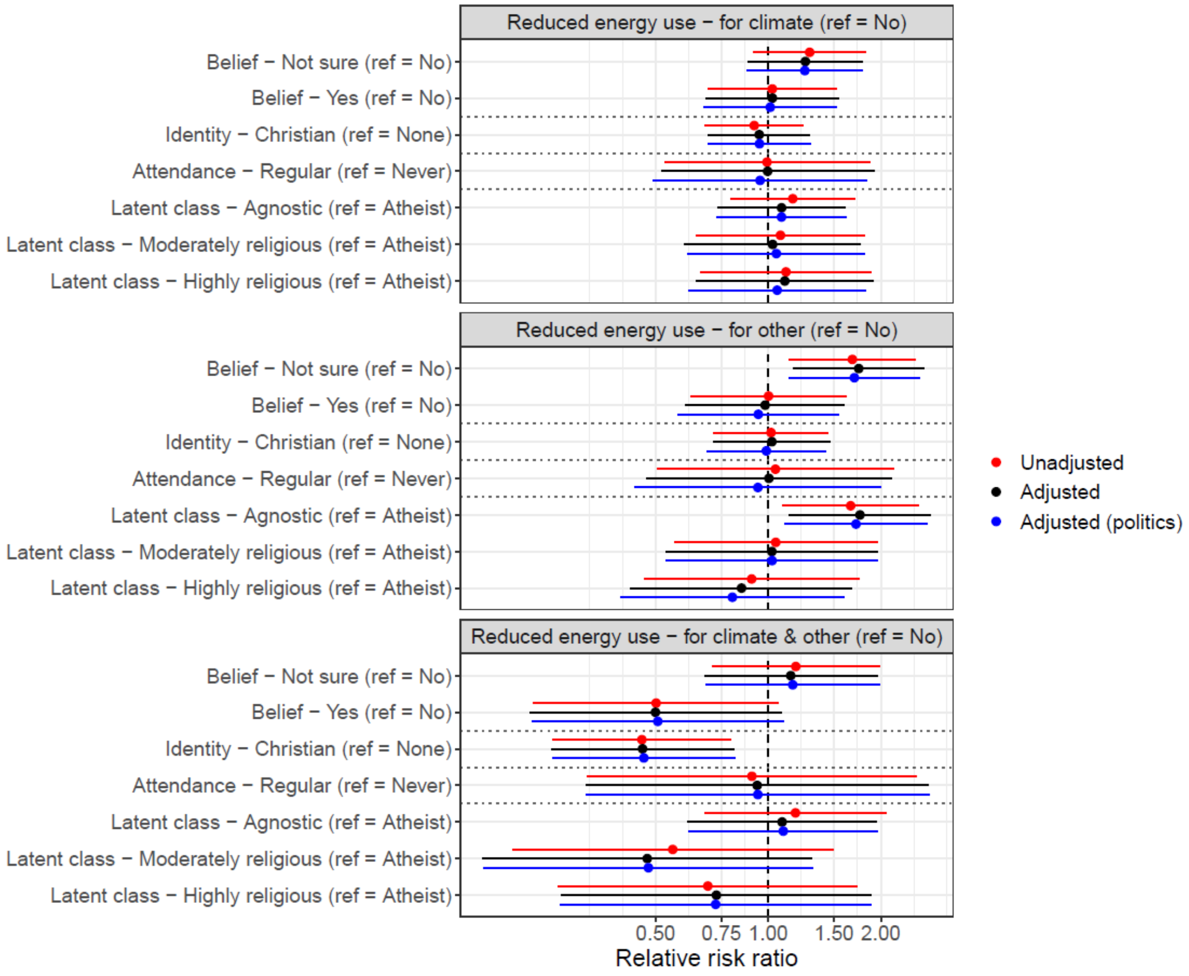

Figure S195: Predicted probabilities of the offspring multinomial regression models with 'reduced energy use at home' as the outcome for four religious exposures (belief, identity, attendance and latent classes). Results are for the adjusted models excluding political ideology (results including political ideology are practically identical).

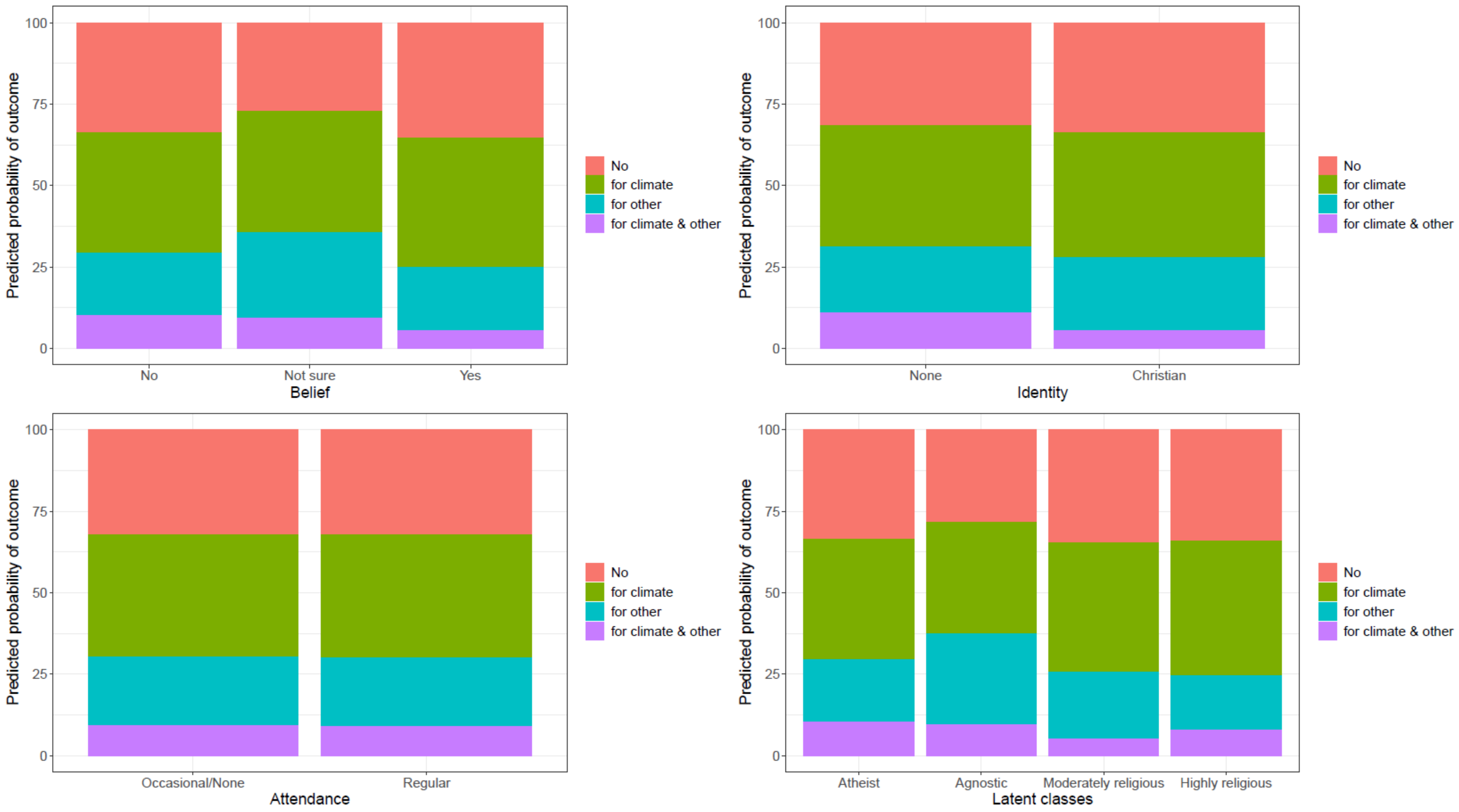

Figure S196: Predicted probabilities of the offspring multinomial regression models with 'reduced energy use at home' as the outcome and the religious identity (with the Christian denominations separated) as the exposure. Results are for the adjusted models excluding political ideology (results including political ideology are practically identical).

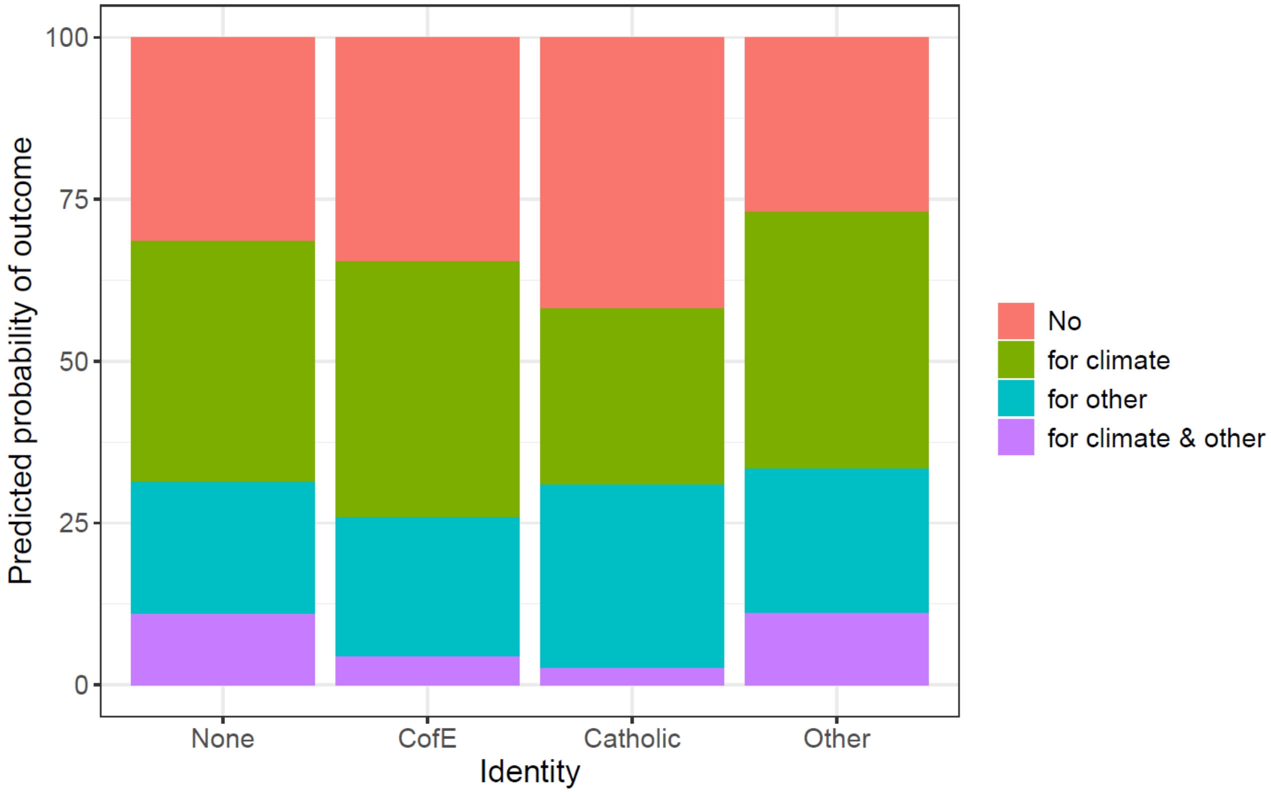

Figure S197: Results of the offspring multinomial regression models with ‘changed what buy’ as the outcome for four religious exposures (belief [*n* = 1,092], identity [*n* = 1,091], attendance [*n* = 1,083], and latent classes [*n* = 1,040]; models are separated by dashed horizontal lines). See table S43 for full results.

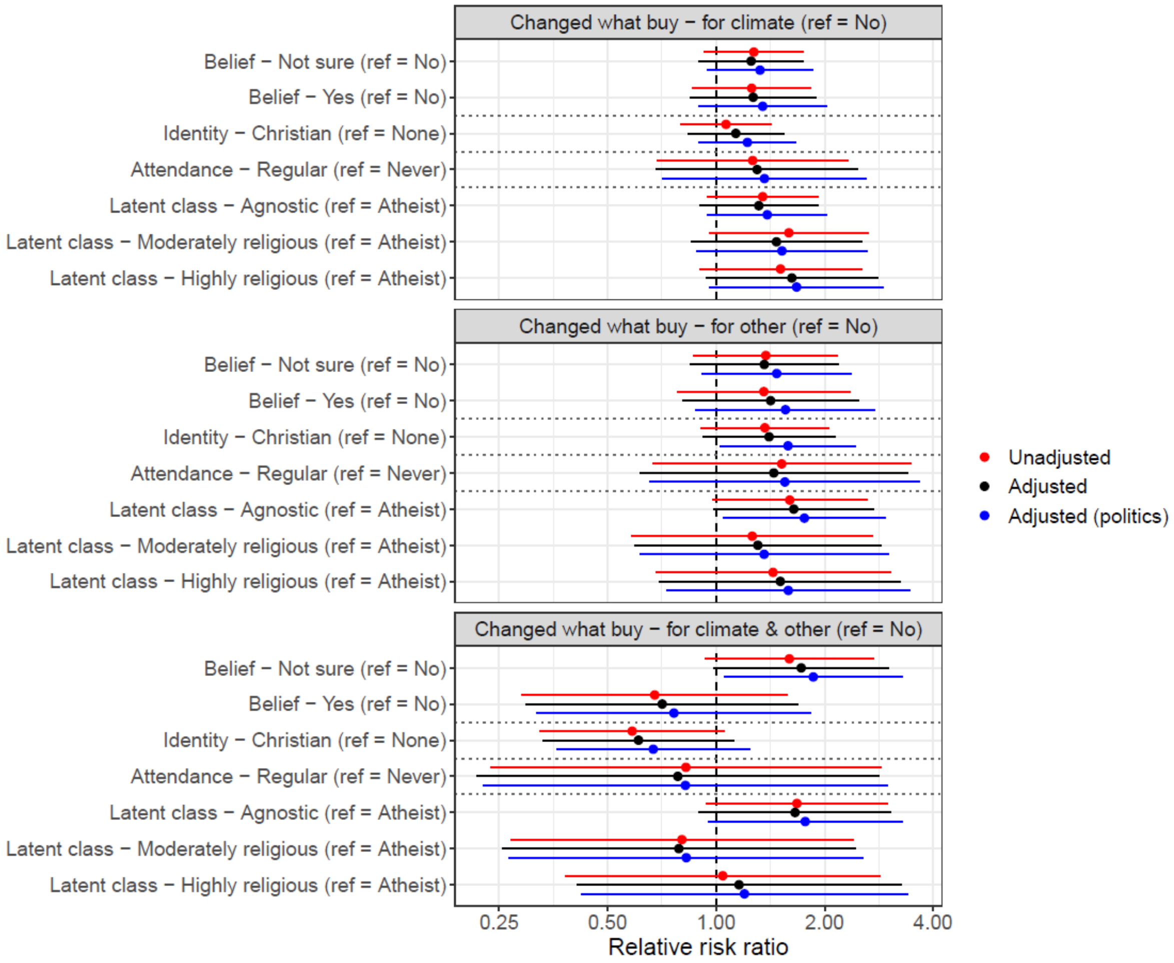

Figure S198: Predicted probabilities of the offspring multinomial regression models with 'changed what buy' as the outcome for four religious exposures (belief, identity, attendance and latent classes). Results are for the adjusted models excluding political ideology (results including political ideology are practically identical).

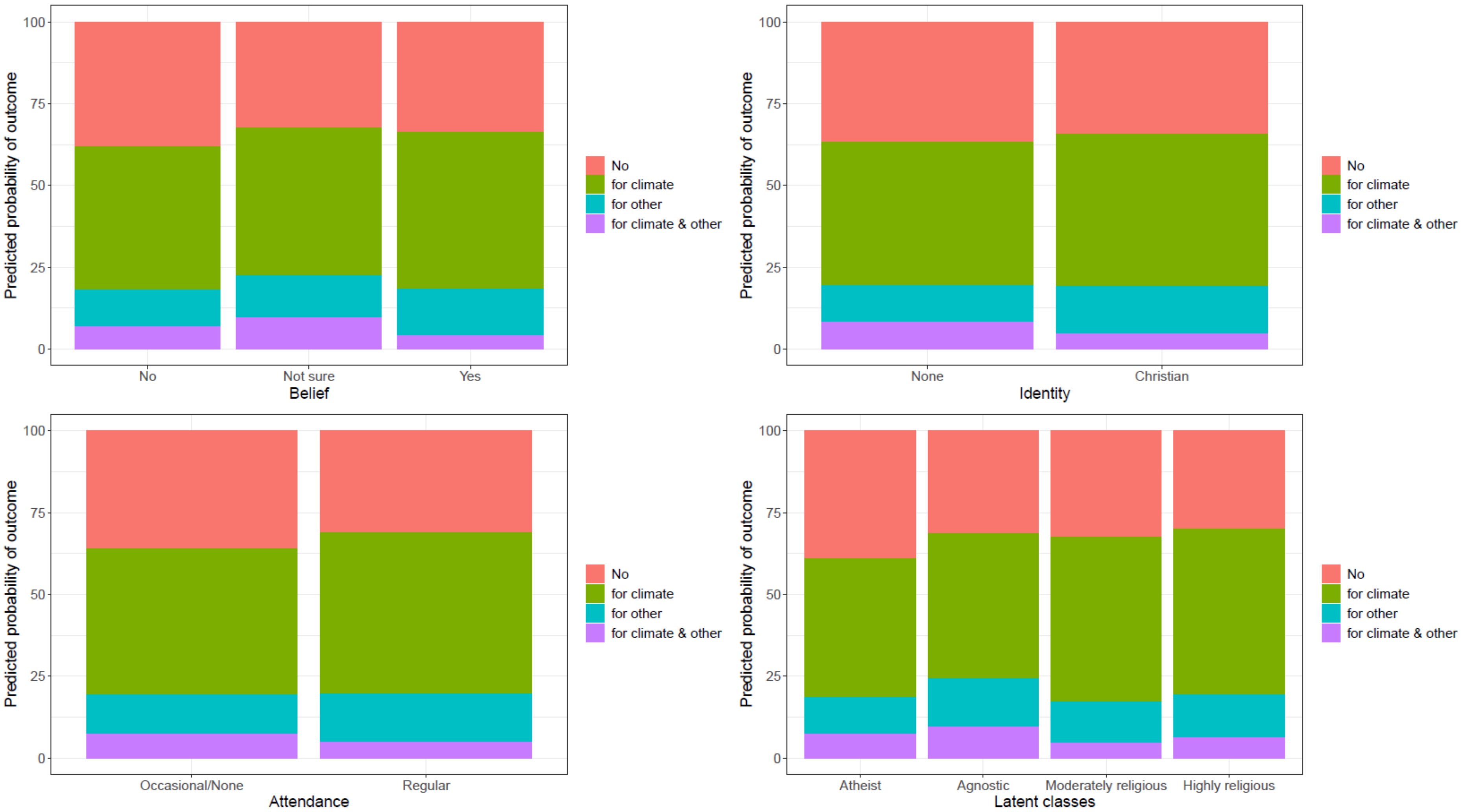

Figure S199: Predicted probabilities of the offspring multinomial regression models with ‘changed what buy’ as the outcome and the religious identity (with the Christian denominations separated) as the exposure. Results are for the adjusted models excluding political ideology (results including political ideology are practically identical).

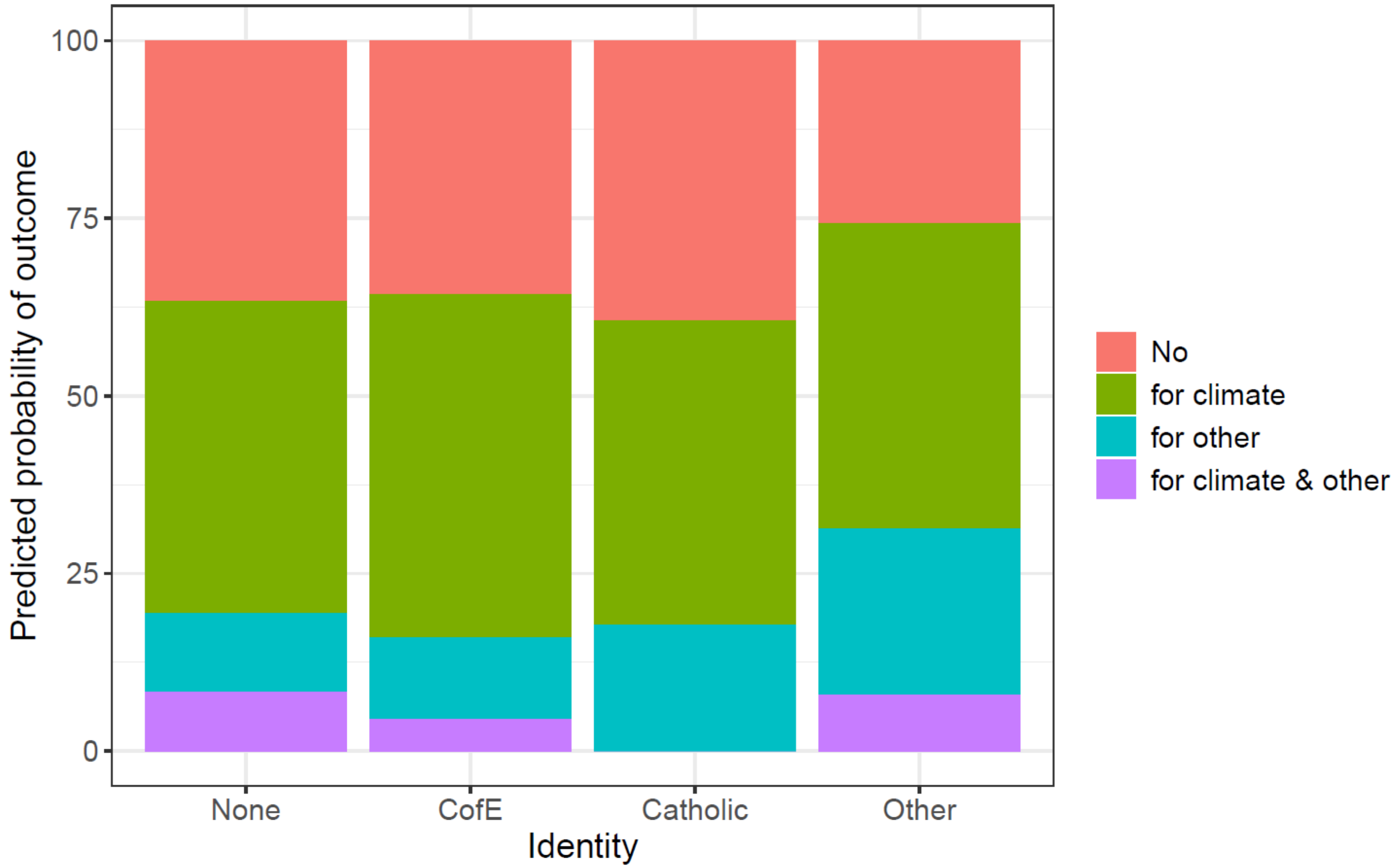

Figure S200: Results of the offspring multinomial regression models with ‘reduced air travel’ as the outcome for four religious exposures (belief [*n* = 1,091], identity [*n* = 1,090], attendance [*n* = 1,082], and latent classes [*n* = 1,040]; models are separated by dashed horizontal lines). See table S43 for full results.

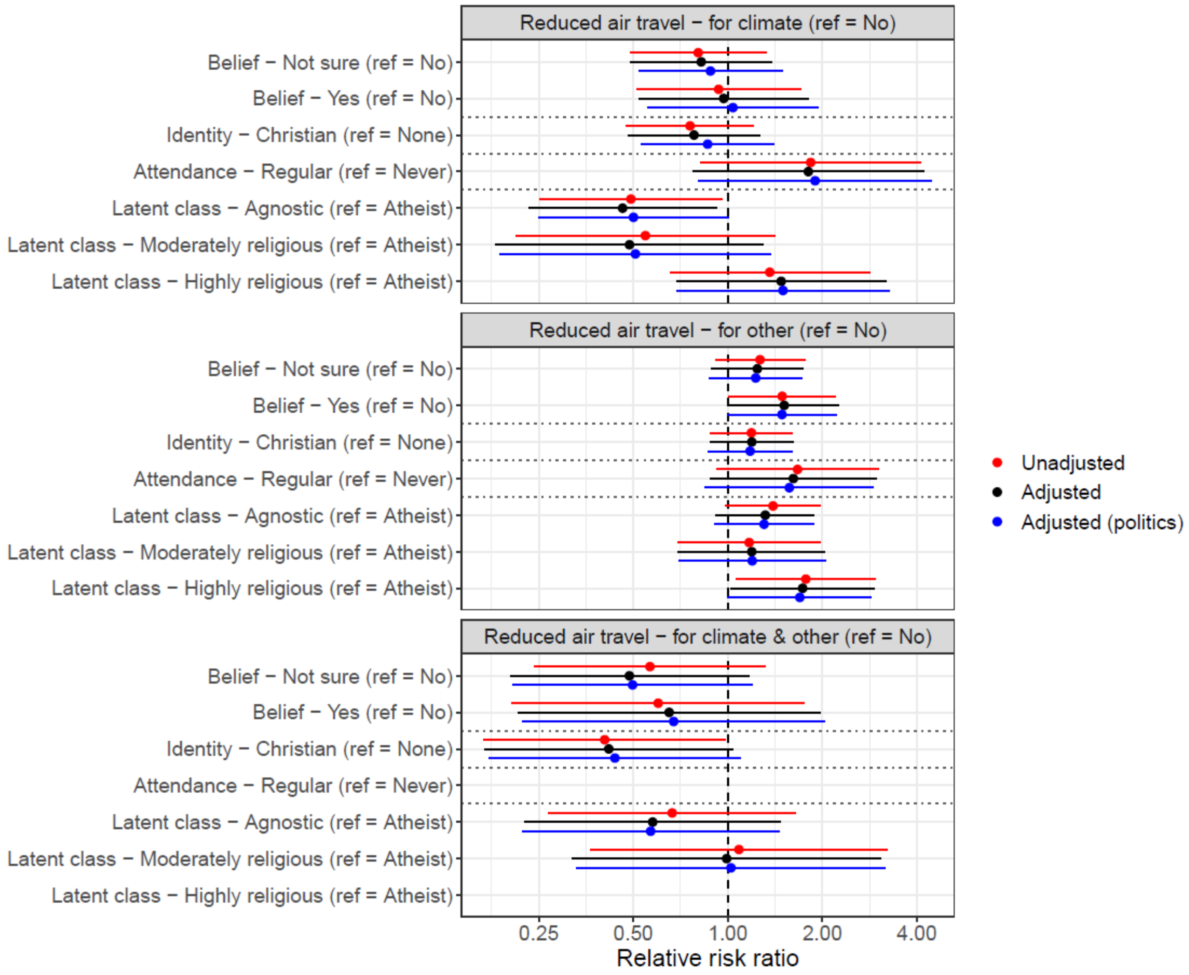

Figure S201: Predicted probabilities of the offspring multinomial regression models with 'reduced air travel' as the outcome for four religious exposures (belief, identity, attendance and latent classes). Results are for the adjusted models excluding political ideology (results including political ideology are practically identical).

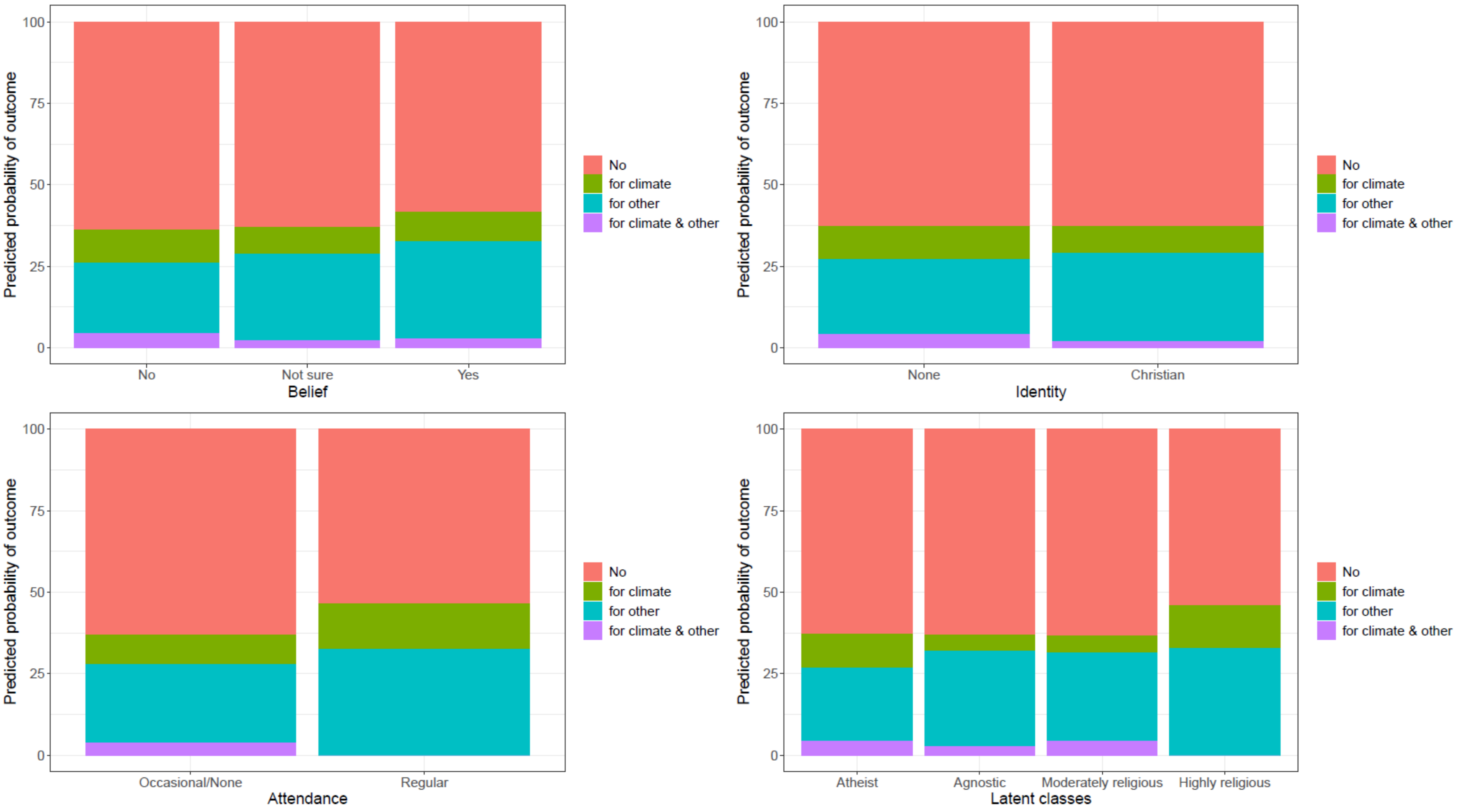

Figure S202: Predicted probabilities of the offspring multinomial regression models with 'reduced air travel' as the outcome and the religious identity (with the Christian denominations separated) as the exposure. Results are for the adjusted models excluding political ideology (results including political ideology are practically identical).

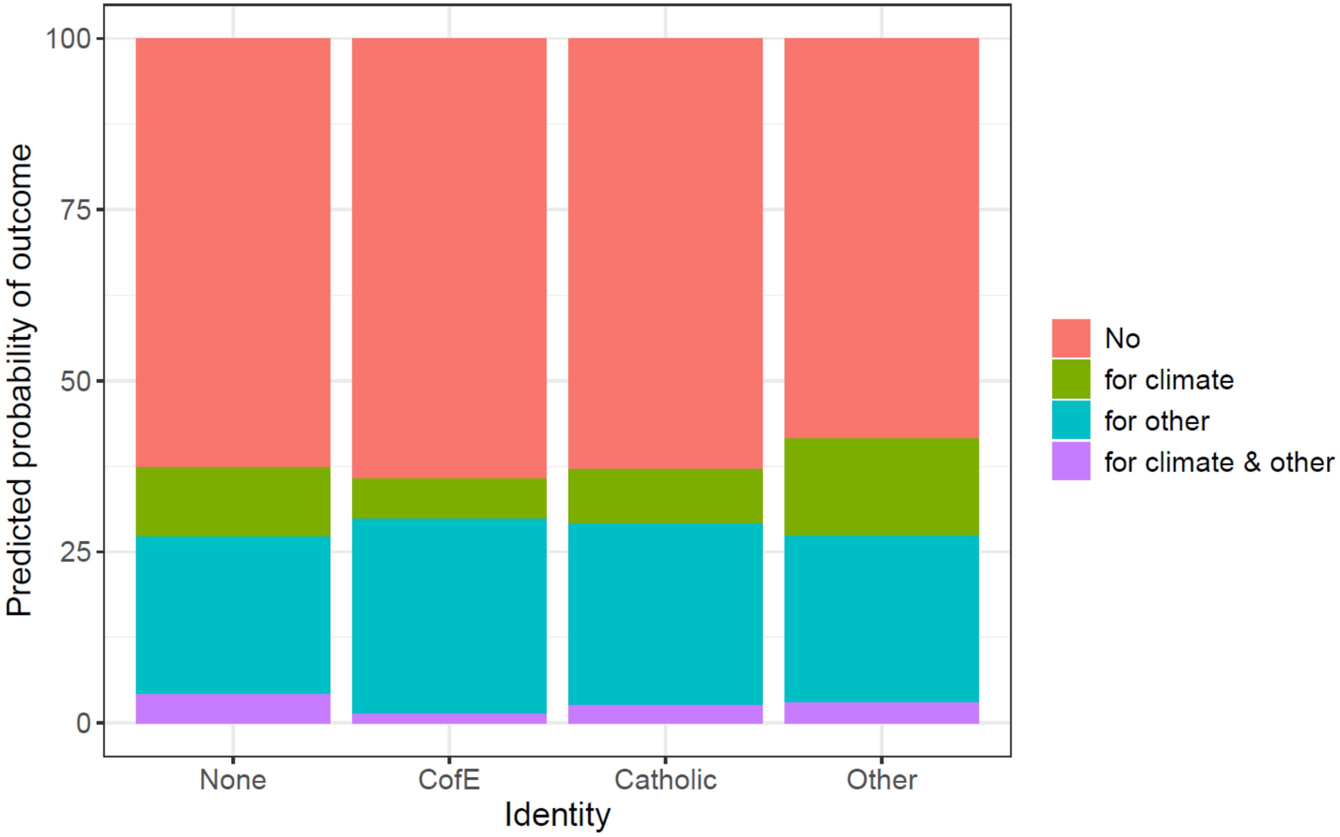

Figure S203: Results of the offspring multinomial regression models with 'bought or hired an electric or hybrid vehicle' as the outcome for four religious exposures (belief [*n* = 1,099], identity [*n* = 1,098], attendance [*n* = 1,090], and latent classes [*n* = 1,047]; models are separated by dashed horizontal lines). See table S43 for full results.

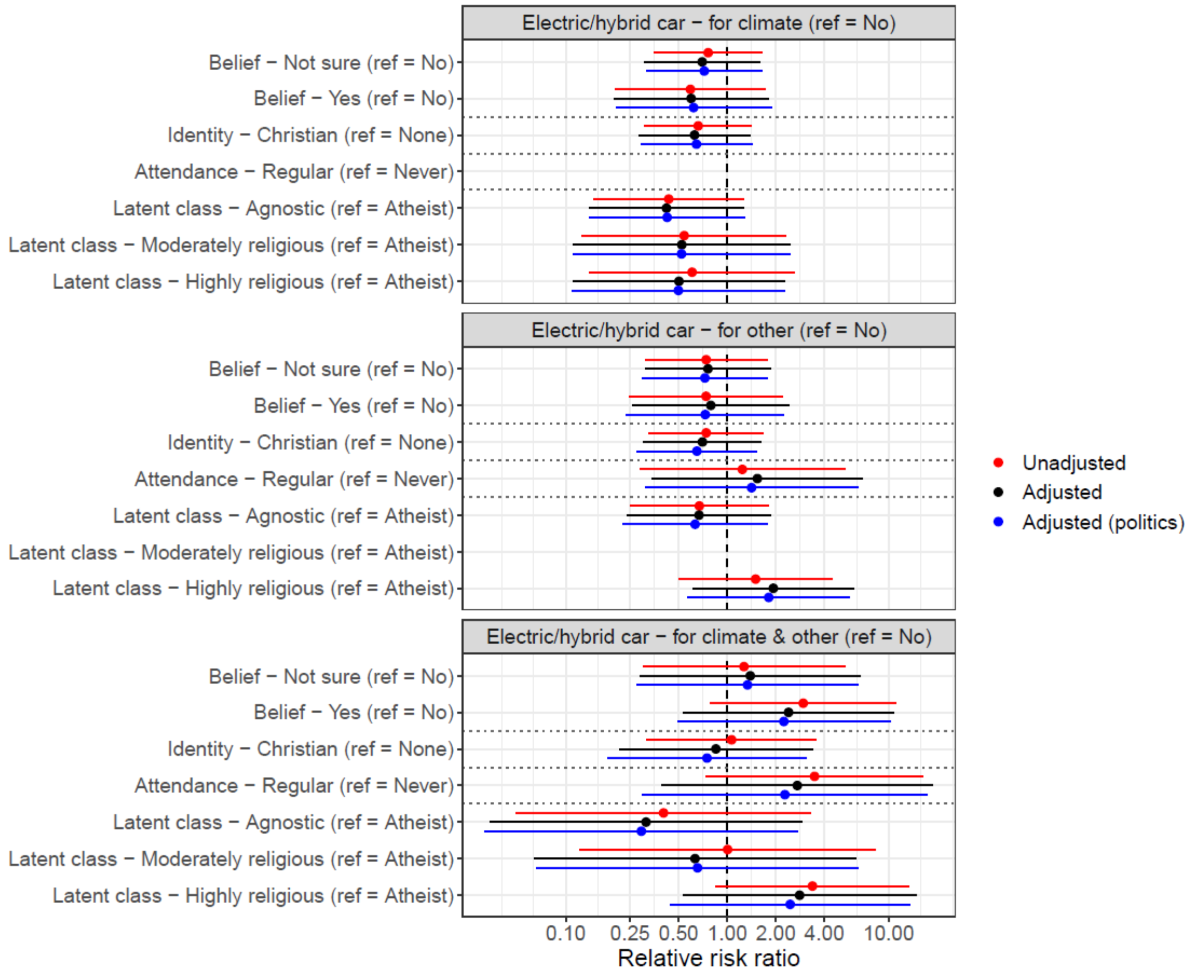

Figure S204: Predicted probabilities of the offspring multinomial regression models with ‘bought or hired an electric or hybrid vehicle’ as the outcome for four religious exposures (belief, identity, attendance and latent classes). Results are for the adjusted models excluding political ideology (results including political ideology are practically identical).

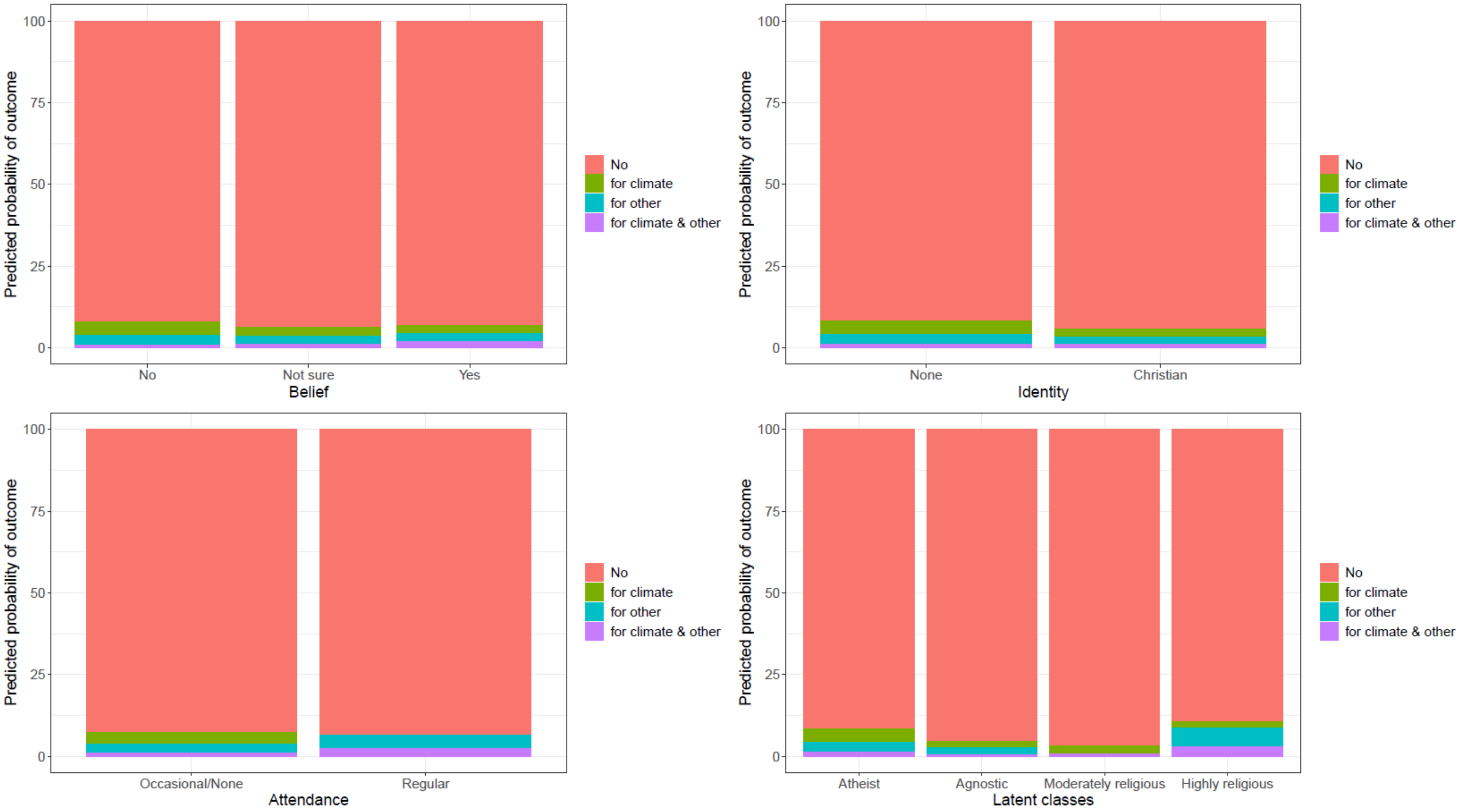

Figure S205: Predicted probabilities of the offspring multinomial regression models with 'bought or hired an electric or hybrid vehicle' as the outcome and the religious identity (with the Christian denominations separated) as the exposure. Results are for the adjusted models excluding political ideology (results including political ideology are practically identical).

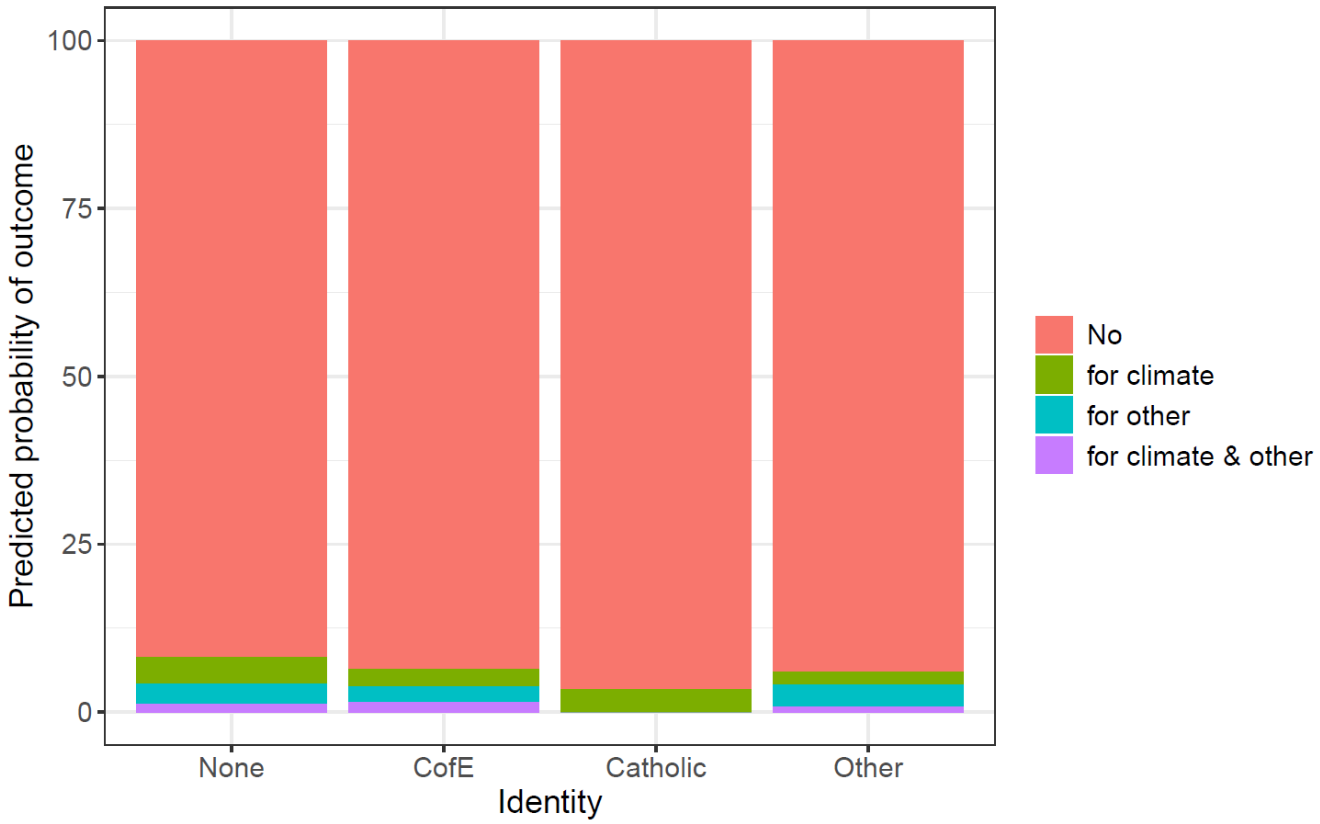

Figure S206: Results of the offspring multinomial regression models with 'bought foods produced locally' as the outcome for four religious exposures (belief [*n* = 1,096], identity [*n* = 1,095], attendance [*n* = 1,087], and latent classes [*n* = 1,044]; models are separated by dashed horizontal lines). See table S43 for full results.

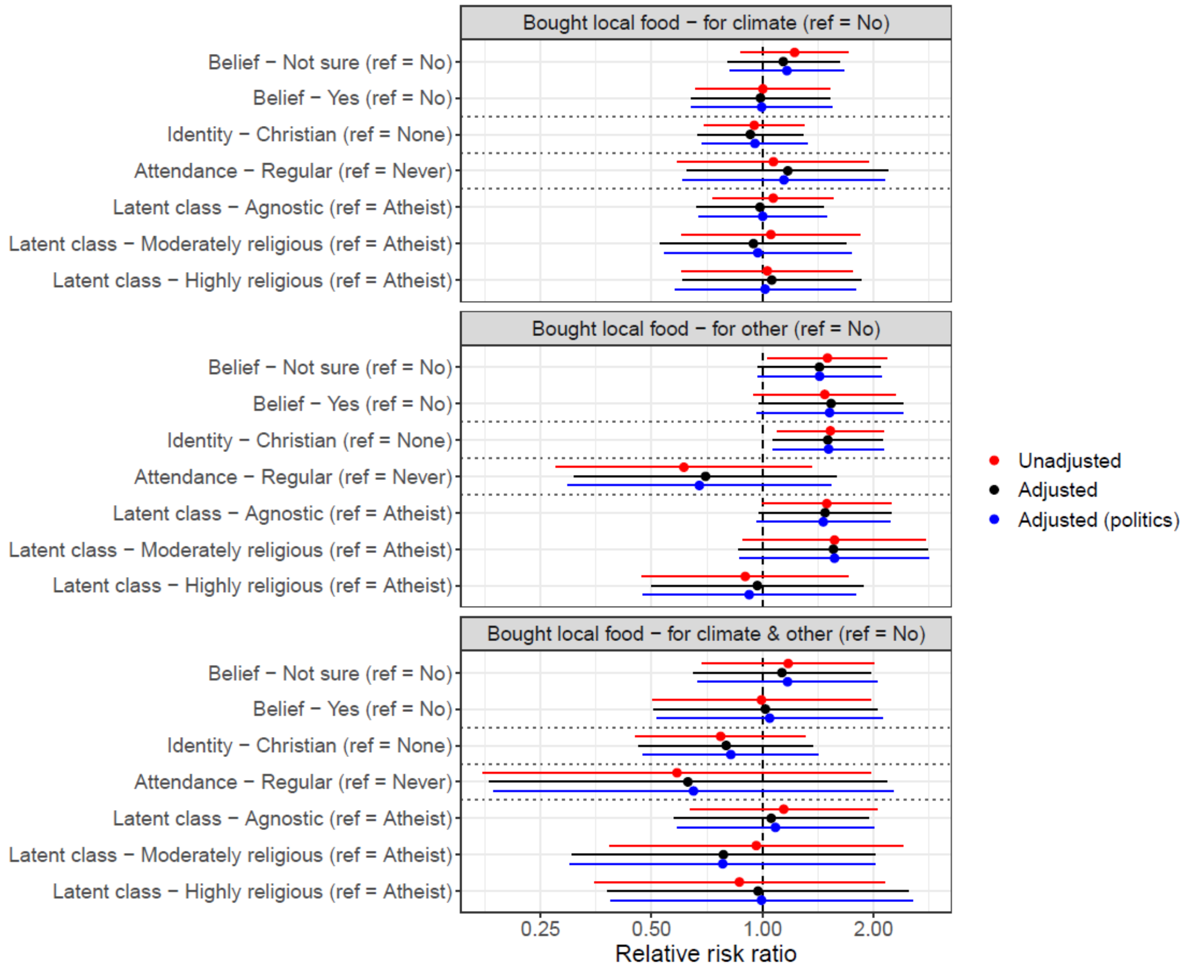

Figure S207: Predicted probabilities of the offspring multinomial regression models with 'bought foods produced locally' as the outcome for four religious exposures (belief, identity, attendance and latent classes). Results are for the adjusted models excluding political ideology (results including political ideology are practically identical).

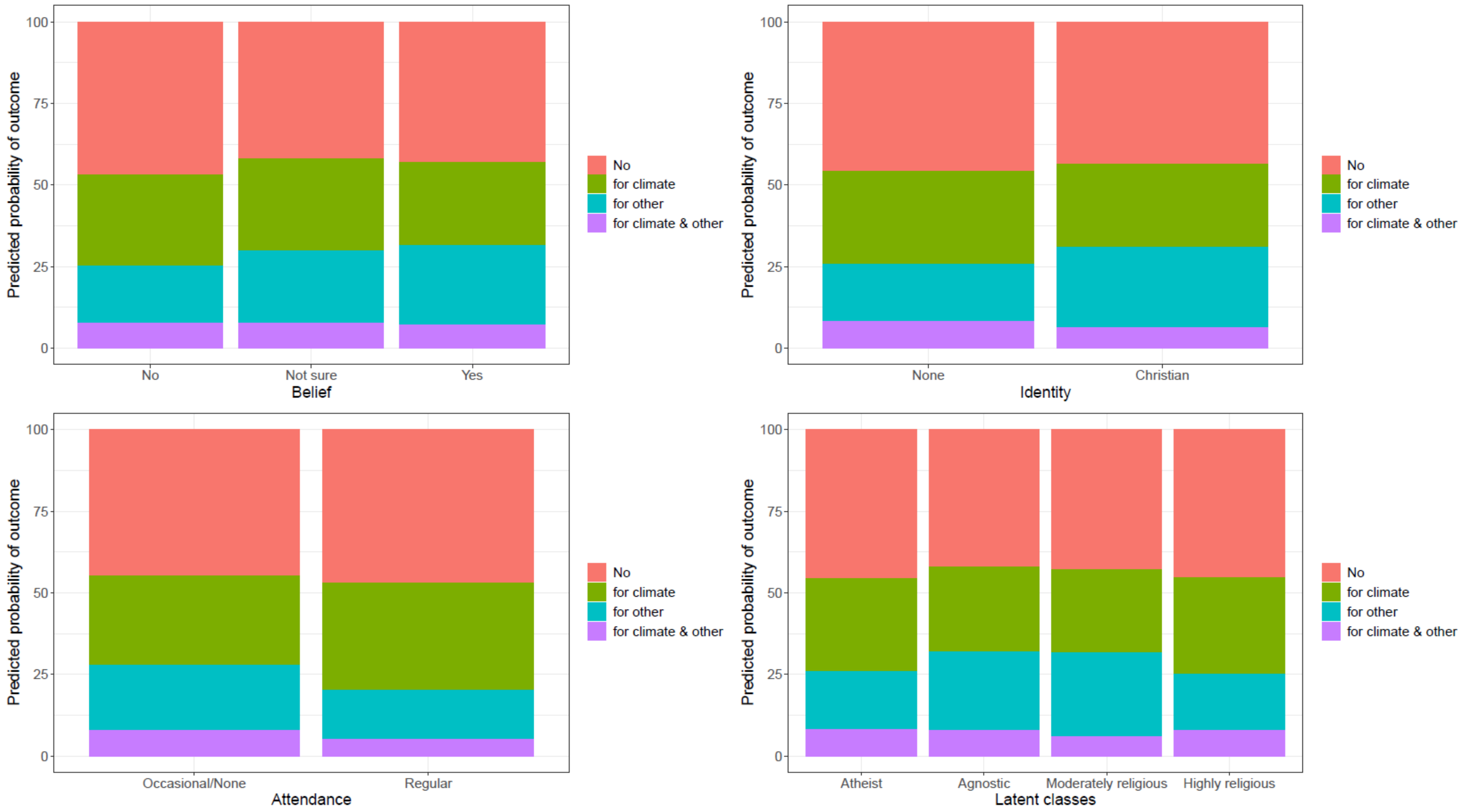

Figure S208: Predicted probabilities of the offspring multinomial regression models with 'bought foods produced locally' as the outcome and the religious identity (with the Christian denominations separated) as the exposure. Results are for the adjusted models excluding political ideology (results including political ideology are practically identical).

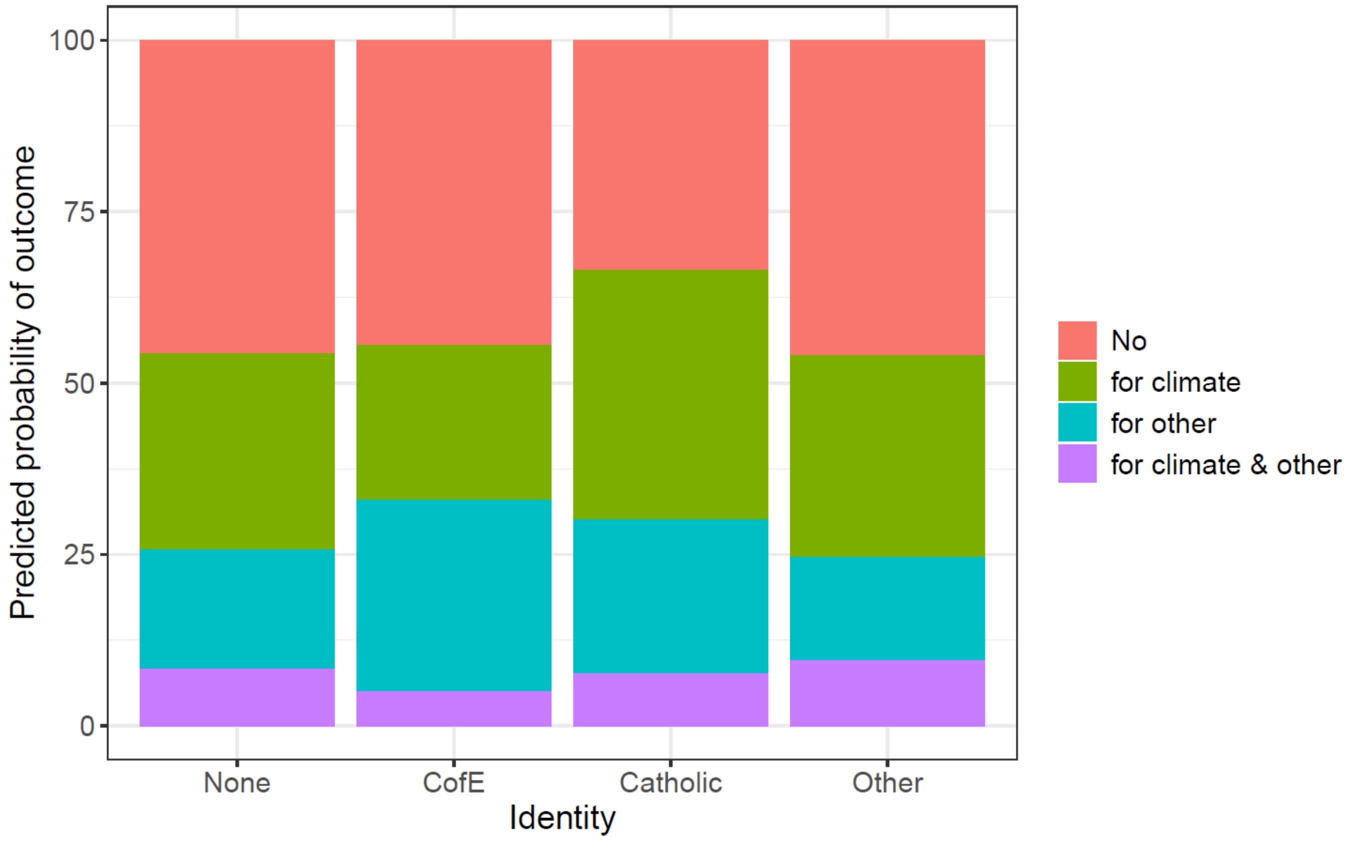

Figure S209: Results of the offspring multinomial regression models with ‘recycled or upcycled more’ as the outcome for four religious exposures (belief [*n* = 1,095], identity [*n* = 1,094], attendance [*n* = 1,086], and latent classes [*n* = 1,043]; models are separated by dashed horizontal lines). See table S43 for full results.

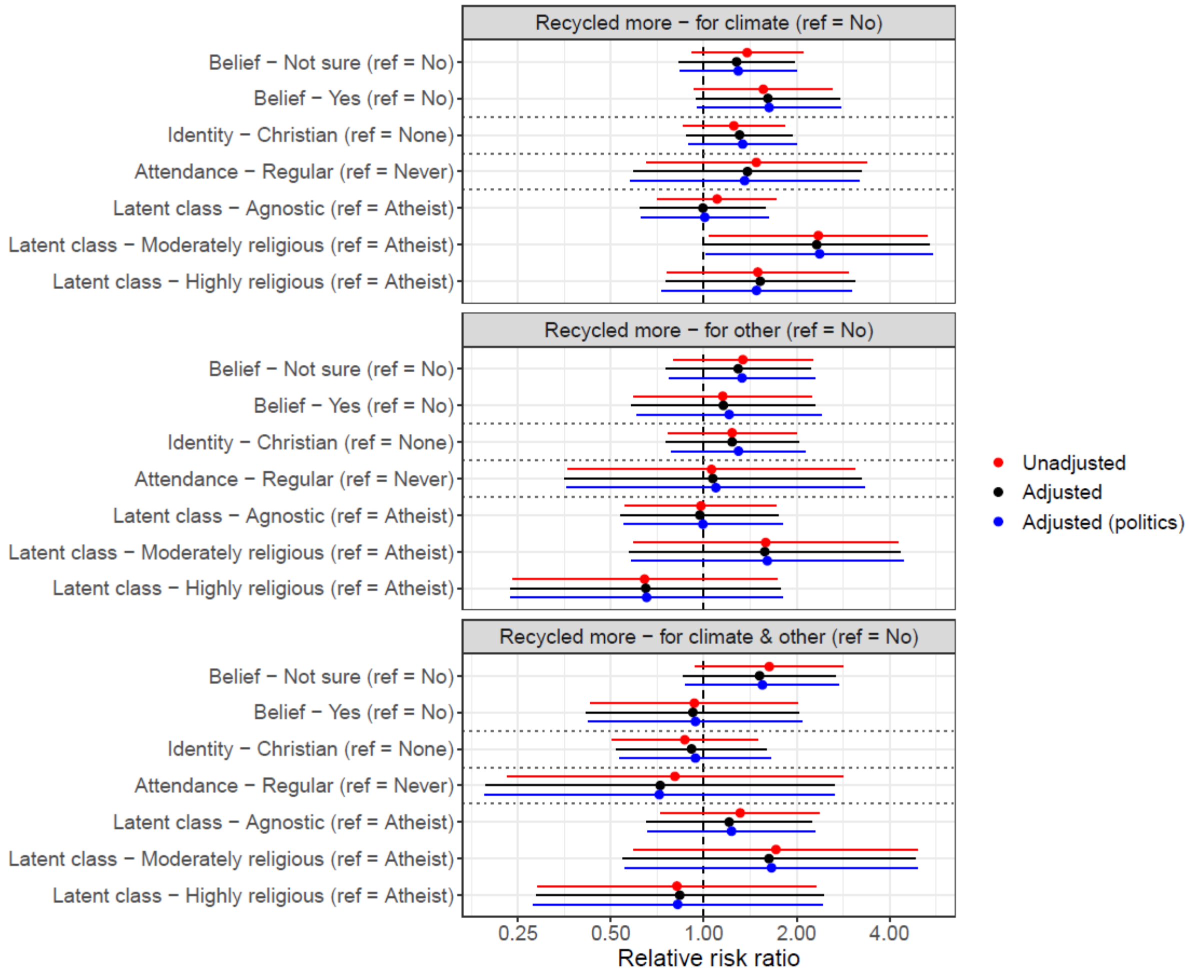

Figure S210: Predicted probabilities of the offspring multinomial regression models with 'recycled or upcycled more' as the outcome for four religious exposures (belief, identity, attendance and latent classes). Results are for the adjusted models excluding political ideology (results including political ideology are practically identical).

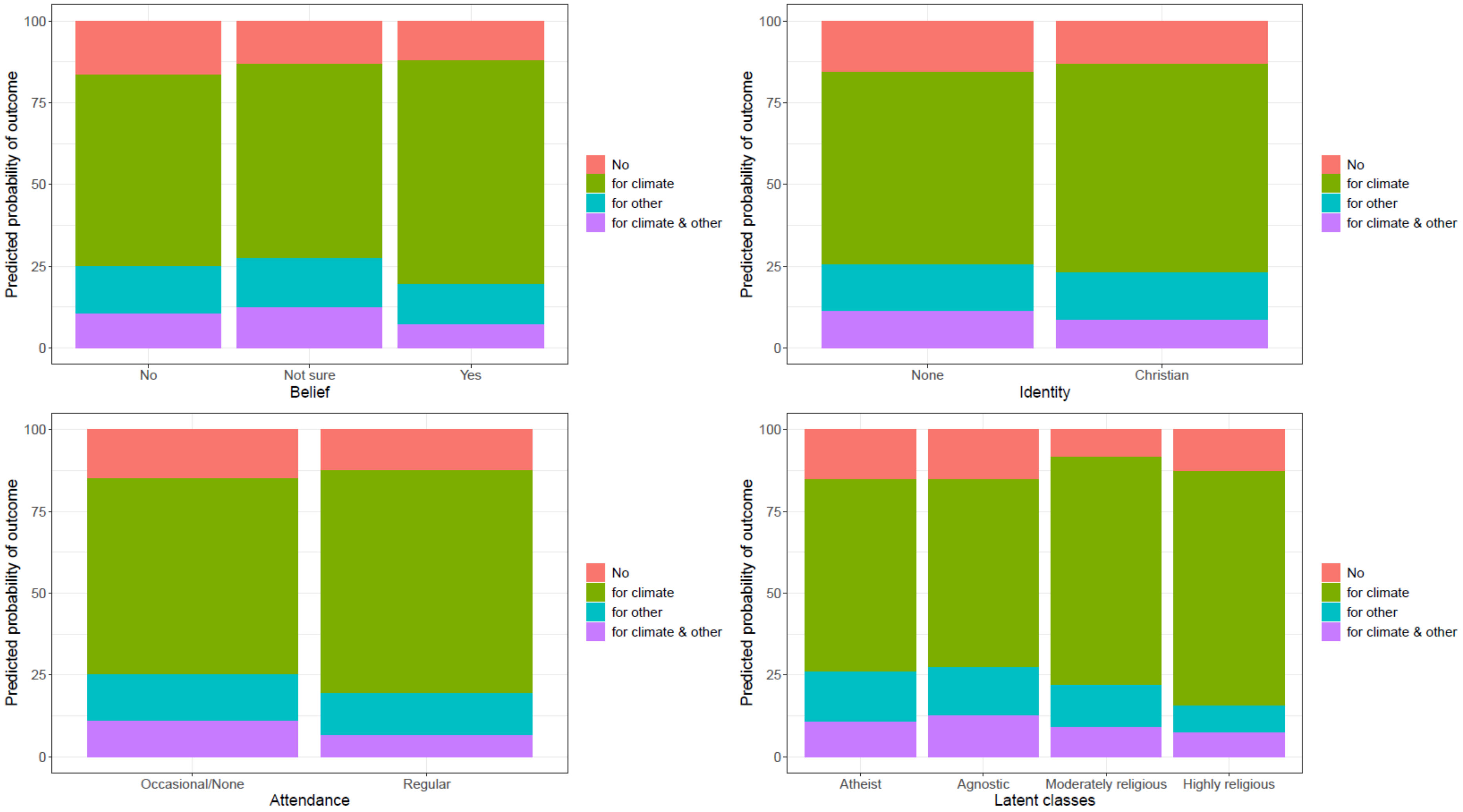

Figure S211: Predicted probabilities of the offspring multinomial regression models with 'recycled or upcycled more' as the outcome and the religious identity (with the Christian denominations separated) as the exposure. Results are for the adjusted models excluding political ideology (results including political ideology are practically identical).

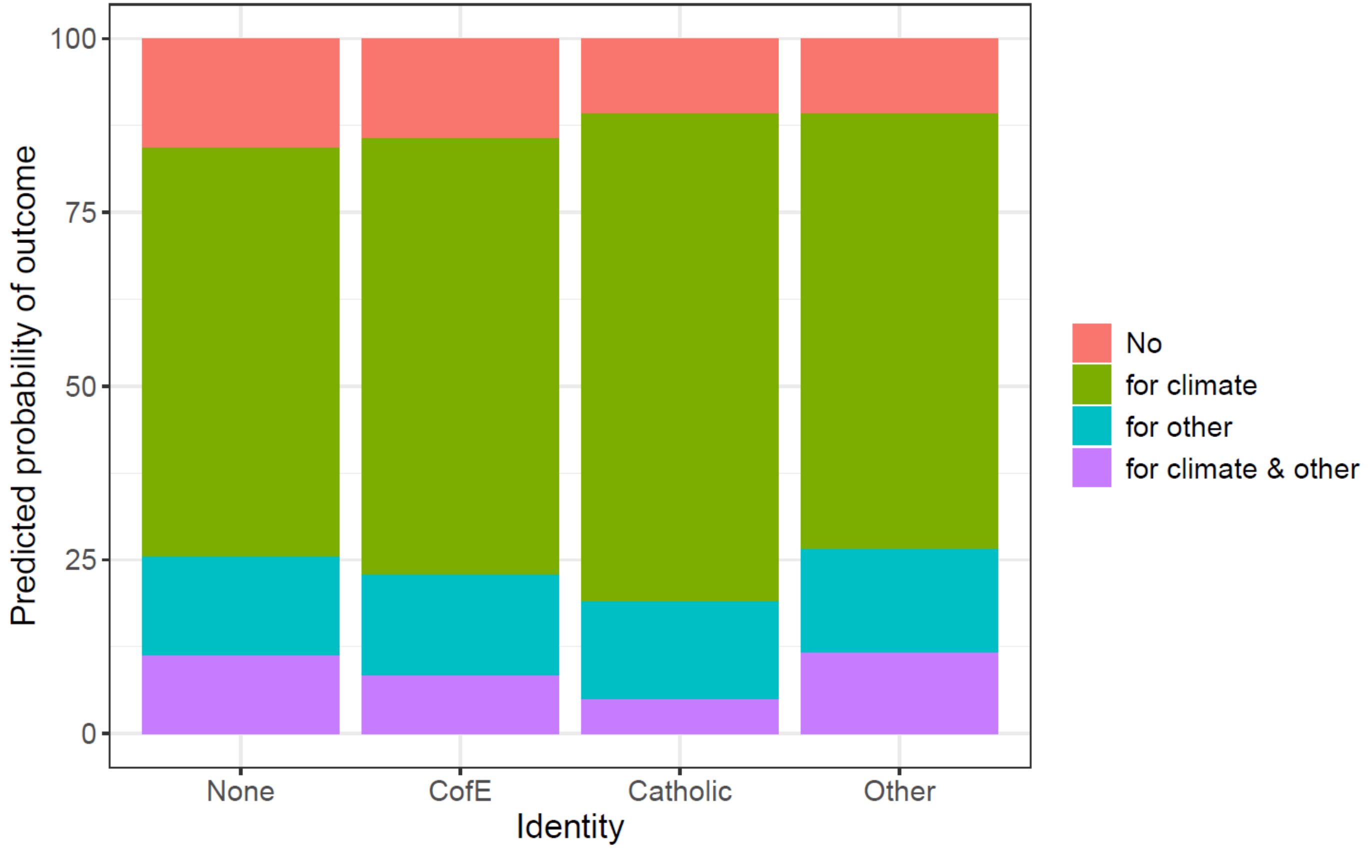

Figure S212: Results of the offspring multinomial regression models with ‘reduced the amount of plastic used’ as the outcome for four religious exposures (belief [*n* = 1,097], identity [*n* = 1,096], attendance [*n* = 1,088], and latent classes [*n* = 1,045]; models are separated by dashed horizontal lines). See table S43 for full results.

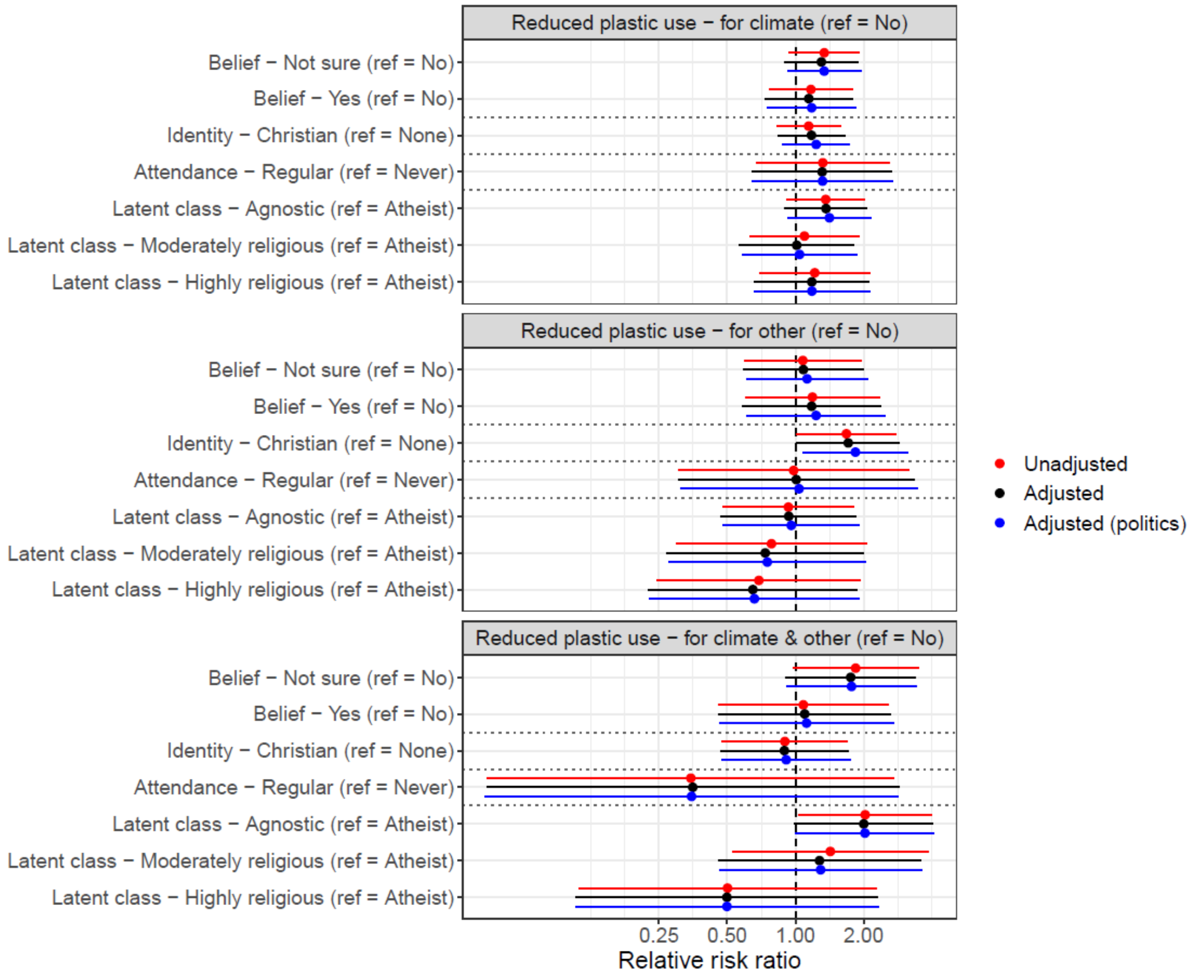

Figure S213: Predicted probabilities of the offspring multinomial regression models with 'reduced the amount of plastic used' as the outcome for four religious exposures (belief, identity, attendance and latent classes). Results are for the adjusted models excluding political ideology (results including political ideology are practically identical).

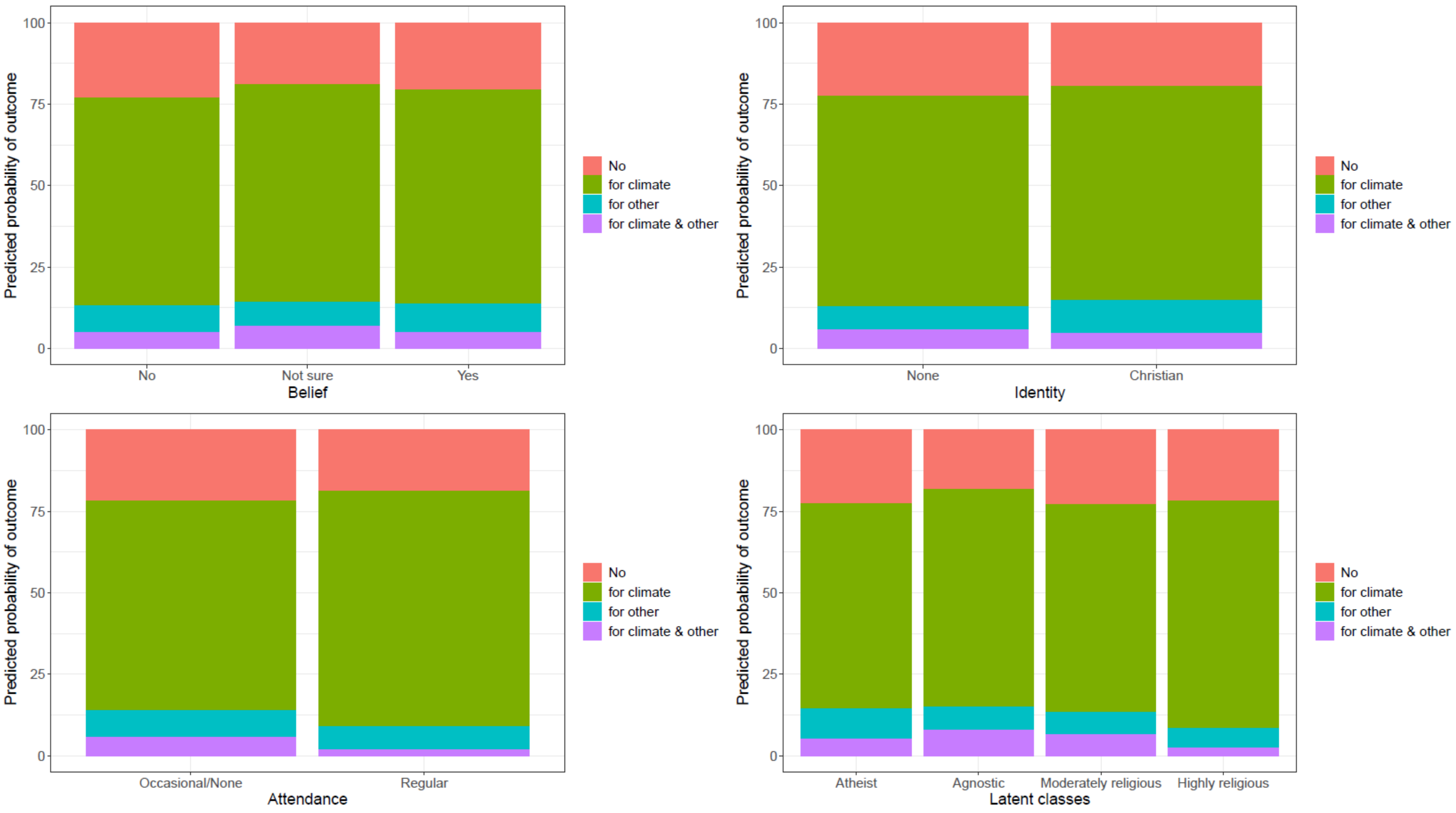

Figure S214: Predicted probabilities of the offspring multinomial regression models with 'reduced the amount of plastic used' as the outcome and the religious identity (with the Christian denominations separated) as the exposure. Results are for the adjusted models excluding political ideology (results including political ideology are practically identical).

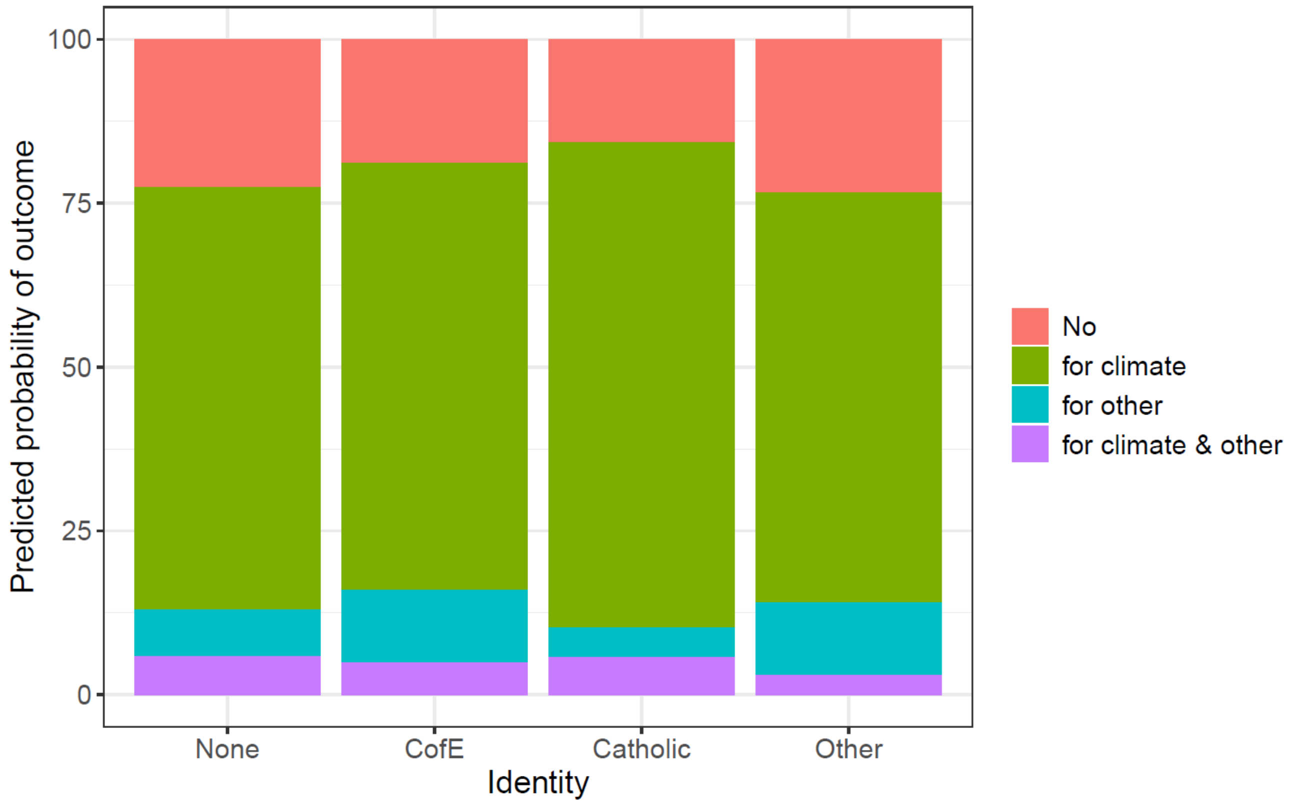

Figure S215: Results of the offspring multinomial regression models with ‘chosen sustainably sourced items’ as the outcome for four religious exposures (belief [*n* = 1,094], identity [*n* = 1,093], attendance [*n* = 1,086], and latent classes [*n* = 1,043]; models are separated by dashed horizontal lines). See table S43 for full results.

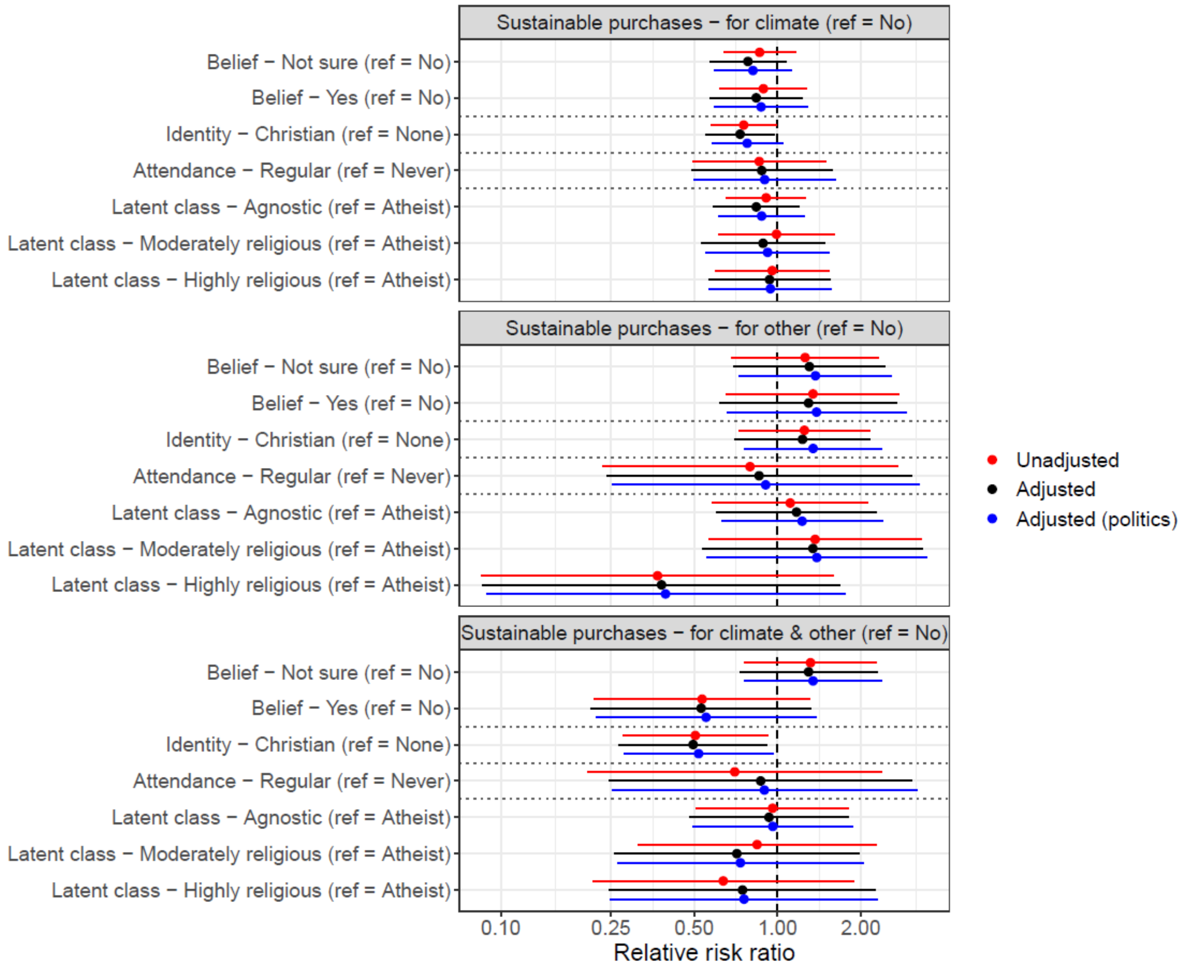

Figure S216: Predicted probabilities of the offspring multinomial regression models with 'chosen sustainably sourced items' as the outcome for four religious exposures (belief, identity, attendance and latent classes). Results are for the adjusted models excluding political ideology (results including political ideology are practically identical).

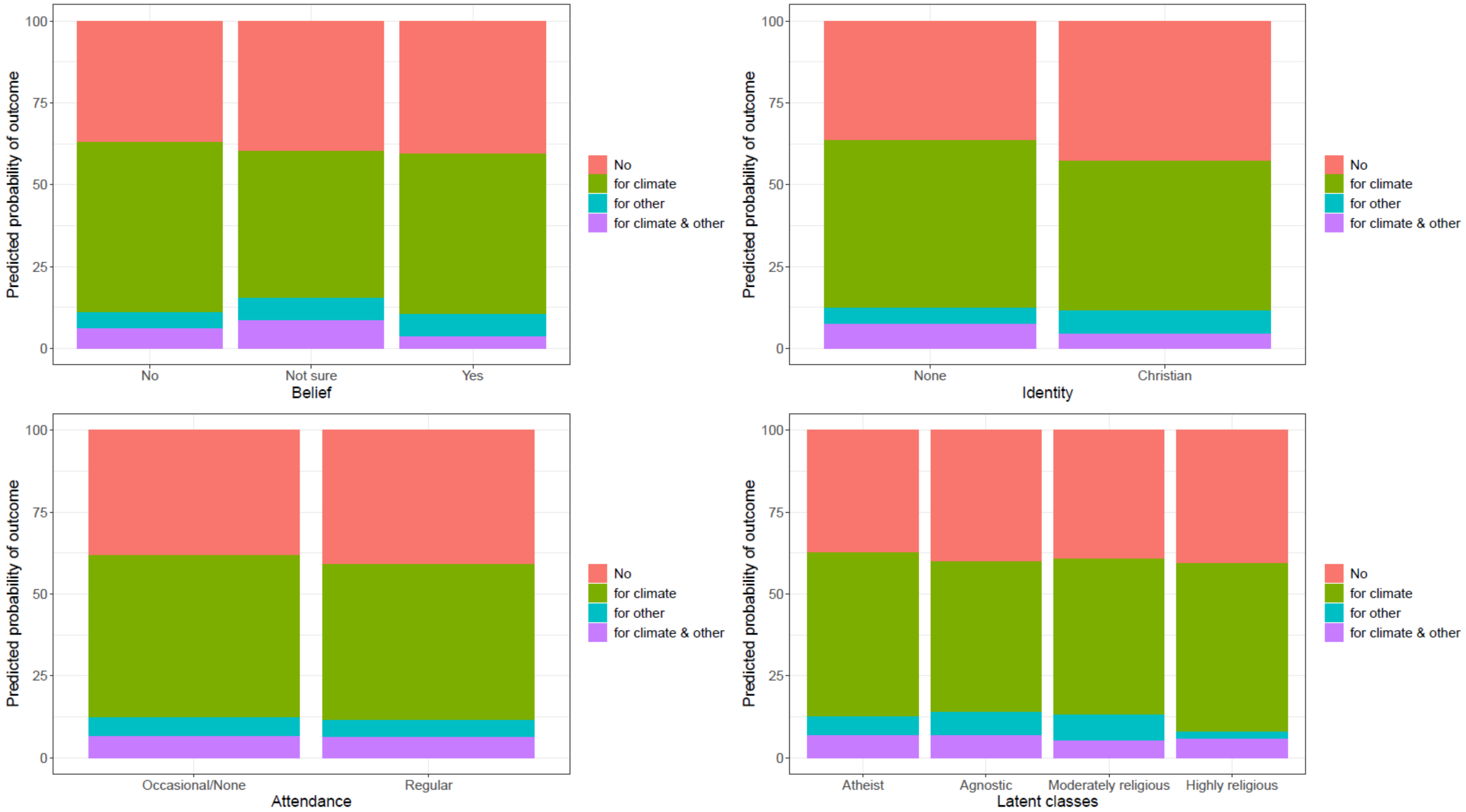

Figure S217: Predicted probabilities of the offspring multinomial regression models with 'chosen sustainably sourced items' as the outcome and the religious identity (with the Christian denominations separated) as the exposure. Results are for the adjusted models excluding political ideology (results including political ideology are practically identical).

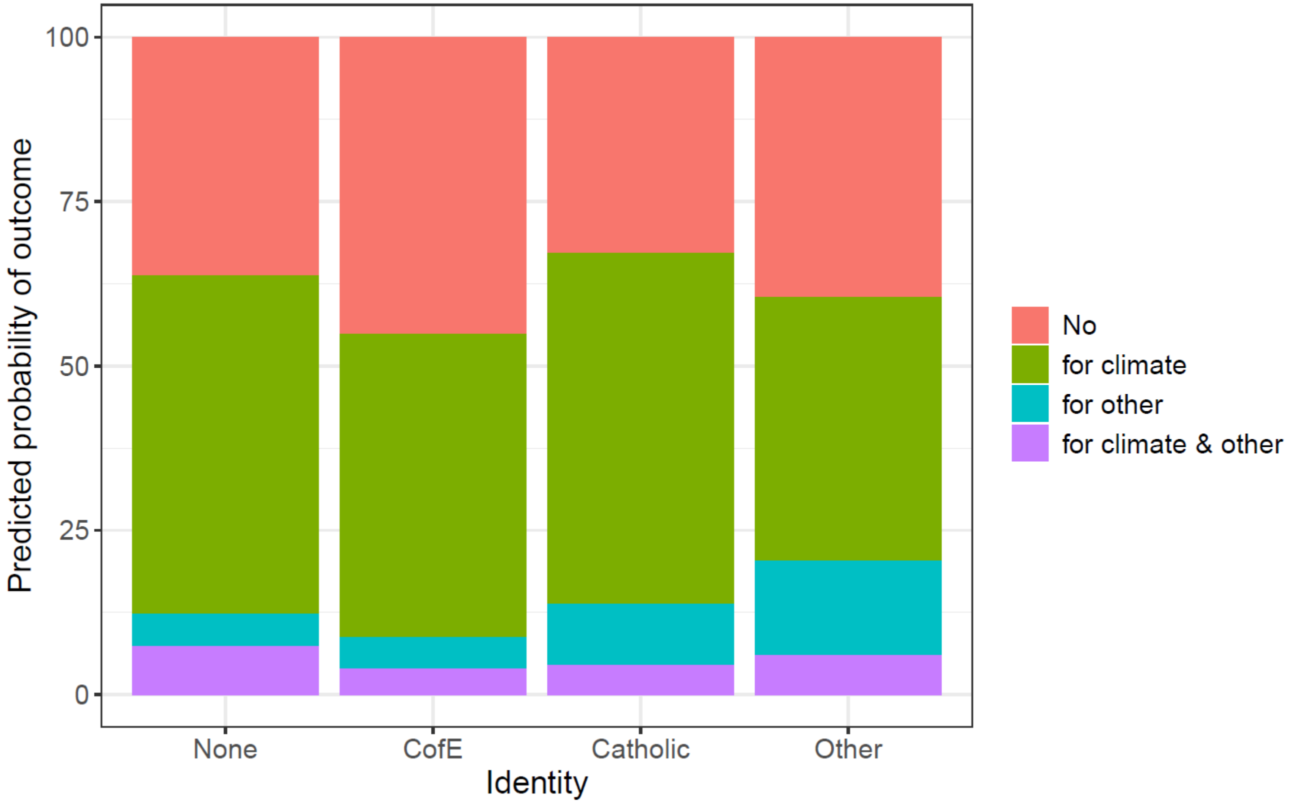

Figure S218: Results of the offspring multinomial regression models with 'improved home insulation' as the outcome for four religious exposures (belief [*n* = 1,099], identity [*n* = 1,098], attendance [*n* = 1,090], and latent classes [*n* = 1,047]; models are separated by dashed horizontal lines). See table S43 for full results.

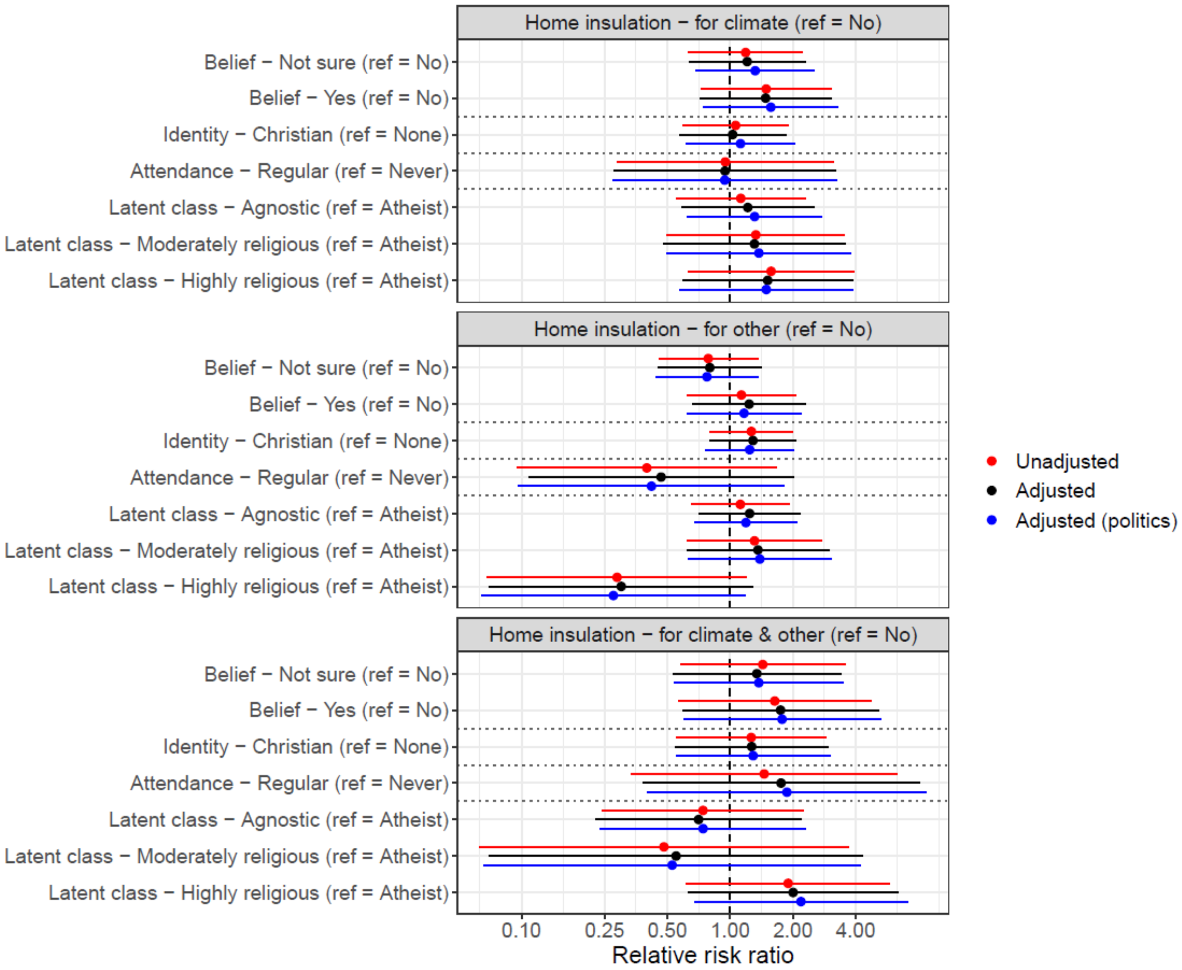

Figure S219: Predicted probabilities of the offspring multinomial regression models with 'improved home insulation' as the outcome for four religious exposures (belief, identity, attendance and latent classes). Results are for the adjusted models excluding political ideology (results including political ideology are practically identical).

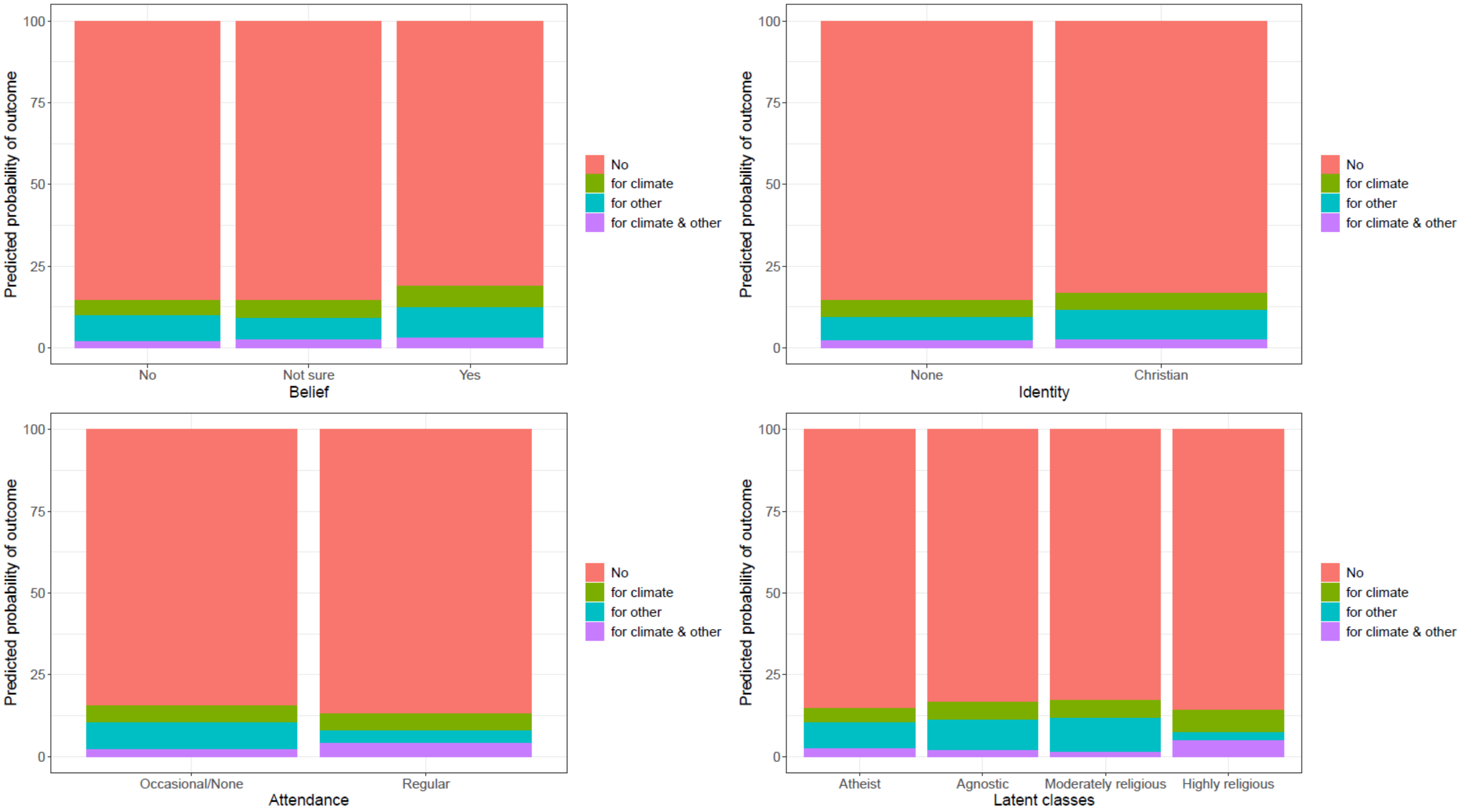

Figure S220: Predicted probabilities of the offspring multinomial regression models with 'improved home insulation' as the outcome and the religious identity (with the Christian denominations separated) as the exposure. Results are for the adjusted models excluding political ideology (results including political ideology are practically identical).

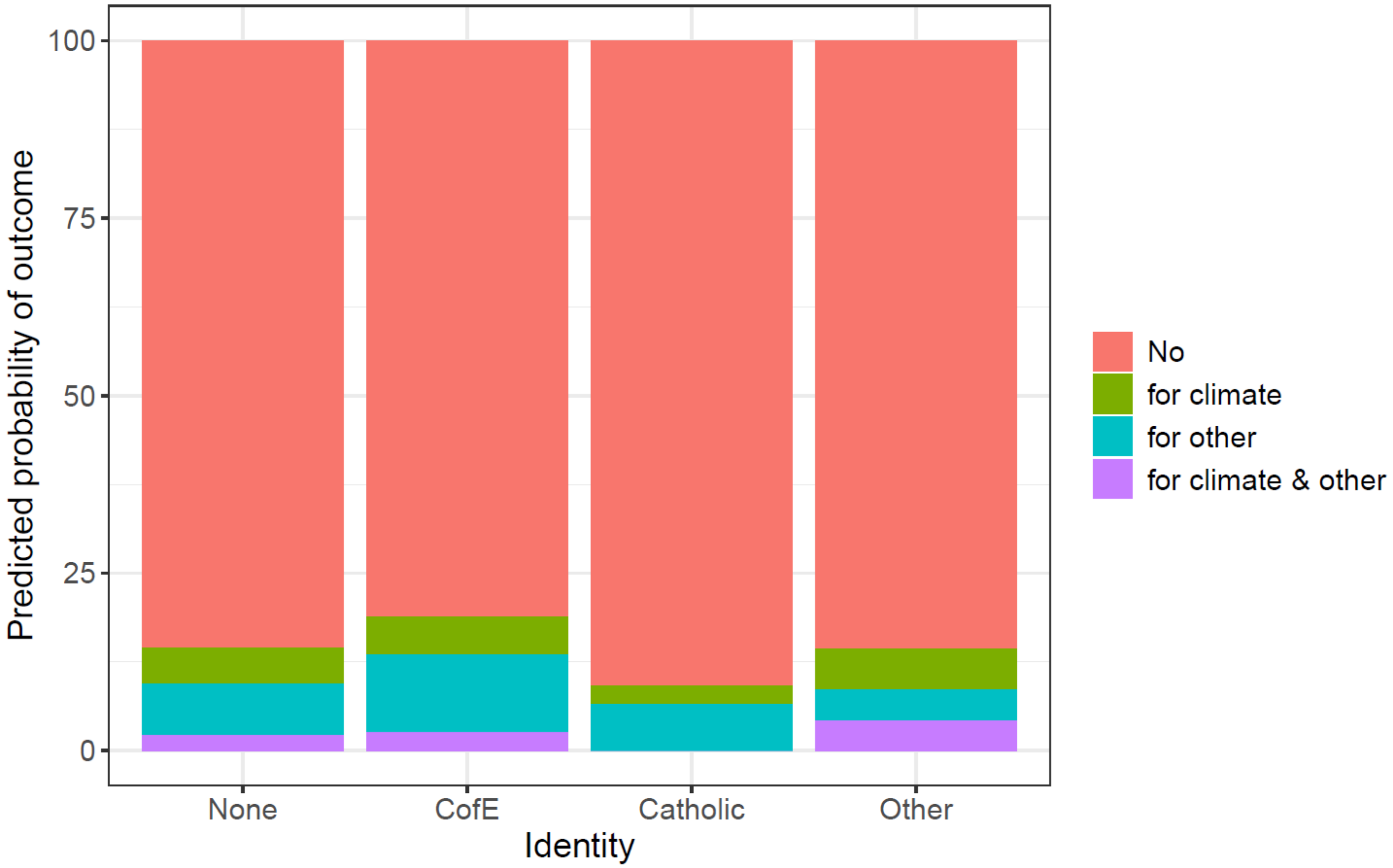

Figure S221: Results of the offspring multinomial regression models with 'started growing vegetables' as the outcome for four religious exposures (belief [*n* = 1,096], identity [*n* = 1,095], attendance [*n* = 1,087], and latent classes [*n* = 1,044]; models are separated by dashed horizontal lines). See table S43 for full results.

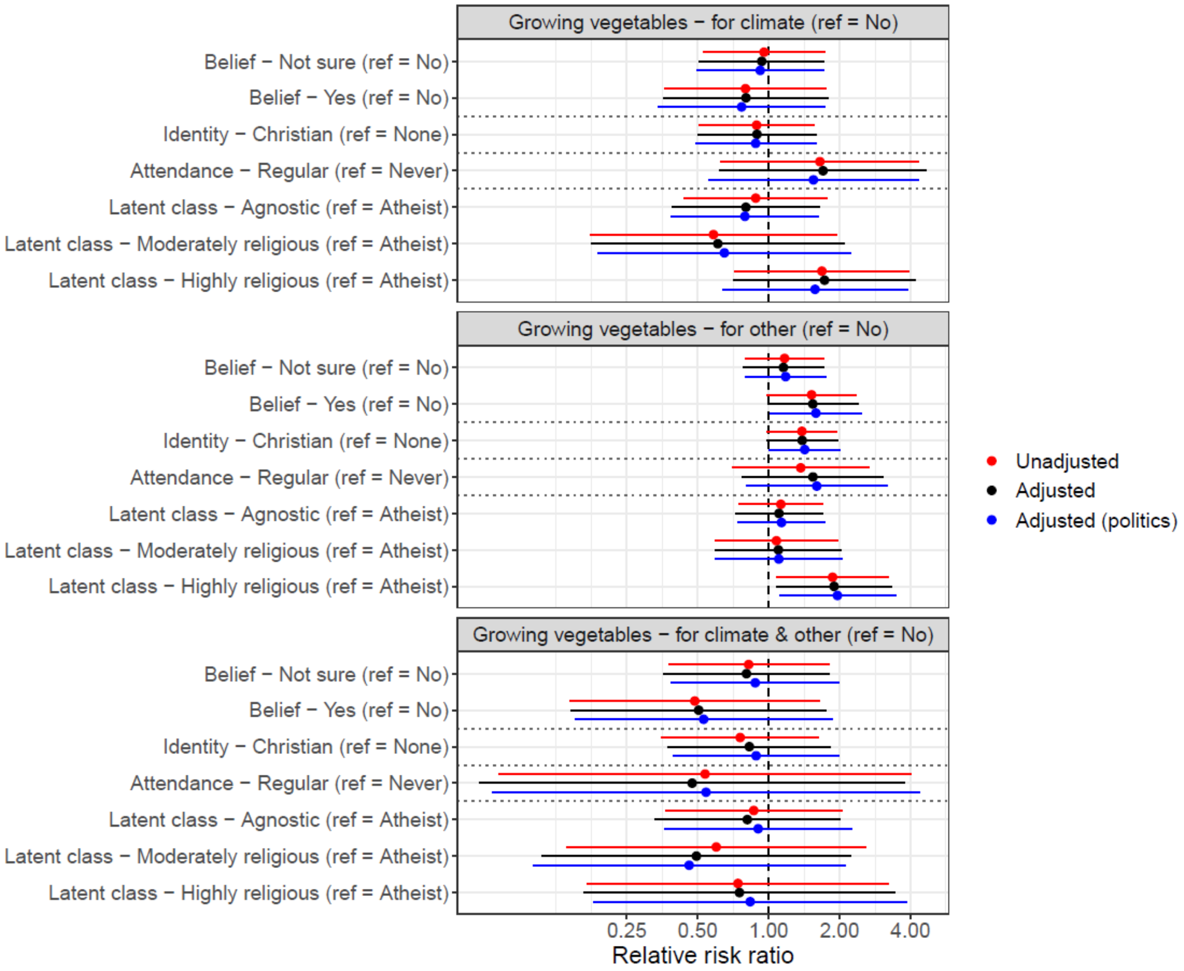

Figure S222: Predicted probabilities of the offspring multinomial regression models with 'started growing vegetables' as the outcome for four religious exposures (belief, identity, attendance and latent classes). Results are for the adjusted models excluding political ideology (results including political ideology are practically identical).

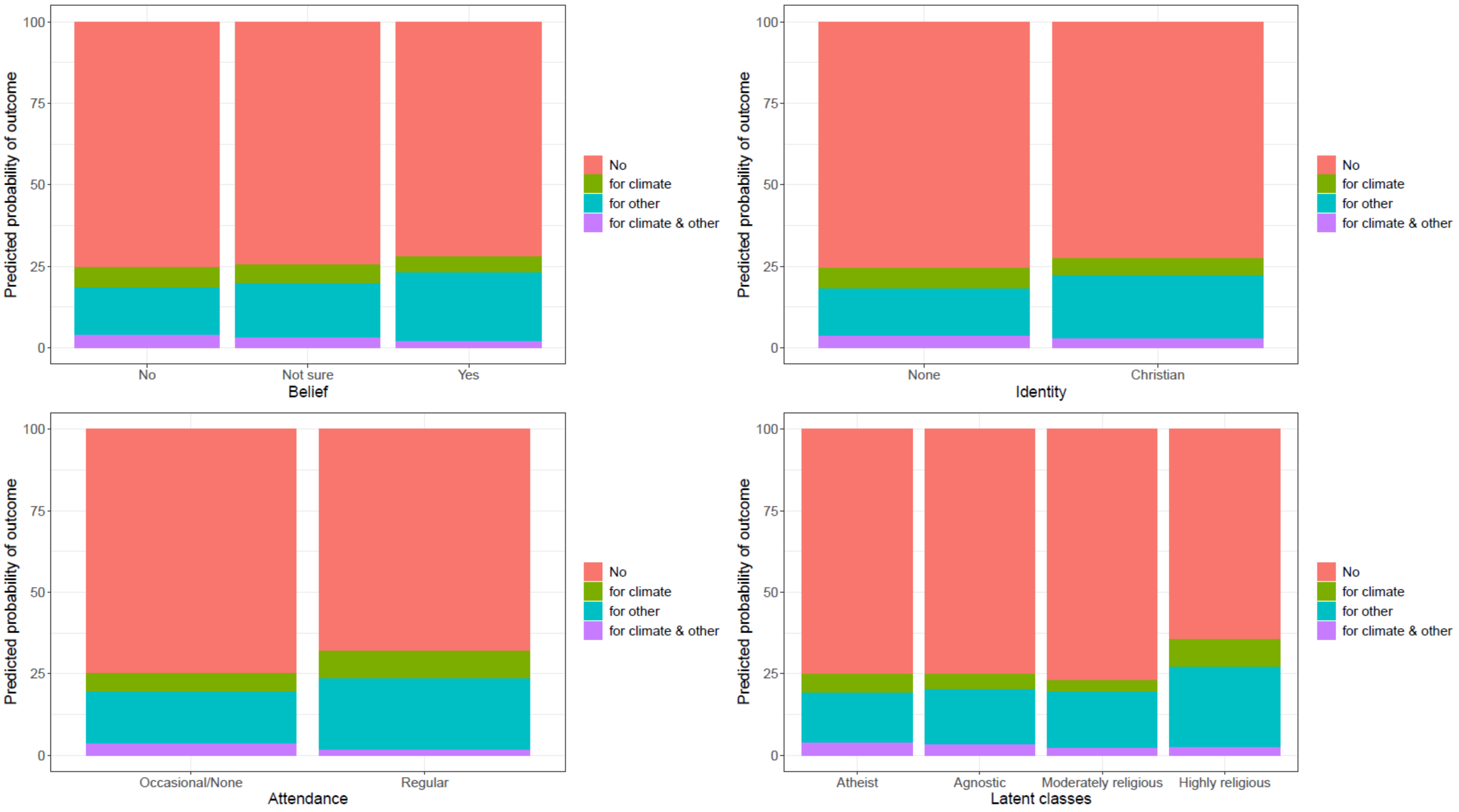

Figure S223: Predicted probabilities of the offspring multinomial regression models with 'started growing vegetables' as the outcome and the religious identity (with the Christian denominations separated) as the exposure. Results are for the adjusted models excluding political ideology (results including political ideology are practically identical).

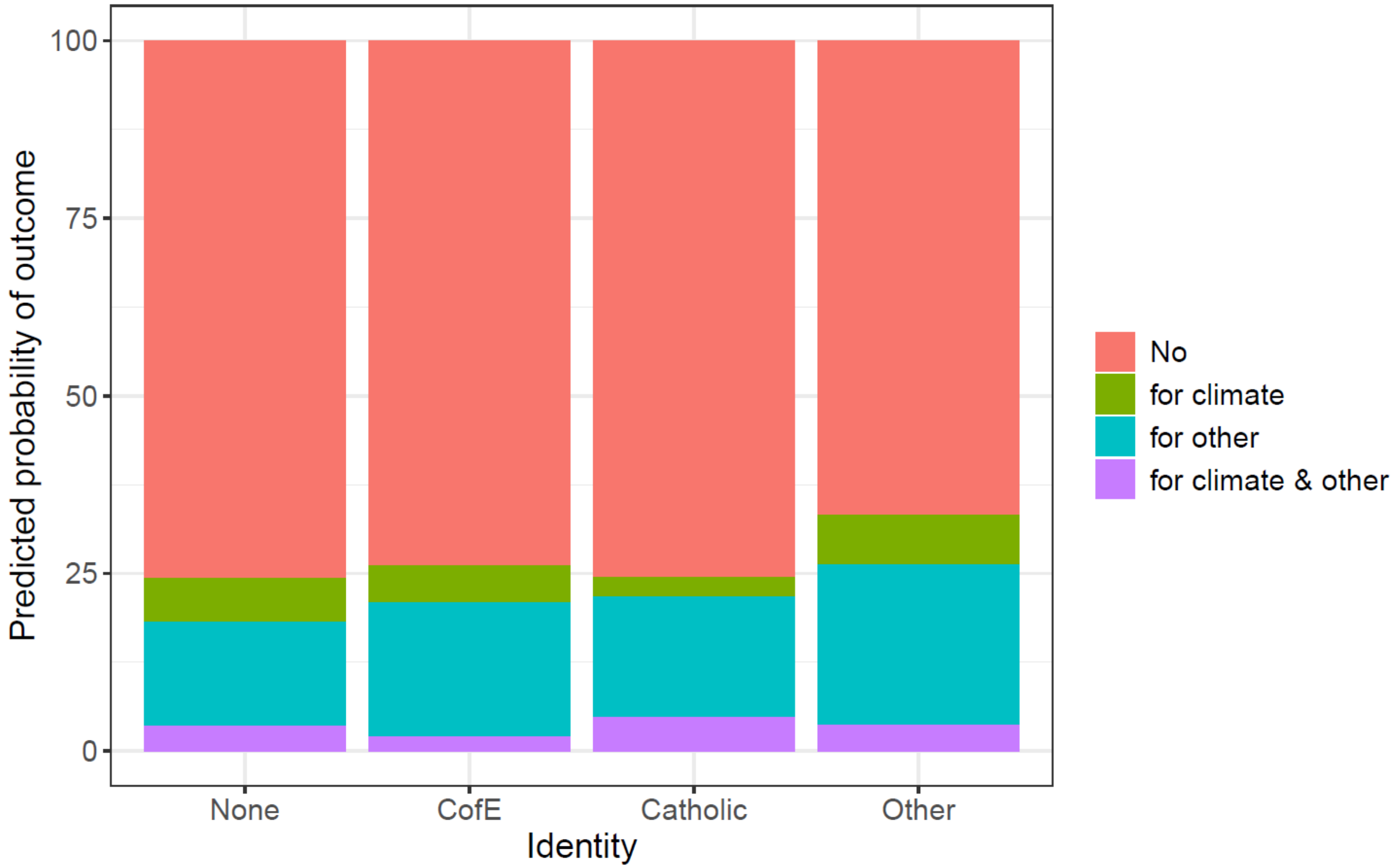

Figure S224: Results of the offspring multinomial regression models with 'planted trees' as the outcome for four religious exposures (belief [*n* = 1,095], identity [*n* = 1,094], attendance [*n* = 1,086], and latent classes [*n* = 1,044]; models are separated by dashed horizontal lines). See table S43 for full results.

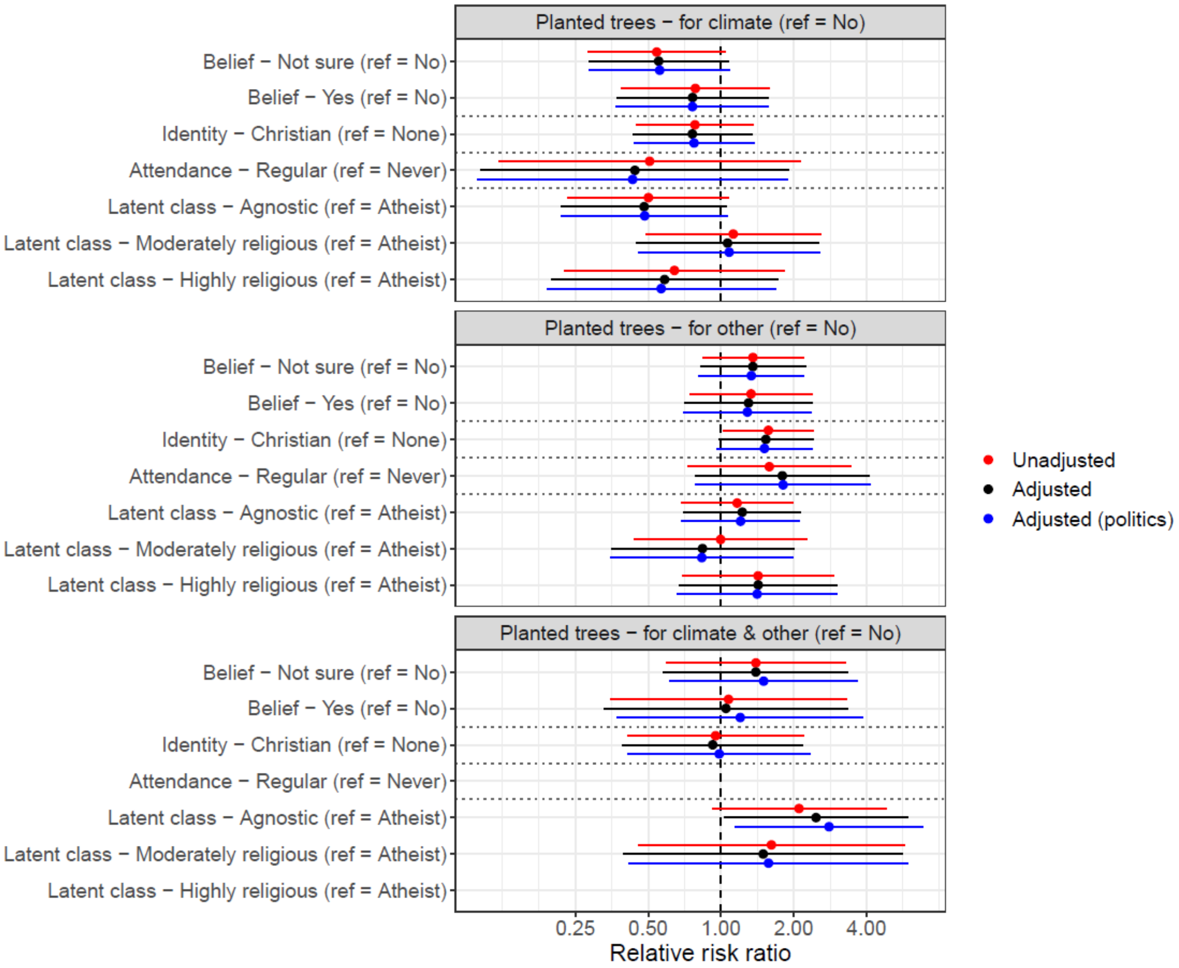

Figure S225: Predicted probabilities of the offspring multinomial regression models with 'planted trees' as the outcome for four religious exposures (belief, identity, attendance and latent classes). Results are for the adjusted models excluding political ideology (results including political ideology are practically identical).

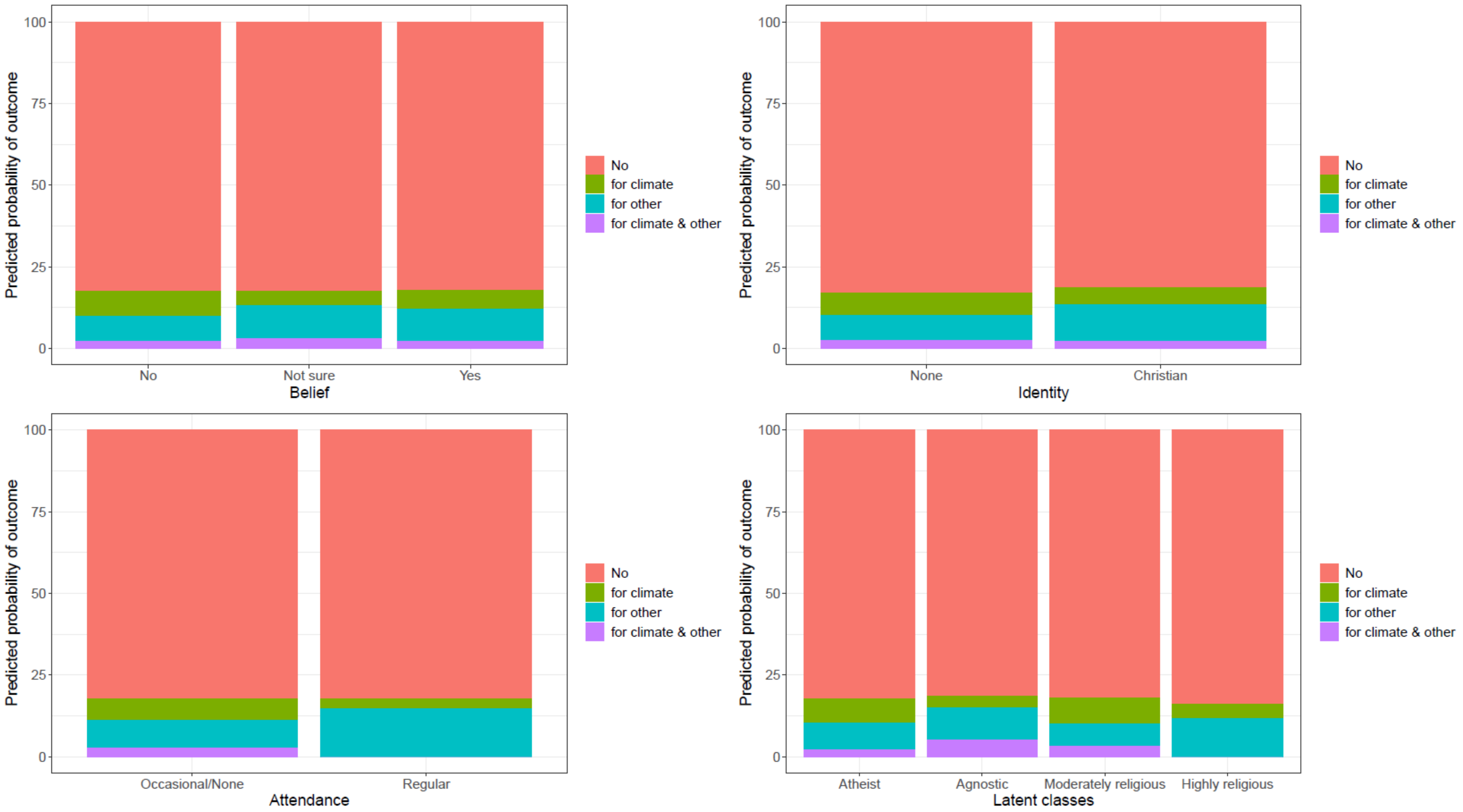

Figure S226: Predicted probabilities of the offspring multinomial regression models with 'planted trees' as the outcome and the religious identity (with the Christian denominations separated) as the exposure. Results are for the adjusted models excluding political ideology (results including political ideology are practically identical).

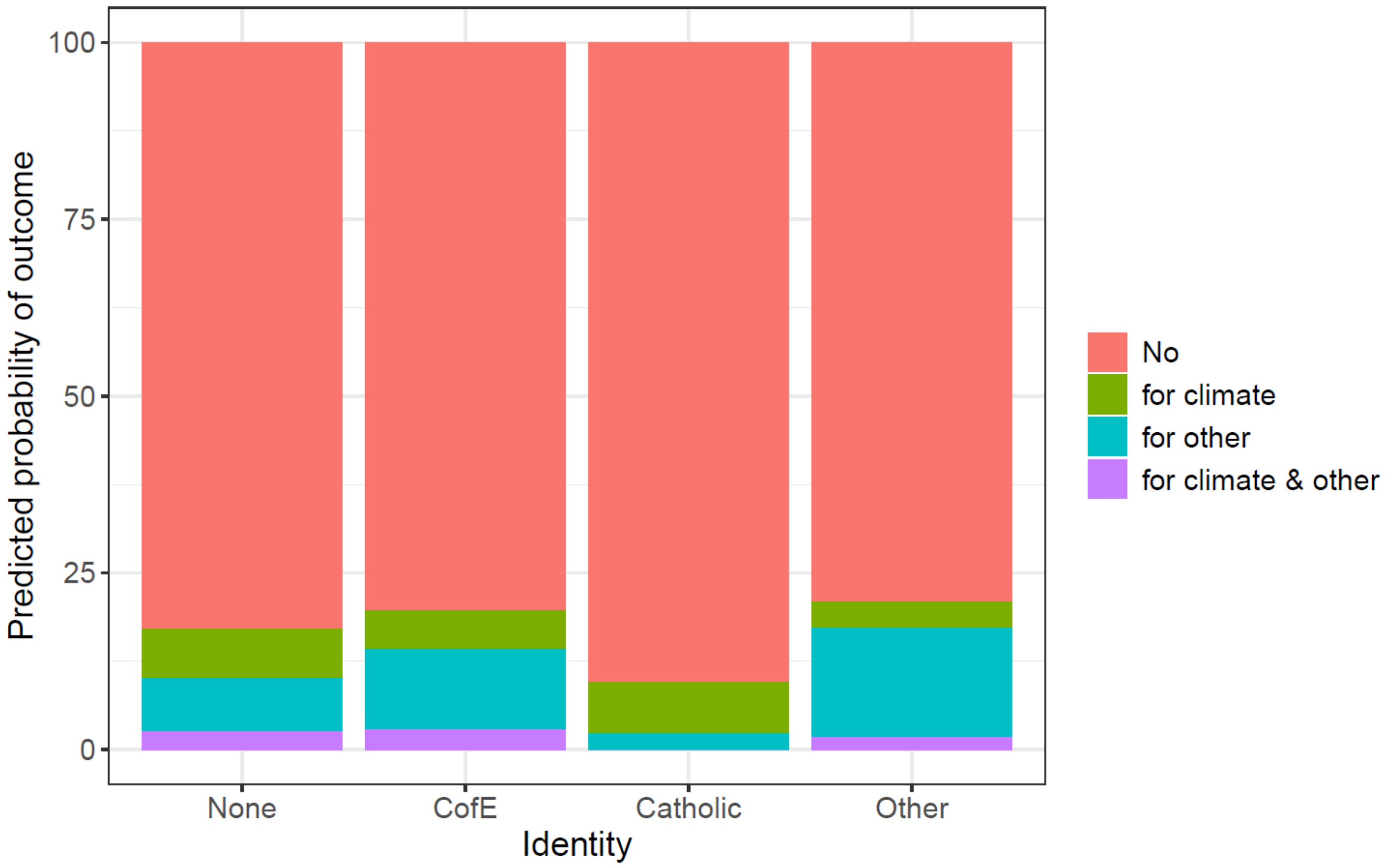

Figure S227: Results of the offspring multinomial regression models with 'avoided organisations that support fossil fuels' as the outcome for four religious exposures (belief [*n* = 1,097], identity [*n* = 1,096], attendance [*n* = 1,088], and latent classes [*n* = 1,045]; models are separated by dashed horizontal lines). See table S43 for full results.

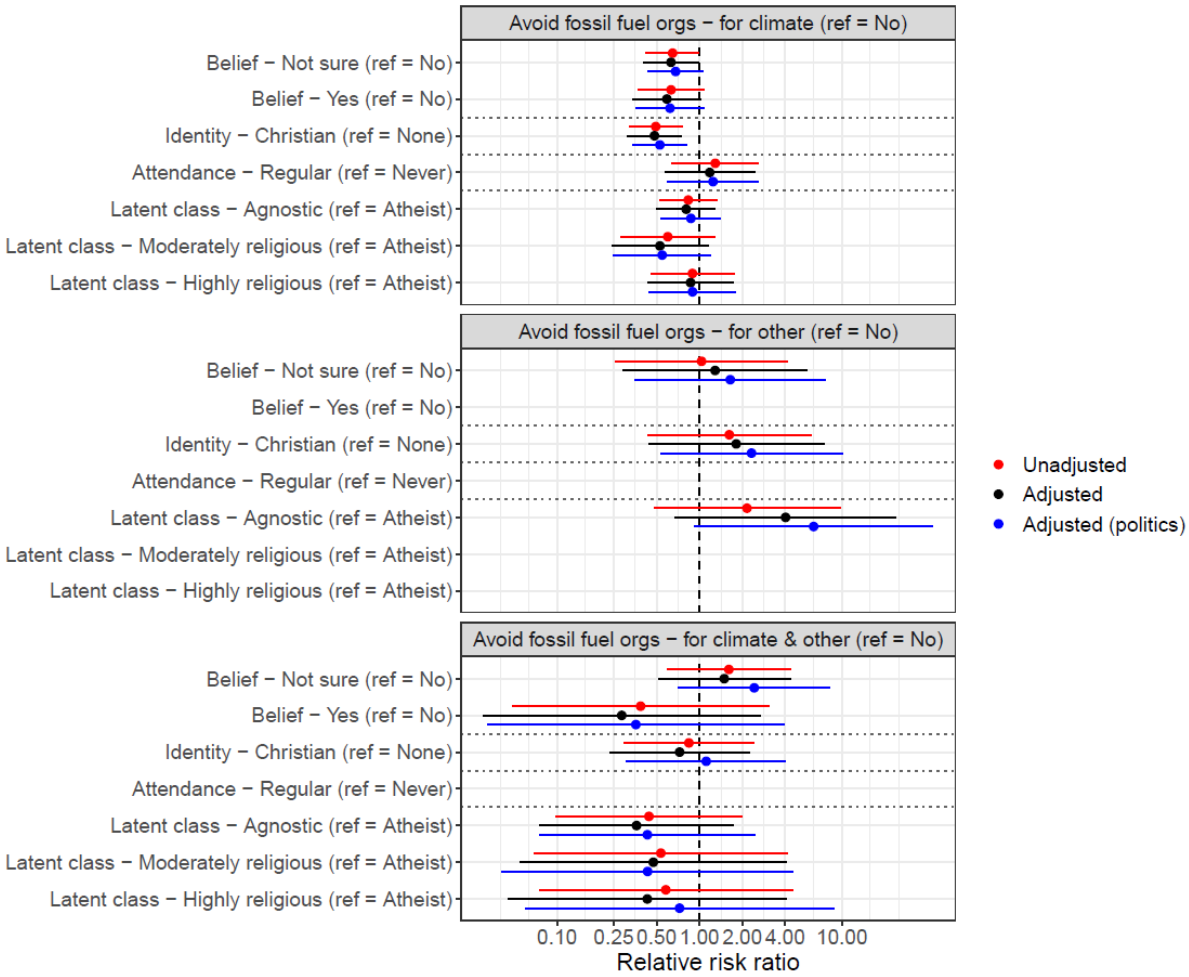

Figure S228: Predicted probabilities of the offspring multinomial regression models with 'avoided organisations that support fossil fuels' as the outcome for four religious exposures (belief, identity, attendance and latent classes). Results are for the adjusted models excluding political ideology (results including political ideology are practically identical).

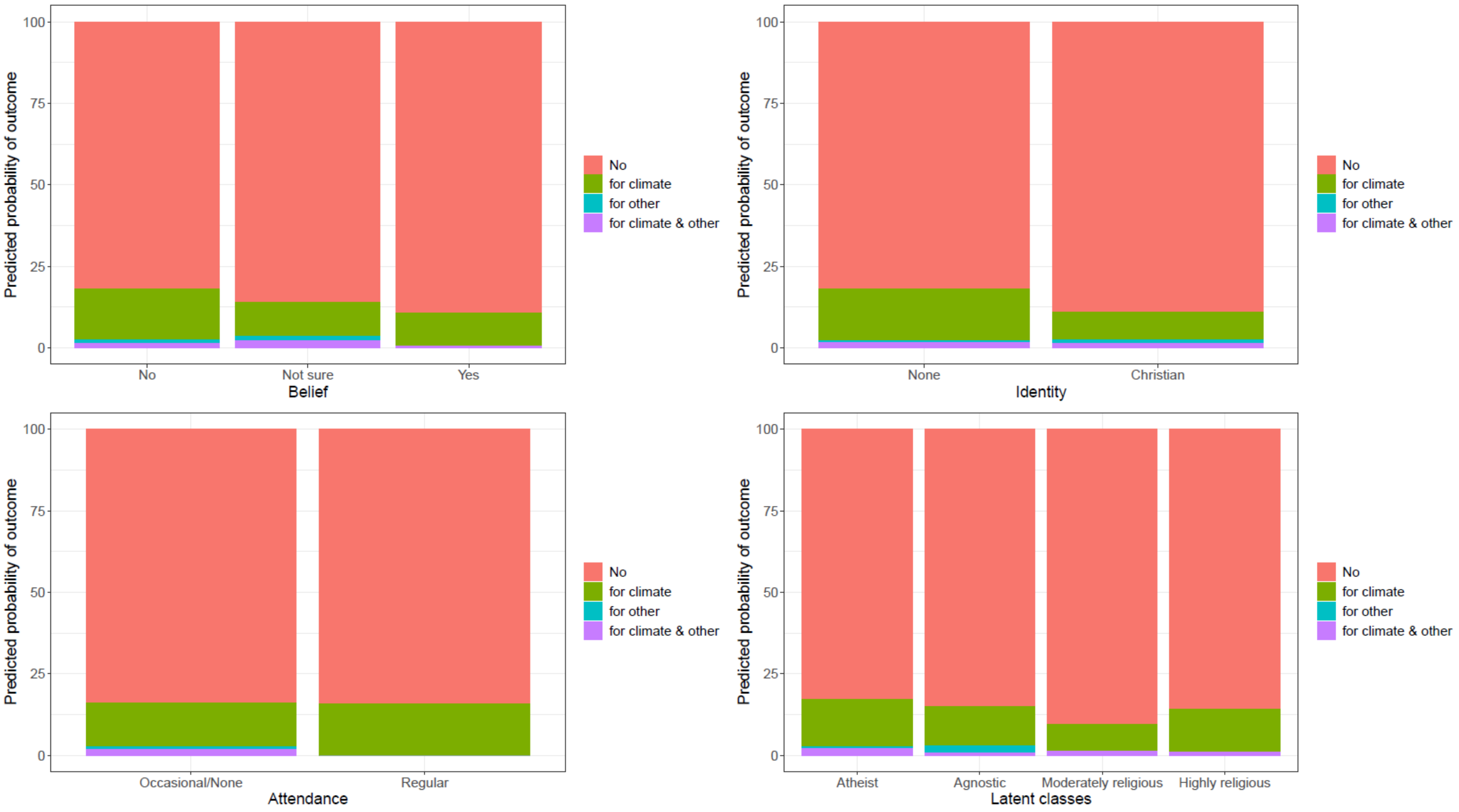

Figure S229: Predicted probabilities of the offspring multinomial regression models with 'avoided organisations that support fossil fuels' as the outcome and the religious identity (with the Christian denominations separated) as the exposure. Results are for the adjusted models excluding political ideology (results including political ideology are practically identical).

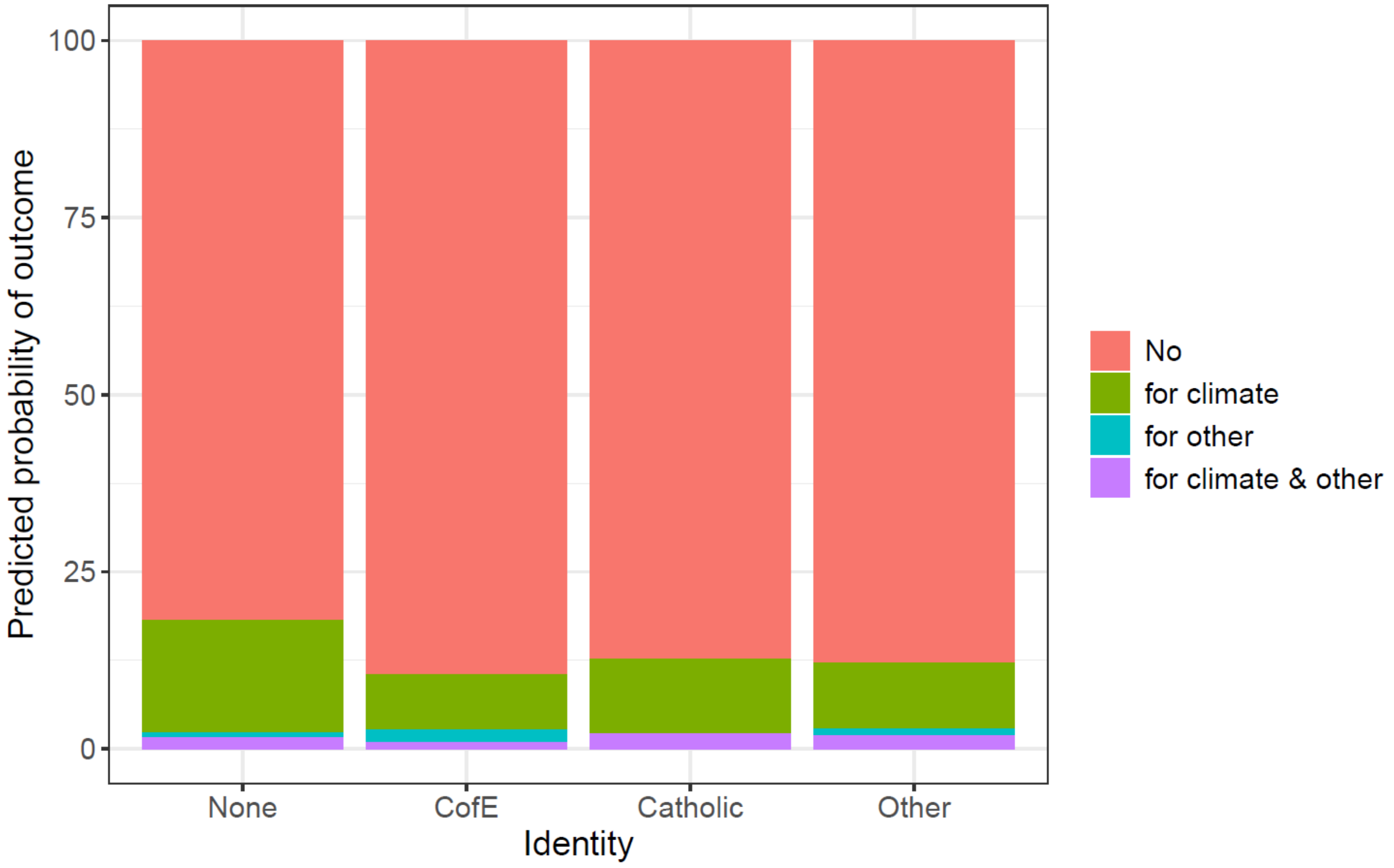

Figure S230: Results of the offspring multinomial regression models with 'chosen to have fewer or no children' as the outcome for four religious exposures (belief [*n* = 1,093], identity [*n* = 1,092], attendance [*n* = 1,084], and latent classes [*n* = 1,043]; models are separated by dashed horizontal lines). See table S43 for full results.

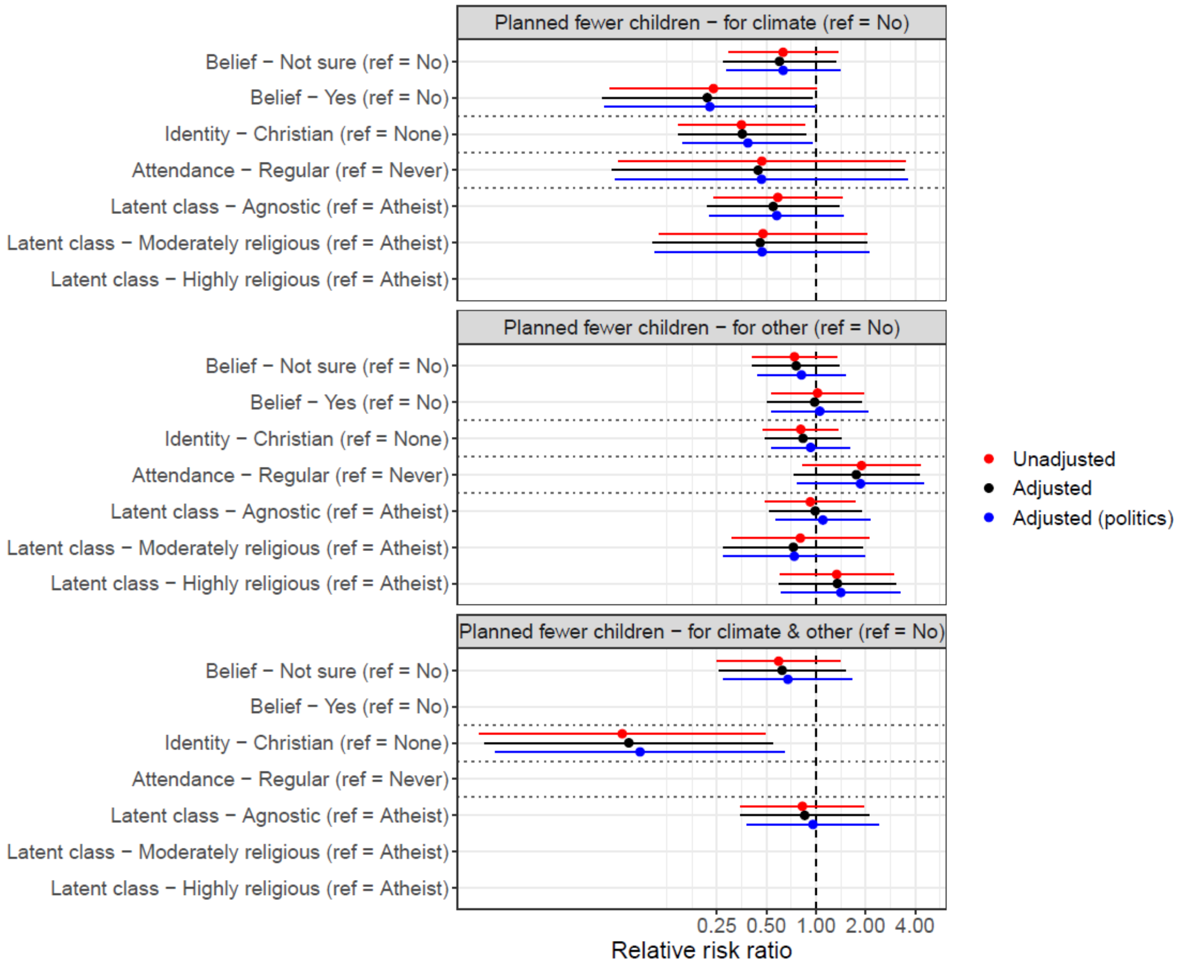

Figure S231: Predicted probabilities of the offspring multinomial regression models with 'chosen to have fewer or no children' as the outcome for four religious exposures (belief, identity, attendance and latent classes). Results are for the adjusted models excluding political ideology (results including political ideology are practically identical).

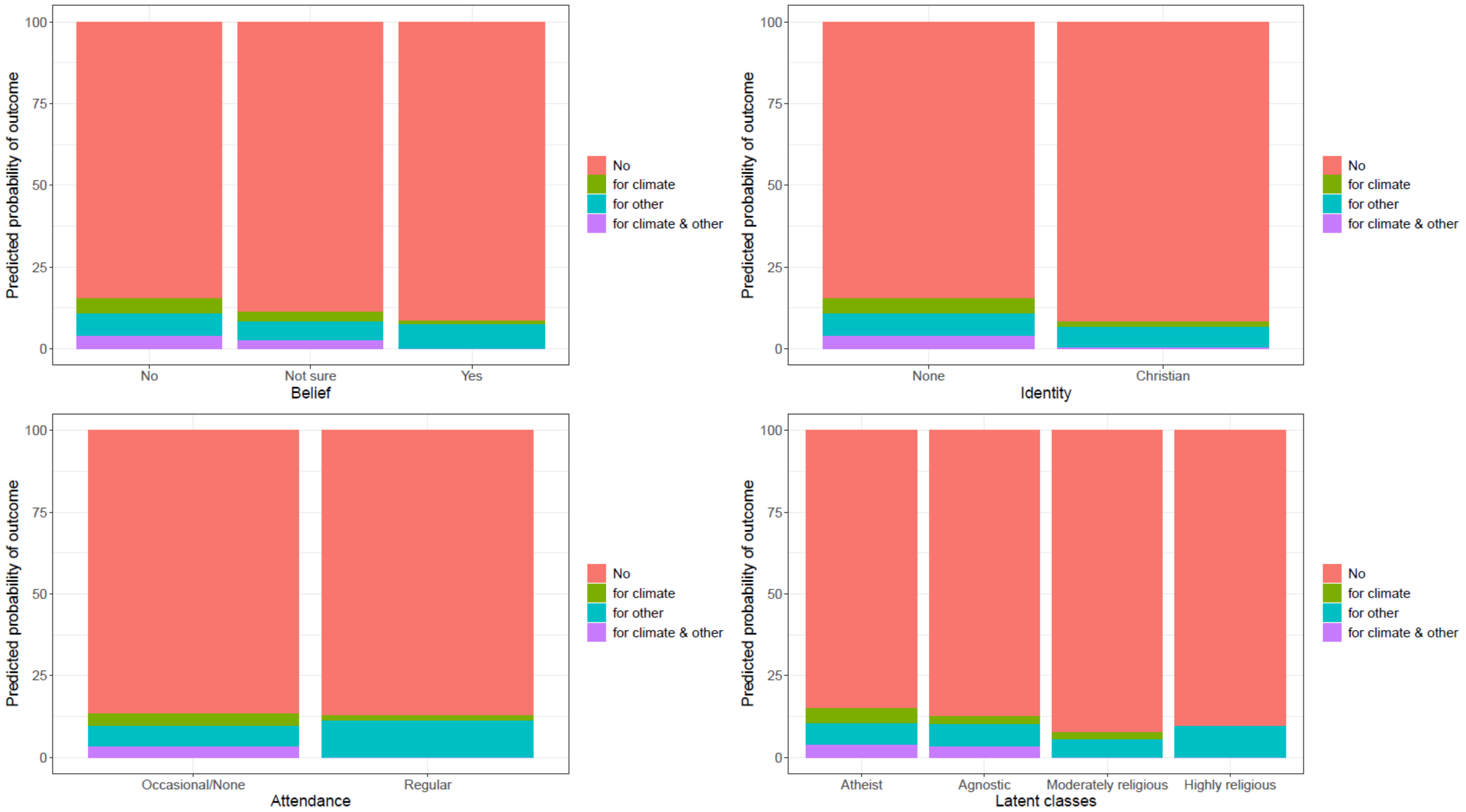

Figure S232: Predicted probabilities of the offspring multinomial regression models with 'chosen to have fewer or no children' as the outcome and the religious identity (with the Christian denominations separated) as the exposure. Results are for the adjusted models excluding political ideology (results including political ideology are practically identical).

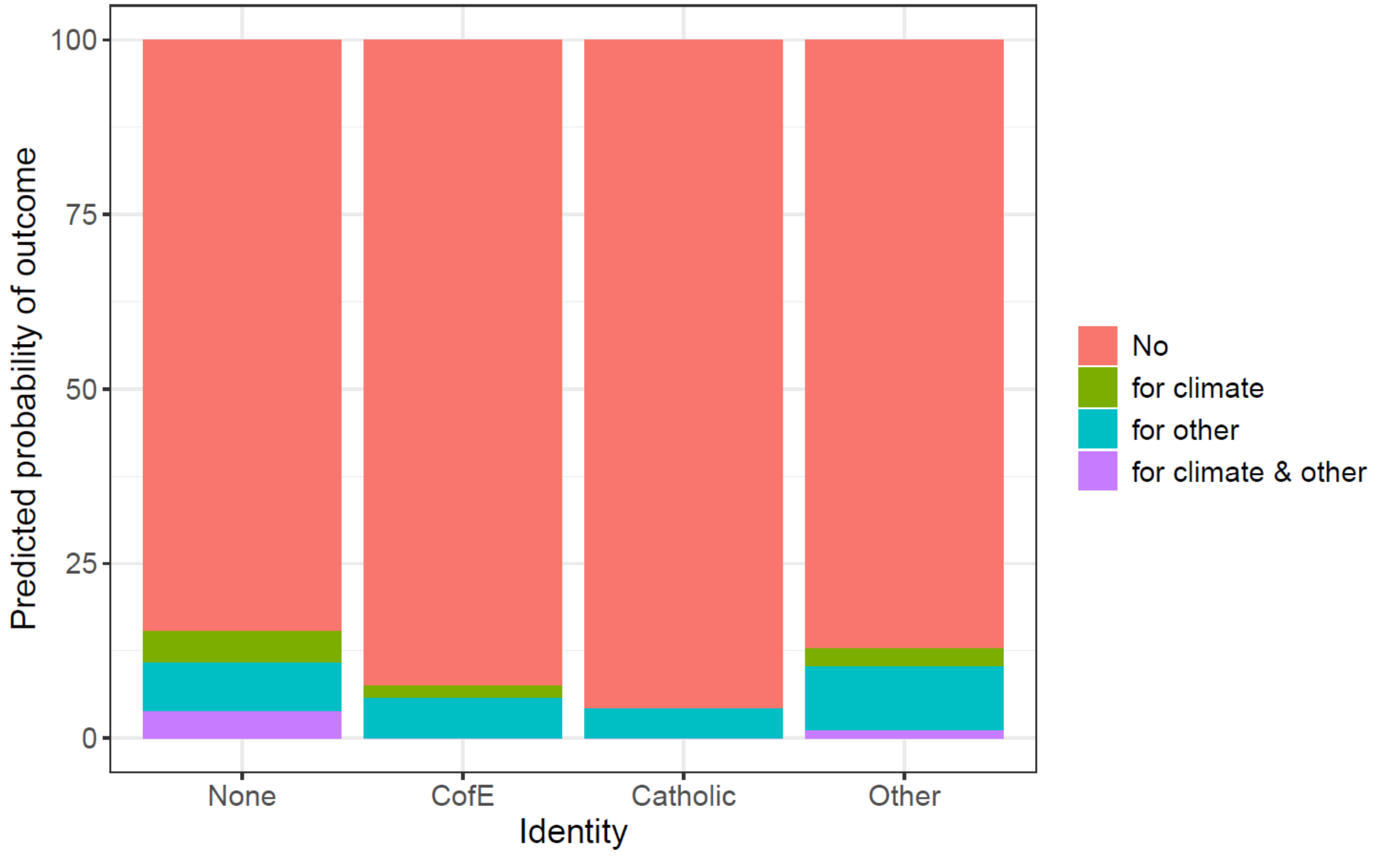

Figure S233: Results of the offspring multinomial regression models with 'taken action to eat less or no meat and/or dairy' as the outcome for four religious exposures (belief [*n* = 1,039], identity [*n* = 1,038], attendance [*n* = 1,030], and latent classes [*n* = 992]; models are separated by dashed horizontal lines). See table S43 for full results.

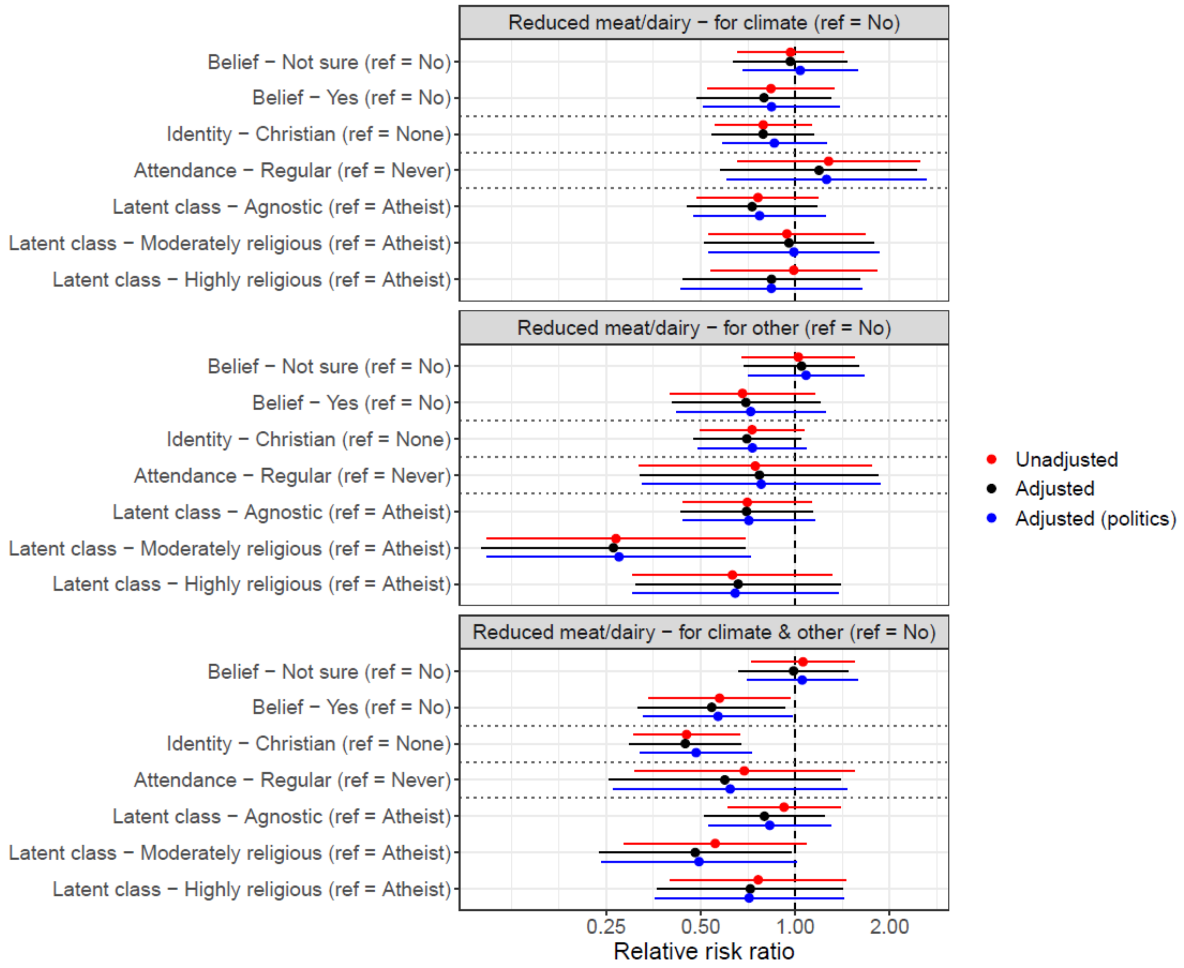

Figure S234: Predicted probabilities of the offspring multinomial regression models with ‘taken action to eat less or no meat and/or dairy’ as the outcome for four religious exposures (belief, identity, attendance and latent classes). Results are for the adjusted models excluding political ideology (results including political ideology are practically identical).

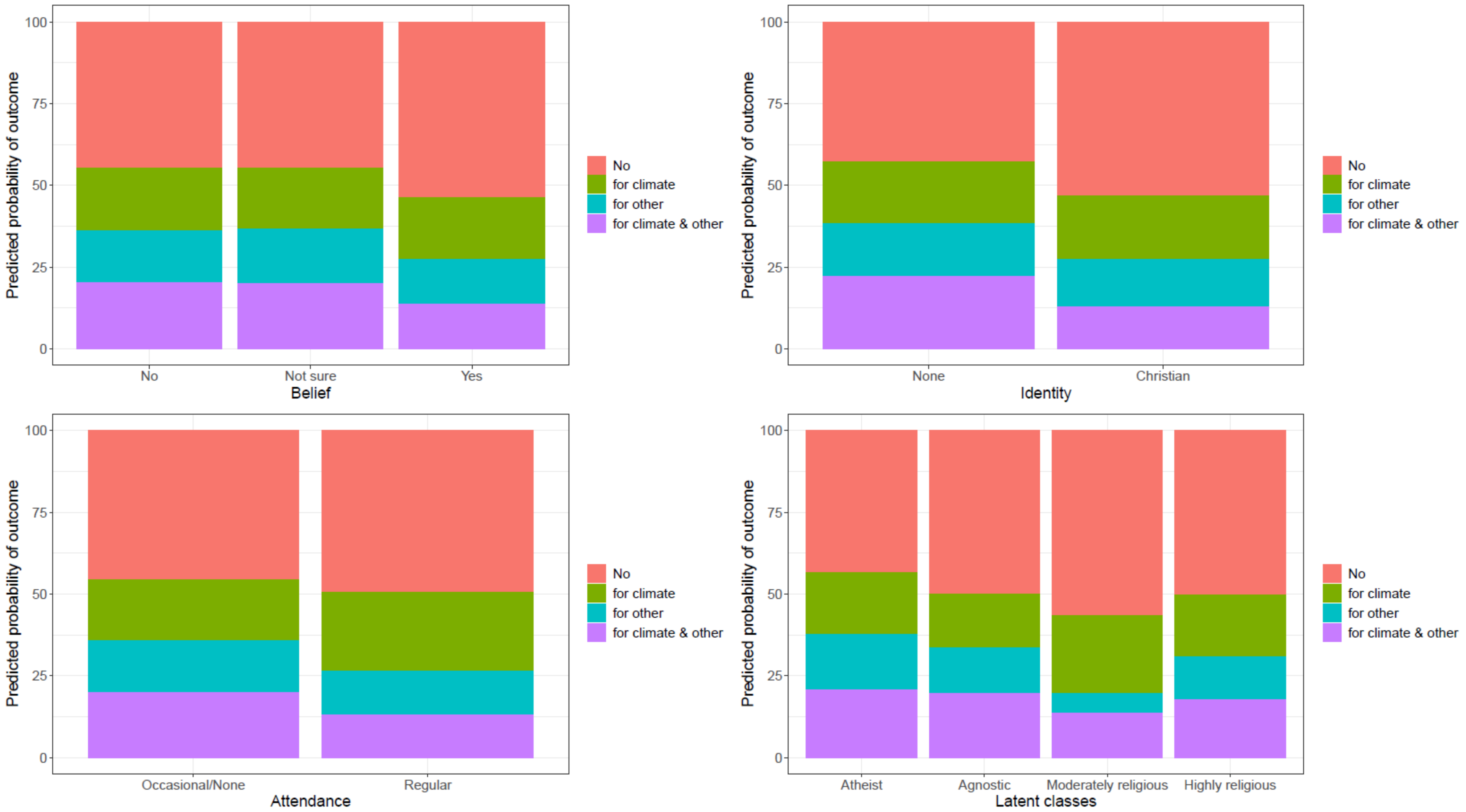

Figure S235: Predicted probabilities of the offspring multinomial regression models with 'taken action to eat less or no meat and/or dairy' as the outcome and the religious identity (with the Christian denominations separated) as the exposure. Results are for the adjusted models excluding political ideology (results including political ideology are practically identical).

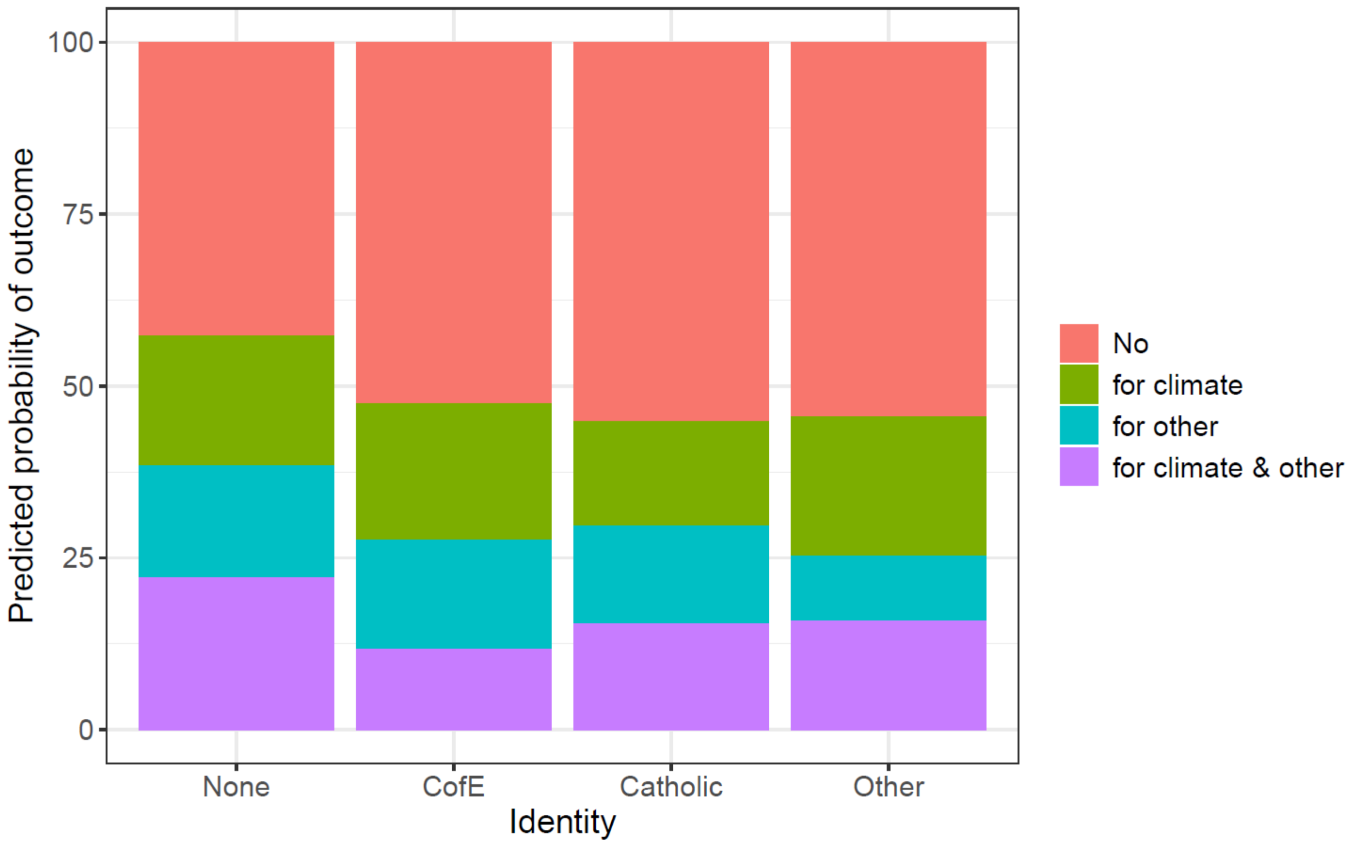

Supplement: Supplementary Information [file EMS209687-supplement-Supplementary_Information.pdf]
